# Supplementary material for: Microbiome Profiling by Illumina Sequencing of Combinatorial Sequence-Tagged PCR Products
Source: PLoS One. 2010 Oct 26;5(10):e15406. doi: 10.1371/journal.pone.0015406 (PMC2964327; doi:10.1371/journal.pone.0015406)
Supplement: Figure S1 — Plots of the sequence variation in each of the operational taxonomic units (OTUs). The plot shows the number of times that each nucleotide occurred at each position in two example OTUs. The identifier at the top gives the OTU number, followed by the skew, and values described in the text. Note that OTUs 46, 97, and 119 show sequence variation that is significantly different than the variation observed in the other OTUs for the reasons described in the text. (PDF) [file pone.0015406.s001.pdf]

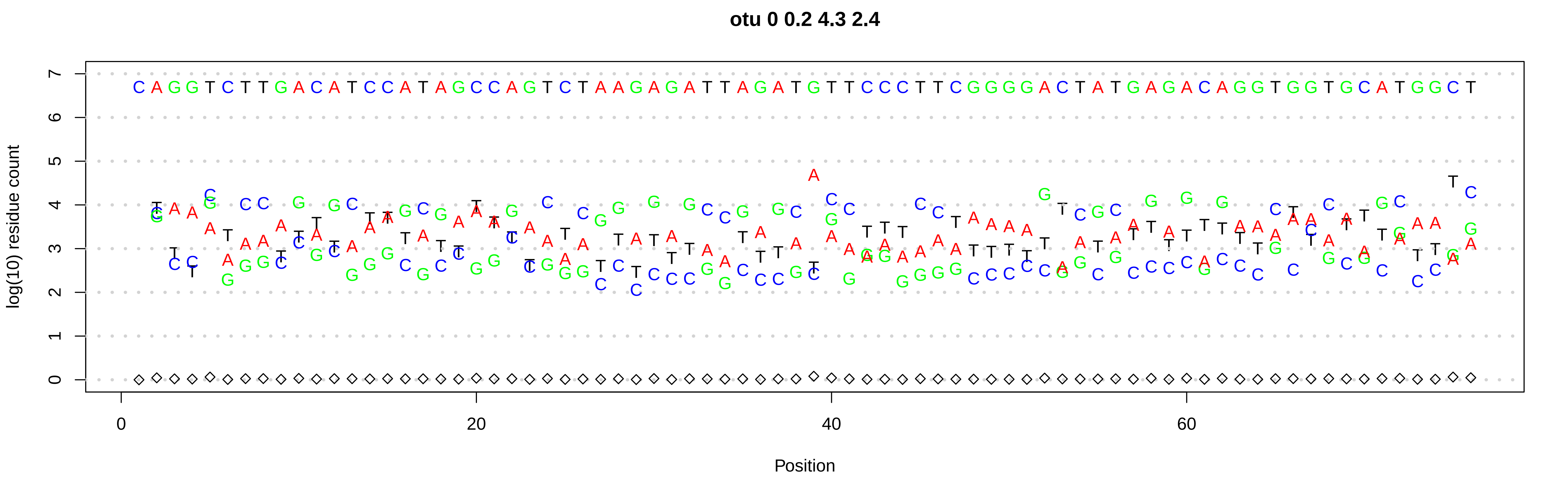
otu 0 0.2 4.3 2.4
log(10) residue count
0
1
2
3
4
5
6
7
Position
0
20
40
60
C A G G T C T T G A C A T C C A T A G C C A G T C T A A G A G A T T A G A T G T T C C C T T C G G G G A C T A T G A G A C A G G T G G T G C A T G G C T

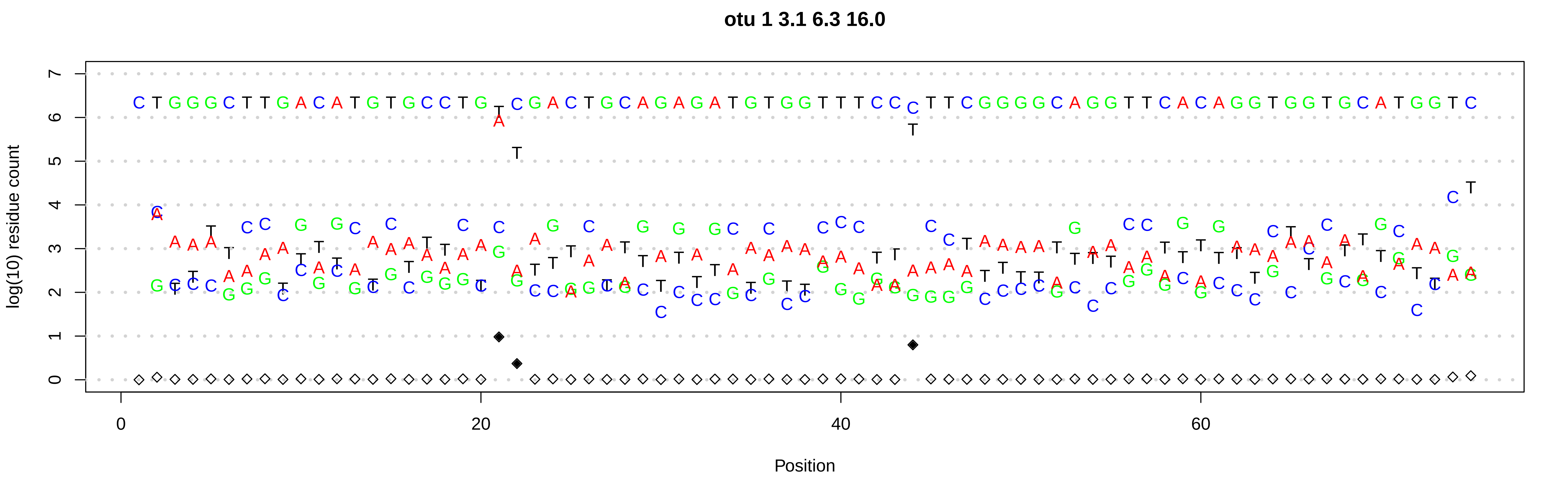

otu 1 3.1 6.3 16.0
log(10) residue count
Position
0
20
40
60
0
1
2
3
4
5
6
7

otu 2 0.4 7.5 11.3

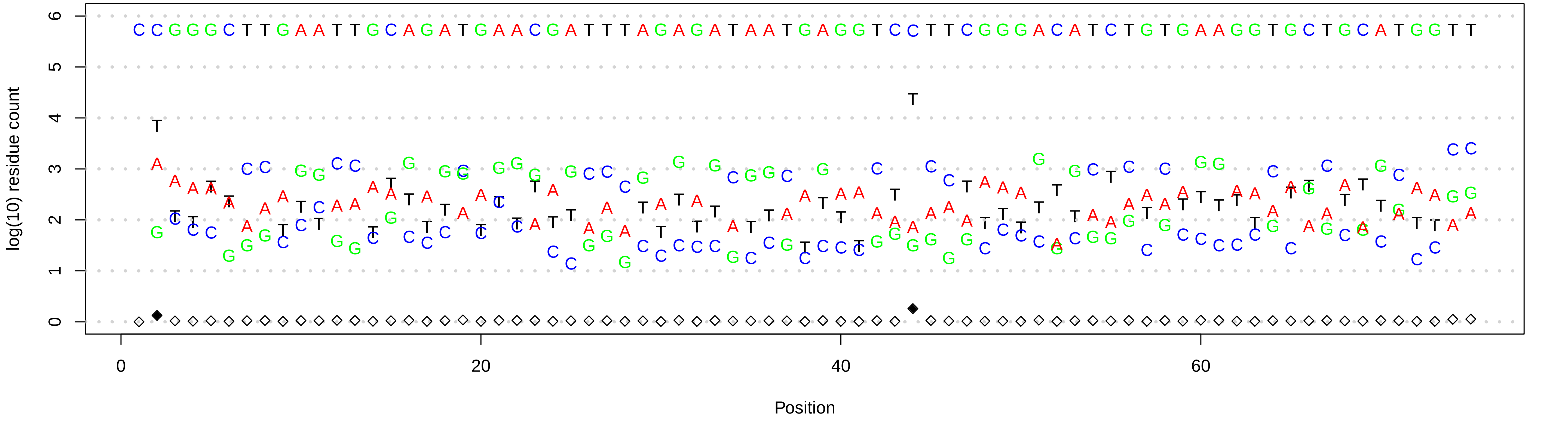

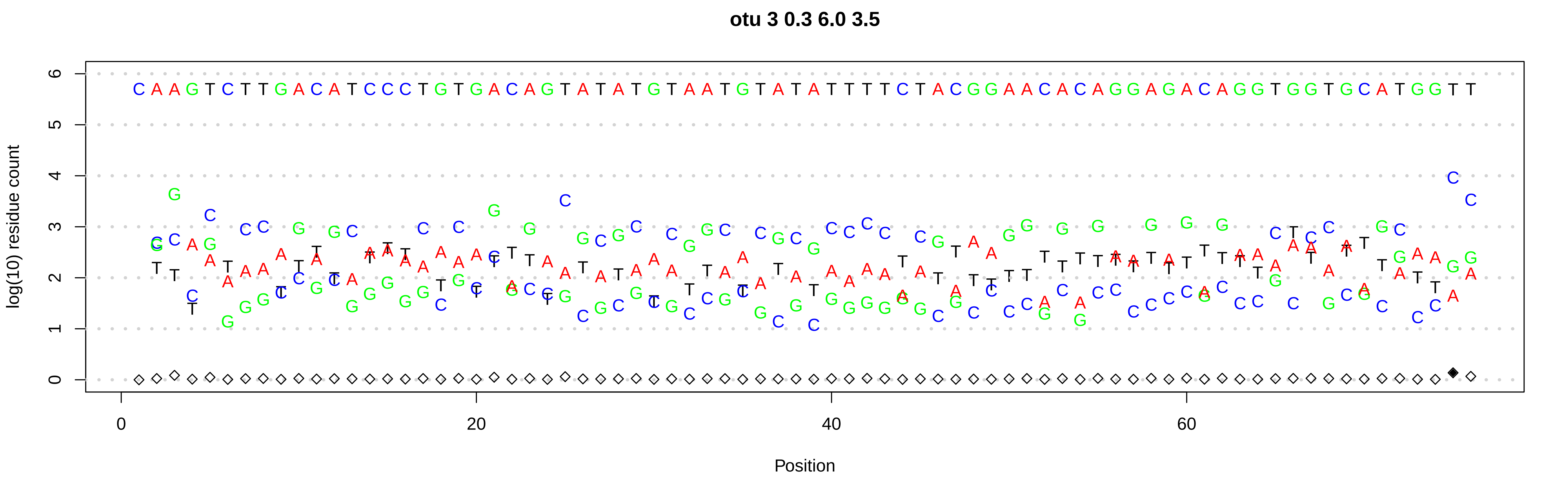
otu 3 0.3 6.0 3.5
CAAGTCTTGACATCCCTGTGACAGTATATGTAATGTATATTTTTCTACGGAACACAGGAGACAGGTGGTGCATGGTT
log(10) residue count
0
1
2
3
4
5
6
Position
0
20
40
60

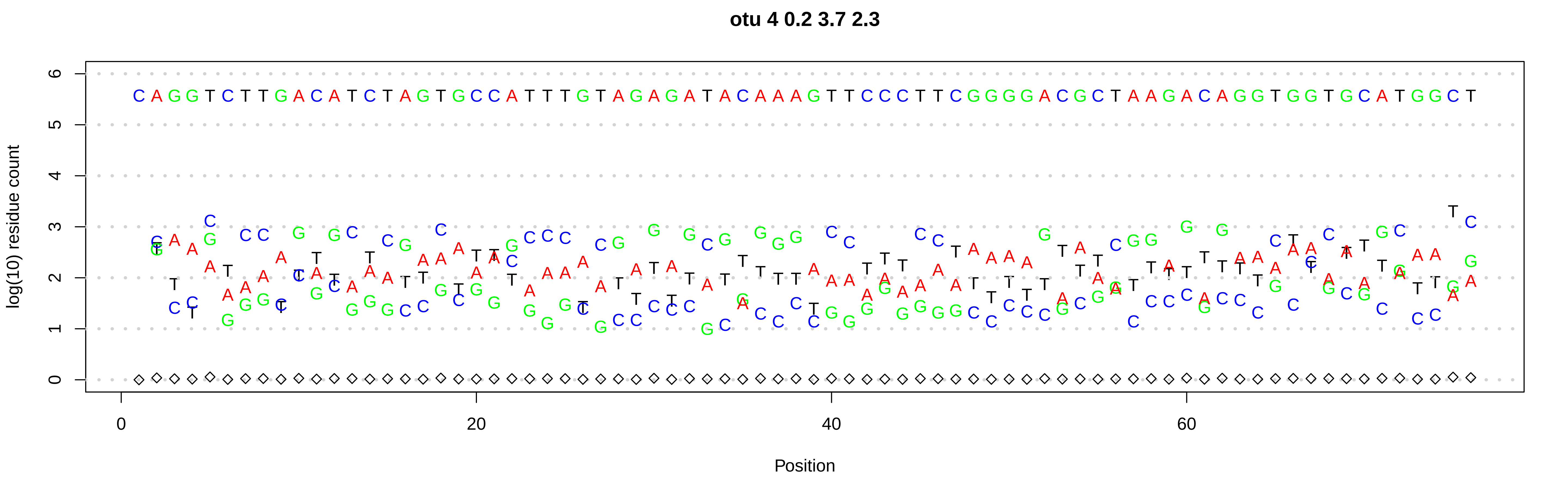
otu 4 0.2 3.7 2.3
CAGGTCTTGACATCTAGTGCCATTTGTAGAGATACAAAGTTCCCTTCGGGGACGCTAAGACAGGTGGTGCATGGCT
log(10) residue count
0
1
2
3
4
5
6
Position
0
20
40
60

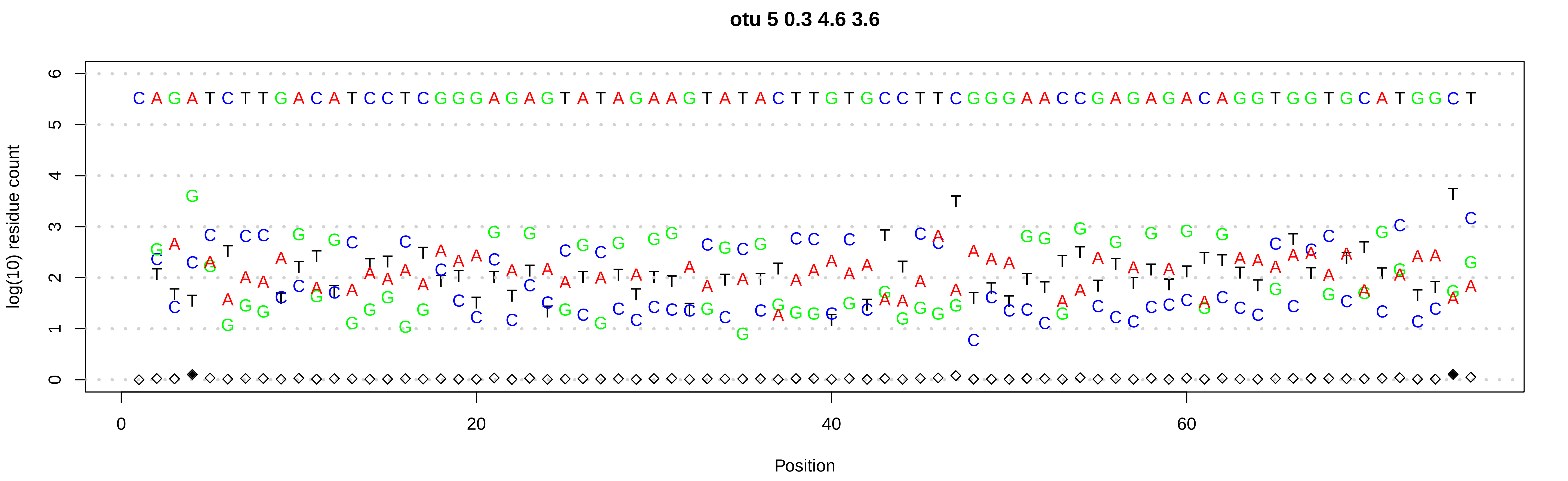

otu 5 0.3 4.6 3.6
CAGATCTTGACATCCTCGGGAGAGTATAGAAGTATACTTGTGCCTTCGGGAACCGAGAGACAGGTGGTGCATGGCT
log(10) residue count
0
1
2
3
4
5
6
Position
0
20
40
60

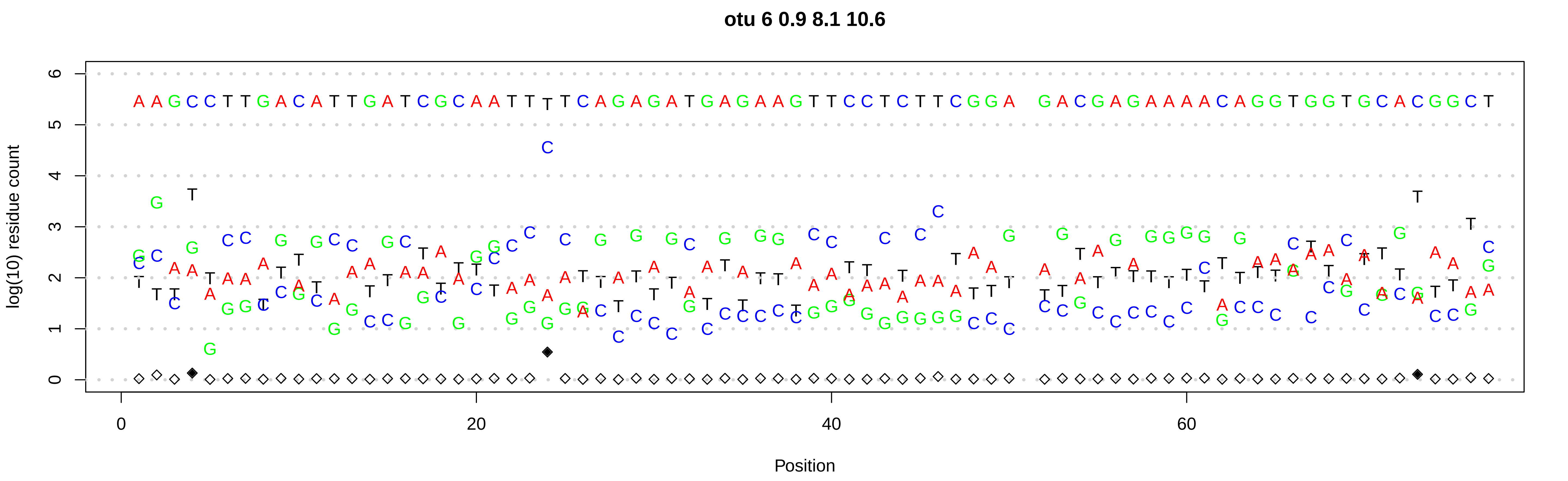
otu 6 0.9 8.1 10.6
log(10) residue count
Position
0
1
2
3
4
5
6
0
20
40
60

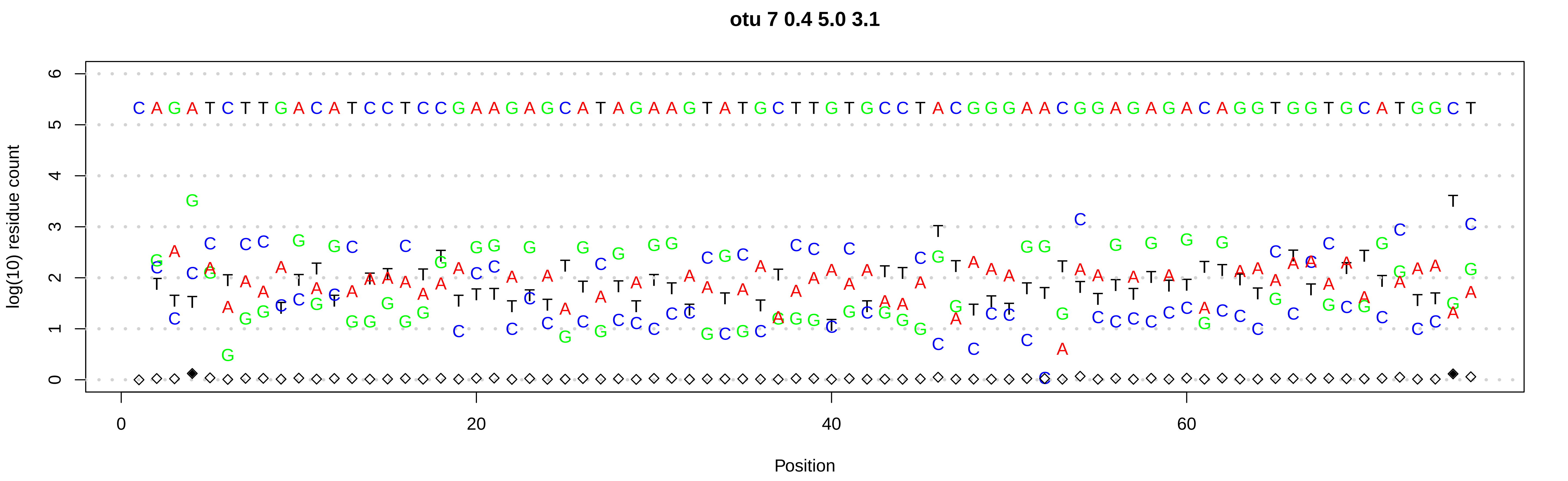
otu 7 0.4 5.0 3.1
log(10) residue count
0
1
2
3
4
5
6
Position
0
20
40
60

otu 8 0.6 5.4 2.8

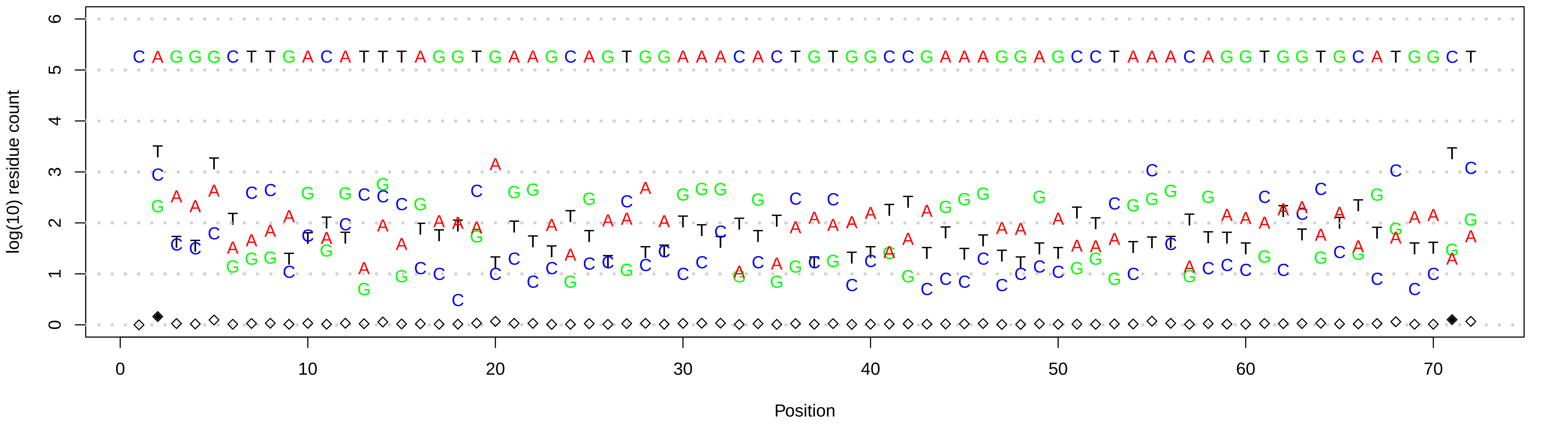

log(10) residue count
0
1
2
3
4
5
6
Position
0
10
20
30
40
50
60
70
C A G G G C T T G A C A T T T A G G T G A A G C A G T G G A A A C A C T G T G G C C G A A A G G A G C C T A A A C A G G T G G T G C A T G G C T

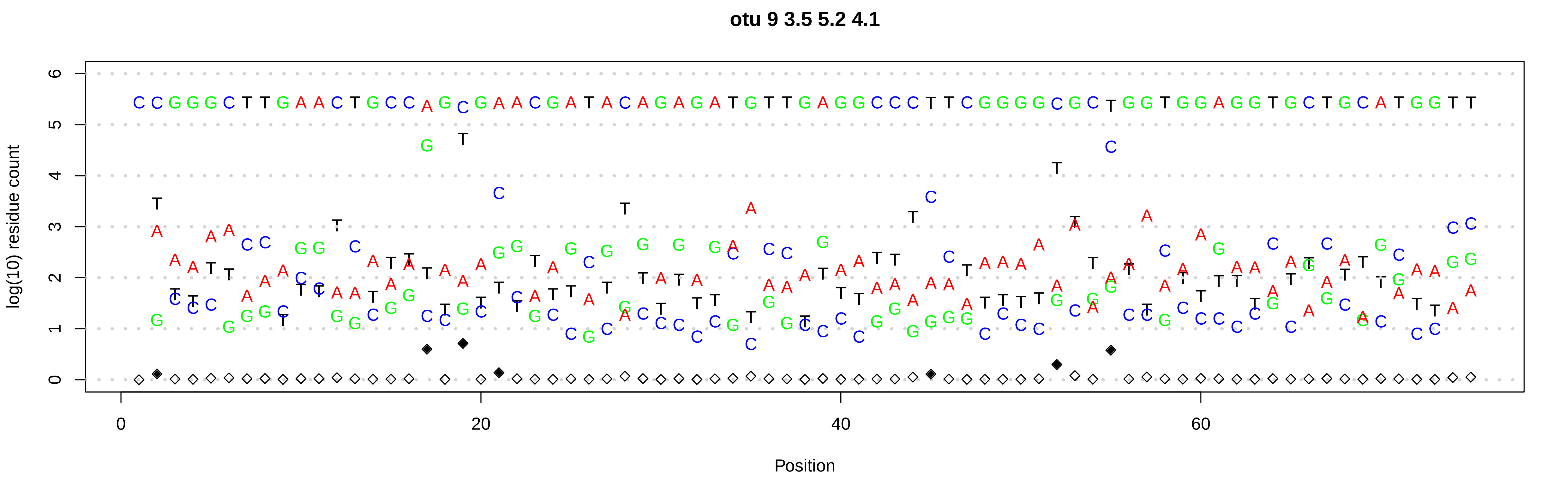
otu 9 3.5 5.2 4.1
log(10) residue count
0
1
2
3
4
5
6
Position
0
20
40
60

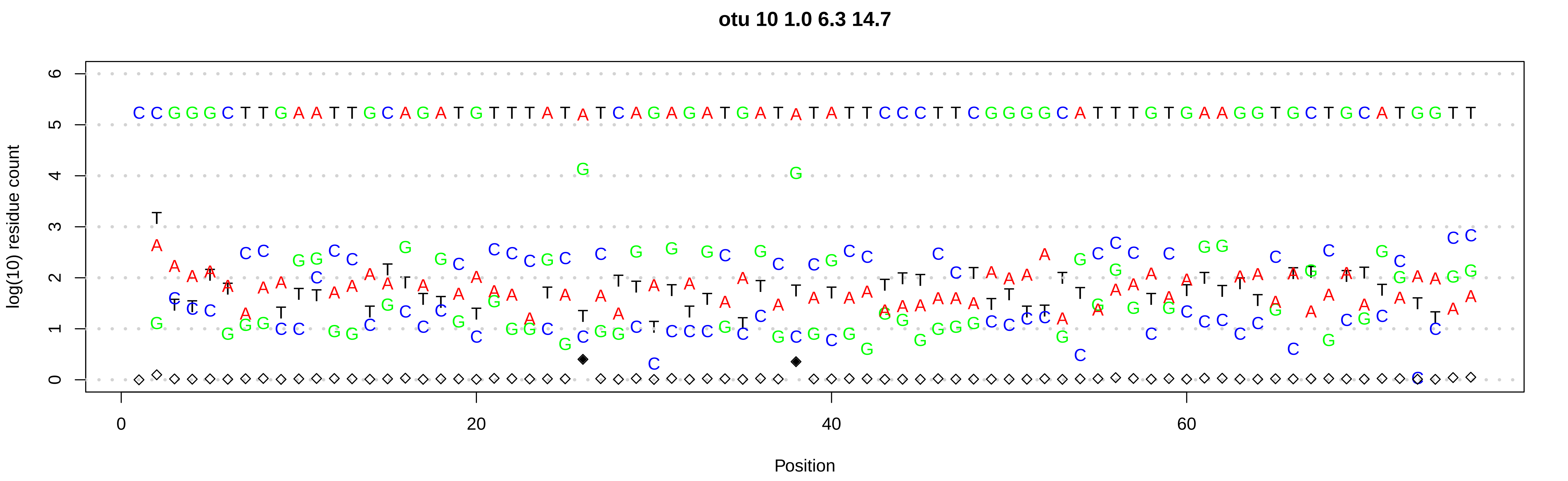

otu 10 1.0 6.3 14.7
log(10) residue count
Position

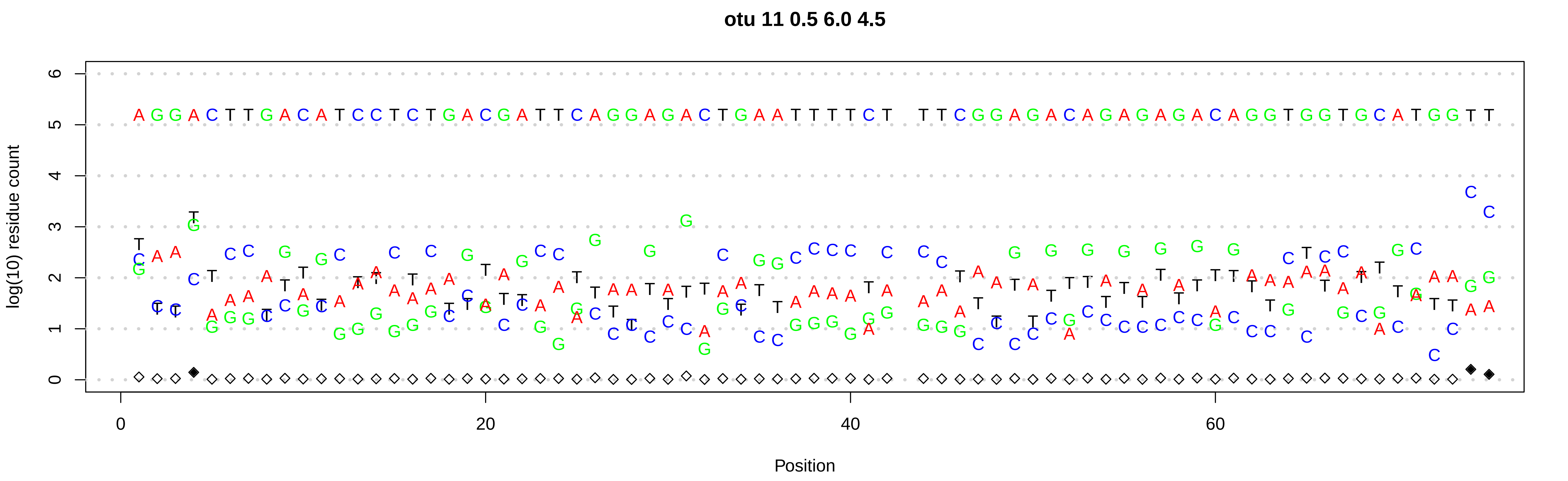

otu 11 0.5 6.0 4.5
log(10) residue count
Position

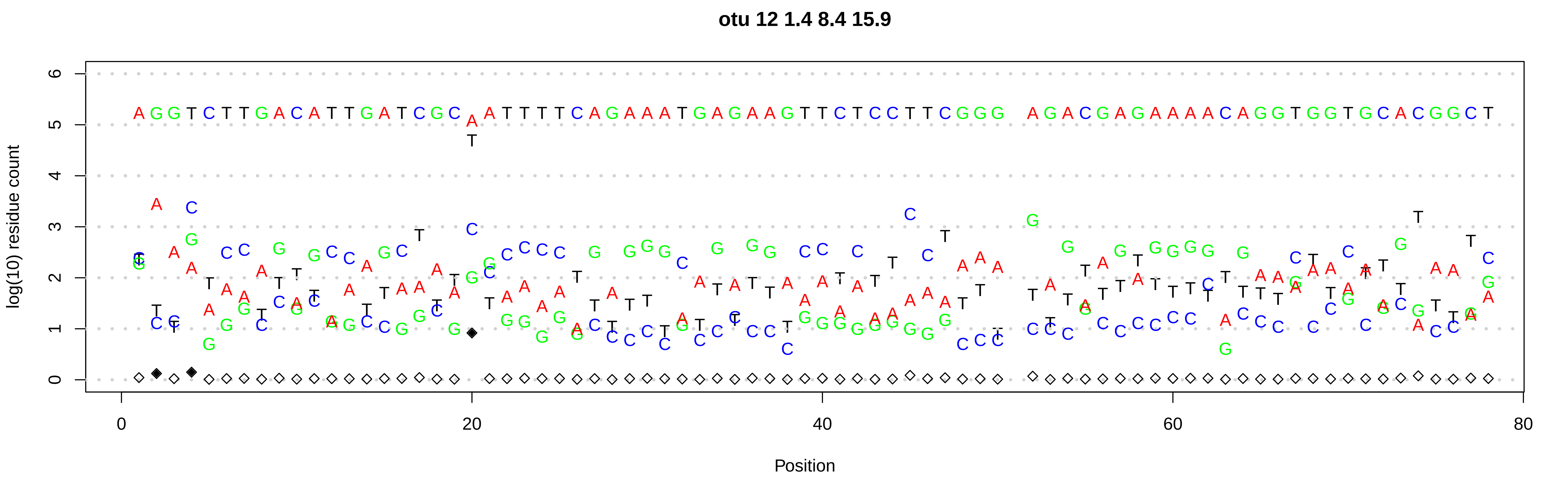
otu 12 1.4 8.4 15.9
log(10) residue count
Position
0
1
2
3
4
5
6
0
20
40
60
80

otu 13 1.9 6.1 2.6

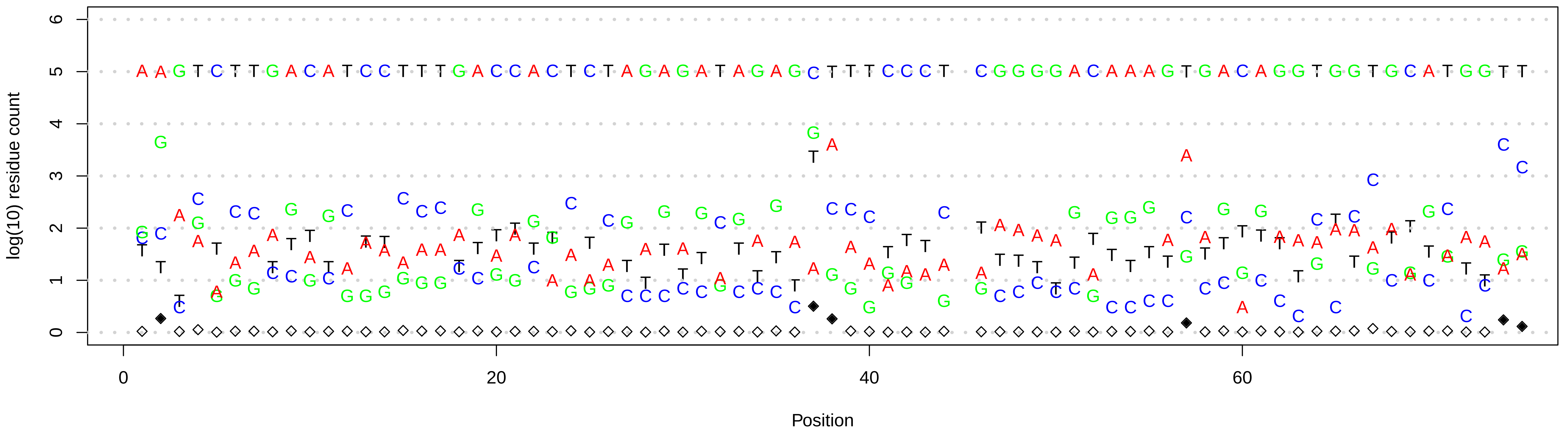

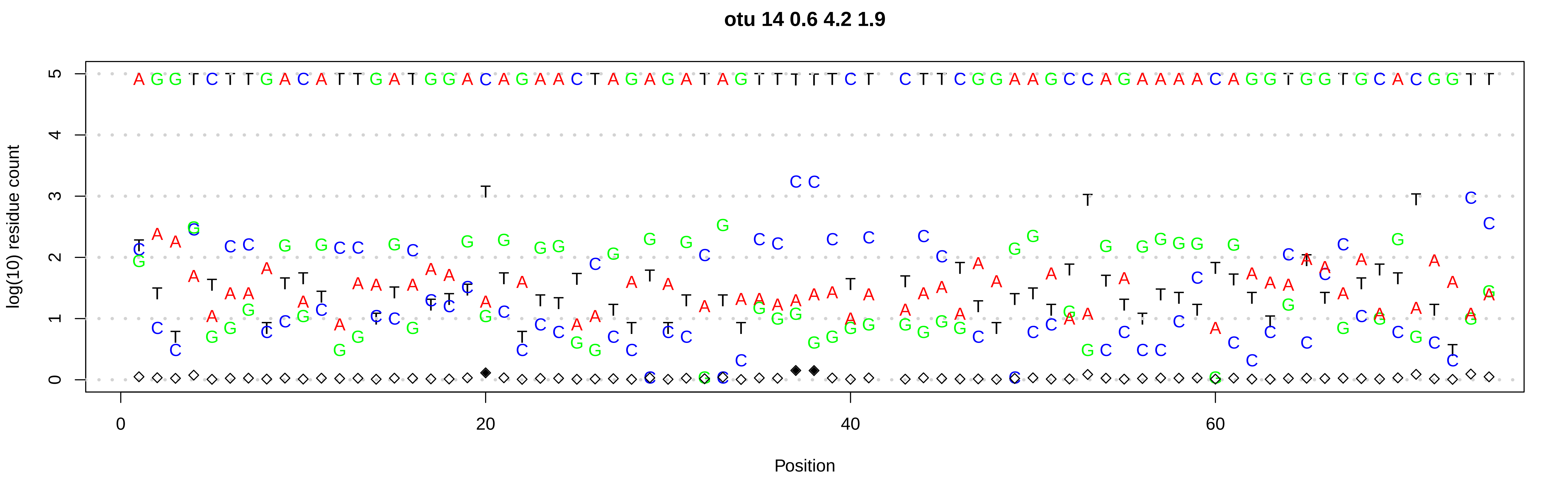

otu 14 0.6 4.2 1.9
log(10) residue count
Position

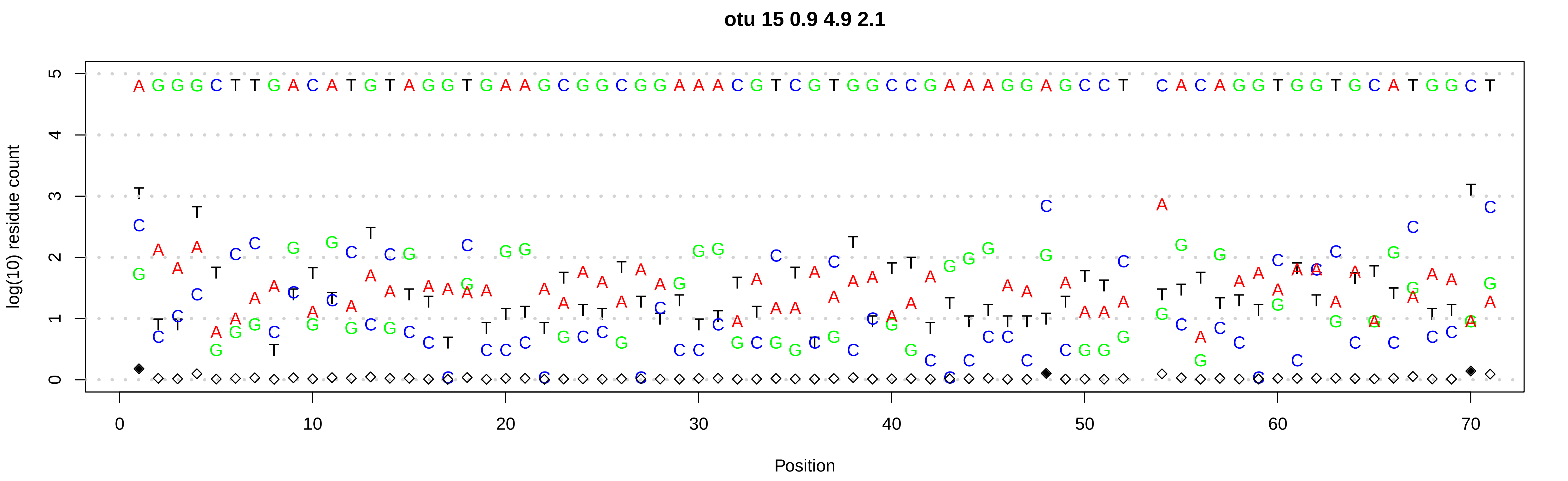
otu 15 0.9 4.9 2.1
A G G G C T T G A C A T G T A G G T G A A G C G G C G G A A A C G T C G T G G C C G A A A G G A G C C T C A C A G G T G G T G C A T G G C T
log(10) residue count
Position

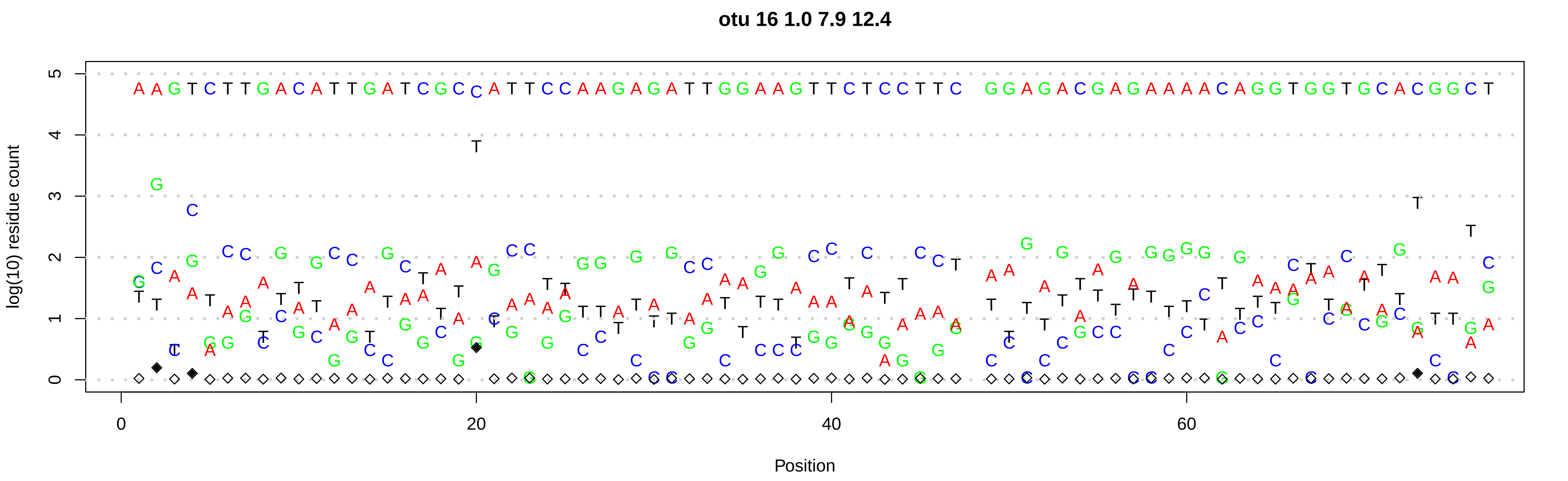
otu 16 1.0 7.9 12.4
log(10) residue count
0
1
2
3
4
5
Position
0
20
40
60

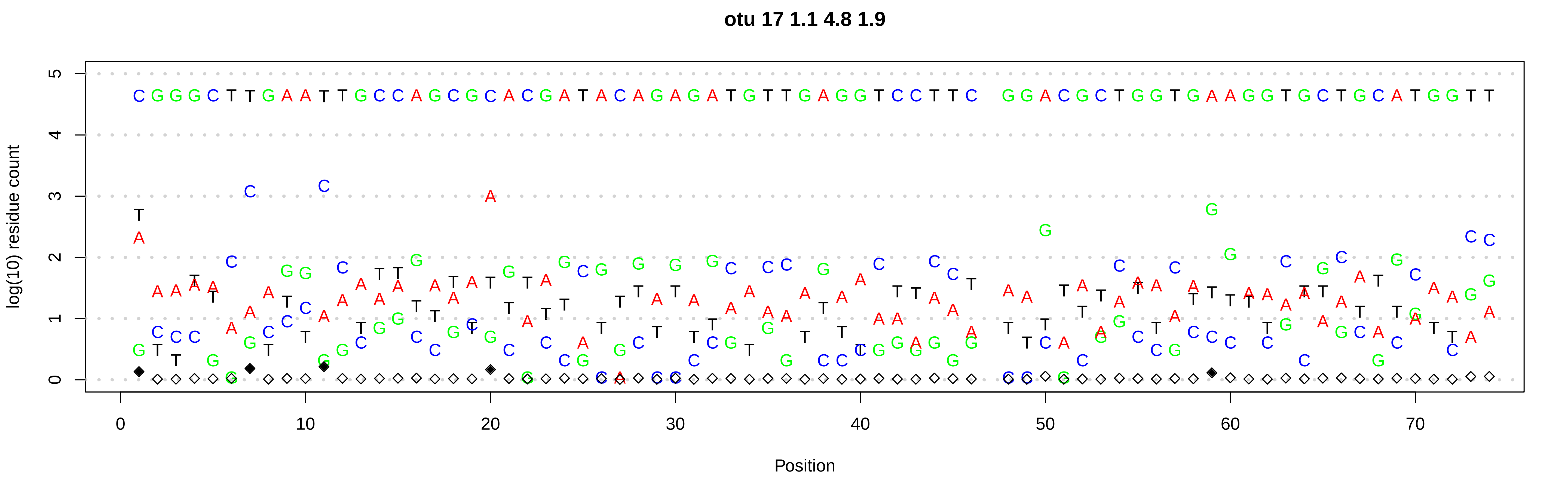
otu 17 1.1 4.8 1.9
log(10) residue count
Position
0
10
20
30
40
50
60
70
0
1
2
3
4
5

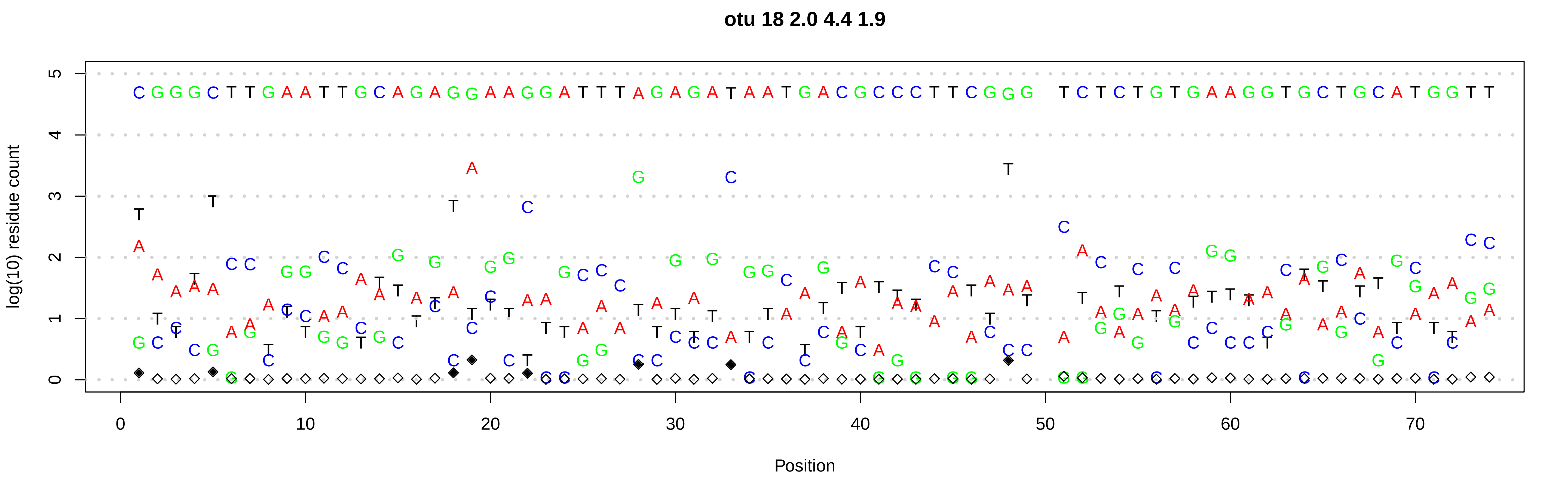
otu 18 2.0 4.4 1.9
CGGGCTTGAATTGCAGAGGAAGGATTTAGAGATAATGACGCCCTTCGGG TCTCTGTGAAGGTGCTGCATGGTT
log(10) residue count
0
1
2
3
4
5
Position
0
10
20
30
40
50
60
70

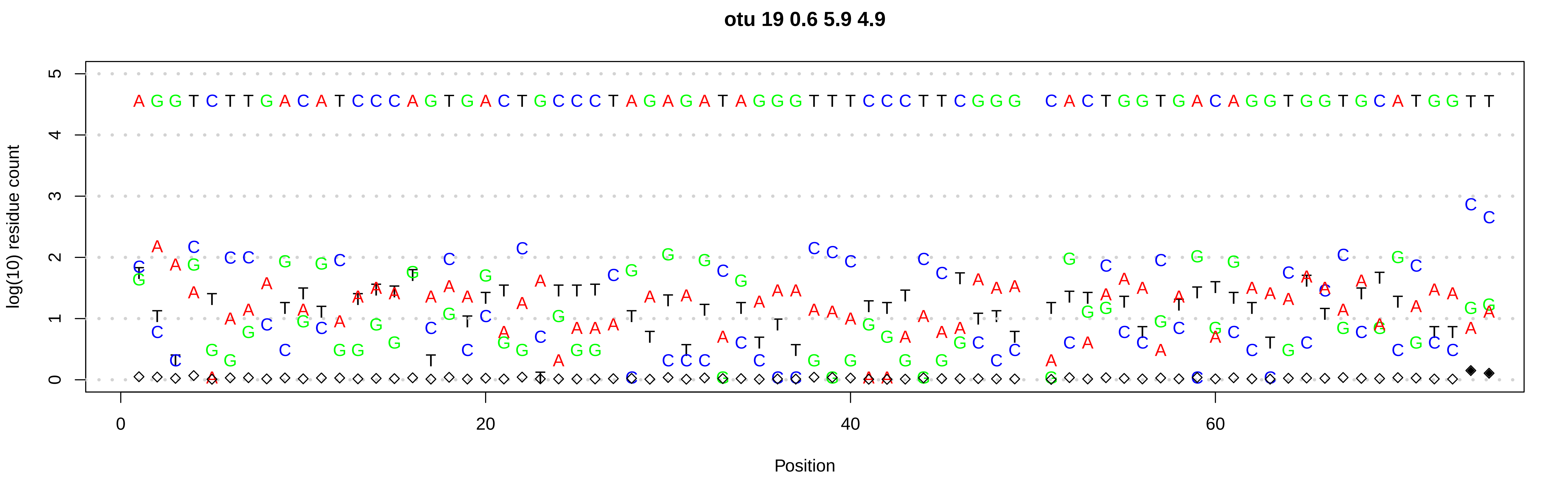
otu 19 0.6 5.9 4.9
AGGTCTTGACATCCCAGTGACTGCCCTAGAGATAGGGTTTCCCTTCGGG CACTGGTGACAGGTGGTGCATGGTT
log(10) residue count
0
1
2
3
4
5
Position
0
20
40
60

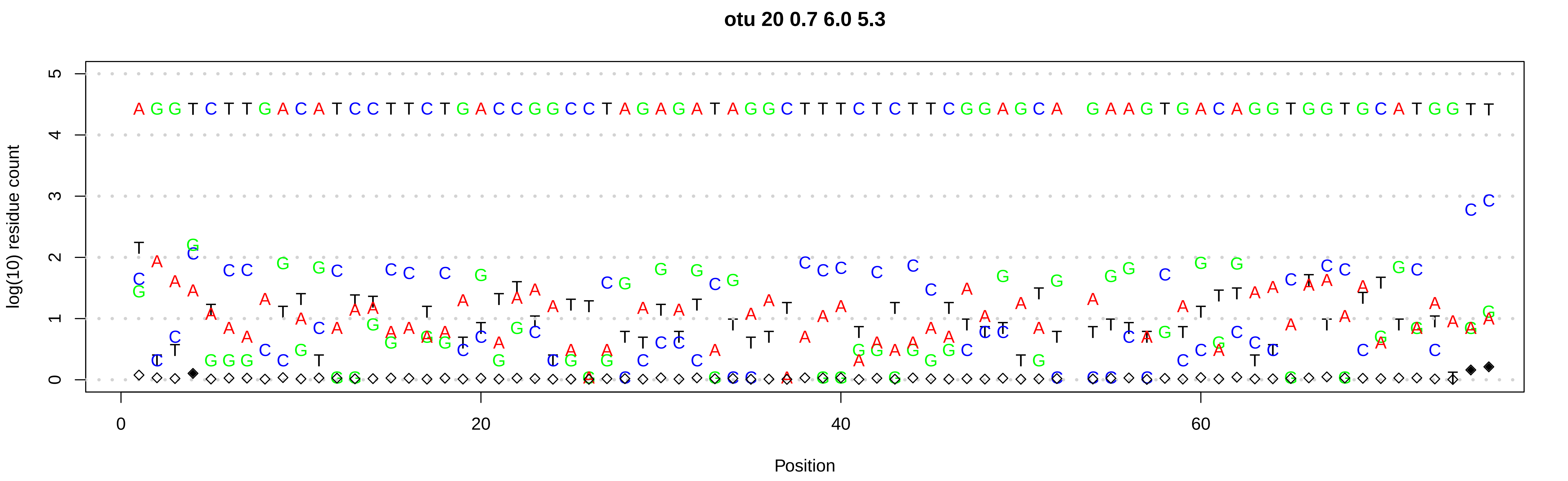
otu 20 0.7 6.0 5.3
log(10) residue count
Position
0
1
2
3
4
5
0
20
40
60

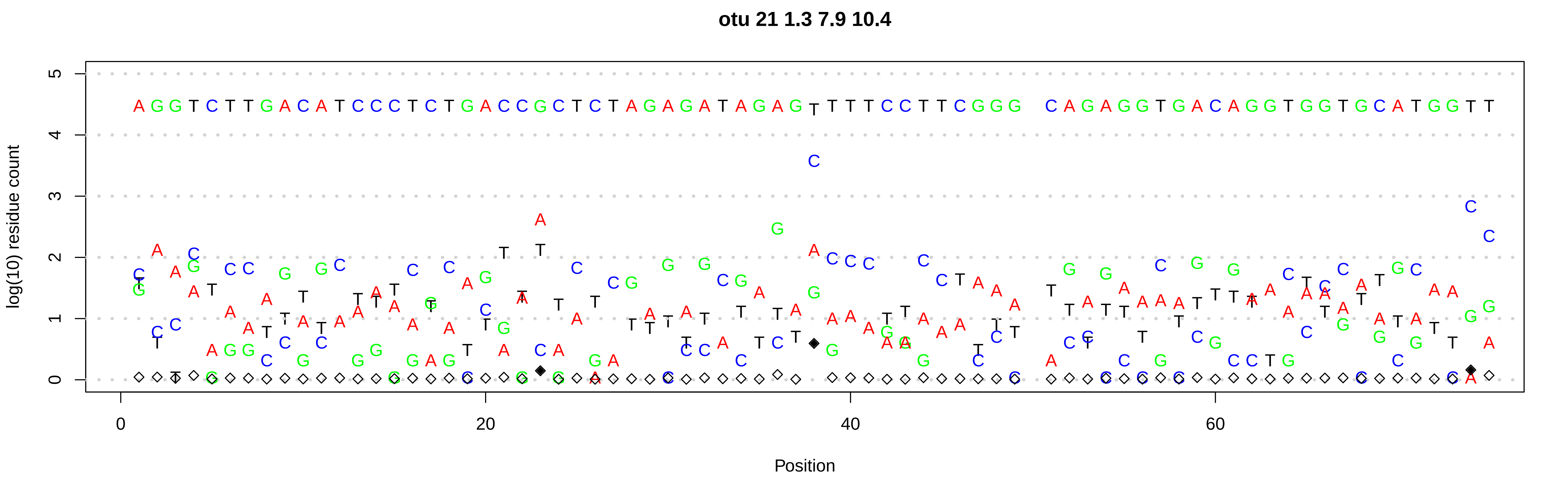

otu 21 1.3 7.9 10.4
AGGTCTTGACATCCCTCTGACCGCTCTAGAGATAGAGTTTTCCTTCGGG CAGAGGTGACAGGTGGTGCATGGTT
log(10) residue count
0
1
2
3
4
5
0
20
40
60
Position

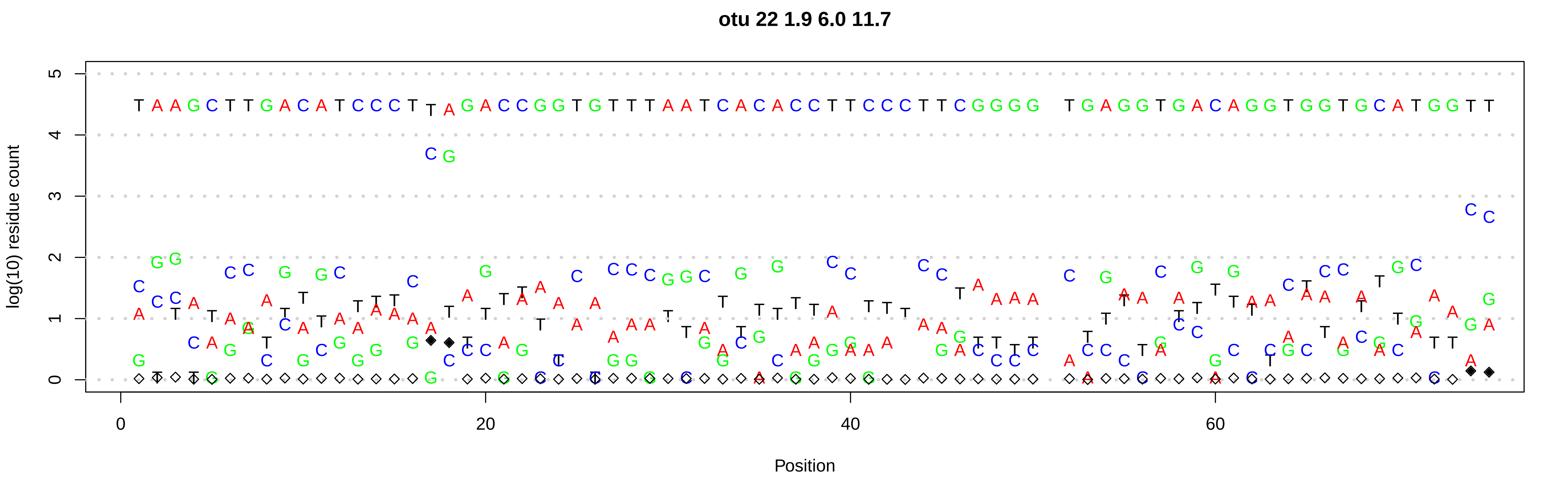
otu 22 1.9 6.0 11.7
log(10) residue count
Position
0
20
40
60
0
1
2
3
4
5

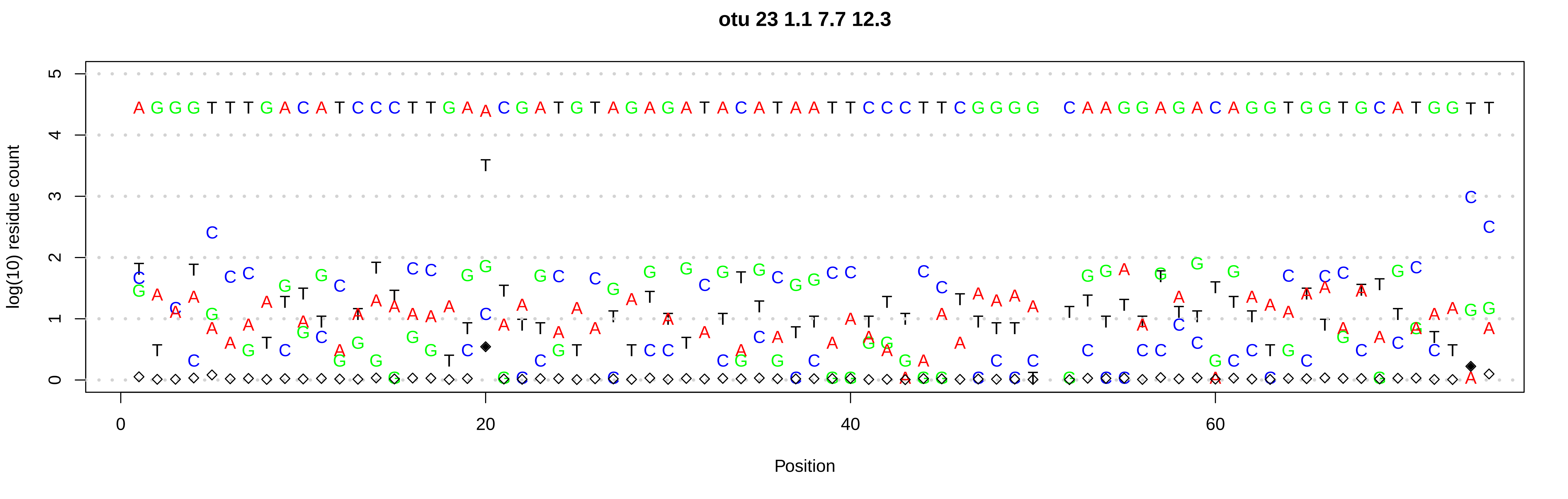
otu 23 1.1 7.7 12.3
log(10) residue count
0
1
2
3
4
5
0
20
40
60
Position

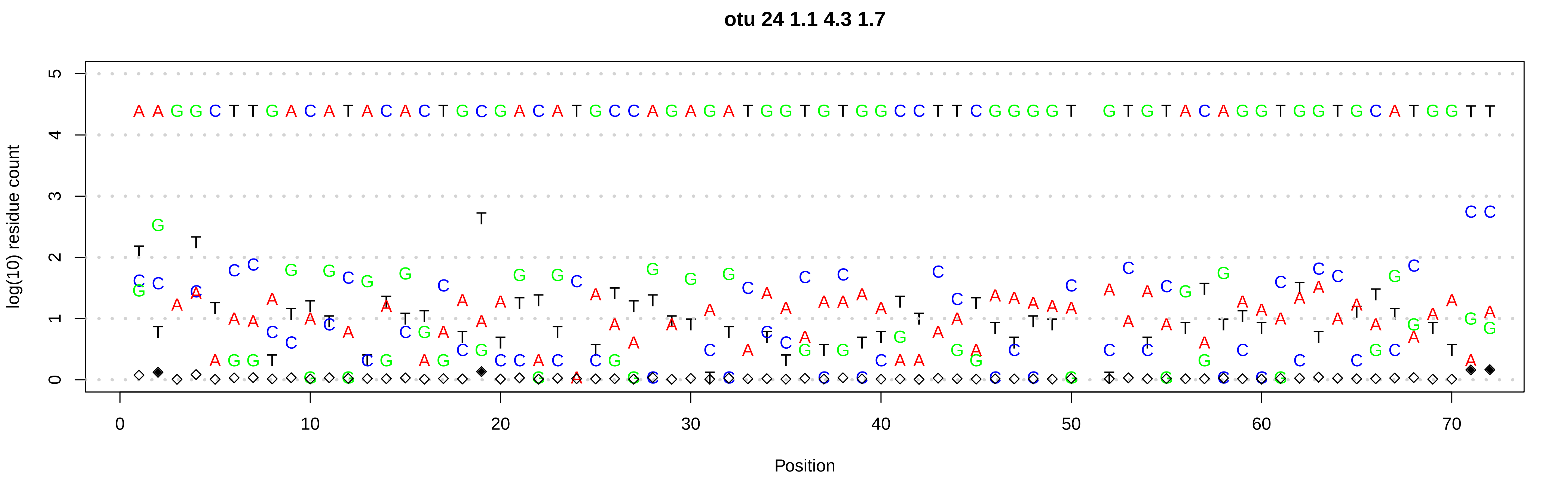
otu 24 1.1 4.3 1.7
log(10) residue count
Position
0
1
2
3
4
5
10
20
30
40
50
60
70

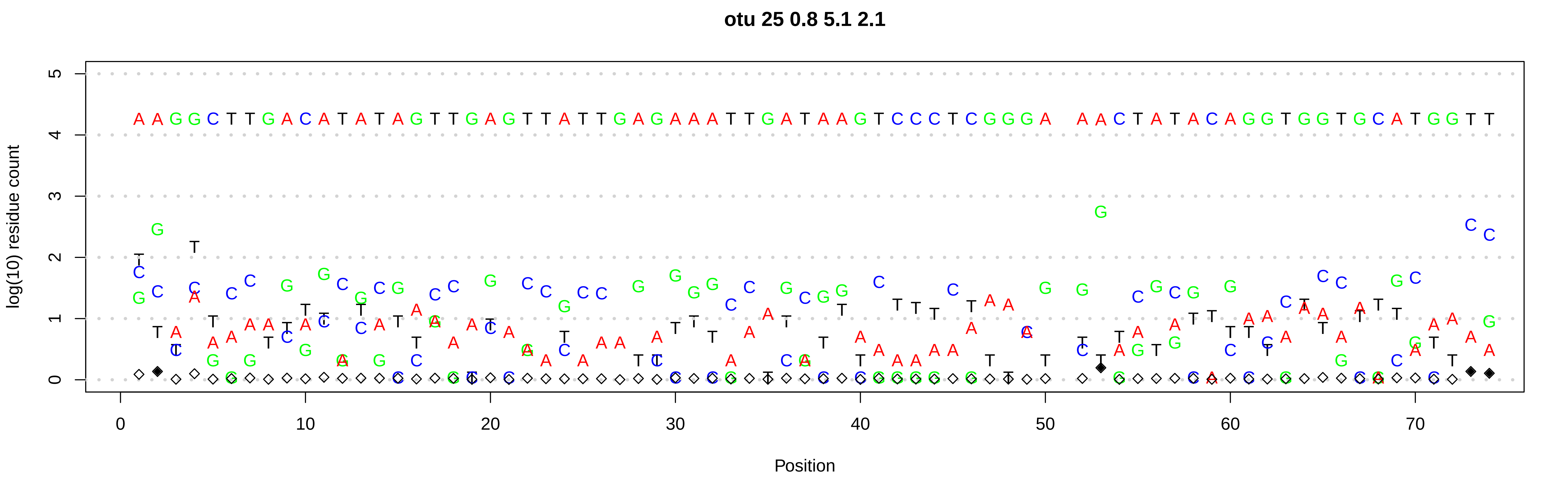

otu 25 0.8 5.1 2.1
log(10) residue count
0
1
2
3
4
5
Position
0
10
20
30
40
50
60
70
AAGGCTTGACATATAGTTGAGTTATTGAGAAATTGATAAGTCCCTCGGGA AACTATACAGGTGGTGCATGGTT

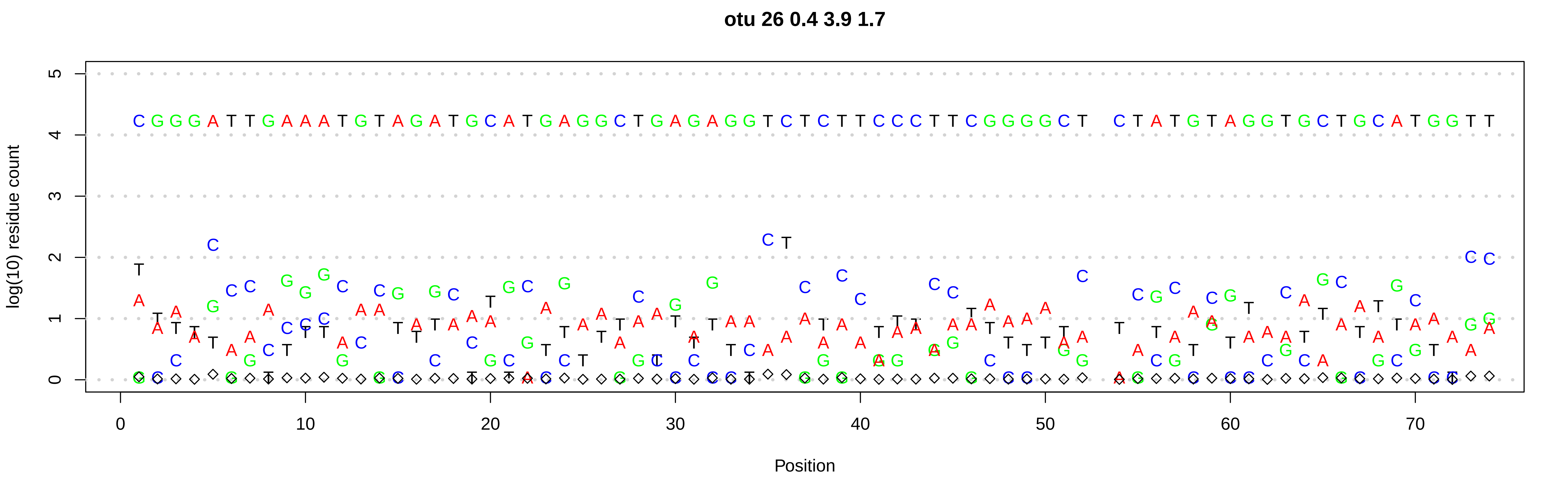
otu 26 0.4 3.9 1.7
log(10) residue count
Position
0
1
2
3
4
5
0
10
20
30
40
50
60
70

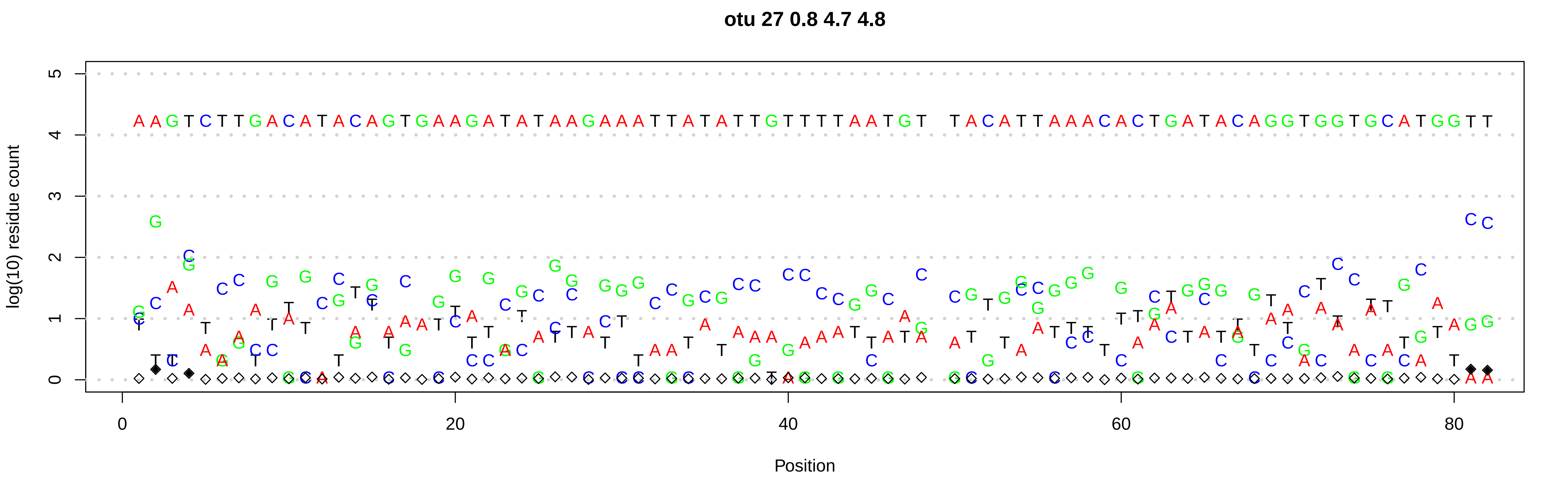

otu 27 0.8 4.7 4.8
log(10) residue count
Position

otu 28 1.1 5.8 4.0

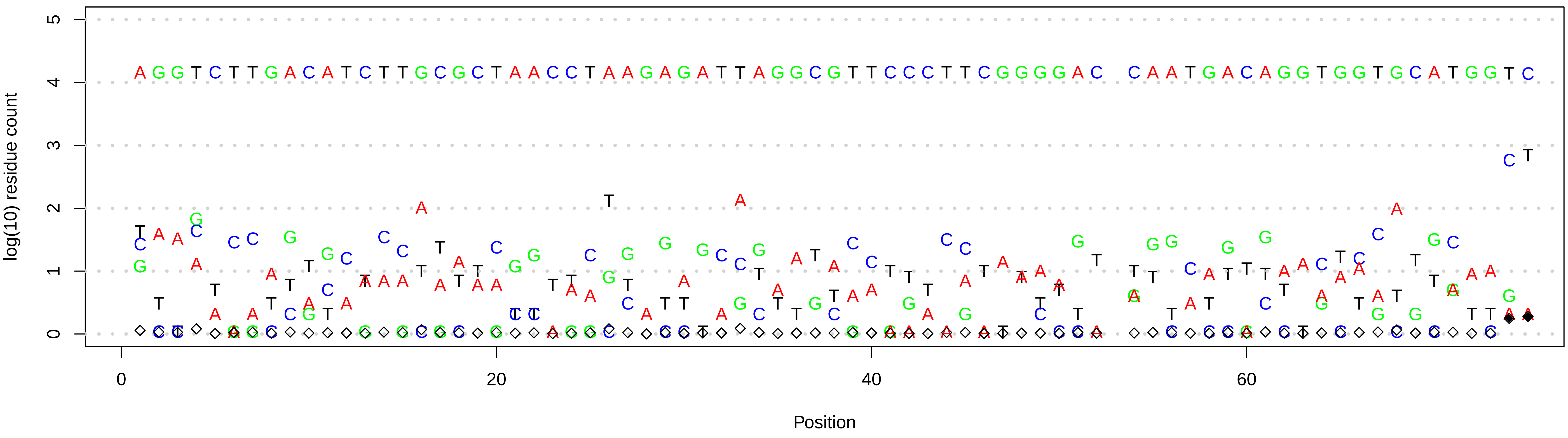

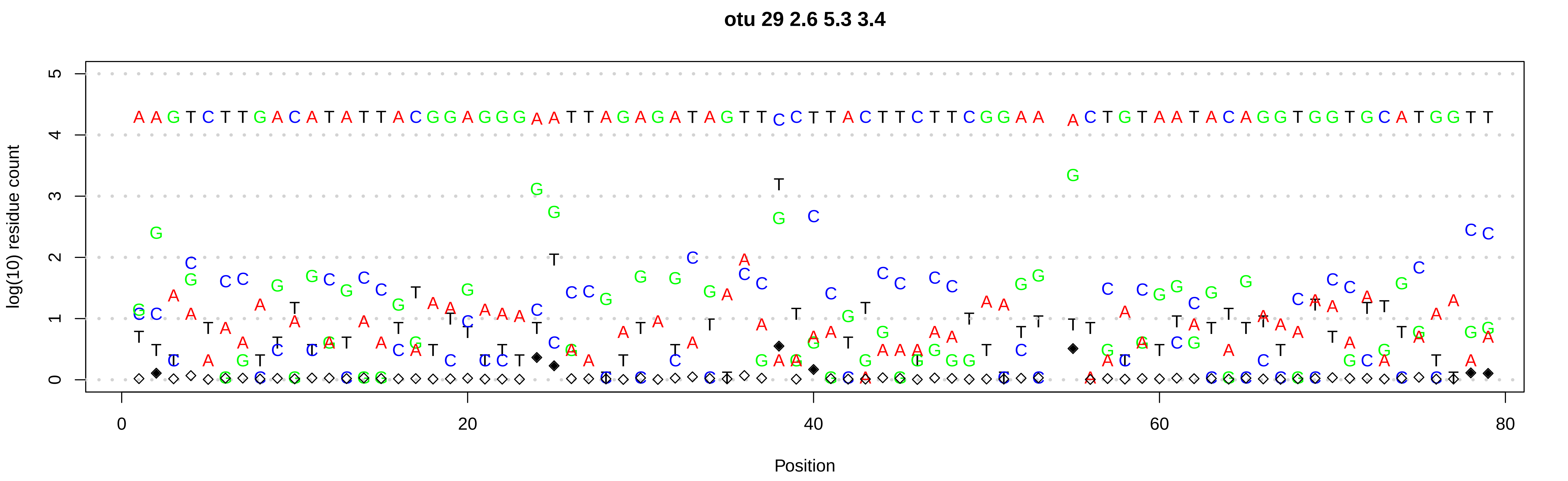
otu 29 2.6 5.3 3.4
log(10) residue count
Position
0
20
40
60
80
0
1
2
3
4
5

otu 30 0.4 5.0 3.9

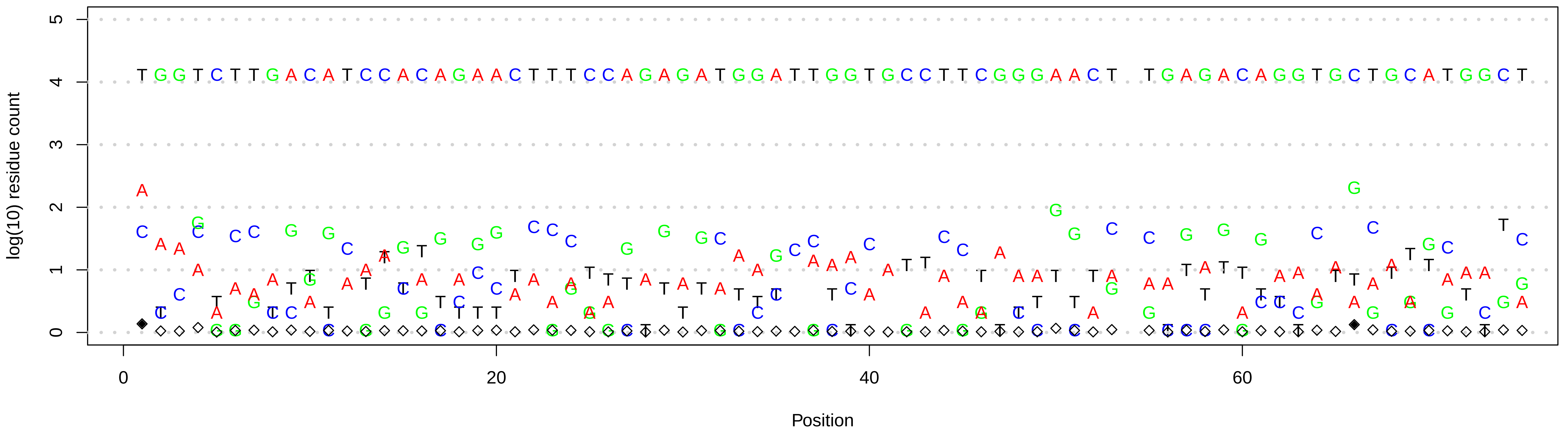

otu 31 1.2 7.9 9.7

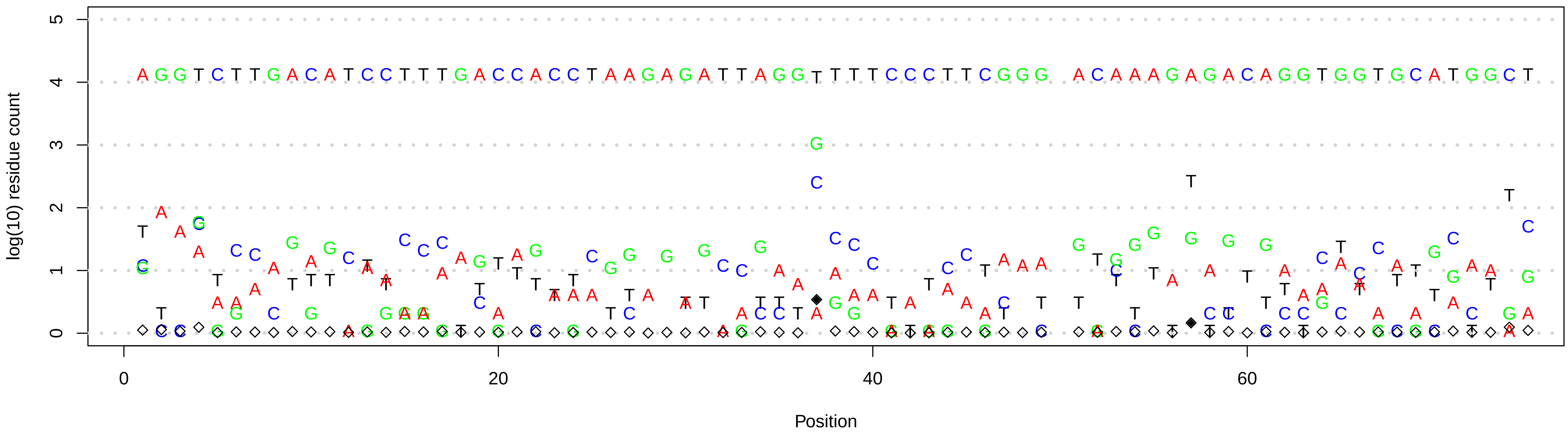

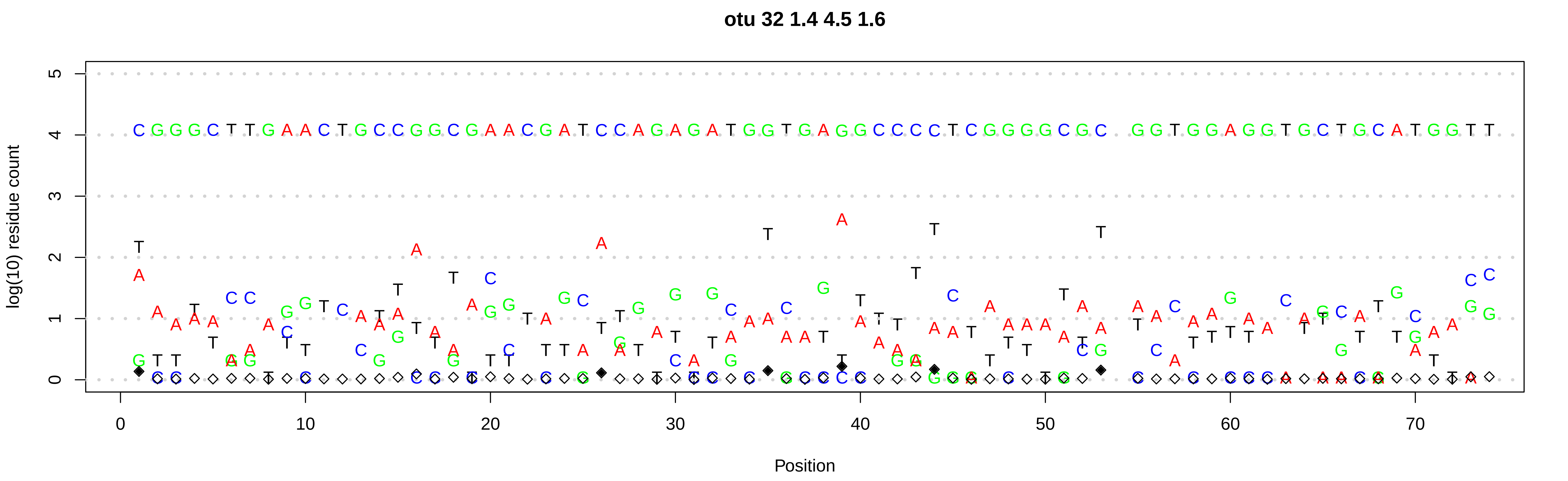
otu 32 1.4 4.5 1.6
log(10) residue count
Position
0
10
20
30
40
50
60
70
1
2
3
4
5

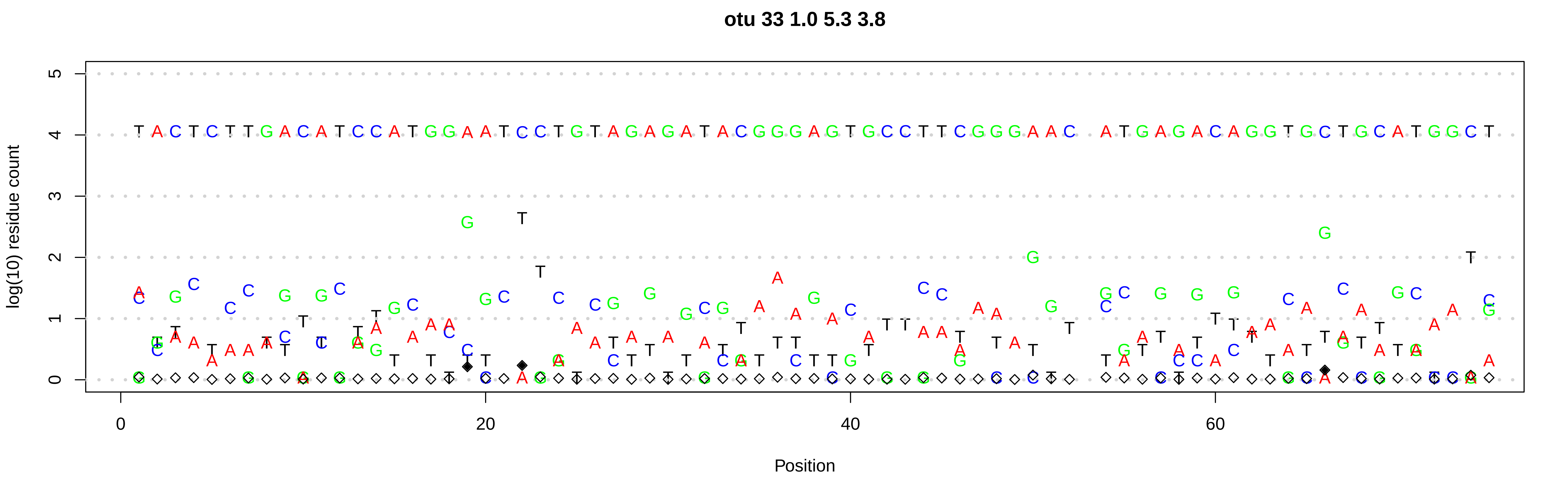
otu 33 1.0 5.3 3.8
log(10) residue count
0
1
2
3
4
5
Position
0
20
40
60

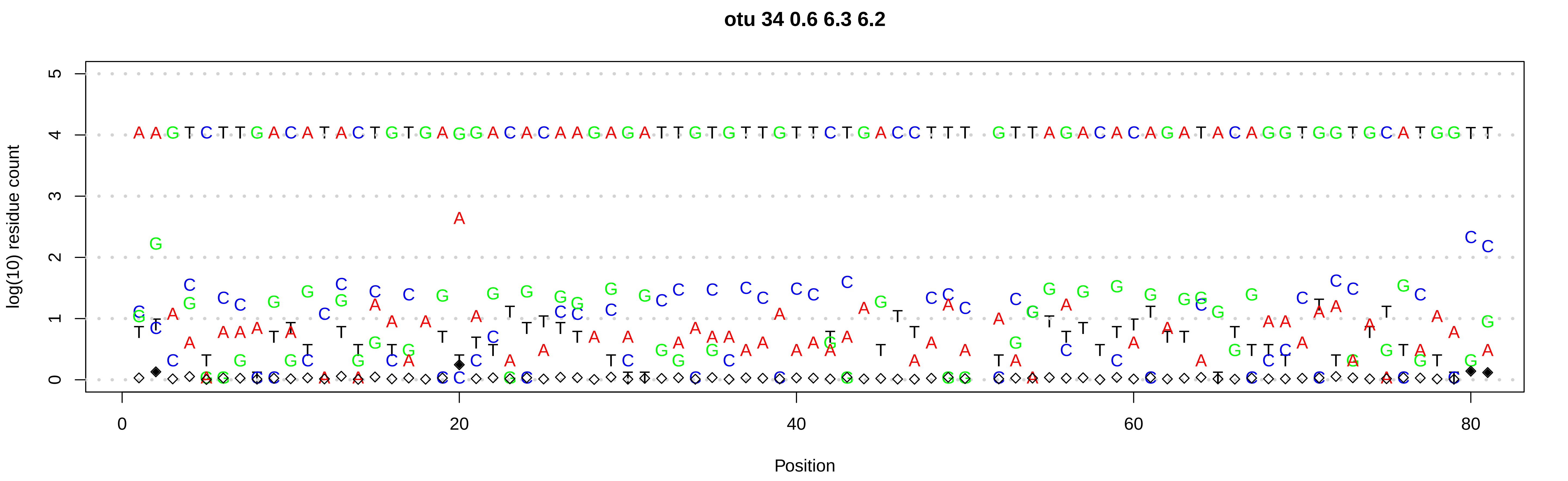
otu 34 0.6 6.3 6.2
log(10) residue count
Position
0
20
40
60
80
0
1
2
3
4
5

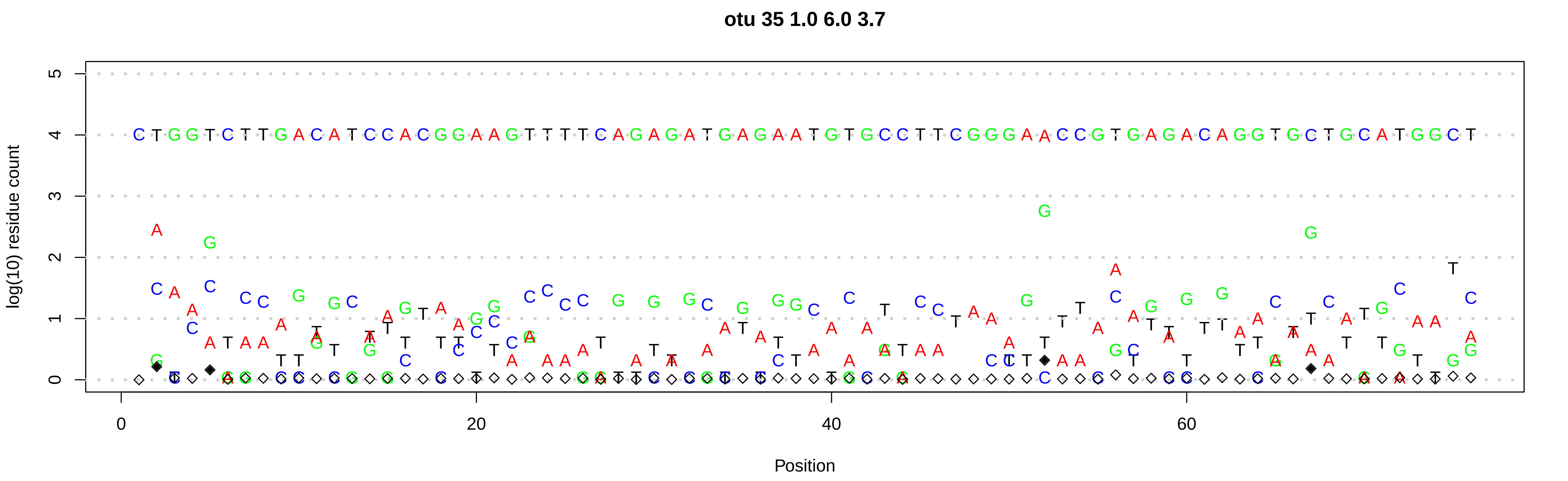
otu 35 1.0 6.0 3.7
log(10) residue count
Position
0
20
40
60
1
2
3
4
5

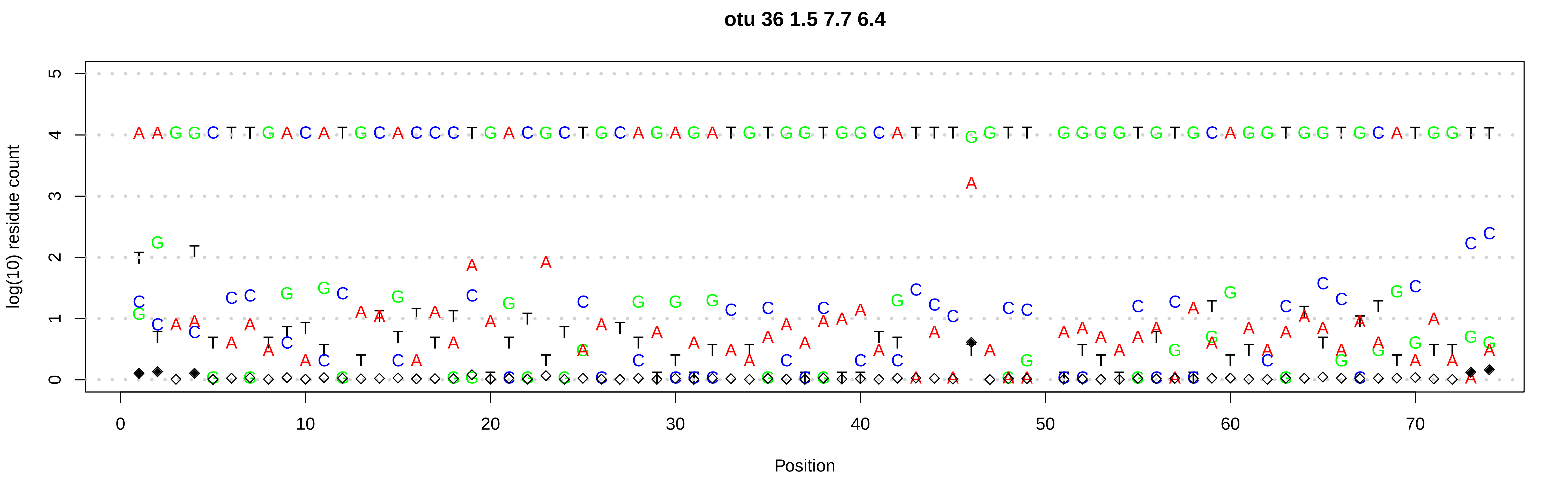
otu 36 1.5 7.7 6.4
log(10) residue count
Position
0
1
2
3
4
5
10
20
30
40
50
60
70

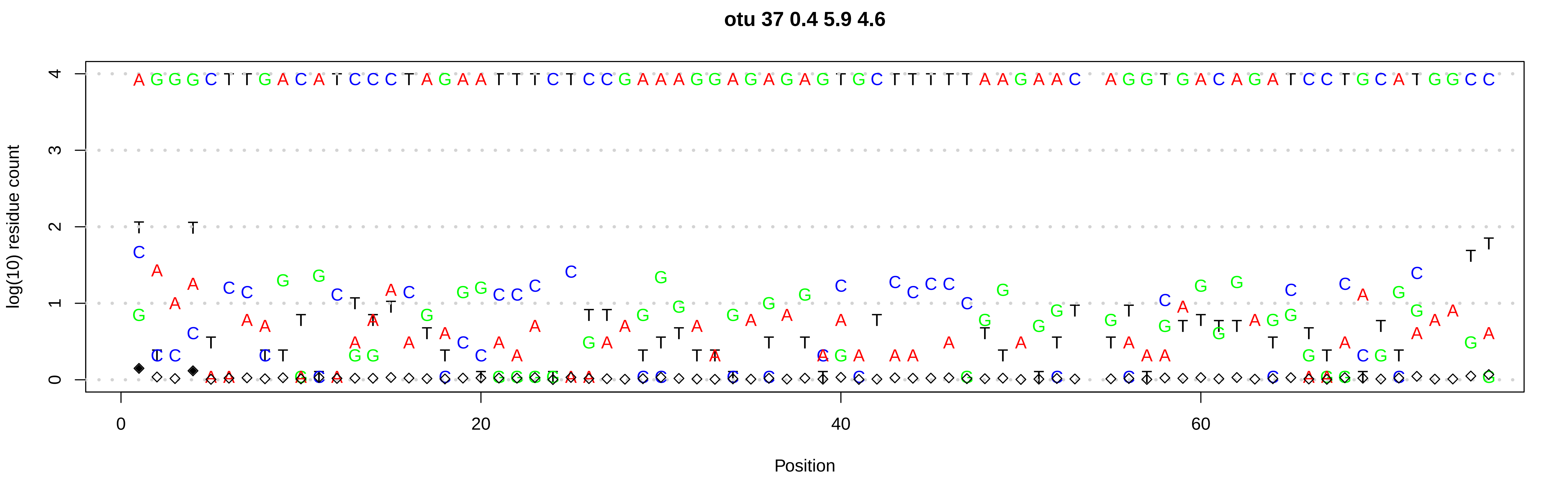

otu 37 0.4 5.9 4.6
log(10) residue count
0
1
2
3
4
Position
0
20
40
60

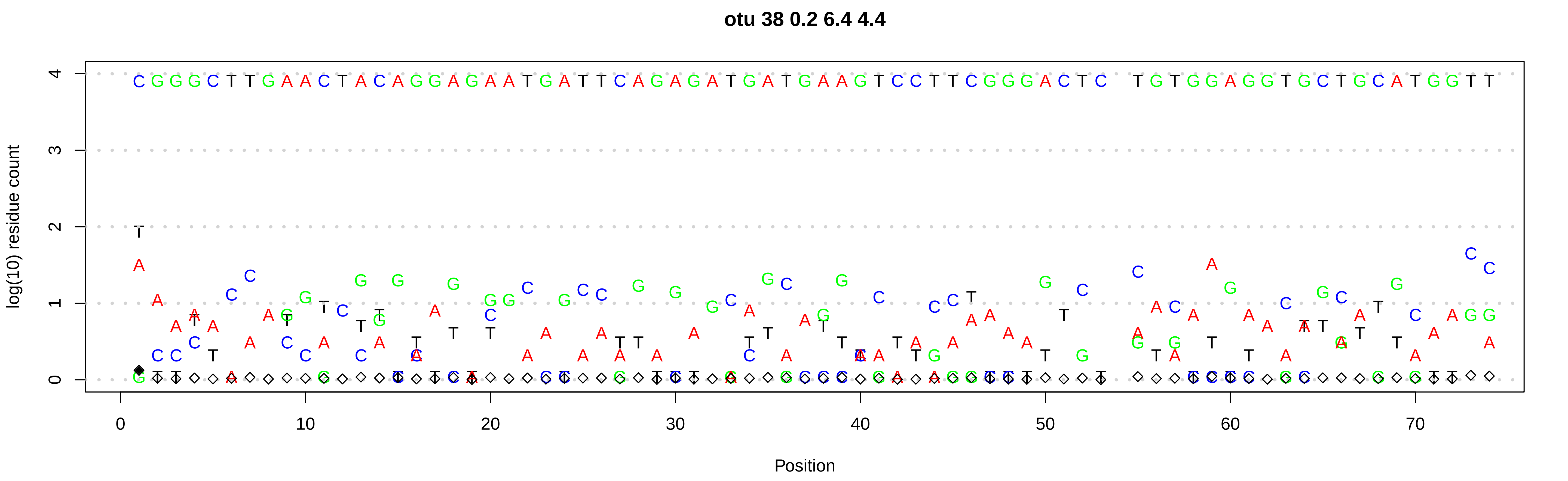
otu 38 0.2 6.4 4.4
log(10) residue count
Position
0
1
2
3
4
0
10
20
30
40
50
60
70

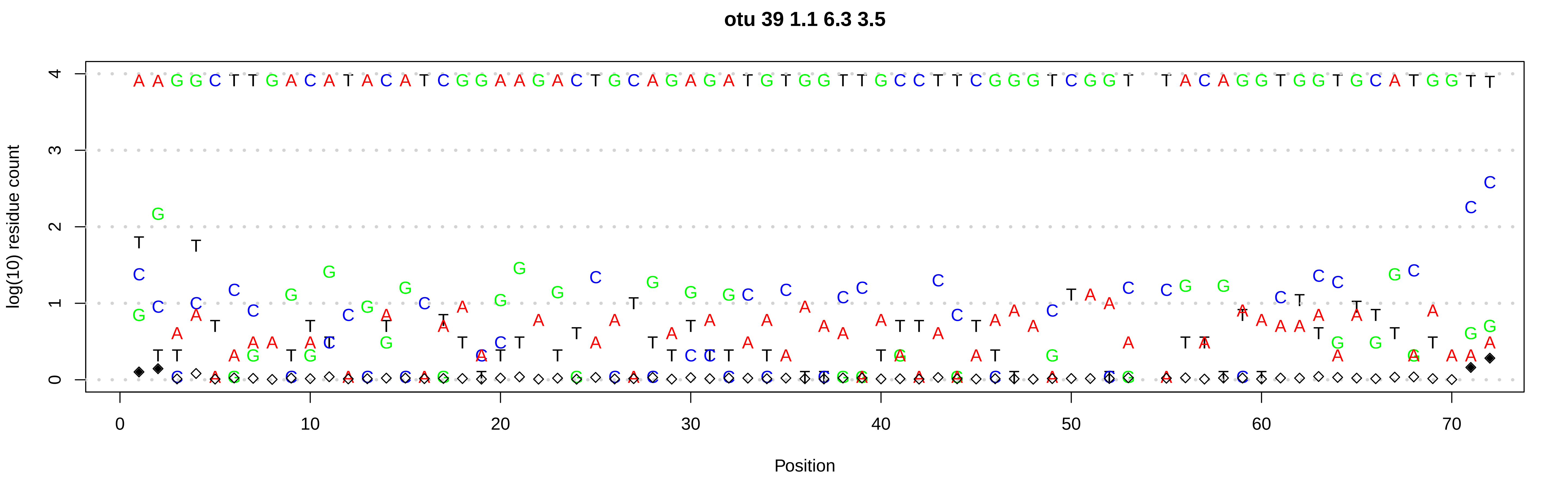
otu 39 1.1 6.3 3.5
log(10) residue count
Position
0
1
2
3
4
0
10
20
30
40
50
60
70

otu 41 1.4 8.6 23.1

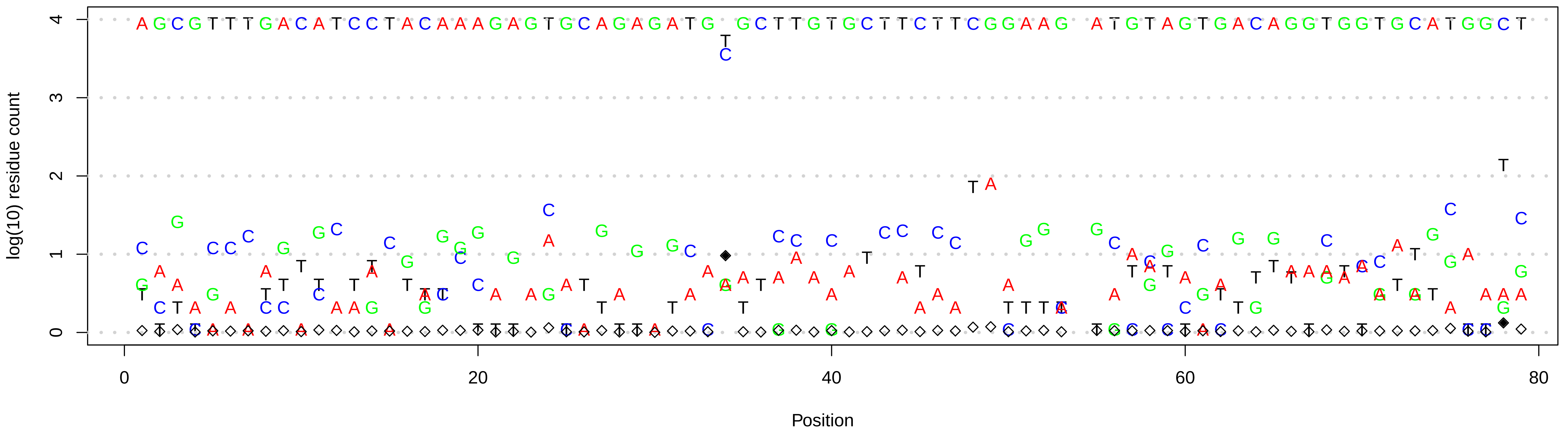

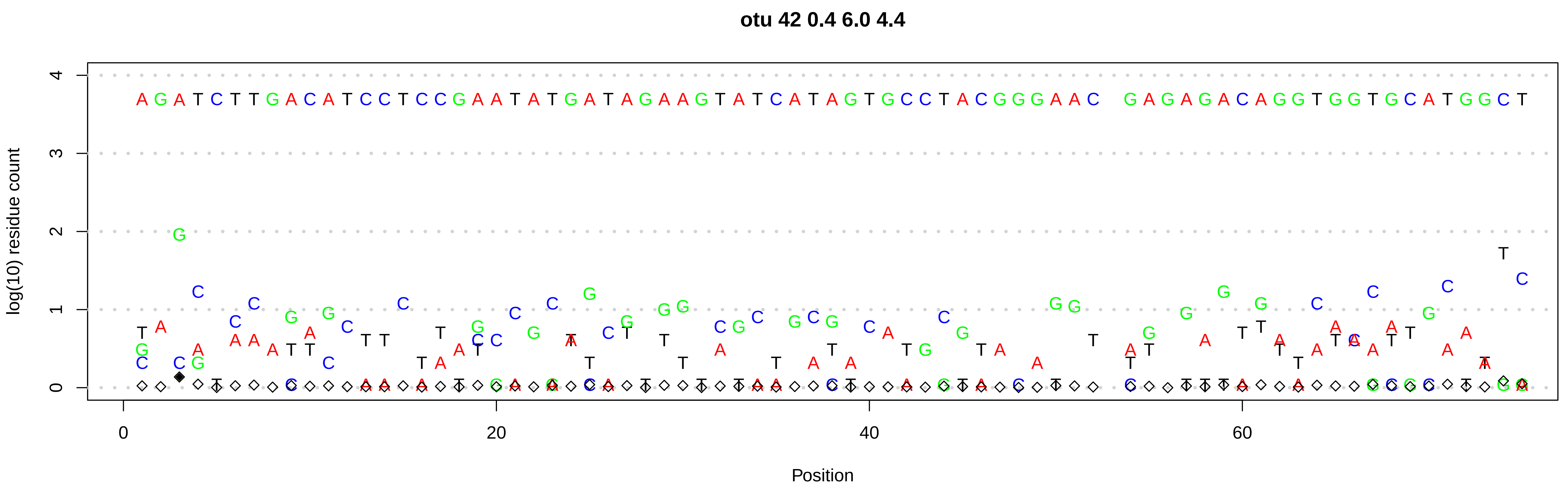
otu 42 0.4 6.0 4.4
A G A T C T T G A C A T C C T C C G A A T A T G A T A G A A G T A T C A T A G T G C C T A C G G G A A C G A G A G A C A G G T G G T G C A T G G C T
log(10) residue count
Position
0
1
2
3
4
0
20
40
60

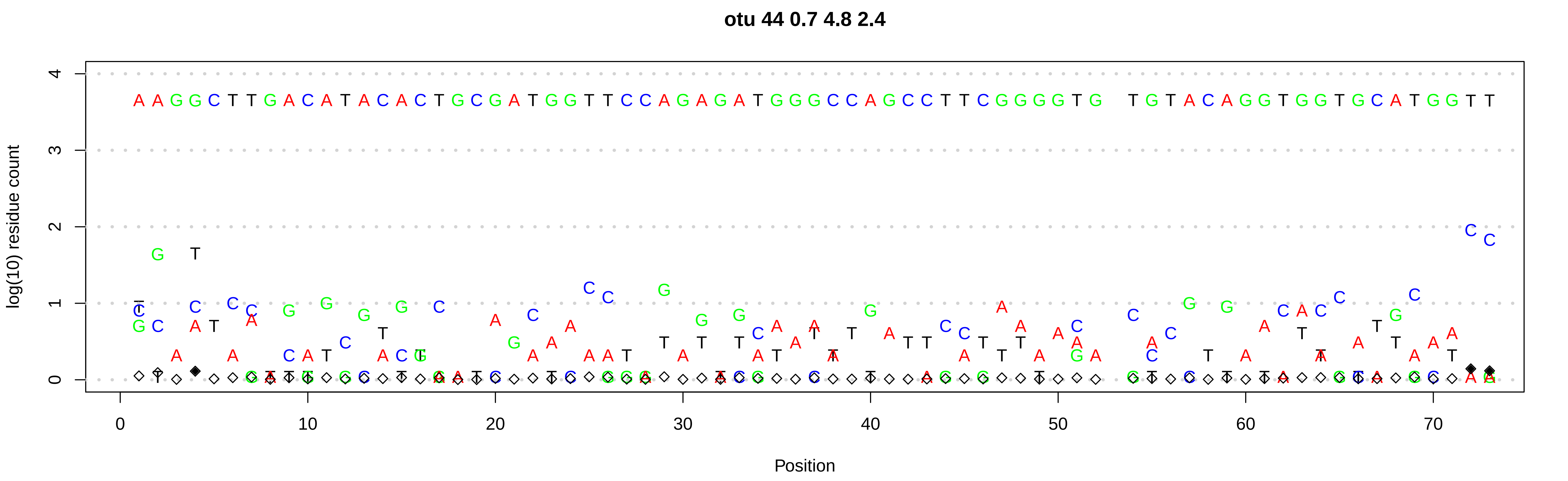

otu 44 0.7 4.8 2.4
log(10) residue count
Position
0
1
2
3
4
0
10
20
30
40
50
60
70
A A G G C T T G A C A T A C A C T G C G A T G G T T C C A G A G A T G G G C C A G C C T T C G G G G T G T G T A C A G G T G G T G C A T G G T T

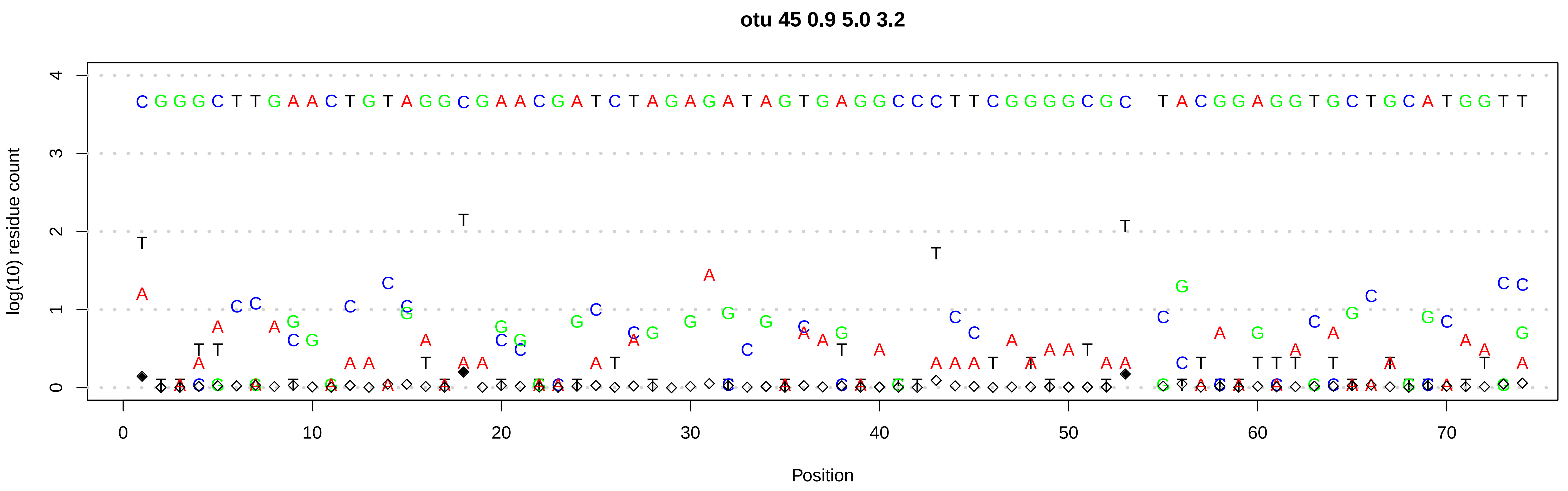
otu 45 0.9 5.0 3.2
log(10) residue count
0
1
2
3
4
Position
0
10
20
30
40
50
60
70

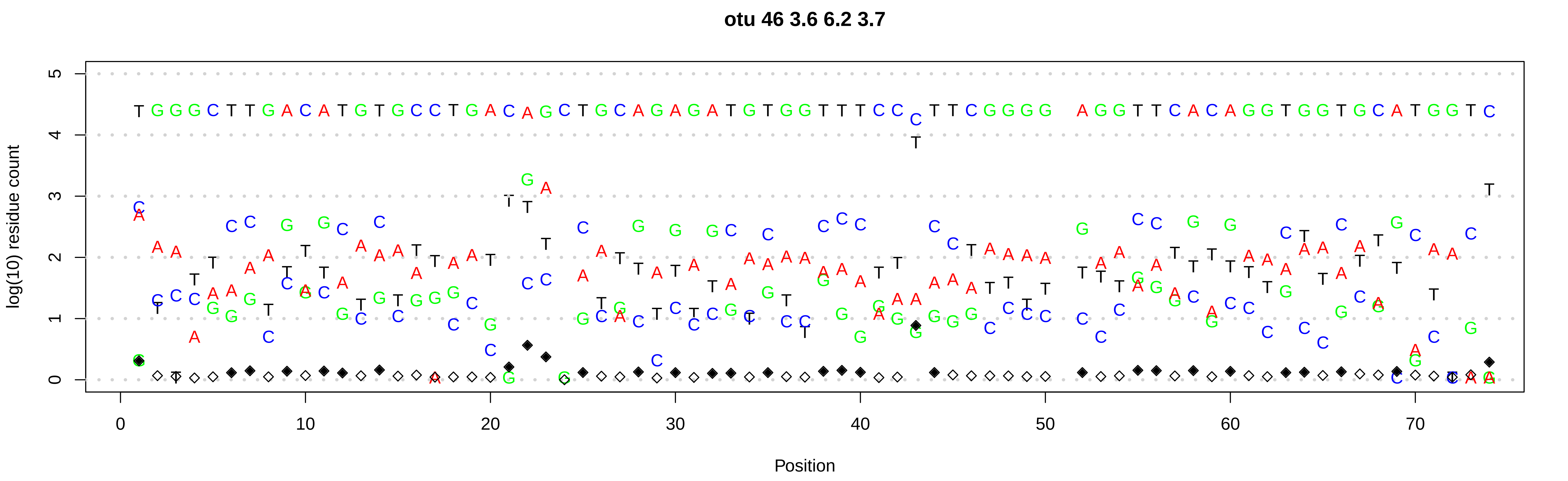
otu 46 3.6 6.2 3.7
log(10) residue count
Position
0
10
20
30
40
50
60
70
0
1
2
3
4
5

otu 47 4.4 6.6 29.1

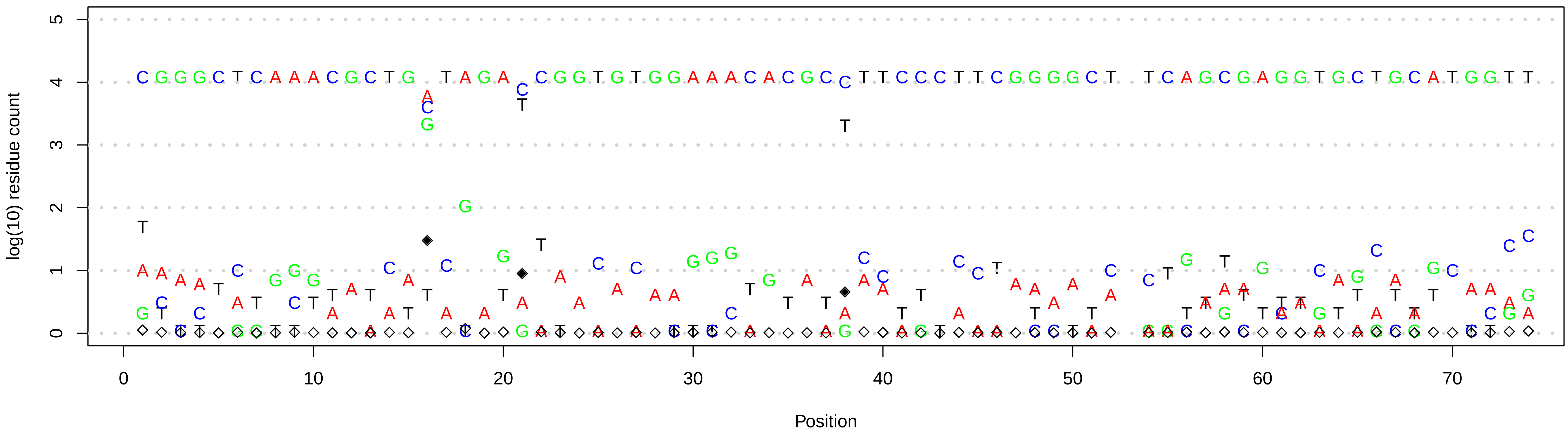

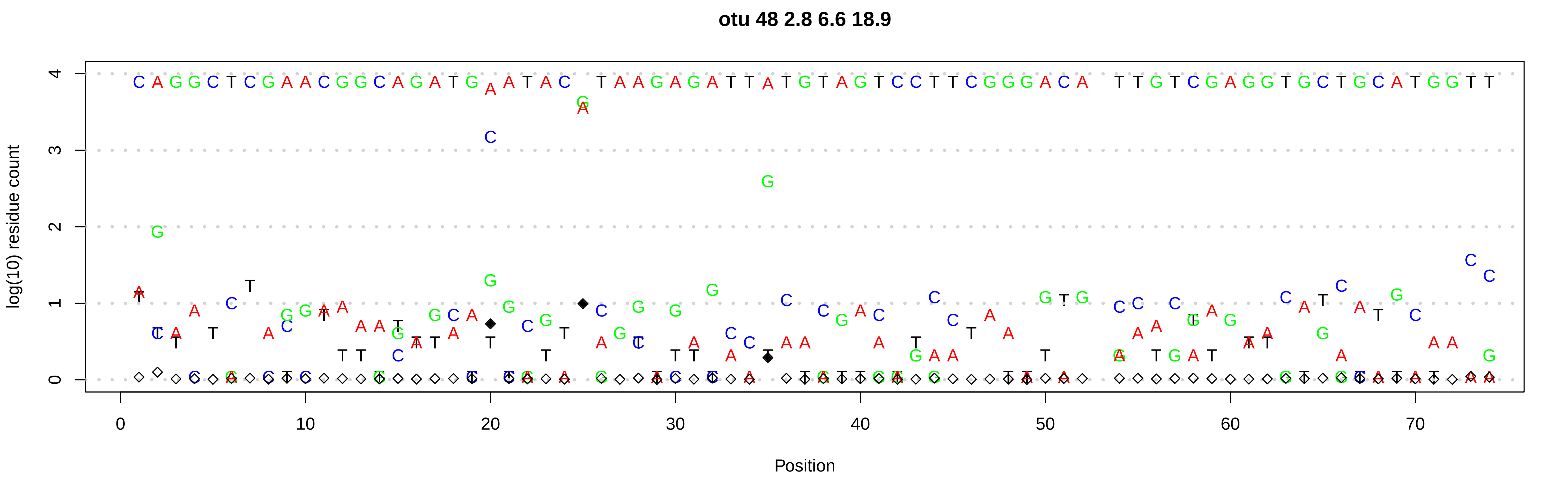
otu 48 2.8 6.6 18.9
log(10) residue count
Position
0
1
2
3
4
0
10
20
30
40
50
60
70

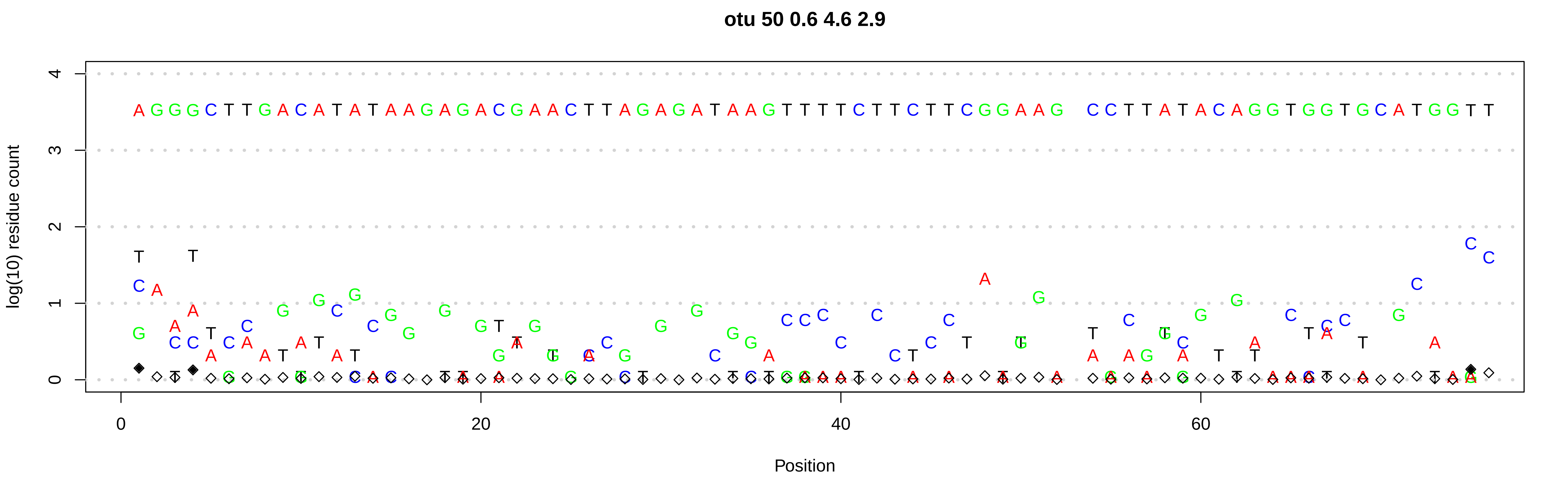
otu 50 0.6 4.6 2.9
log(10) residue count
Position
0
1
2
3
4
0
20
40
60

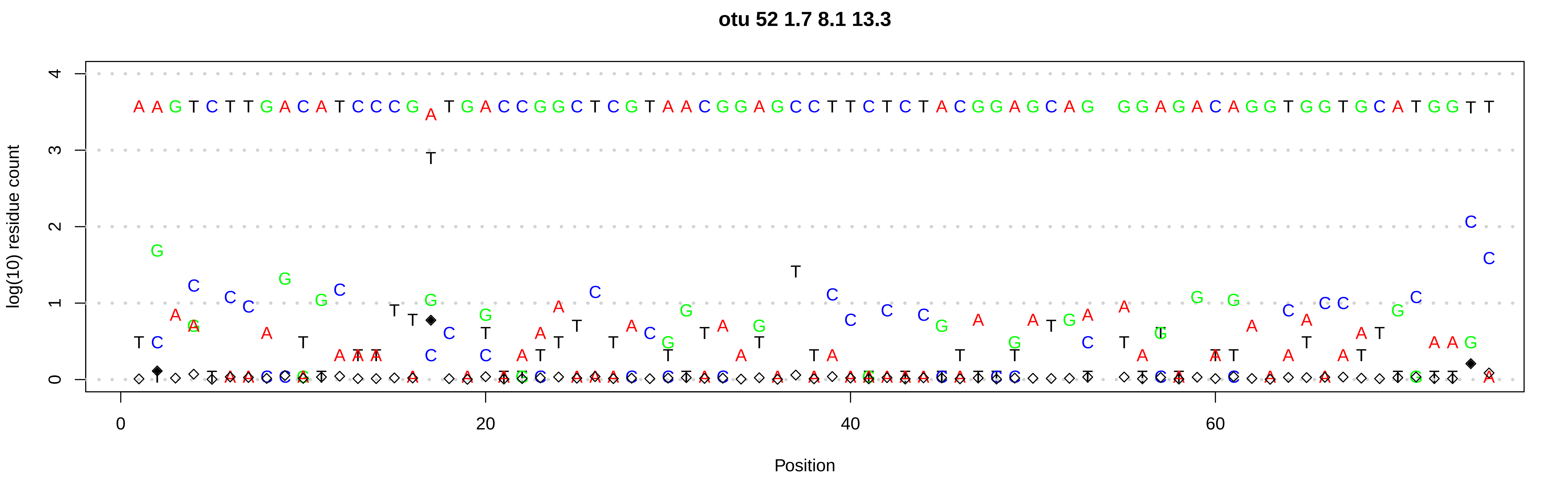

otu 52 1.7 8.1 13.3
log(10) residue count
0
1
2
3
4
Position
0
20
40
60

otu 56 3.0 4.3 1.9

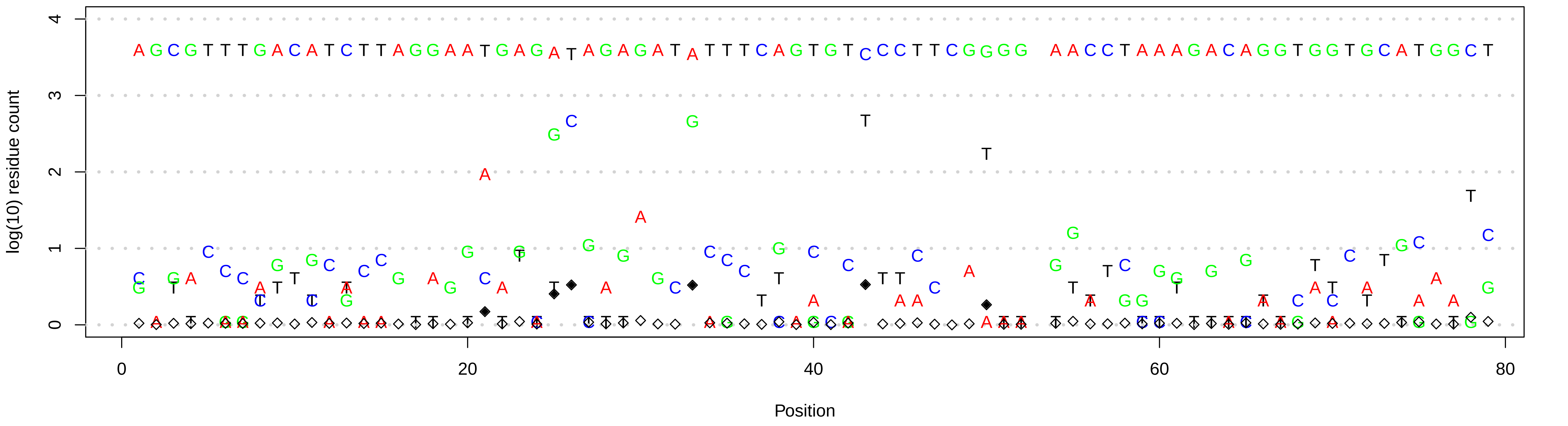

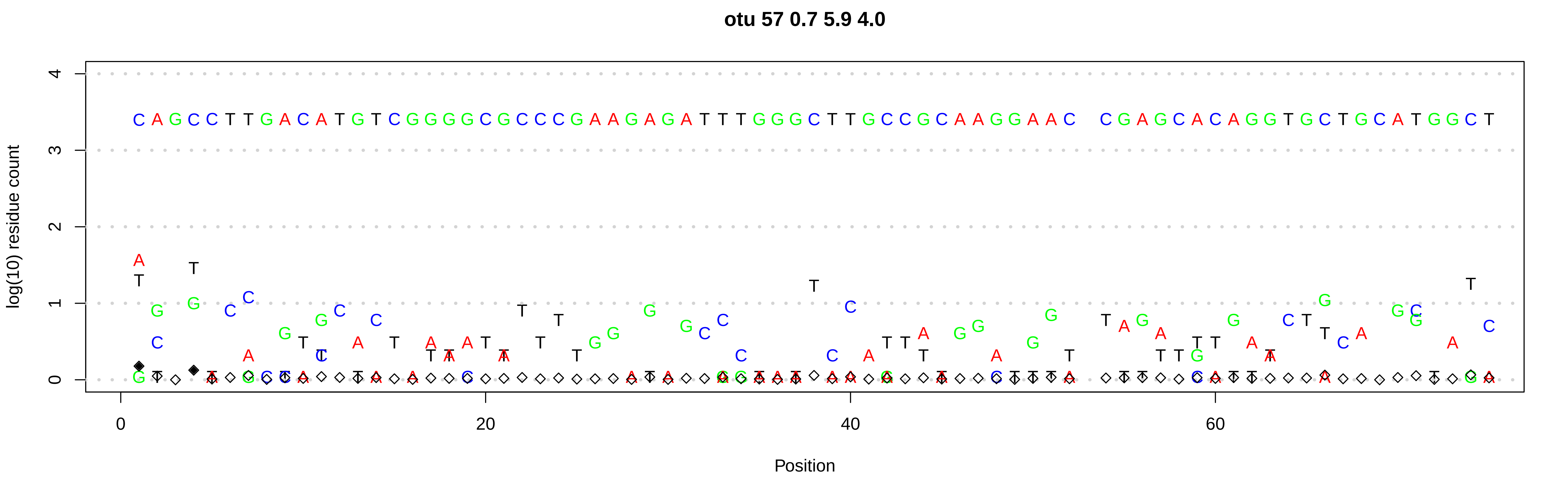
otu 57 0.7 5.9 4.0
log(10) residue count
Position
0
1
2
3
4
0
20
40
60
CAGCCTTGACATGTCGGGGCGCCCGAAGAGATTTGGGCTTGCCGCAAGGAAC CGAGCACAGGTGCTGCATGGCT

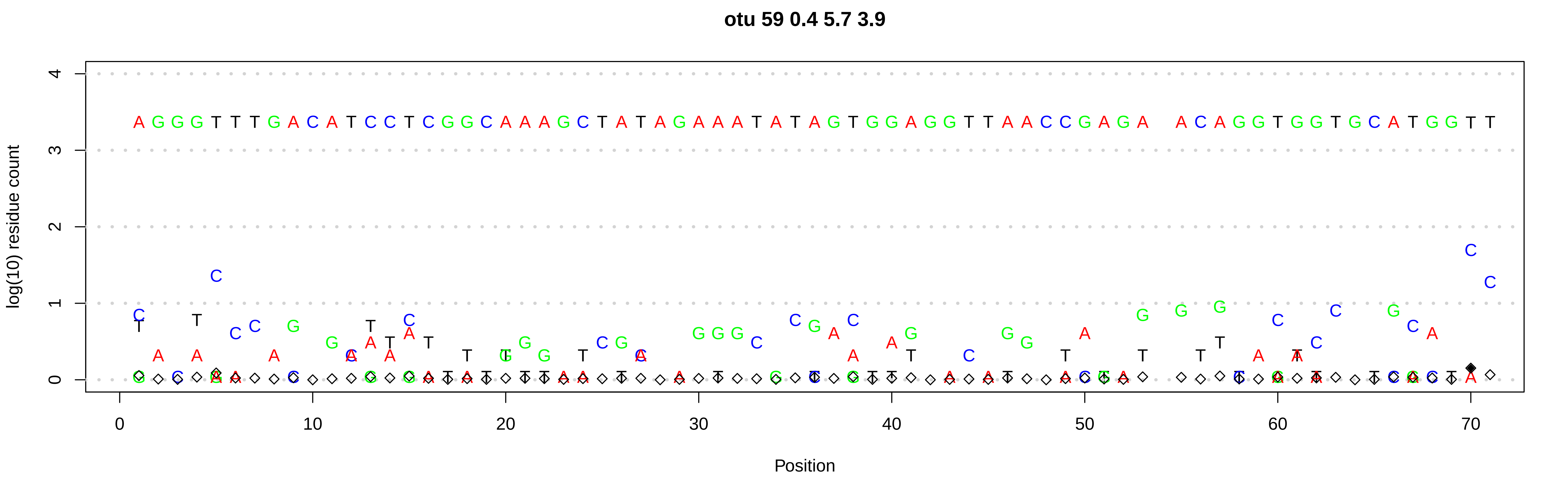
otu 59 0.4 5.7 3.9
A G G G T T T G A C A T C C T C G G C A A A G C T A T A G A A A T A T A G T G G A G G T T A A C C G A G A A C A G G T G G T G C A T G G T T
log(10) residue count
Position
0
1
2
3
4
0
10
20
30
40
50
60
70

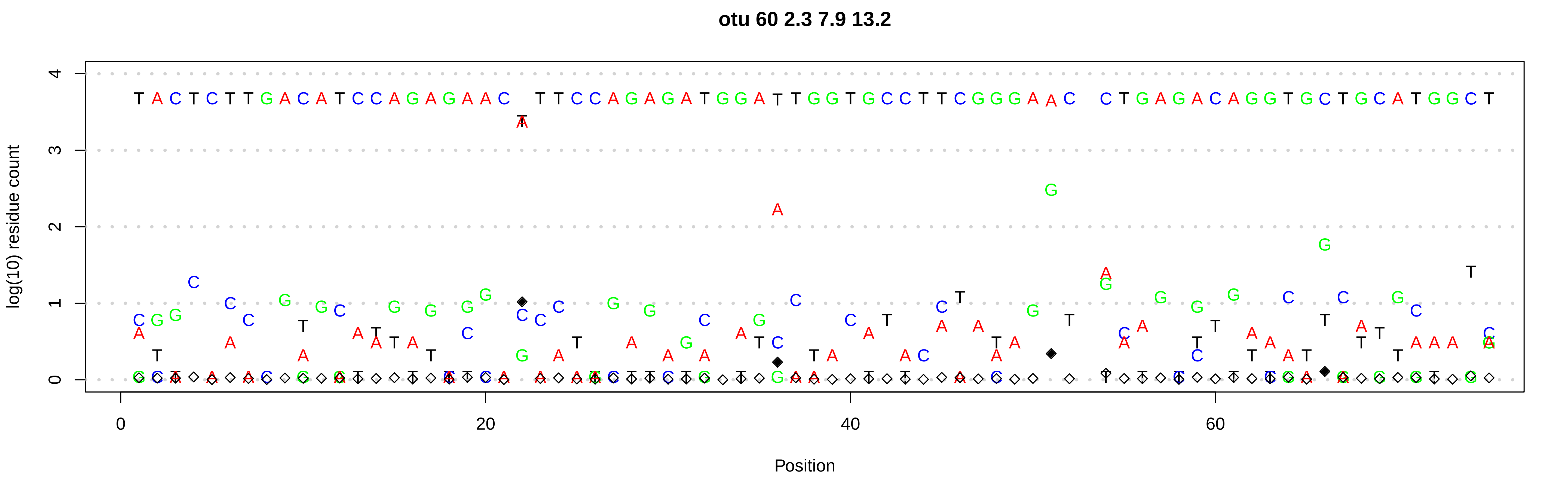
otu 60 2.3 7.9 13.2
log(10) residue count
Position
0
1
2
3
4
0
20
40
60

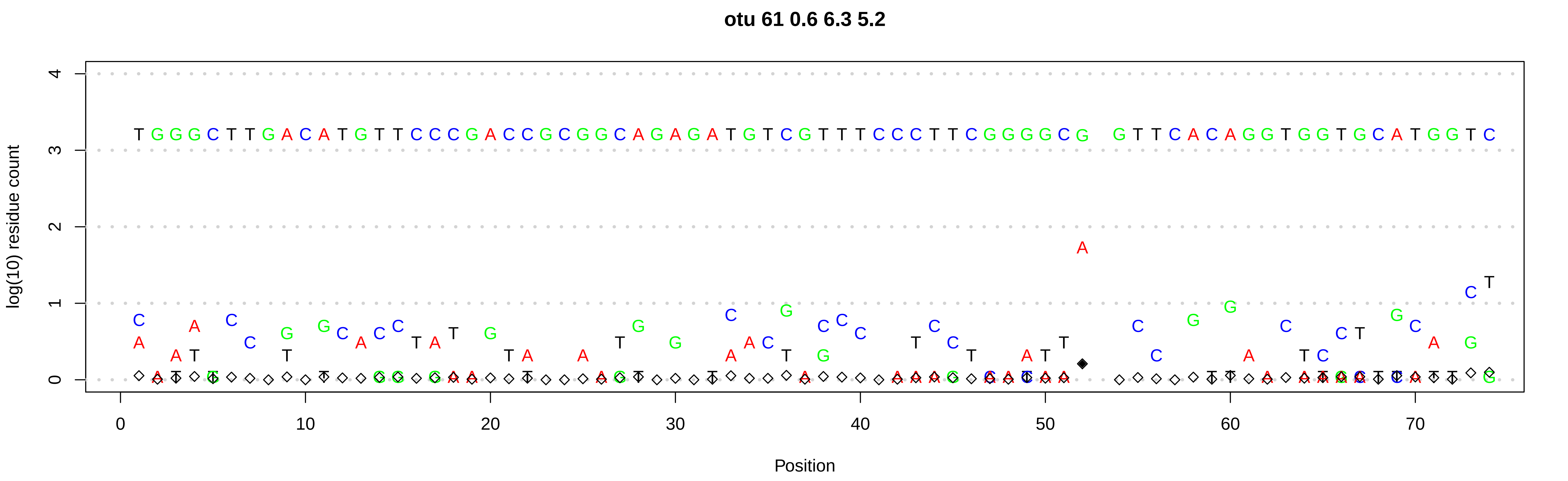

otu 61 0.6 6.3 5.2
log(10) residue count
Position

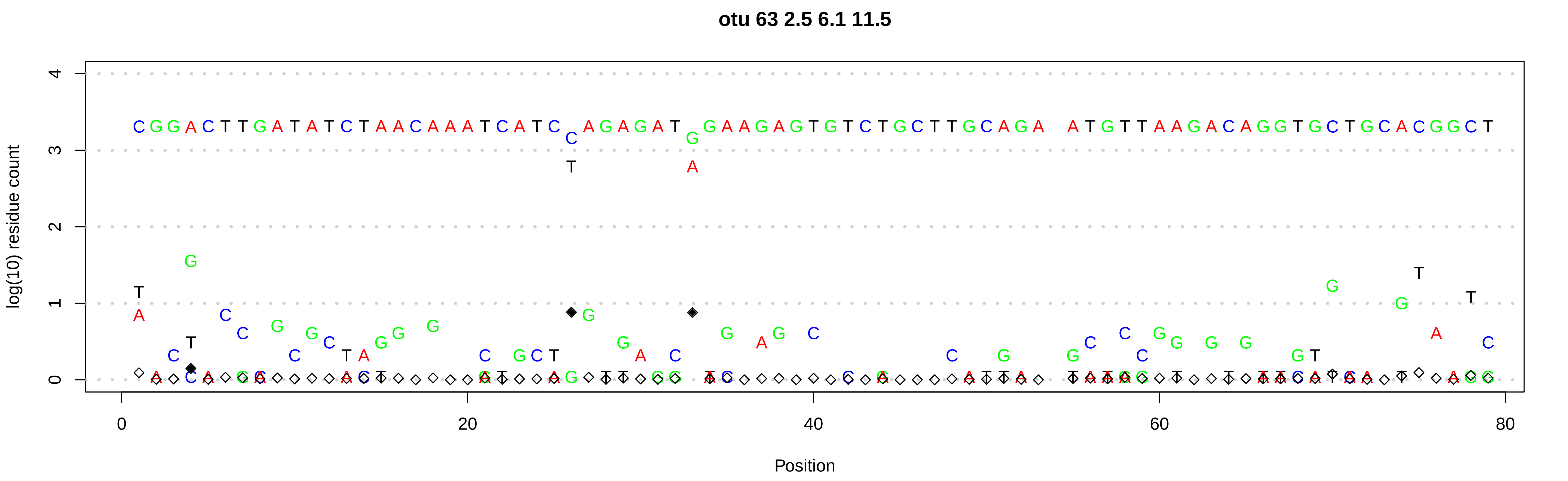
otu 63 2.5 6.1 11.5
log(10) residue count
Position
0
1
2
3
4
0
20
40
60
80

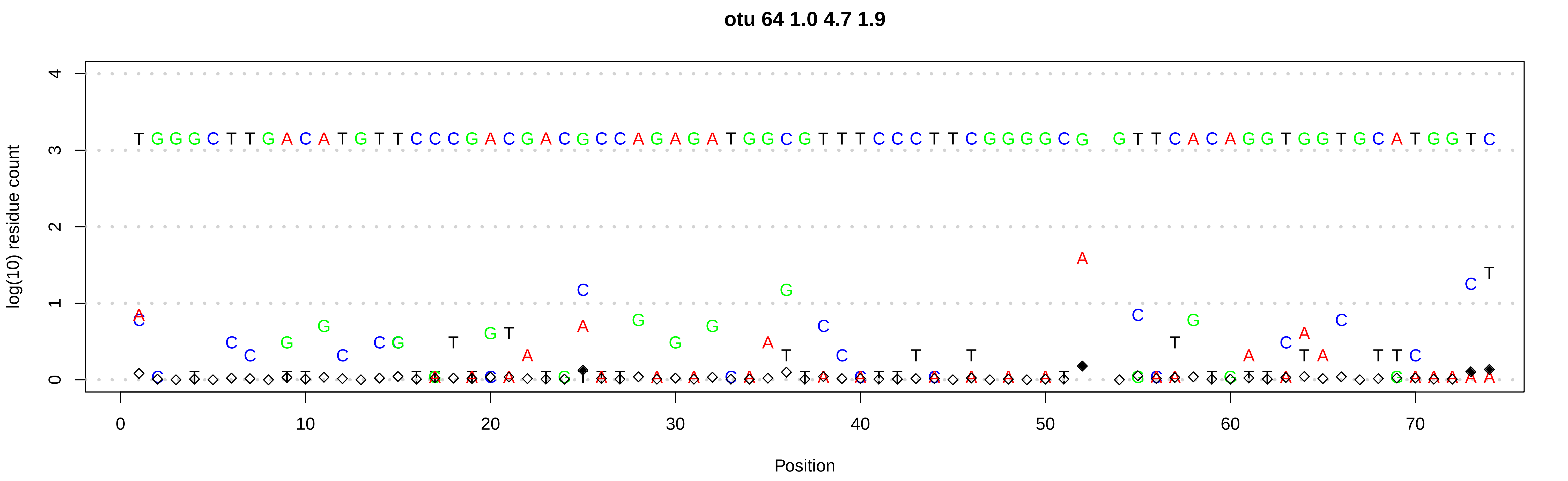
otu 64 1.0 4.7 1.9
log(10) residue count
Position
0
10
20
30
40
50
60
70
0
1
2
3
4

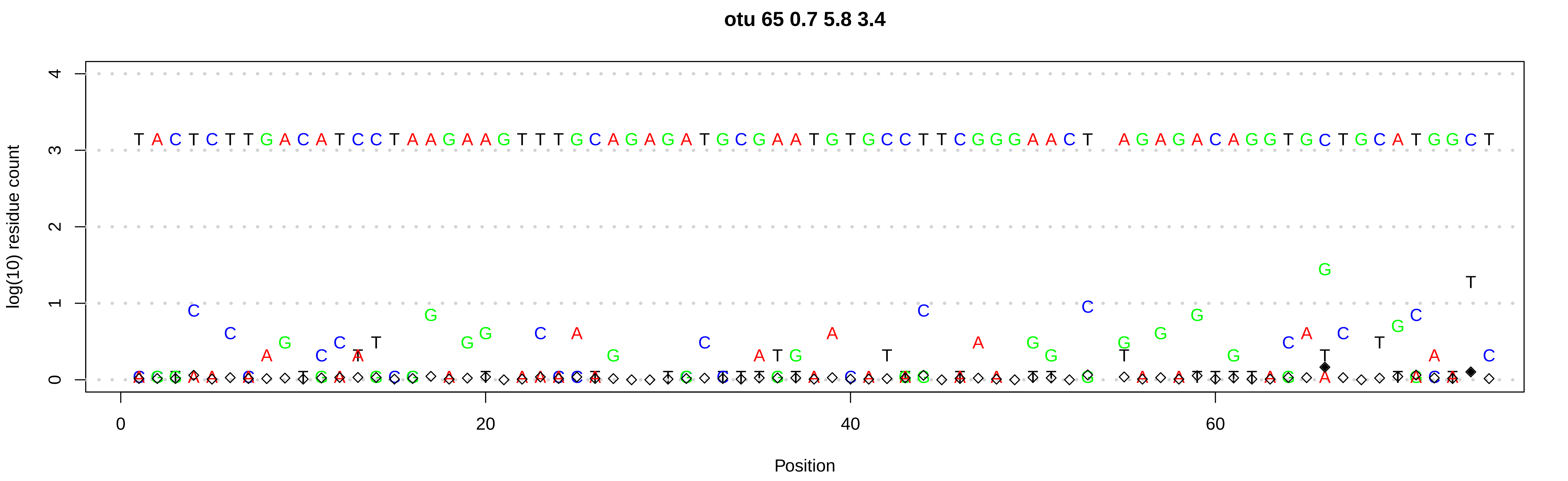
otu 65 0.7 5.8 3.4
T A C T C T T G A C A T C C T A A G A A G T T T G C A G A G A T G C G A A T G T G C C T T C G G G A A C T A G A G A C A G G T G C T G C A T G G C T
log(10) residue count
0
1
2
3
4
0
20
40
60
Position

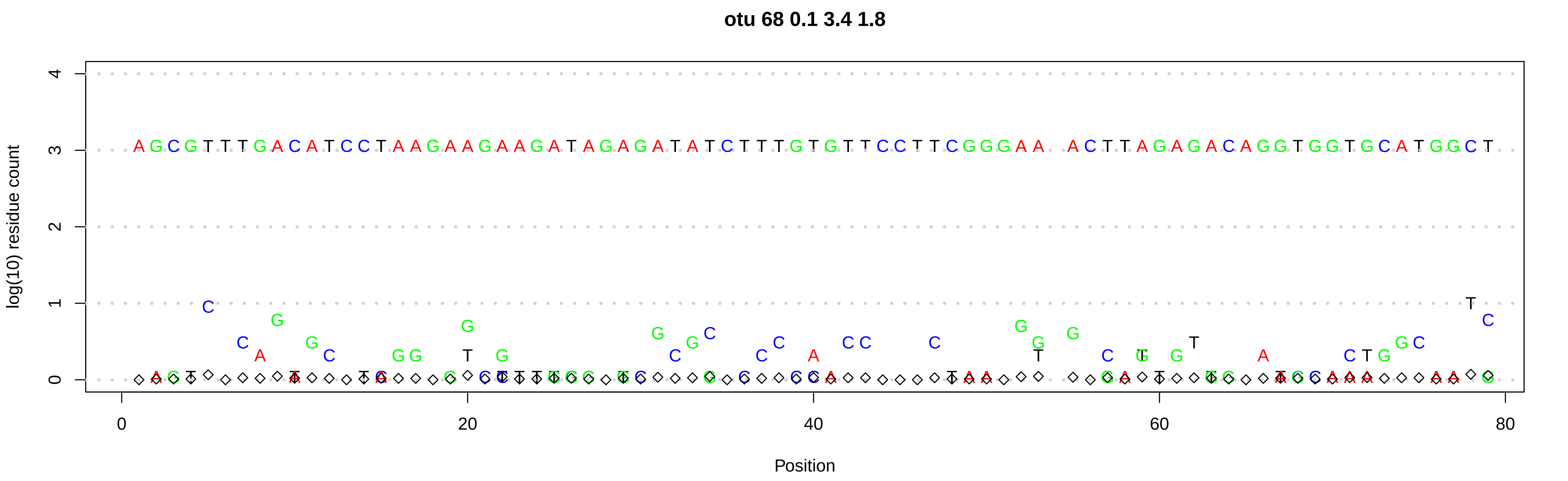
otu 68 0.1 3.4 1.8
log(10) residue count
Position
AGCGTTTGACATCCTAAGAAGAAGATAGAGATATCTTTGTGTTCCTTCGGGAA ACTTAGAGACAGGTGGTGCATGGCT

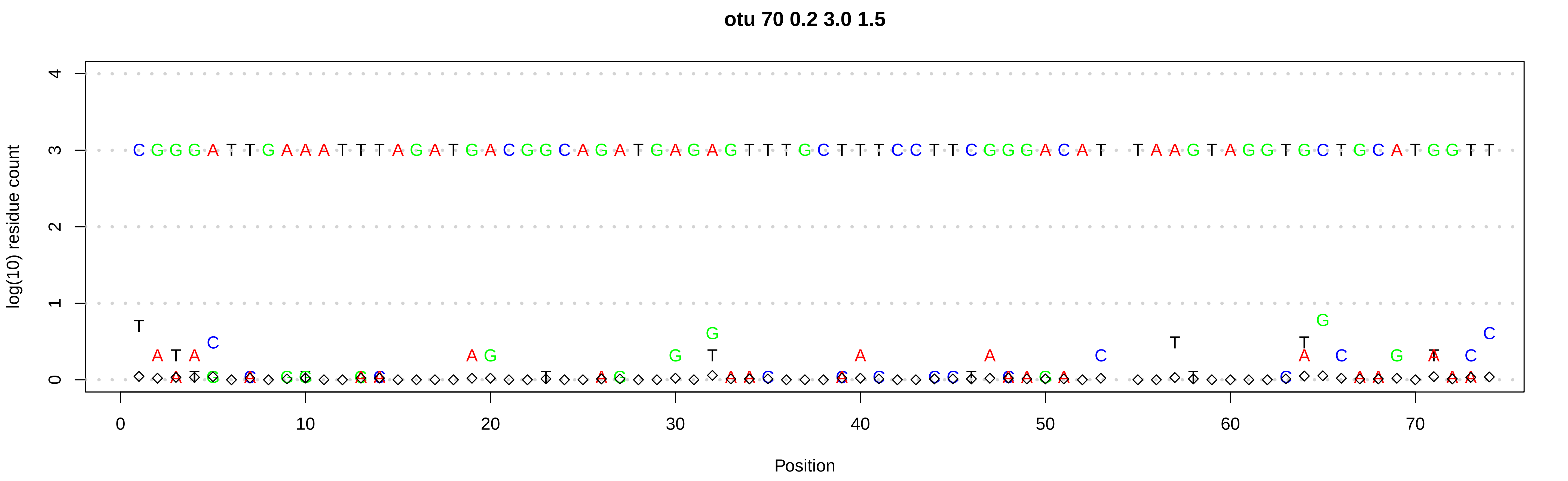
otu 70 0.2 3.0 1.5
log(10) residue count
Position

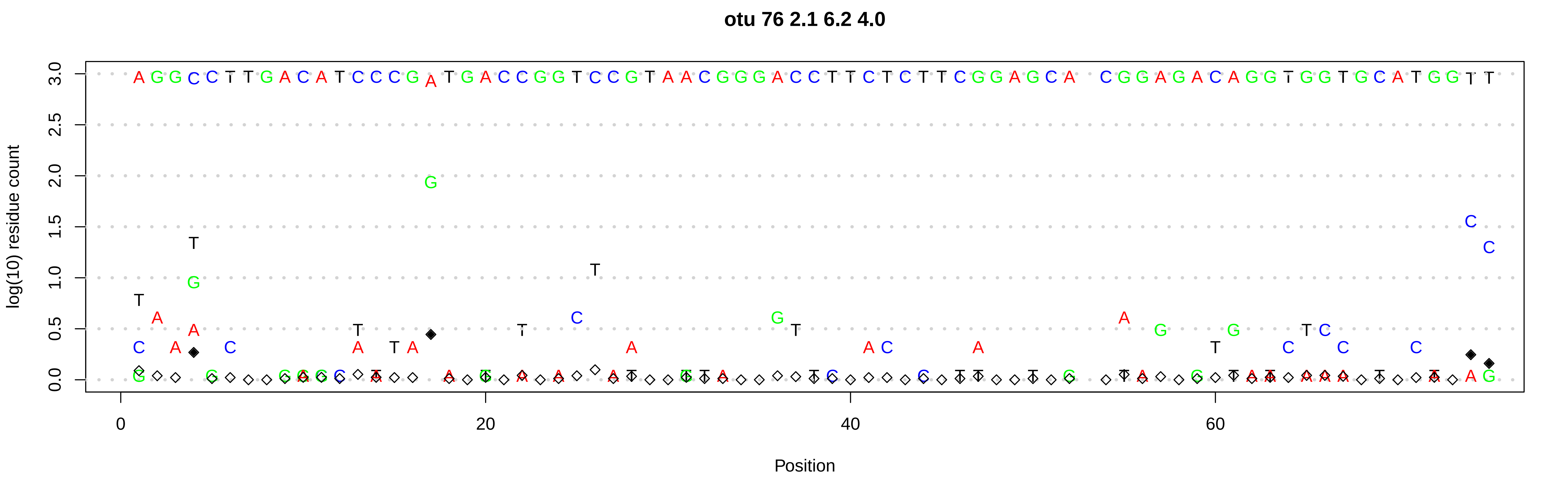
otu 76 2.1 6.2 4.0
log(10) residue count
0.0
0.5
1.0
1.5
2.0
2.5
3.0
Position
0
20
40
60

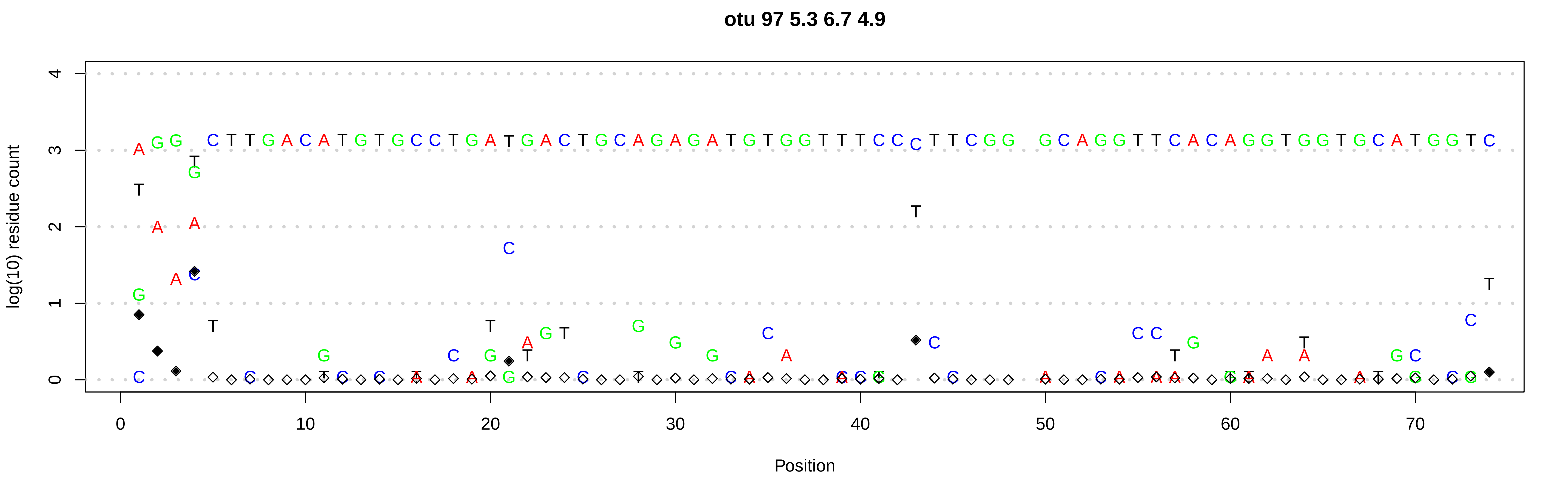
otu 97 5.3 6.7 4.9
log(10) residue count
0
1
2
3
4
Position
0
10
20
30
40
50
60
70

otu 102 1.0 4.2 2.2

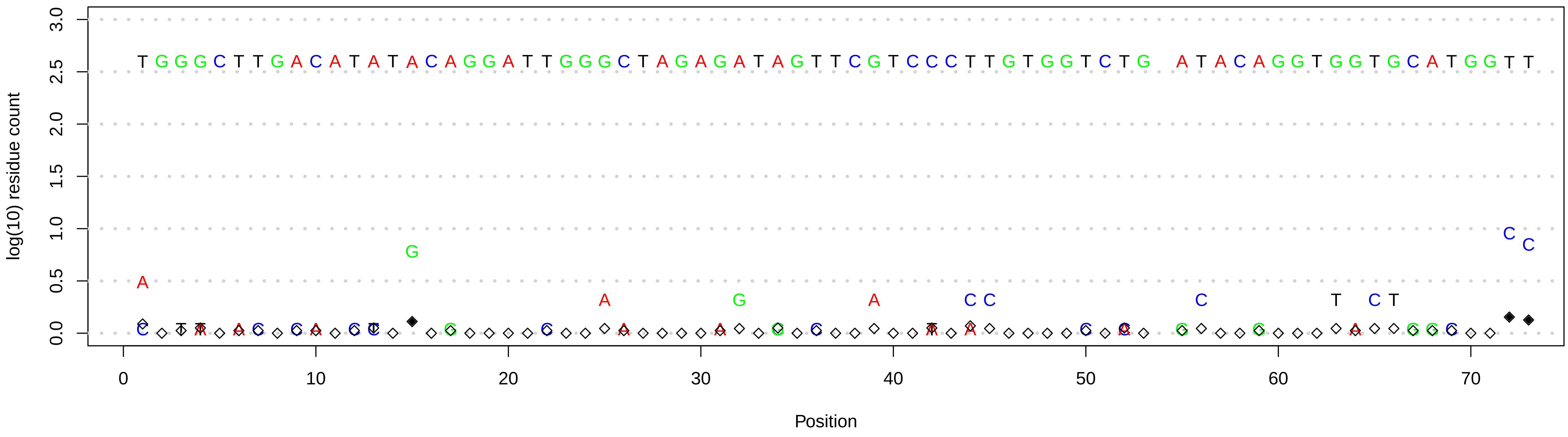

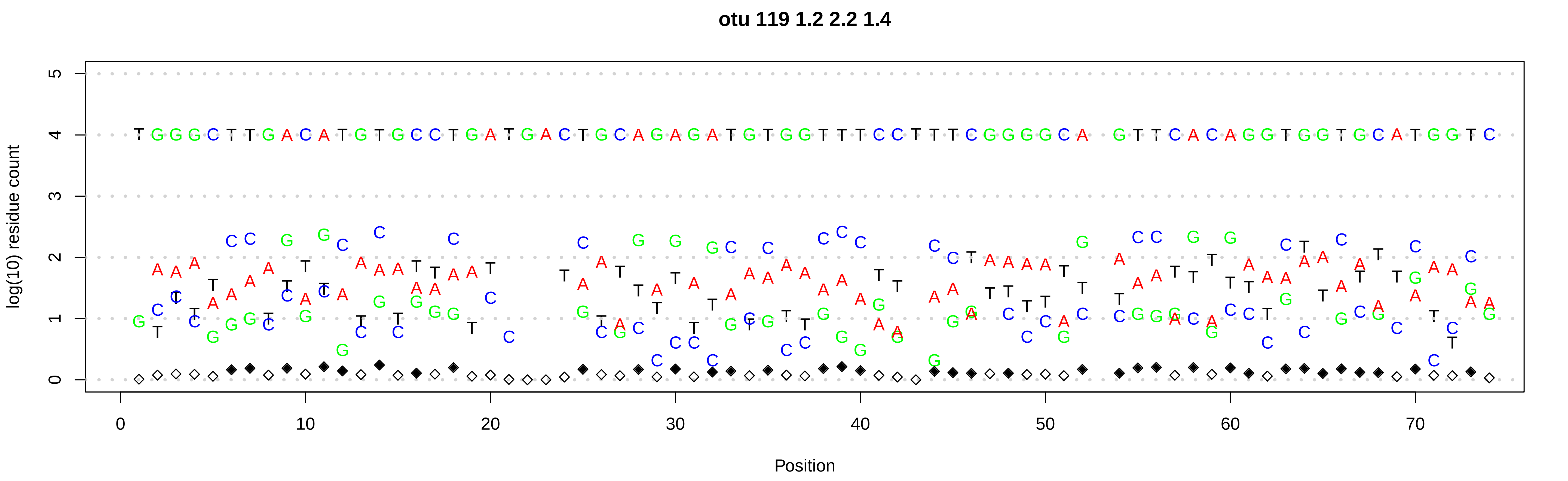
otu 119 1.2 2.2 1.4
log(10) residue count
Position
