# Supplementary material for: Microbiome Profiling by Illumina Sequencing of Combinatorial Sequence-Tagged PCR Products
Source: PLoS One. 2010 Oct 26;5(10):e15406. doi: 10.1371/journal.pone.0015406 (PMC2964327; doi:10.1371/journal.pone.0015406)
Supplement: Figure S2 — Rarefaction curves, calculated as described in the text, for each of the OTUs. (PDF) [file pone.0015406.s002.pdf]

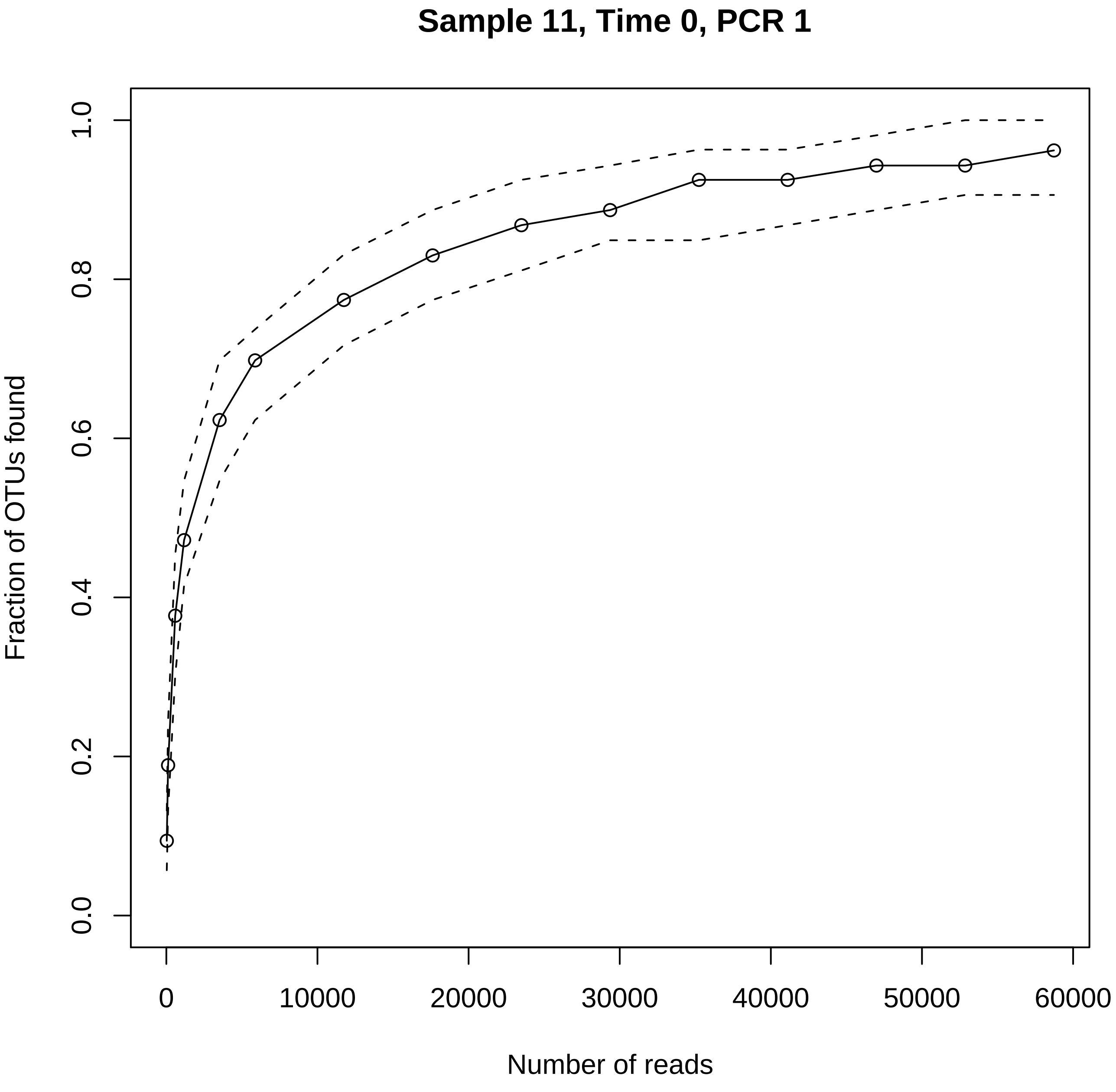
Sample 11, Time 0, PCR 1
Fraction of OTUs found
Number of reads
0.0
0.2
0.4
0.6
0.8
1.0
0
10000
20000
30000
40000
50000
60000

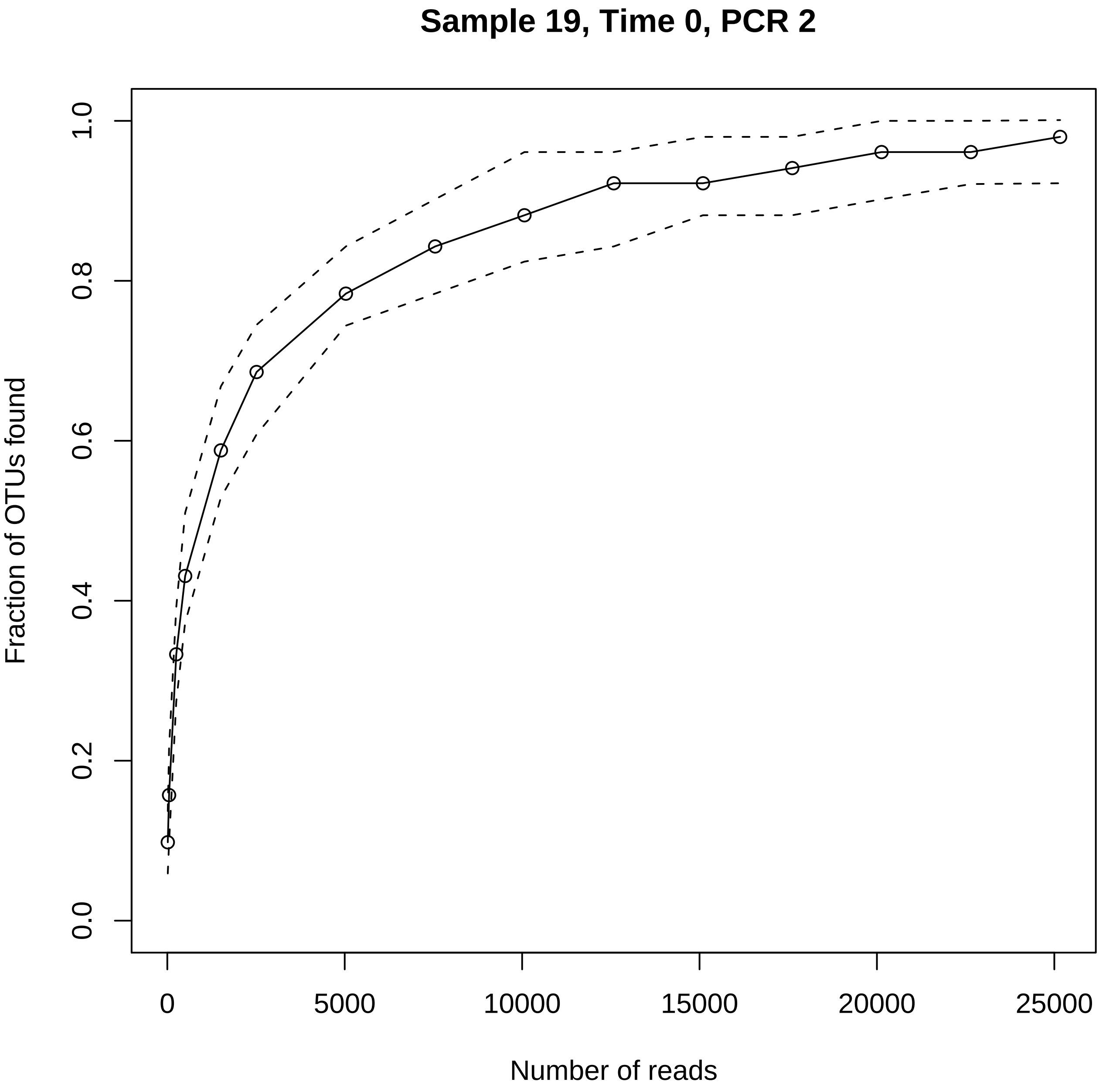
Sample 19, Time 0, PCR 2
Fraction of OTUs found
0.0
0.2
0.4
0.6
0.8
1.0
0
5000
10000
15000
20000
25000
Number of reads

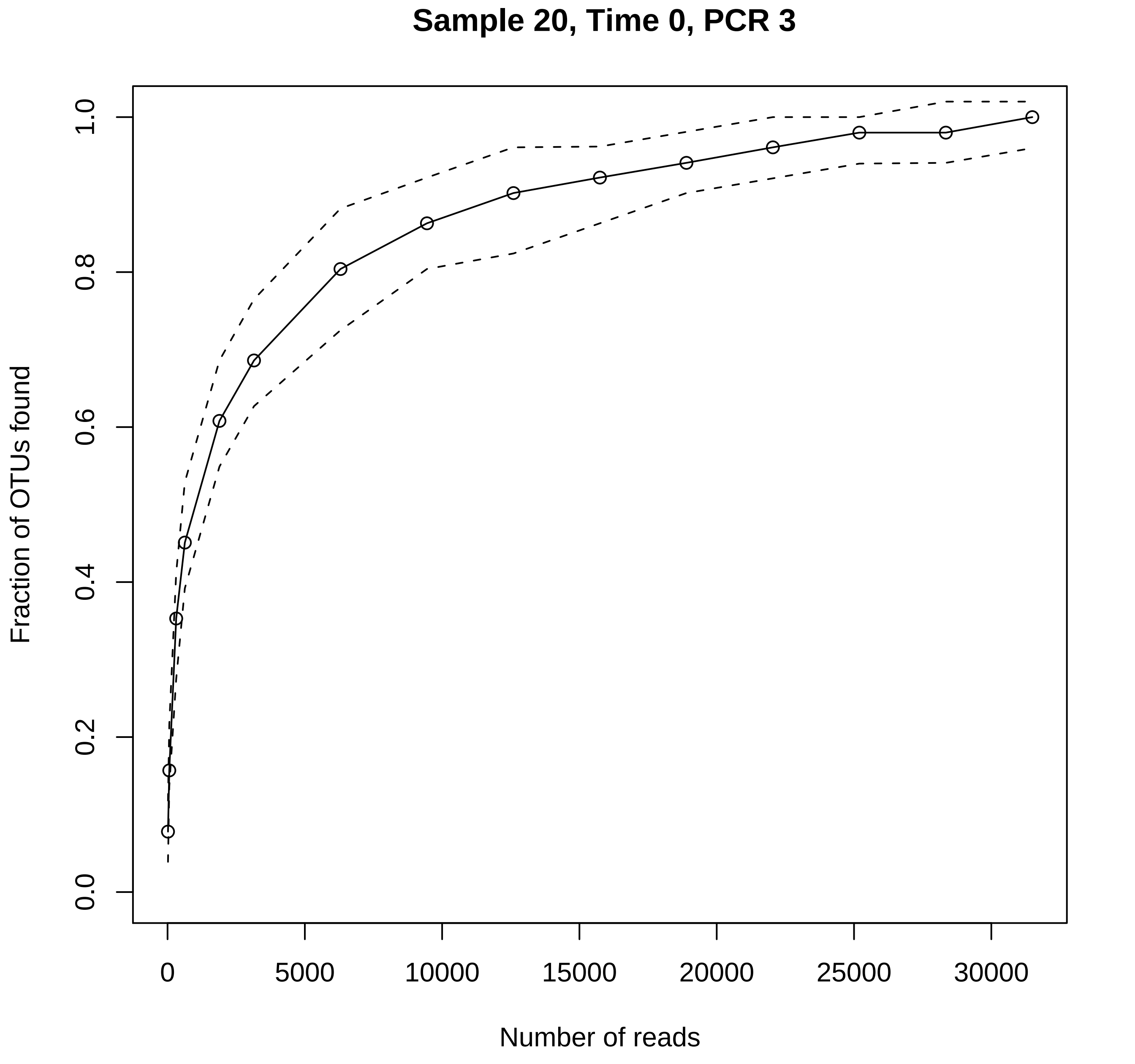
Sample 20, Time 0, PCR 3
Fraction of OTUs found
0.0
0.2
0.4
0.6
0.8
1.0
0
5000
10000
15000
20000
25000
30000
Number of reads

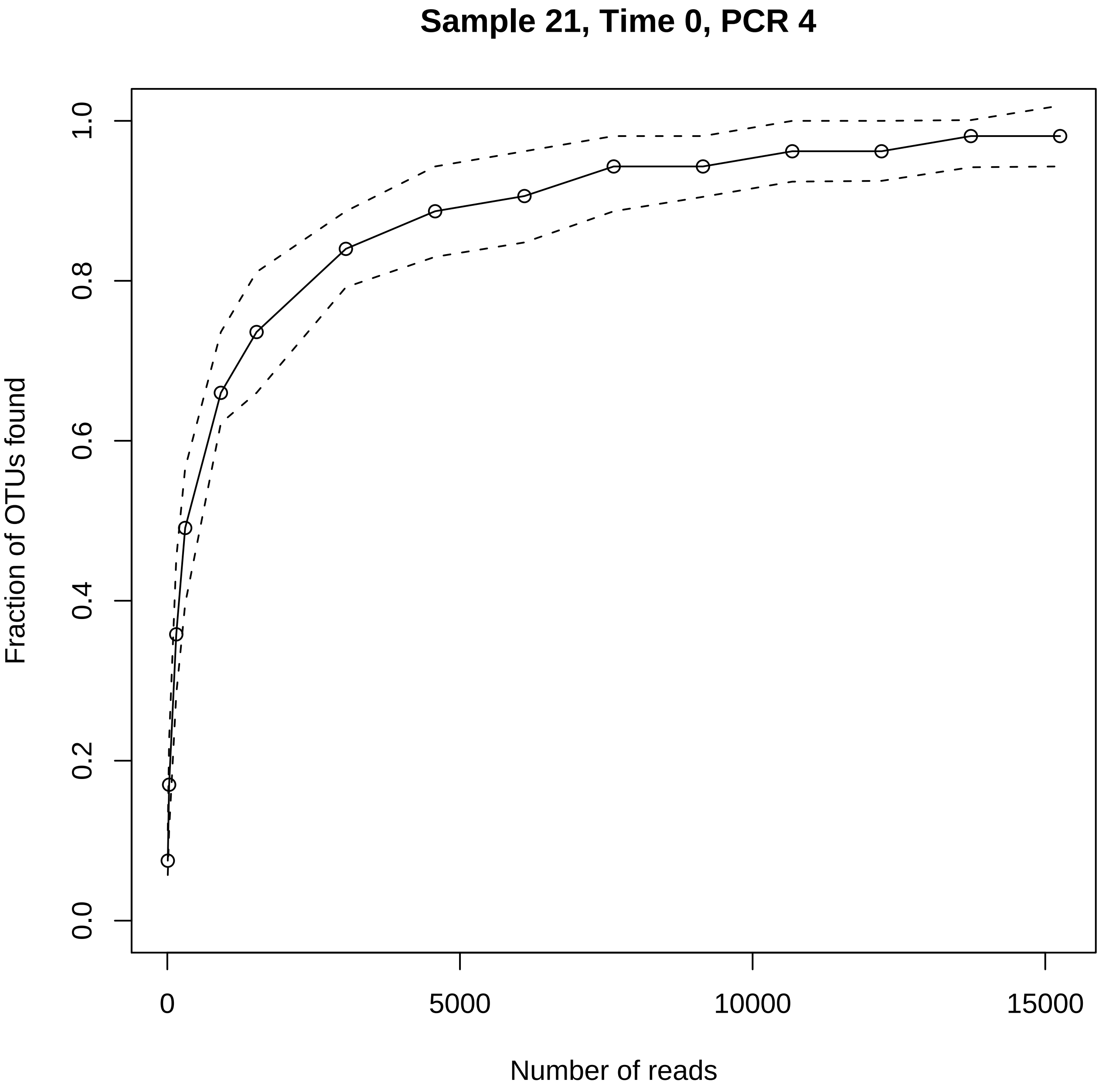

Sample 21, Time 0, PCR 4
Fraction of OTUs found
0.0
0.2
0.4
0.6
0.8
1.0
0
5000
10000
15000
Number of reads

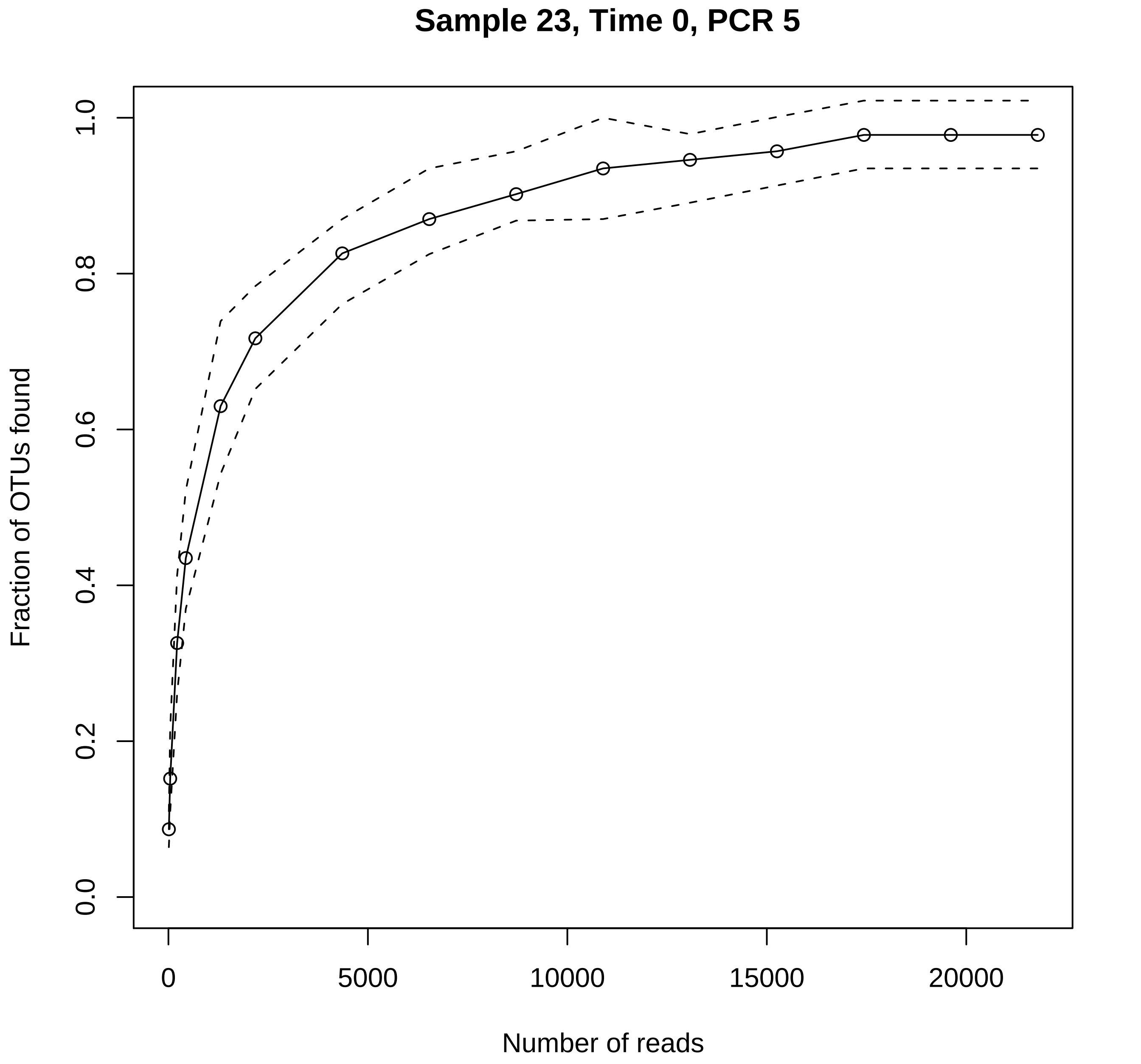
Sample 23, Time 0, PCR 5
Fraction of OTUs found
0.0
0.2
0.4
0.6
0.8
1.0
0
5000
10000
15000
20000
Number of reads

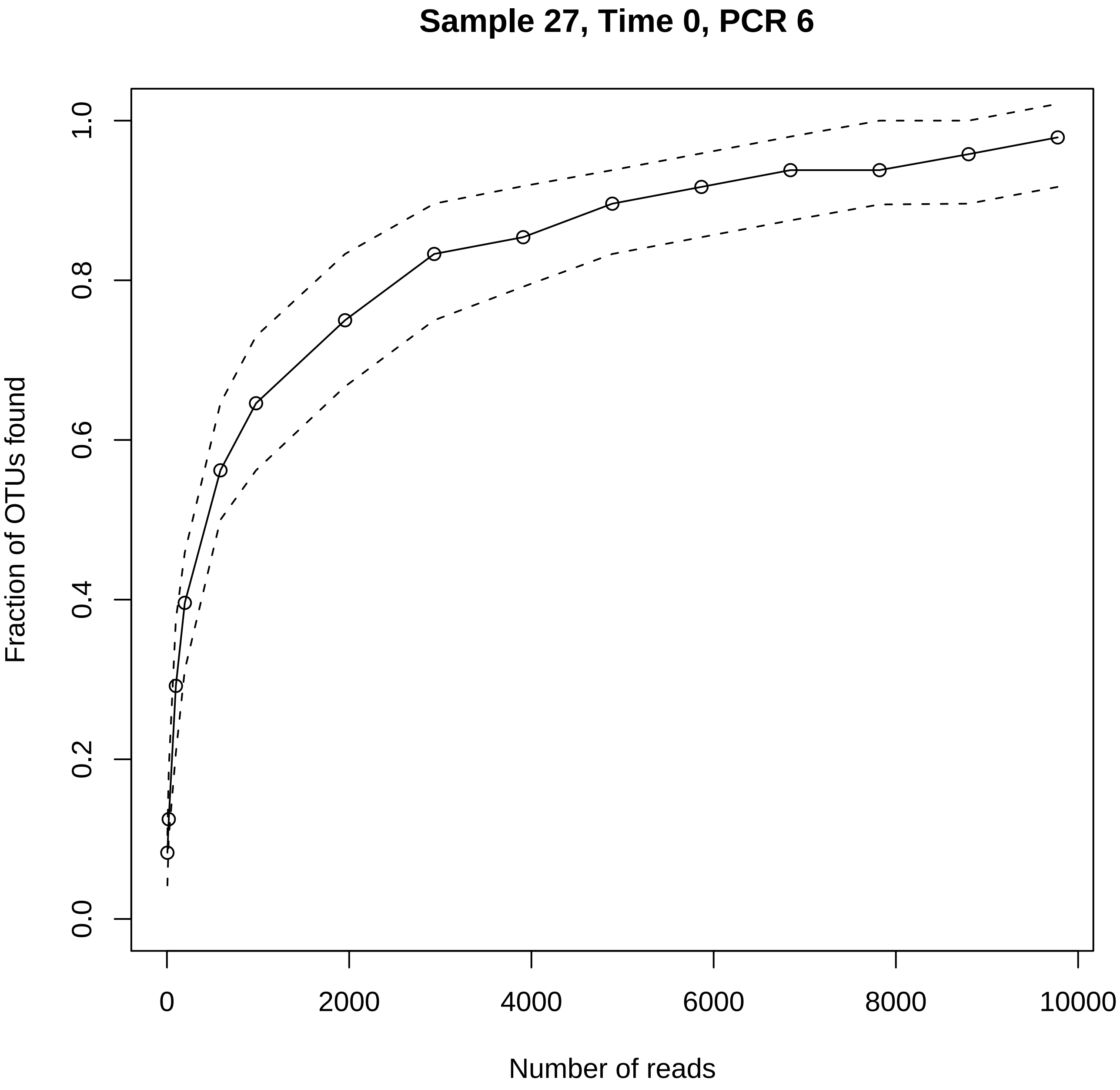
Sample 27, Time 0, PCR 6
Fraction of OTUs found
0.0
0.2
0.4
0.6
0.8
1.0
0
2000
4000
6000
8000
10000
Number of reads

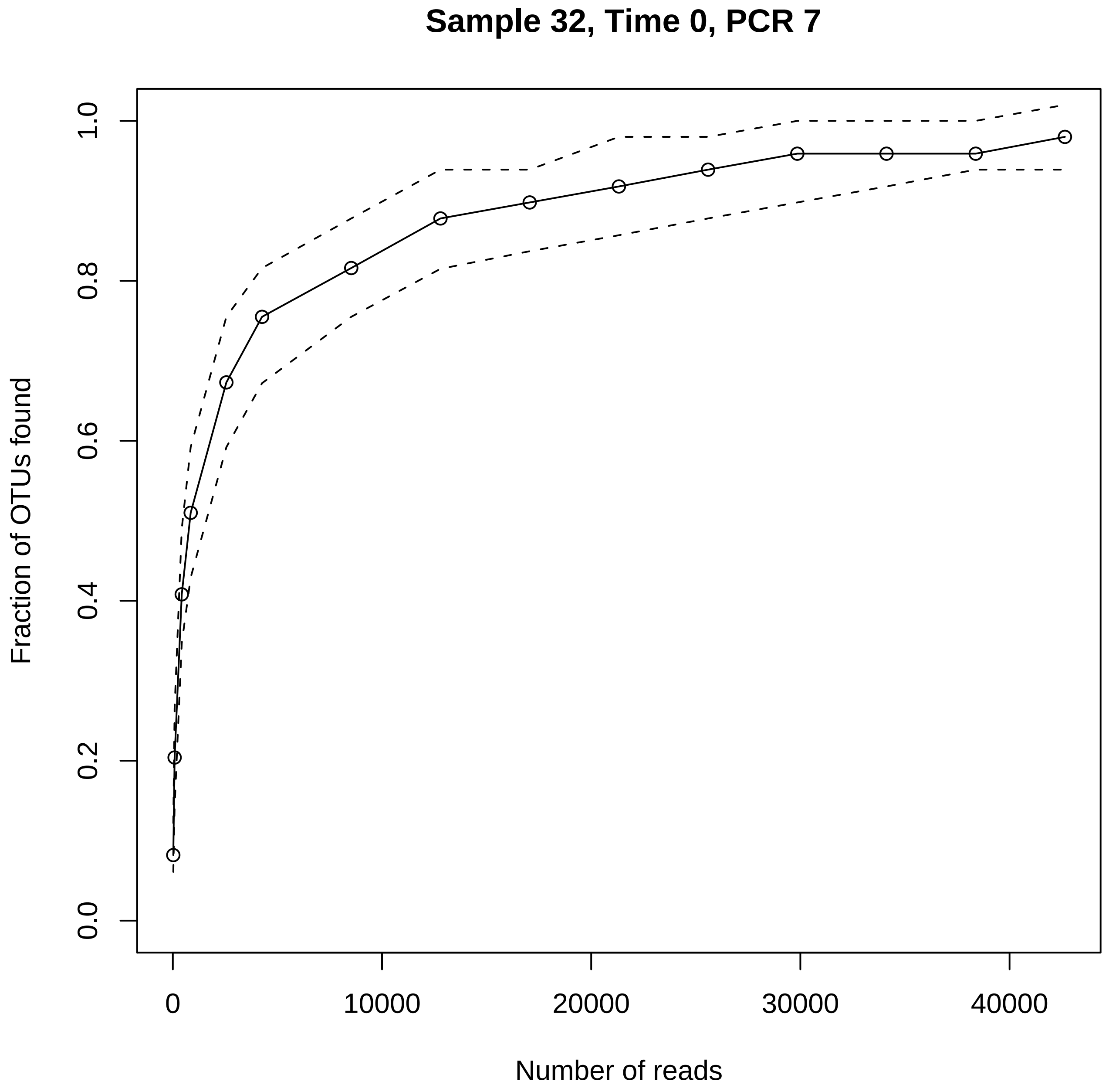

Sample 32, Time 0, PCR 7
Fraction of OTUs found
0.0
0.2
0.4
0.6
0.8
1.0
0
10000
20000
30000
40000
Number of reads

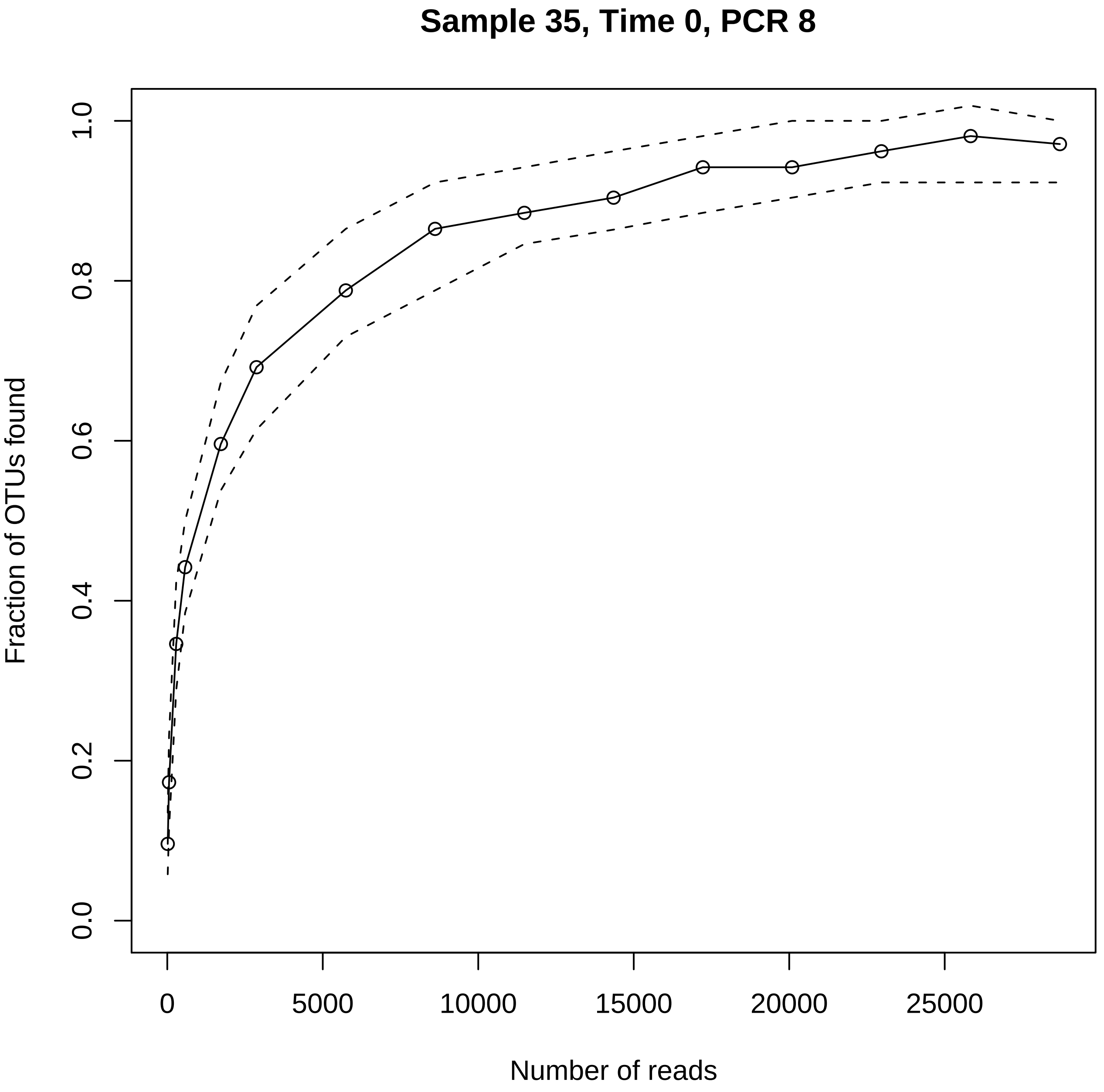
Sample 35, Time 0, PCR 8
Fraction of OTUs found
0.0
0.2
0.4
0.6
0.8
1.0
0
5000
10000
15000
20000
25000
Number of reads

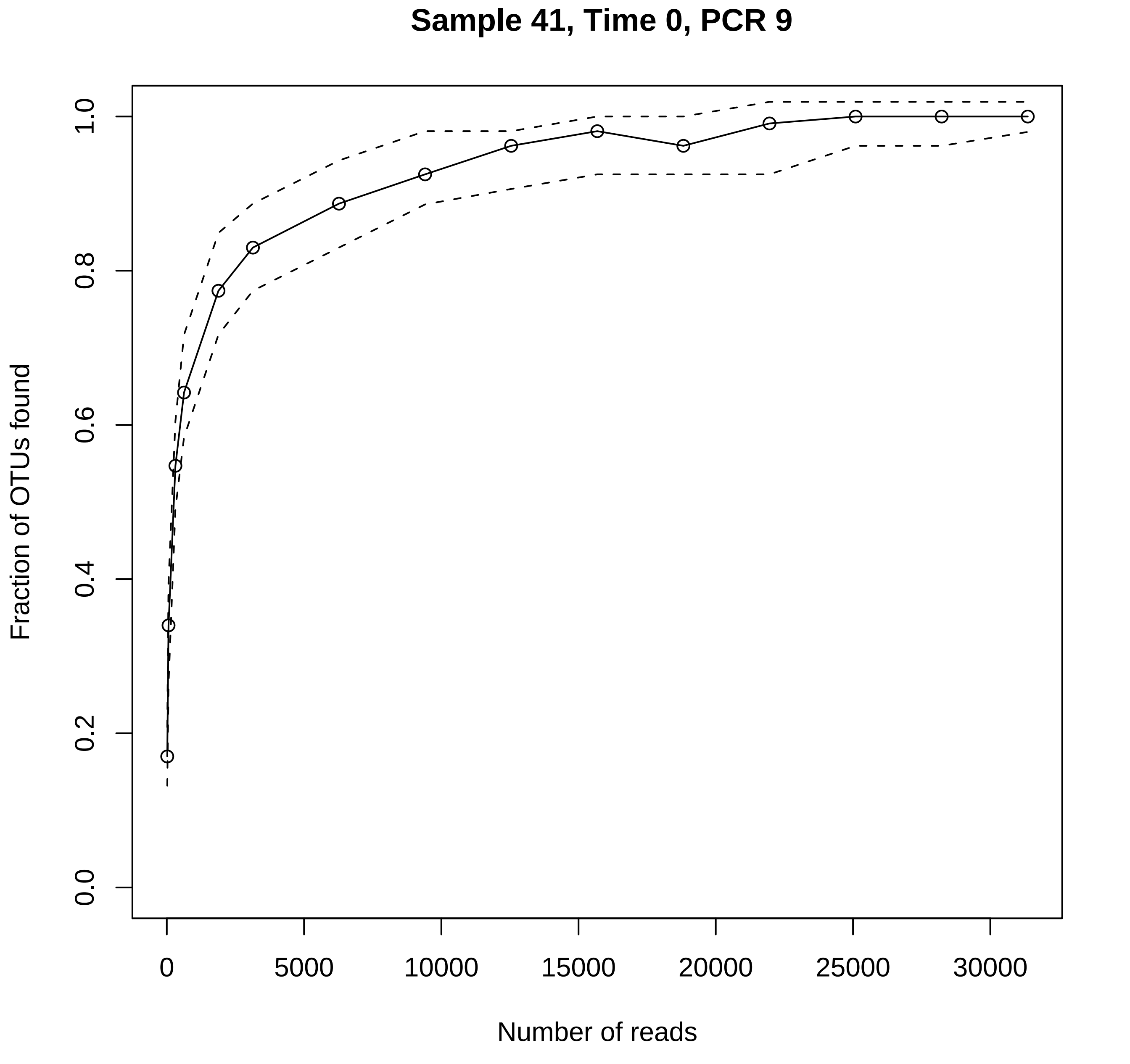
Sample 41, Time 0, PCR 9
Fraction of OTUs found
0.0
0.2
0.4
0.6
0.8
1.0
Number of reads
0
5000
10000
15000
20000
25000
30000

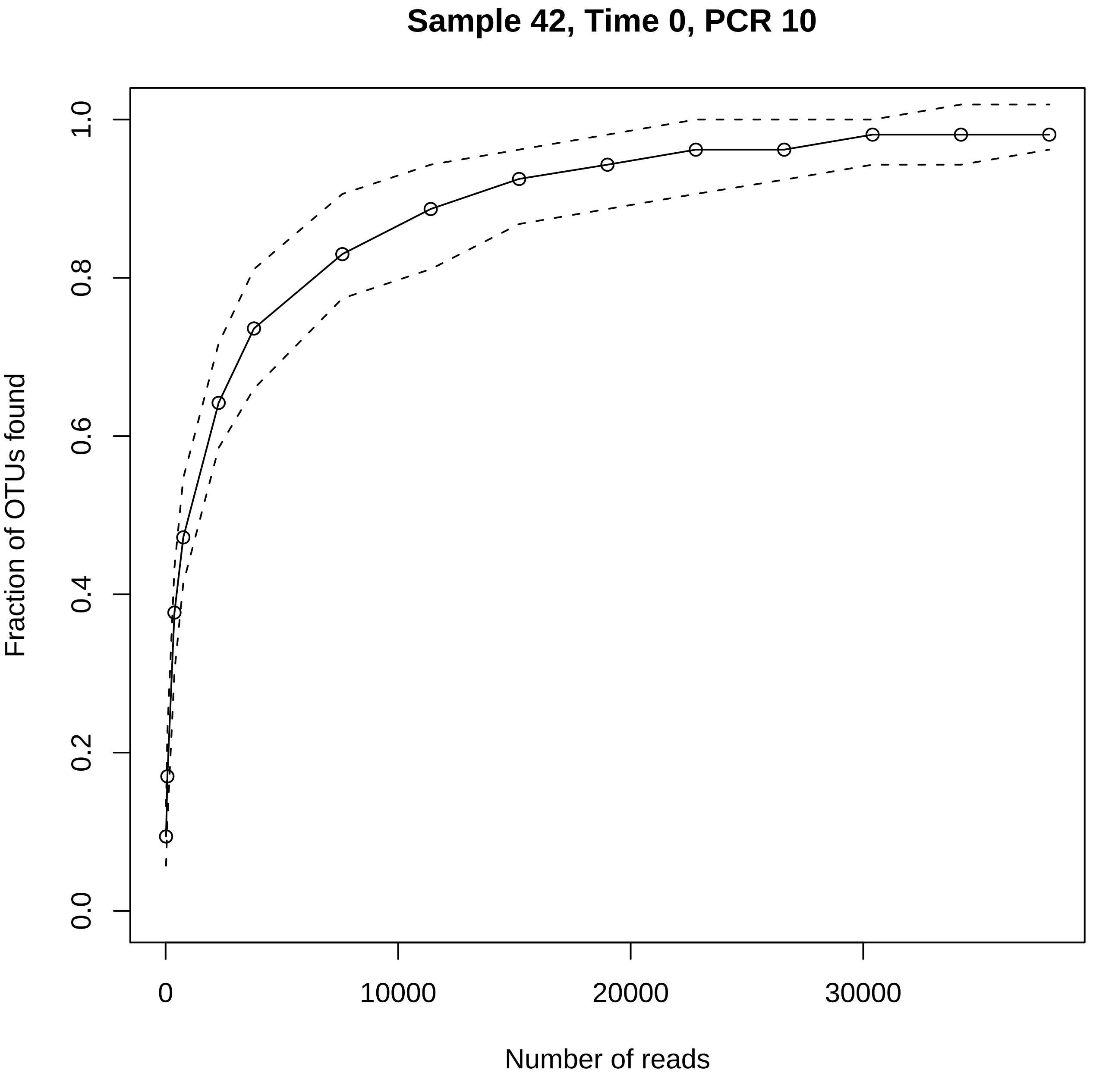

Sample 42, Time 0, PCR 10
Fraction of OTUs found
Number of reads
0.0
0.2
0.4
0.6
0.8
1.0
0
10000
20000
30000

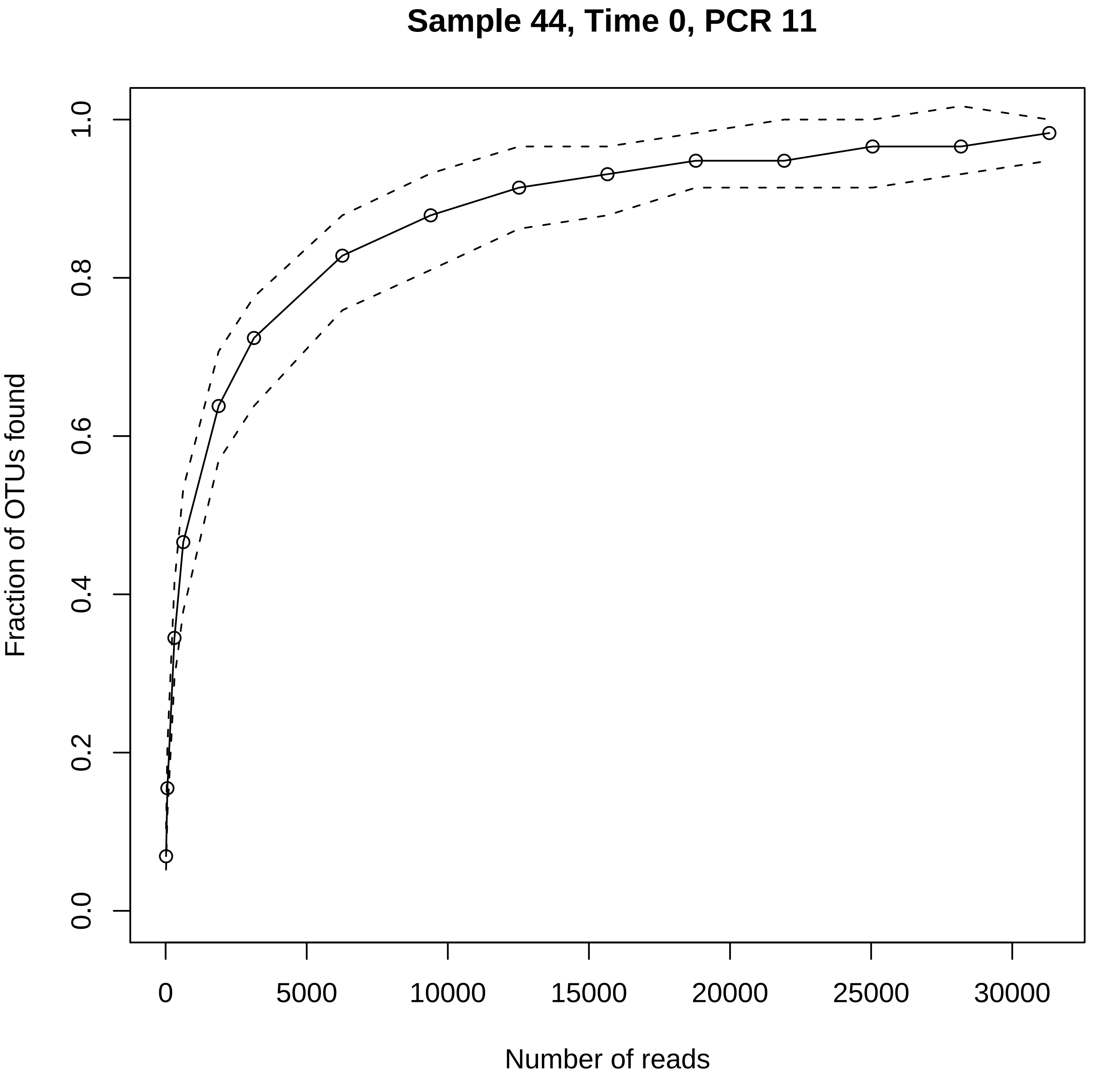
Sample 44, Time 0, PCR 11
Fraction of OTUs found
0.0
0.2
0.4
0.6
0.8
1.0
0
5000
10000
15000
20000
25000
30000
Number of reads

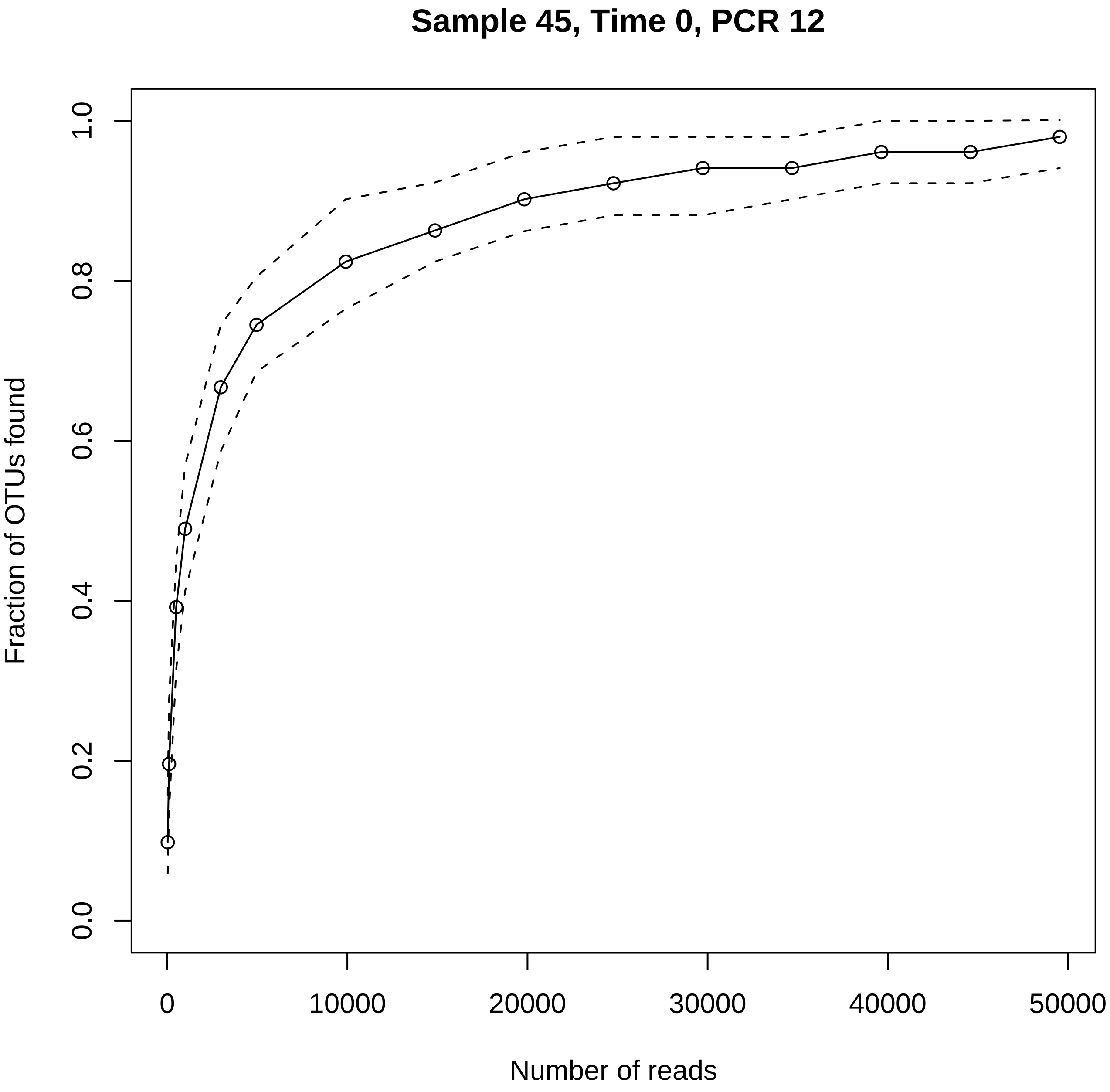

Sample 45, Time 0, PCR 12
Fraction of OTUs found
0.0
0.2
0.4
0.6
0.8
1.0
0
10000
20000
30000
40000
50000
Number of reads

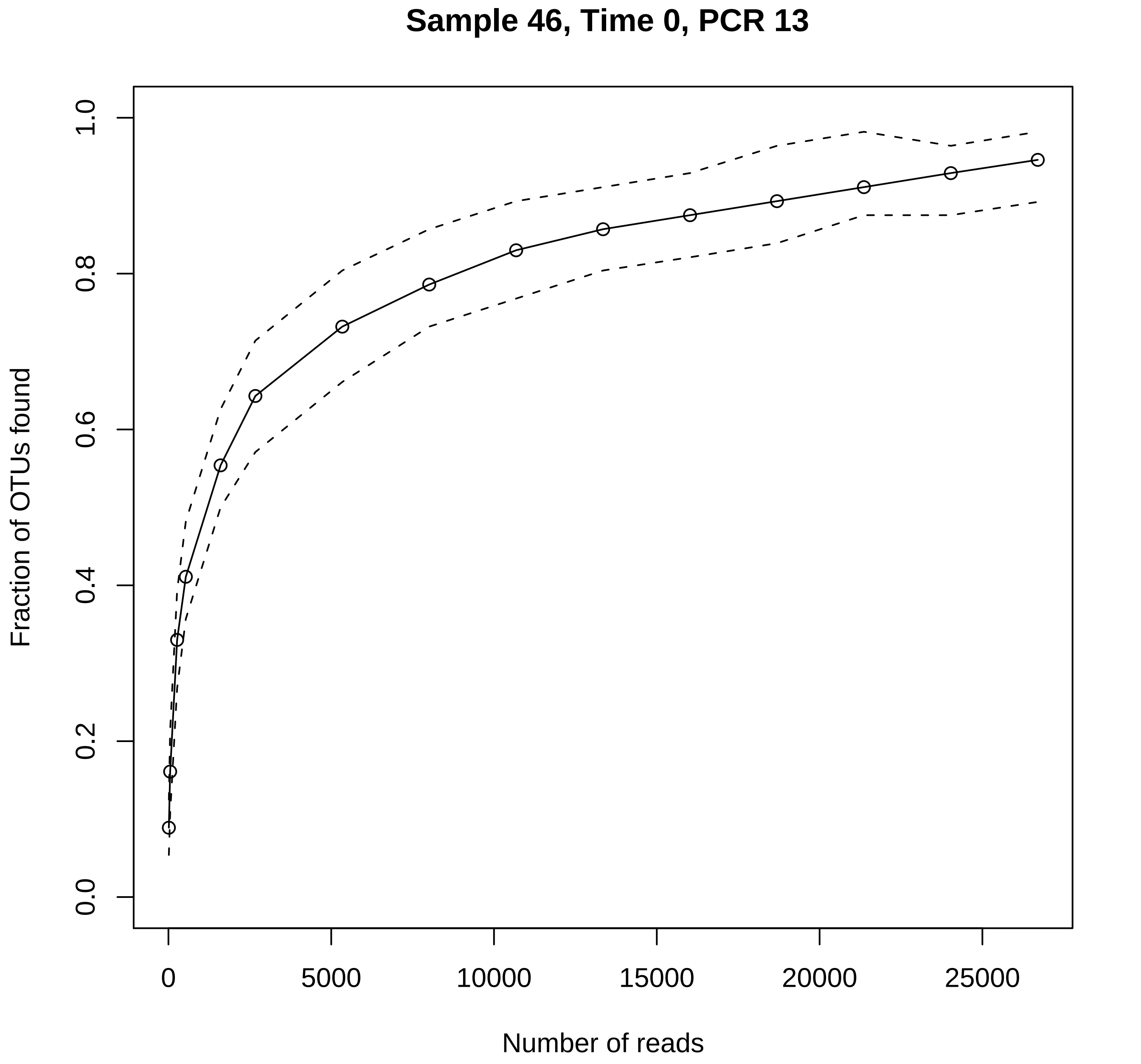
Sample 46, Time 0, PCR 13
Fraction of OTUs found
0.0
0.2
0.4
0.6
0.8
1.0
0
5000
10000
15000
20000
25000
Number of reads

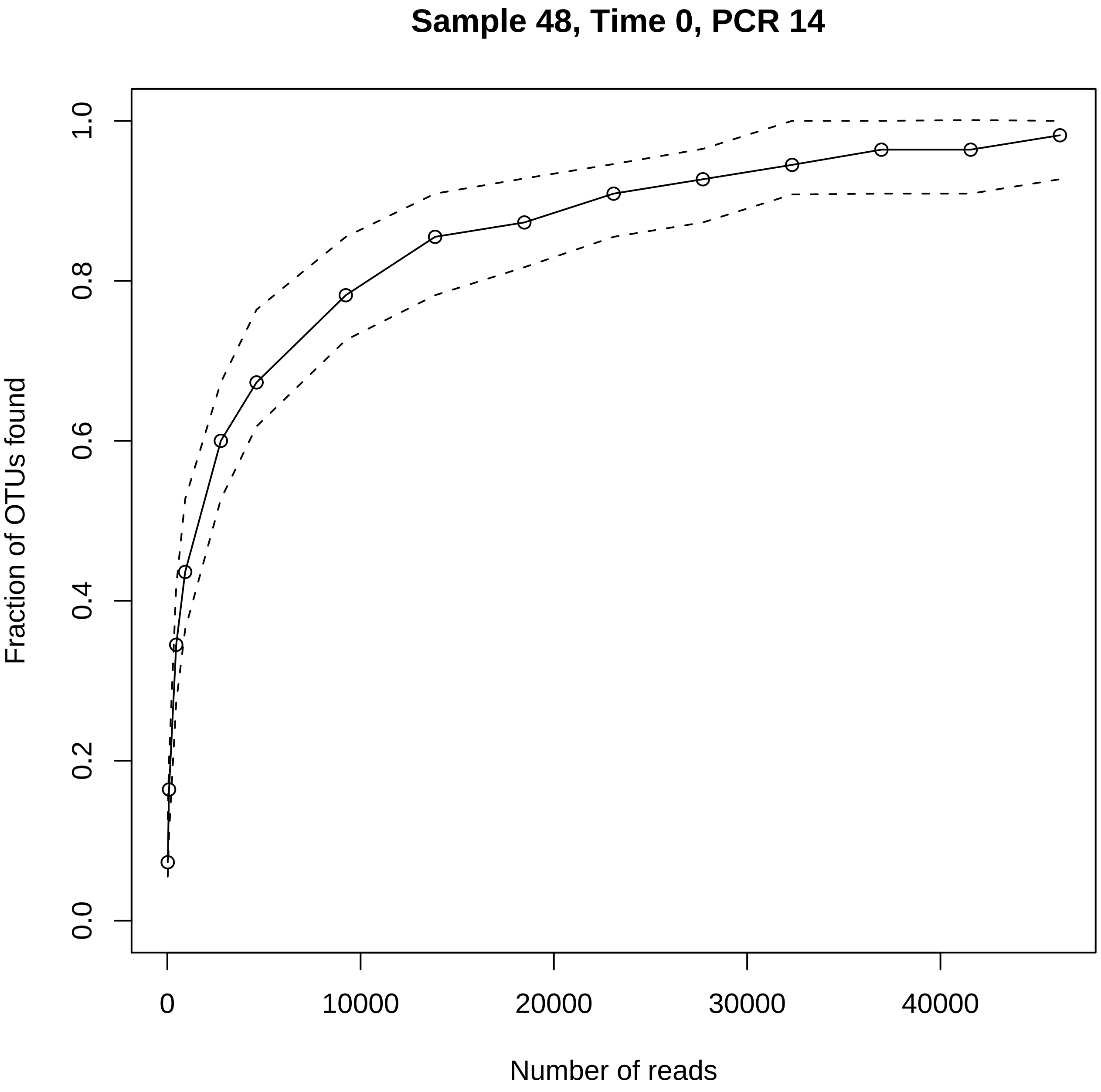

Sample 48, Time 0, PCR 14
Fraction of OTUs found
0.0
0.2
0.4
0.6
0.8
1.0
0
10000
20000
30000
40000
Number of reads

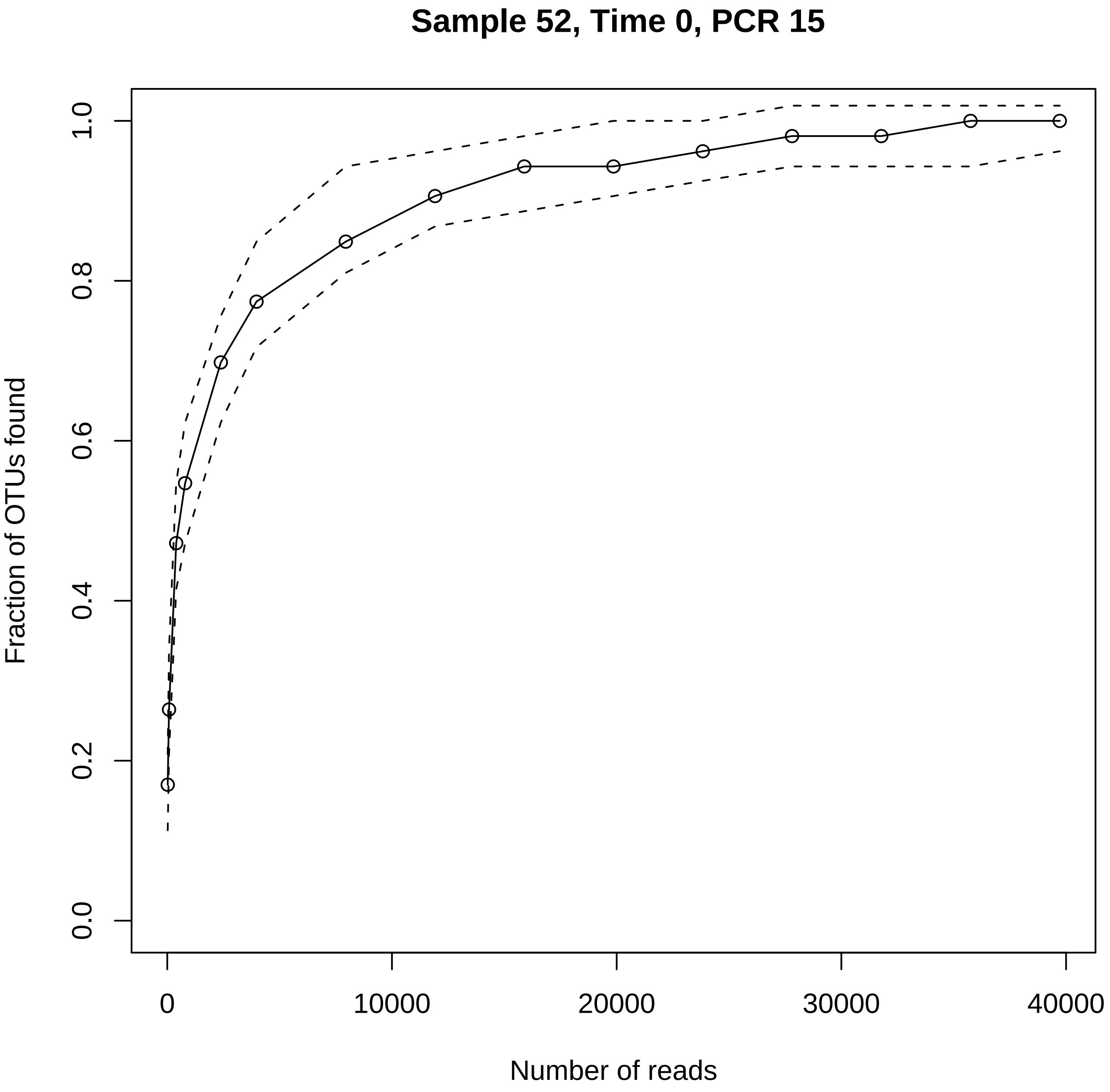

Sample 52, Time 0, PCR 15
Fraction of OTUs found
0.0
0.2
0.4
0.6
0.8
1.0
0
10000
20000
30000
40000
Number of reads

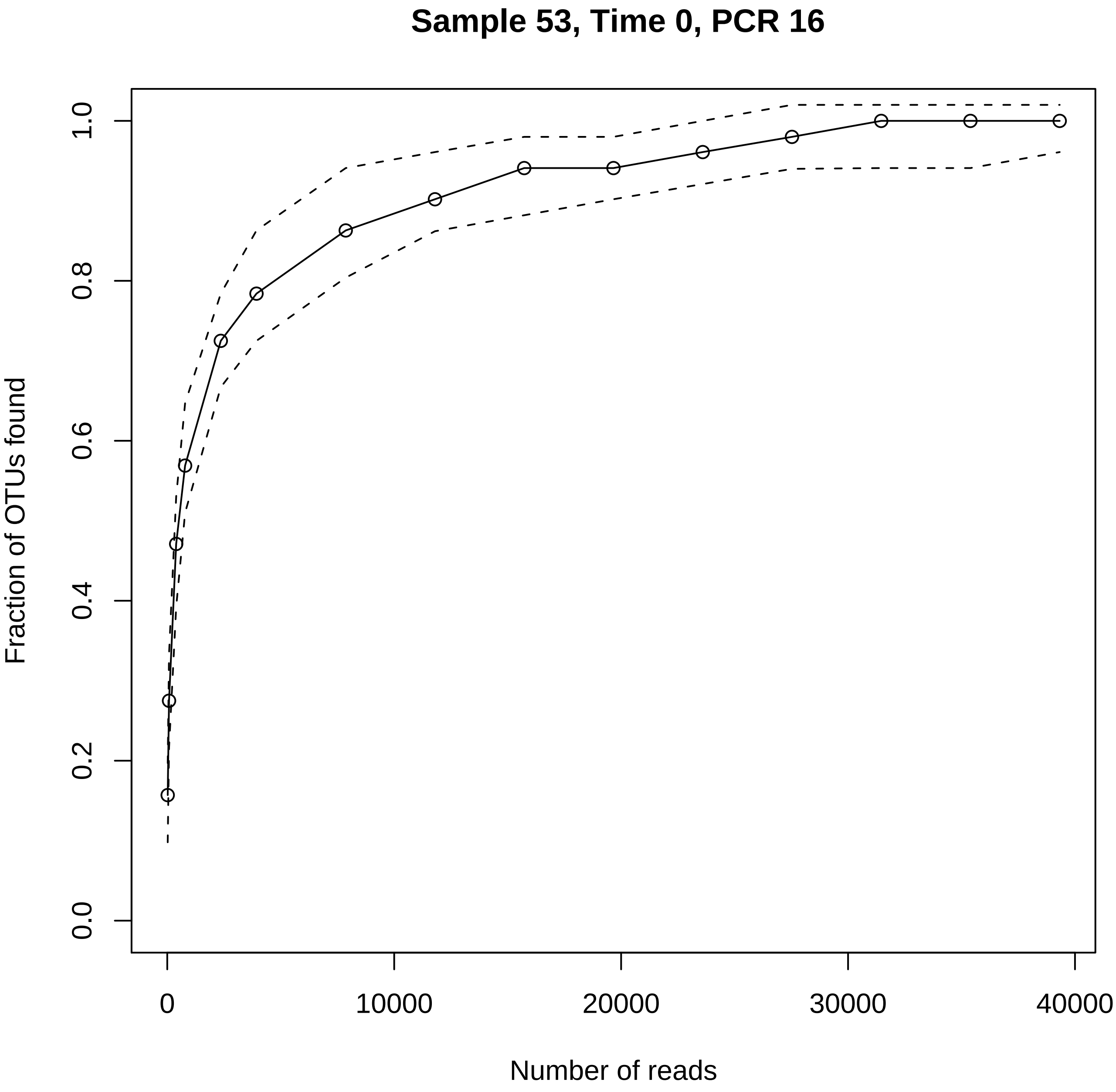

Sample 53, Time 0, PCR 16
Fraction of OTUs found
Number of reads
0.0
0.2
0.4
0.6
0.8
1.0
0
10000
20000
30000
40000

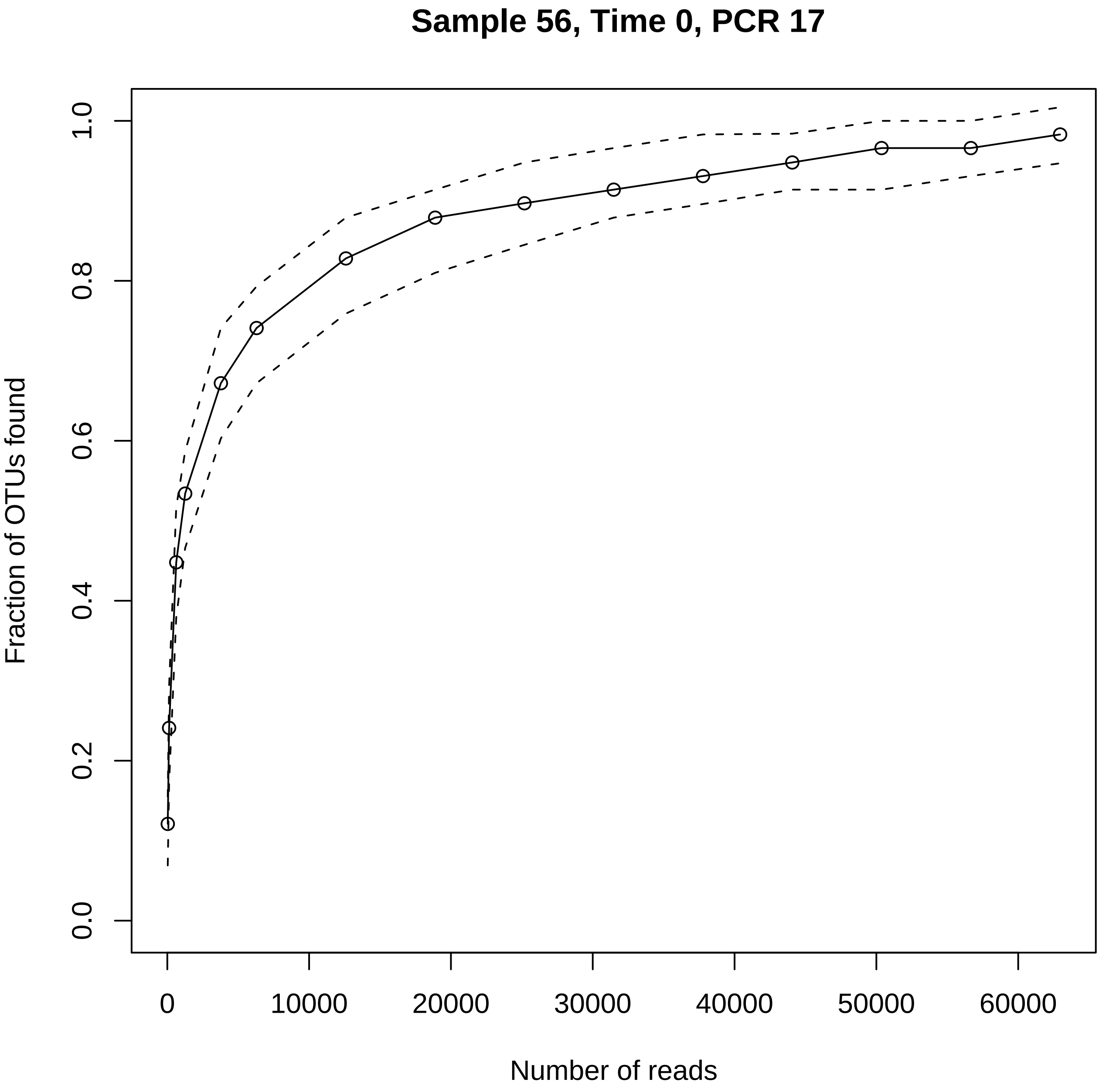

Sample 56, Time 0, PCR 17
Fraction of OTUs found
0.0
0.2
0.4
0.6
0.8
1.0
0
10000
20000
30000
40000
50000
60000
Number of reads

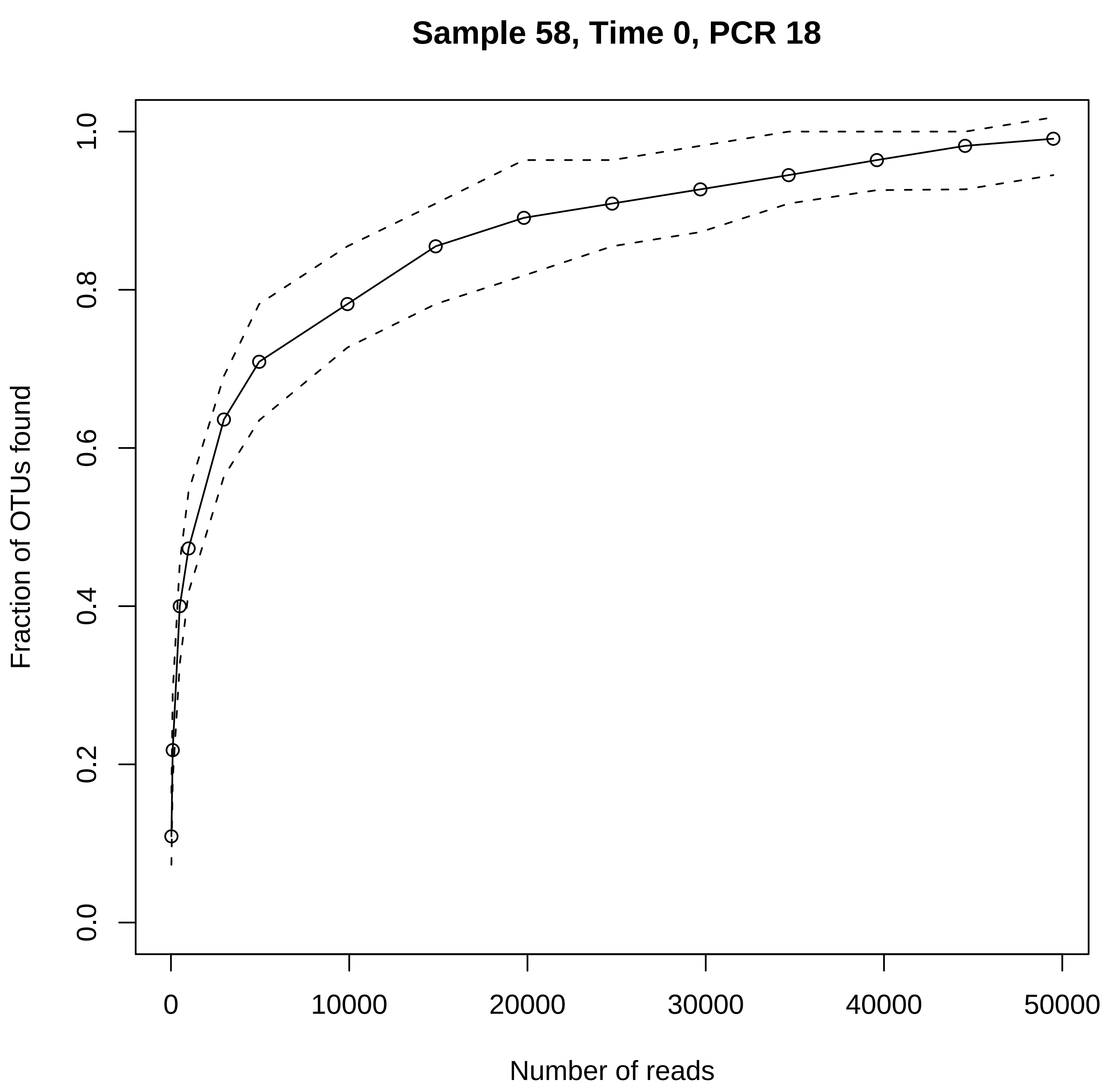
Sample 58, Time 0, PCR 18
Fraction of OTUs found
0.0
0.2
0.4
0.6
0.8
1.0
0
10000
20000
30000
40000
50000
Number of reads

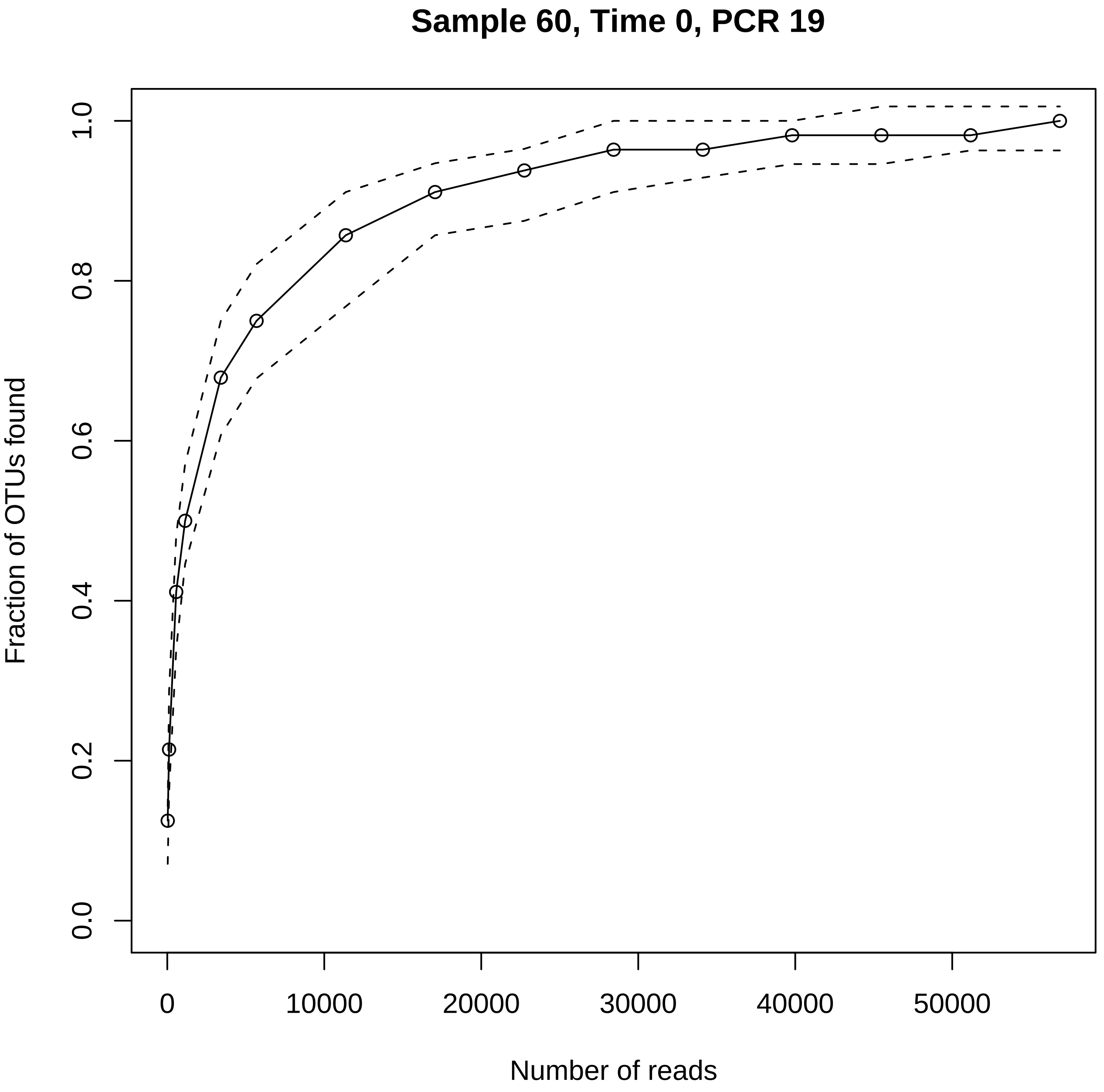
Sample 60, Time 0, PCR 19
Fraction of OTUs found
0.0
0.2
0.4
0.6
0.8
1.0
0
10000
20000
30000
40000
50000
Number of reads

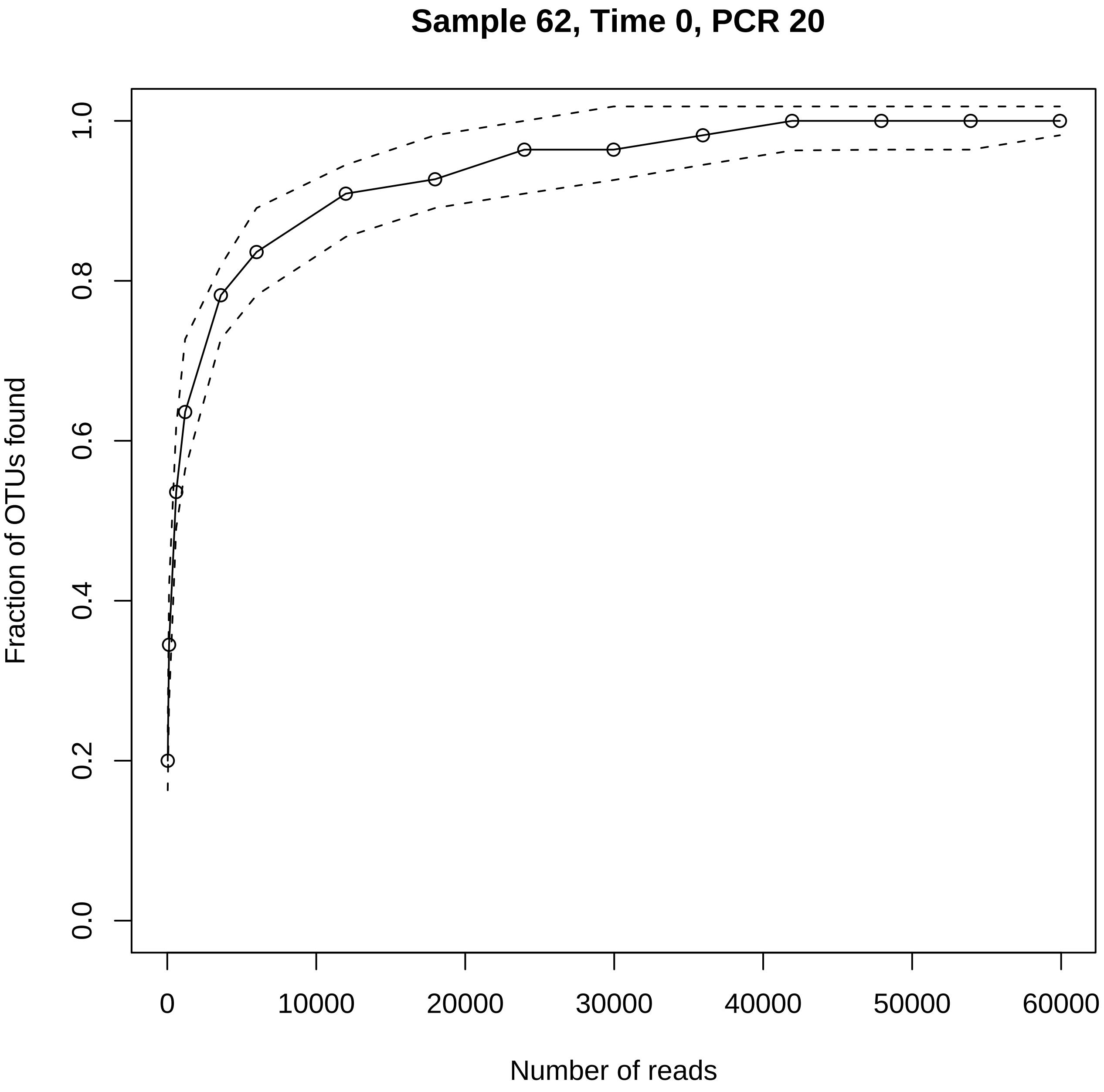
Sample 62, Time 0, PCR 20
Fraction of OTUs found
0.0
0.2
0.4
0.6
0.8
1.0
0
10000
20000
30000
40000
50000
60000
Number of reads

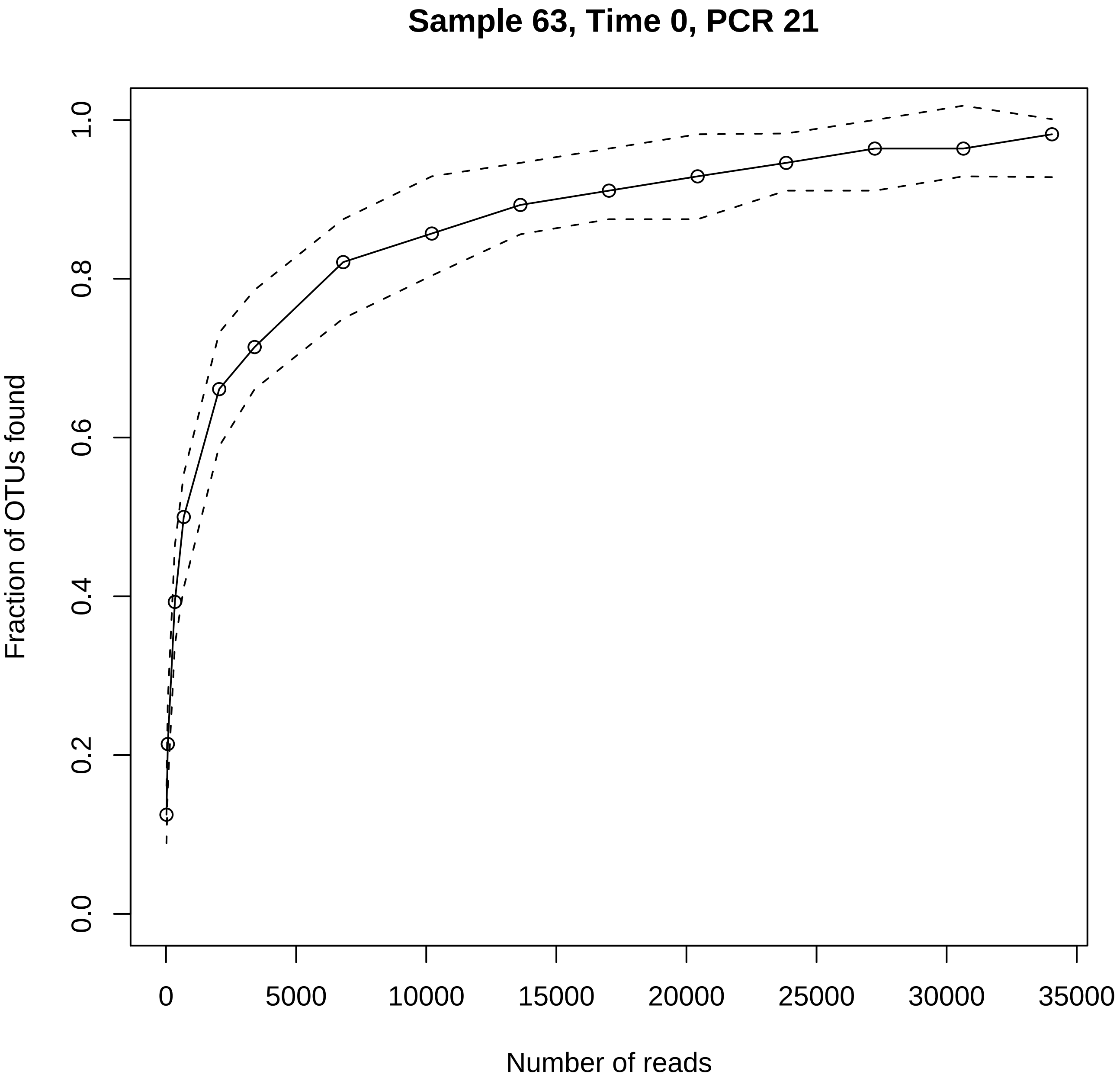

Sample 63, Time 0, PCR 21
Fraction of OTUs found
0.0
0.2
0.4
0.6
0.8
1.0
0
5000
10000
15000
20000
25000
30000
35000
Number of reads

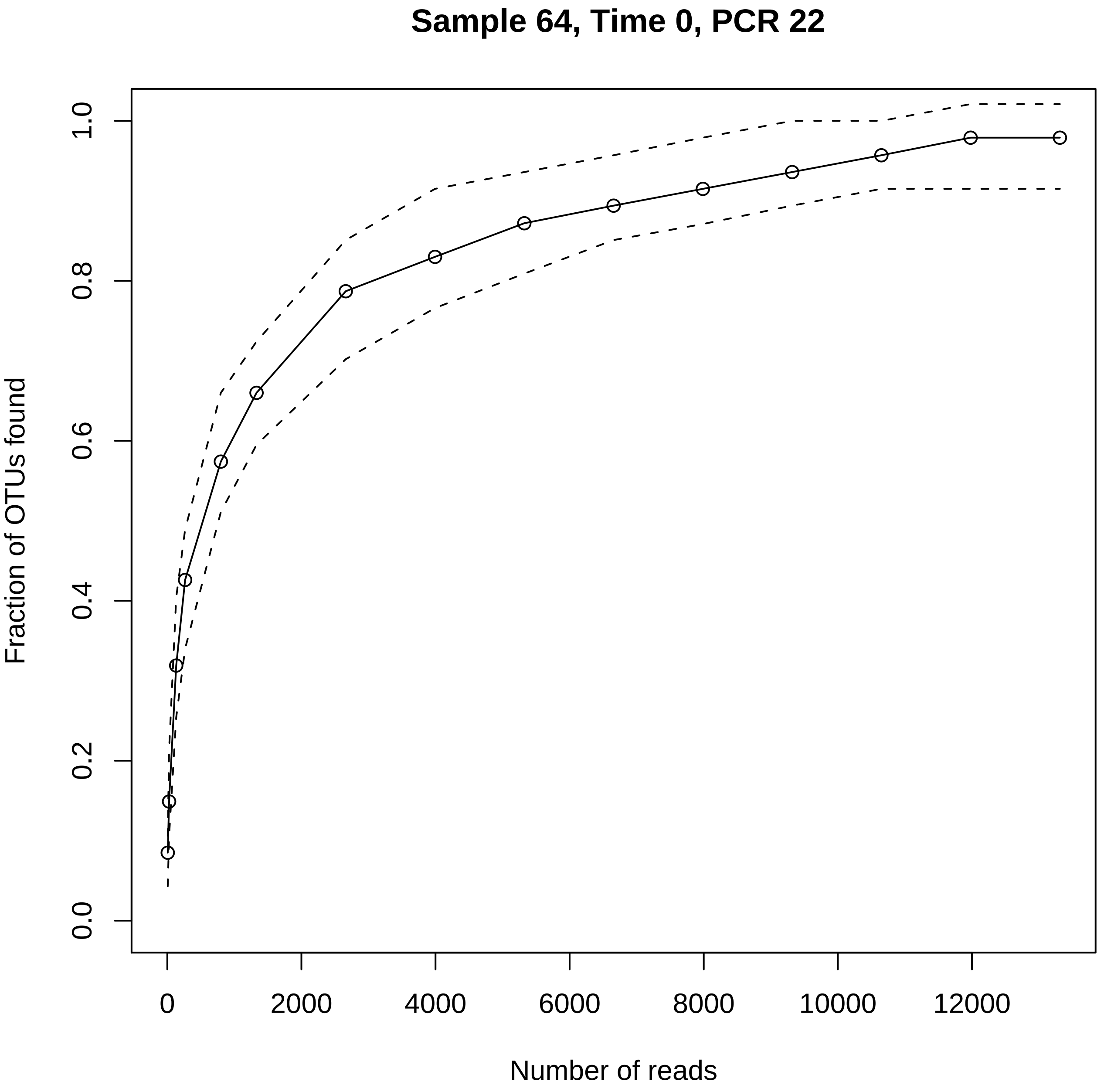
Sample 64, Time 0, PCR 22
Fraction of OTUs found
0.0
0.2
0.4
0.6
0.8
1.0
0
2000
4000
6000
8000
10000
12000
Number of reads

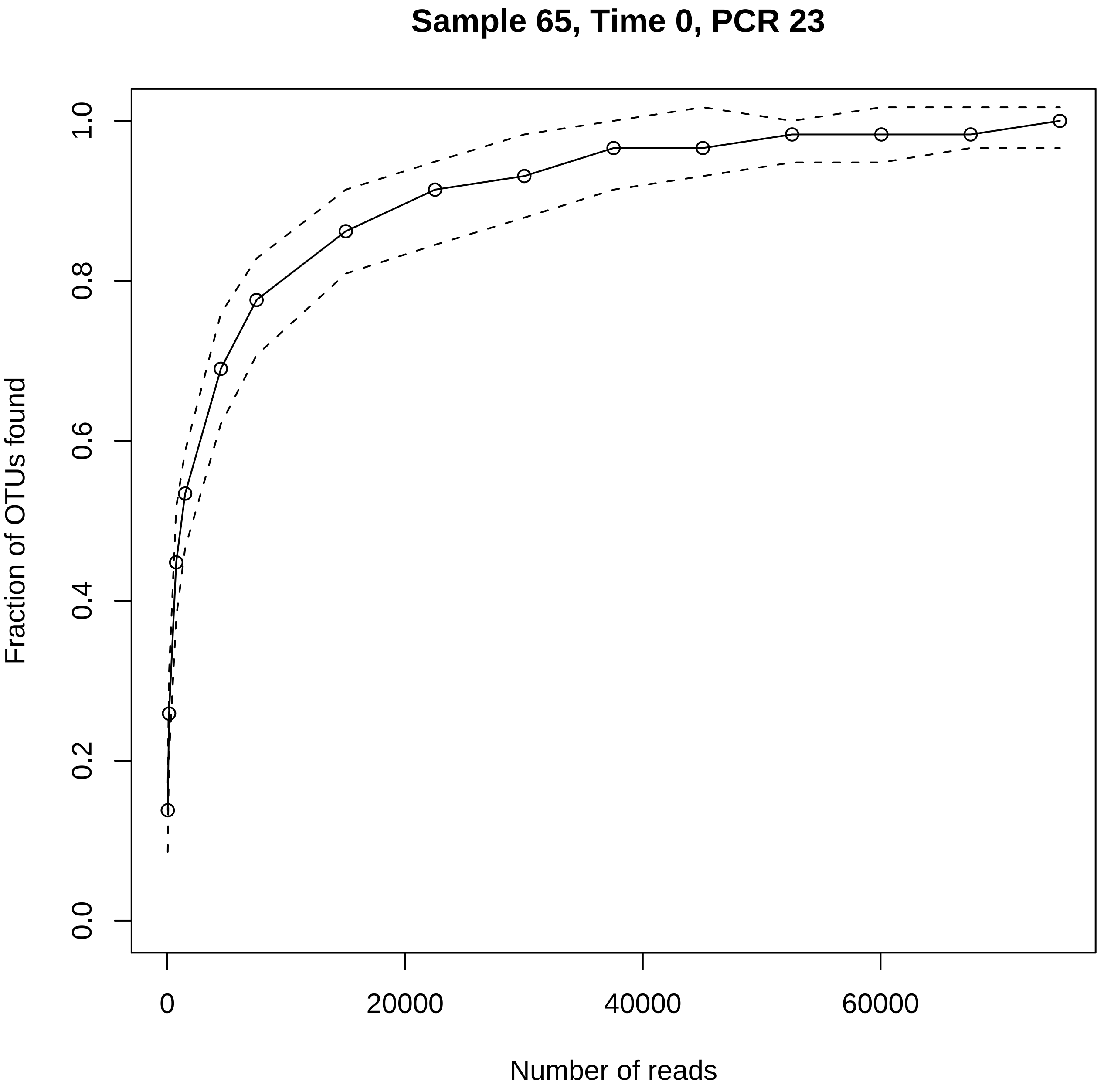

Sample 65, Time 0, PCR 23
Fraction of OTUs found
0.0
0.2
0.4
0.6
0.8
1.0
0
20000
40000
60000
Number of reads

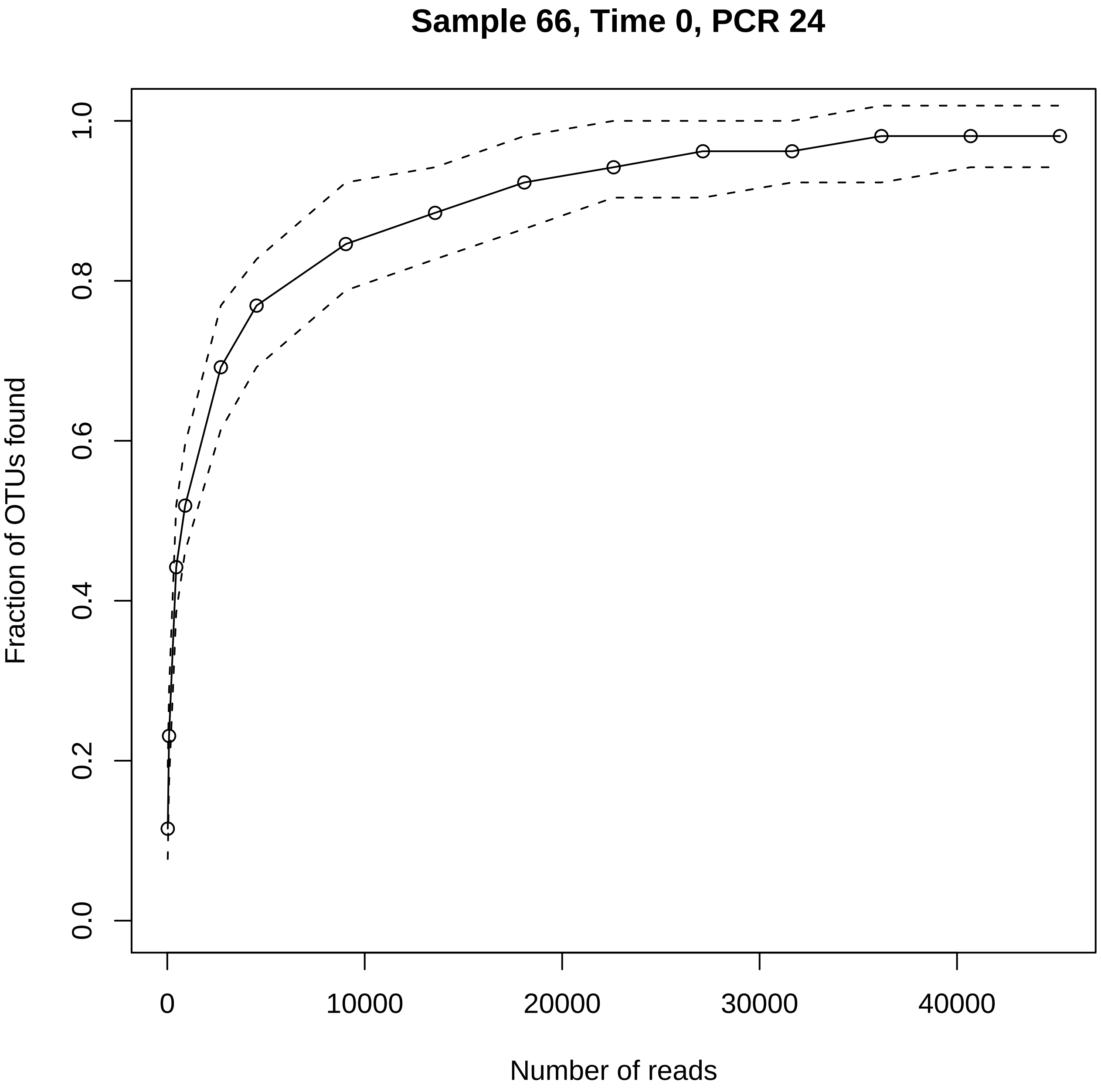
Sample 66, Time 0, PCR 24
Fraction of OTUs found
0.0
0.2
0.4
0.6
0.8
1.0
0
10000
20000
30000
40000
Number of reads

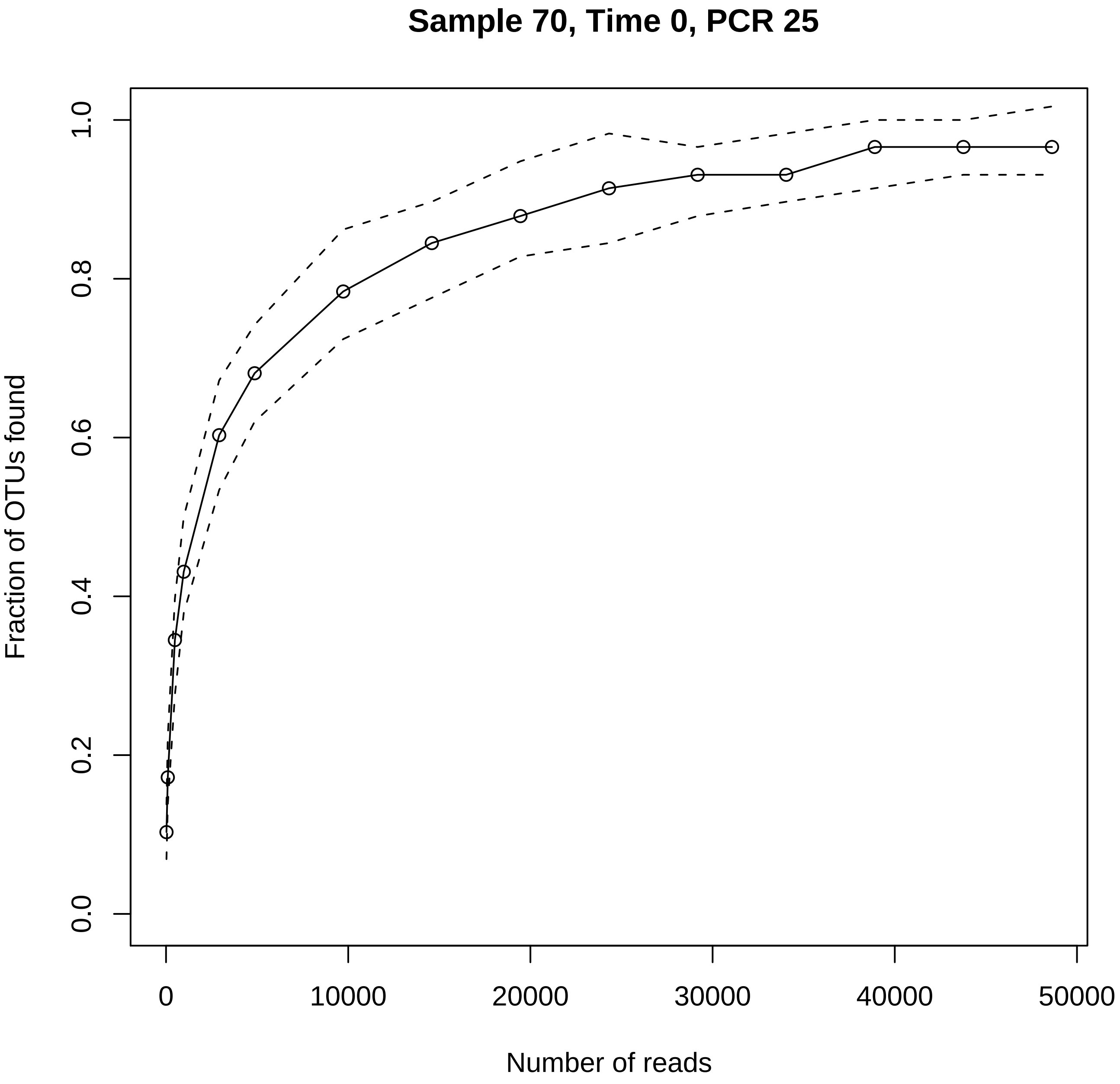
Sample 70, Time 0, PCR 25
Fraction of OTUs found
0.0
0.2
0.4
0.6
0.8
1.0
0
10000
20000
30000
40000
50000
Number of reads

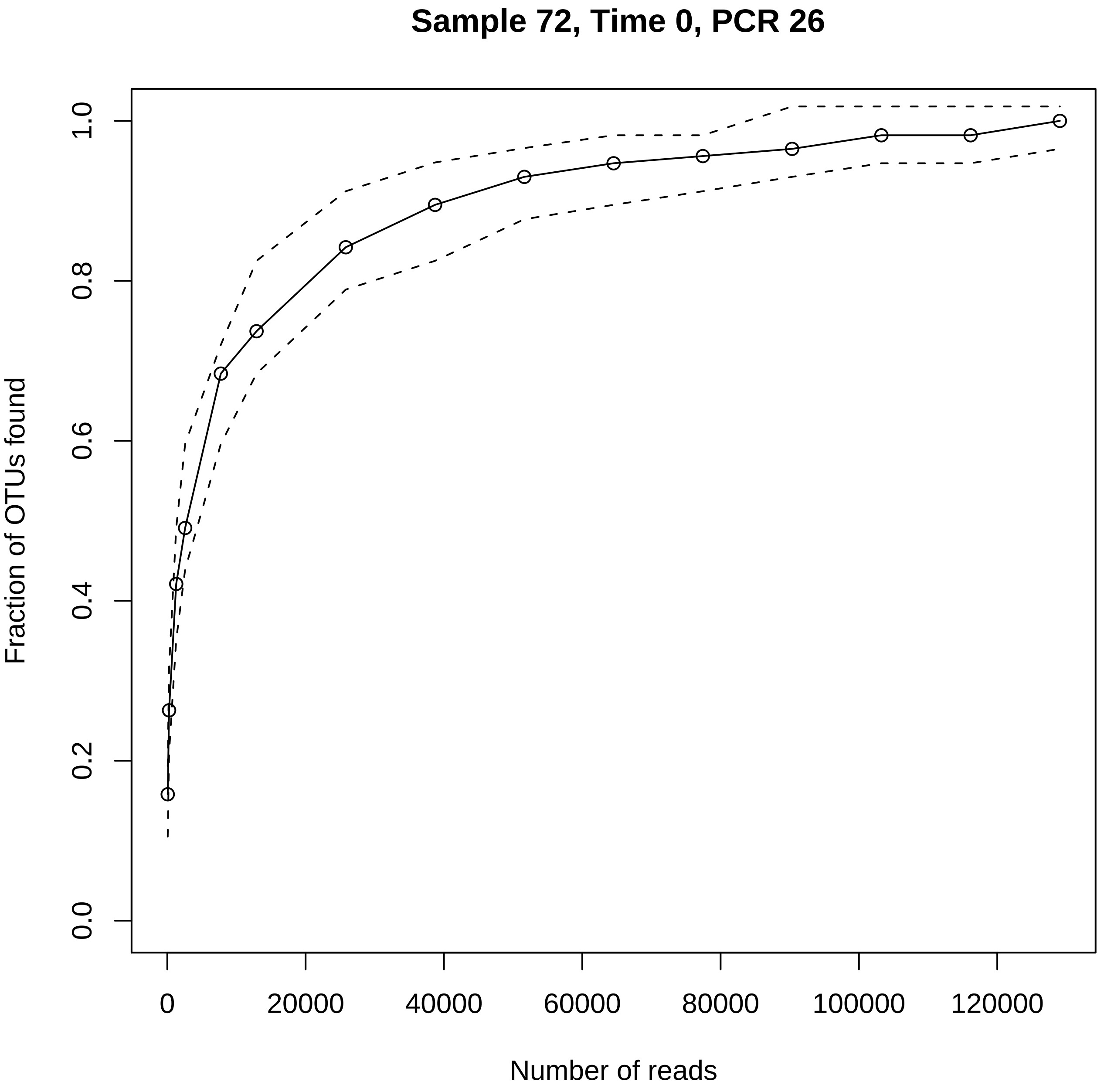
Sample 72, Time 0, PCR 26
Fraction of OTUs found
0.0
0.2
0.4
0.6
0.8
1.0
0
20000
40000
60000
80000
100000
120000
Number of reads

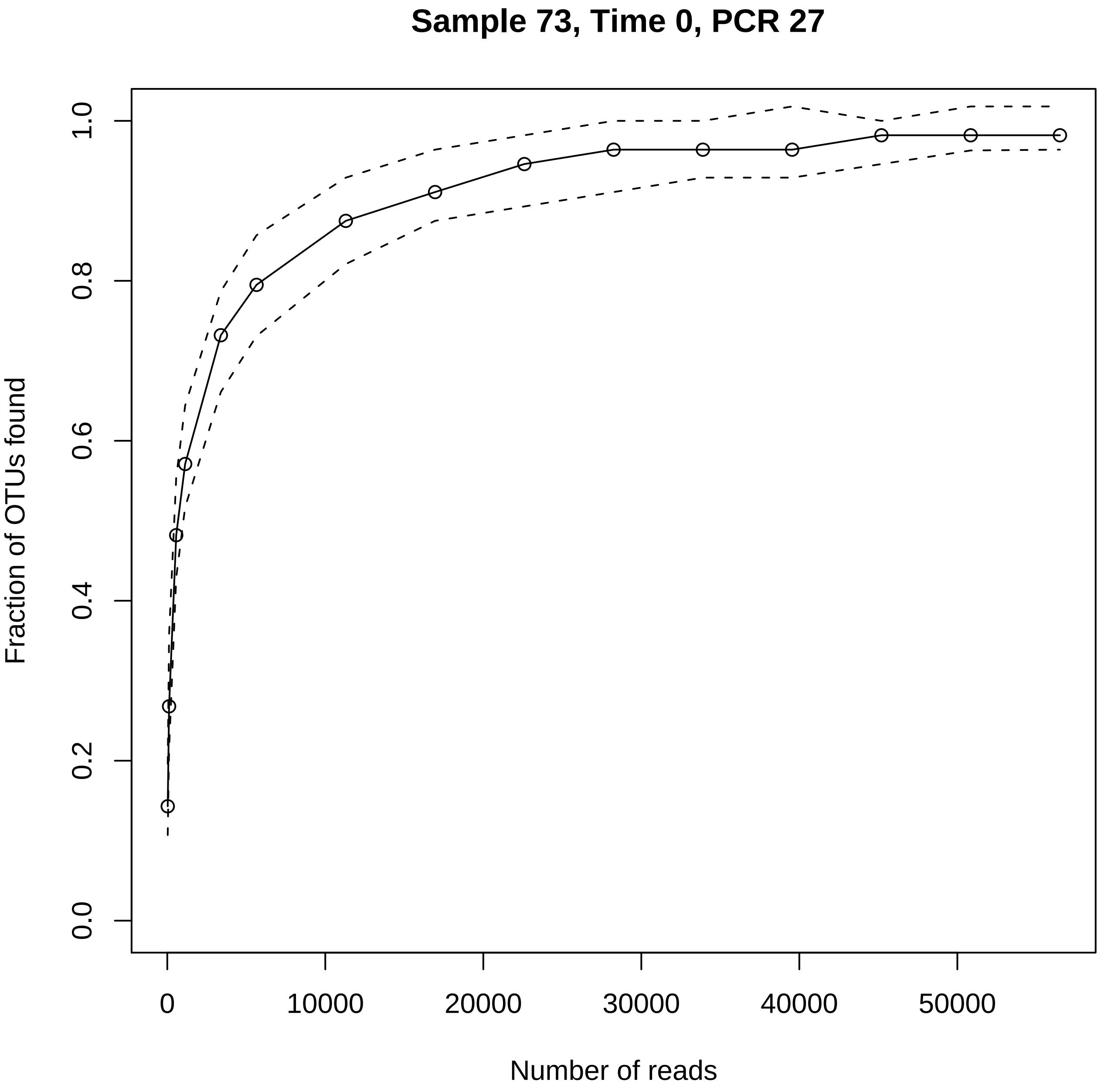

Sample 73, Time 0, PCR 27
Fraction of OTUs found
0.0
0.2
0.4
0.6
0.8
1.0
0
10000
20000
30000
40000
50000
Number of reads

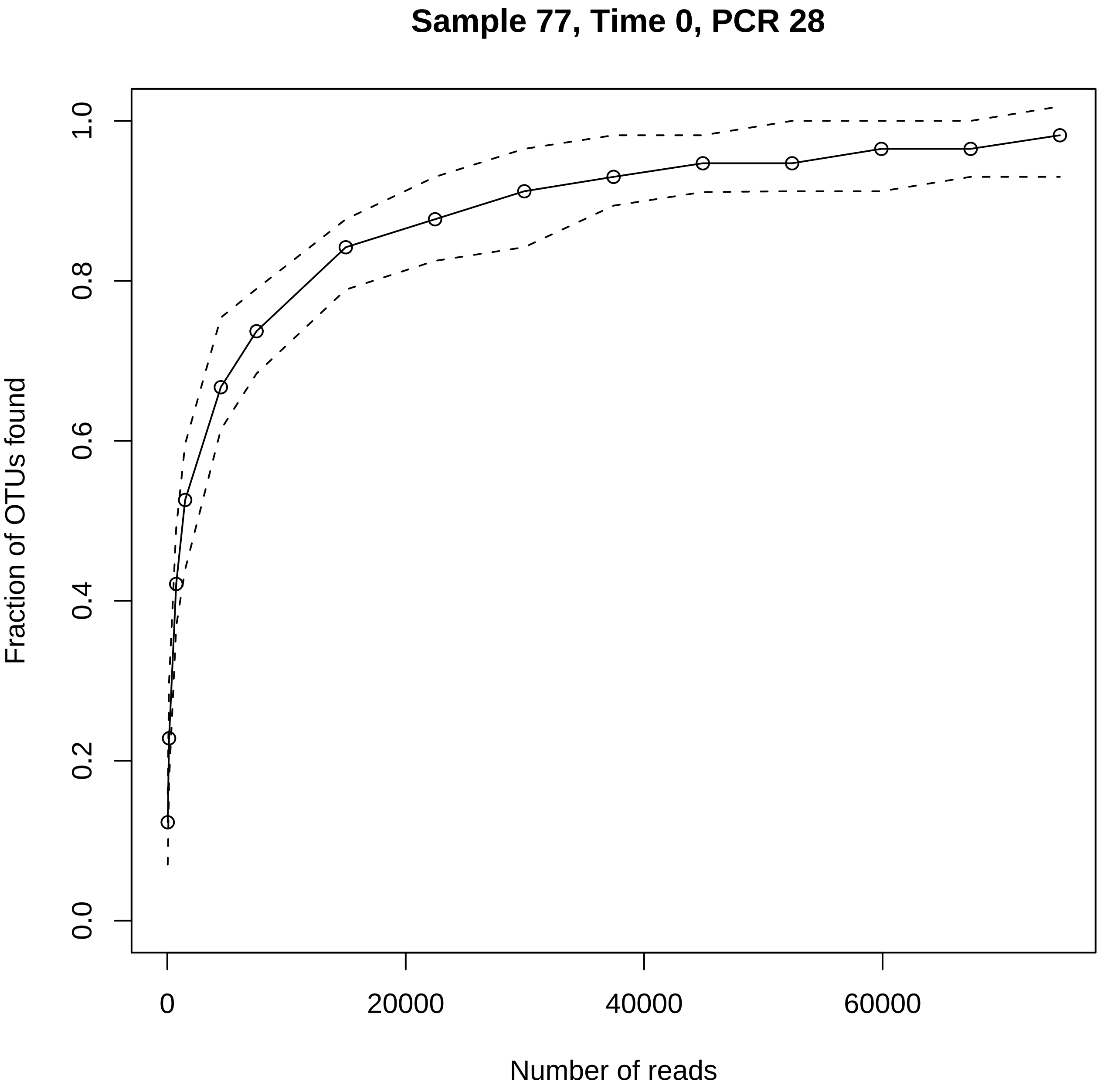

Sample 77, Time 0, PCR 28
Fraction of OTUs found
0.0
0.2
0.4
0.6
0.8
1.0
0
20000
40000
60000
Number of reads

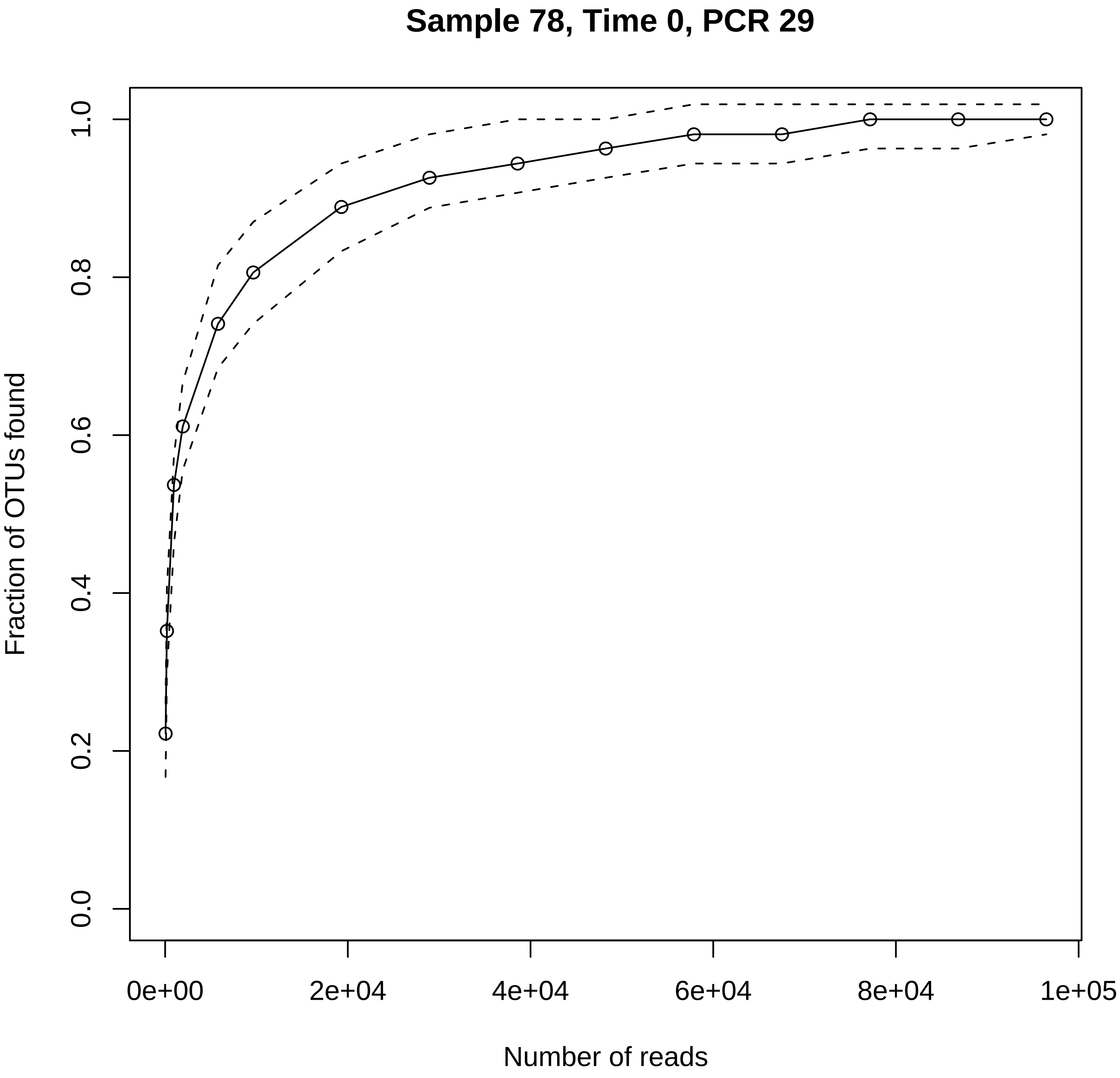

Sample 78, Time 0, PCR 29
Fraction of OTUs found
Number of reads
0.0
0.2
0.4
0.6
0.8
1.0
0e+00
2e+04
4e+04
6e+04
8e+04
1e+05

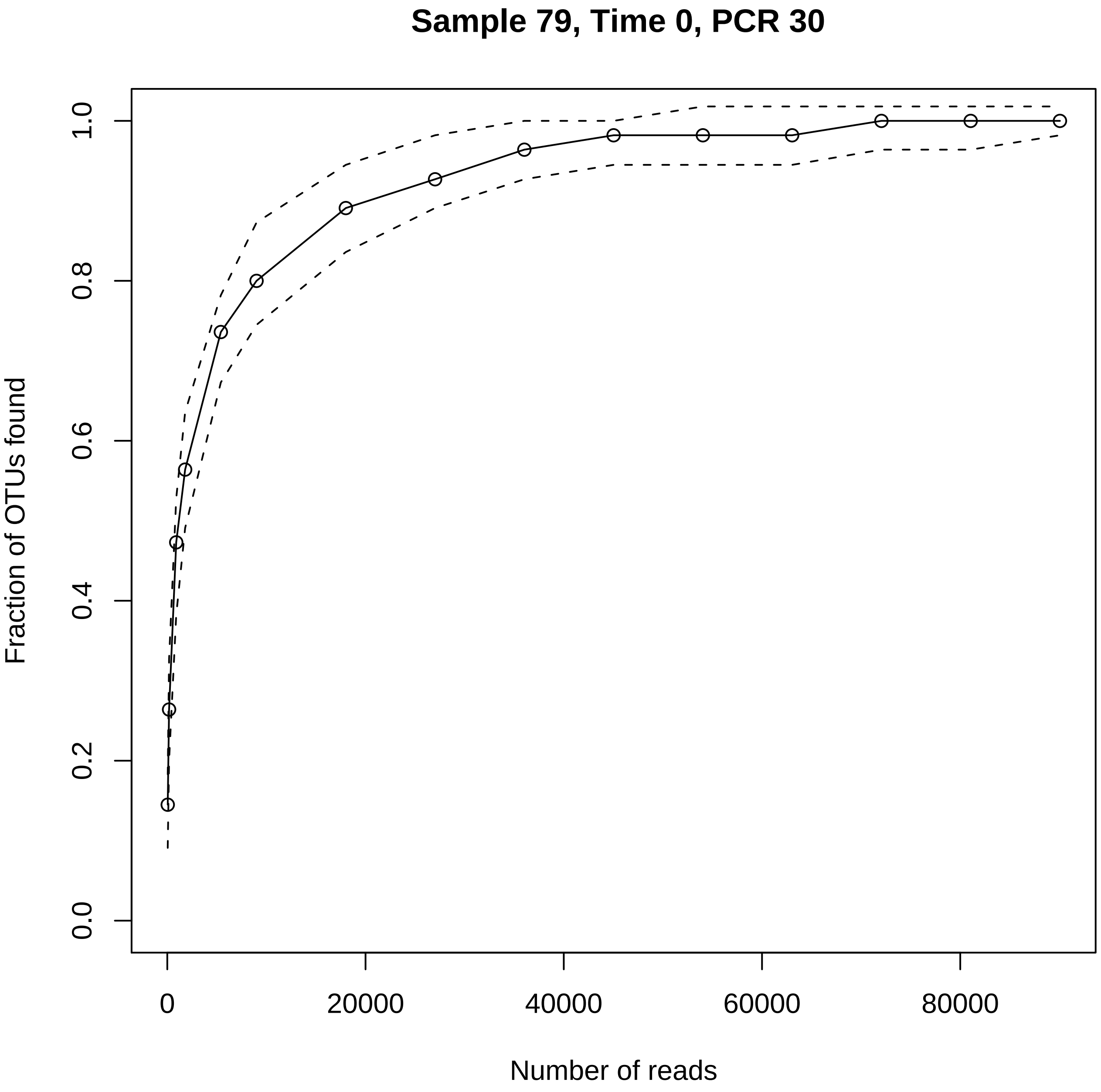
Sample 79, Time 0, PCR 30
Fraction of OTUs found
0.0
0.2
0.4
0.6
0.8
1.0
0
20000
40000
60000
80000
Number of reads

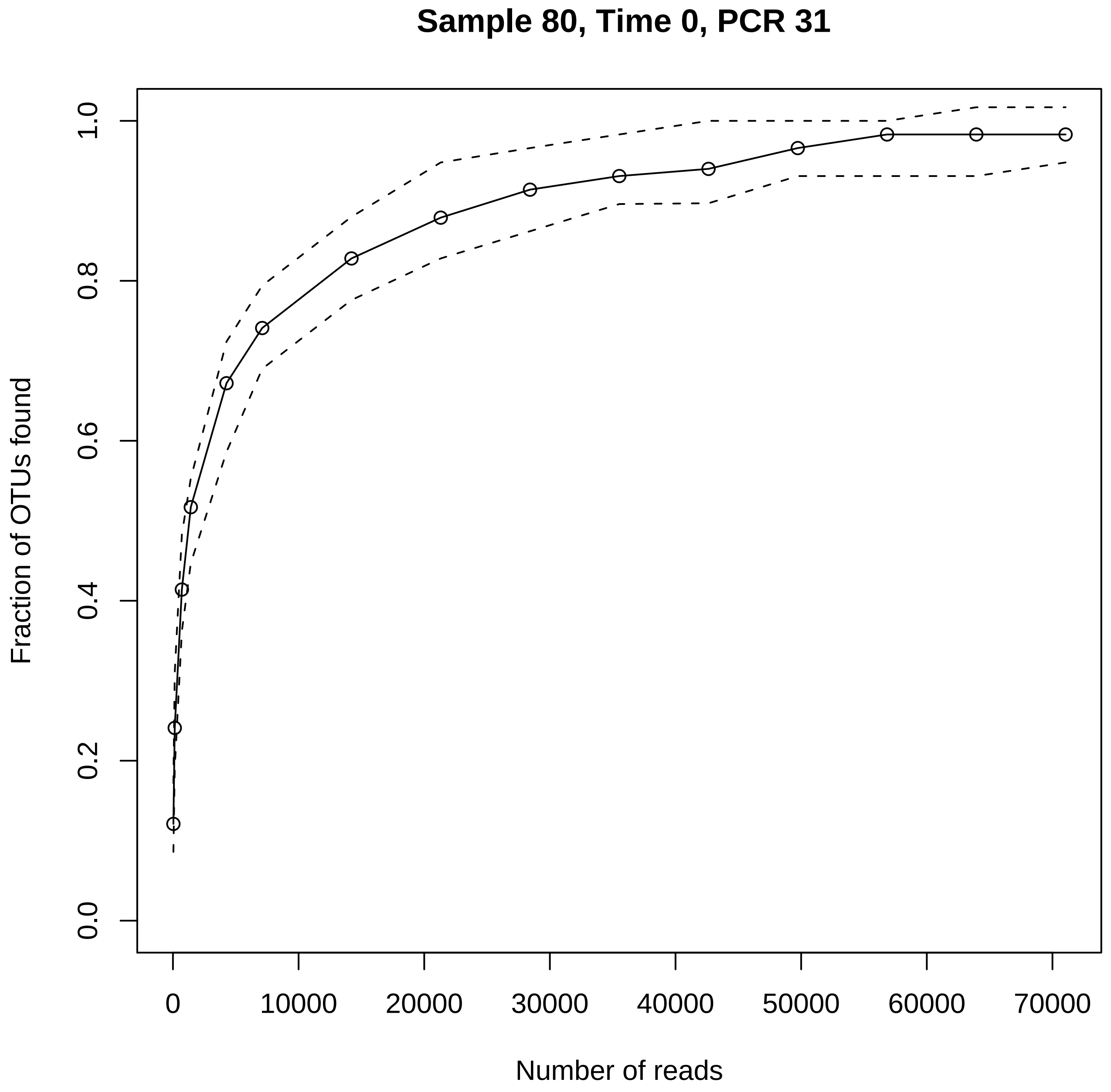
Sample 80, Time 0, PCR 31
Fraction of OTUs found
0.0
0.2
0.4
0.6
0.8
1.0
0
10000
20000
30000
40000
50000
60000
70000
Number of reads

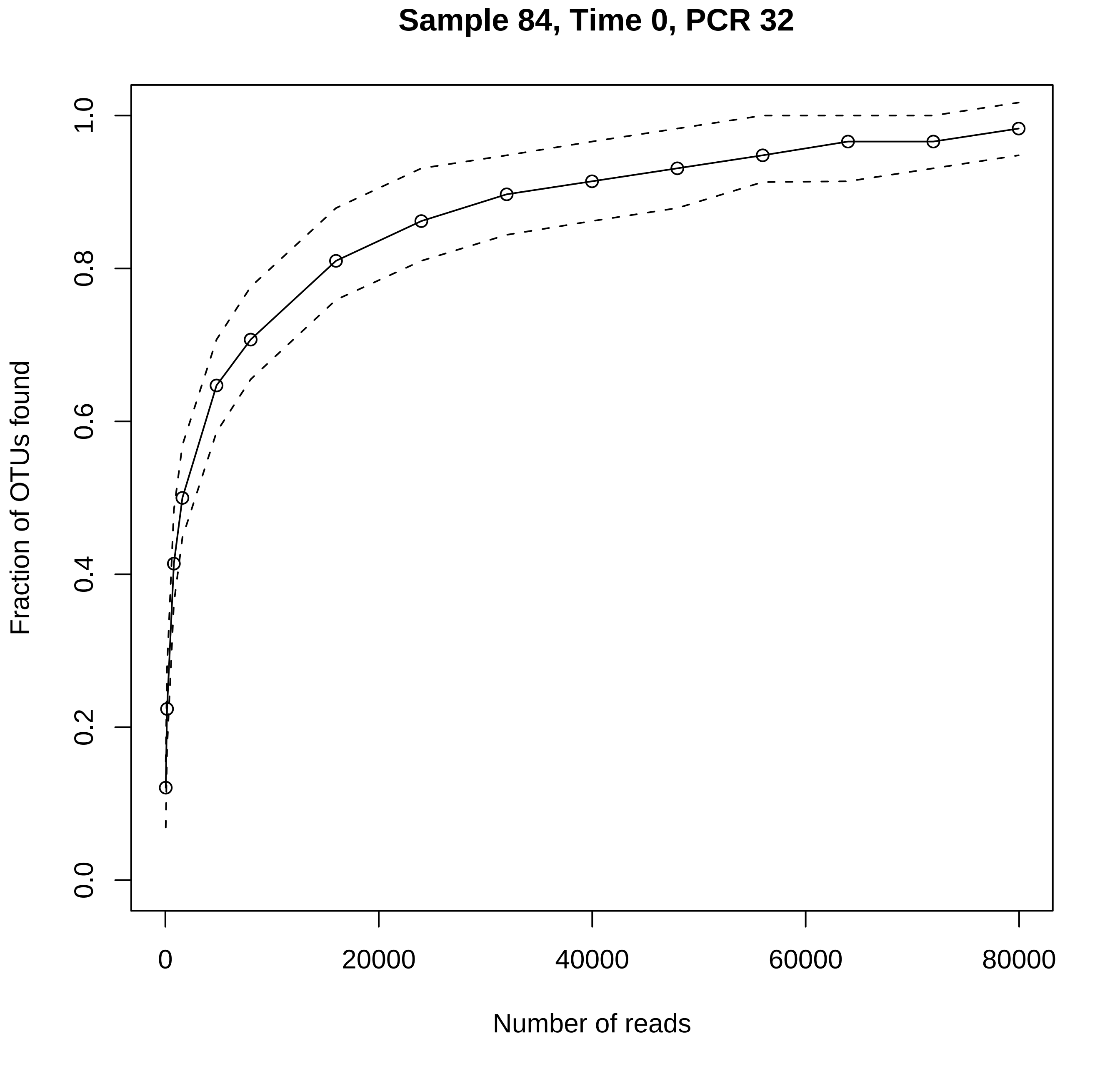
Sample 84, Time 0, PCR 32
Fraction of OTUs found
0.0
0.2
0.4
0.6
0.8
1.0
0
20000
40000
60000
80000
Number of reads

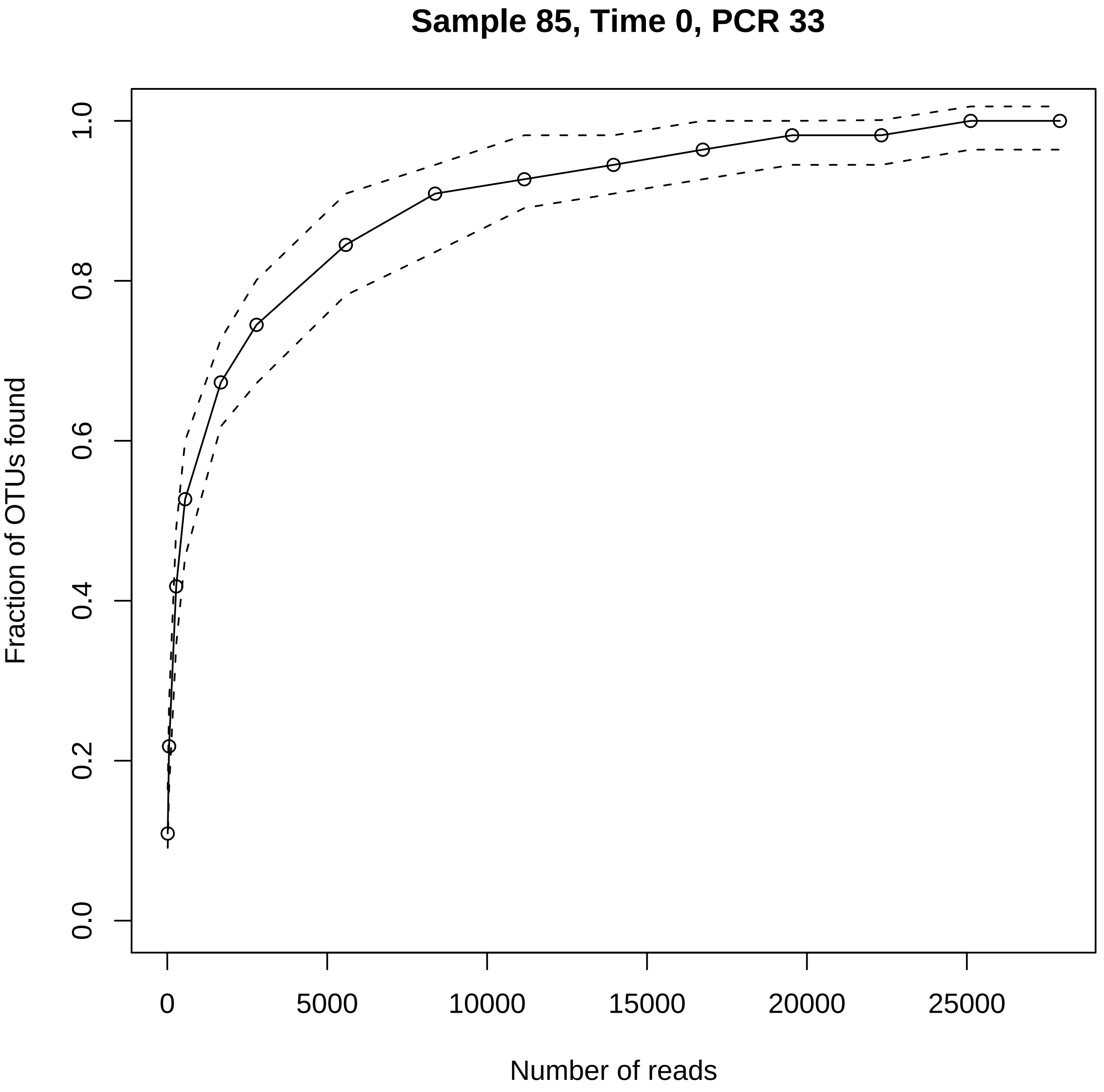
Sample 85, Time 0, PCR 33
Fraction of OTUs found
Number of reads
0.0
0.2
0.4
0.6
0.8
1.0
0
5000
10000
15000
20000
25000

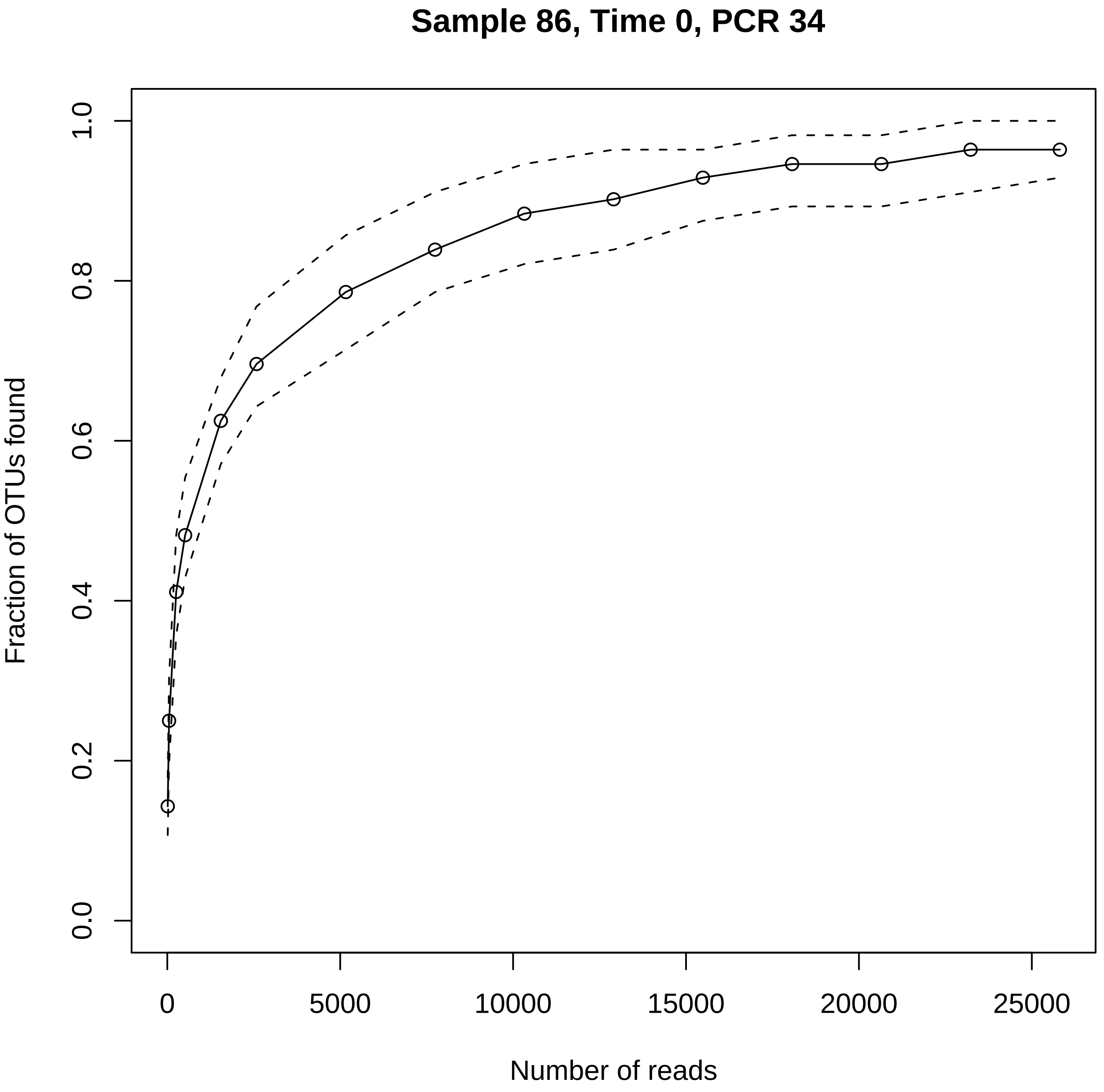

Sample 86, Time 0, PCR 34
Fraction of OTUs found
0.0
0.2
0.4
0.6
0.8
1.0
0
5000
10000
15000
20000
25000
Number of reads

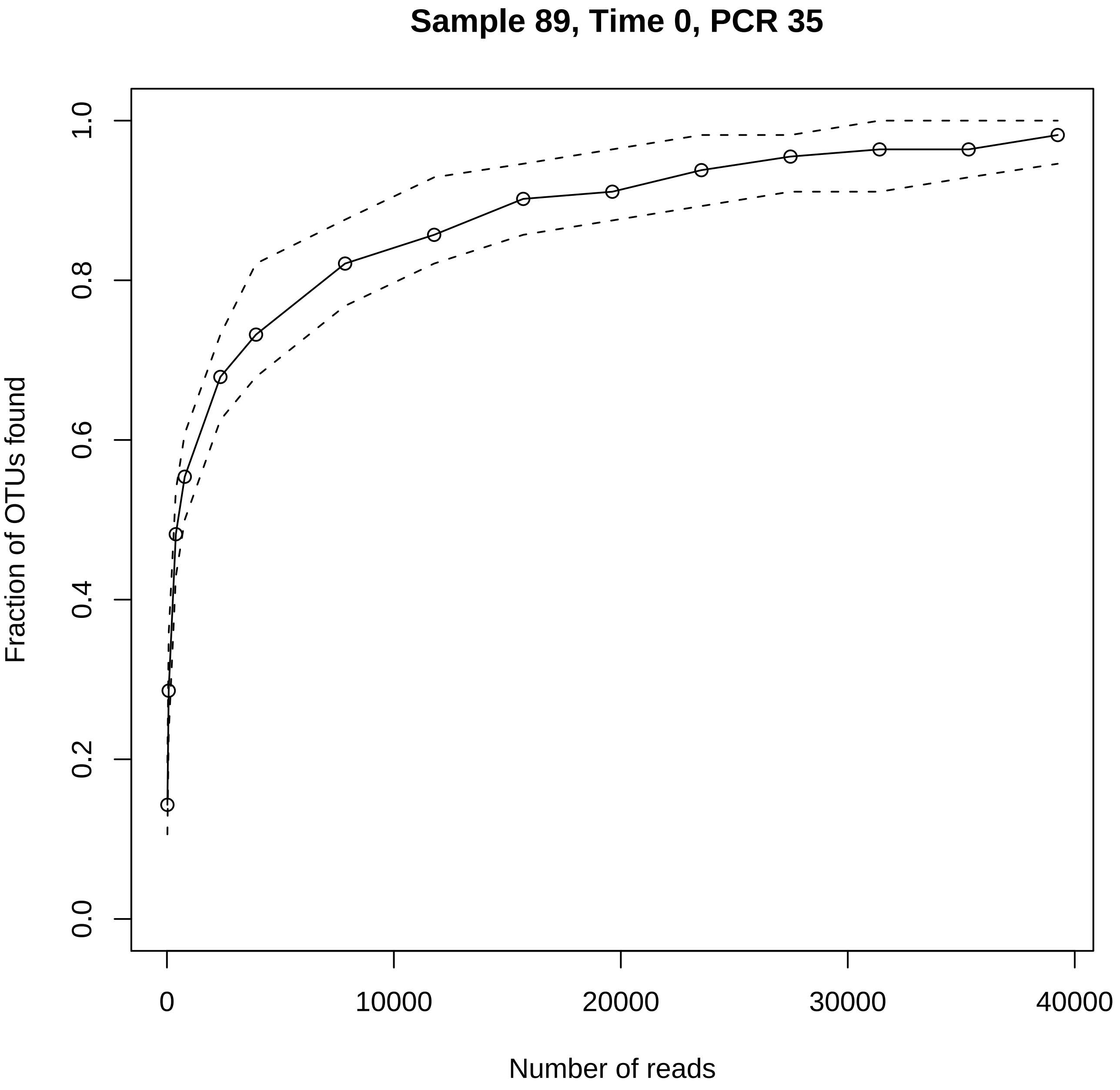

Sample 89, Time 0, PCR 35
Fraction of OTUs found
0.0
0.2
0.4
0.6
0.8
1.0
0
10000
20000
30000
40000
Number of reads

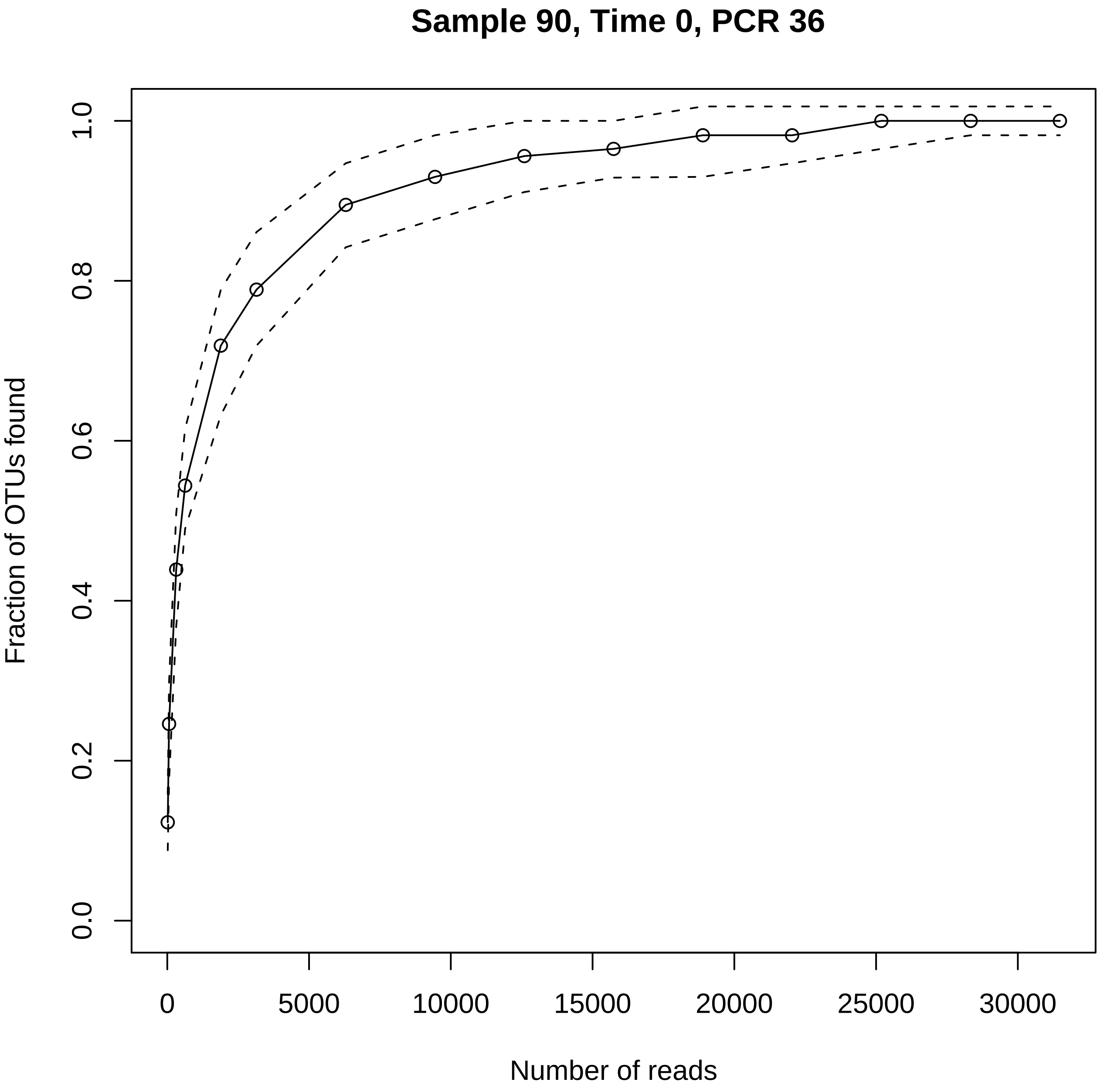
Sample 90, Time 0, PCR 36
Fraction of OTUs found
0.0
0.2
0.4
0.6
0.8
1.0
0
5000
10000
15000
20000
25000
30000
Number of reads

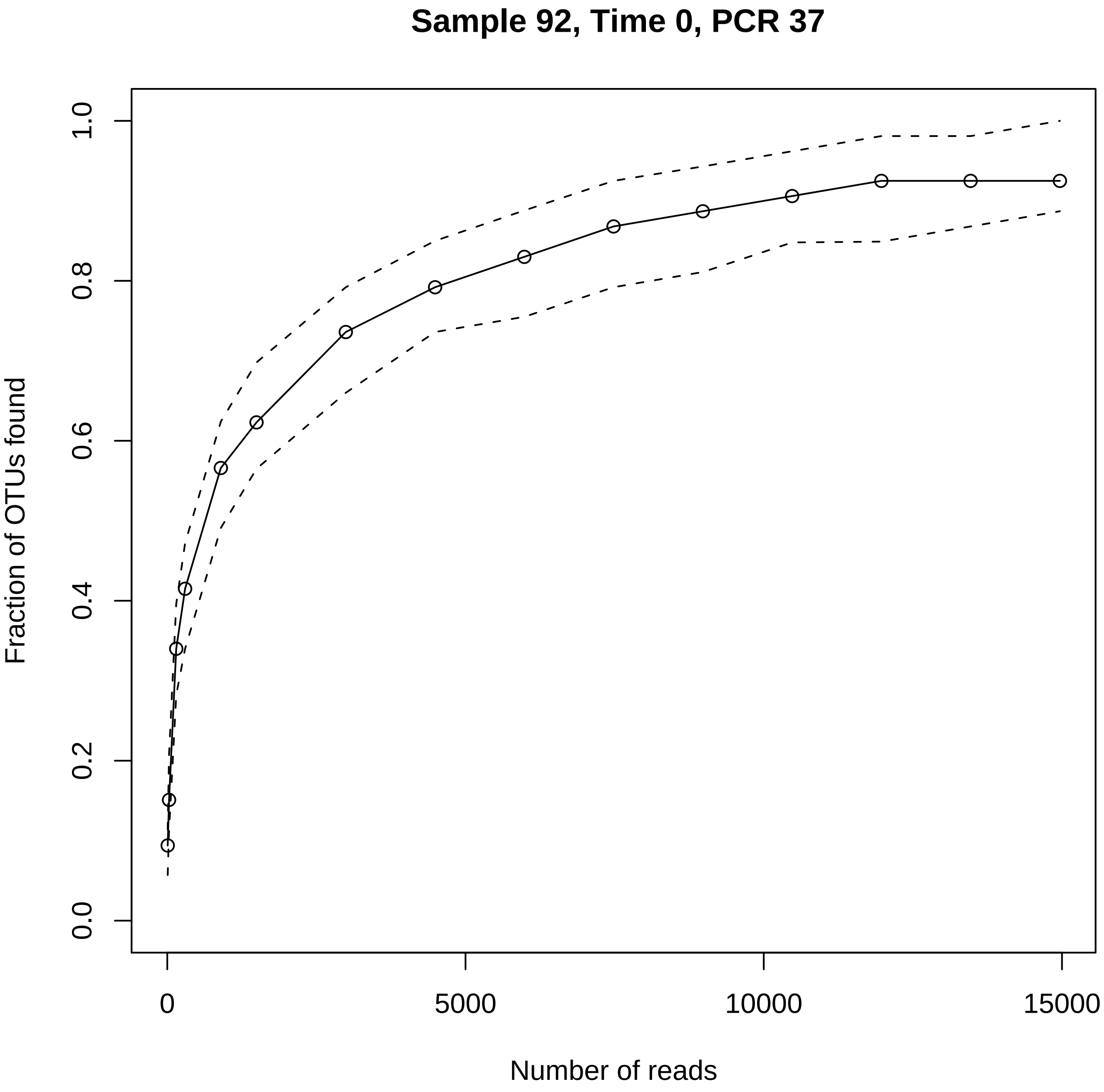

Sample 92, Time 0, PCR 37
Fraction of OTUs found
0.0
0.2
0.4
0.6
0.8
1.0
0
5000
10000
15000
Number of reads

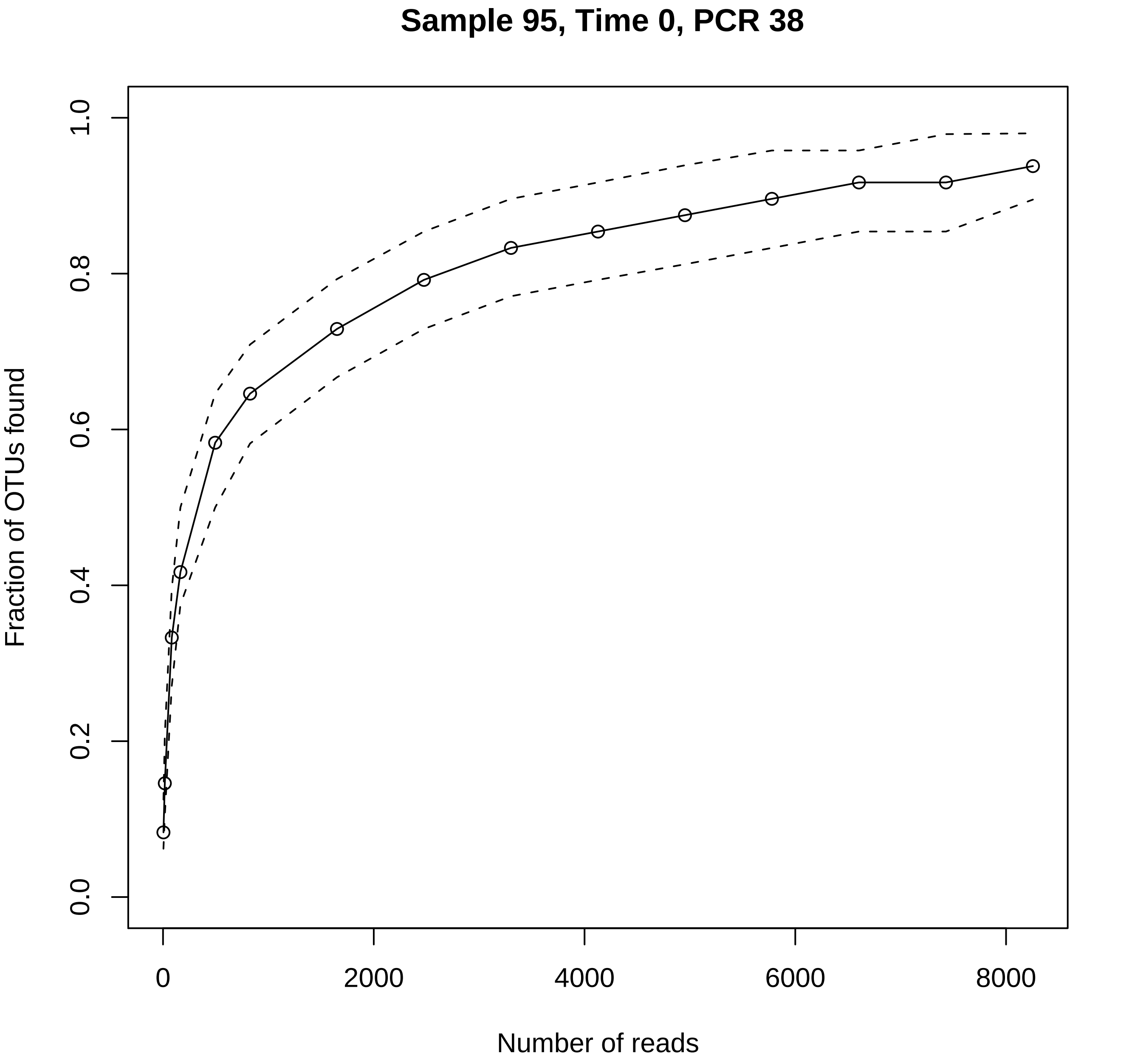
Sample 95, Time 0, PCR 38
Fraction of OTUs found
0.0
0.2
0.4
0.6
0.8
1.0
0
2000
4000
6000
8000
Number of reads

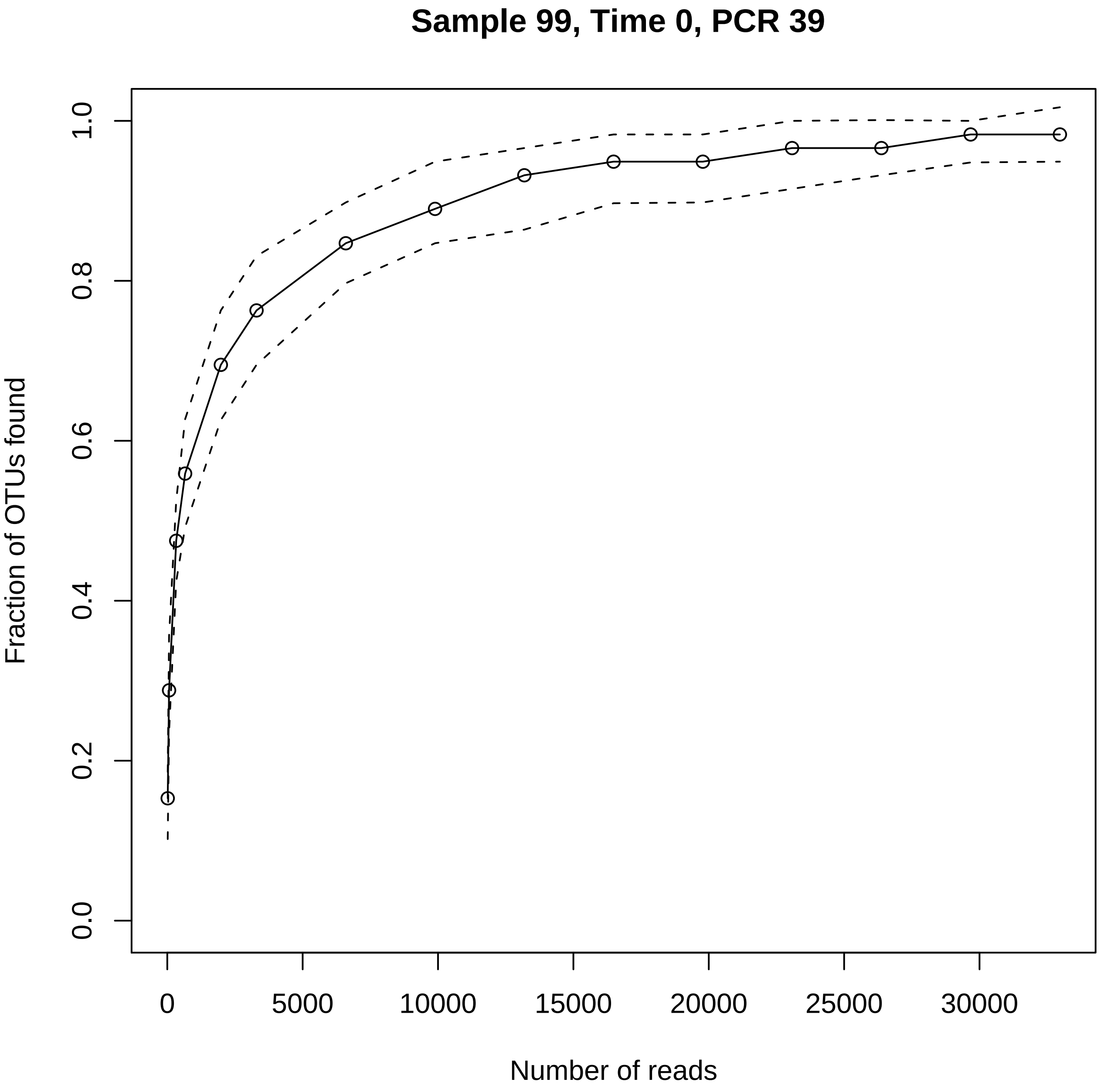
Sample 99, Time 0, PCR 39
Fraction of OTUs found
0.0
0.2
0.4
0.6
0.8
1.0
0
5000
10000
15000
20000
25000
30000
Number of reads

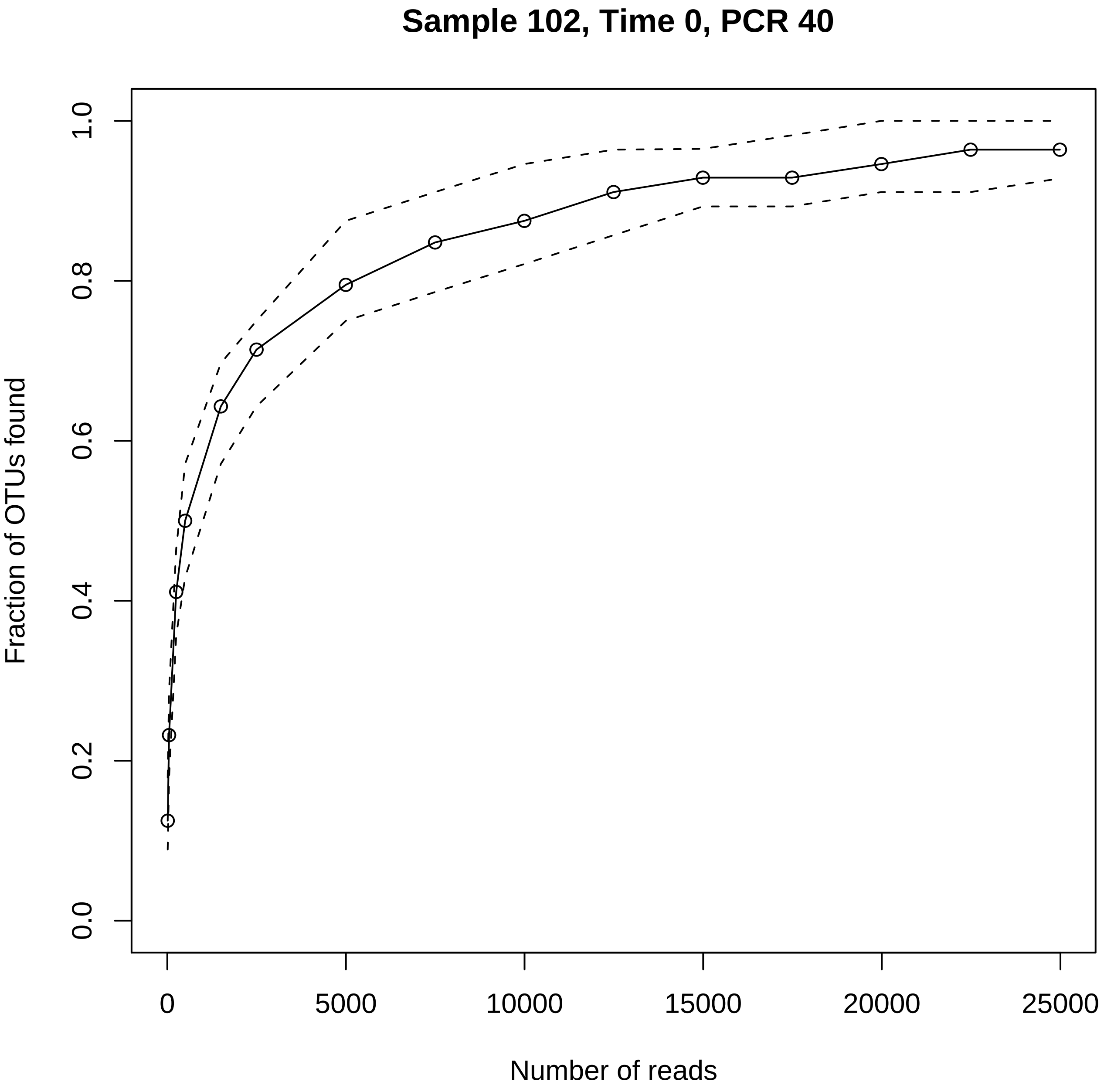

Sample 102, Time 0, PCR 40
Fraction of OTUs found
0.0
0.2
0.4
0.6
0.8
1.0
0
5000
10000
15000
20000
25000
Number of reads

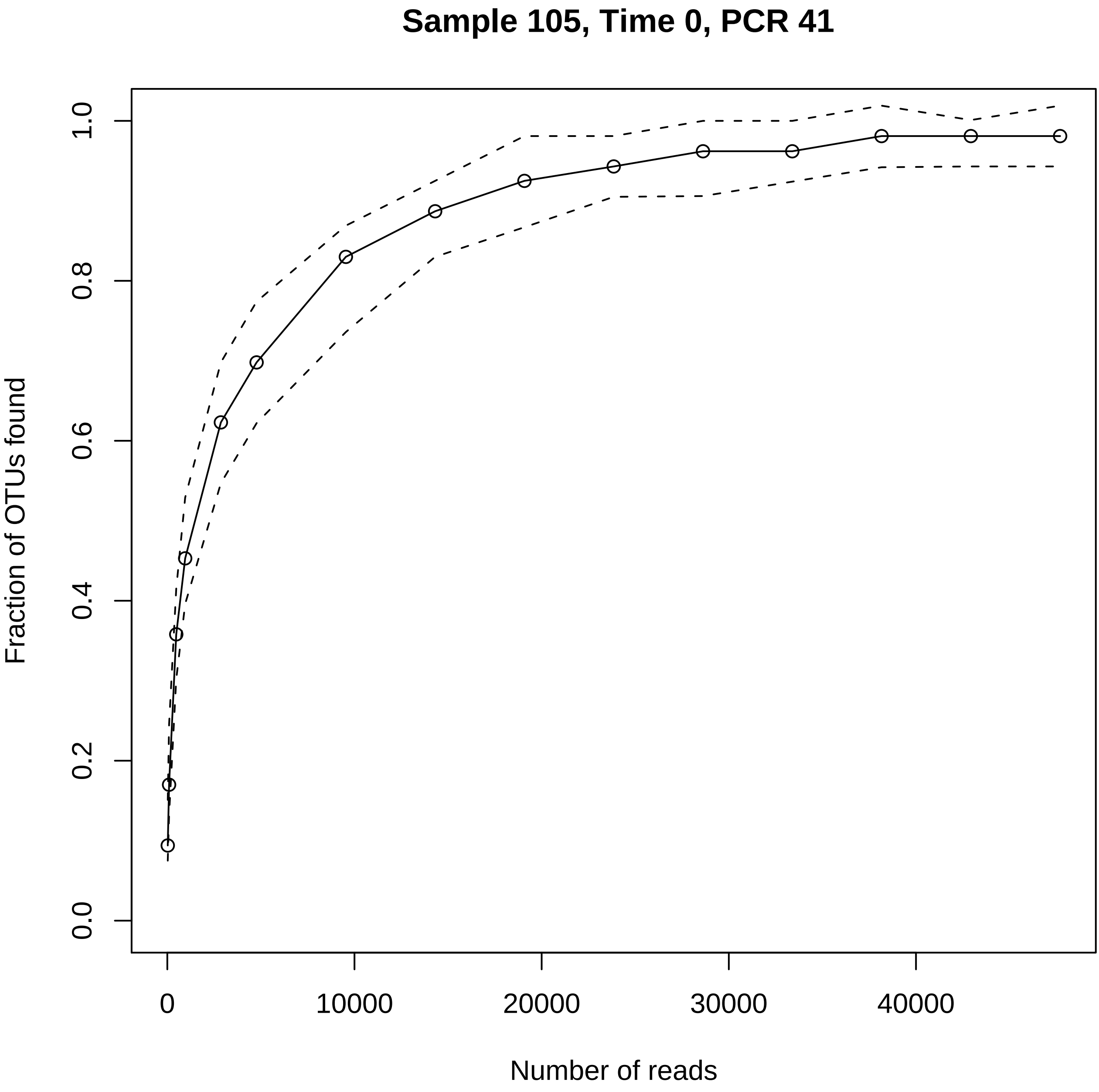

Sample 105, Time 0, PCR 41
Fraction of OTUs found
0.0
0.2
0.4
0.6
0.8
1.0
0
10000
20000
30000
40000
Number of reads

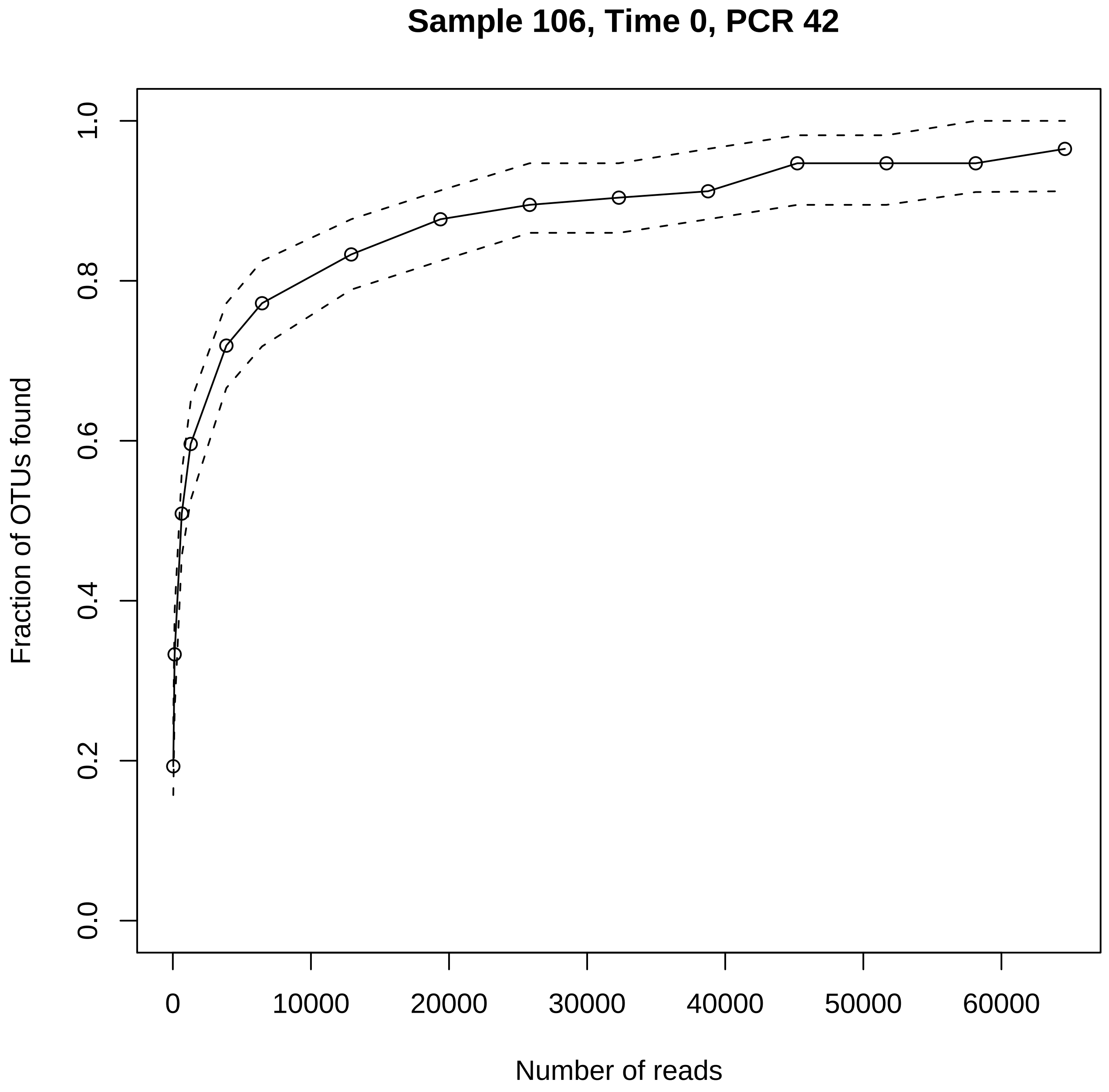
Sample 106, Time 0, PCR 42
Fraction of OTUs found
0.0
0.2
0.4
0.6
0.8
1.0
0
10000
20000
30000
40000
50000
60000
Number of reads

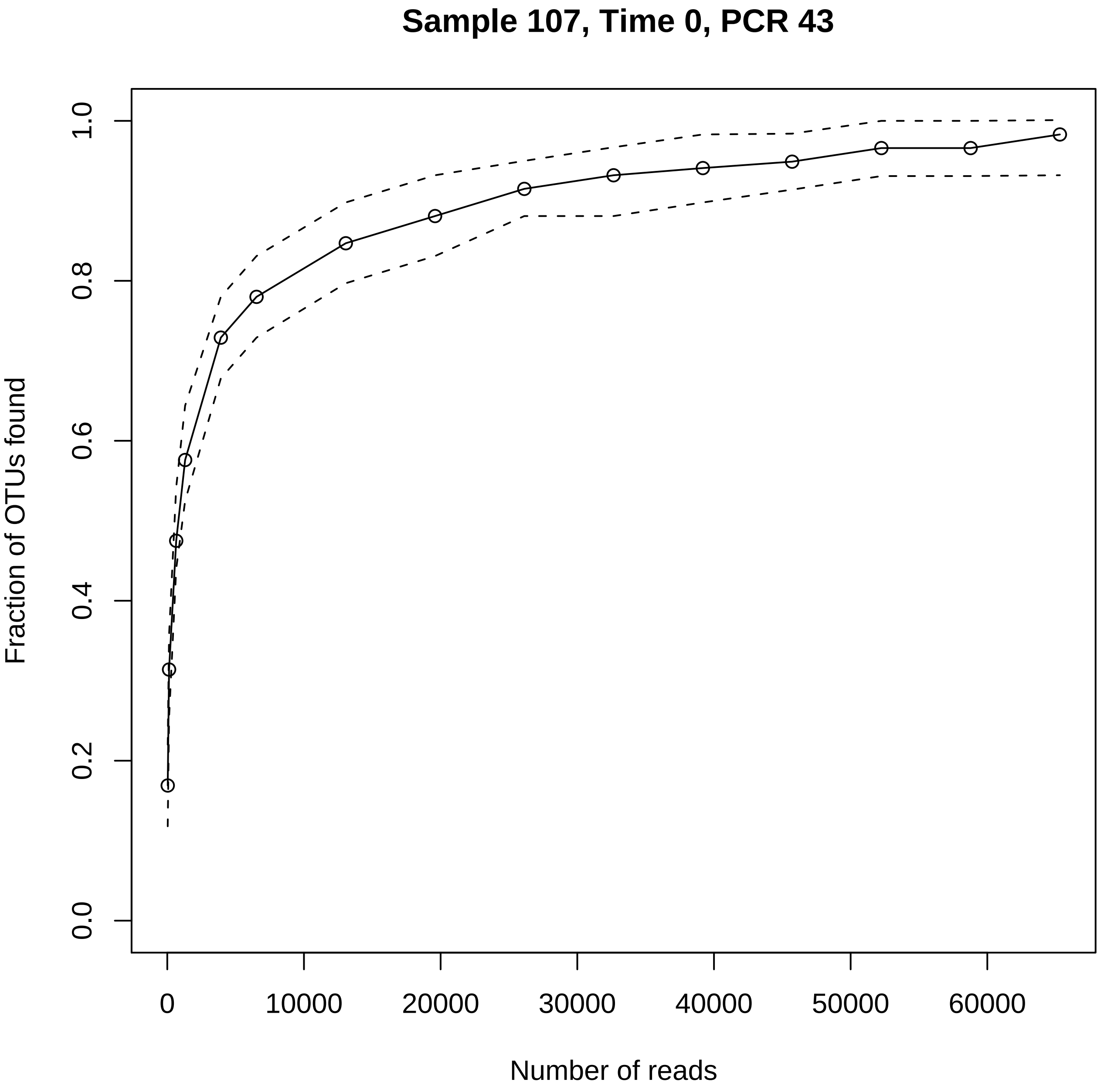
Sample 107, Time 0, PCR 43
Fraction of OTUs found
0.0
0.2
0.4
0.6
0.8
1.0
0
10000
20000
30000
40000
50000
60000
Number of reads

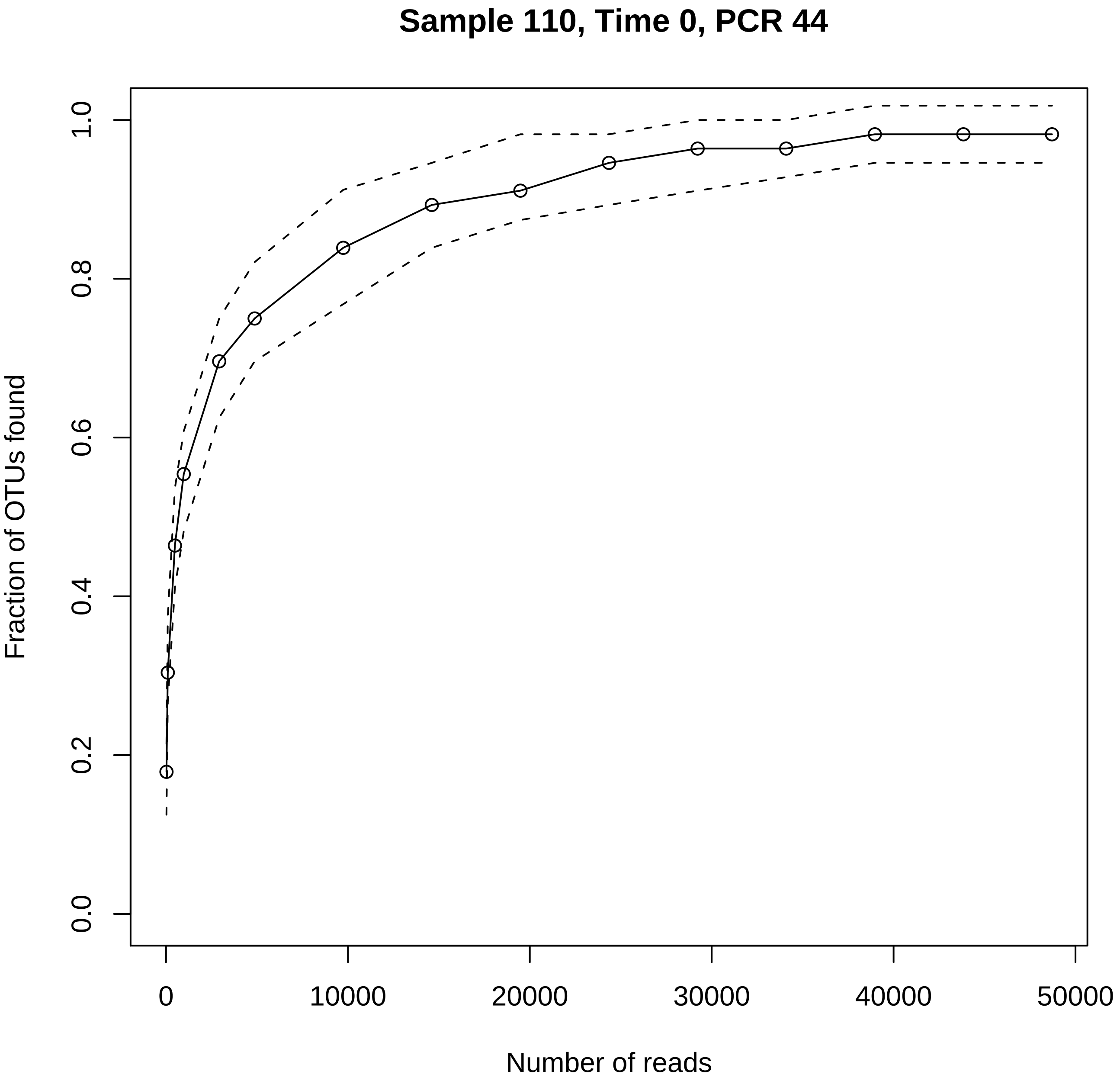
Sample 110, Time 0, PCR 44
Fraction of OTUs found
0.0
0.2
0.4
0.6
0.8
1.0
0
10000
20000
30000
40000
50000
Number of reads

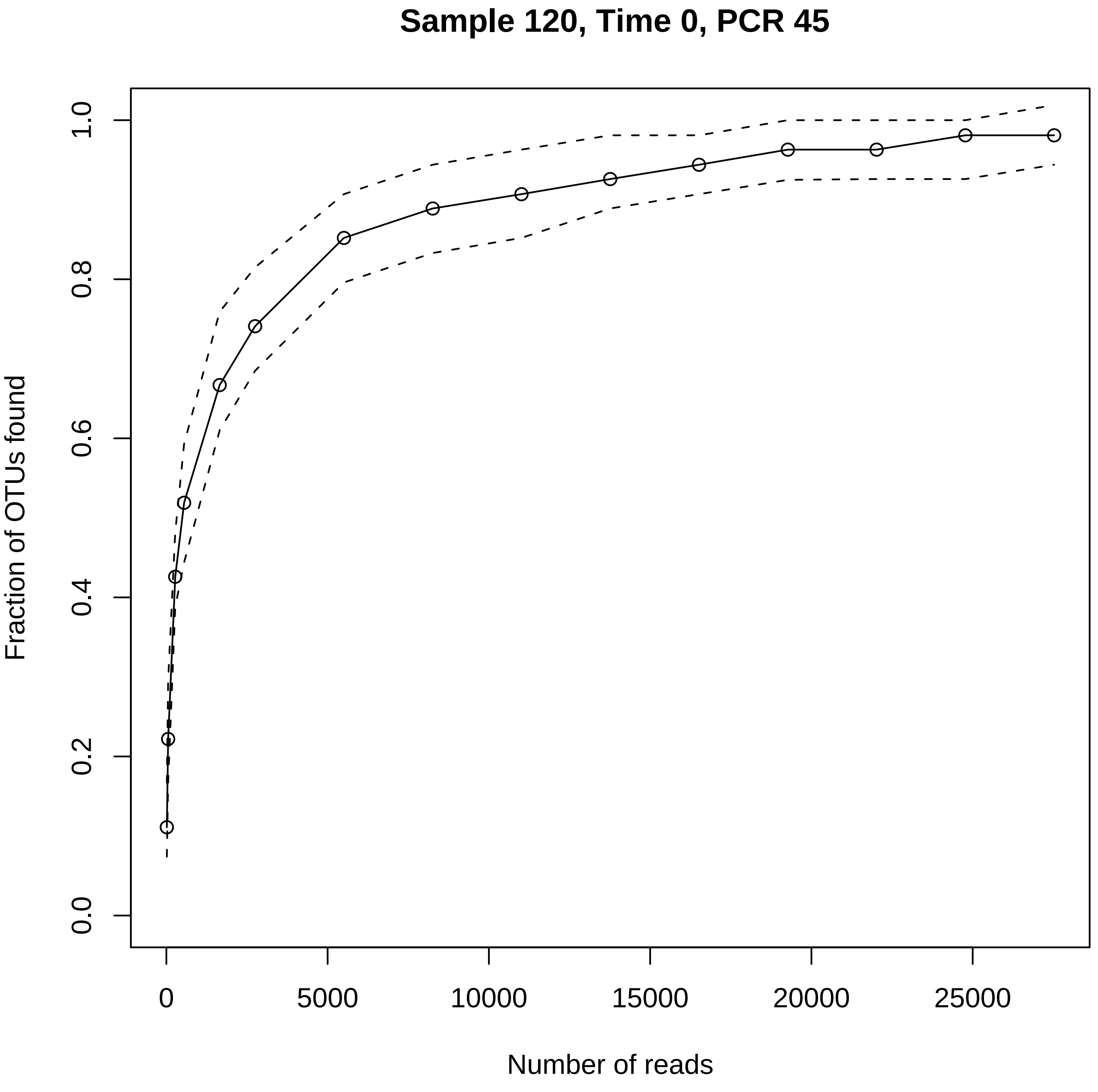
Sample 120, Time 0, PCR 45
Fraction of OTUs found
Number of reads
0.0
0.2
0.4
0.6
0.8
1.0
0
5000
10000
15000
20000
25000

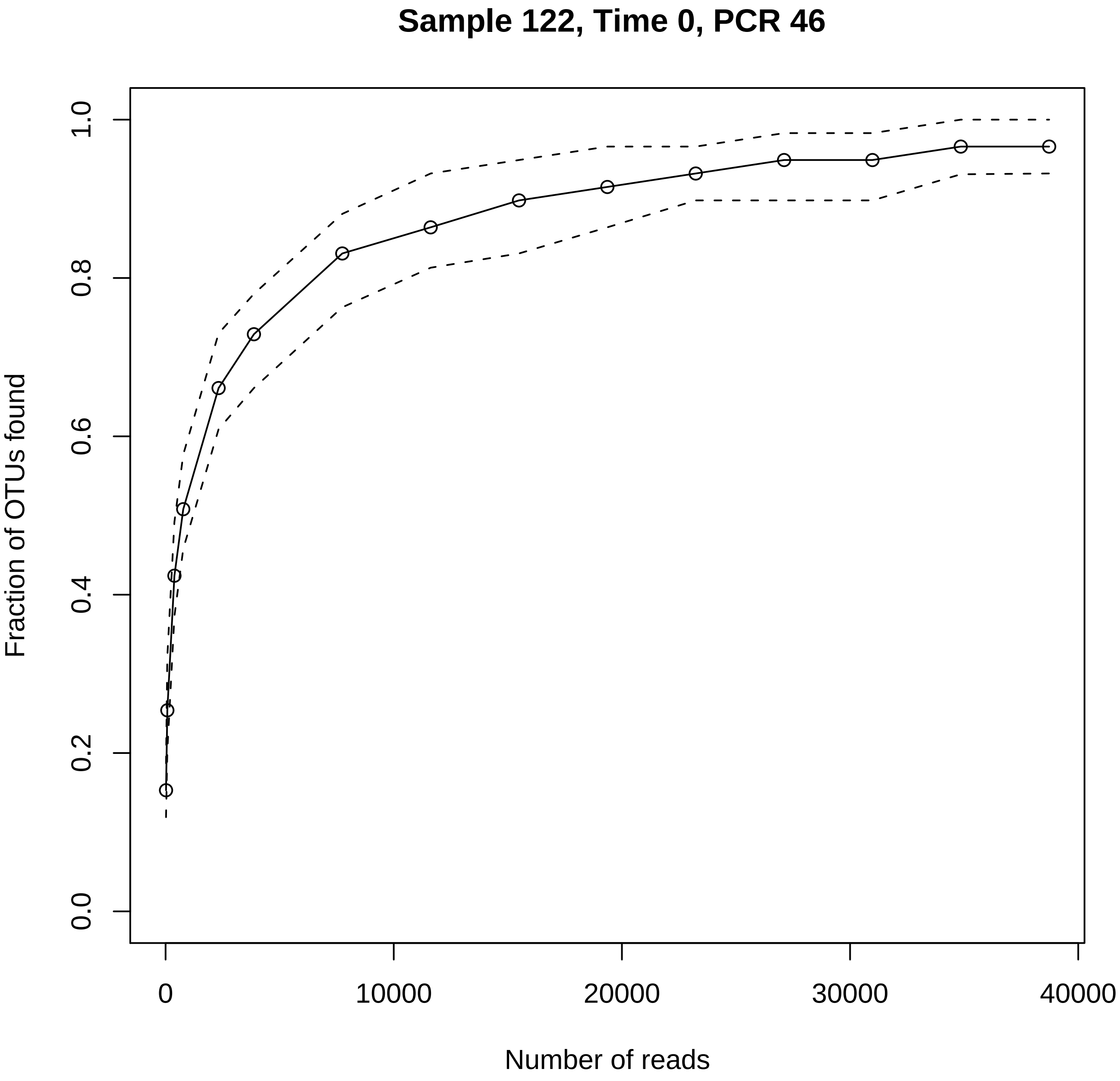
Sample 122, Time 0, PCR 46
Fraction of OTUs found
0.0
0.2
0.4
0.6
0.8
1.0
0
10000
20000
30000
40000
Number of reads

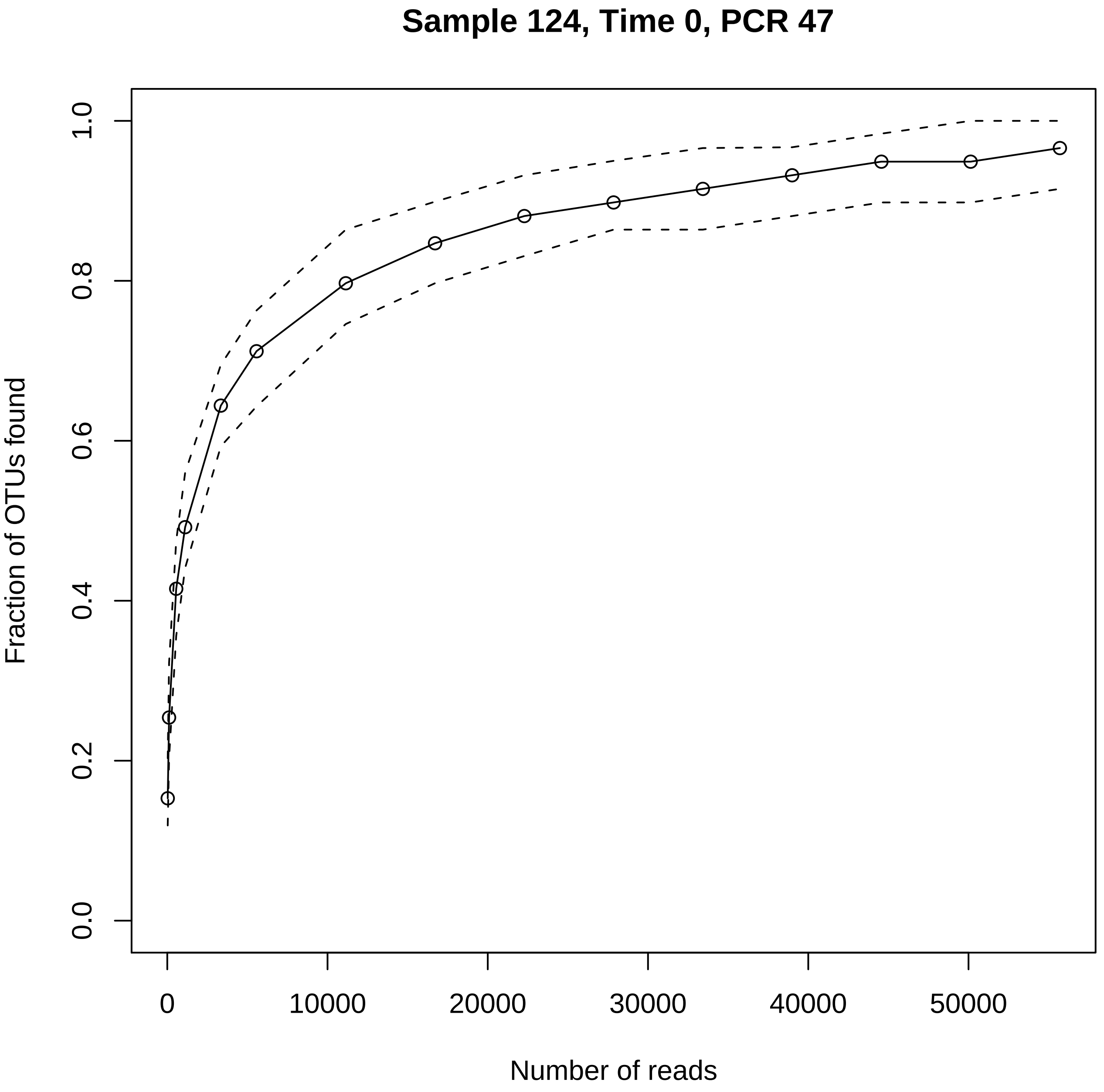

Sample 124, Time 0, PCR 47
Fraction of OTUs found
0.0
0.2
0.4
0.6
0.8
1.0
0
10000
20000
30000
40000
50000
Number of reads

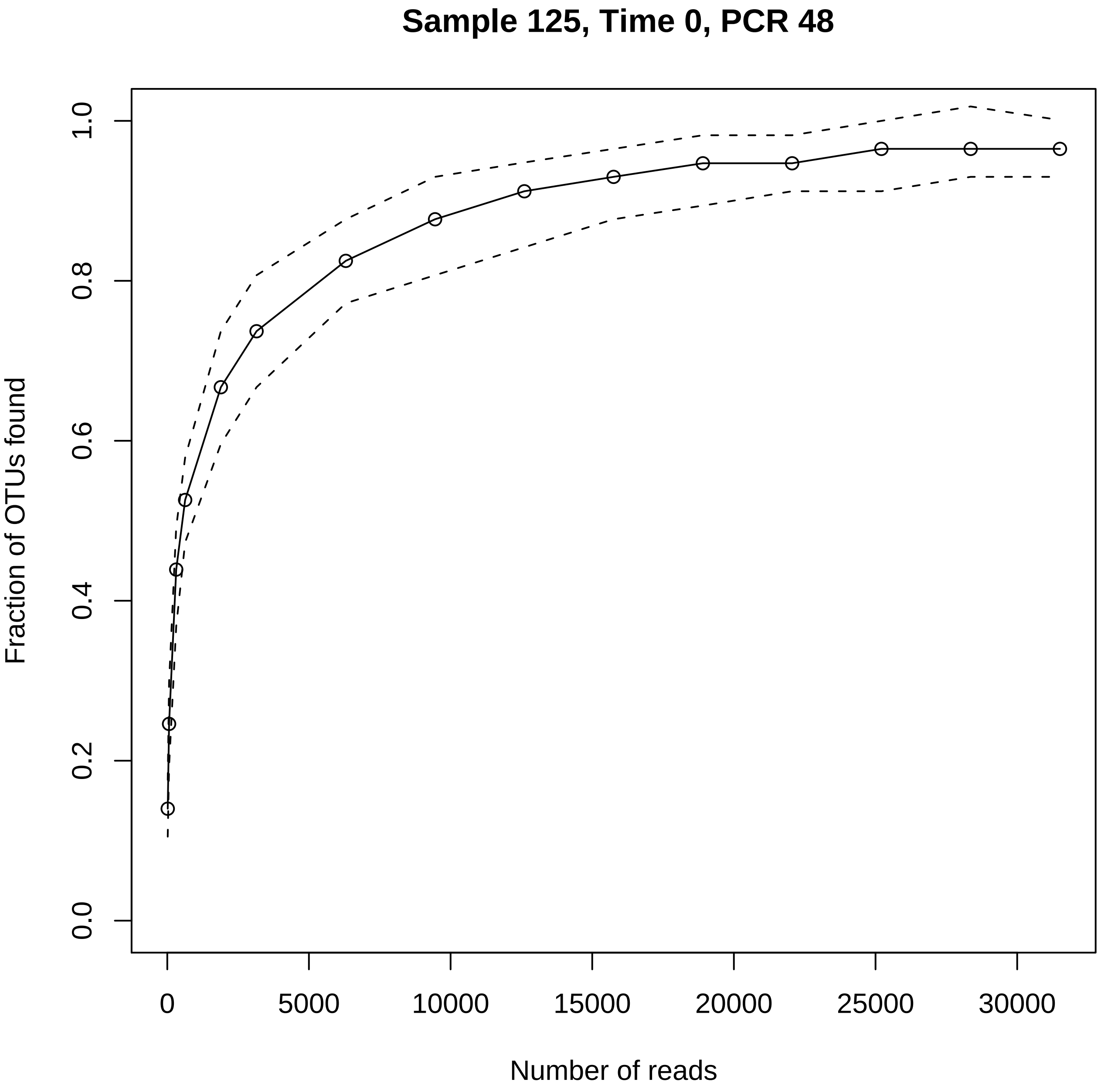
Sample 125, Time 0, PCR 48
Fraction of OTUs found
0.0
0.2
0.4
0.6
0.8
1.0
0
5000
10000
15000
20000
25000
30000
Number of reads

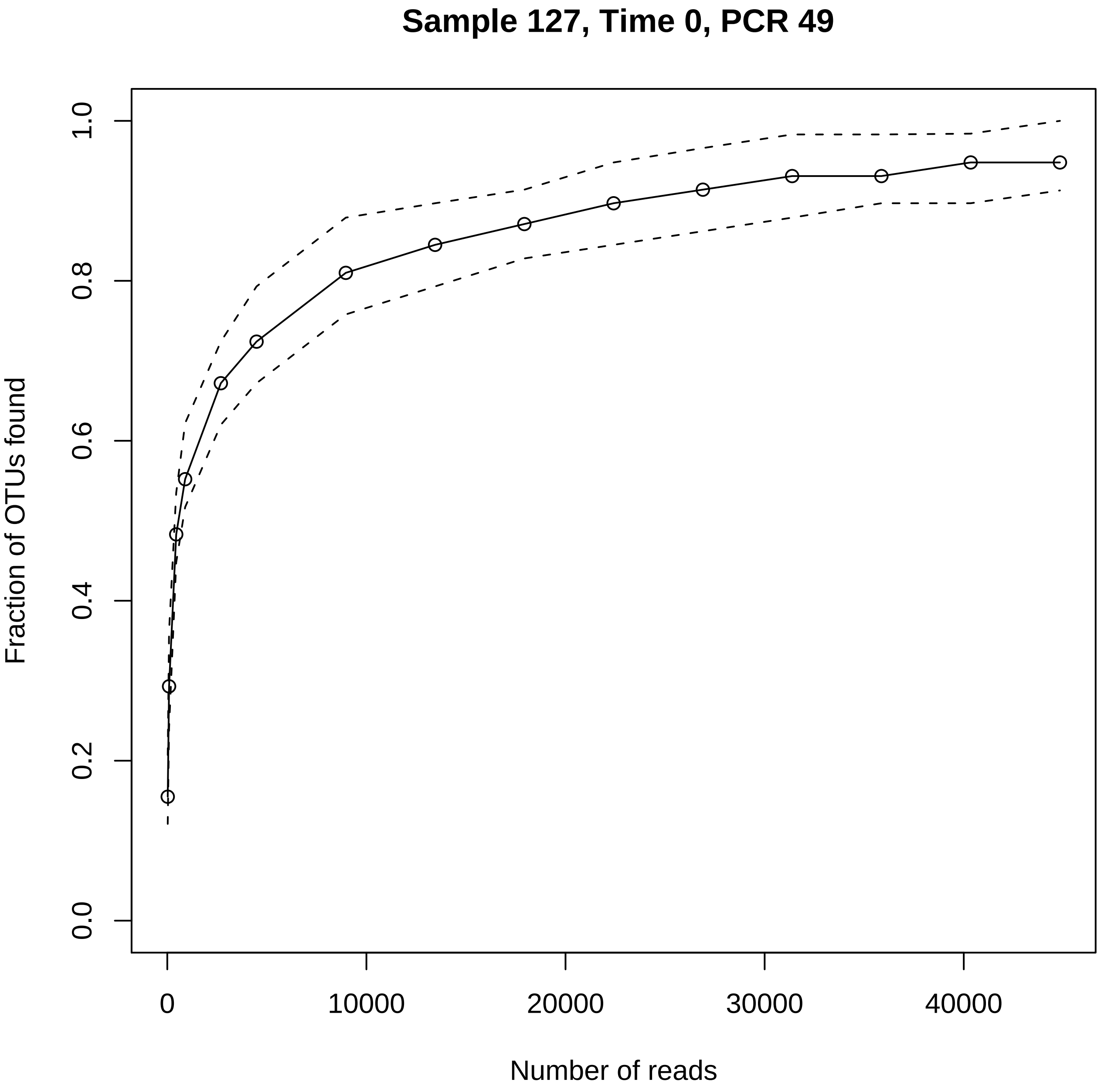
Sample 127, Time 0, PCR 49
Fraction of OTUs found
0.0
0.2
0.4
0.6
0.8
1.0
0
10000
20000
30000
40000
Number of reads

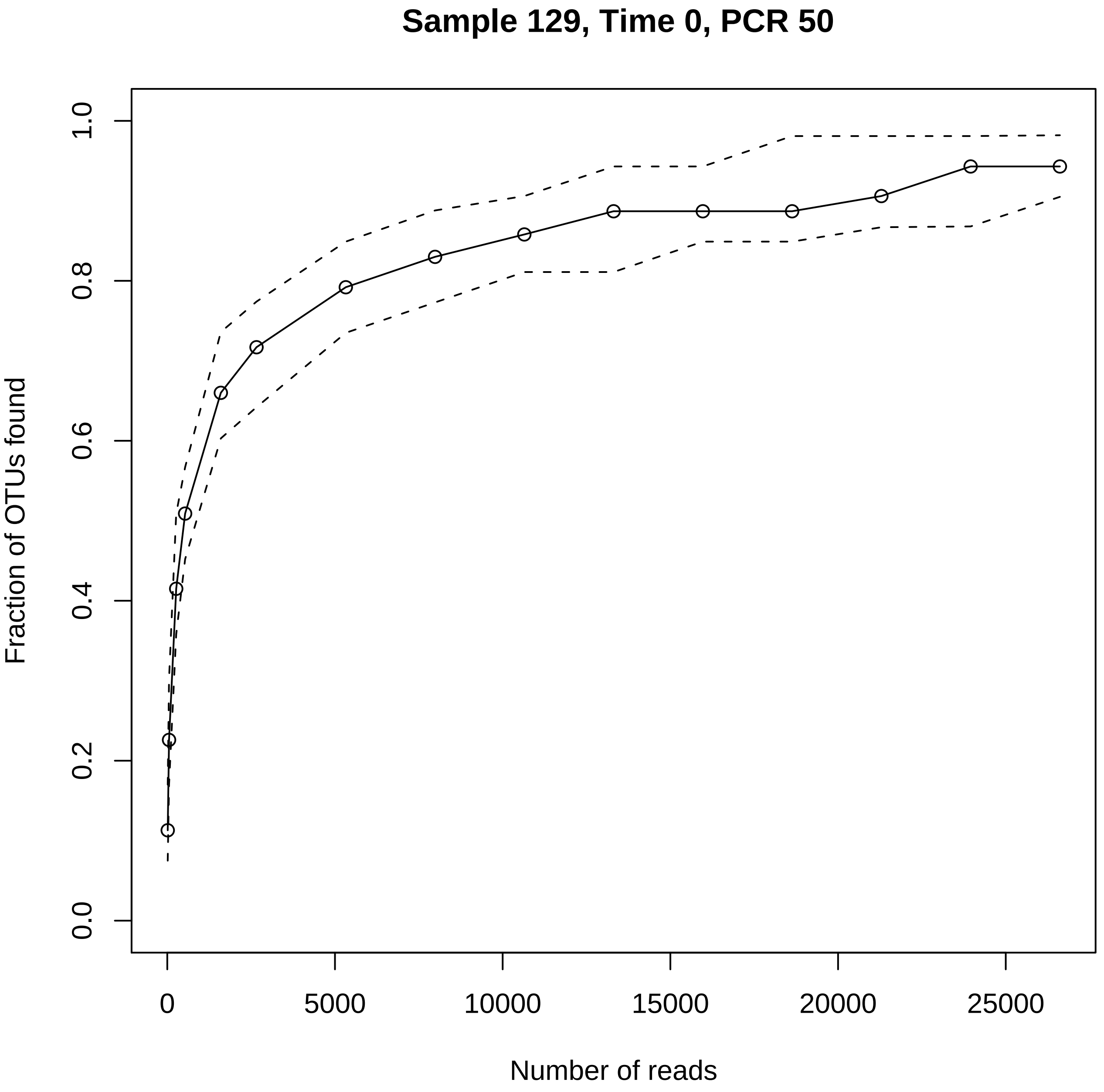

Sample 129, Time 0, PCR 50
Fraction of OTUs found
0.0
0.2
0.4
0.6
0.8
1.0
0
5000
10000
15000
20000
25000
Number of reads

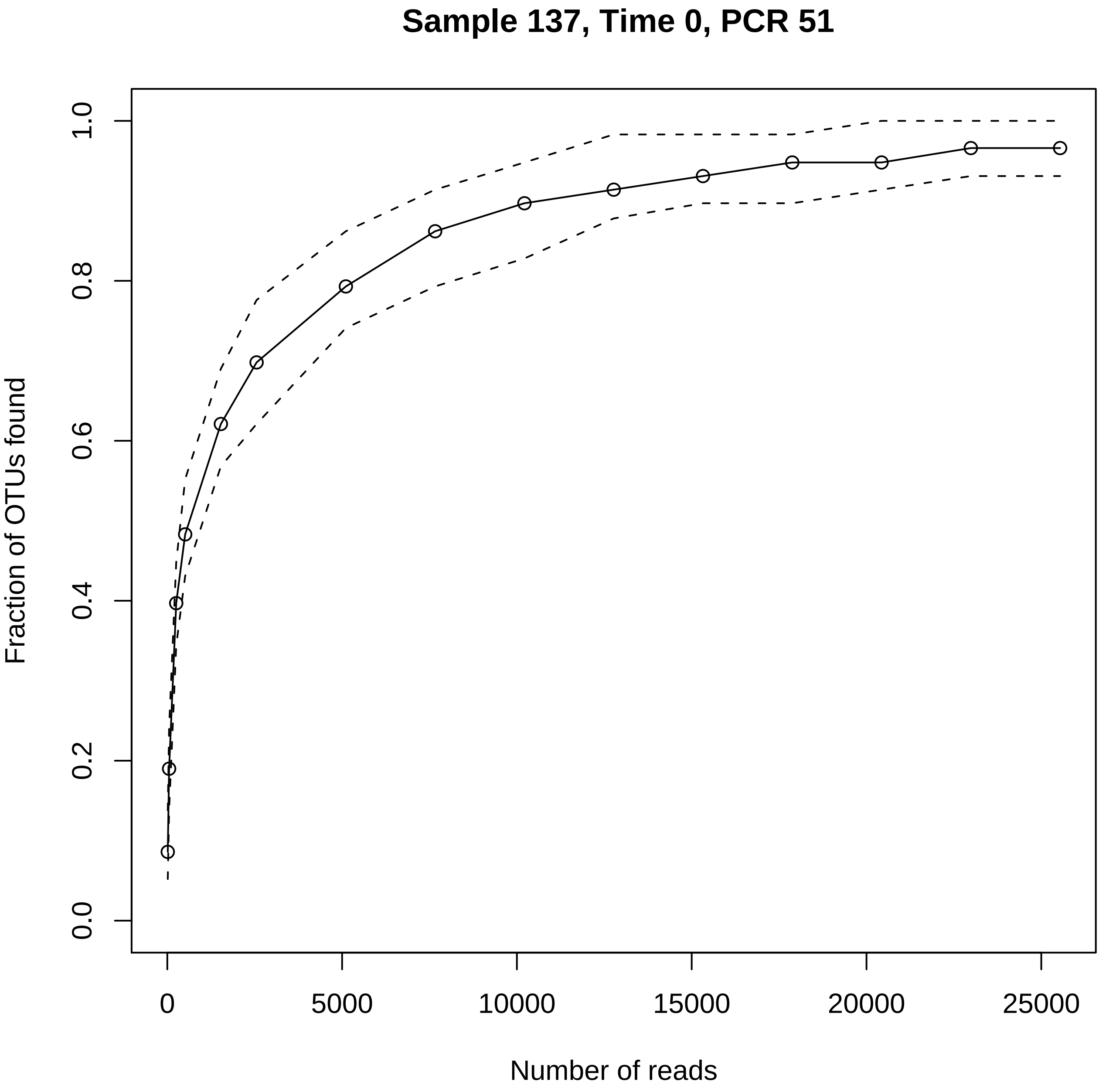

Sample 137, Time 0, PCR 51
Fraction of OTUs found
0.0
0.2
0.4
0.6
0.8
1.0
0
5000
10000
15000
20000
25000
Number of reads

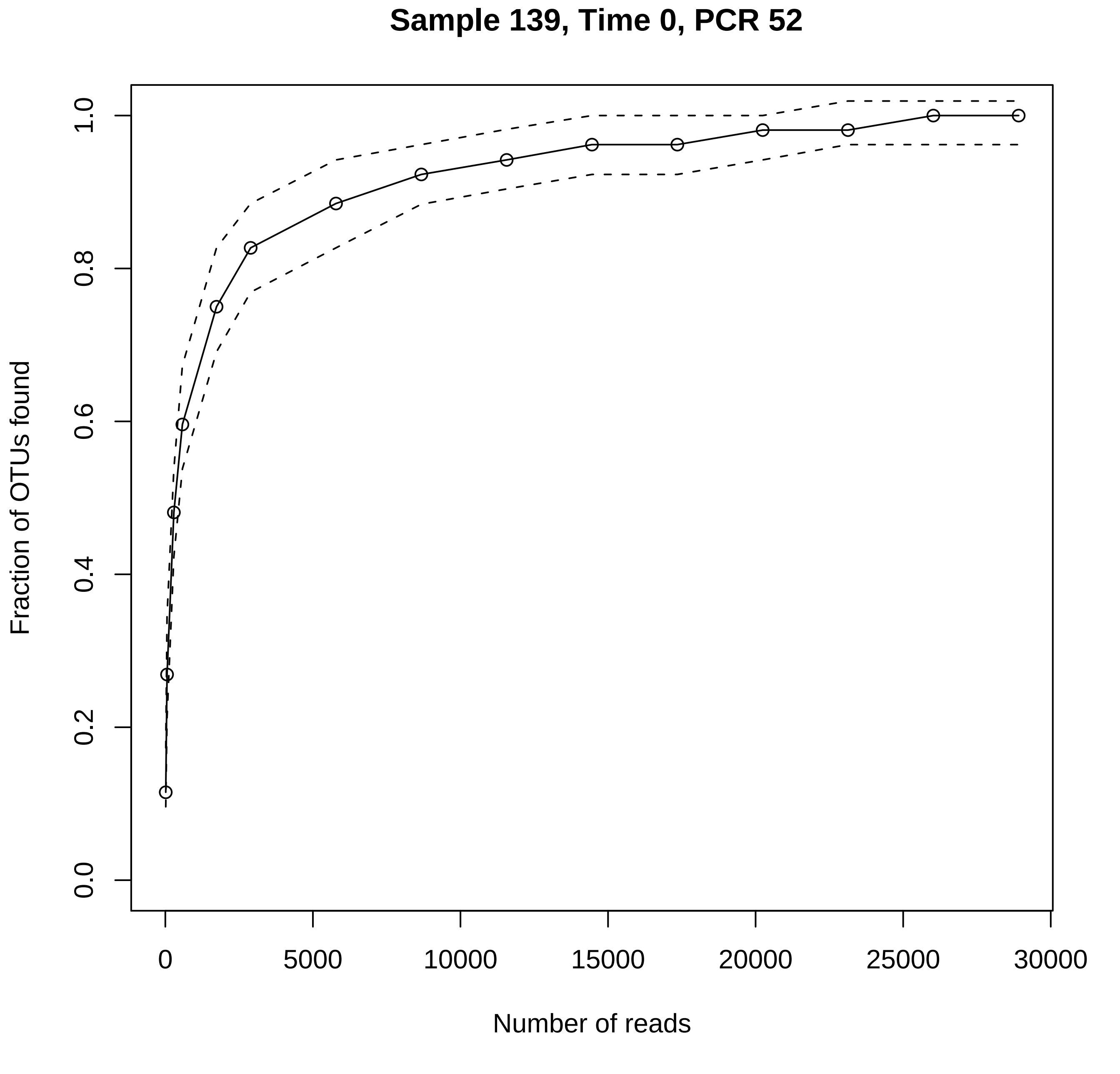
Sample 139, Time 0, PCR 52
Fraction of OTUs found
0.0
0.2
0.4
0.6
0.8
1.0
0
5000
10000
15000
20000
25000
30000
Number of reads

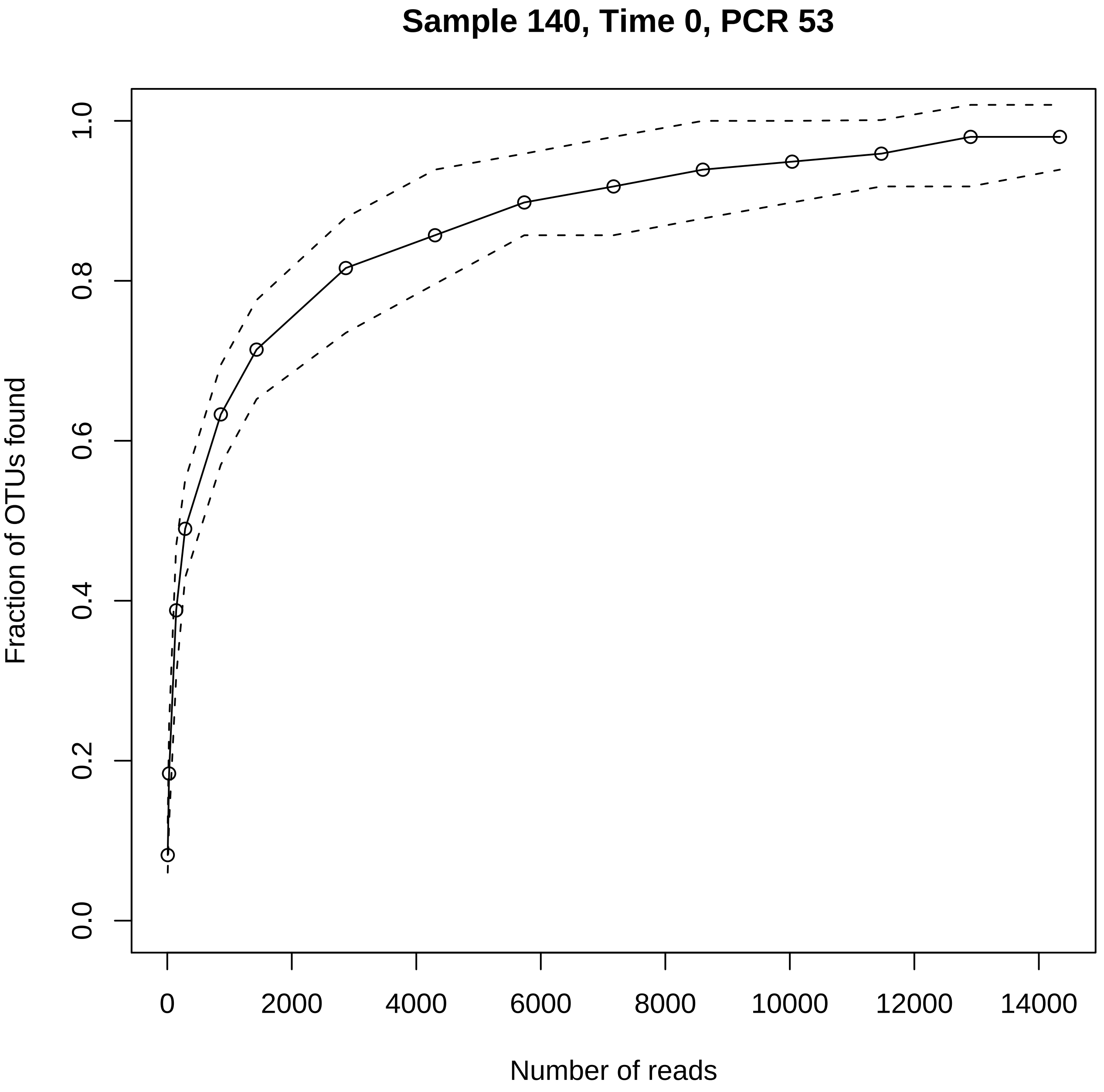
Sample 140, Time 0, PCR 53
Fraction of OTUs found
0.0
0.2
0.4
0.6
0.8
1.0
0
2000
4000
6000
8000
10000
12000
14000
Number of reads

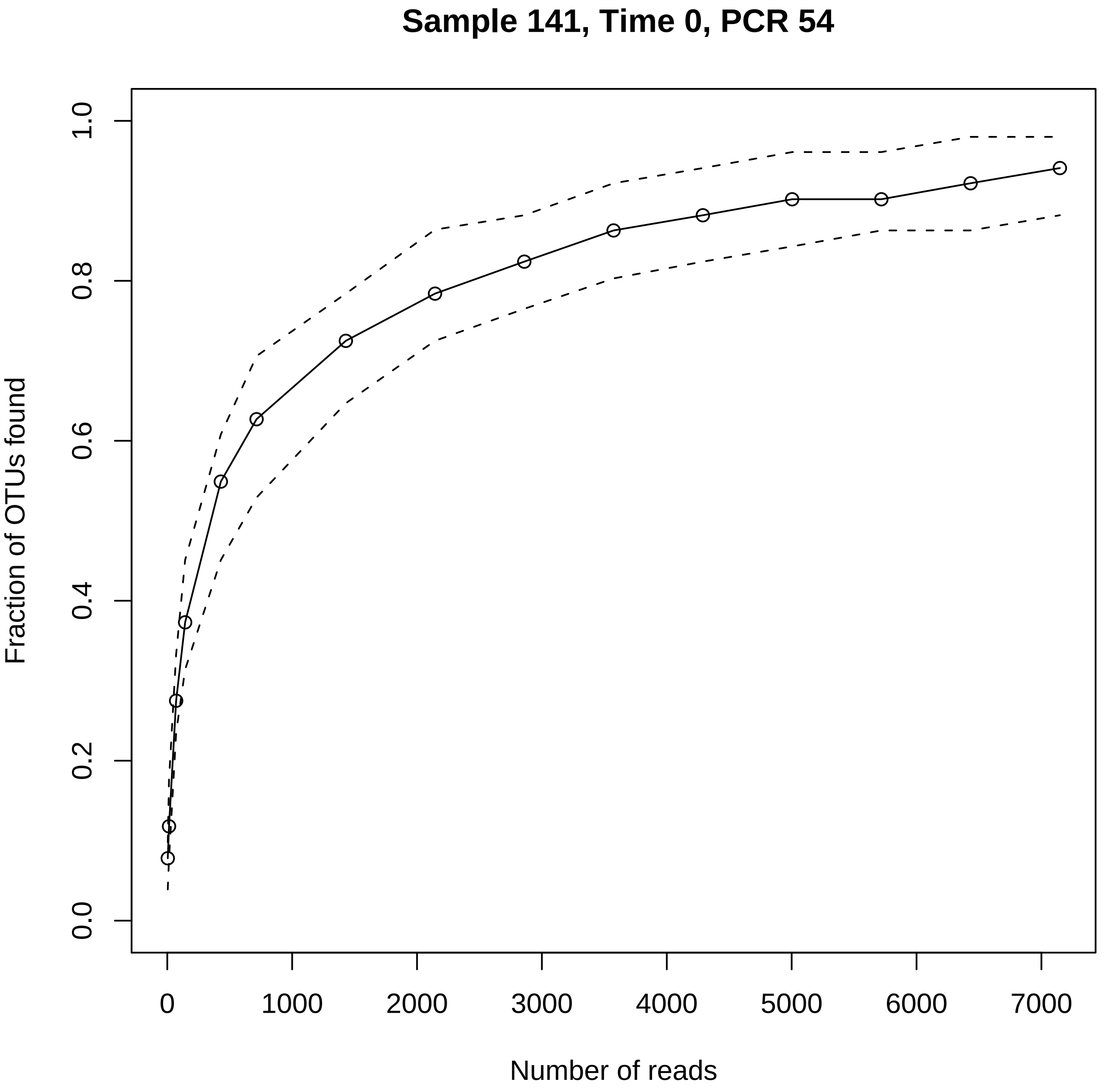

Sample 141, Time 0, PCR 54
Fraction of OTUs found
0.0
0.2
0.4
0.6
0.8
1.0
0
1000
2000
3000
4000
5000
6000
7000
Number of reads

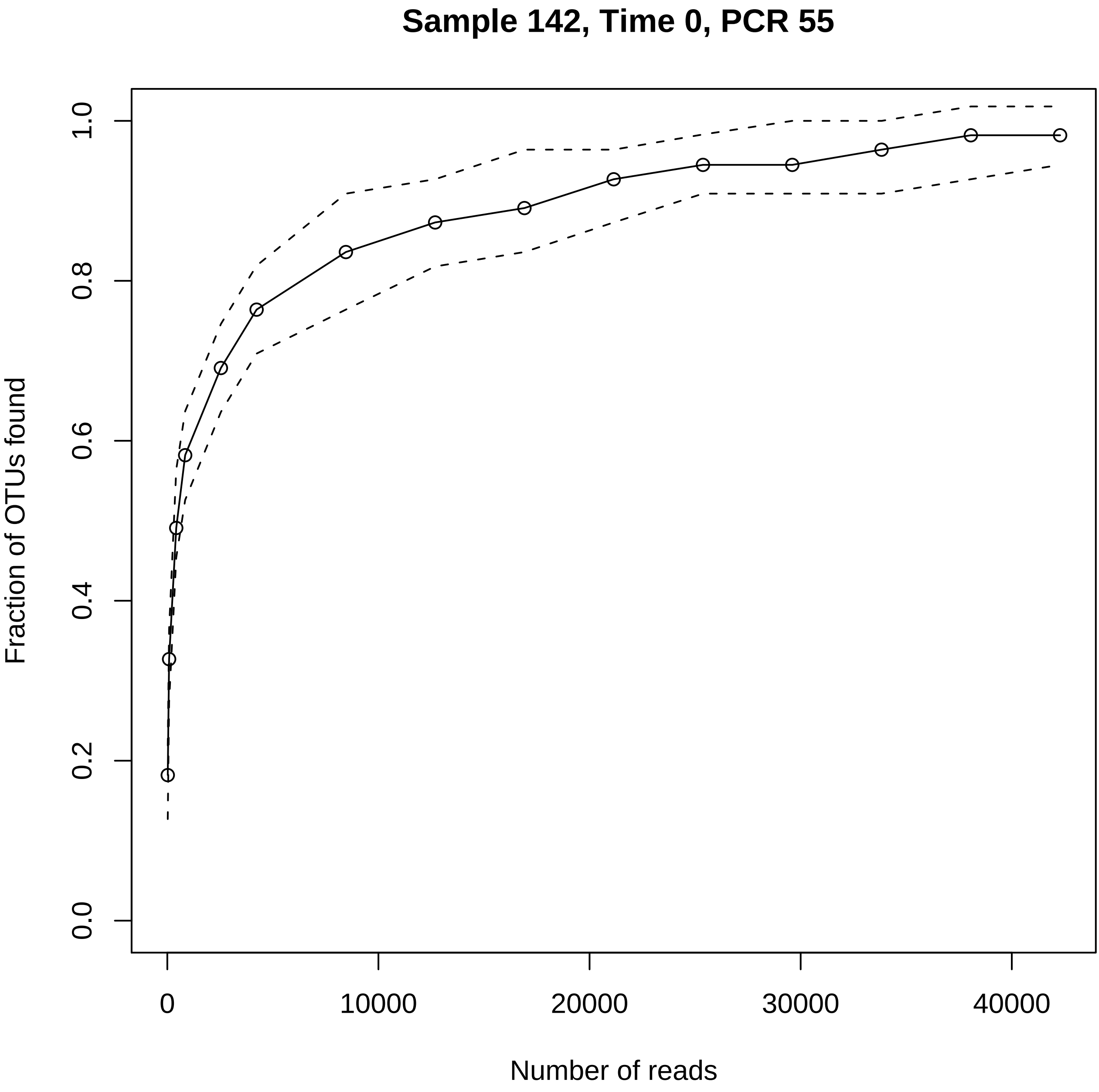
Sample 142, Time 0, PCR 55
Fraction of OTUs found
0.0
0.2
0.4
0.6
0.8
1.0
0
10000
20000
30000
40000
Number of reads

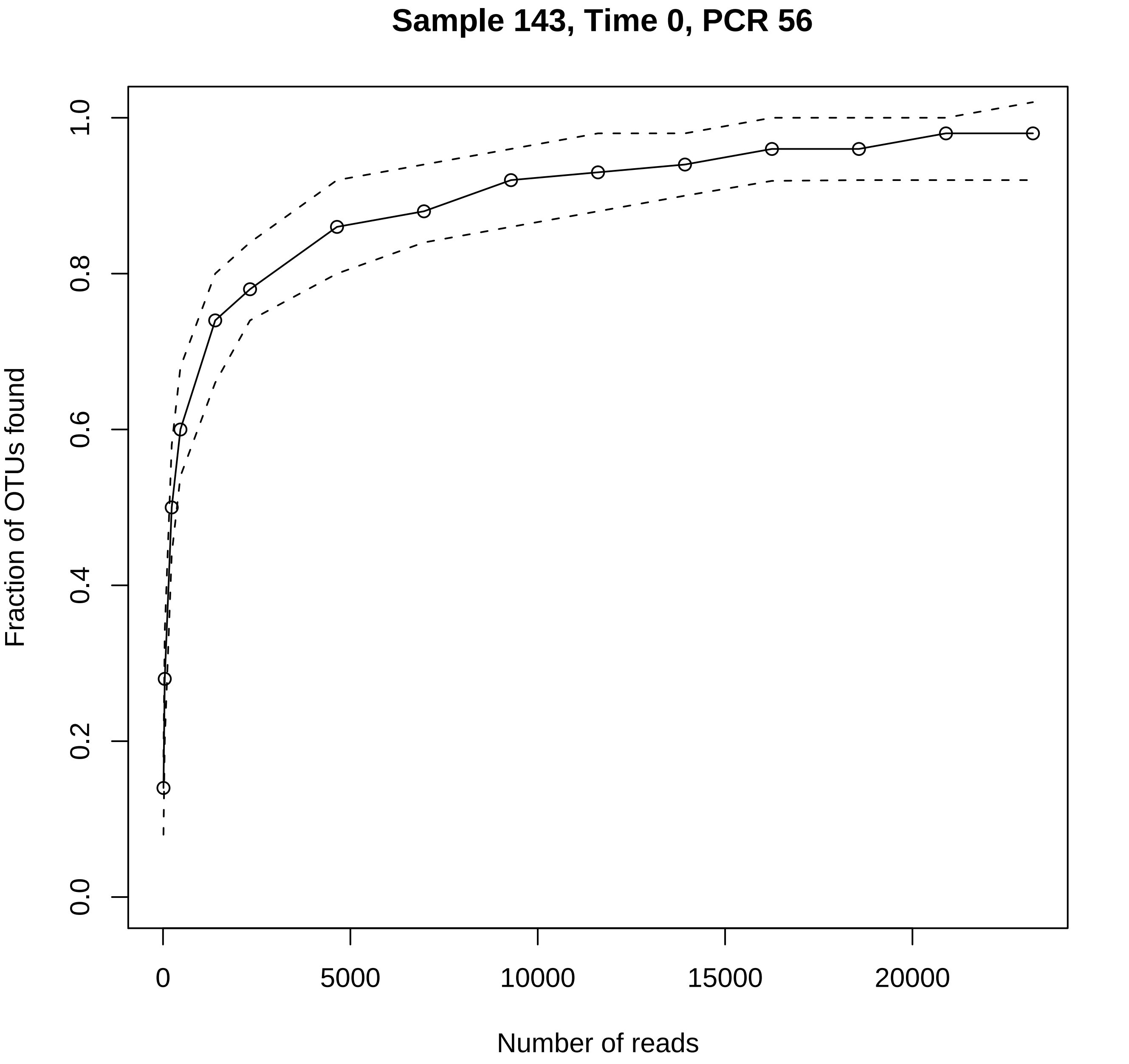
Sample 143, Time 0, PCR 56
Fraction of OTUs found
0.0
0.2
0.4
0.6
0.8
1.0
0
5000
10000
15000
20000
Number of reads

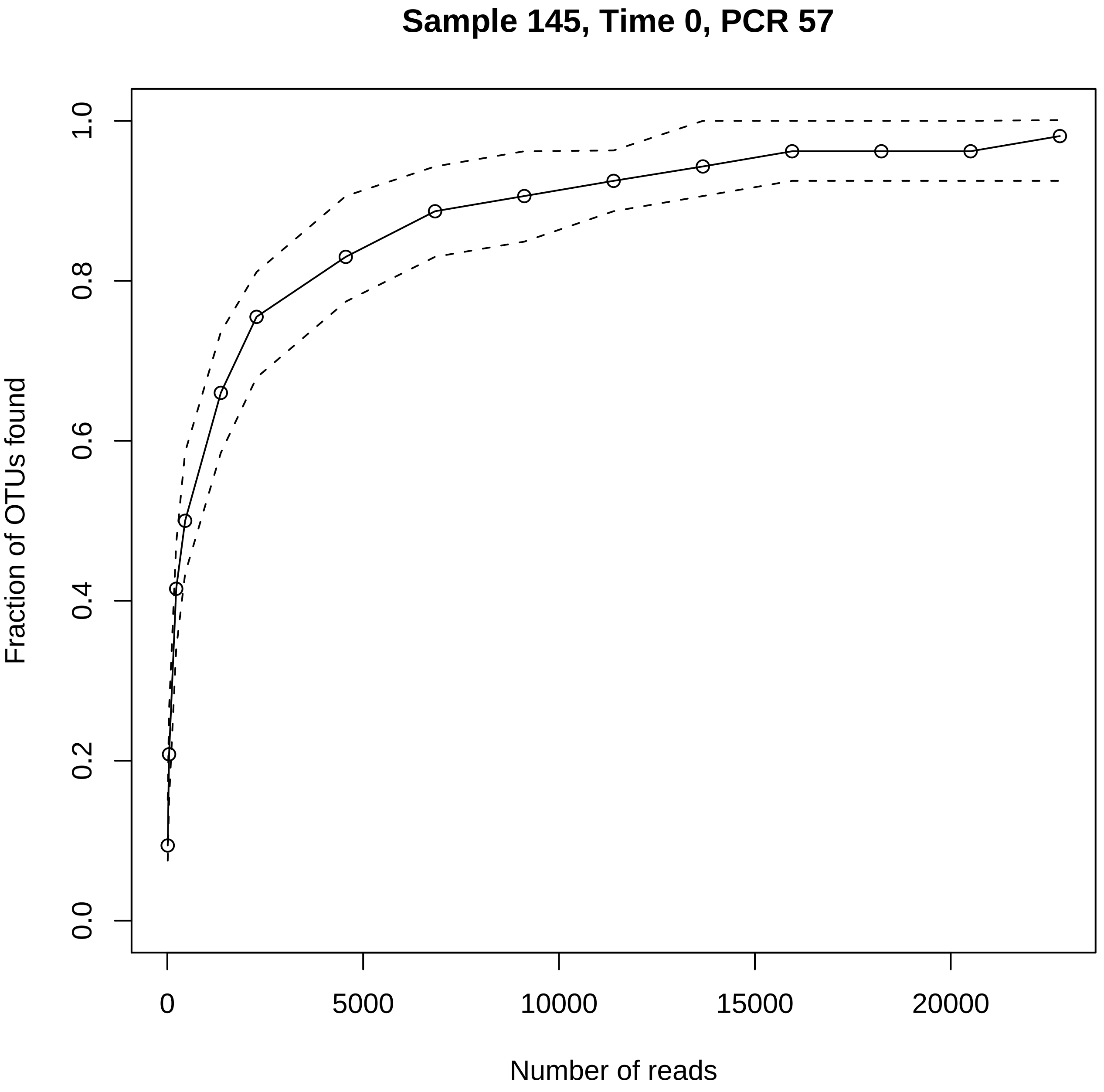
Sample 145, Time 0, PCR 57
Fraction of OTUs found
0.0
0.2
0.4
0.6
0.8
1.0
0
5000
10000
15000
20000
Number of reads

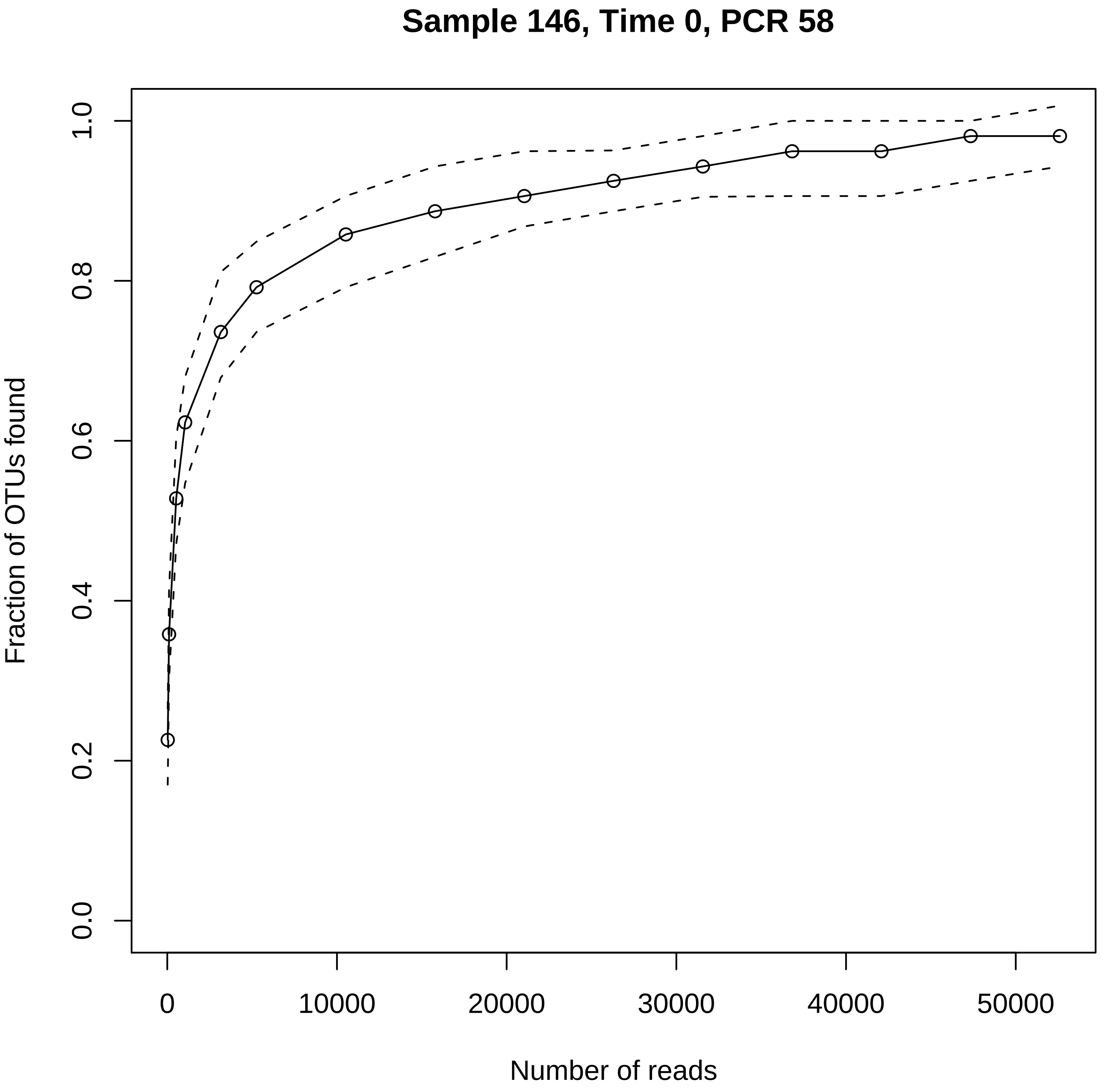
Sample 146, Time 0, PCR 58
Fraction of OTUs found
Number of reads
0.0
0.2
0.4
0.6
0.8
1.0
0
10000
20000
30000
40000
50000

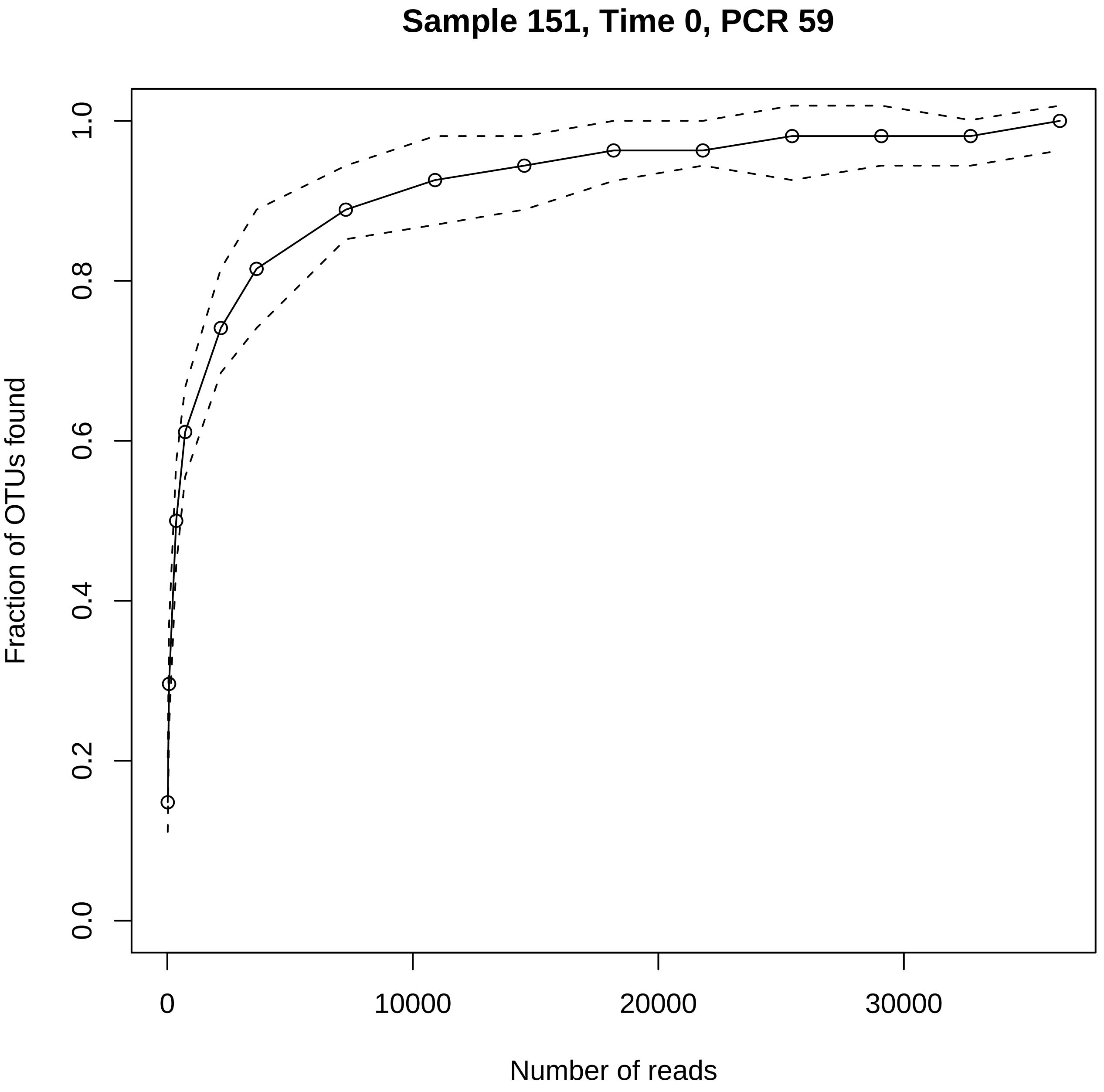
Sample 151, Time 0, PCR 59
Fraction of OTUs found
0.0
0.2
0.4
0.6
0.8
1.0
0
10000
20000
30000
Number of reads

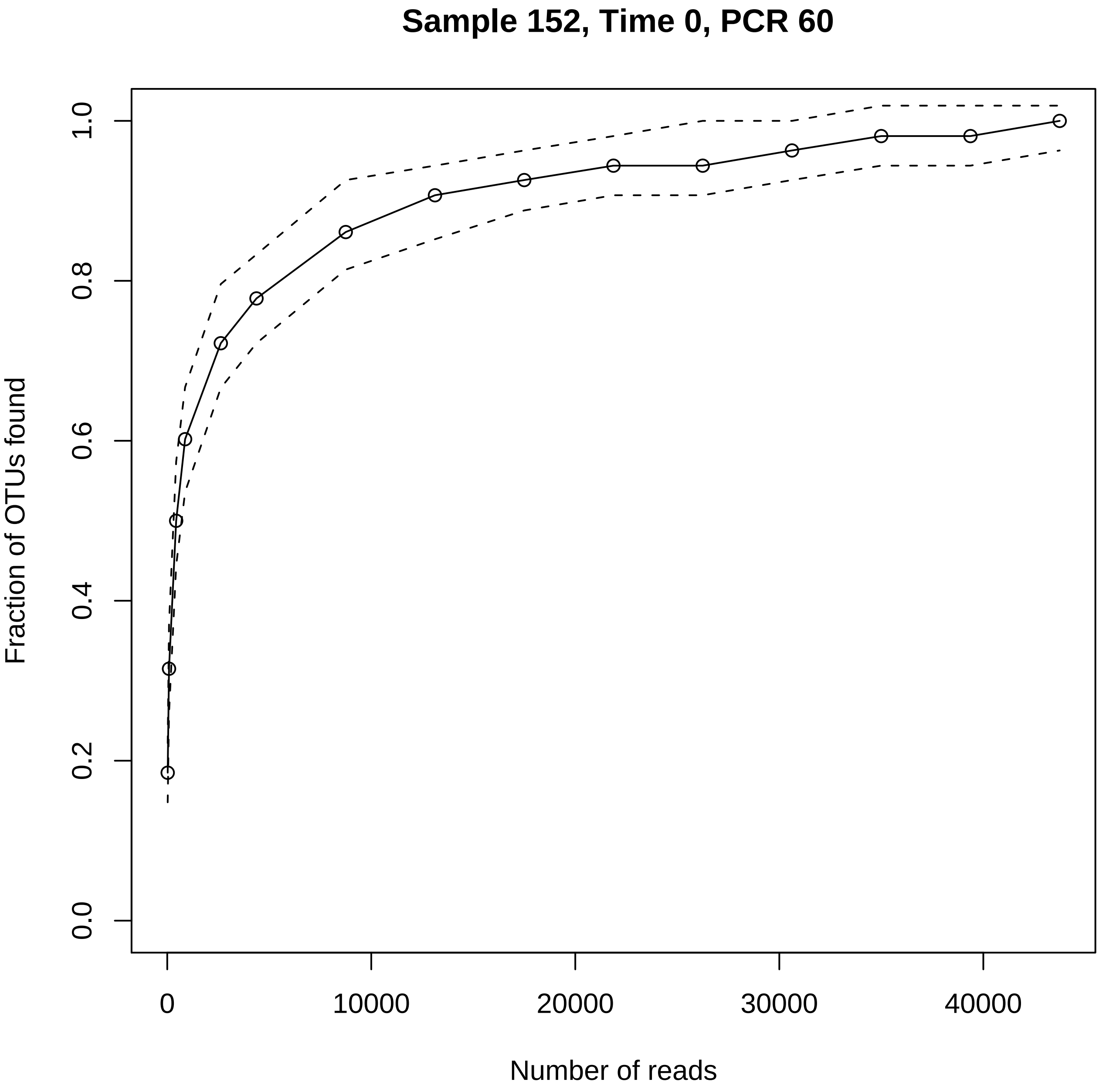
Sample 152, Time 0, PCR 60
Fraction of OTUs found
0.0
0.2
0.4
0.6
0.8
1.0
0
10000
20000
30000
40000
Number of reads

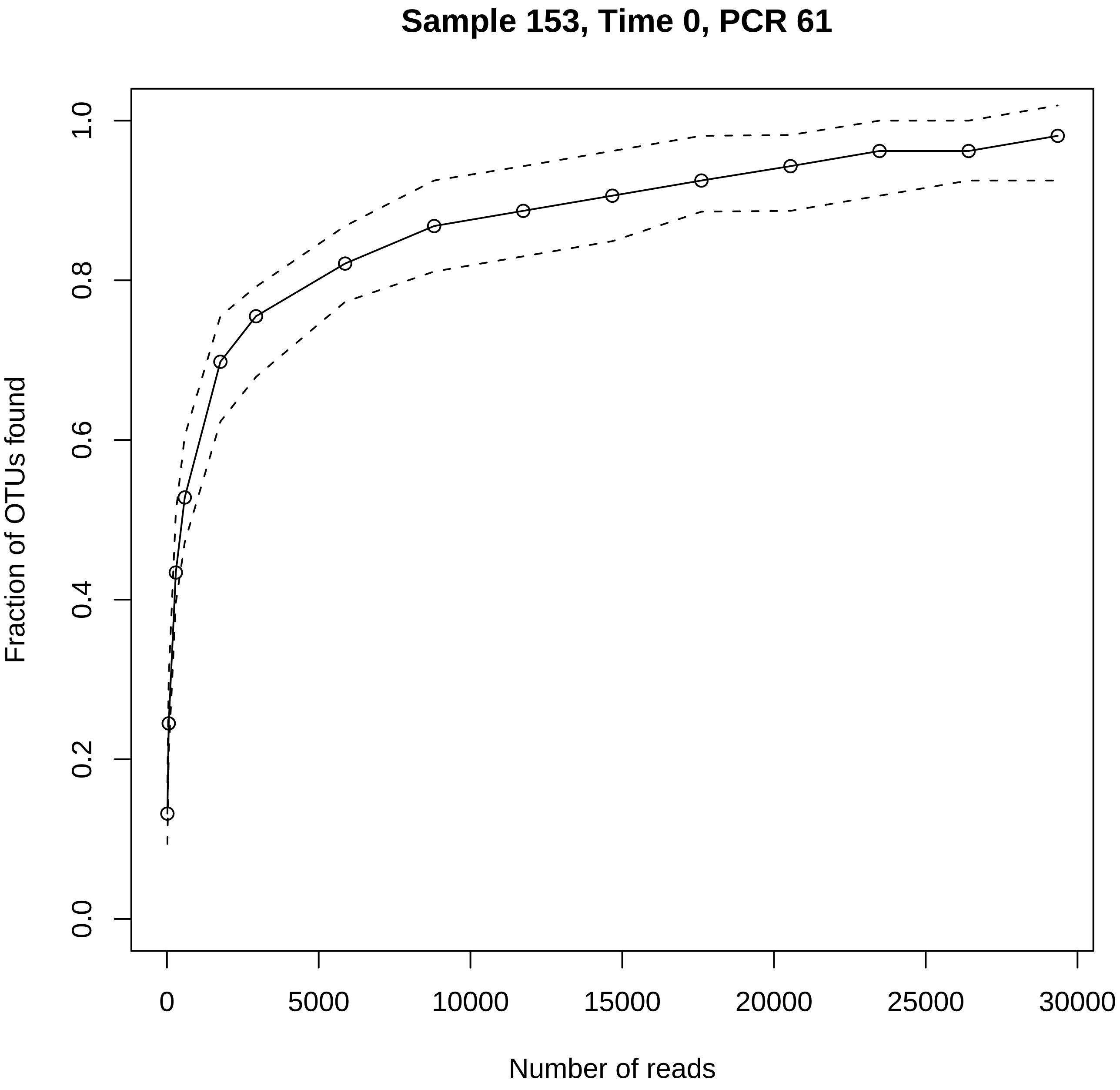
Sample 153, Time 0, PCR 61
Fraction of OTUs found
0.0
0.2
0.4
0.6
0.8
1.0
0
5000
10000
15000
20000
25000
30000
Number of reads

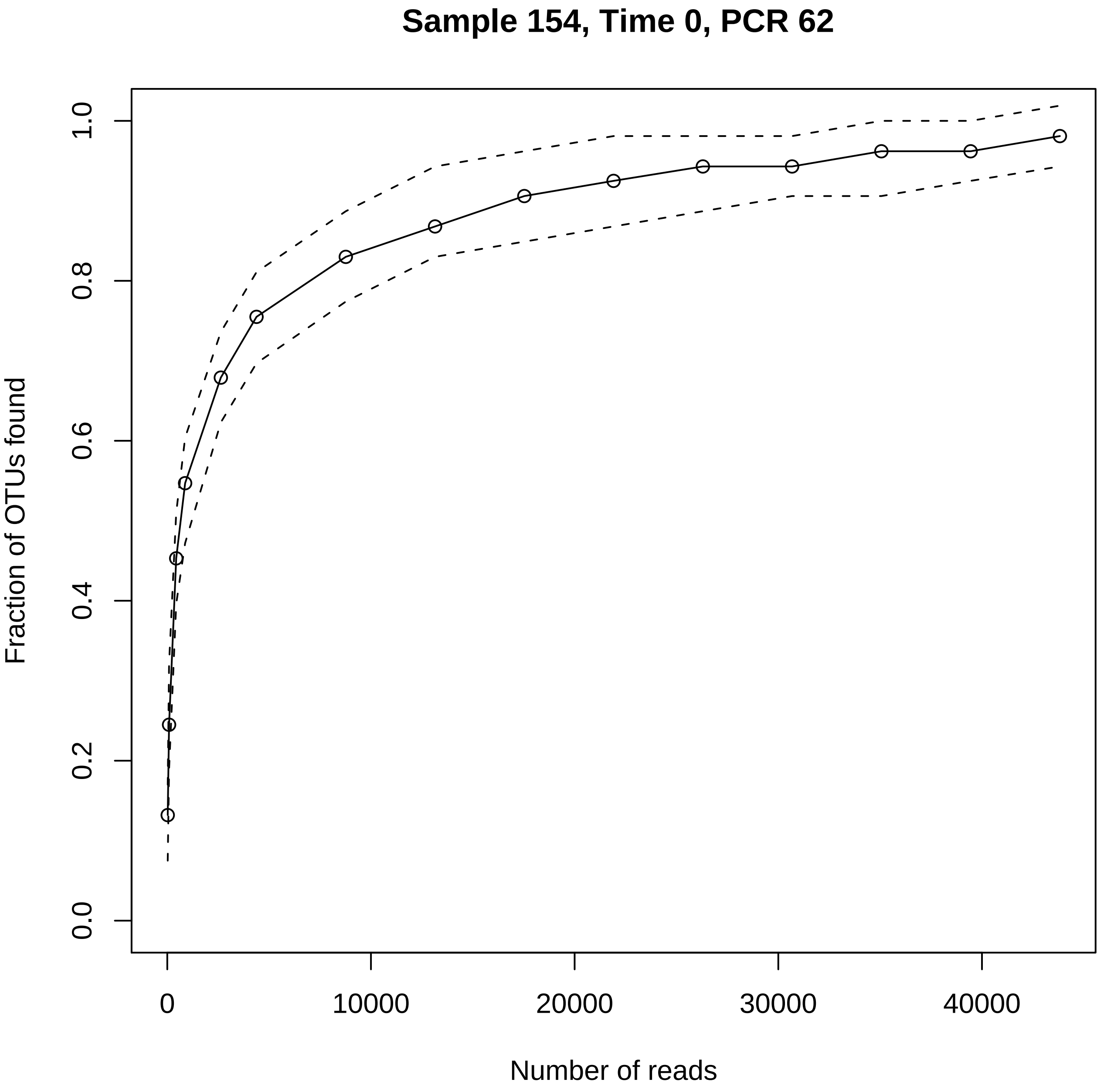
Sample 154, Time 0, PCR 62
Fraction of OTUs found
Number of reads
0.0
0.2
0.4
0.6
0.8
1.0
0
10000
20000
30000
40000

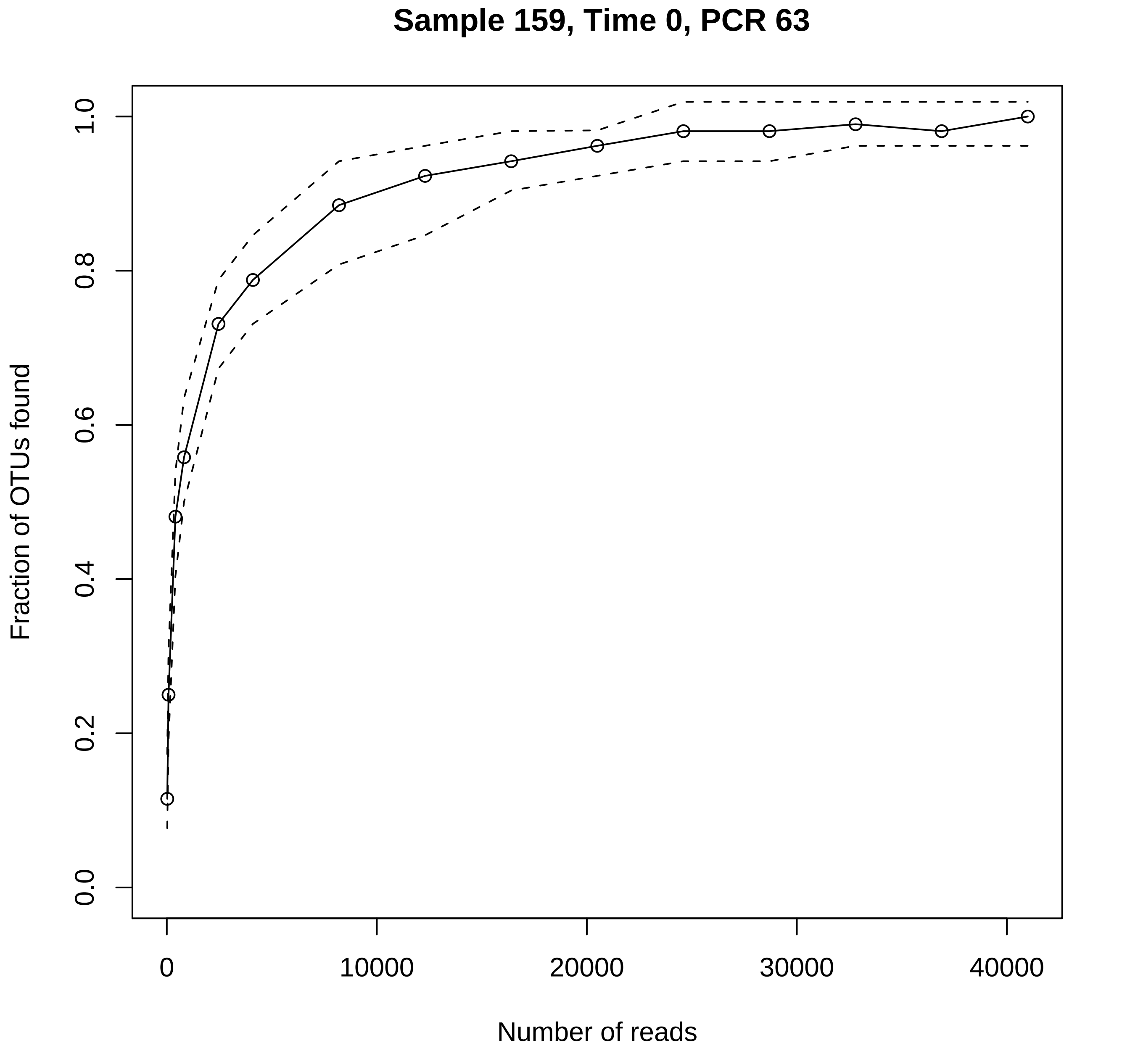

Sample 159, Time 0, PCR 63
Fraction of OTUs found
Number of reads
0.0
0.2
0.4
0.6
0.8
1.0
0
10000
20000
30000
40000

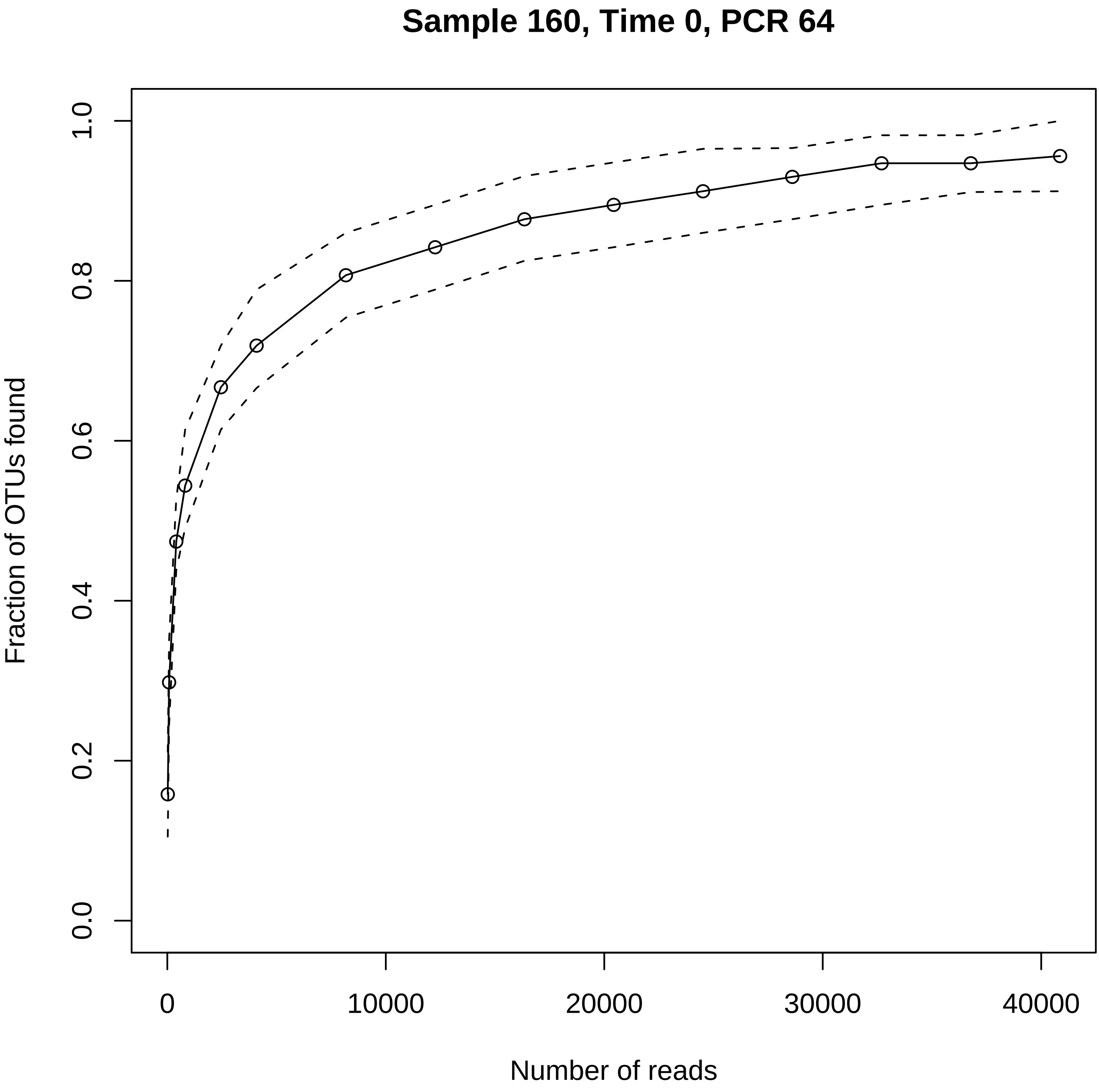

Sample 160, Time 0, PCR 64
Fraction of OTUs found
0.0
0.2
0.4
0.6
0.8
1.0
0
10000
20000
30000
40000
Number of reads

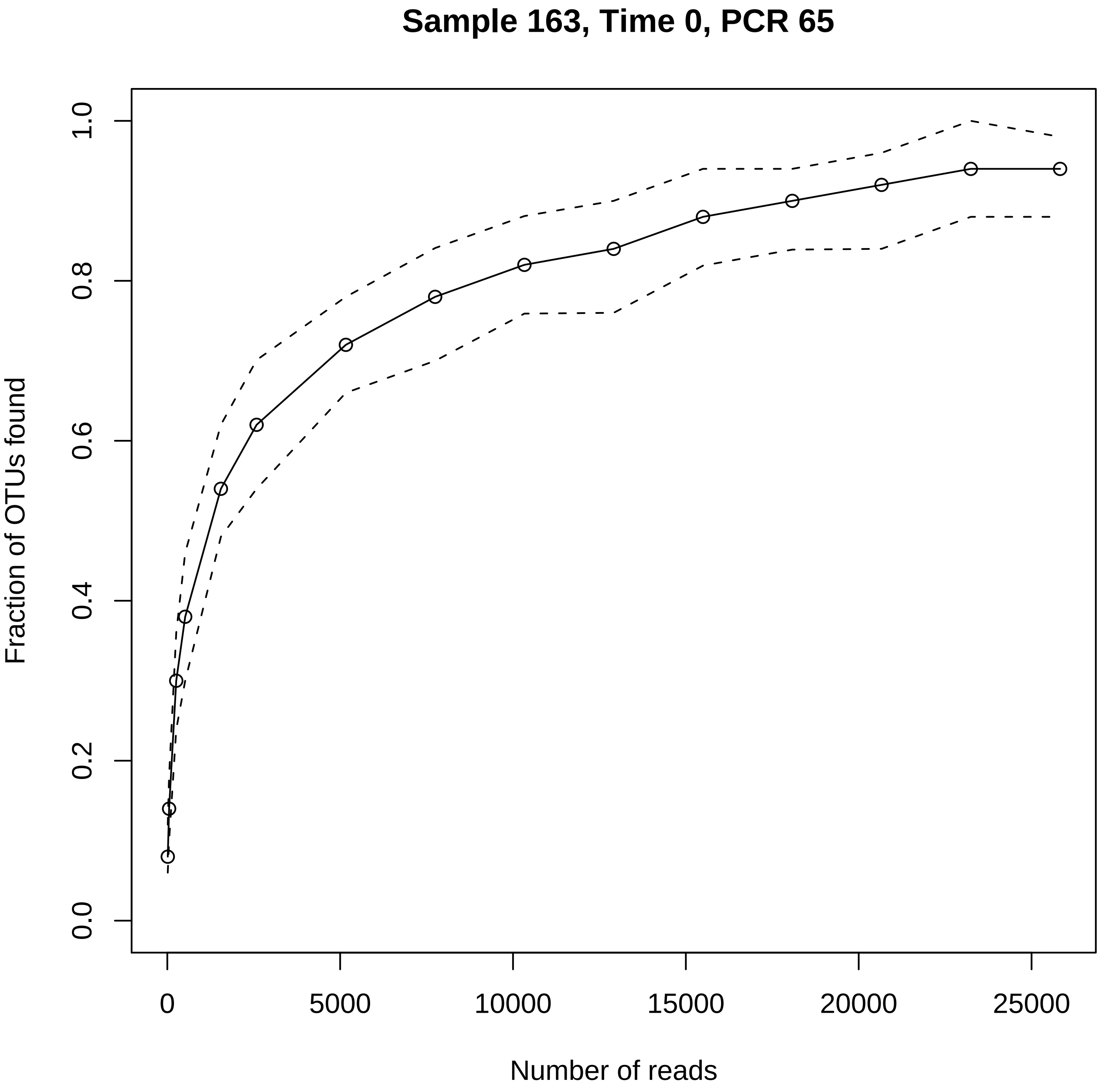

Sample 163, Time 0, PCR 65
Fraction of OTUs found
0.0
0.2
0.4
0.6
0.8
1.0
0
5000
10000
15000
20000
25000
Number of reads

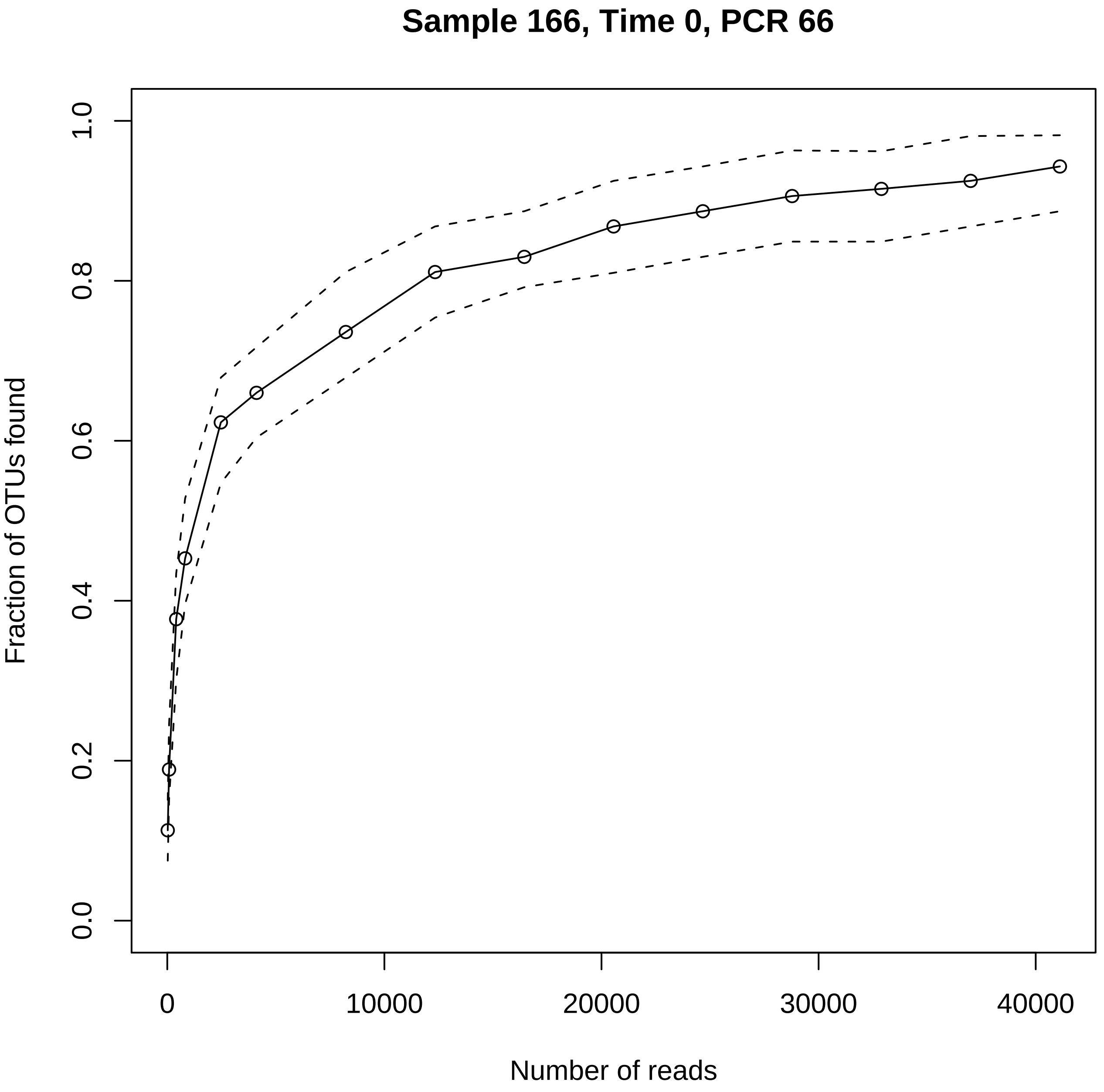
Sample 166, Time 0, PCR 66
Fraction of OTUs found
Number of reads
0.0
0.2
0.4
0.6
0.8
1.0
0
10000
20000
30000
40000

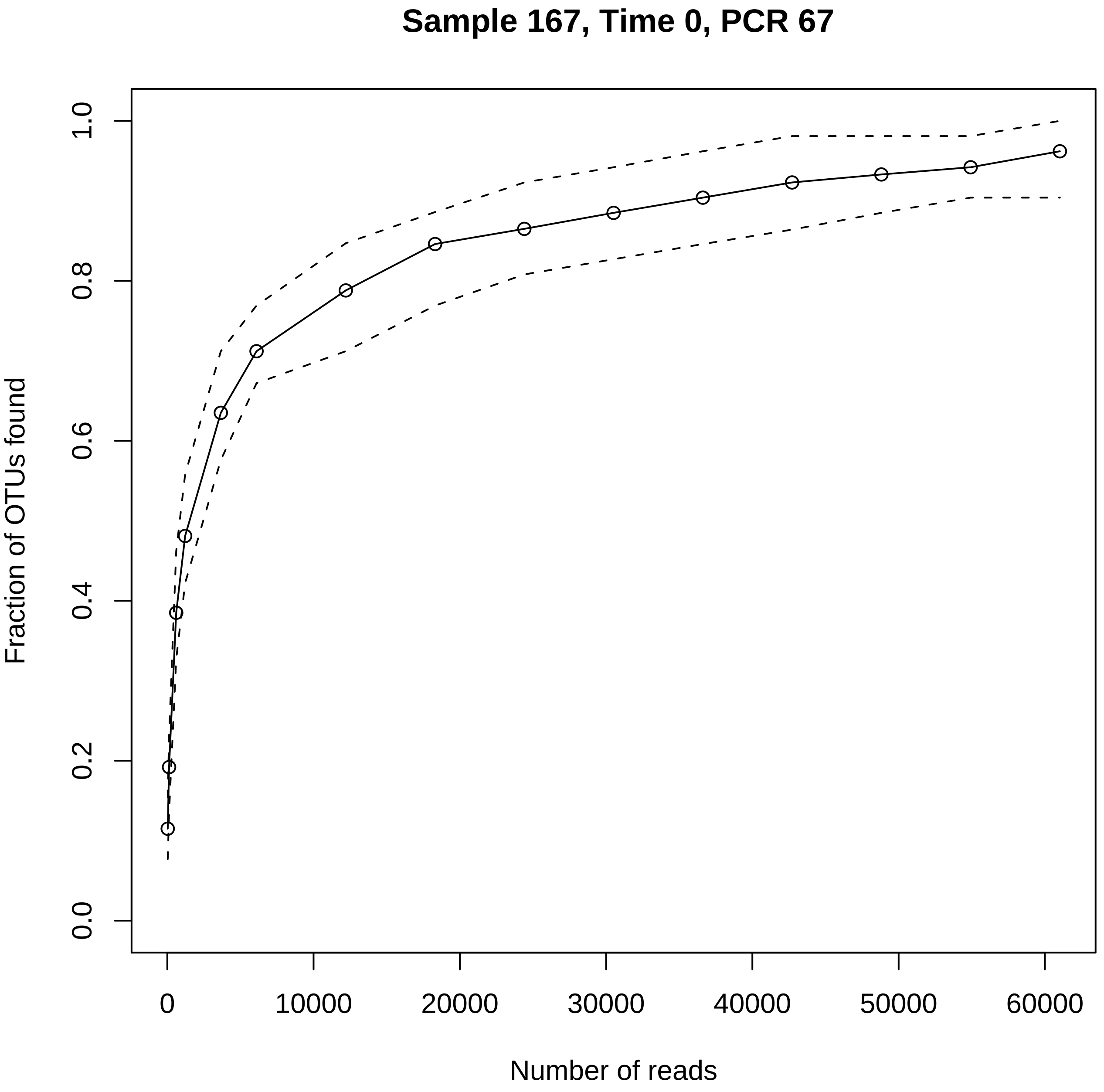

Sample 167, Time 0, PCR 67
Fraction of OTUs found
0.0
0.2
0.4
0.6
0.8
1.0
0
10000
20000
30000
40000
50000
60000
Number of reads

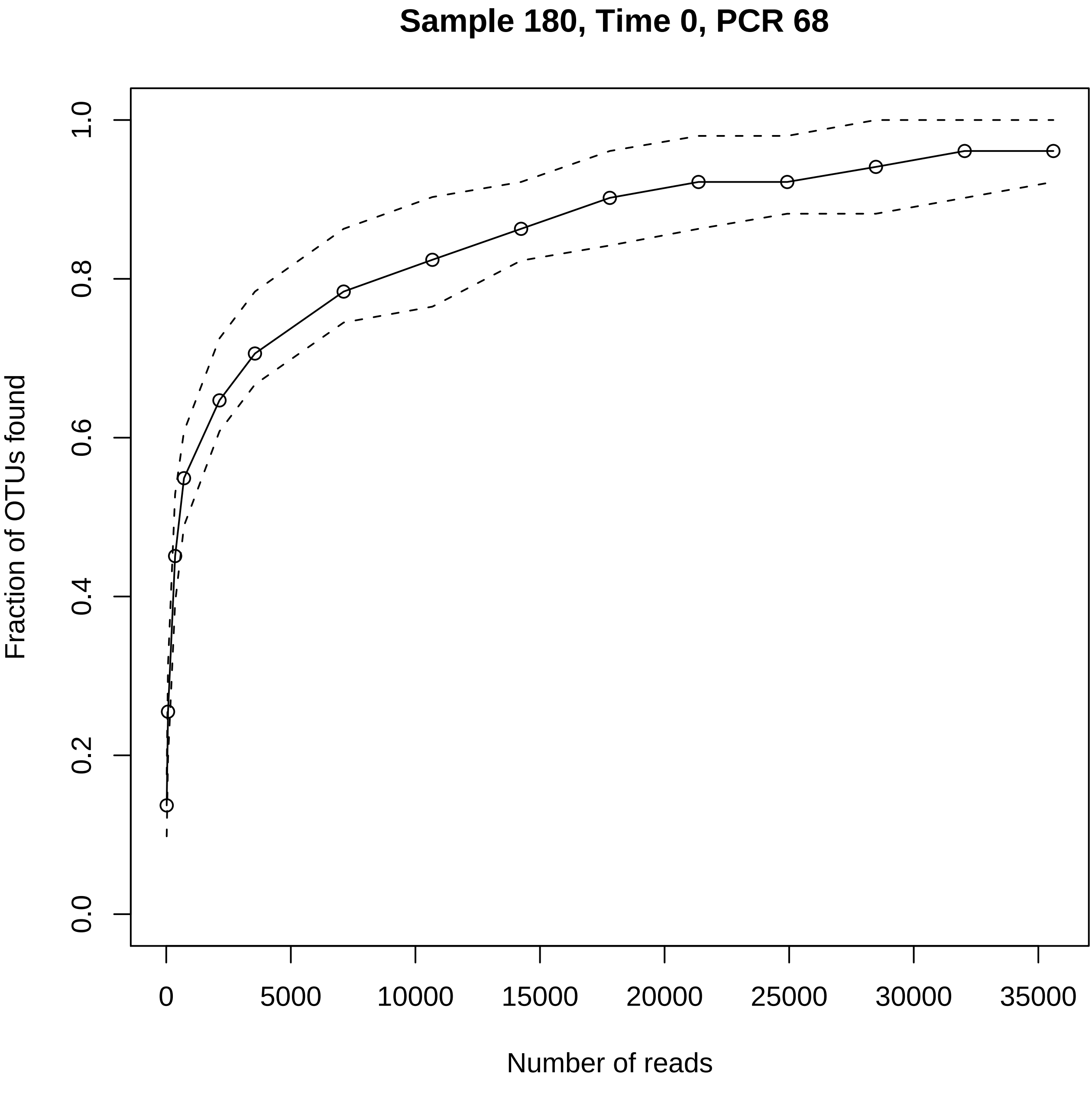
Sample 180, Time 0, PCR 68
Fraction of OTUs found
0.0
0.2
0.4
0.6
0.8
1.0
0
5000
10000
15000
20000
25000
30000
35000
Number of reads

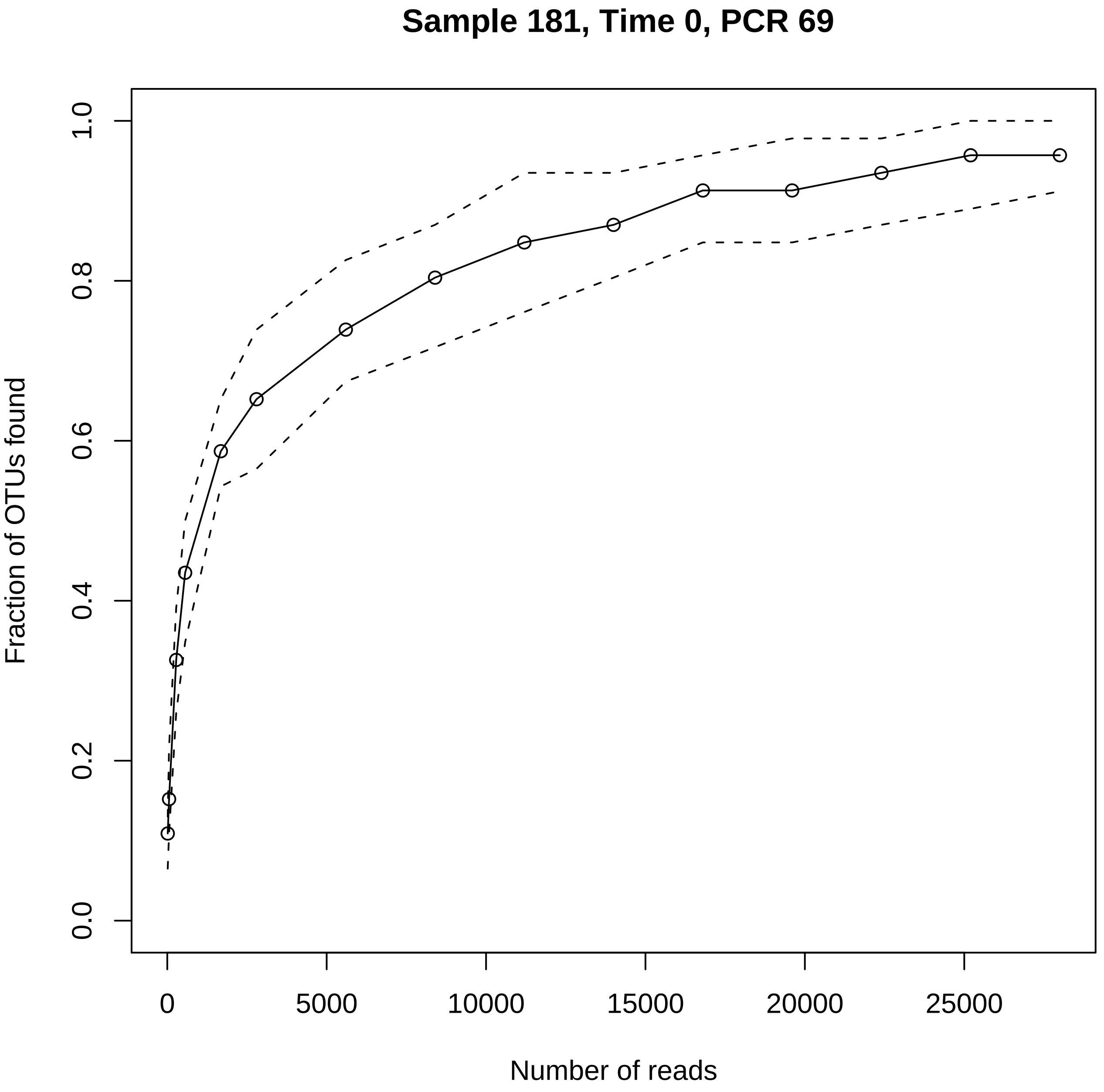

Sample 181, Time 0, PCR 69
Fraction of OTUs found
0.0
0.2
0.4
0.6
0.8
1.0
0
5000
10000
15000
20000
25000
Number of reads

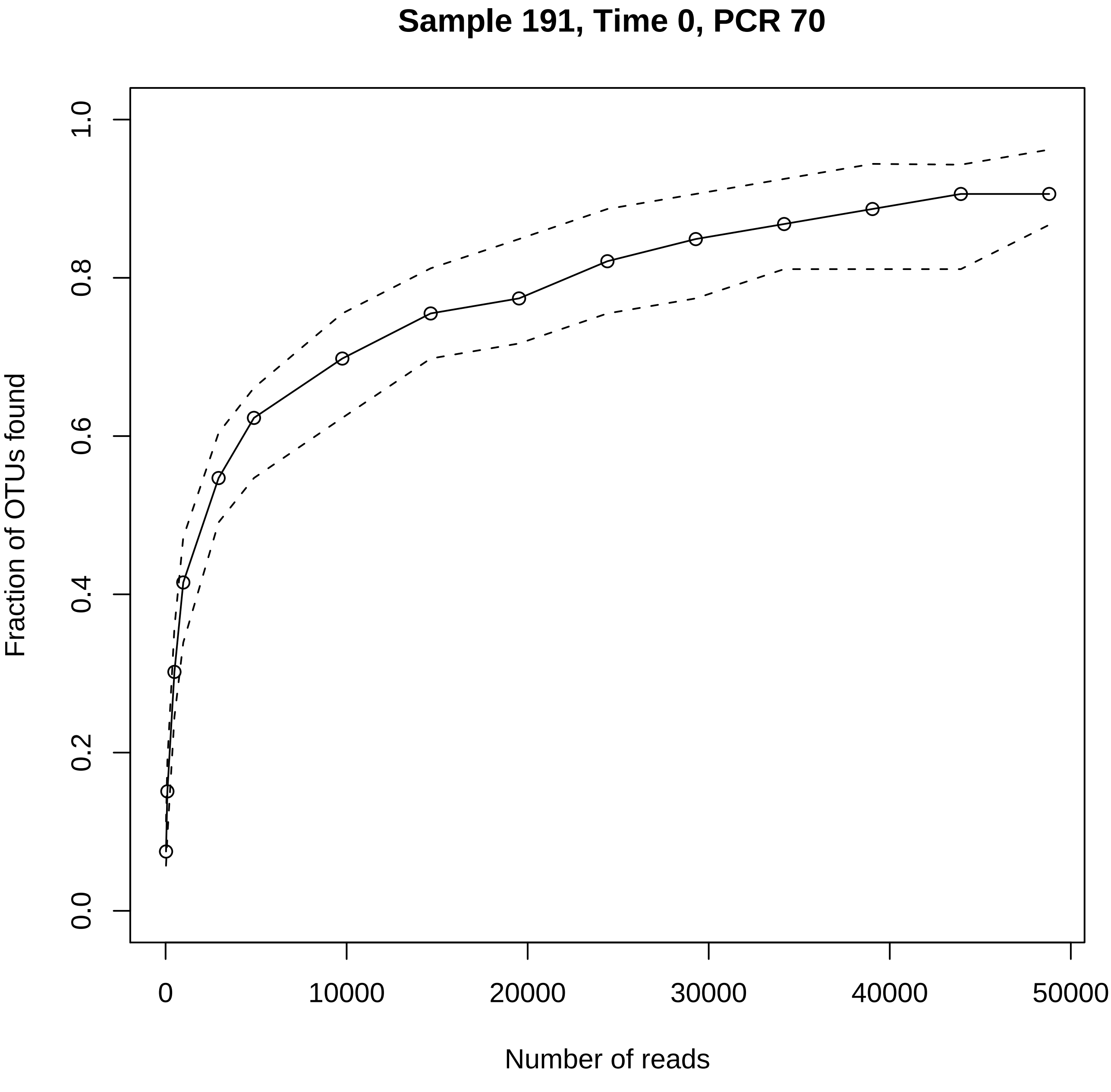
Sample 191, Time 0, PCR 70
Fraction of OTUs found
0.0
0.2
0.4
0.6
0.8
1.0
0
10000
20000
30000
40000
50000
Number of reads

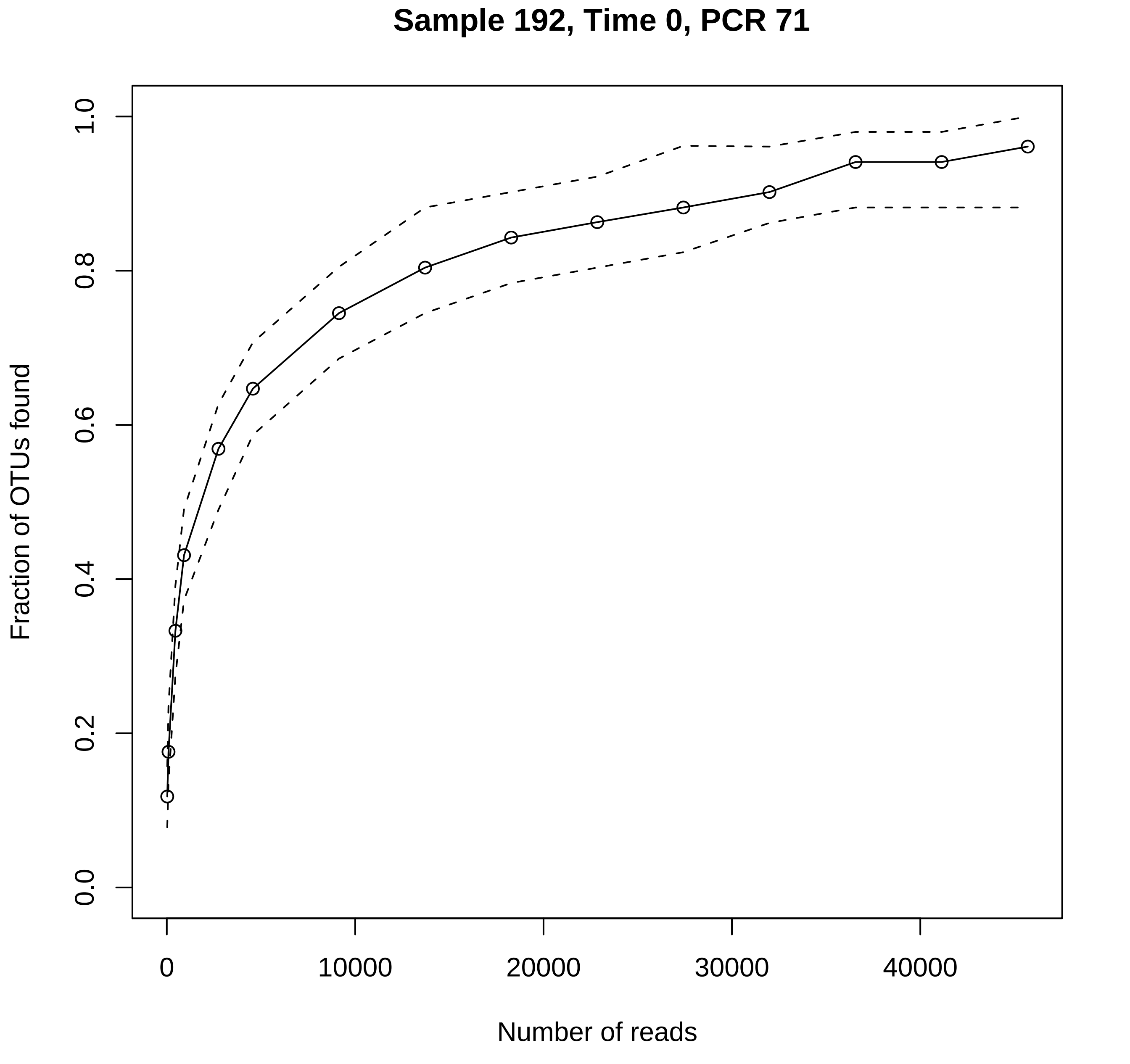
Sample 192, Time 0, PCR 71
Fraction of OTUs found
0.0
0.2
0.4
0.6
0.8
1.0
0
10000
20000
30000
40000
Number of reads

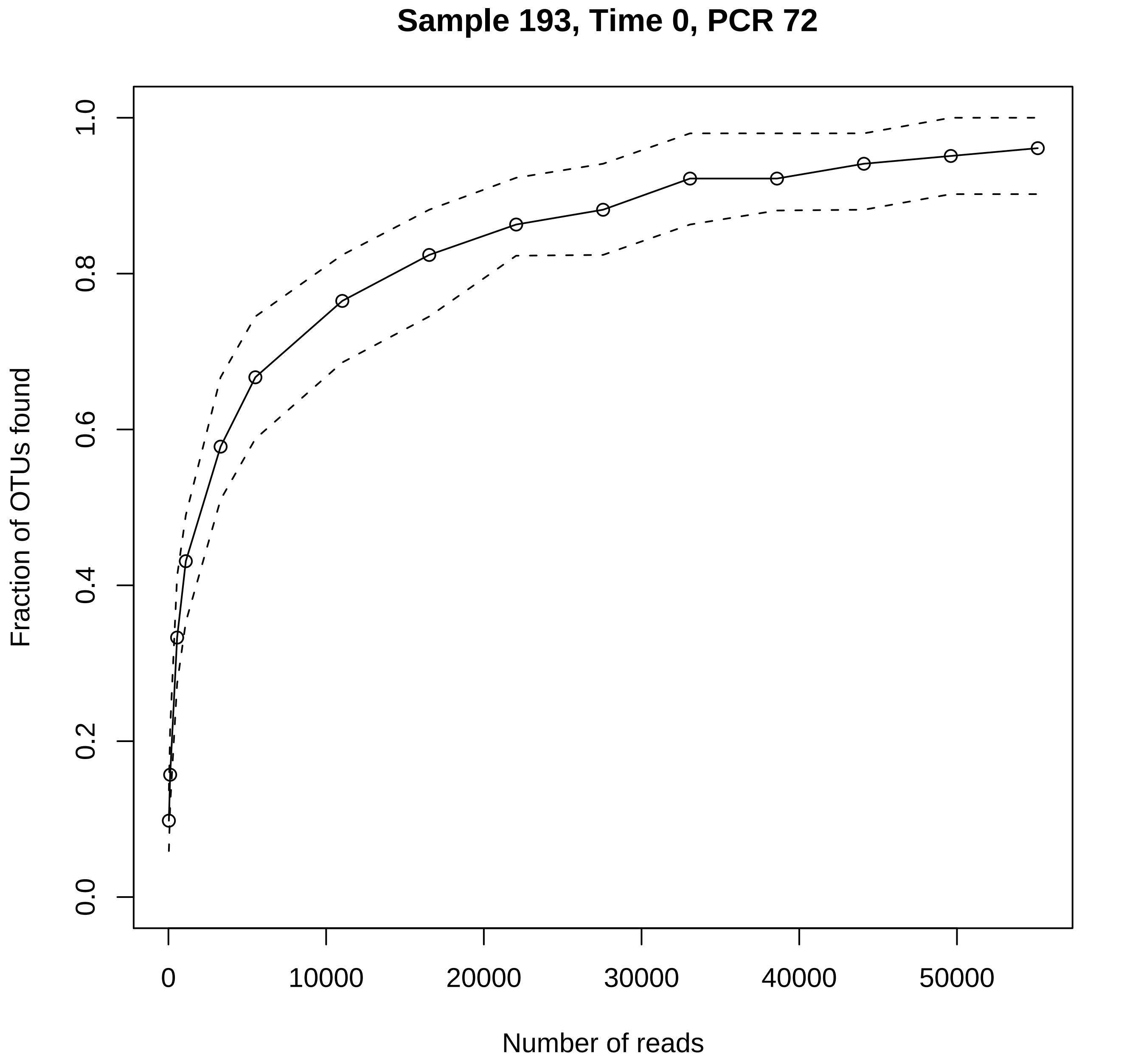
Sample 193, Time 0, PCR 72
Fraction of OTUs found
0.0
0.2
0.4
0.6
0.8
1.0
0
10000
20000
30000
40000
50000
Number of reads

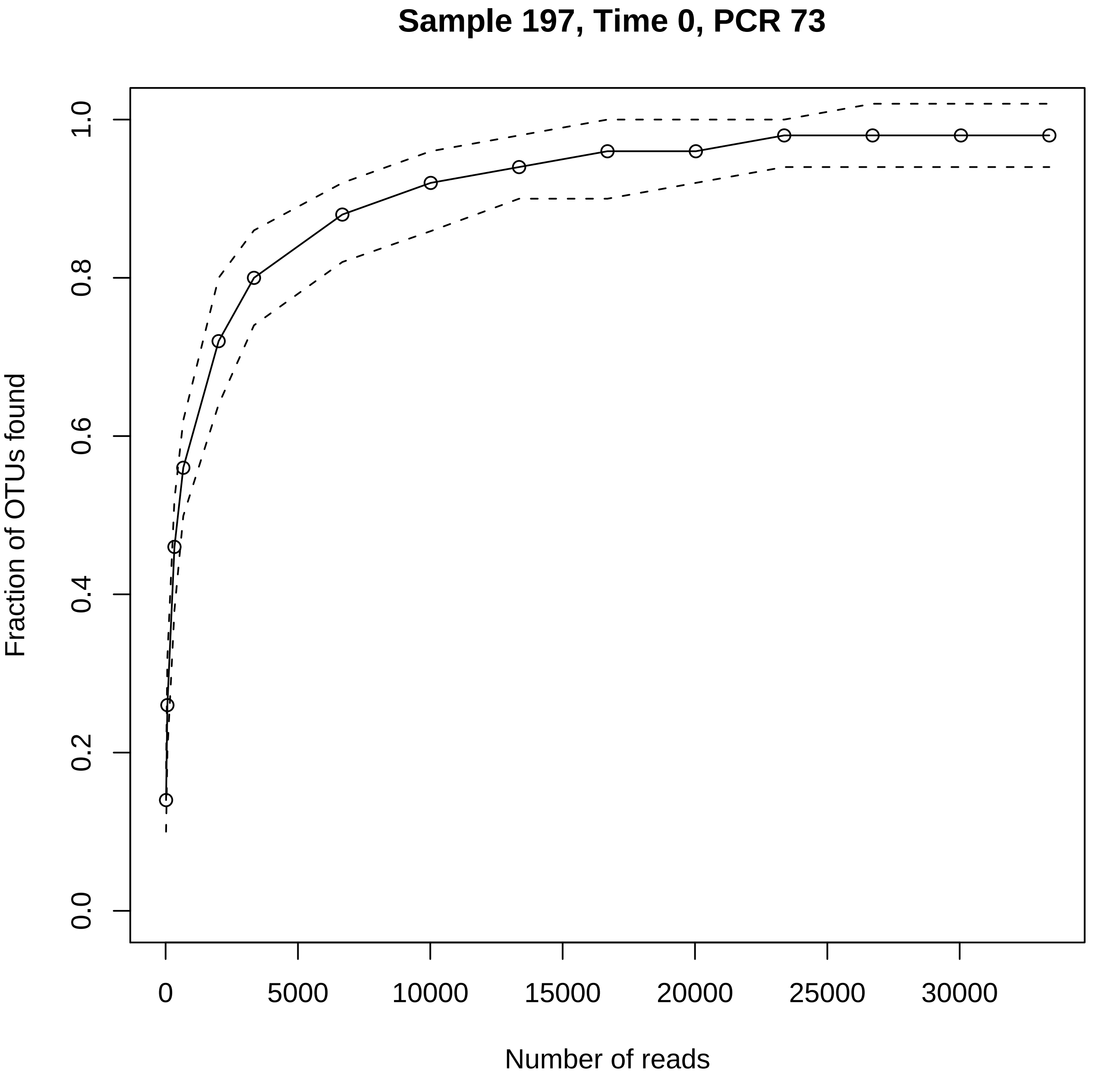

Sample 197, Time 0, PCR 73
Fraction of OTUs found
0.0
0.2
0.4
0.6
0.8
1.0
0
5000
10000
15000
20000
25000
30000
Number of reads

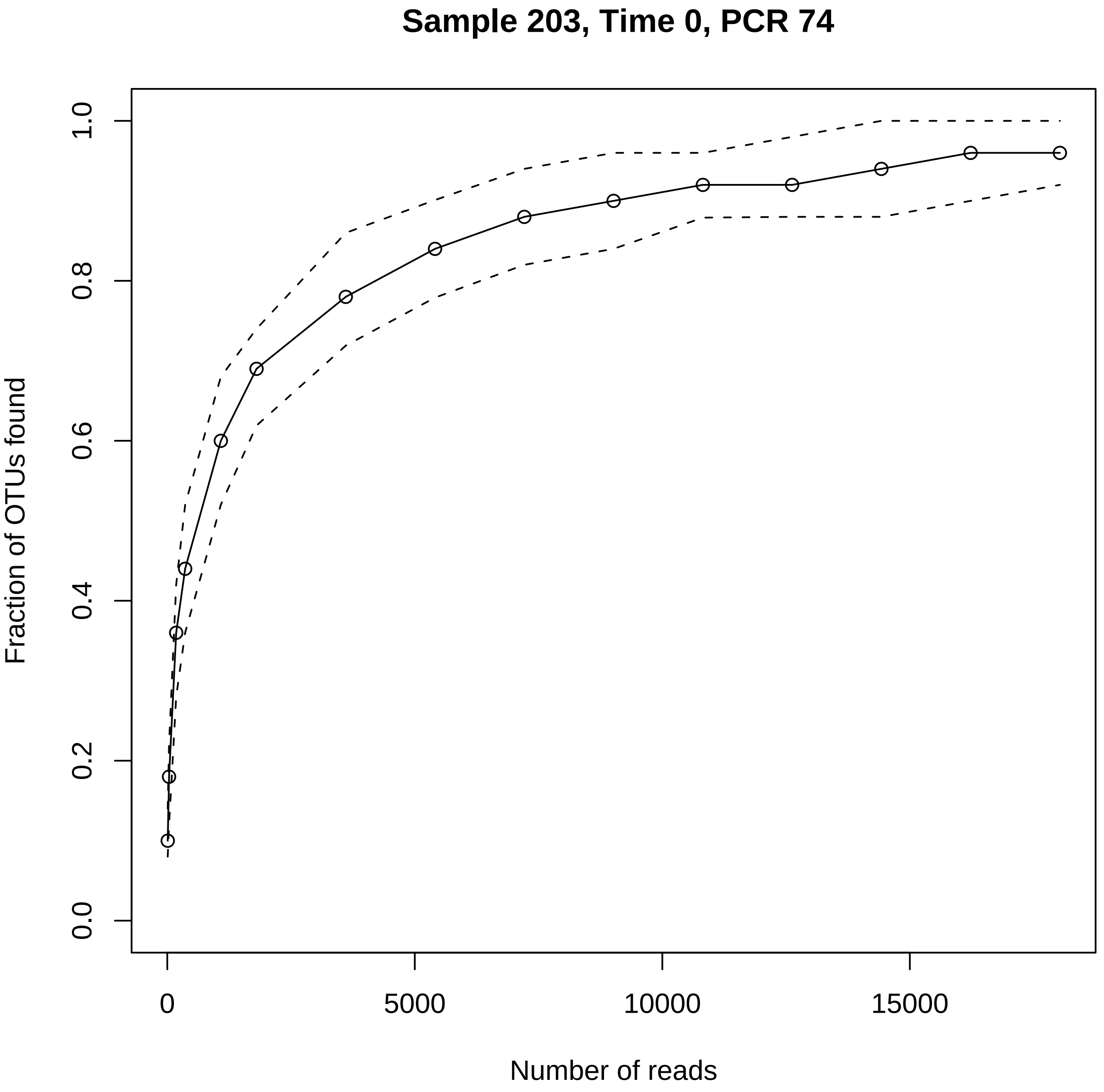
Sample 203, Time 0, PCR 74
Fraction of OTUs found
Number of reads
0.0
0.2
0.4
0.6
0.8
1.0
0
5000
10000
15000

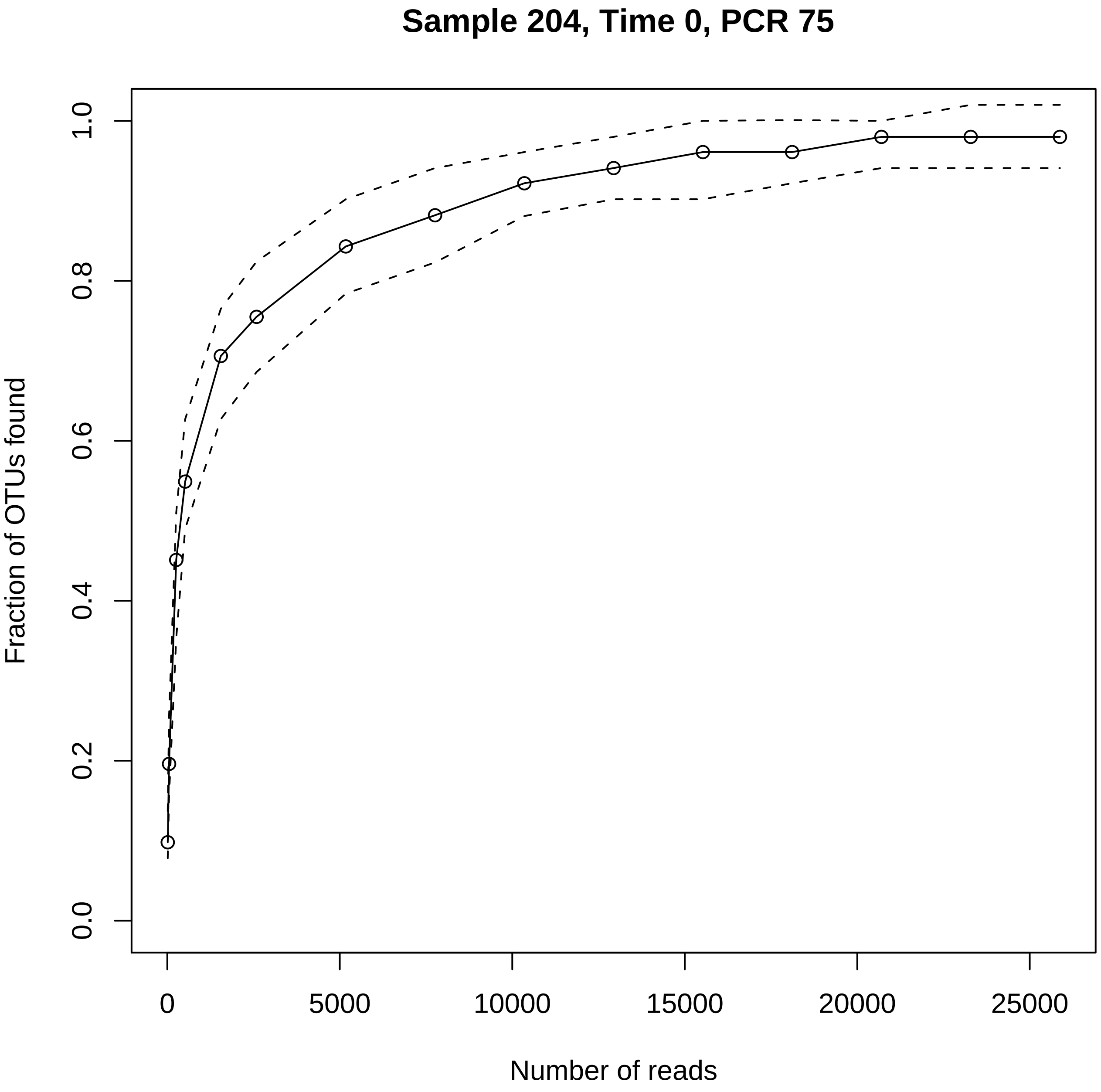
Sample 204, Time 0, PCR 75
Fraction of OTUs found
Number of reads
0.0
0.2
0.4
0.6
0.8
1.0
0
5000
10000
15000
20000
25000

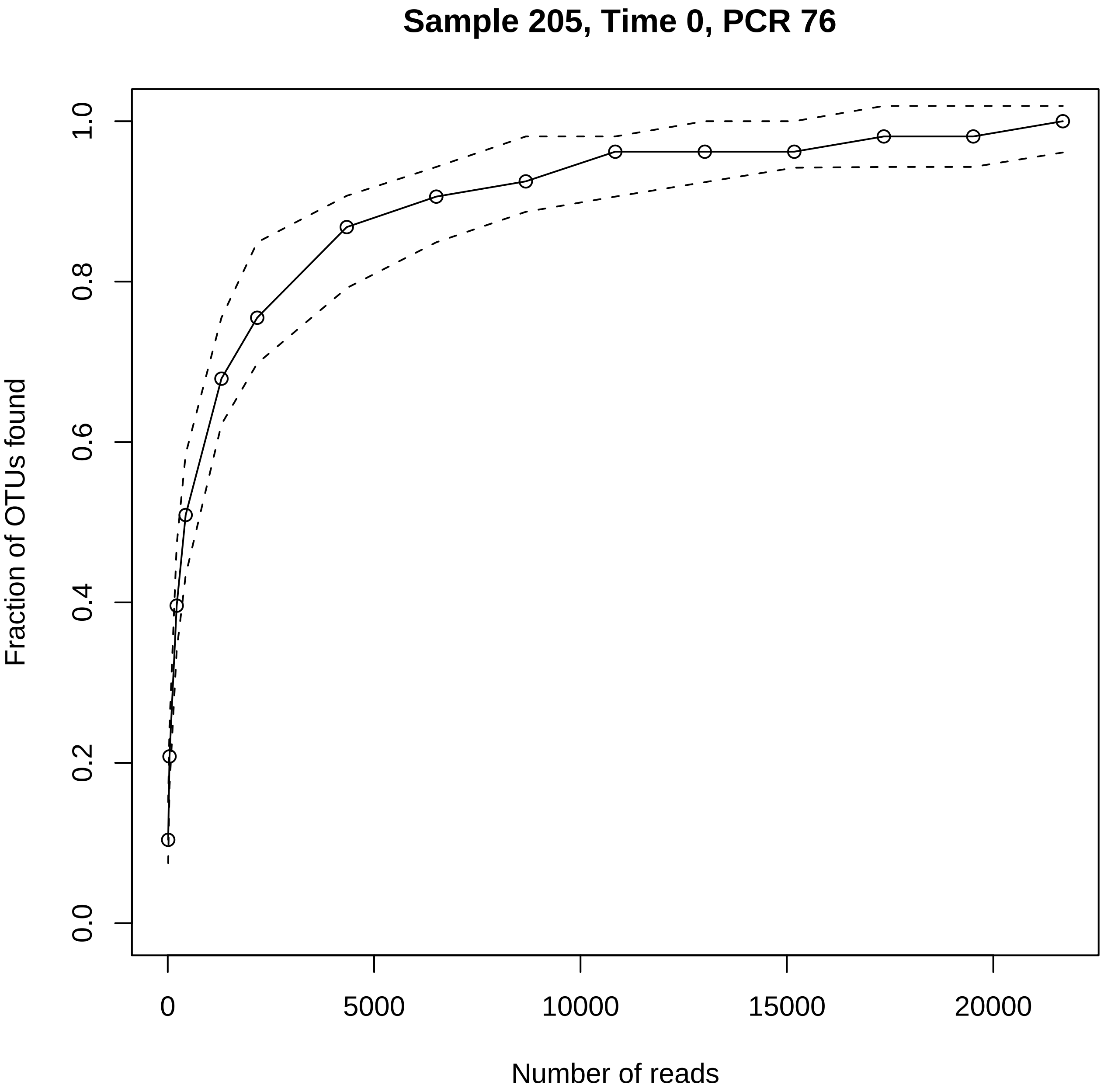

Sample 205, Time 0, PCR 76
Fraction of OTUs found
Number of reads
0.0
0.2
0.4
0.6
0.8
1.0
0
5000
10000
15000
20000

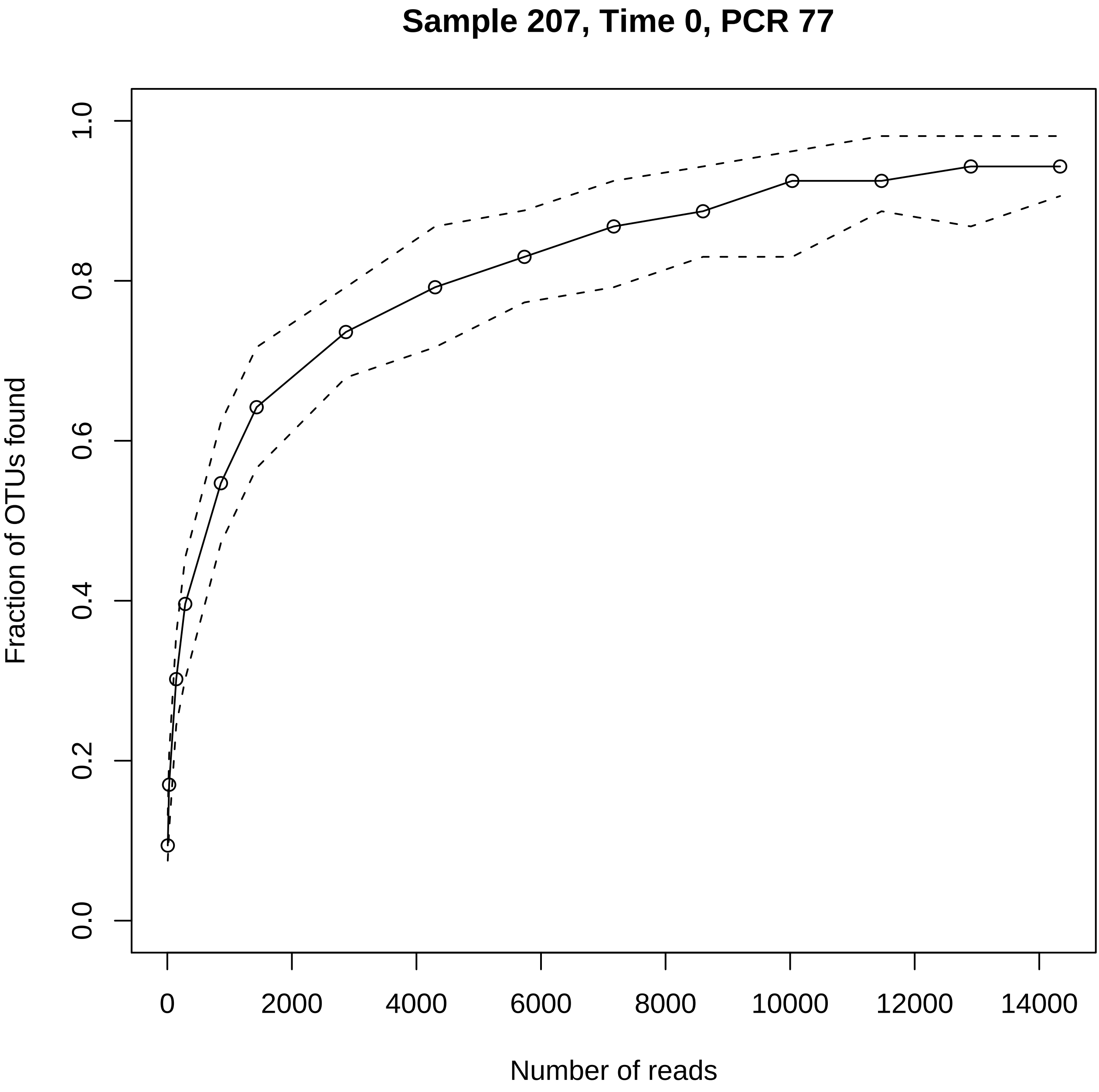

Sample 207, Time 0, PCR 77
Fraction of OTUs found
0.0
0.2
0.4
0.6
0.8
1.0
0
2000
4000
6000
8000
10000
12000
14000
Number of reads

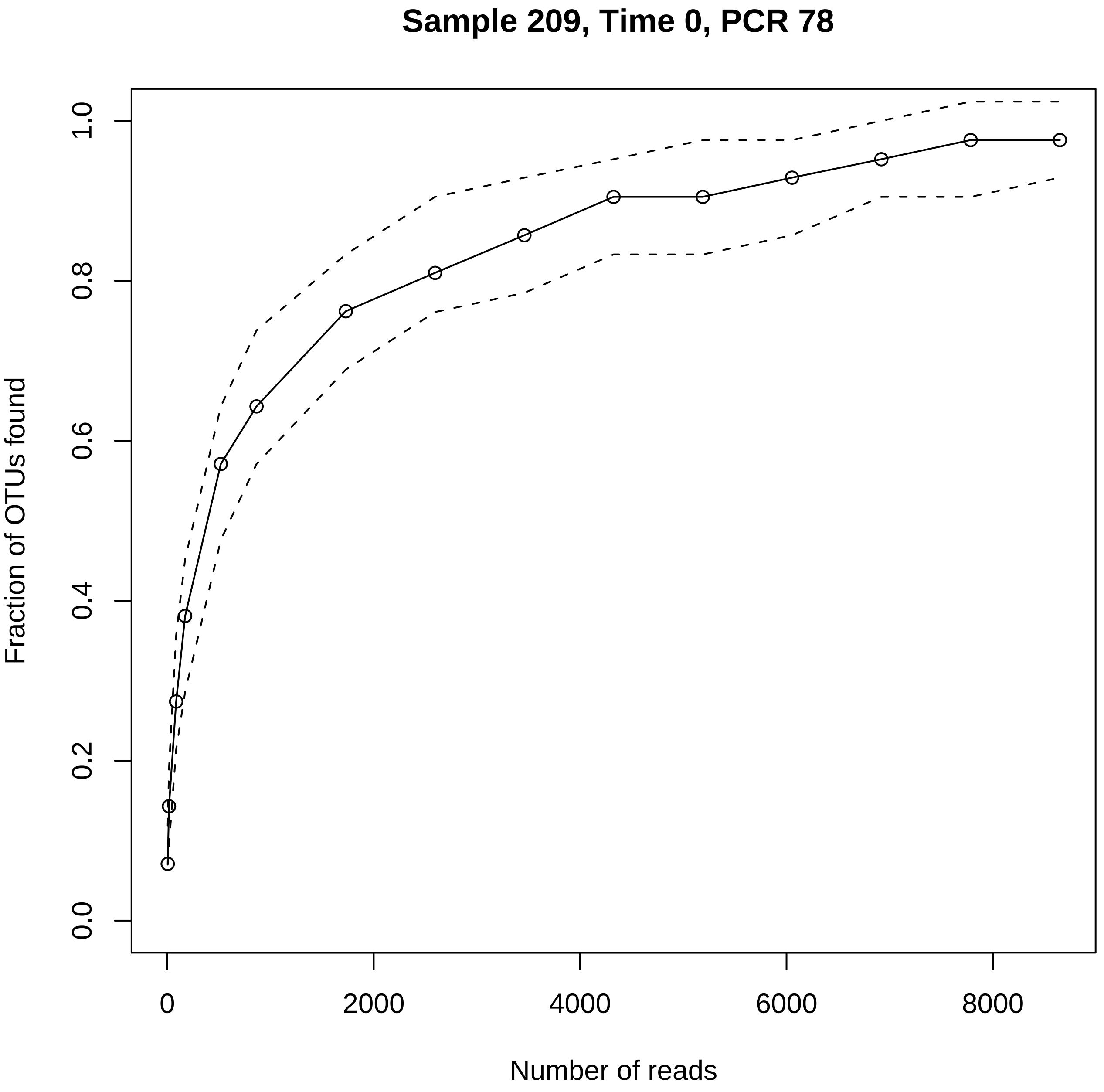

Sample 209, Time 0, PCR 78
Fraction of OTUs found
0.0
0.2
0.4
0.6
0.8
1.0
0
2000
4000
6000
8000
Number of reads

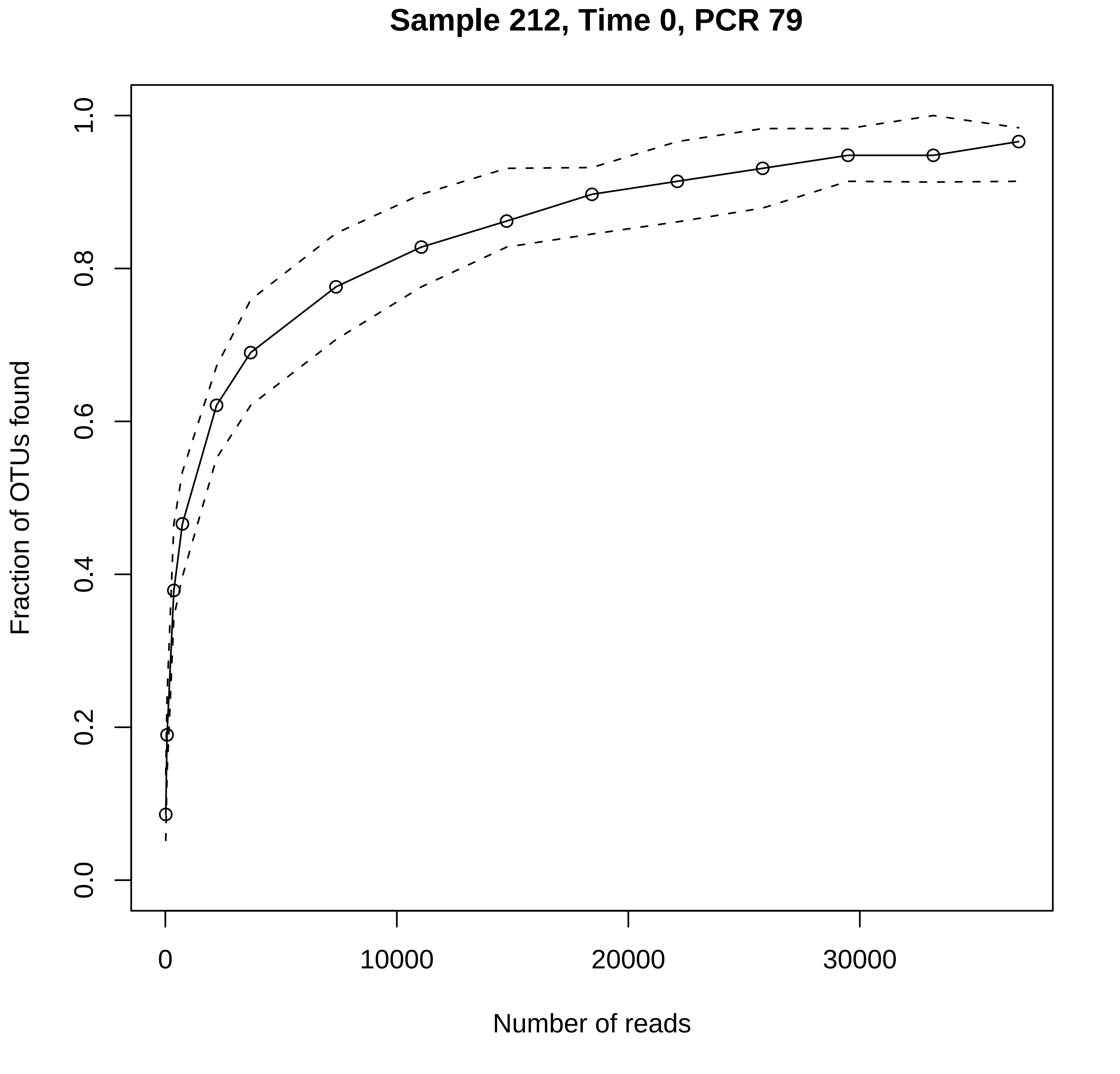

Sample 212, Time 0, PCR 79
Fraction of OTUs found
Number of reads
0.0
0.2
0.4
0.6
0.8
1.0
0
10000
20000
30000

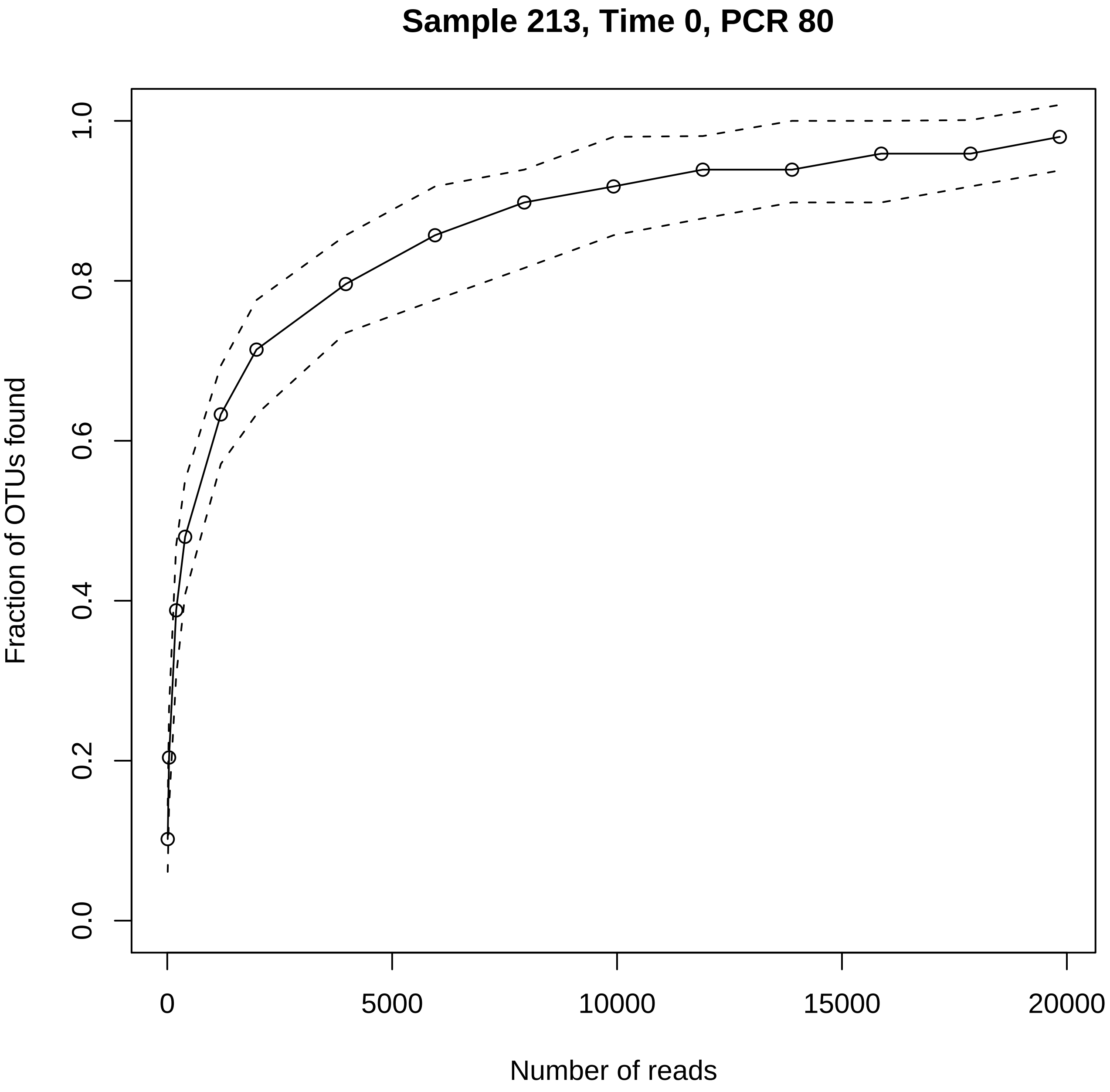

Sample 213, Time 0, PCR 80
Fraction of OTUs found
0.0
0.2
0.4
0.6
0.8
1.0
0
5000
10000
15000
20000
Number of reads

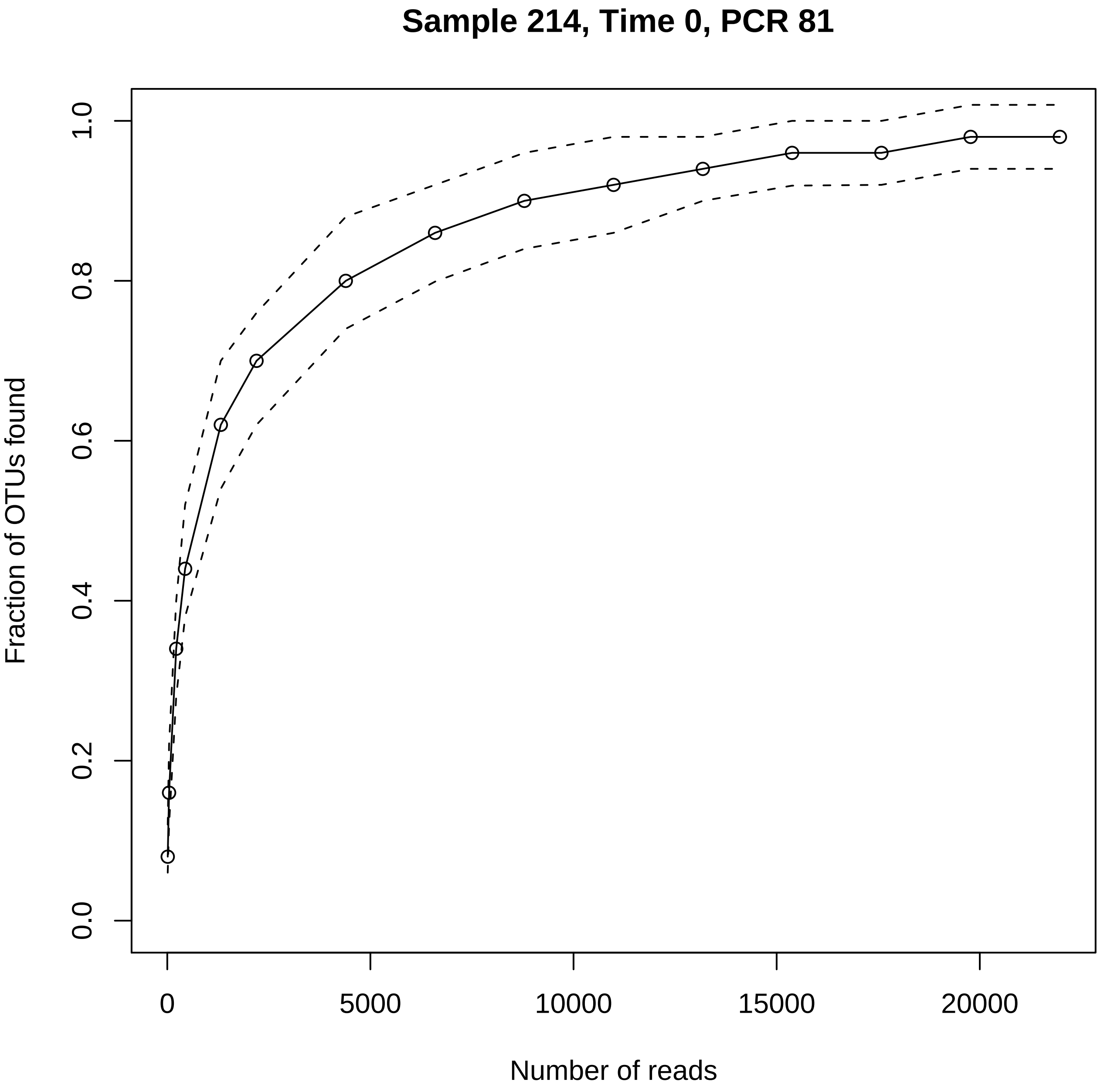
Sample 214, Time 0, PCR 81
Fraction of OTUs found
0.0
0.2
0.4
0.6
0.8
1.0
0
5000
10000
15000
20000
Number of reads

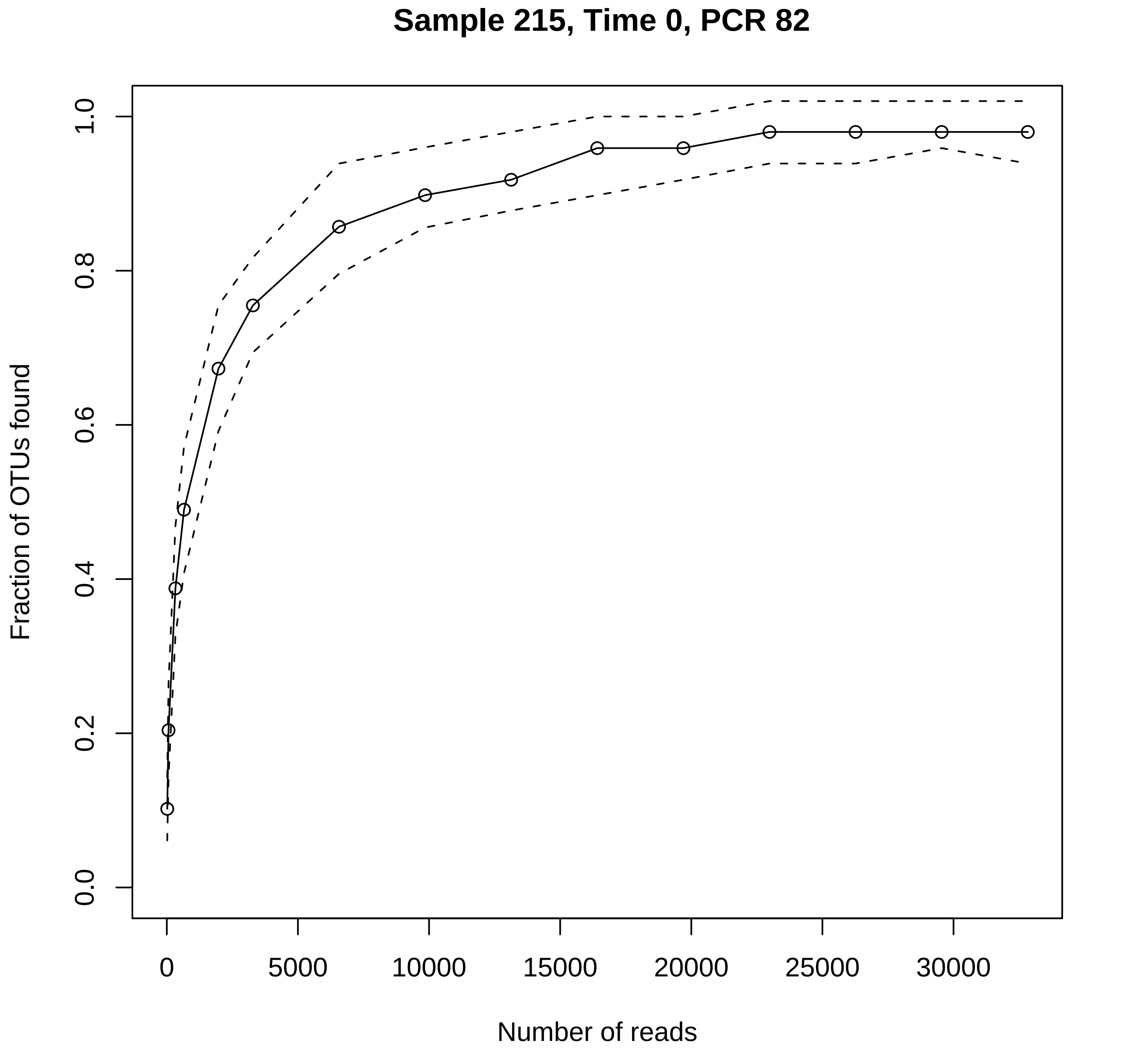

Sample 215, Time 0, PCR 82
Fraction of OTUs found
0.0
0.2
0.4
0.6
0.8
1.0
0
5000
10000
15000
20000
25000
30000
Number of reads

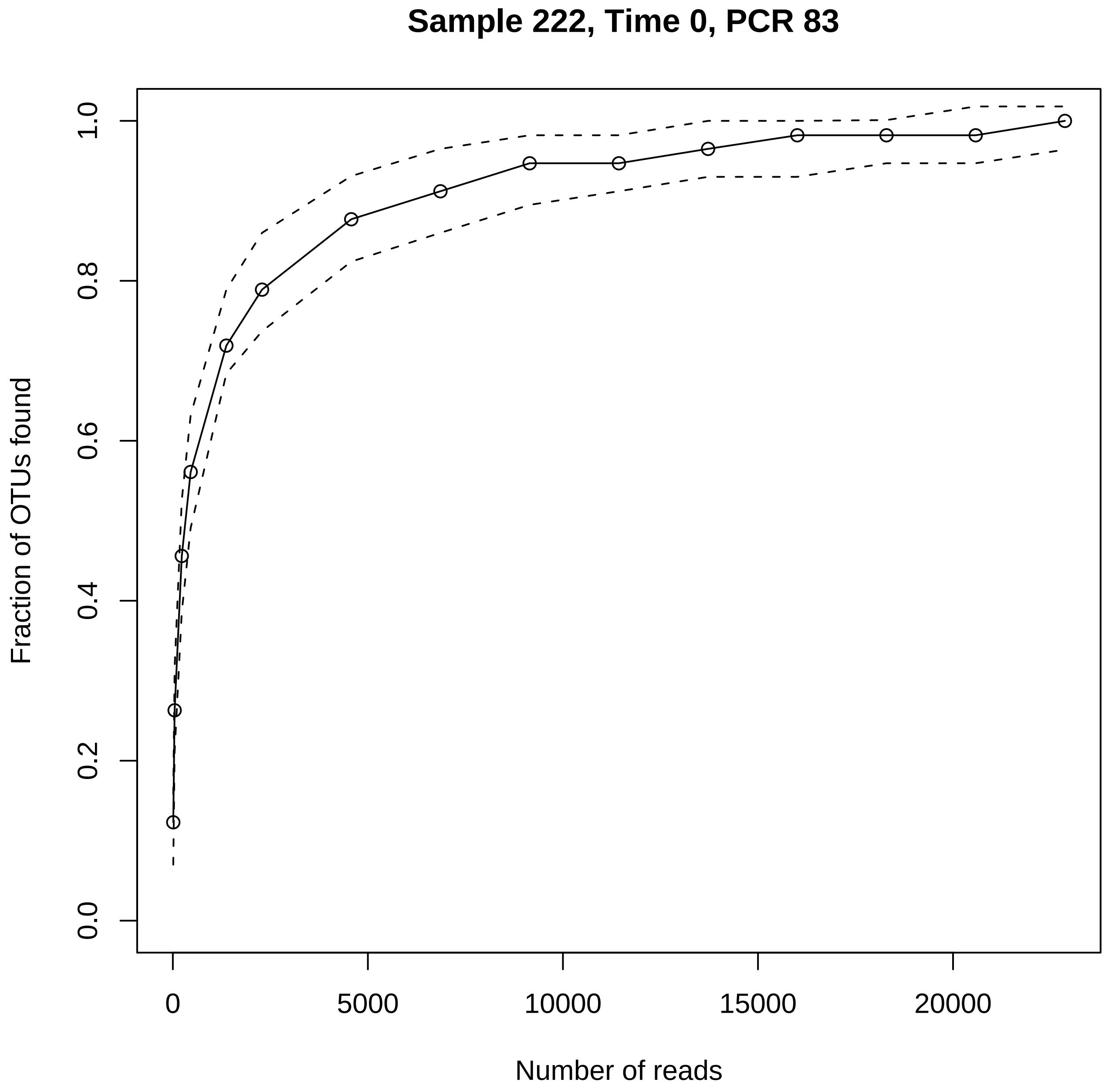

Sample 222, Time 0, PCR 83
Fraction of OTUs found
0.0
0.2
0.4
0.6
0.8
1.0
0
5000
10000
15000
20000
Number of reads

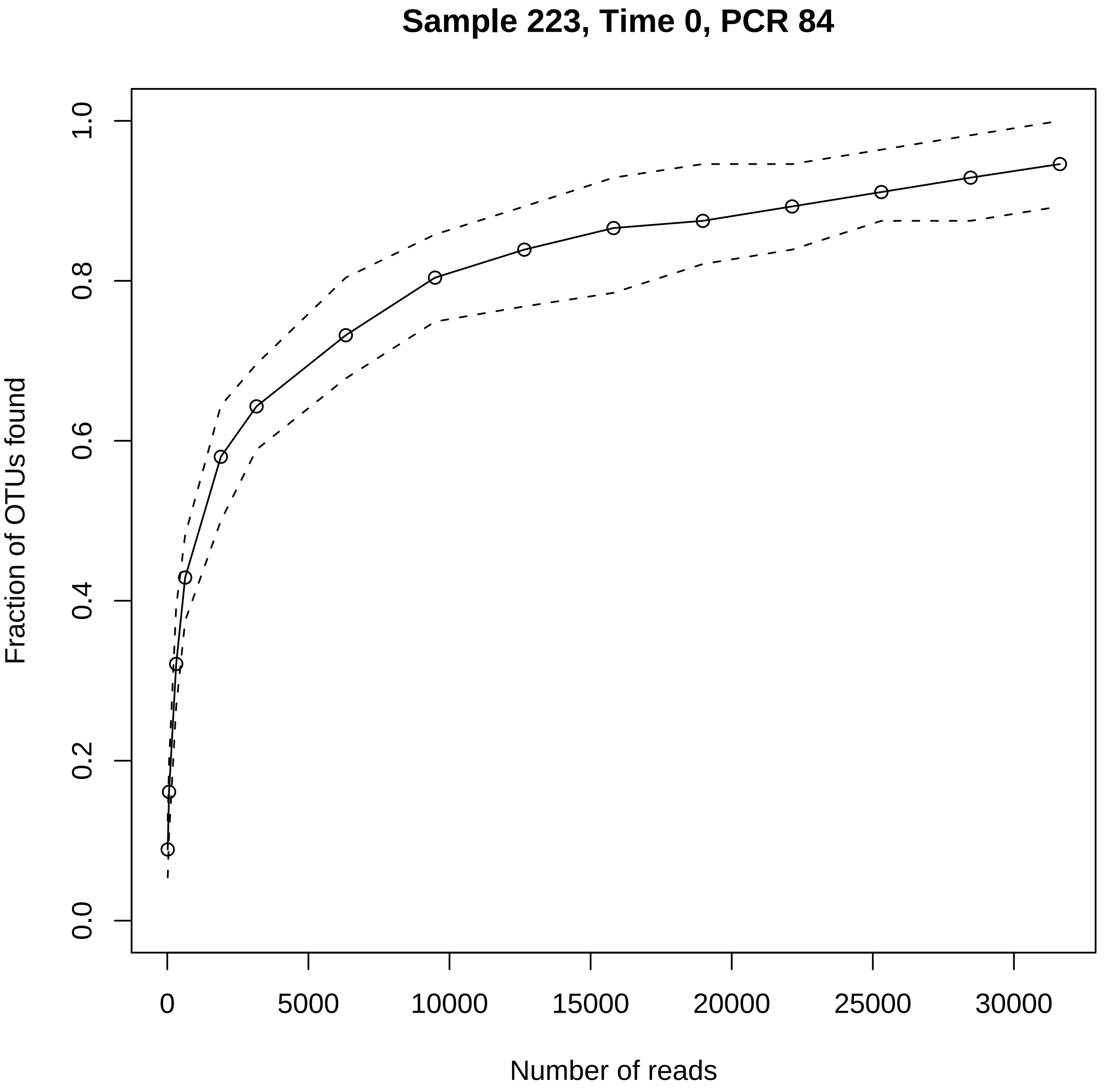
Sample 223, Time 0, PCR 84
Fraction of OTUs found
0.0
0.2
0.4
0.6
0.8
1.0
0
5000
10000
15000
20000
25000
30000
Number of reads

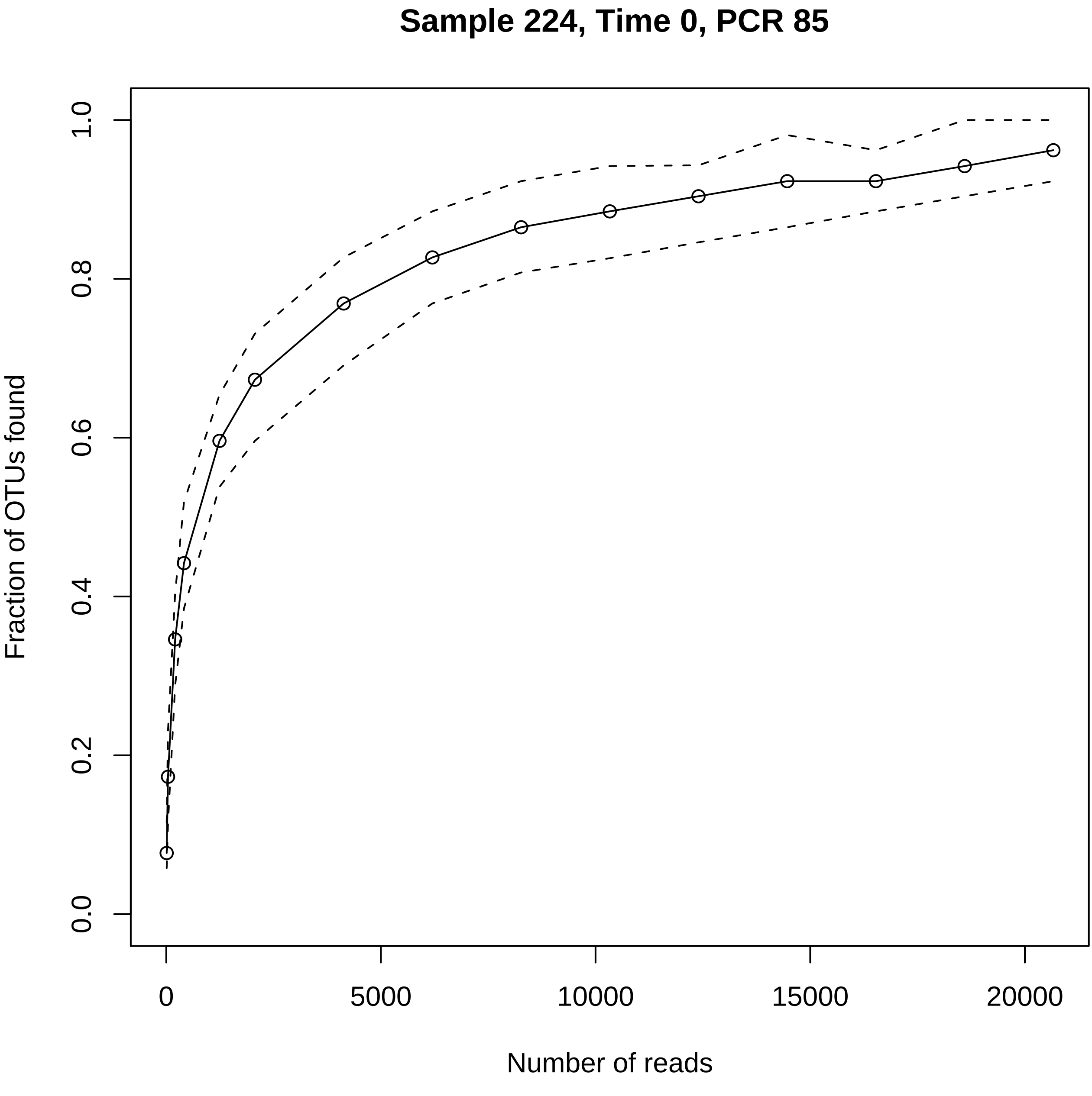

Sample 224, Time 0, PCR 85
Fraction of OTUs found
Number of reads
0.0
0.2
0.4
0.6
0.8
1.0
0
5000
10000
15000
20000

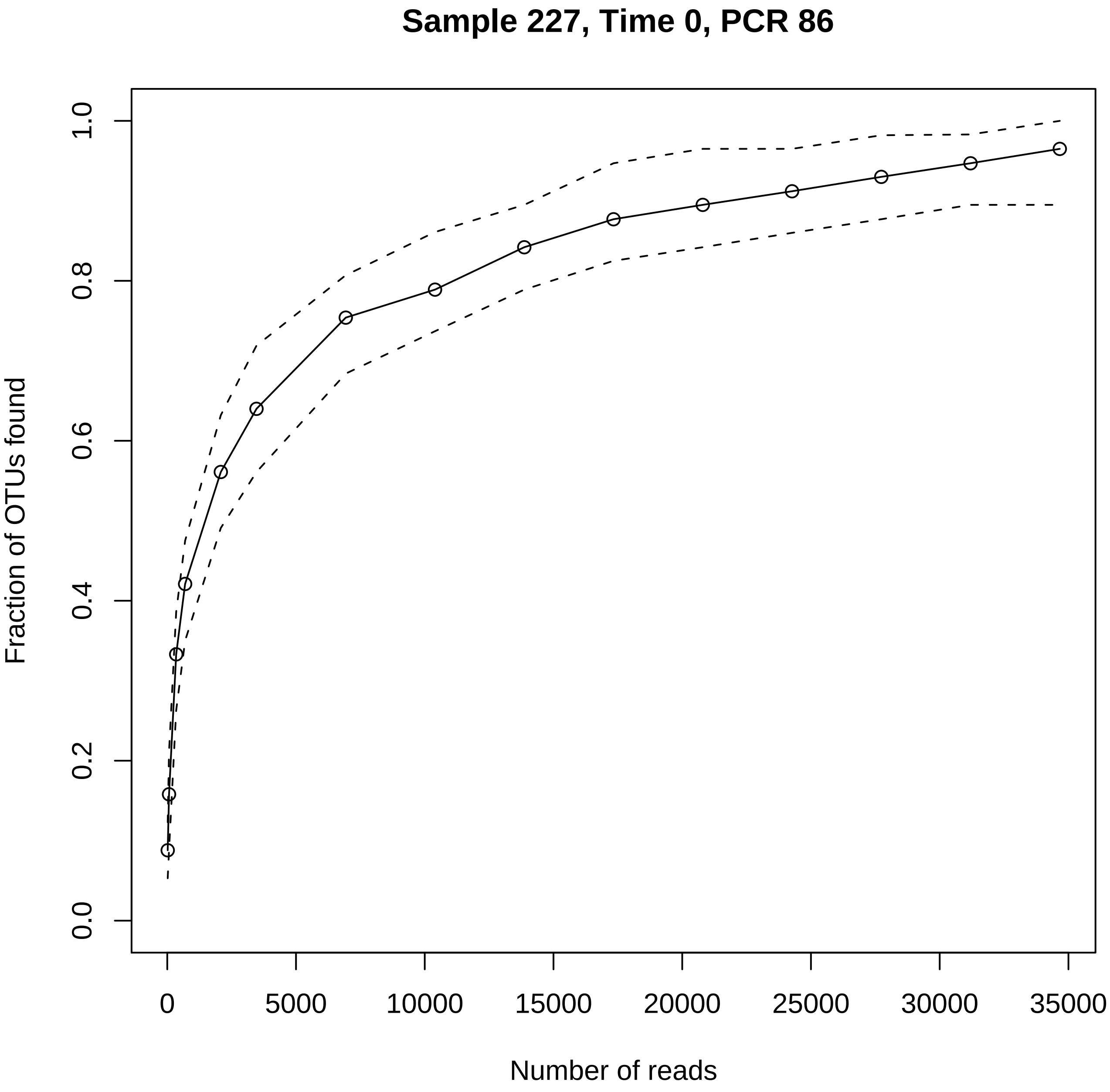
Sample 227, Time 0, PCR 86
Fraction of OTUs found
0.0
0.2
0.4
0.6
0.8
1.0
0
5000
10000
15000
20000
25000
30000
35000
Number of reads

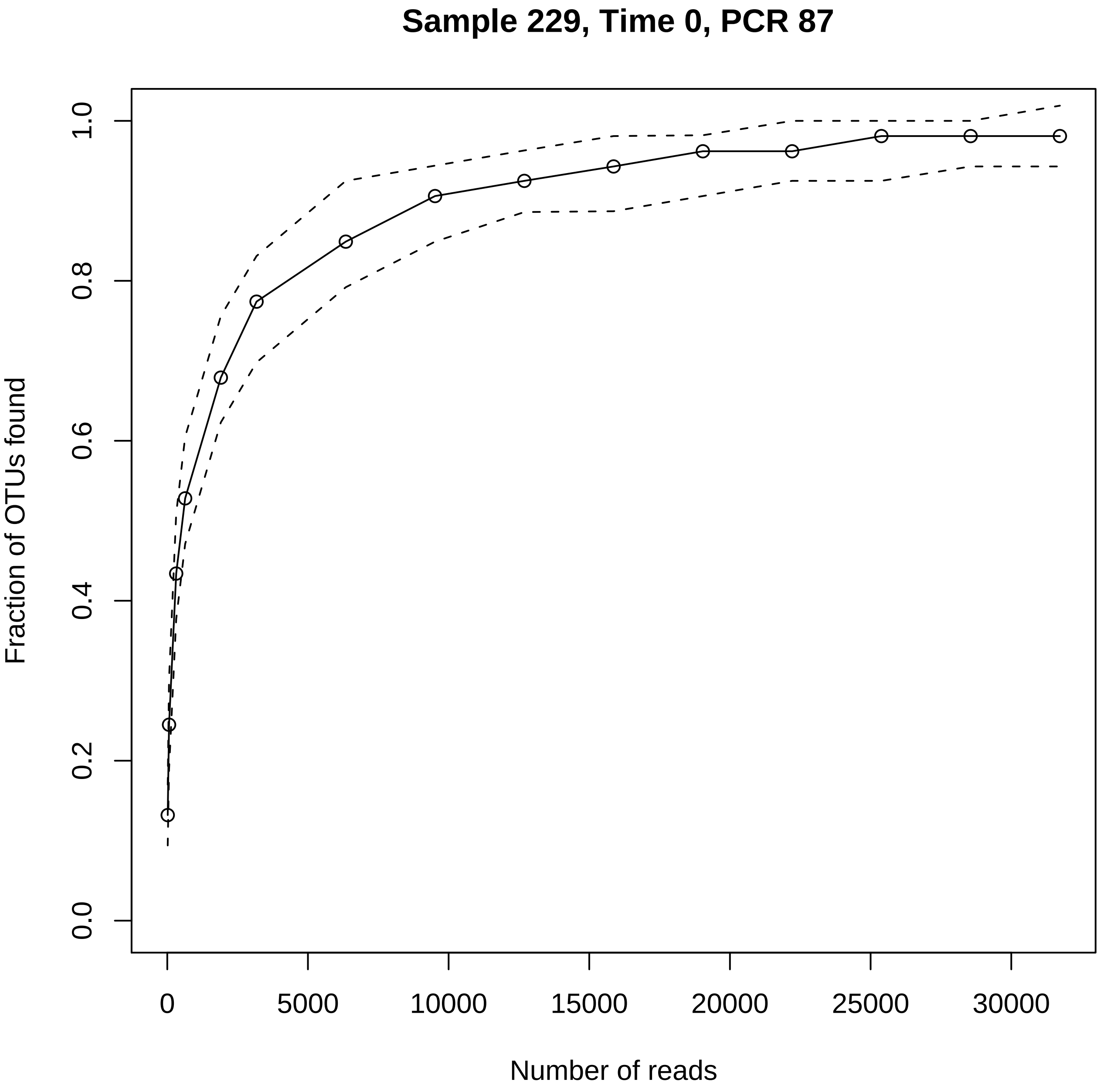
Sample 229, Time 0, PCR 87
Fraction of OTUs found
0.0
0.2
0.4
0.6
0.8
1.0
0
5000
10000
15000
20000
25000
30000
Number of reads

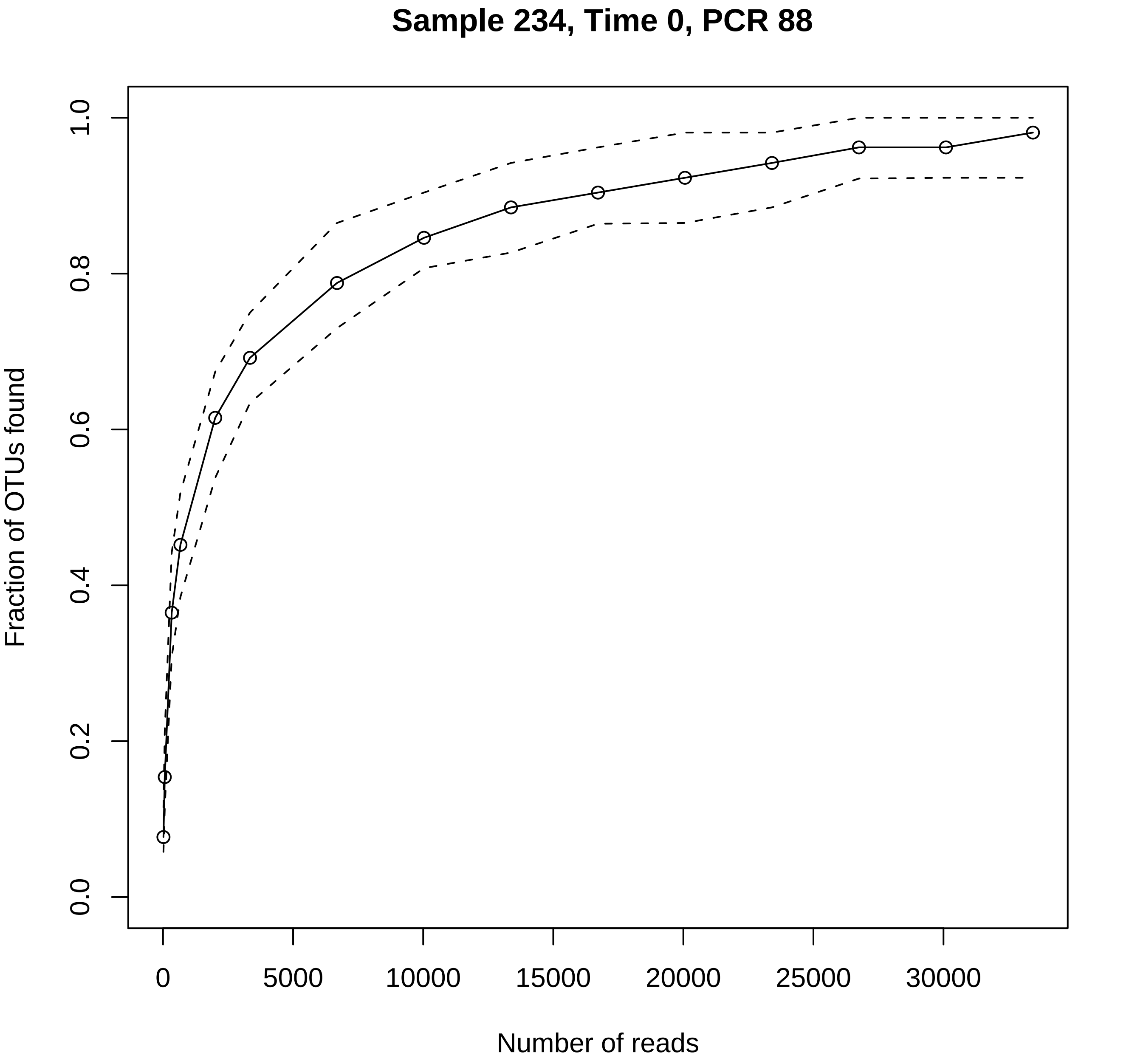

Sample 234, Time 0, PCR 88
Fraction of OTUs found
0.0
0.2
0.4
0.6
0.8
1.0
0
5000
10000
15000
20000
25000
30000
Number of reads

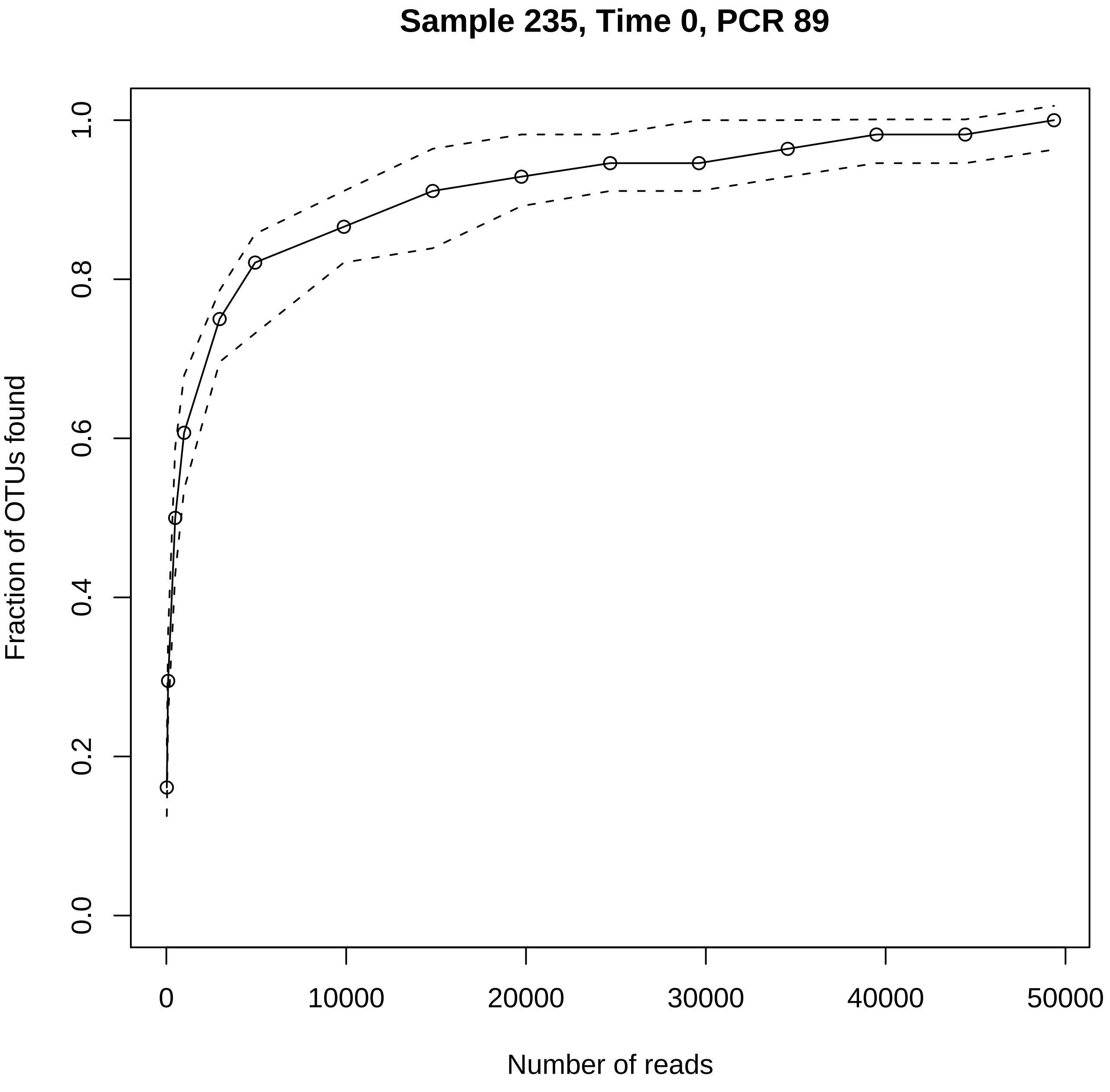

Sample 235, Time 0, PCR 89
Fraction of OTUs found
Number of reads
0.0
0.2
0.4
0.6
0.8
1.0
0
10000
20000
30000
40000
50000

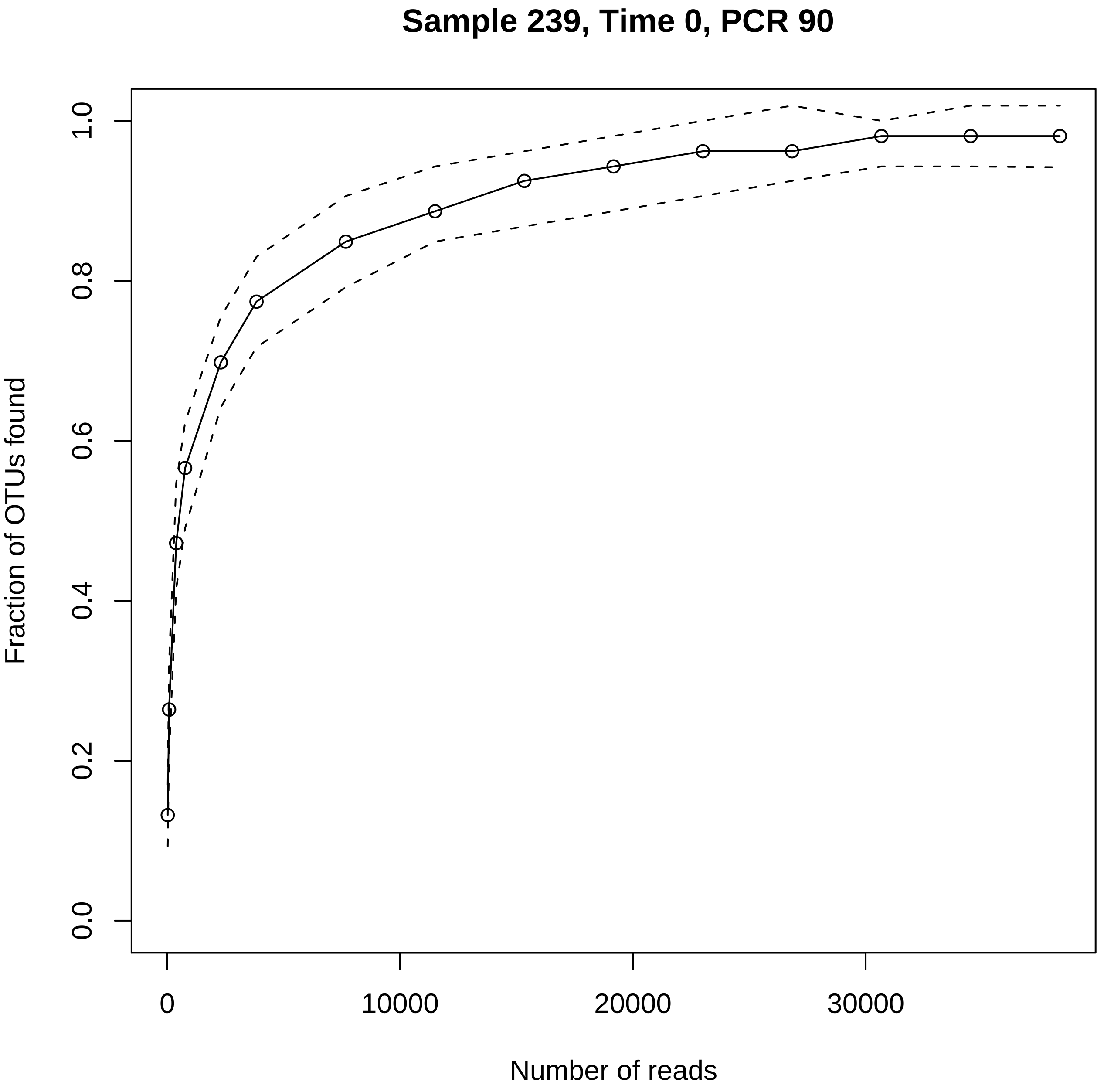
Sample 239, Time 0, PCR 90
Fraction of OTUs found
0.0
0.2
0.4
0.6
0.8
1.0
0
10000
20000
30000
Number of reads

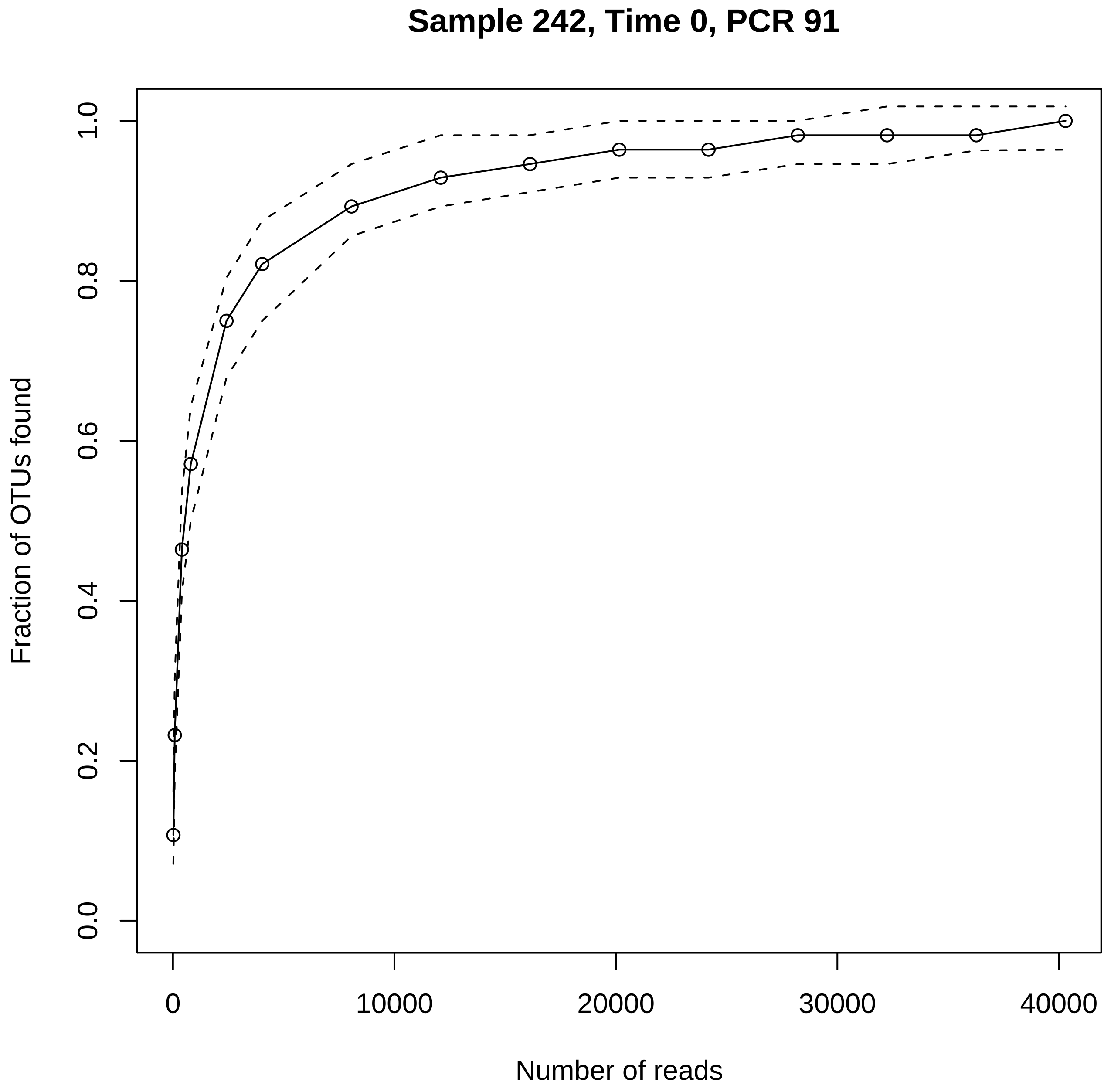
Sample 242, Time 0, PCR 91
Fraction of OTUs found
Number of reads
0.0
0.2
0.4
0.6
0.8
1.0
0
10000
20000
30000
40000

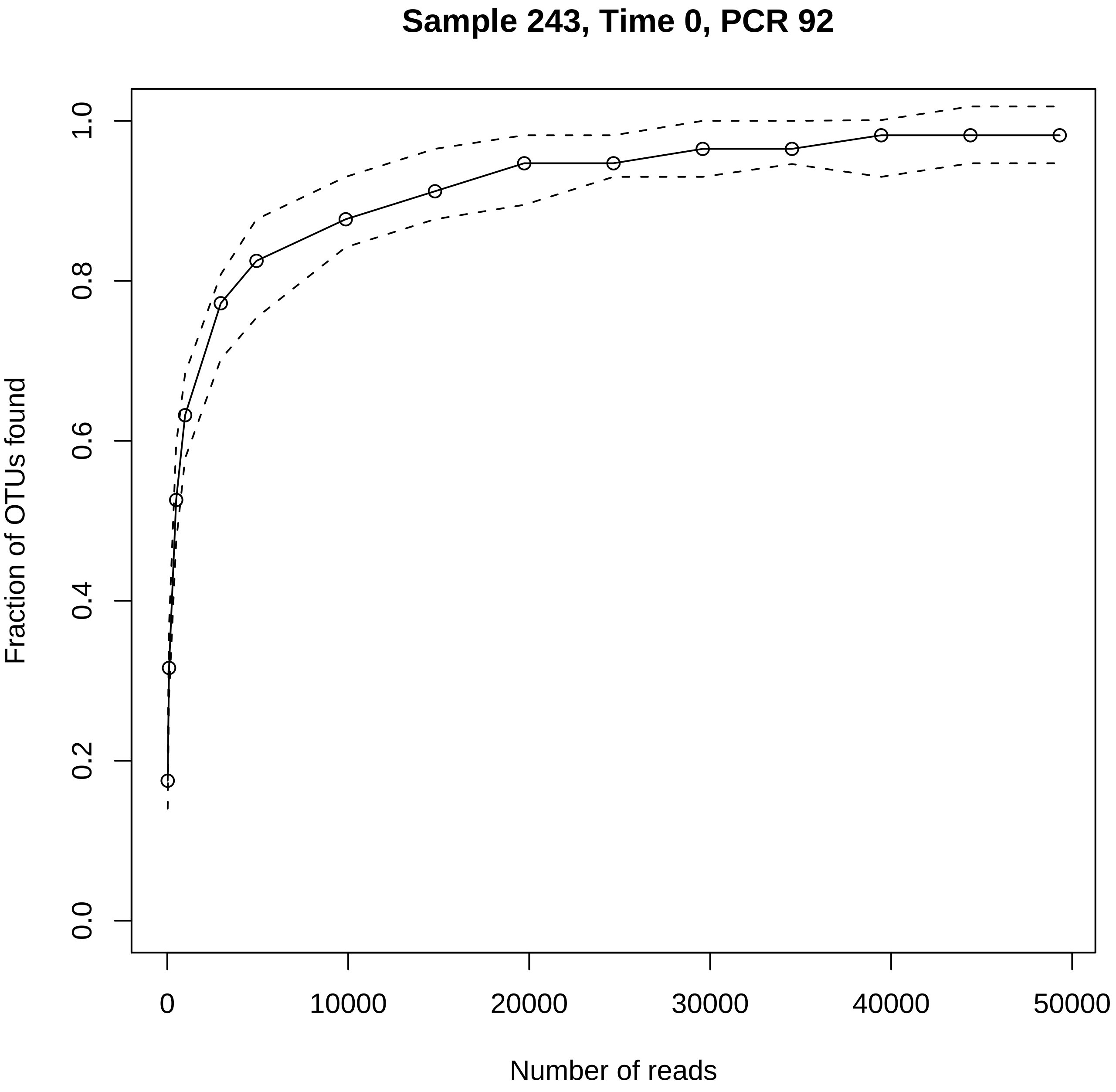
Sample 243, Time 0, PCR 92
Fraction of OTUs found
Number of reads
0.0
0.2
0.4
0.6
0.8
1.0
0
10000
20000
30000
40000
50000

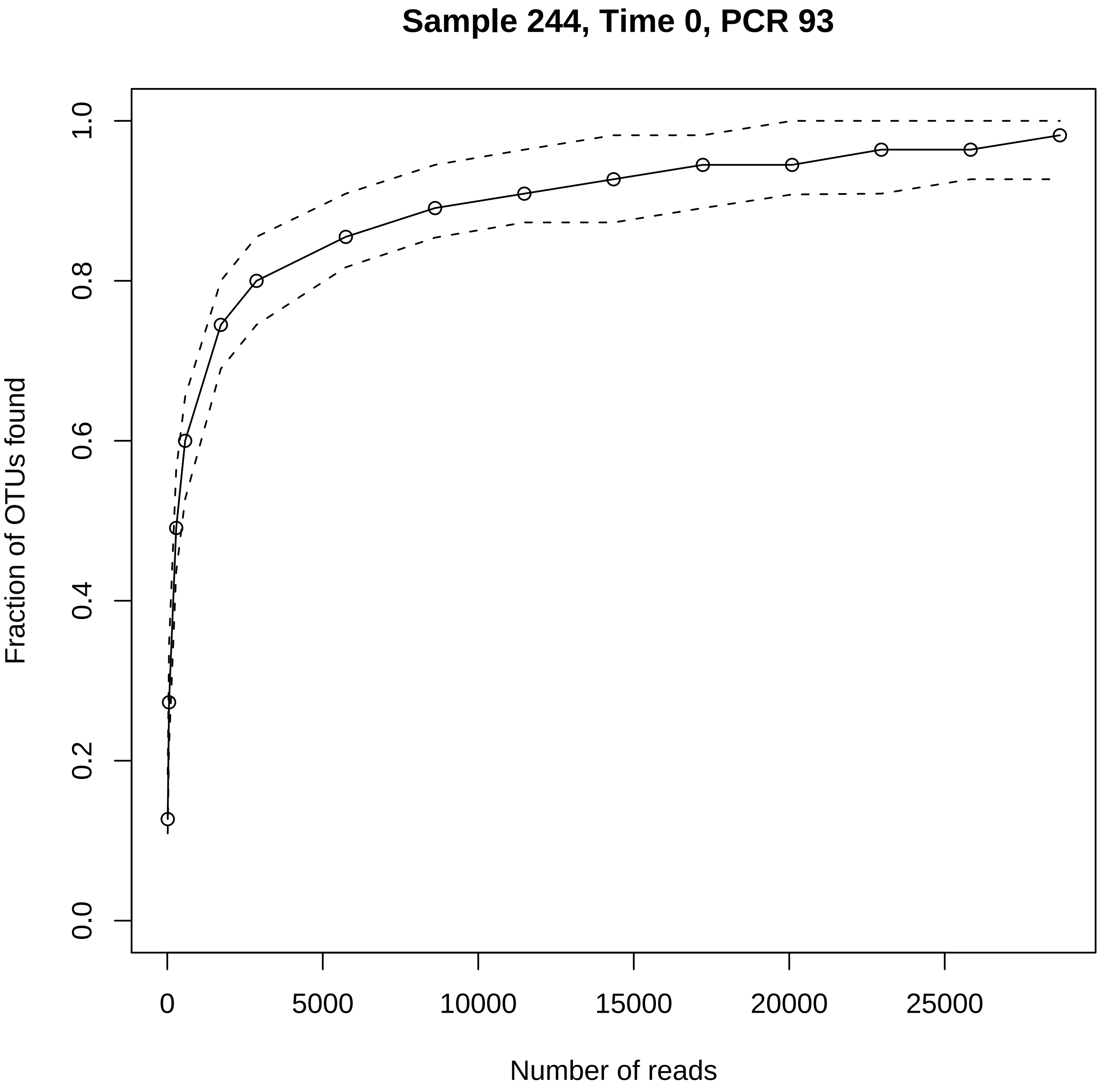

Sample 244, Time 0, PCR 93
Fraction of OTUs found
0.0
0.2
0.4
0.6
0.8
1.0
0
5000
10000
15000
20000
25000
Number of reads

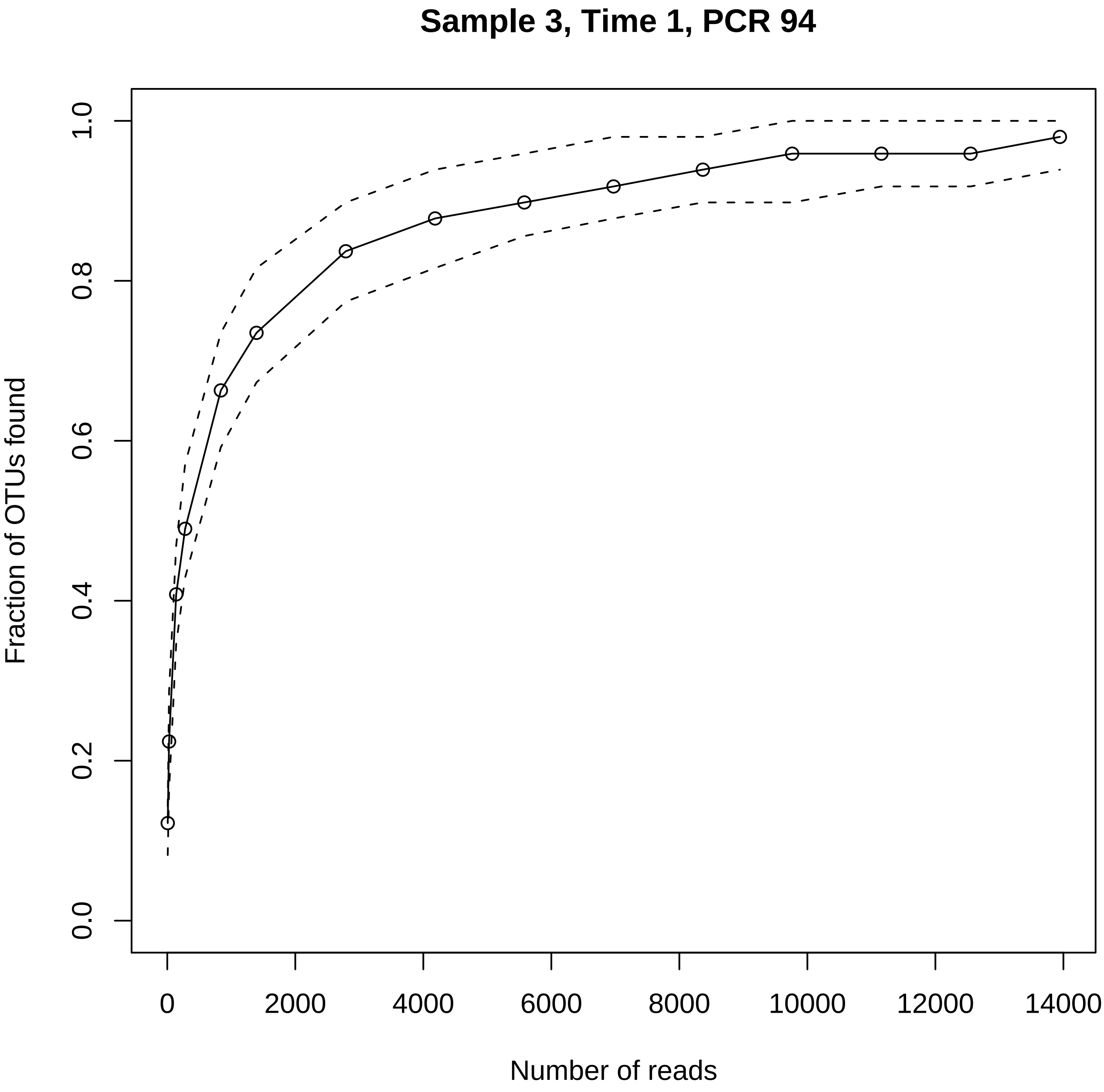
Sample 3, Time 1, PCR 94
Fraction of OTUs found
0.0
0.2
0.4
0.6
0.8
1.0
0
2000
4000
6000
8000
10000
12000
14000
Number of reads

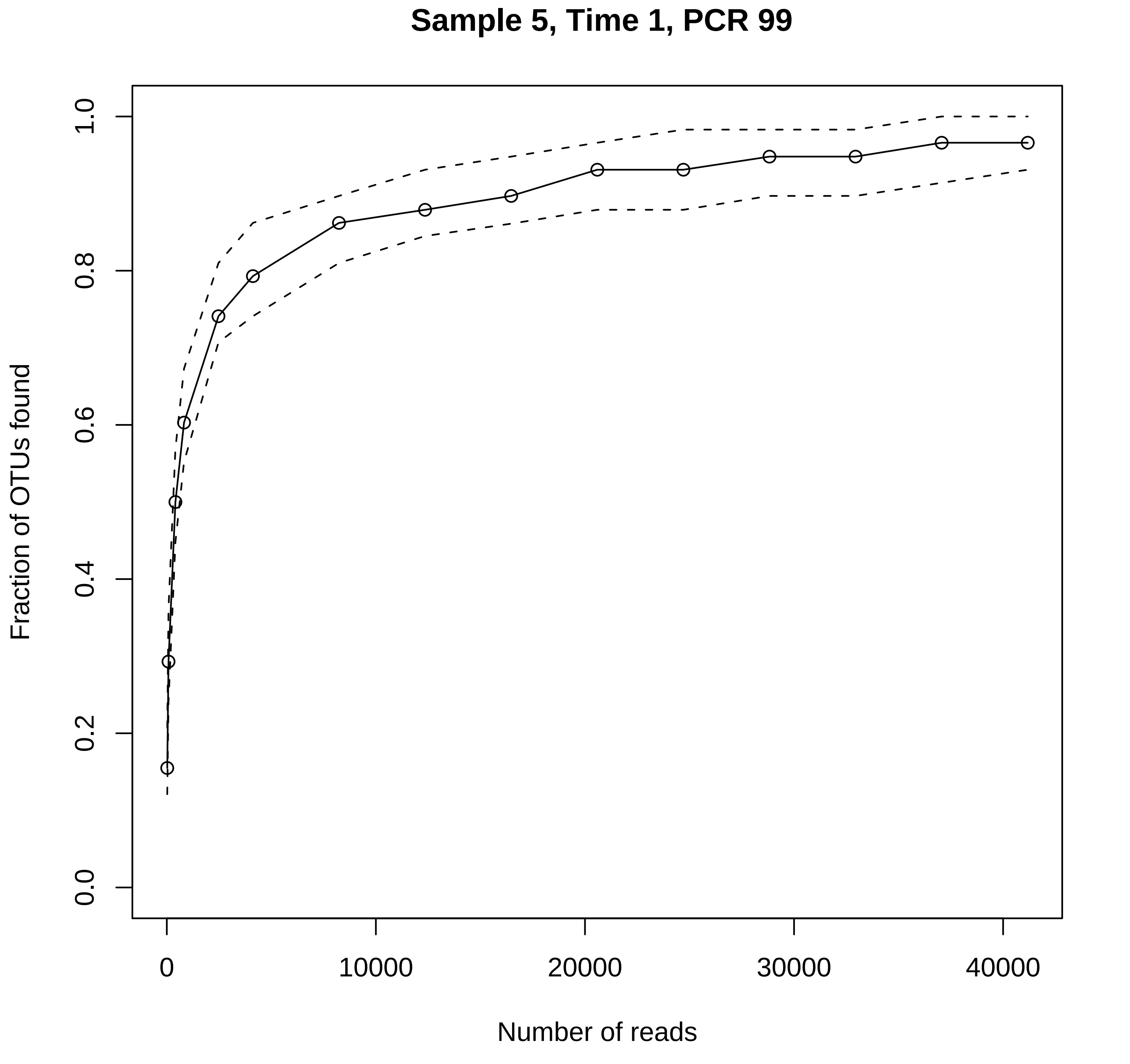

Sample 5, Time 1, PCR 99
Fraction of OTUs found
0.0
0.2
0.4
0.6
0.8
1.0
0
10000
20000
30000
40000
Number of reads

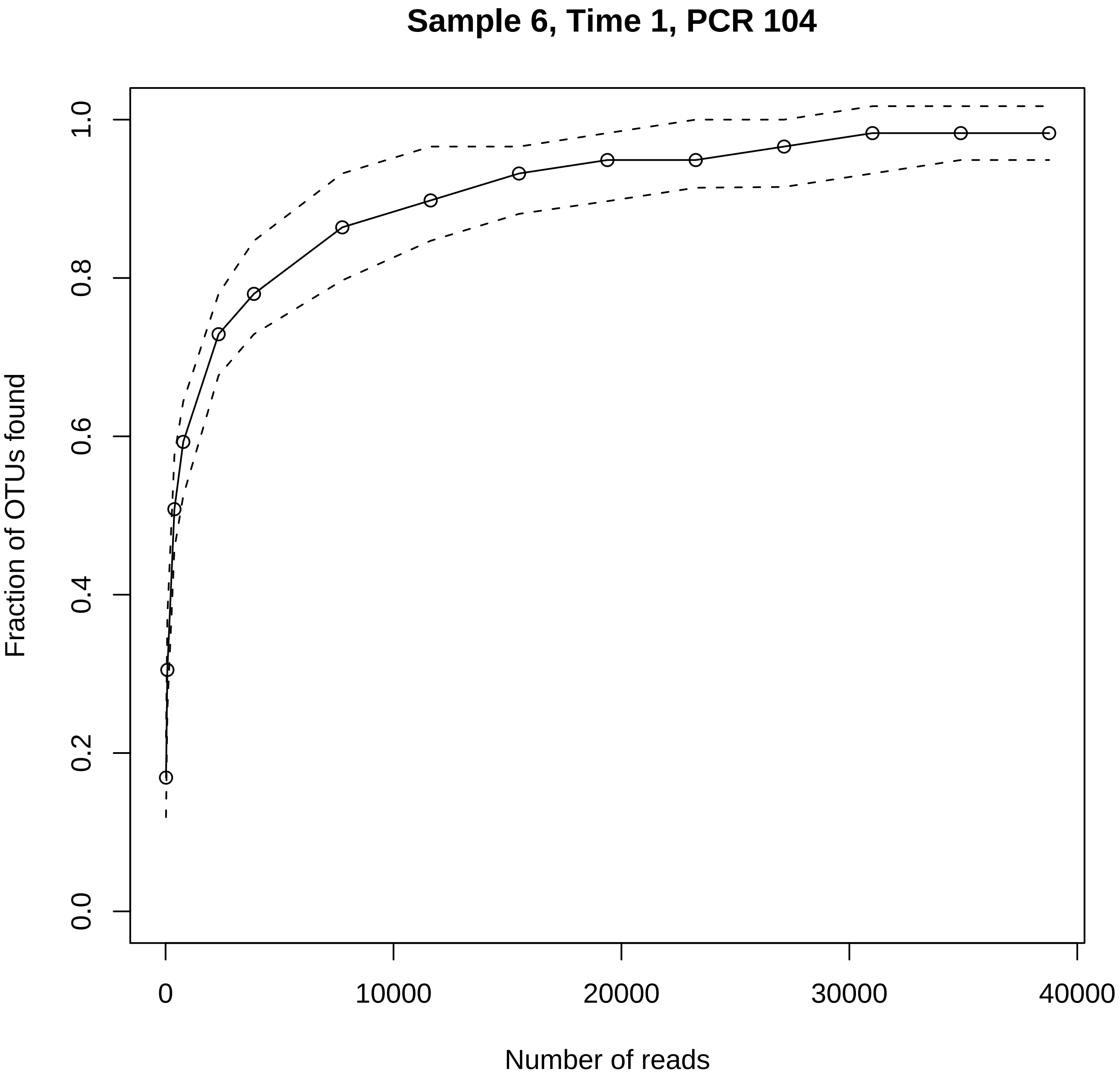

Sample 6, Time 1, PCR 104
Fraction of OTUs found
Number of reads
0.0
0.2
0.4
0.6
0.8
1.0
0
10000
20000
30000
40000

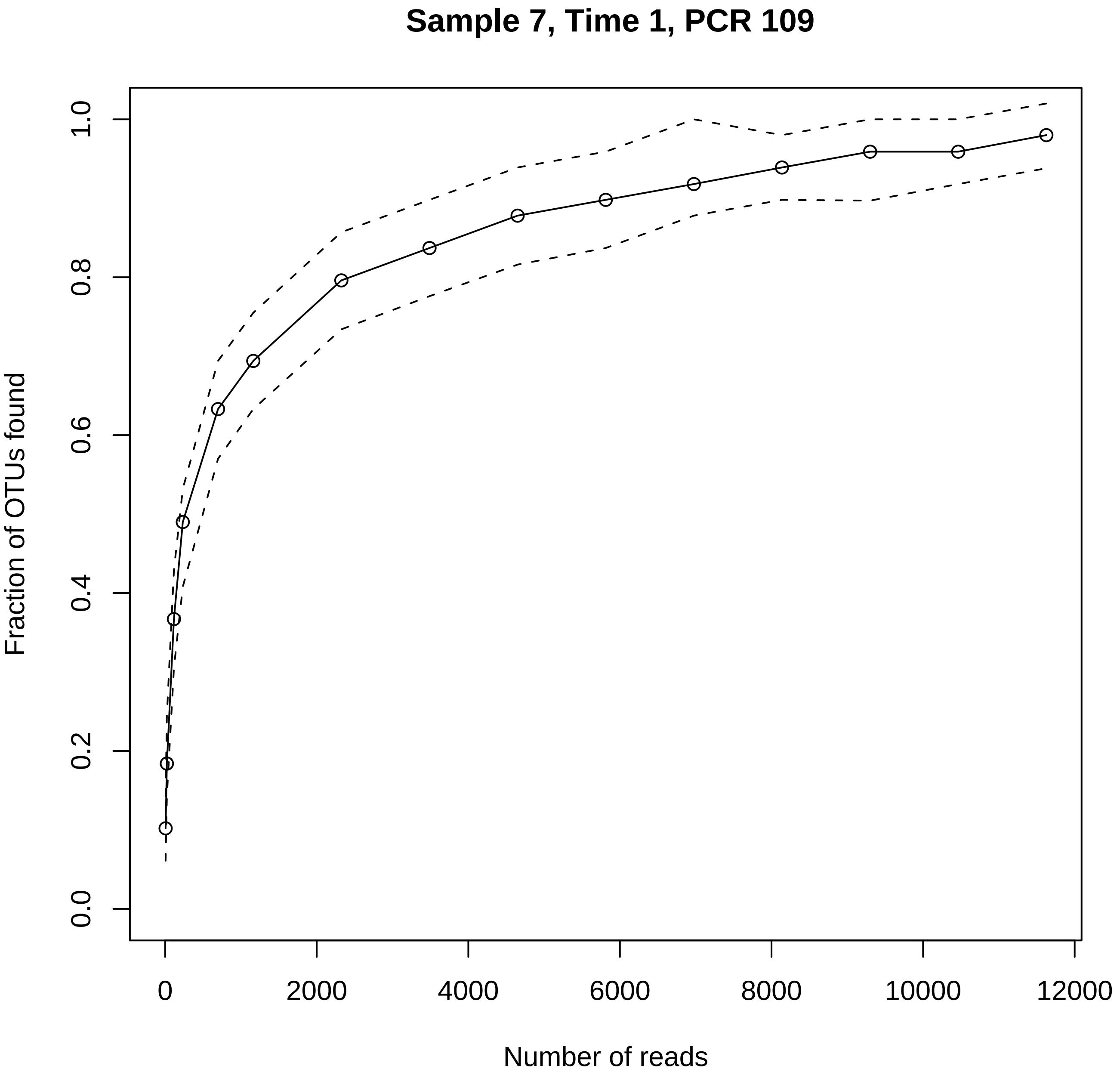

Sample 7, Time 1, PCR 109
Fraction of OTUs found
0.0
0.2
0.4
0.6
0.8
1.0
0
2000
4000
6000
8000
10000
12000
Number of reads

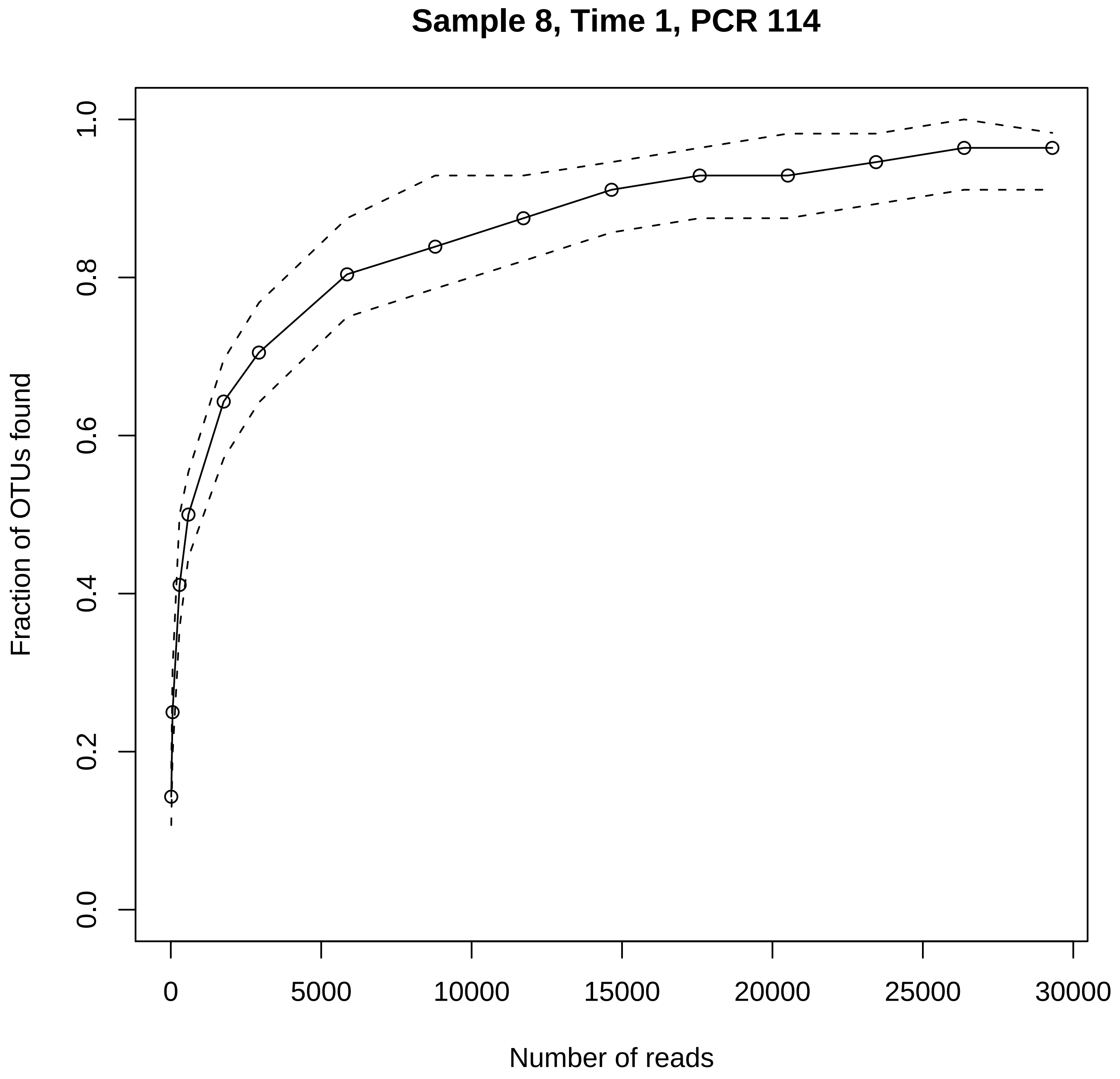
Sample 8, Time 1, PCR 114
Fraction of OTUs found
Number of reads
0.0
0.2
0.4
0.6
0.8
1.0
0
5000
10000
15000
20000
25000
30000

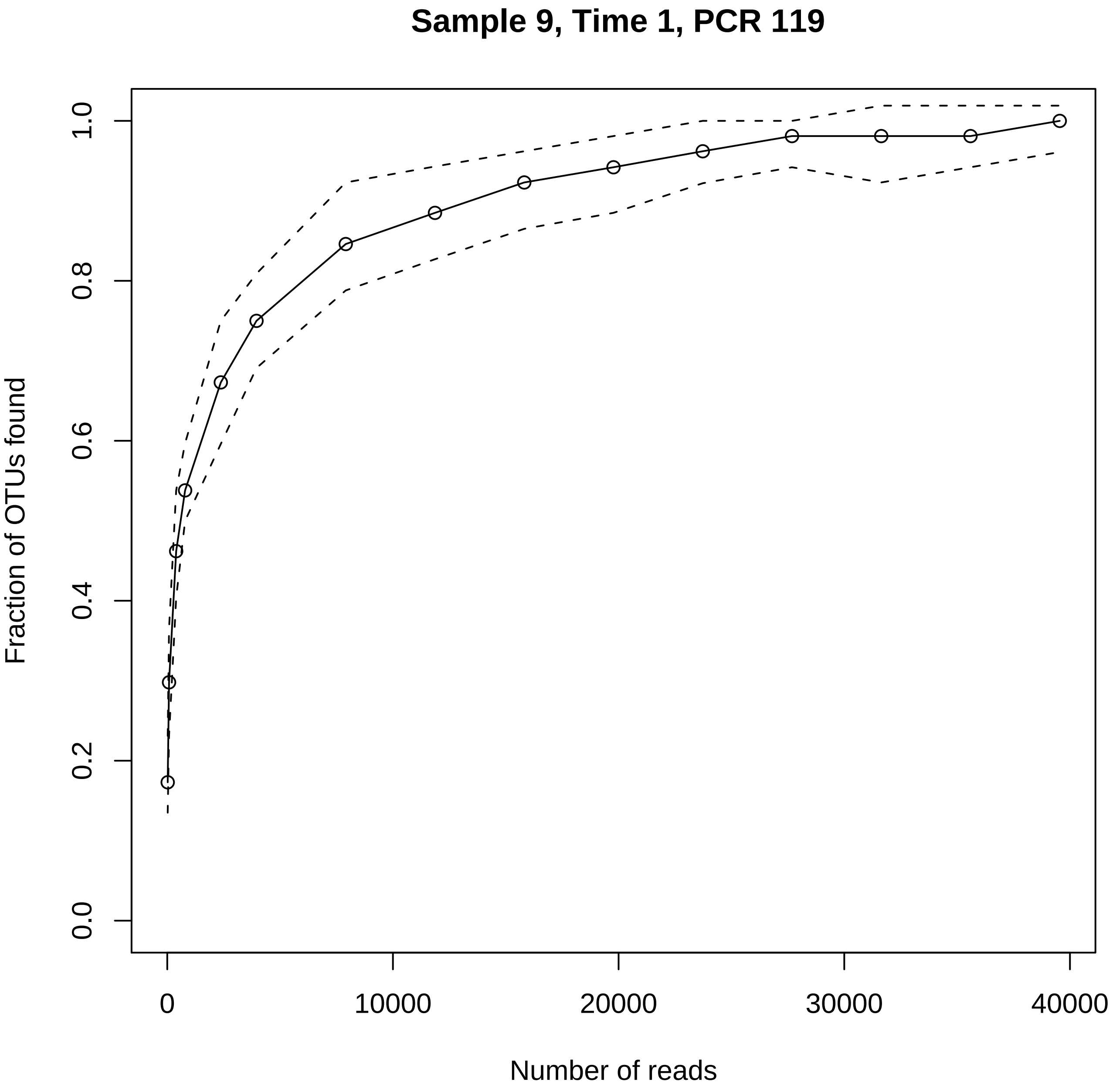
Sample 9, Time 1, PCR 119
Fraction of OTUs found
0.0
0.2
0.4
0.6
0.8
1.0
0
10000
20000
30000
40000
Number of reads

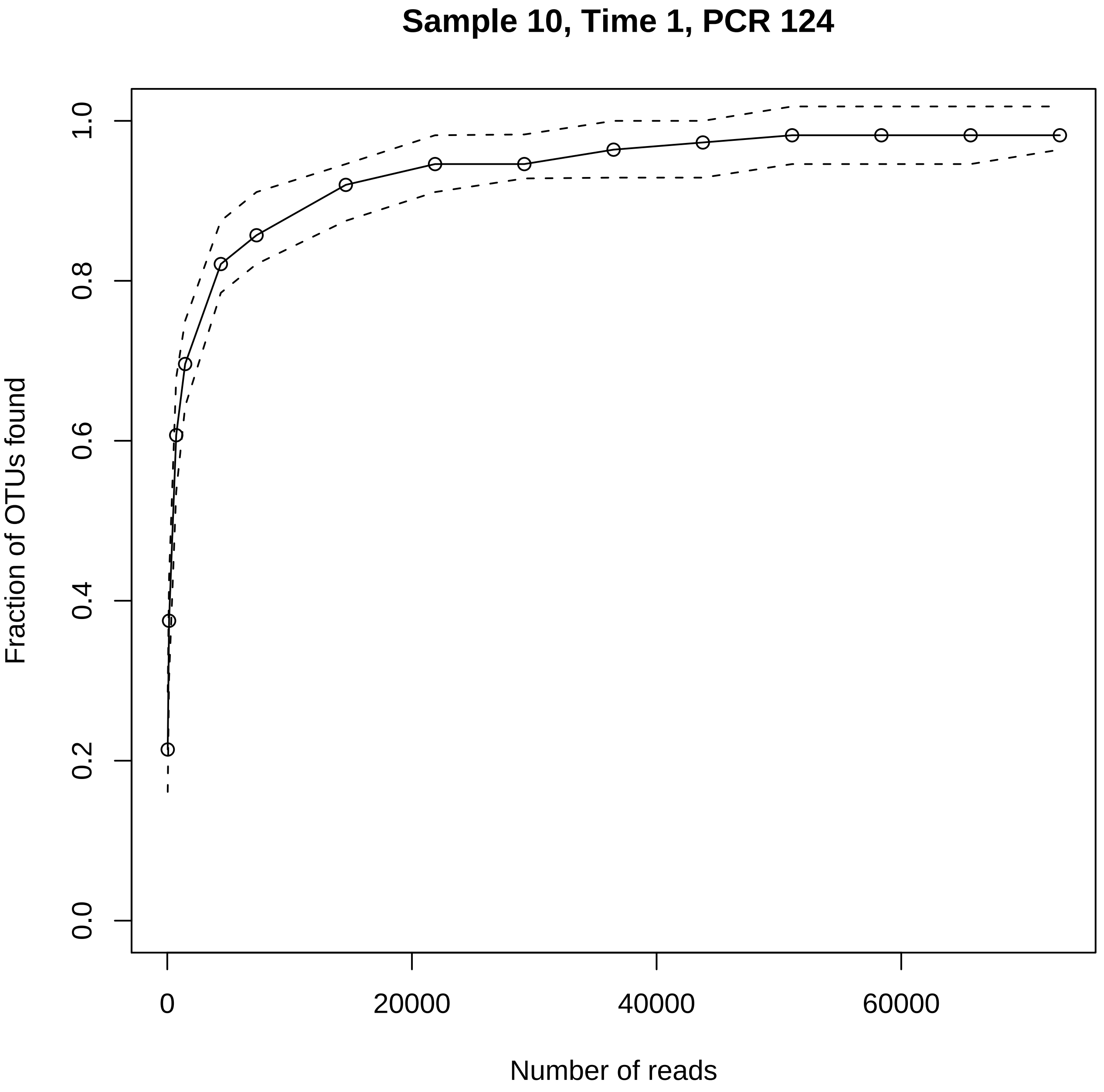

Sample 10, Time 1, PCR 124
Fraction of OTUs found
Number of reads
0.0
0.2
0.4
0.6
0.8
1.0
0
20000
40000
60000

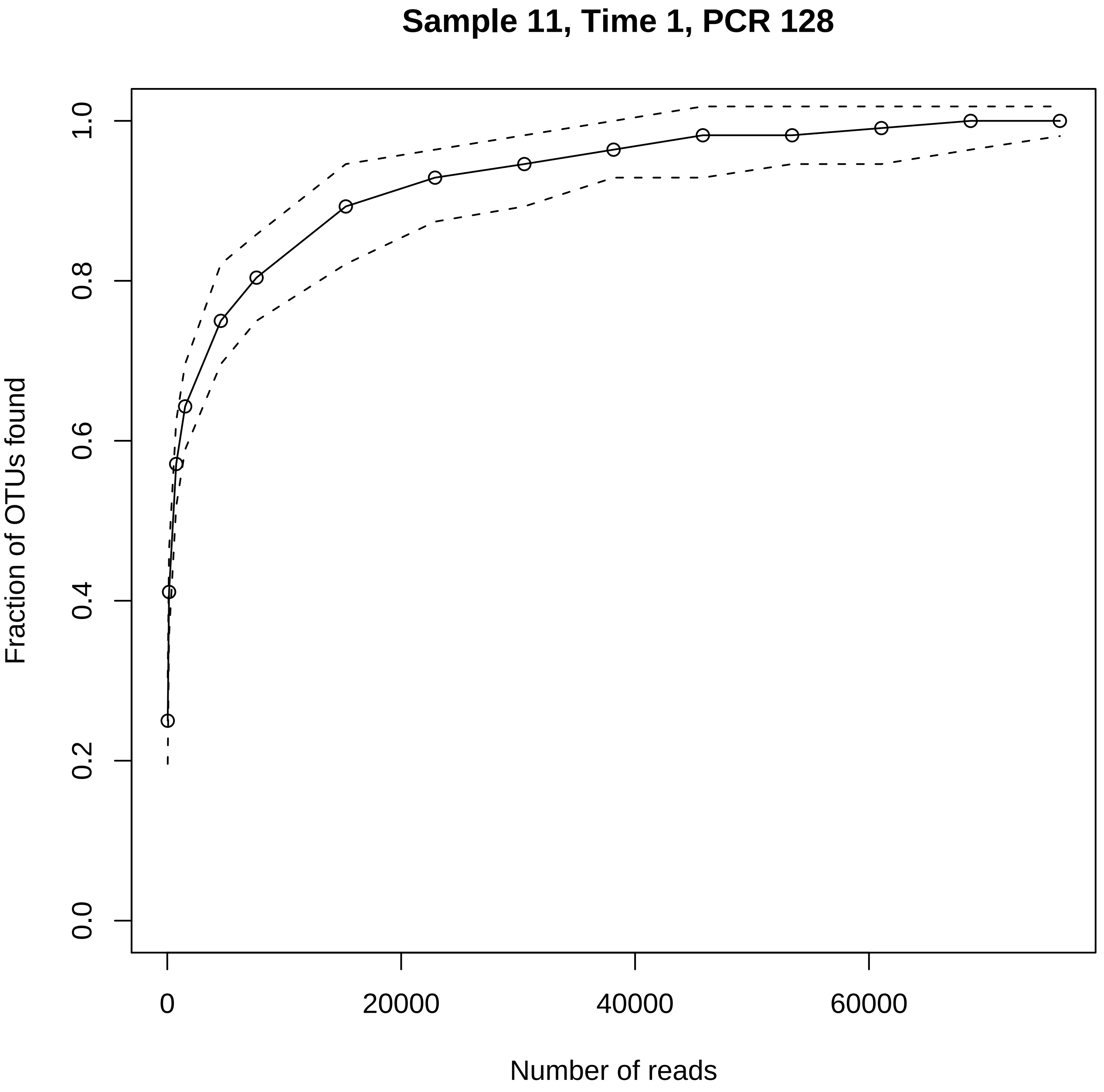

Sample 11, Time 1, PCR 128
Fraction of OTUs found
0.0
0.2
0.4
0.6
0.8
1.0
0
20000
40000
60000
Number of reads

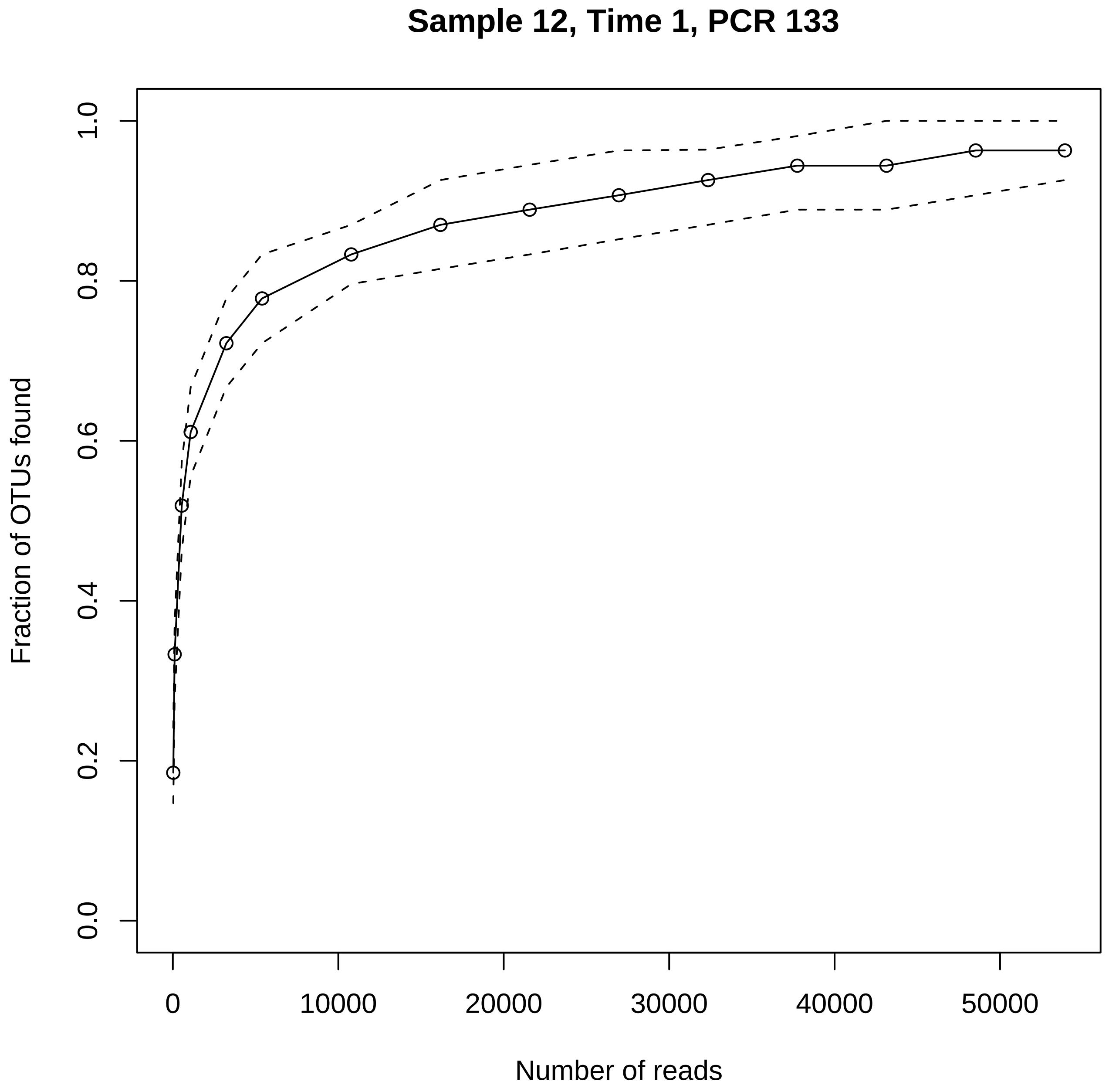
Sample 12, Time 1, PCR 133
Fraction of OTUs found
0.0
0.2
0.4
0.6
0.8
1.0
0
10000
20000
30000
40000
50000
Number of reads

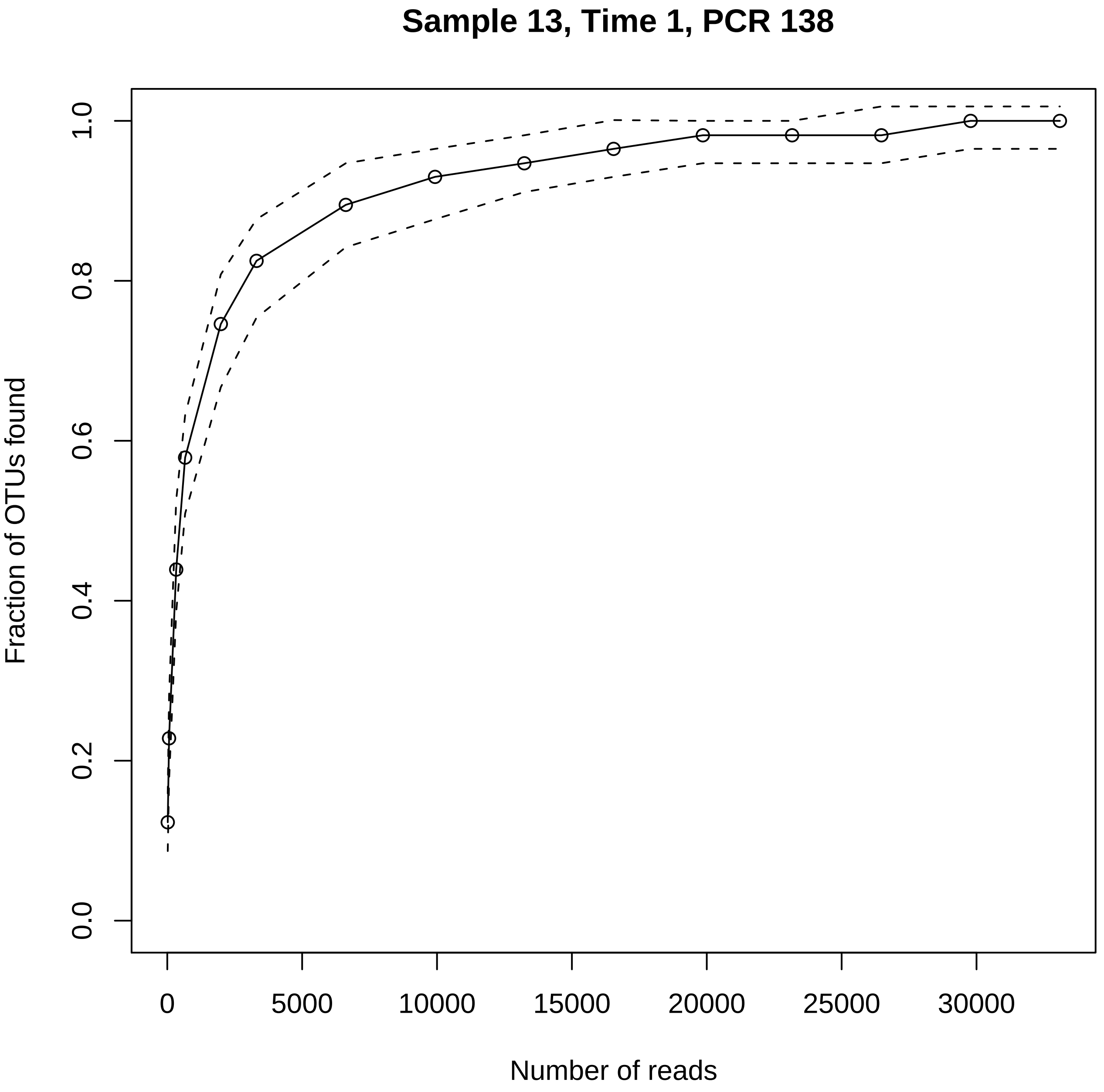
Sample 13, Time 1, PCR 138
Fraction of OTUs found
0.0
0.2
0.4
0.6
0.8
1.0
0
5000
10000
15000
20000
25000
30000
Number of reads

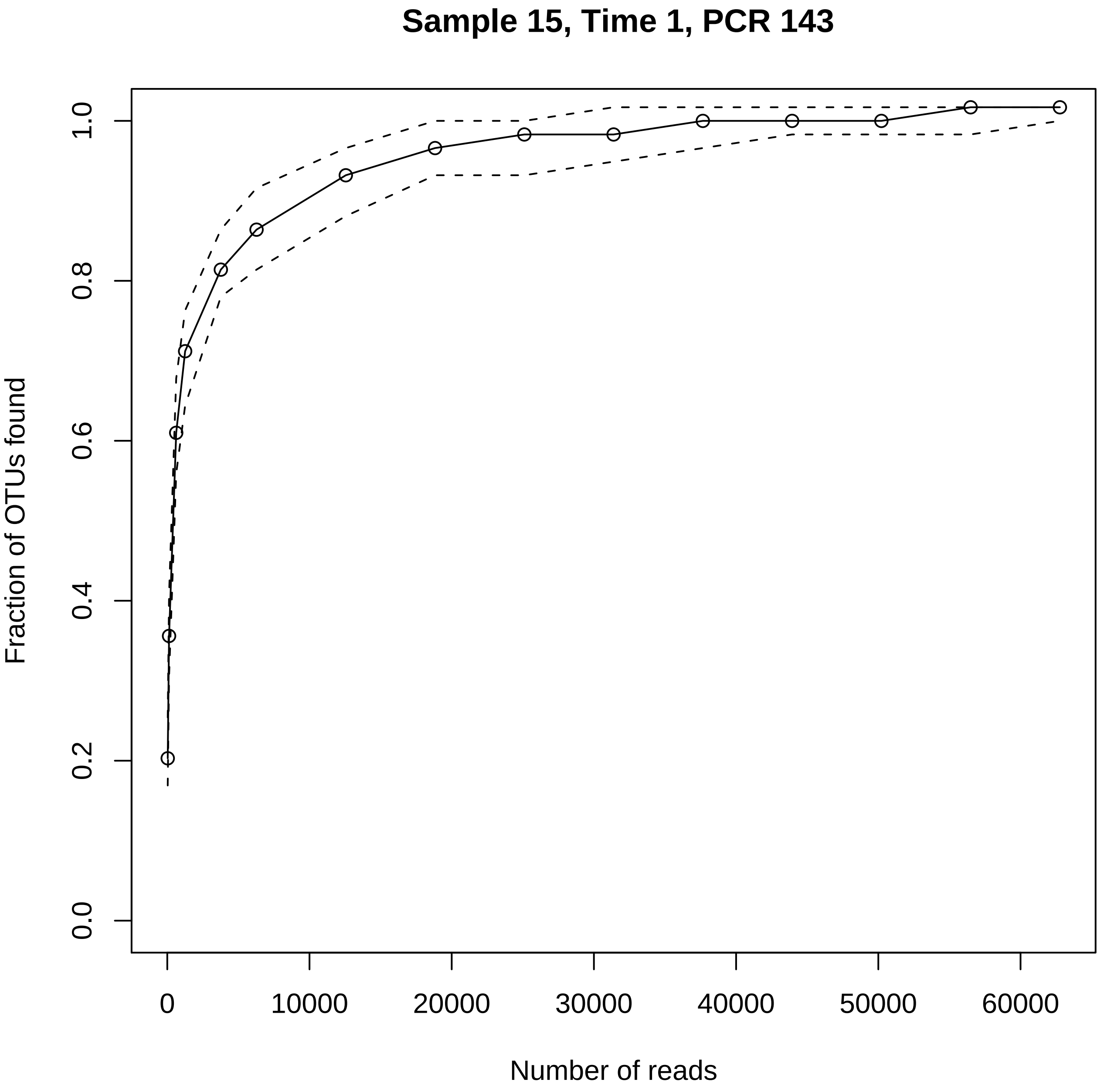

Sample 15, Time 1, PCR 143
Fraction of OTUs found
0.0
0.2
0.4
0.6
0.8
1.0
0
10000
20000
30000
40000
50000
60000
Number of reads

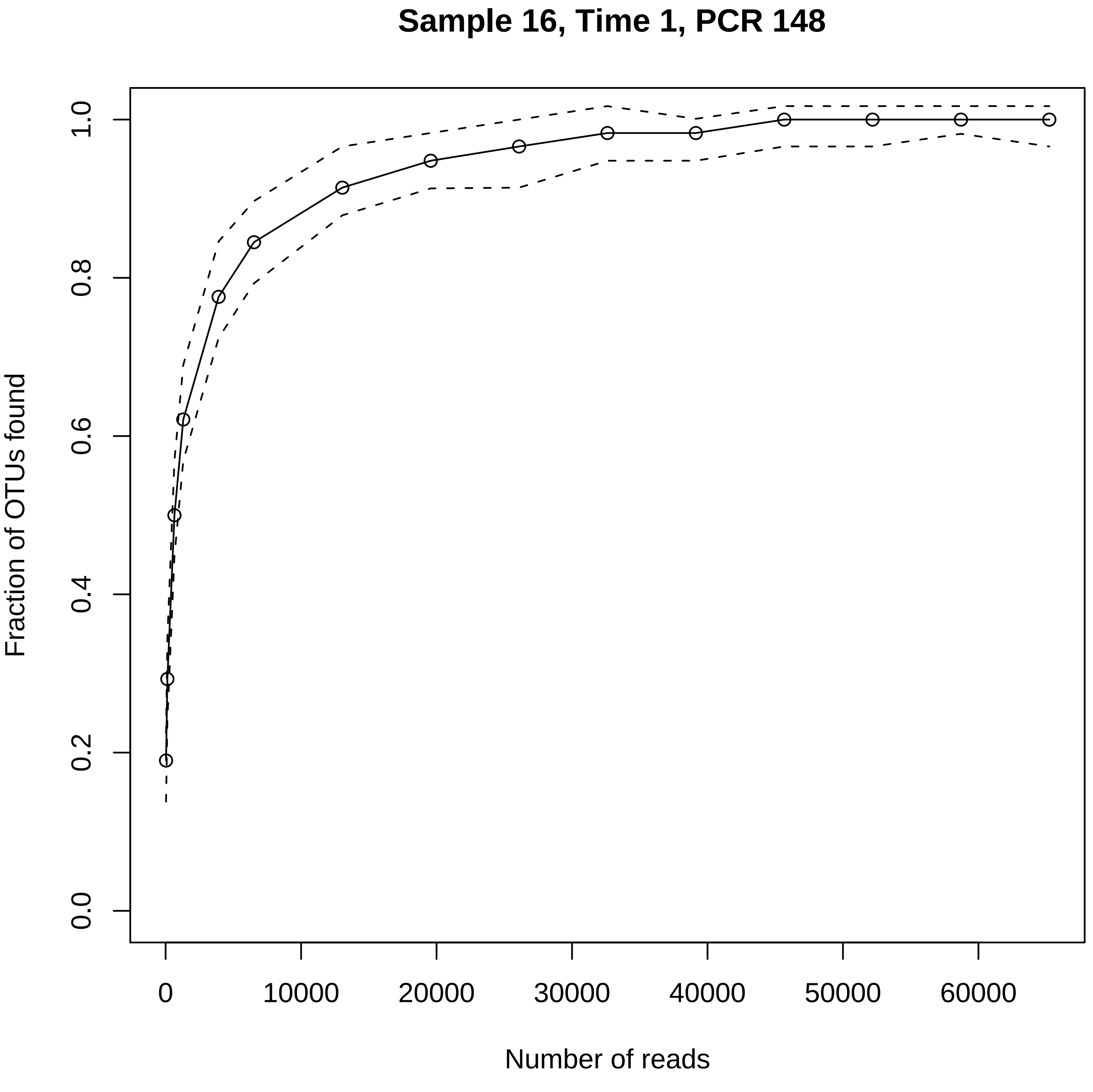
Sample 16, Time 1, PCR 148
Fraction of OTUs found
0.0
0.2
0.4
0.6
0.8
1.0
0
10000
20000
30000
40000
50000
60000
Number of reads

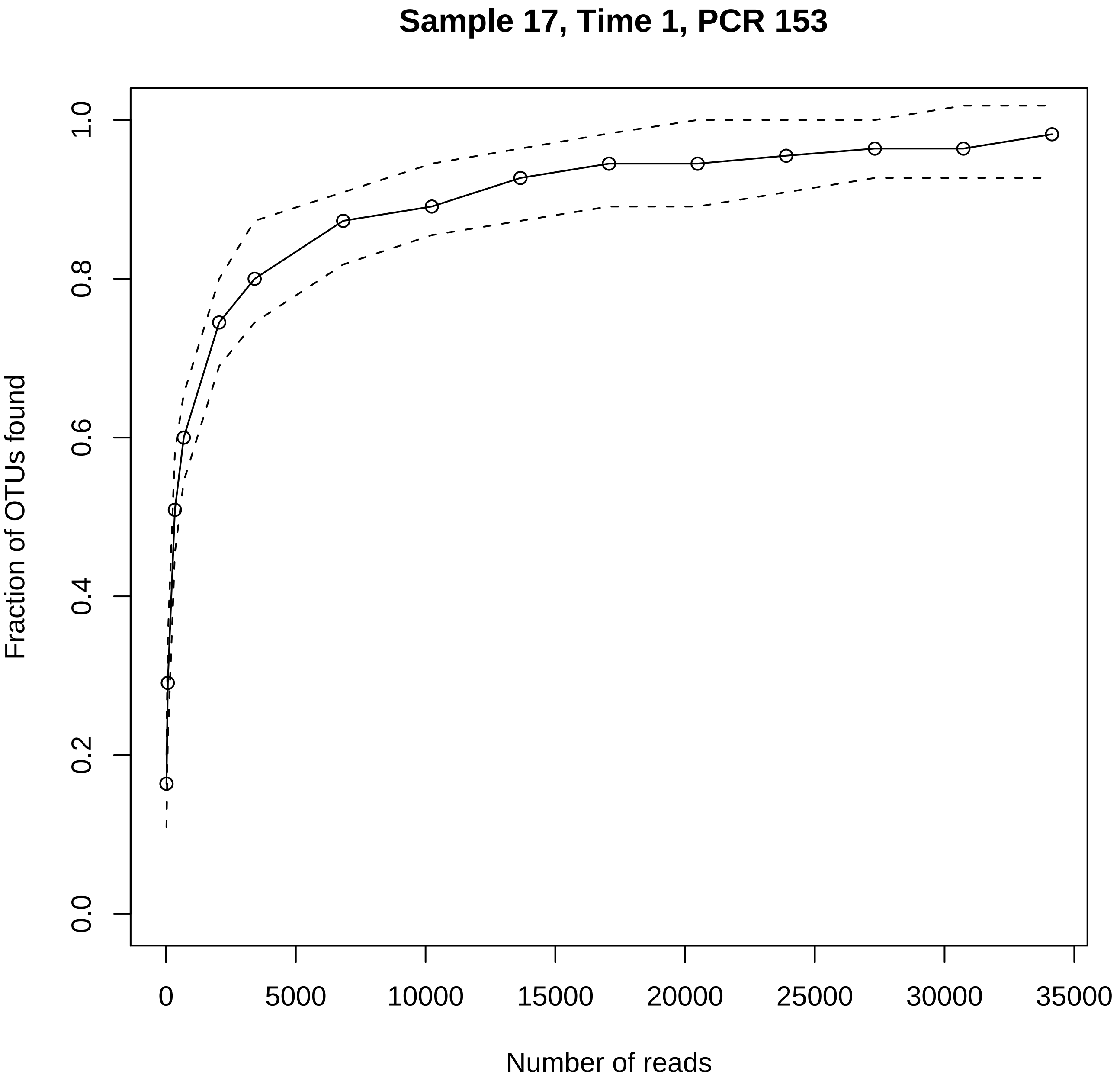

Sample 17, Time 1, PCR 153
Fraction of OTUs found
Number of reads
0.0
0.2
0.4
0.6
0.8
1.0
0
5000
10000
15000
20000
25000
30000
35000

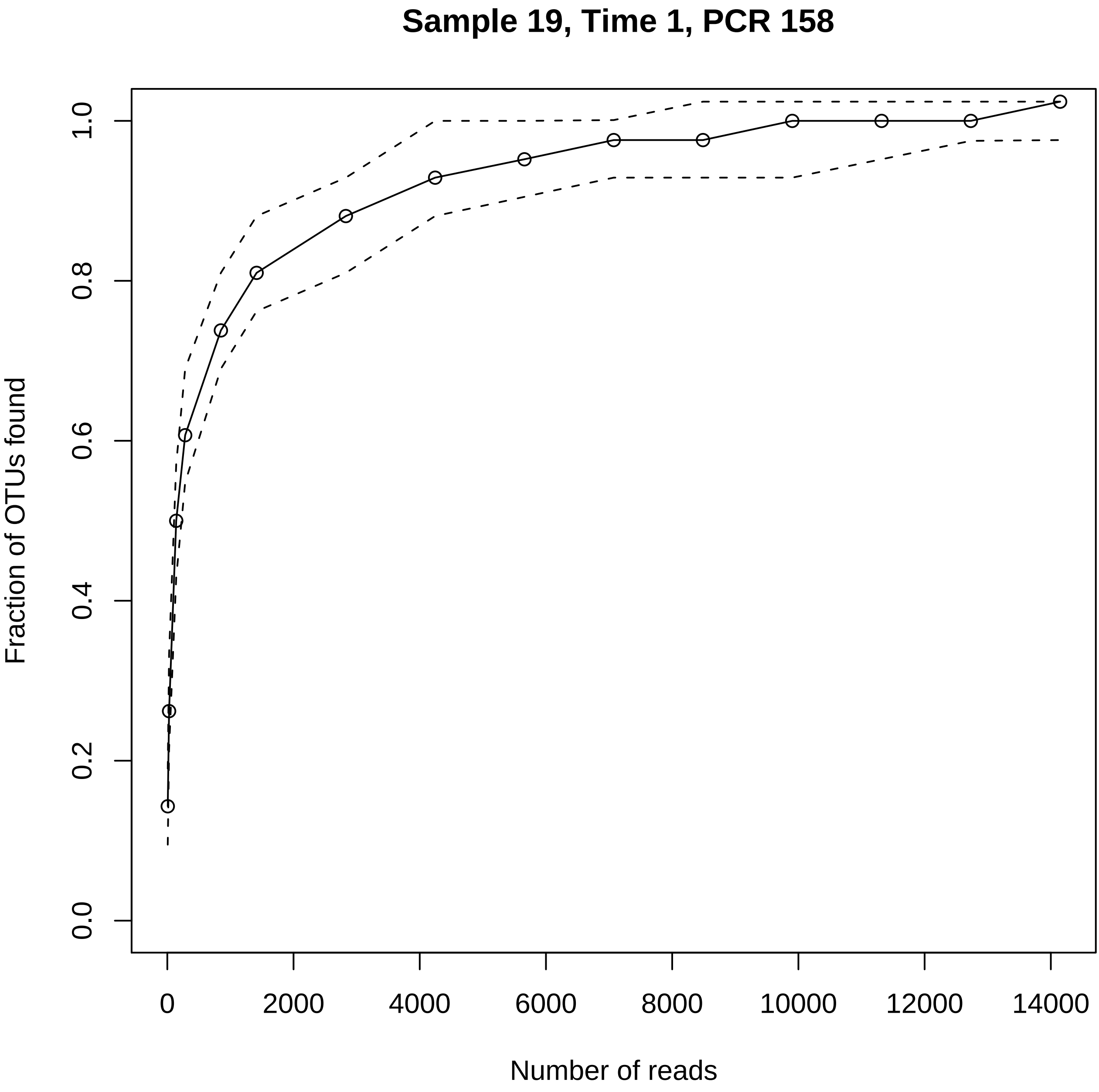
Sample 19, Time 1, PCR 158
Fraction of OTUs found
0.0
0.2
0.4
0.6
0.8
1.0
0
2000
4000
6000
8000
10000
12000
14000
Number of reads

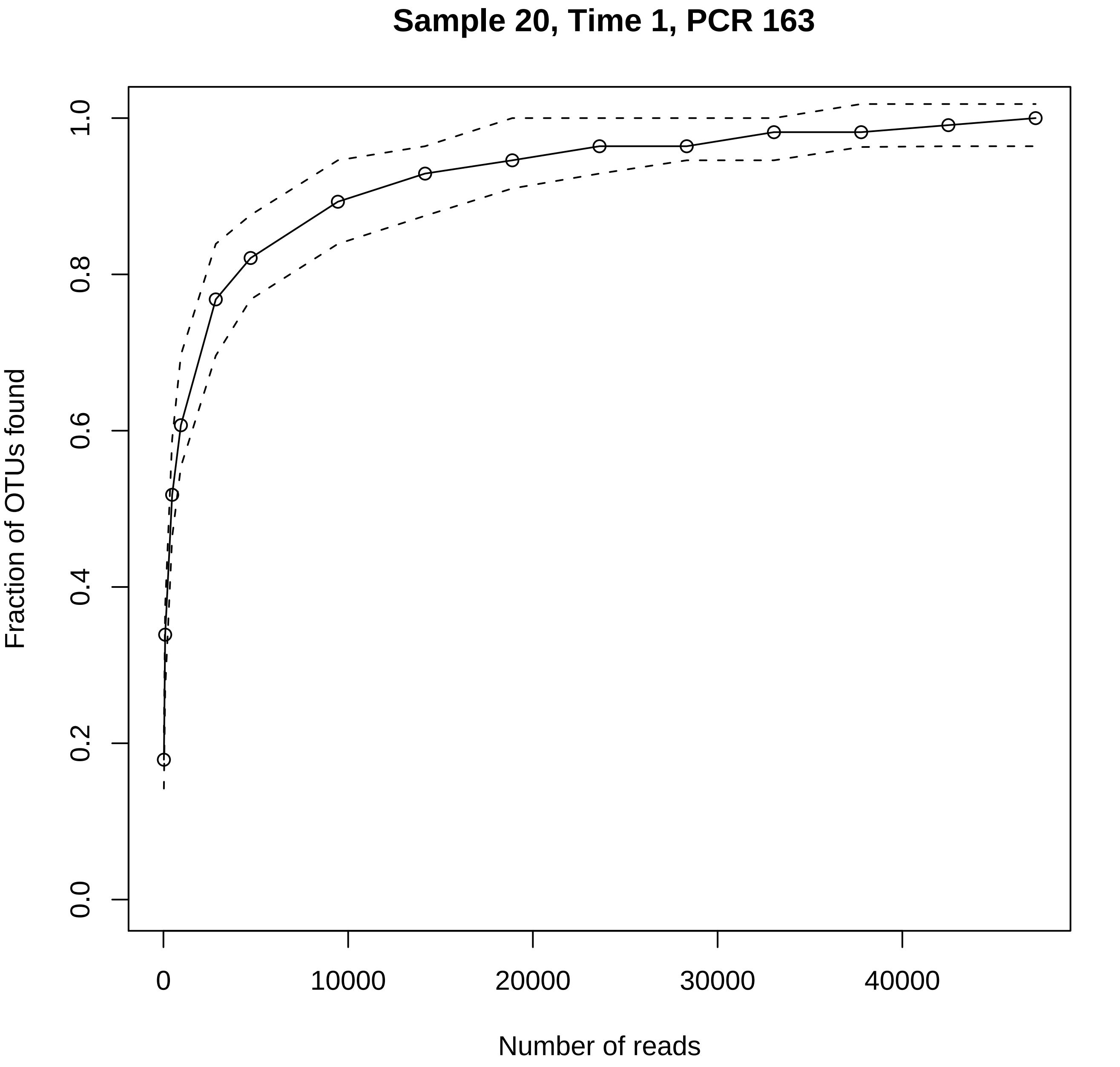

Sample 20, Time 1, PCR 163
Fraction of OTUs found
0.0
0.2
0.4
0.6
0.8
1.0
0
10000
20000
30000
40000
Number of reads

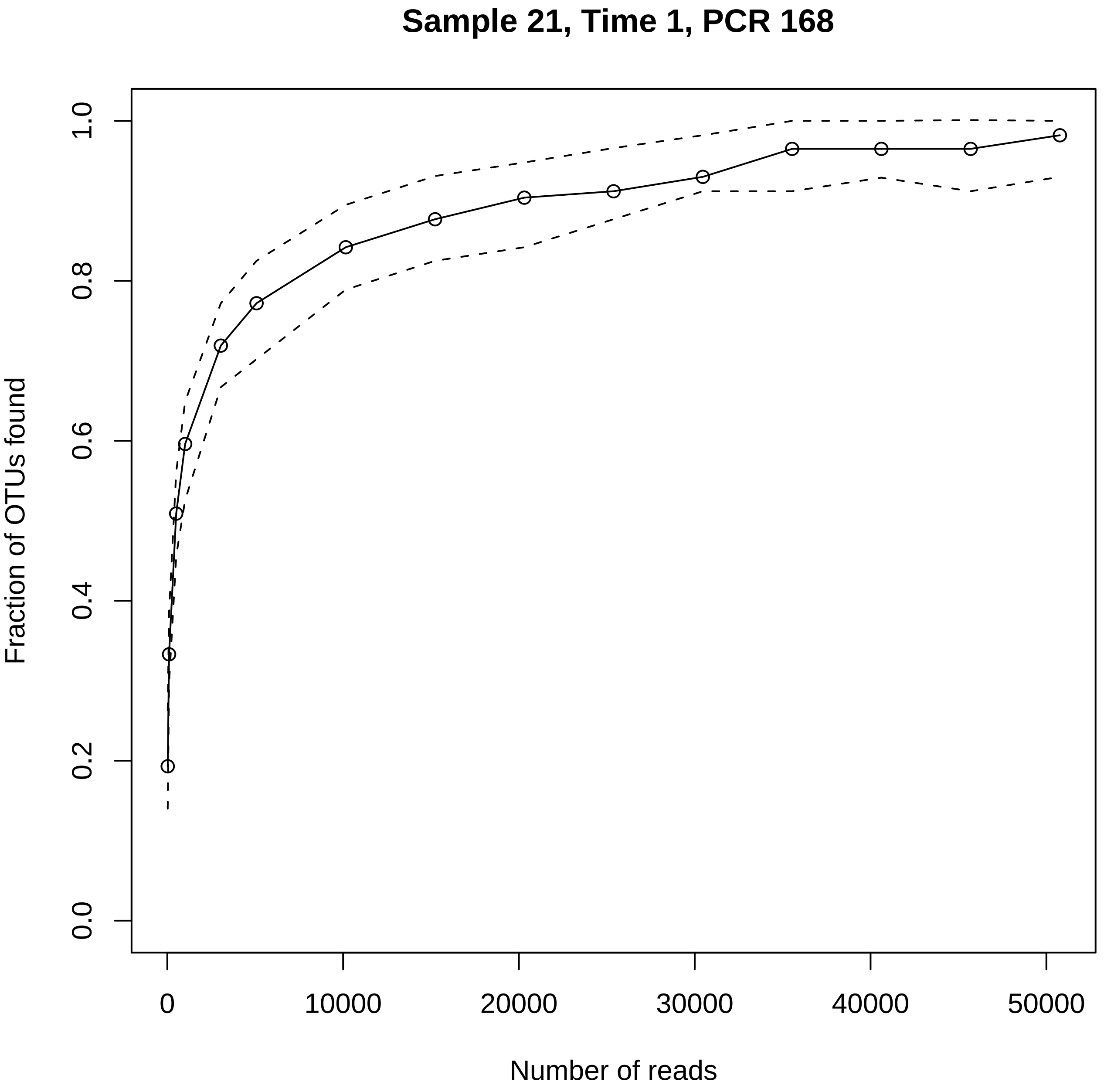
Sample 21, Time 1, PCR 168
Fraction of OTUs found
0.0
0.2
0.4
0.6
0.8
1.0
0
10000
20000
30000
40000
50000
Number of reads

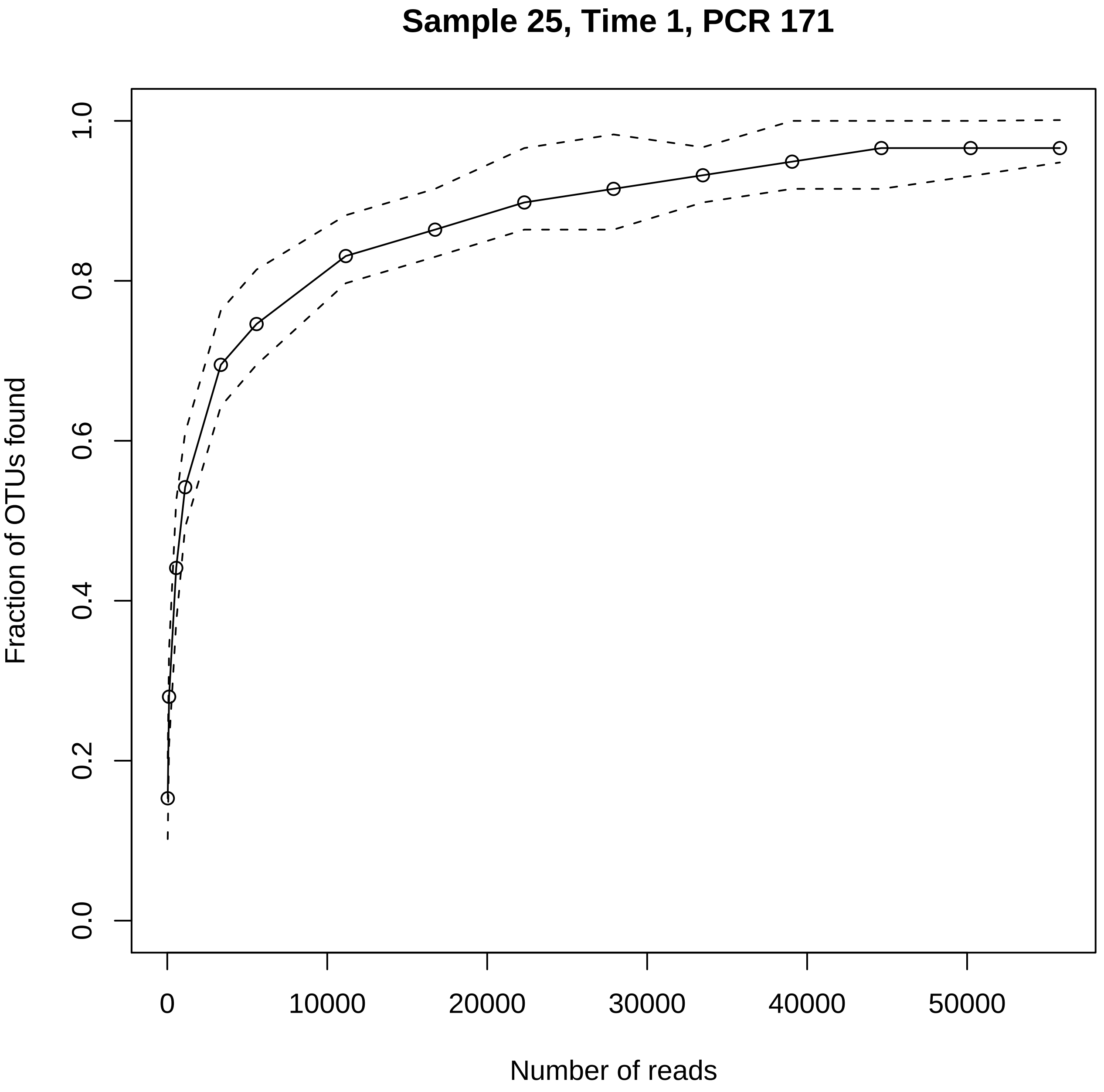
Sample 25, Time 1, PCR 171
Fraction of OTUs found
0.0
0.2
0.4
0.6
0.8
1.0
0
10000
20000
30000
40000
50000
Number of reads

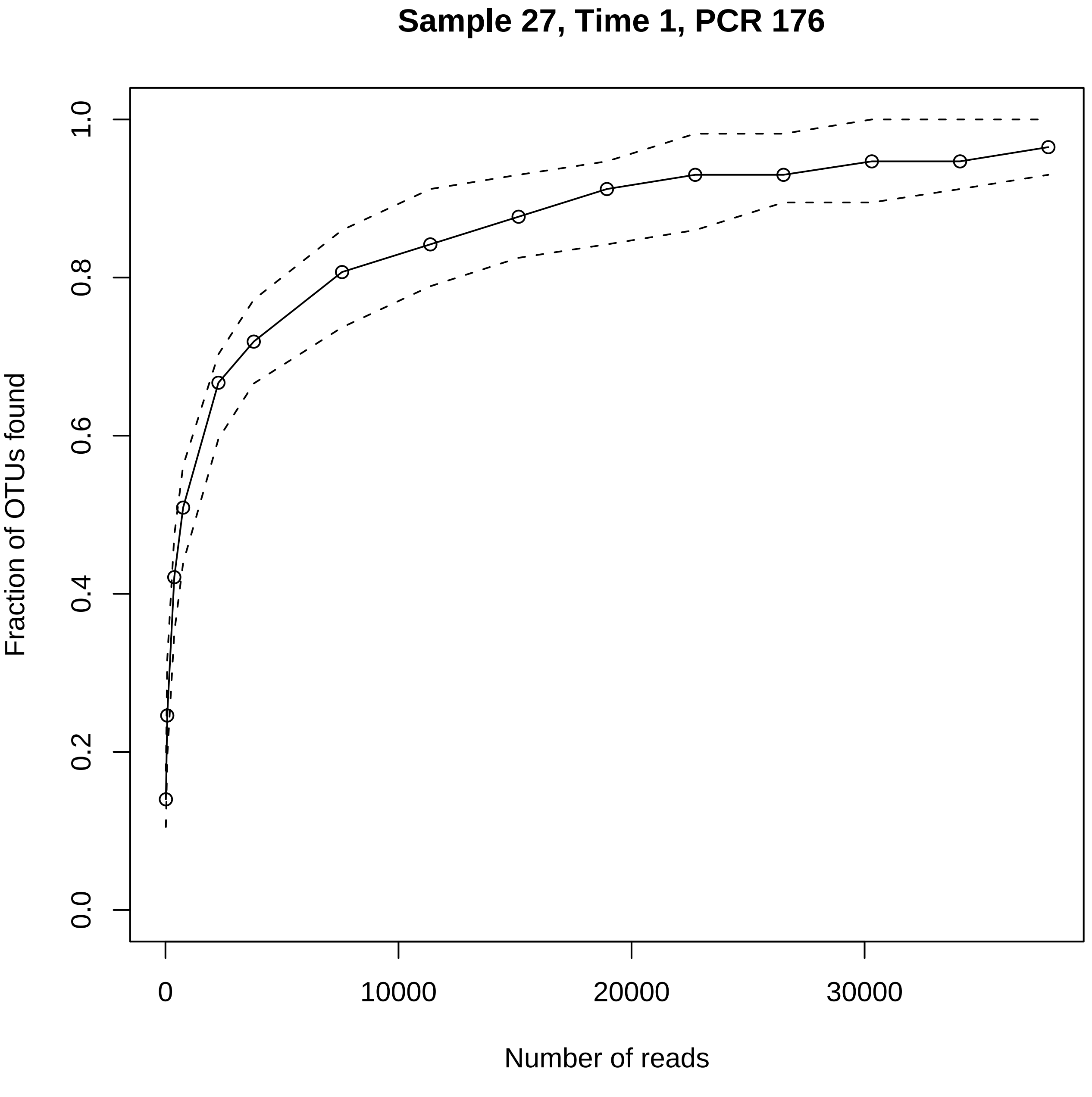
Sample 27, Time 1, PCR 176
Fraction of OTUs found
0.0
0.2
0.4
0.6
0.8
1.0
0
10000
20000
30000
Number of reads

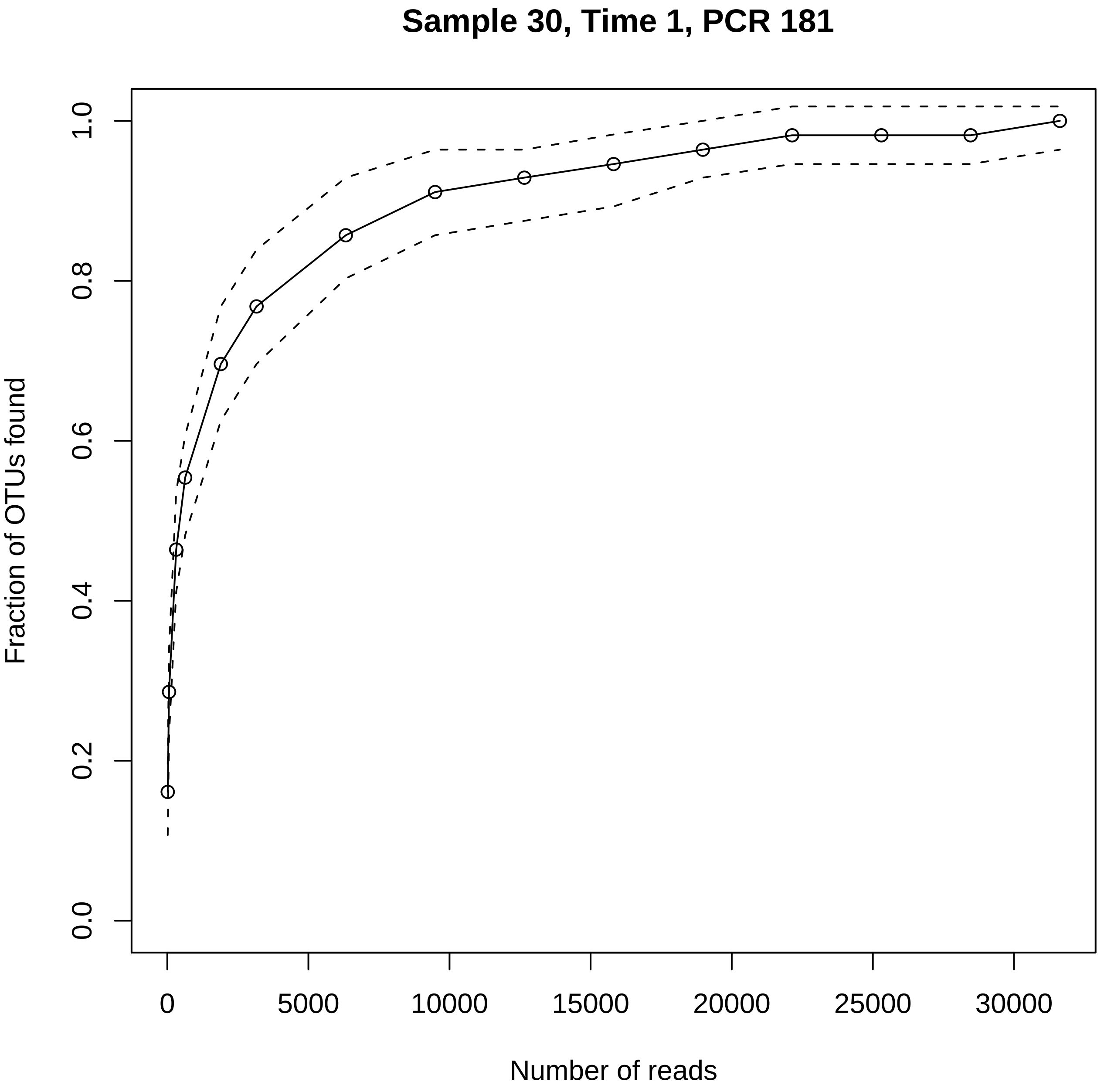

Sample 30, Time 1, PCR 181
Fraction of OTUs found
Number of reads
0.0
0.2
0.4
0.6
0.8
1.0
0
5000
10000
15000
20000
25000
30000

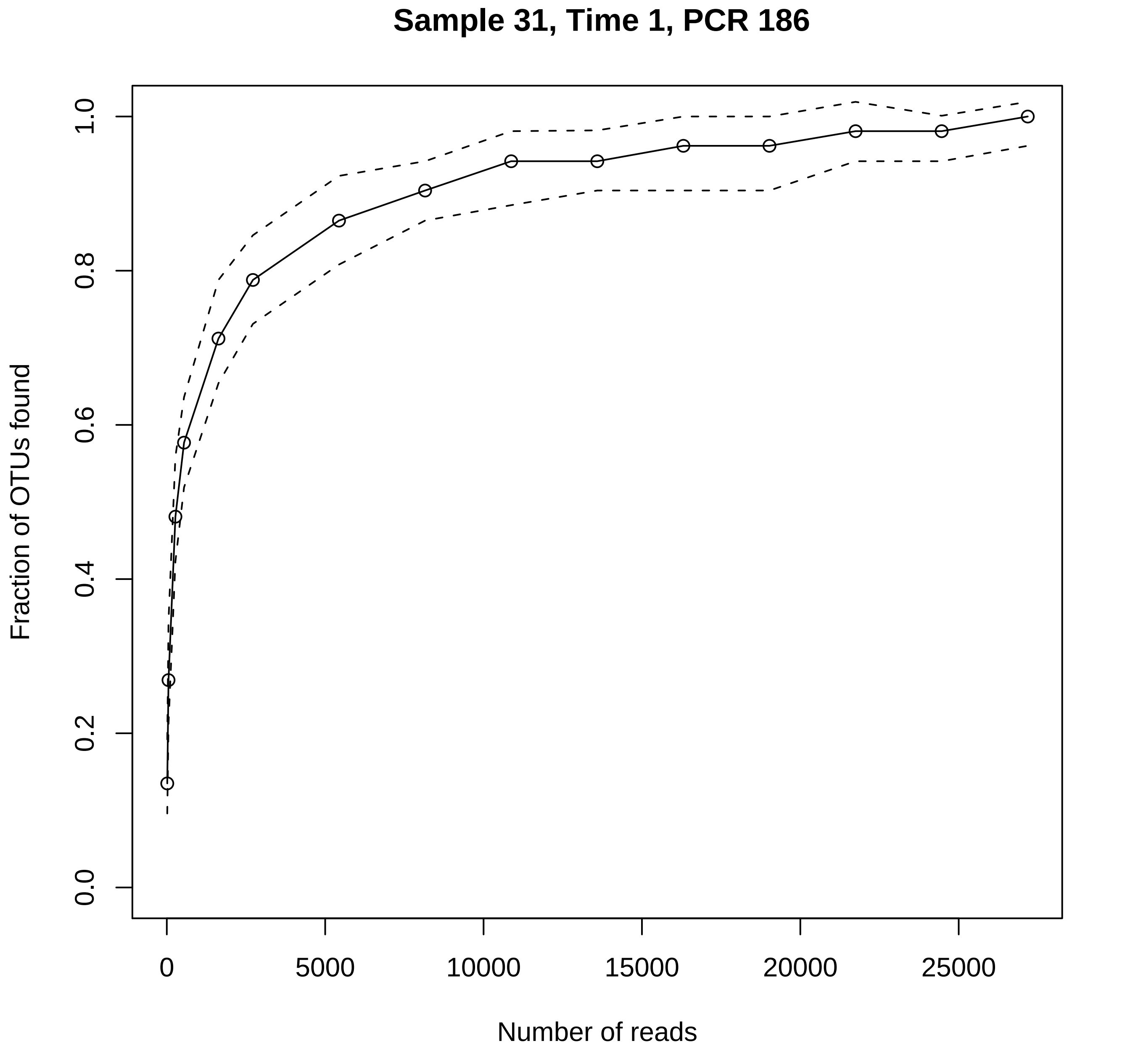
Sample 31, Time 1, PCR 186
Fraction of OTUs found
0.0
0.2
0.4
0.6
0.8
1.0
0
5000
10000
15000
20000
25000
Number of reads

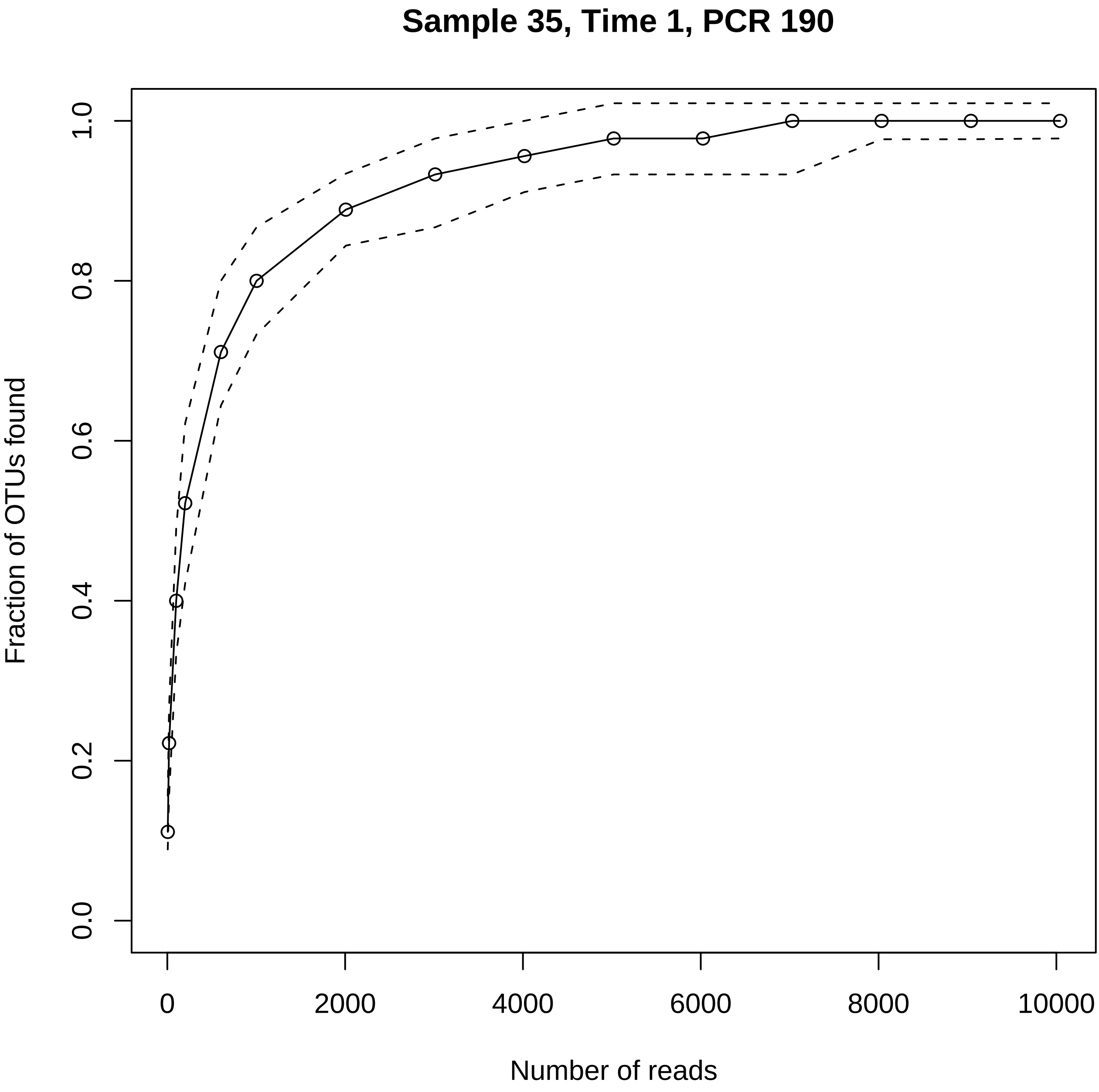

Sample 35, Time 1, PCR 190
Fraction of OTUs found
0.0
0.2
0.4
0.6
0.8
1.0
0
2000
4000
6000
8000
10000
Number of reads

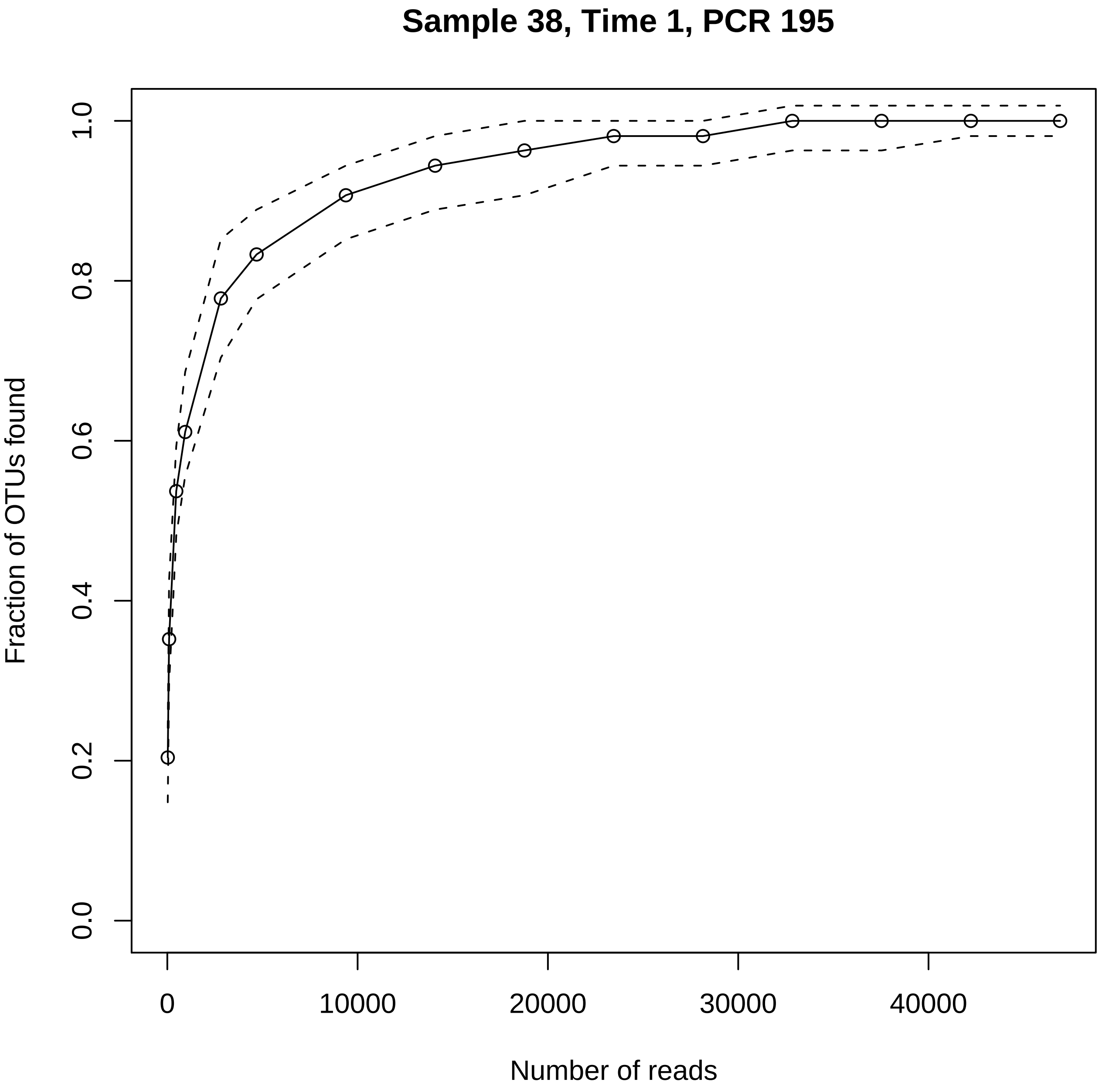
Sample 38, Time 1, PCR 195
Fraction of OTUs found
0.0
0.2
0.4
0.6
0.8
1.0
0
10000
20000
30000
40000
Number of reads

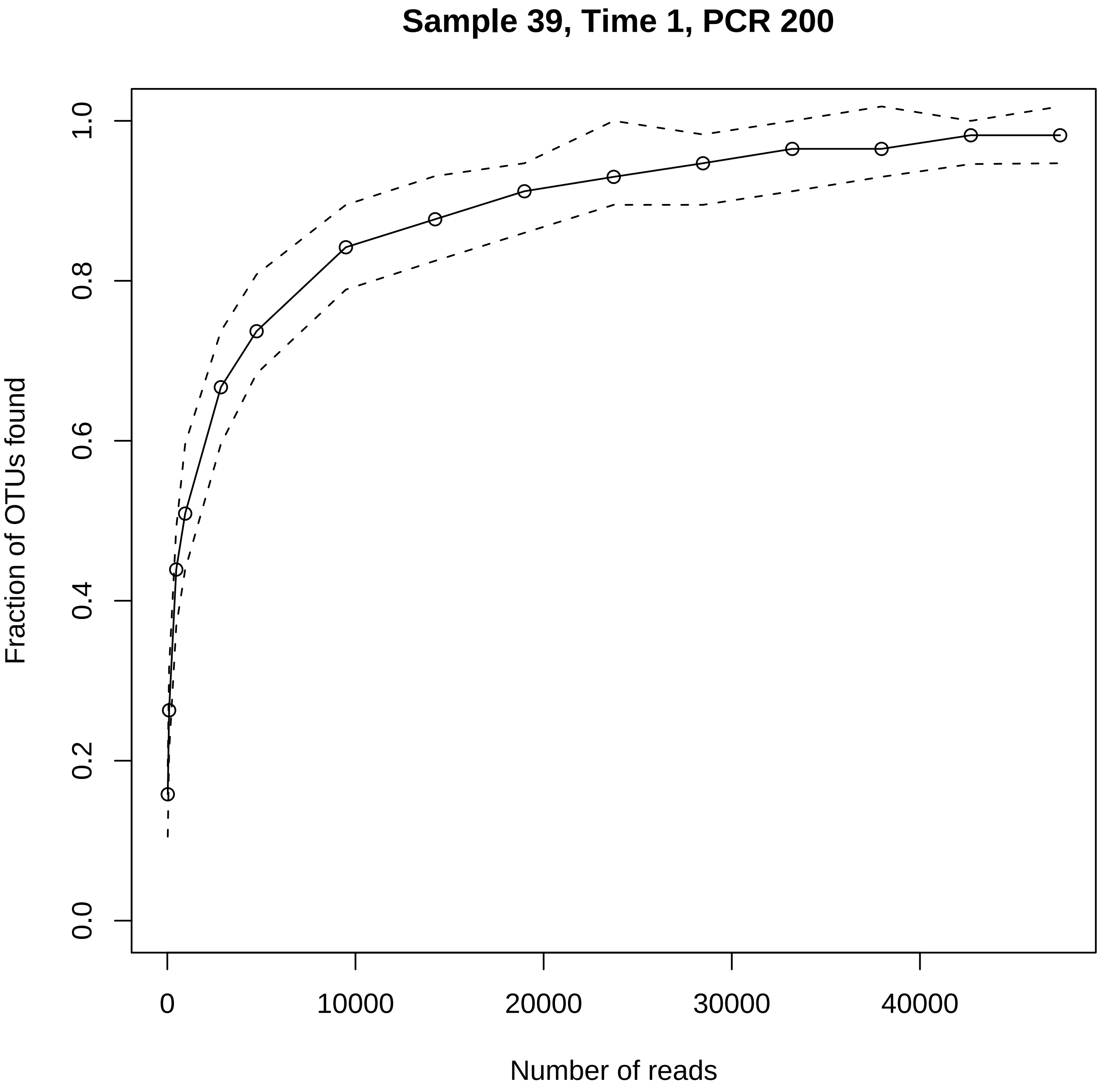
Sample 39, Time 1, PCR 200
Fraction of OTUs found
0.0
0.2
0.4
0.6
0.8
1.0
0
10000
20000
30000
40000
Number of reads

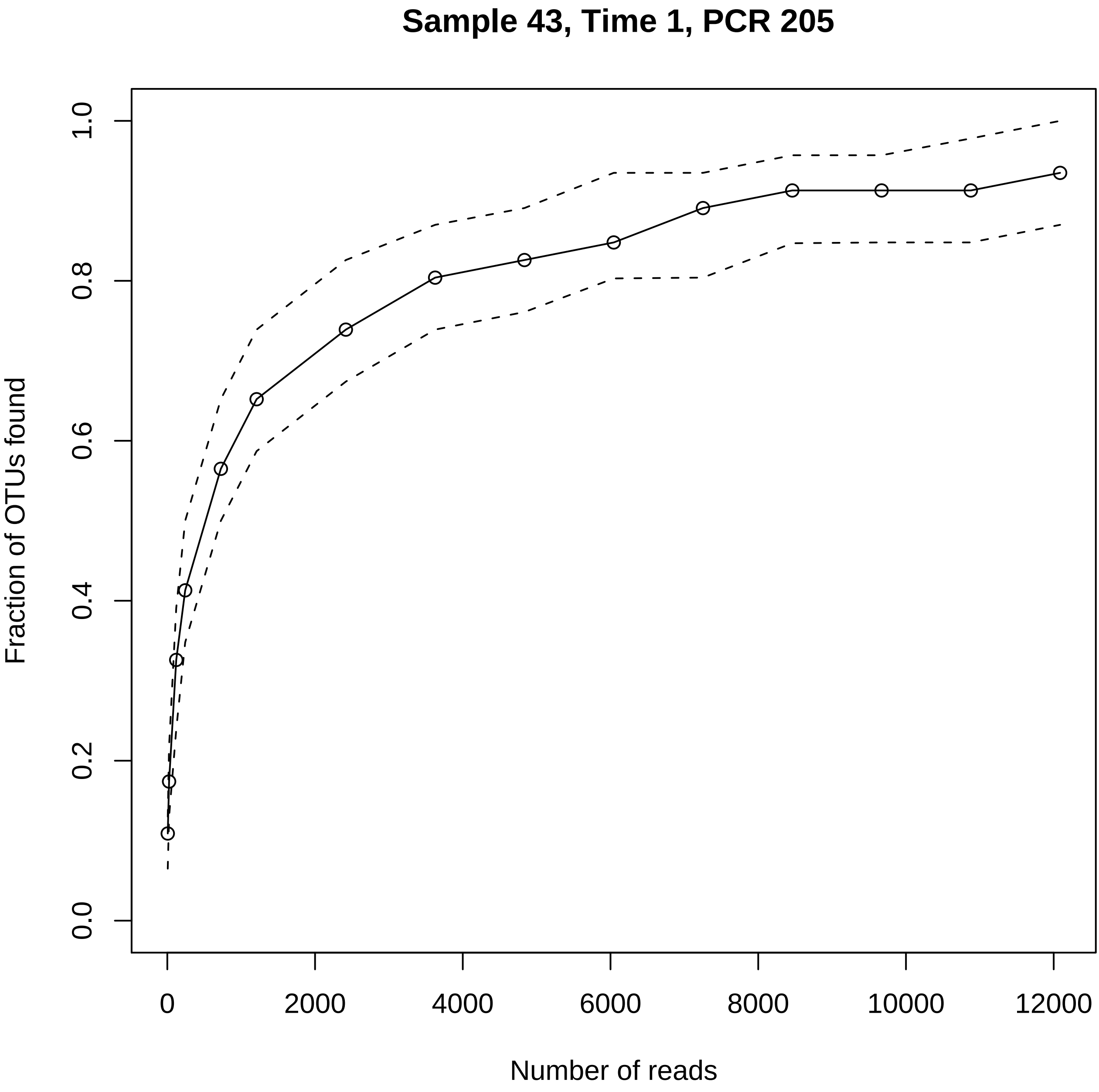

Sample 43, Time 1, PCR 205
Fraction of OTUs found
0.0
0.2
0.4
0.6
0.8
1.0
0
2000
4000
6000
8000
10000
12000
Number of reads

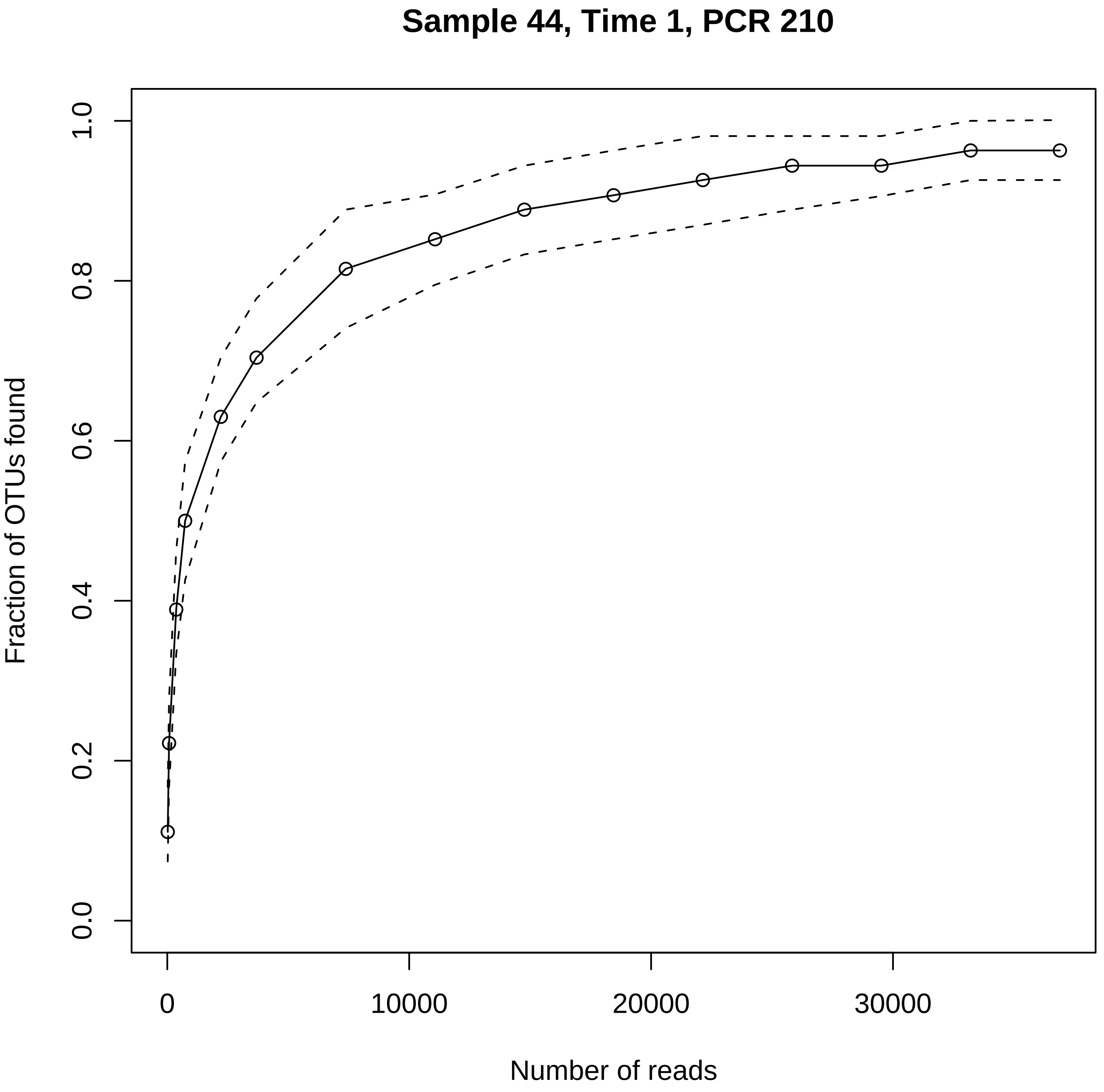
Sample 44, Time 1, PCR 210
Fraction of OTUs found
0.0
0.2
0.4
0.6
0.8
1.0
0
10000
20000
30000
Number of reads

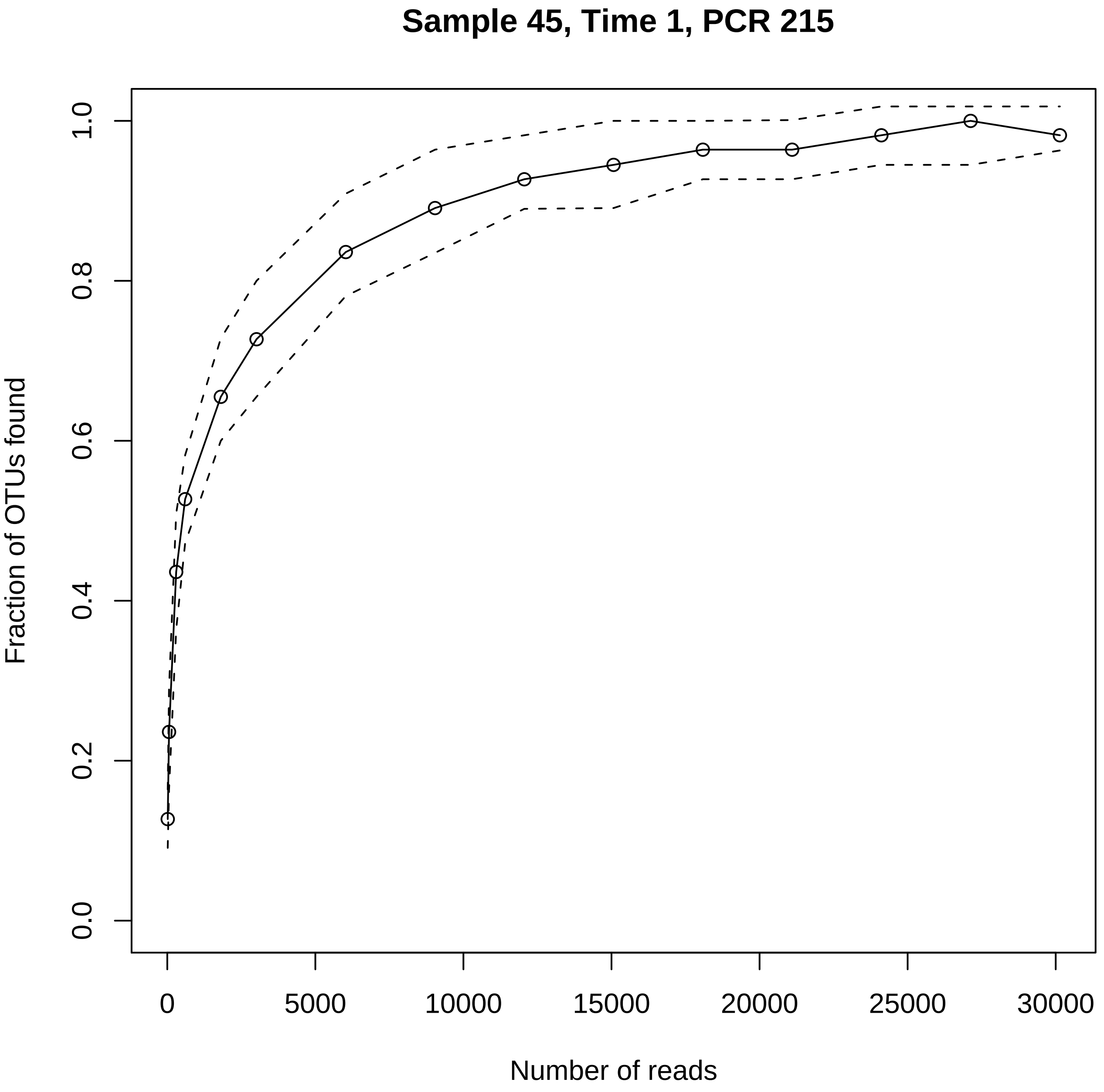
Sample 45, Time 1, PCR 215
Fraction of OTUs found
Number of reads
0.0
0.2
0.4
0.6
0.8
1.0
0
5000
10000
15000
20000
25000
30000

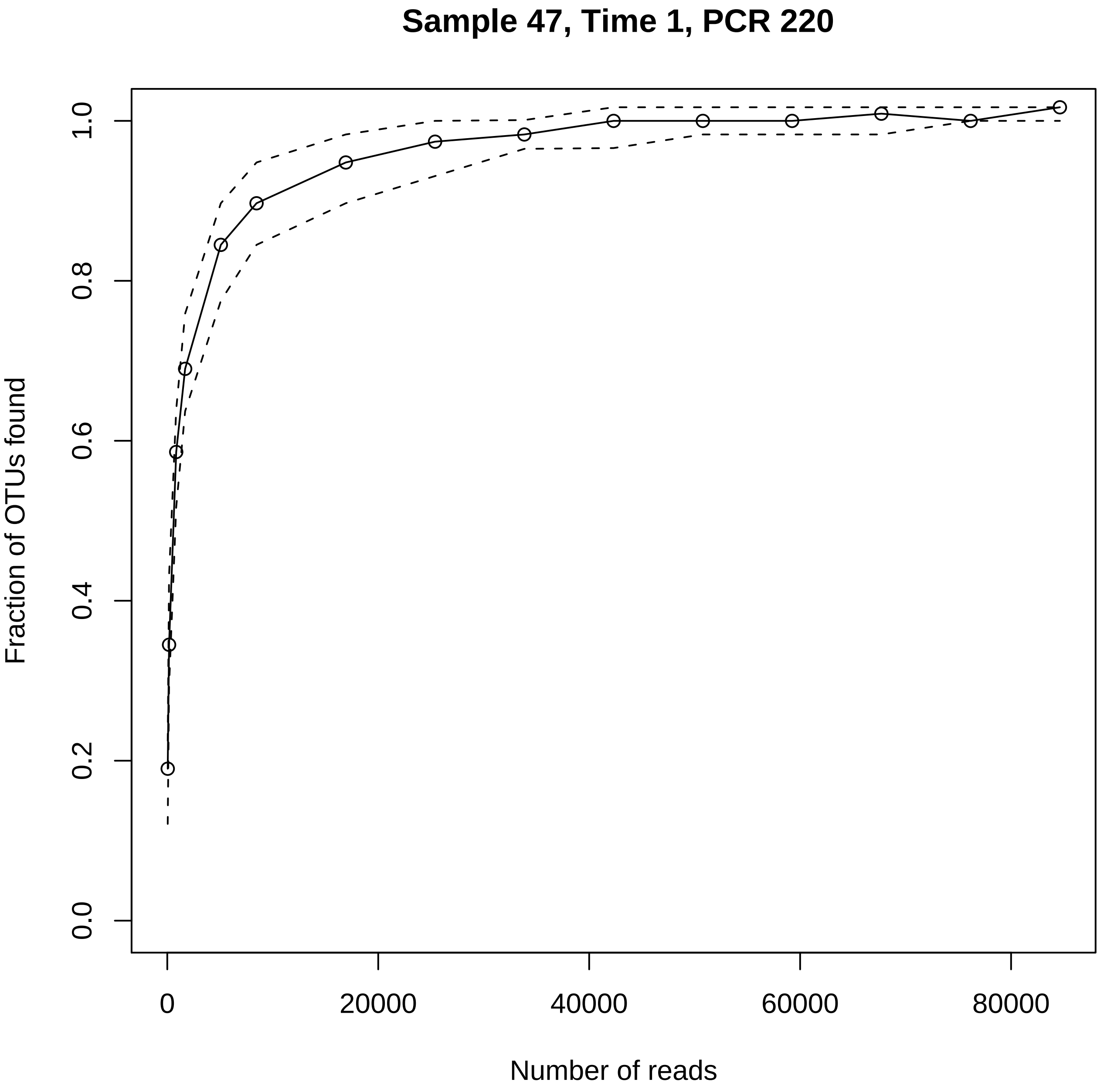

Sample 47, Time 1, PCR 220
Fraction of OTUs found
0.0
0.2
0.4
0.6
0.8
1.0
0
20000
40000
60000
80000
Number of reads

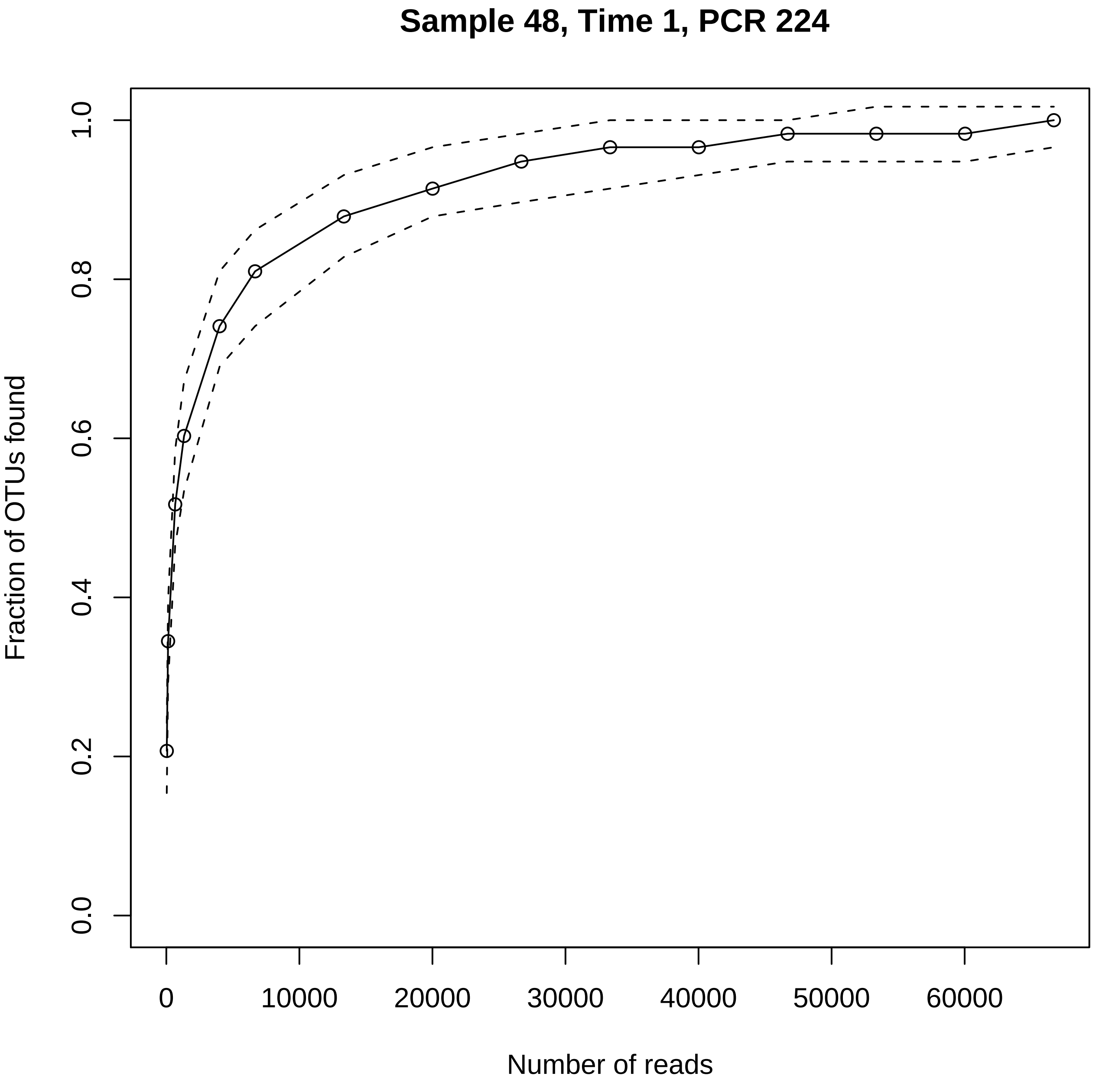
Sample 48, Time 1, PCR 224
Fraction of OTUs found
0.0
0.2
0.4
0.6
0.8
1.0
0
10000
20000
30000
40000
50000
60000
Number of reads

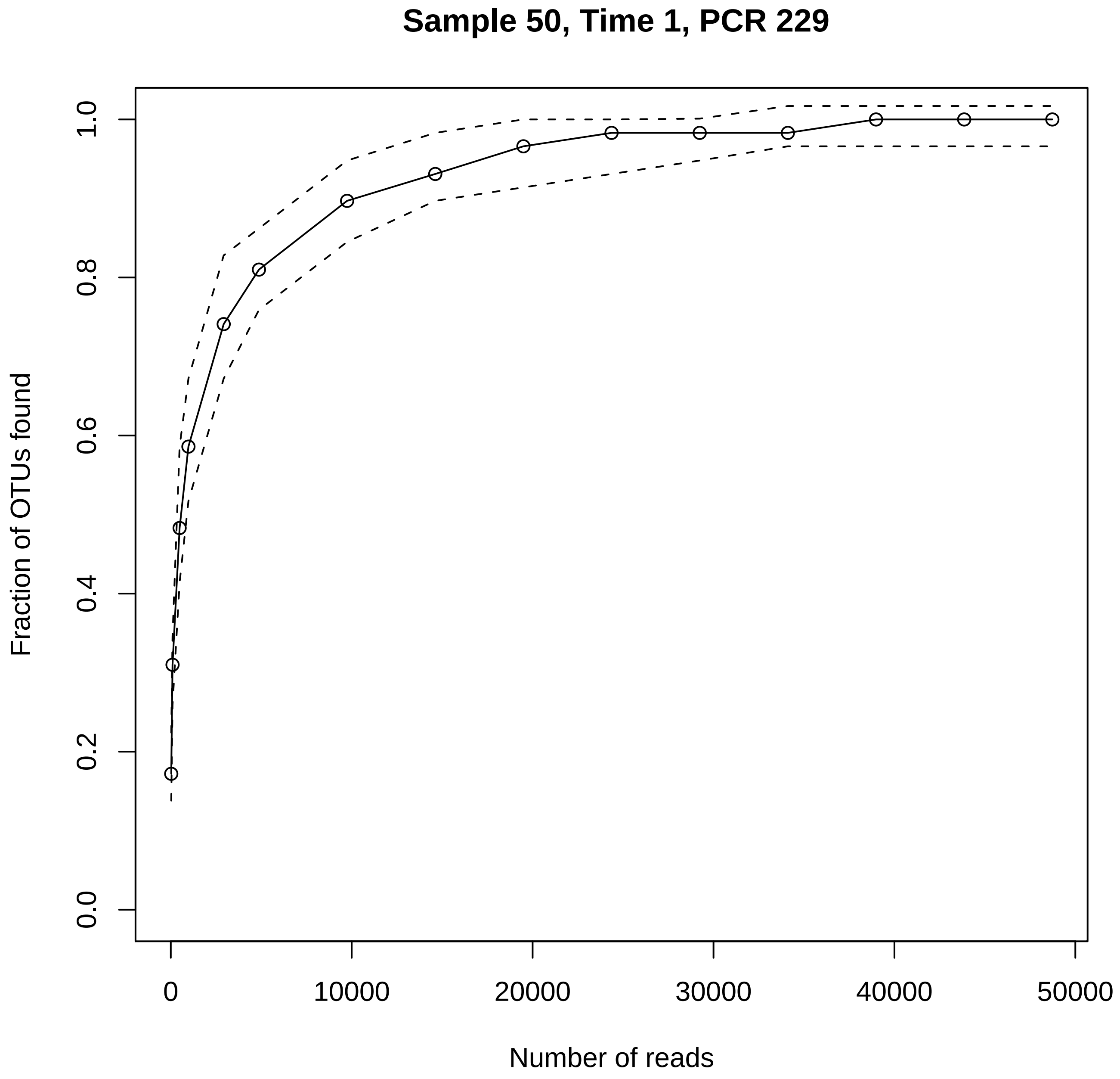
Sample 50, Time 1, PCR 229
Fraction of OTUs found
0.0
0.2
0.4
0.6
0.8
1.0
Number of reads
0
10000
20000
30000
40000
50000

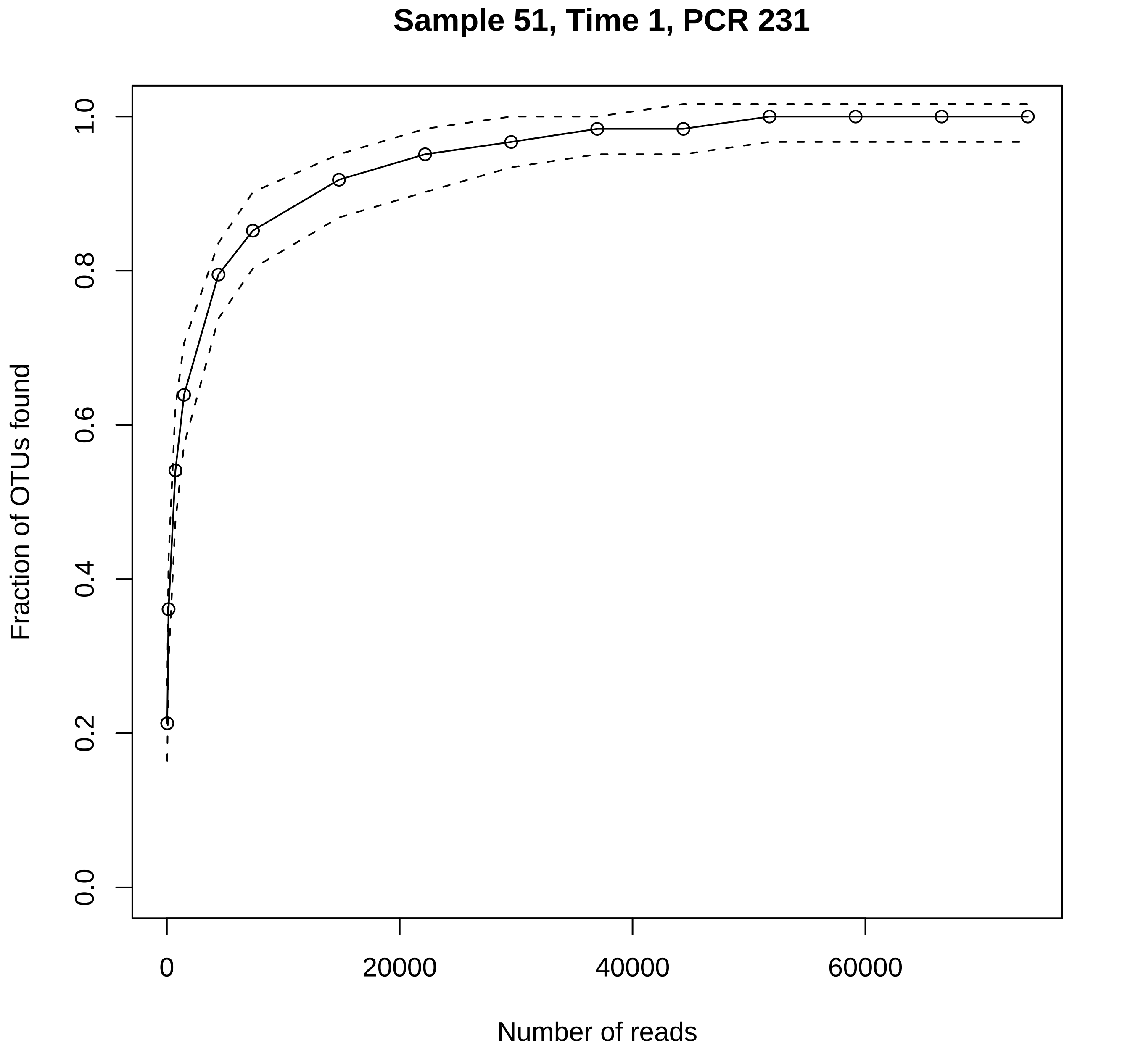
Sample 51, Time 1, PCR 231
Fraction of OTUs found
0.0
0.2
0.4
0.6
0.8
1.0
0
20000
40000
60000
Number of reads

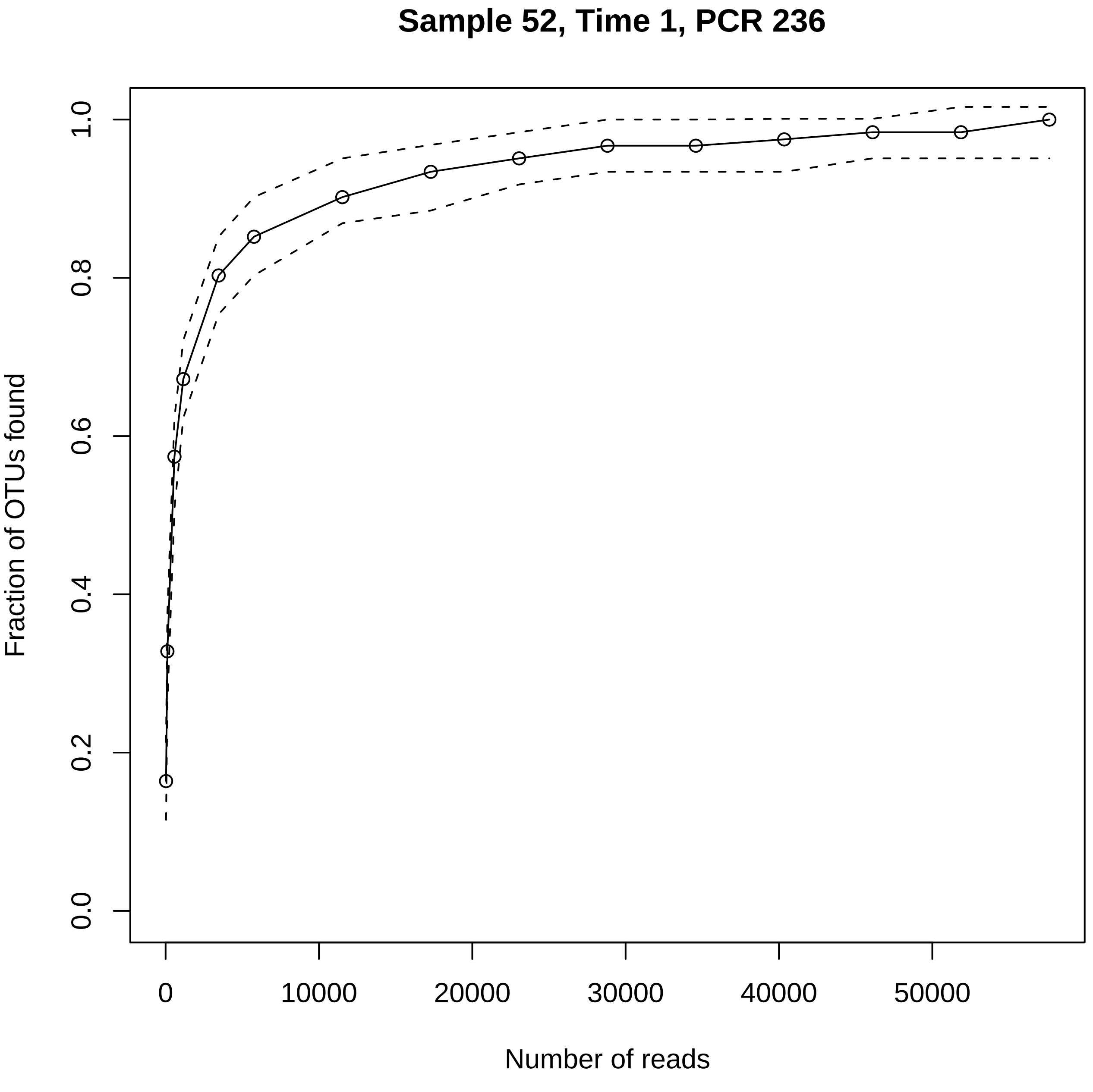

Sample 52, Time 1, PCR 236
Fraction of OTUs found
Number of reads
0.0
0.2
0.4
0.6
0.8
1.0
0
10000
20000
30000
40000
50000

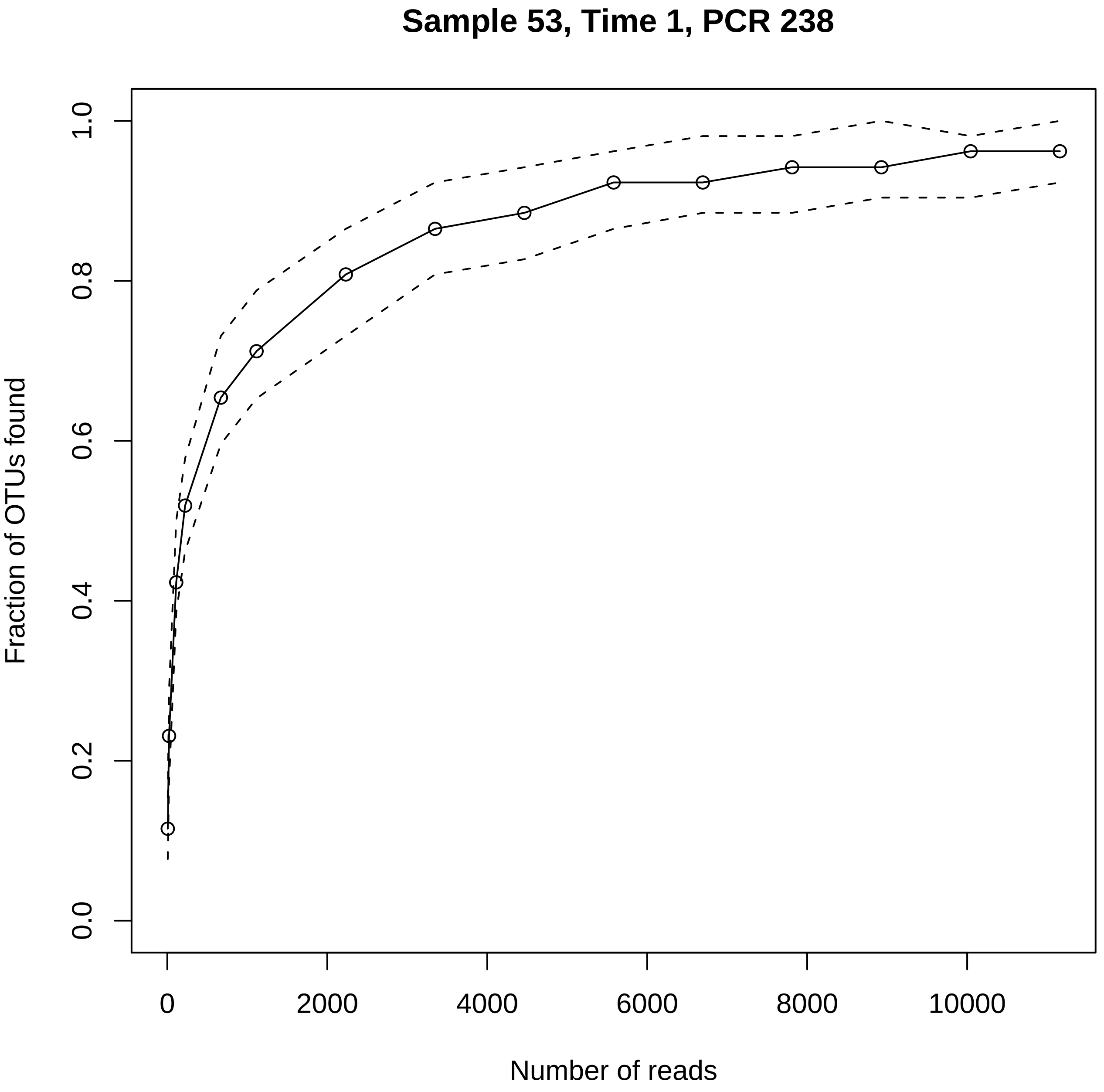

Sample 53, Time 1, PCR 238
Fraction of OTUs found
0.0
0.2
0.4
0.6
0.8
1.0
0
2000
4000
6000
8000
10000
Number of reads

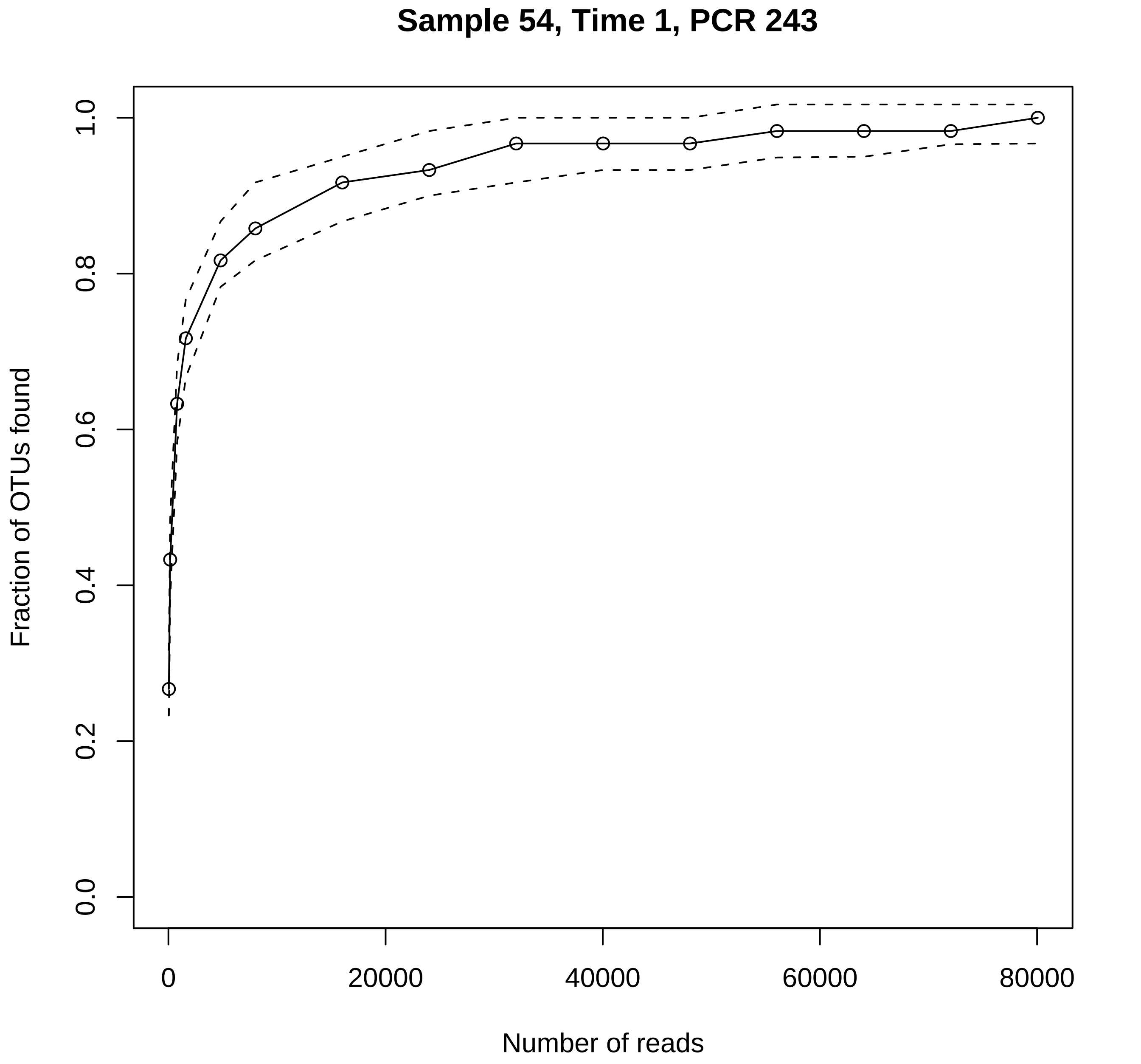
Sample 54, Time 1, PCR 243
Fraction of OTUs found
0.0
0.2
0.4
0.6
0.8
1.0
0
20000
40000
60000
80000
Number of reads

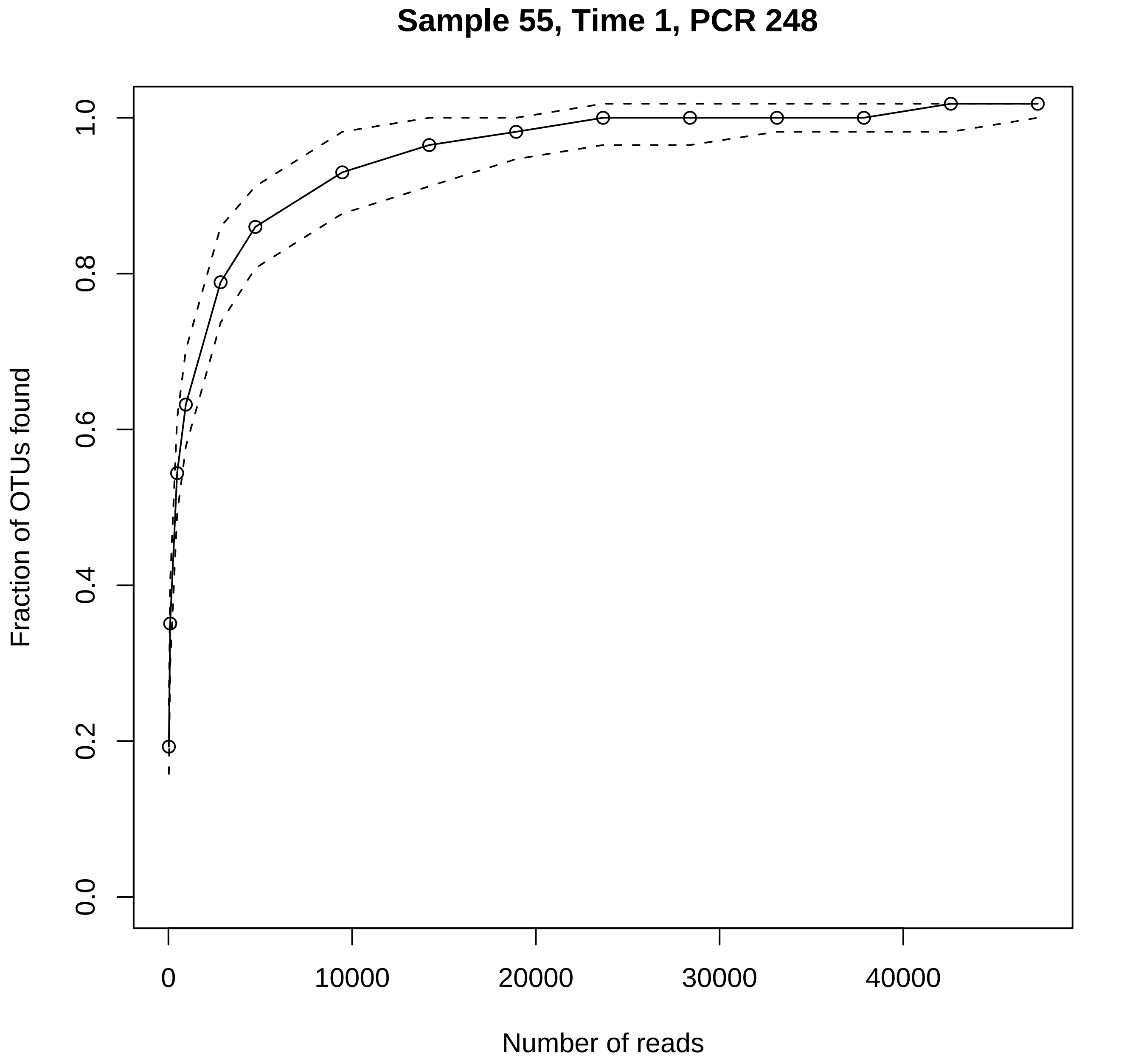
Sample 55, Time 1, PCR 248
Fraction of OTUs found
0.0
0.2
0.4
0.6
0.8
1.0
0
10000
20000
30000
40000
Number of reads

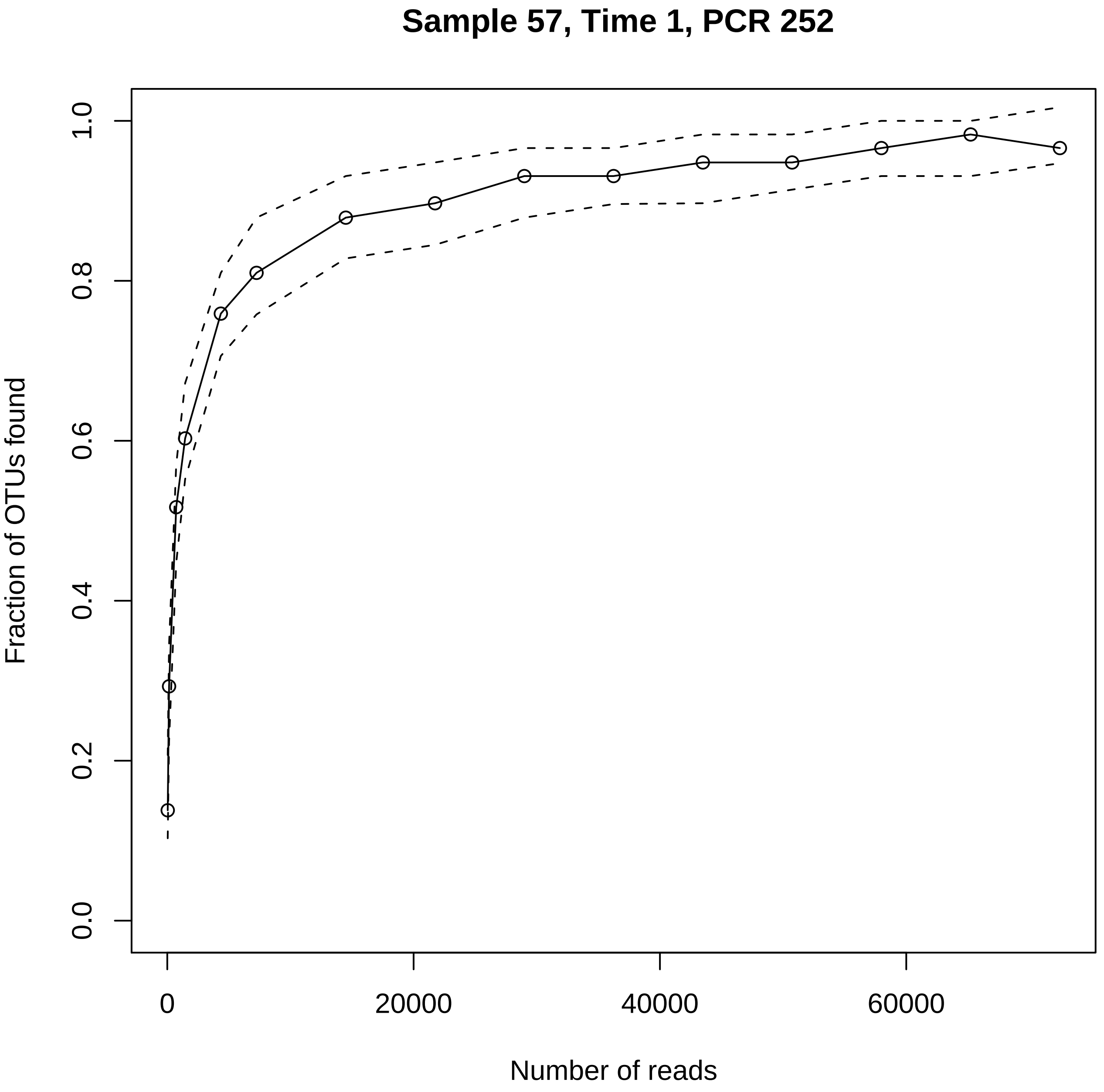
Sample 57, Time 1, PCR 252
Fraction of OTUs found
0.0
0.2
0.4
0.6
0.8
1.0
0
20000
40000
60000
Number of reads

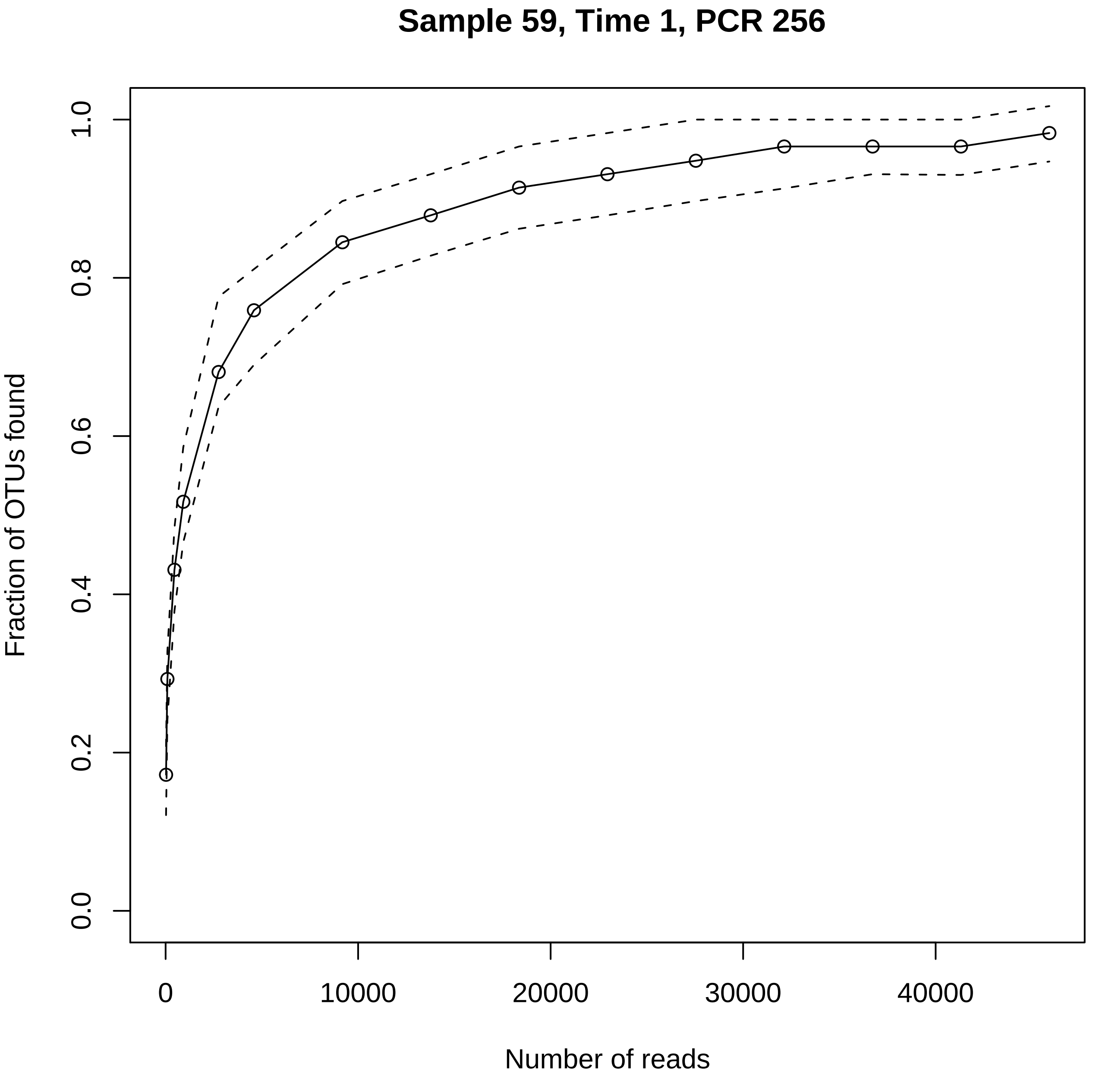

Sample 59, Time 1, PCR 256
Fraction of OTUs found
Number of reads
0.0
0.2
0.4
0.6
0.8
1.0
0
10000
20000
30000
40000

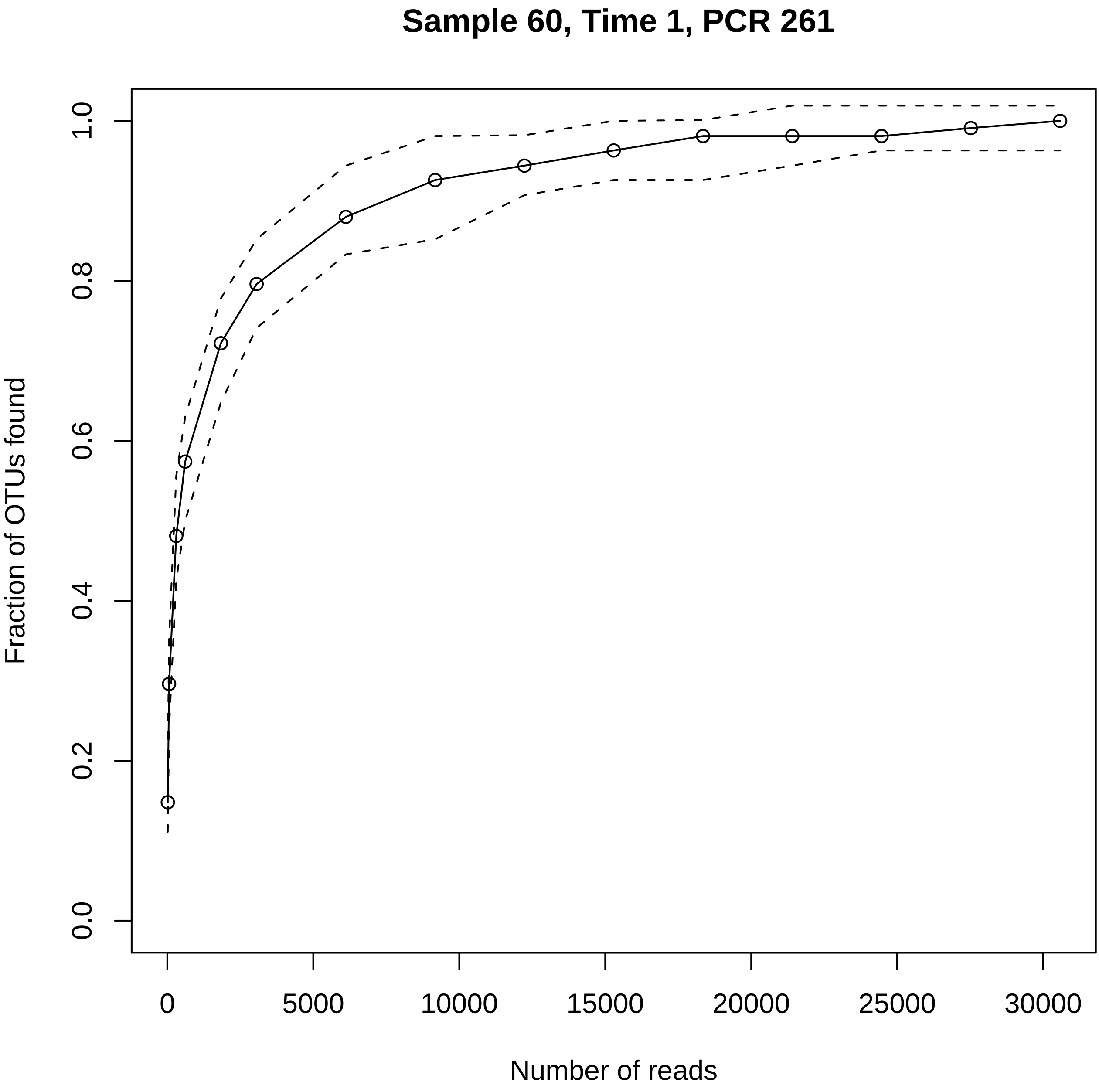
Sample 60, Time 1, PCR 261
Fraction of OTUs found
0.0
0.2
0.4
0.6
0.8
1.0
0
5000
10000
15000
20000
25000
30000
Number of reads

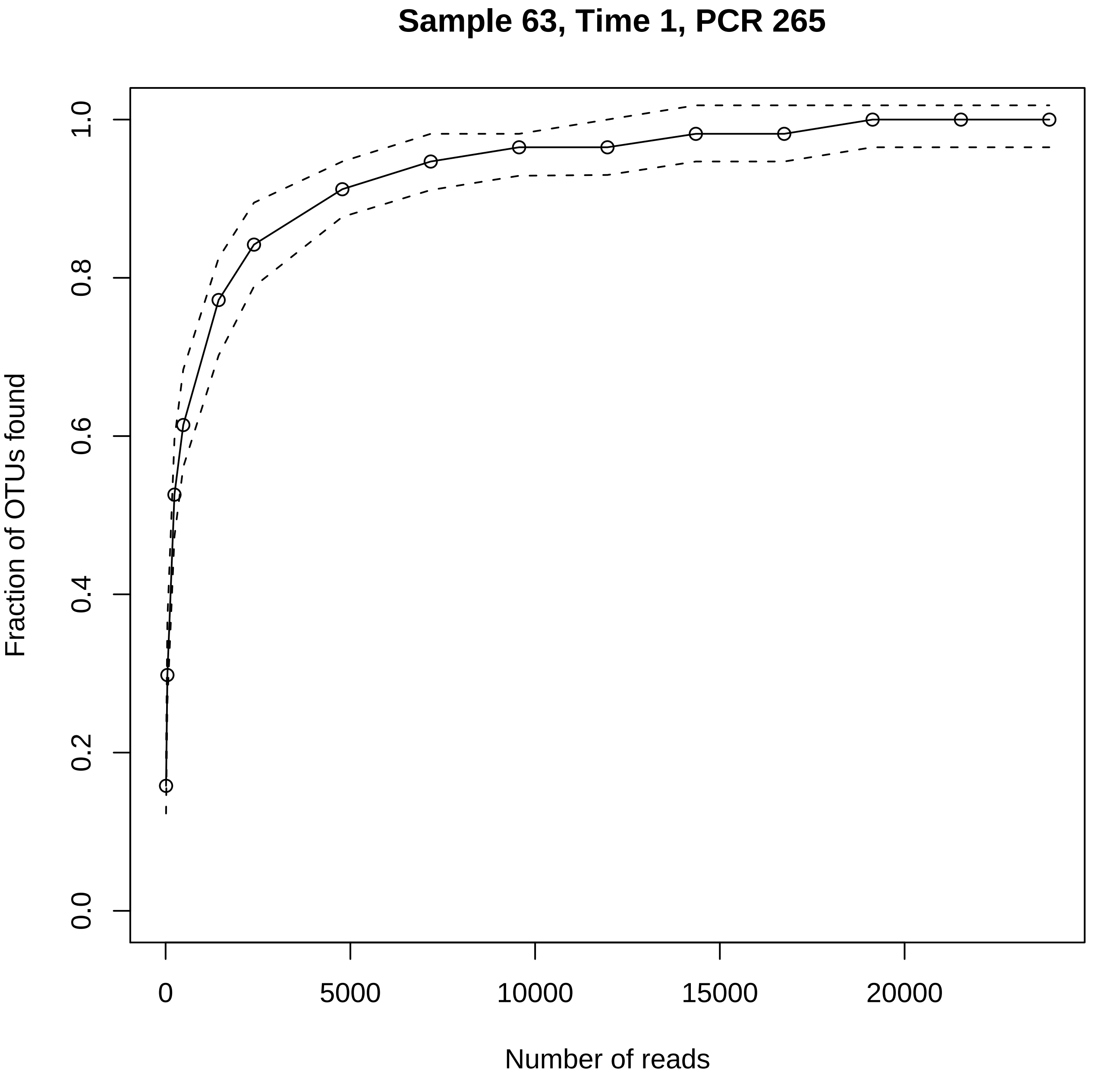

Sample 63, Time 1, PCR 265
Fraction of OTUs found
0.0
0.2
0.4
0.6
0.8
1.0
0
5000
10000
15000
20000
Number of reads

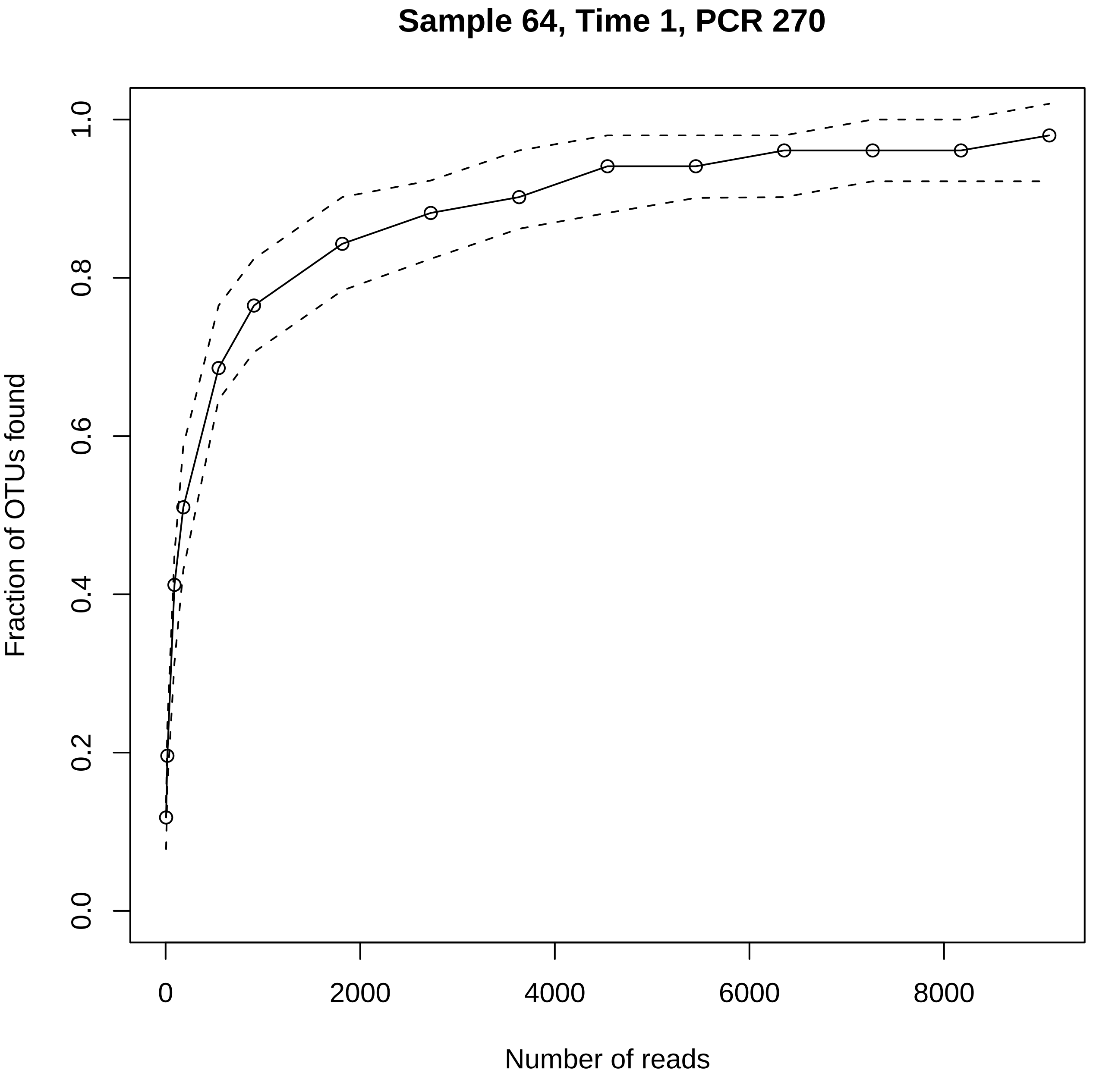
Sample 64, Time 1, PCR 270
Fraction of OTUs found
0.0
0.2
0.4
0.6
0.8
1.0
0
2000
4000
6000
8000
Number of reads

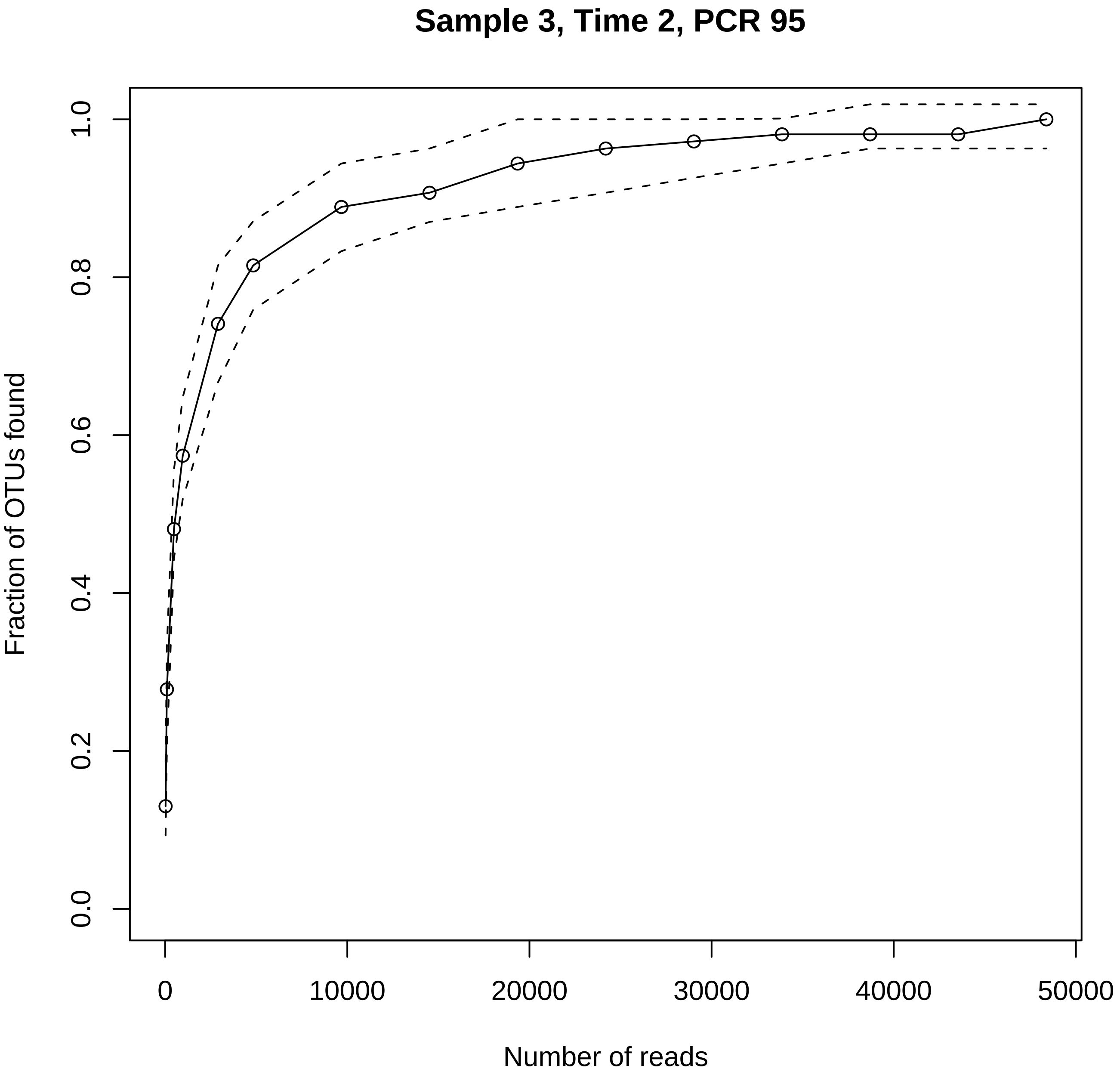
Sample 3, Time 2, PCR 95
Fraction of OTUs found
0.0
0.2
0.4
0.6
0.8
1.0
0
10000
20000
30000
40000
50000
Number of reads

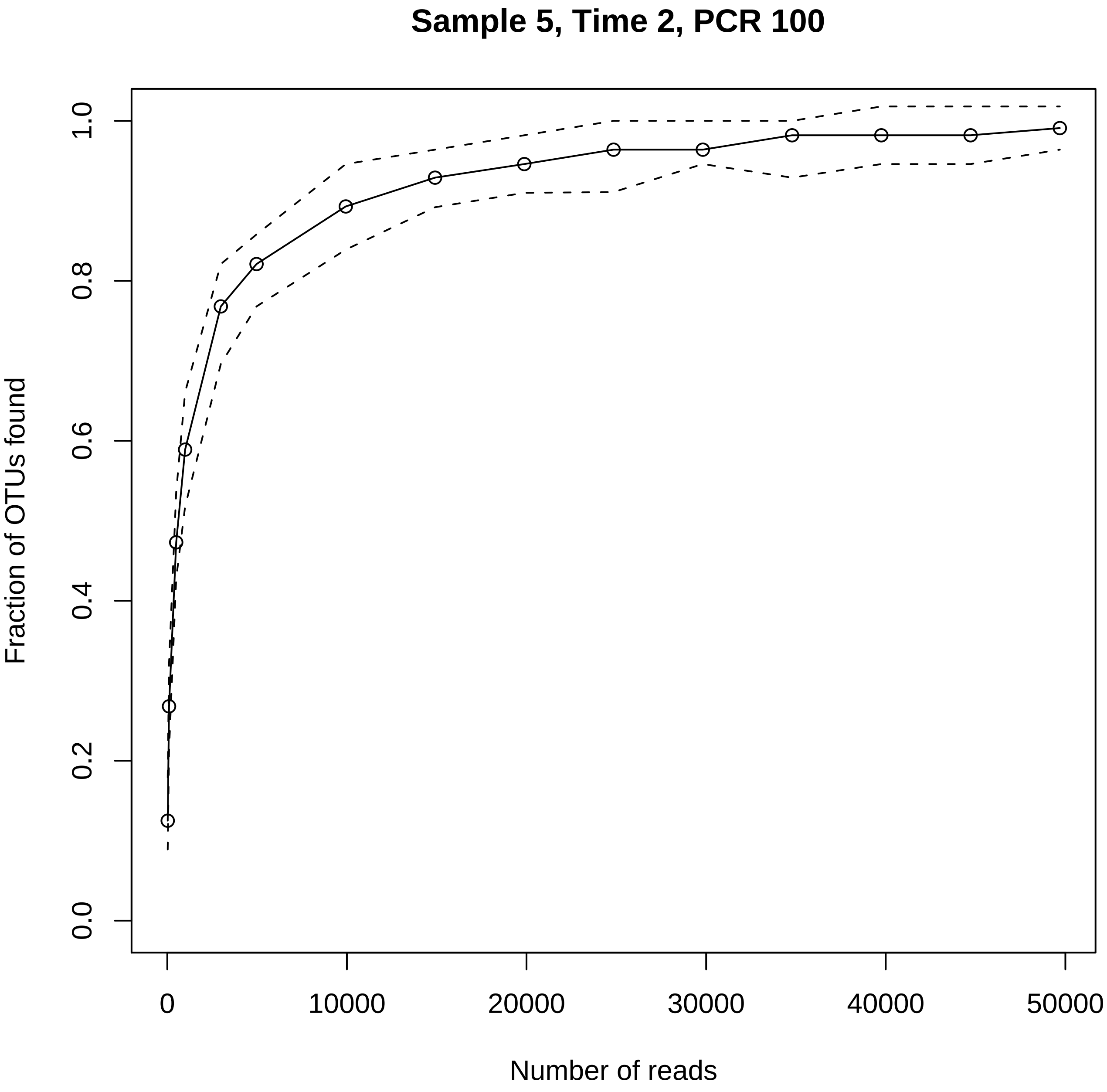

Sample 5, Time 2, PCR 100
Fraction of OTUs found
0.0
0.2
0.4
0.6
0.8
1.0
0
10000
20000
30000
40000
50000
Number of reads

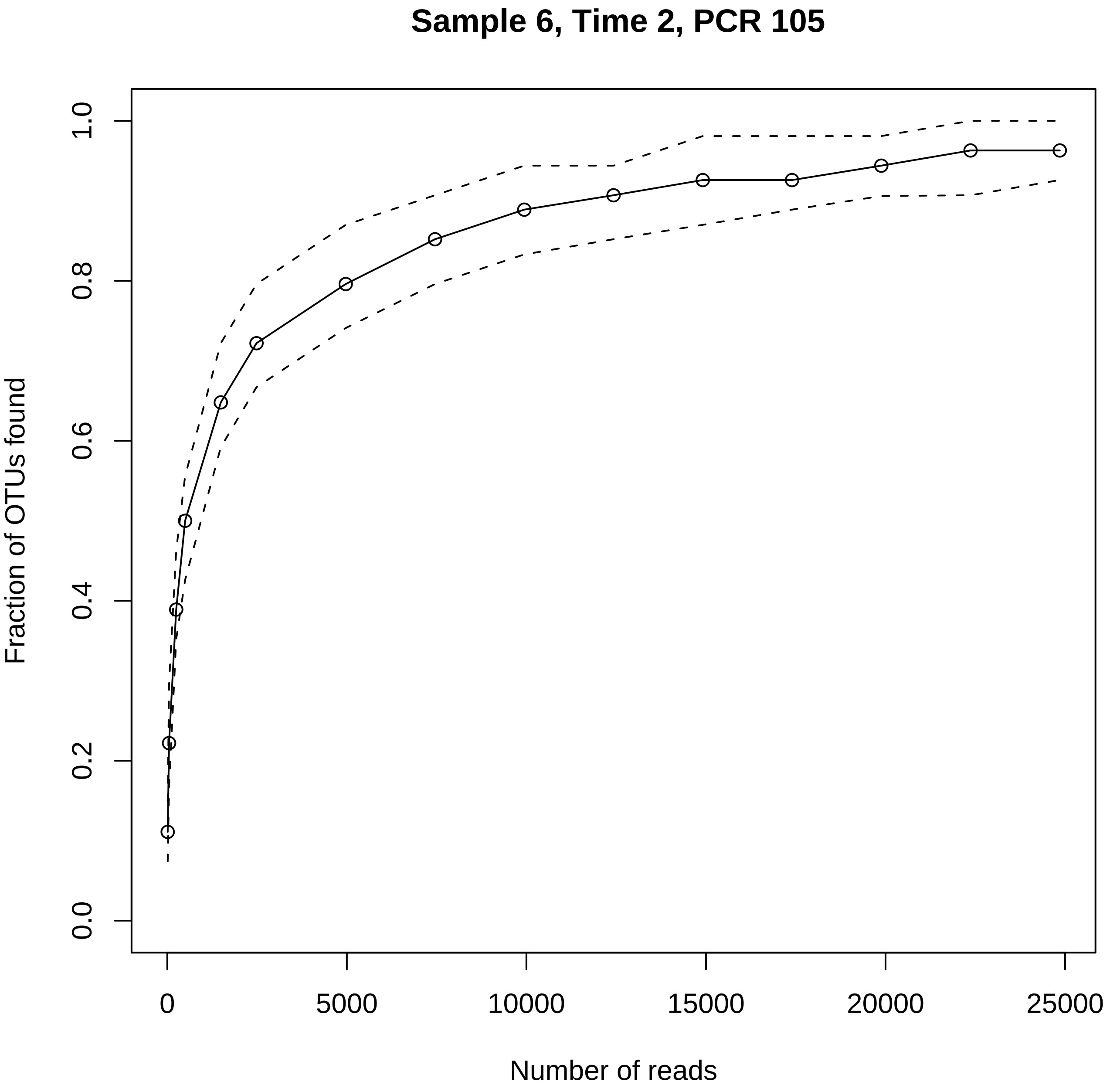

Sample 6, Time 2, PCR 105
Fraction of OTUs found
0.0
0.2
0.4
0.6
0.8
1.0
0
5000
10000
15000
20000
25000
Number of reads

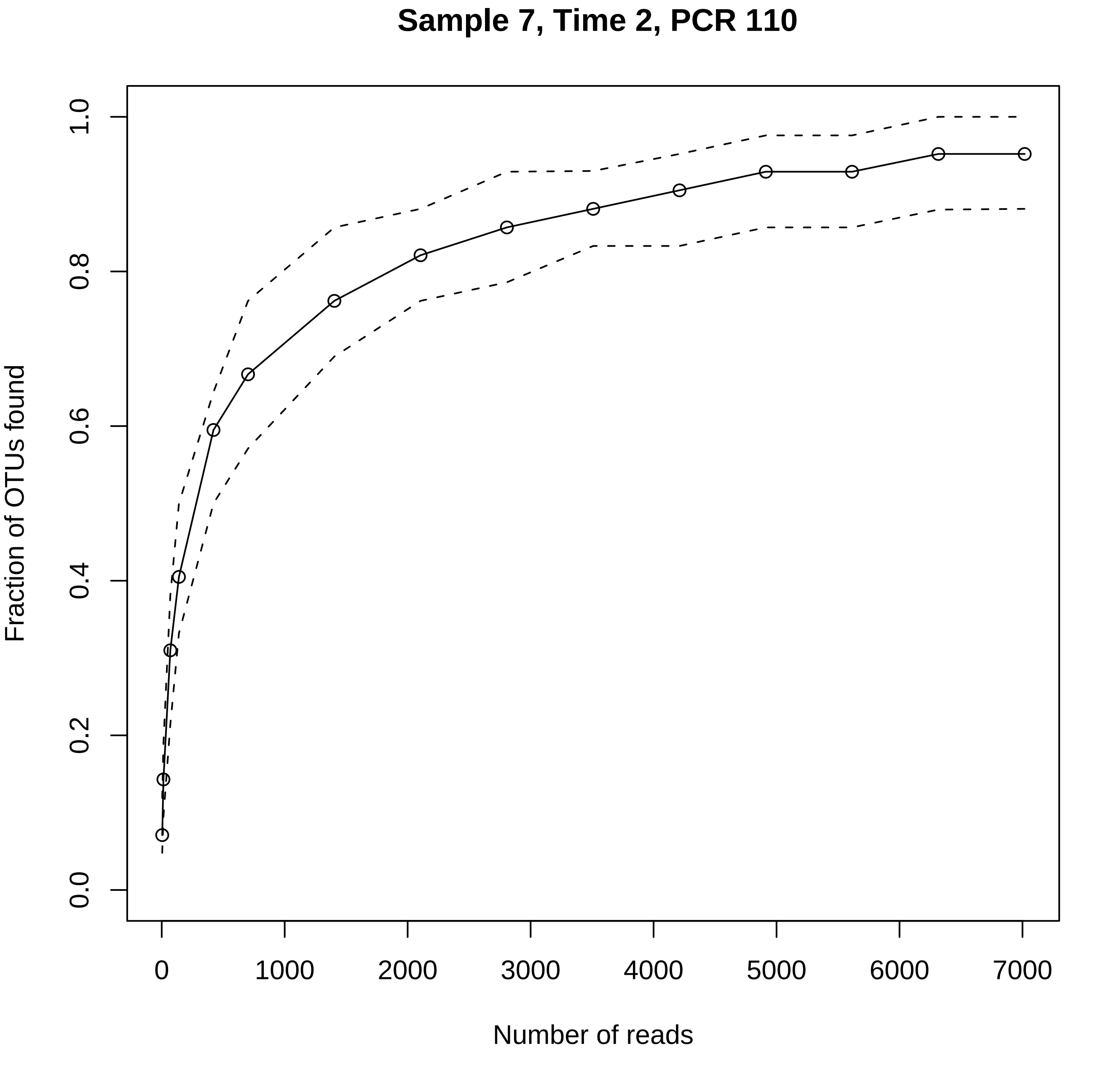
Sample 7, Time 2, PCR 110
Fraction of OTUs found
0.0
0.2
0.4
0.6
0.8
1.0
0
1000
2000
3000
4000
5000
6000
7000
Number of reads

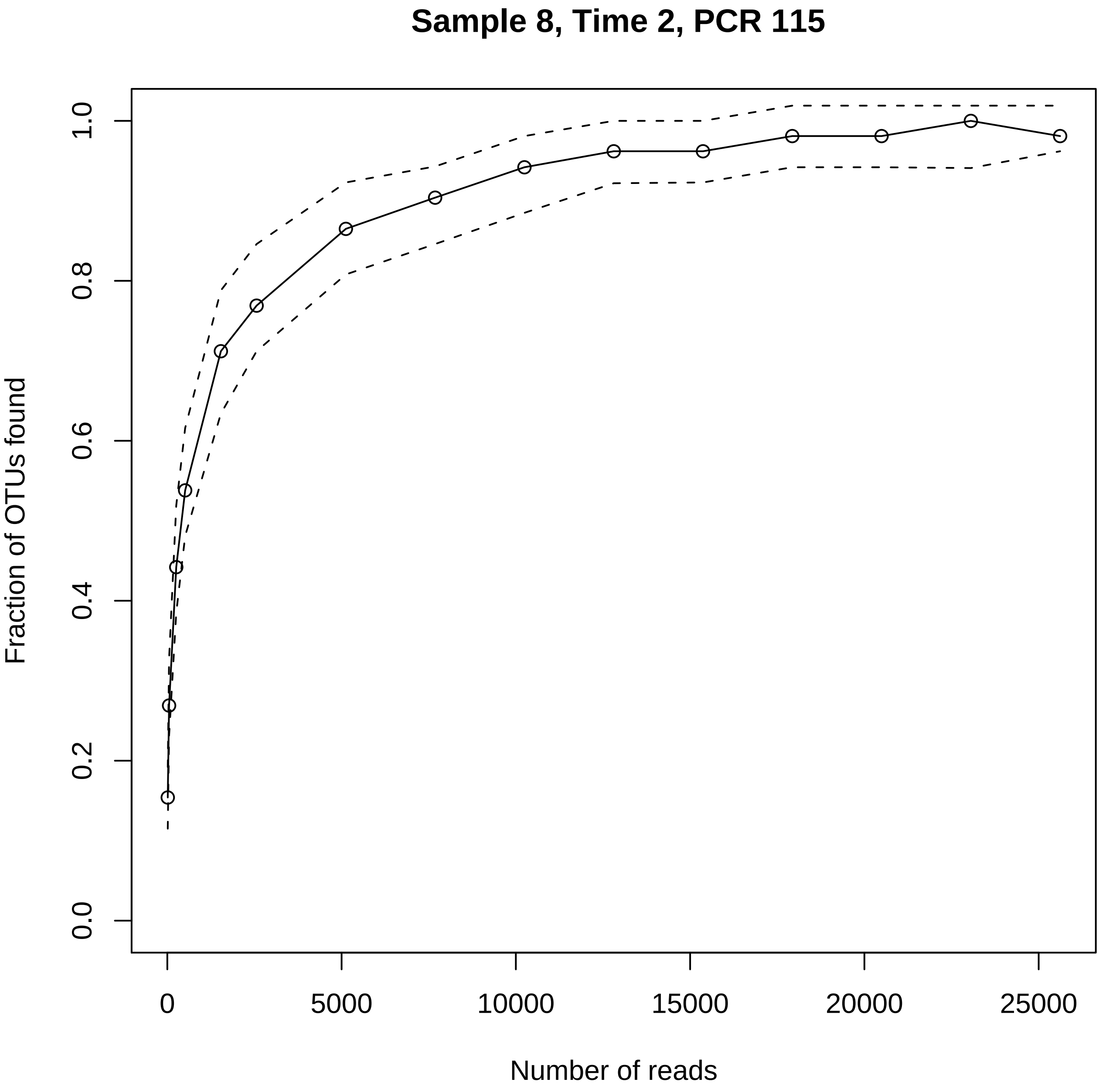

Sample 8, Time 2, PCR 115
Fraction of OTUs found
0.0
0.2
0.4
0.6
0.8
1.0
0
5000
10000
15000
20000
25000
Number of reads

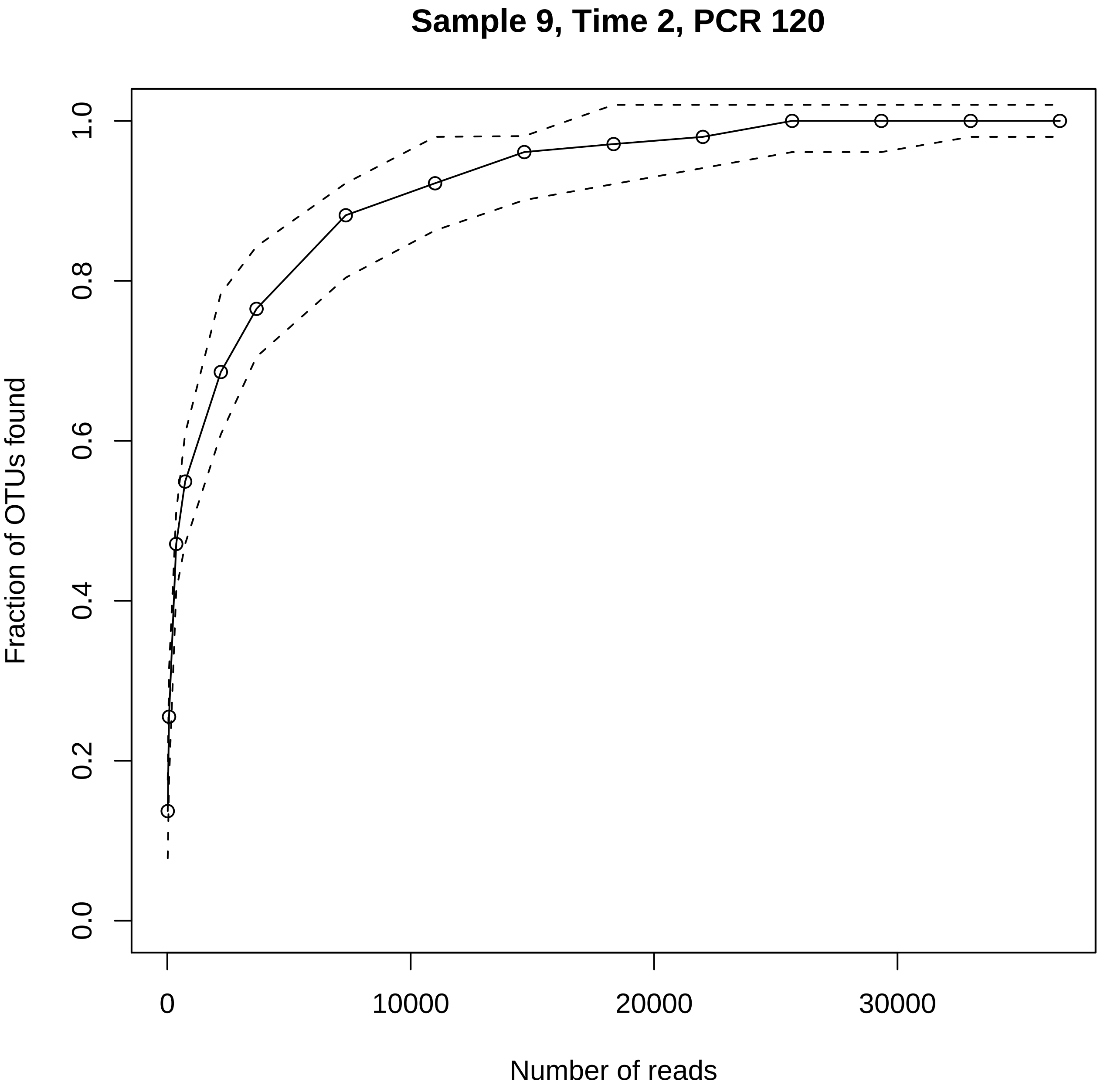
Sample 9, Time 2, PCR 120
Fraction of OTUs found
0.0
0.2
0.4
0.6
0.8
1.0
0
10000
20000
30000
Number of reads

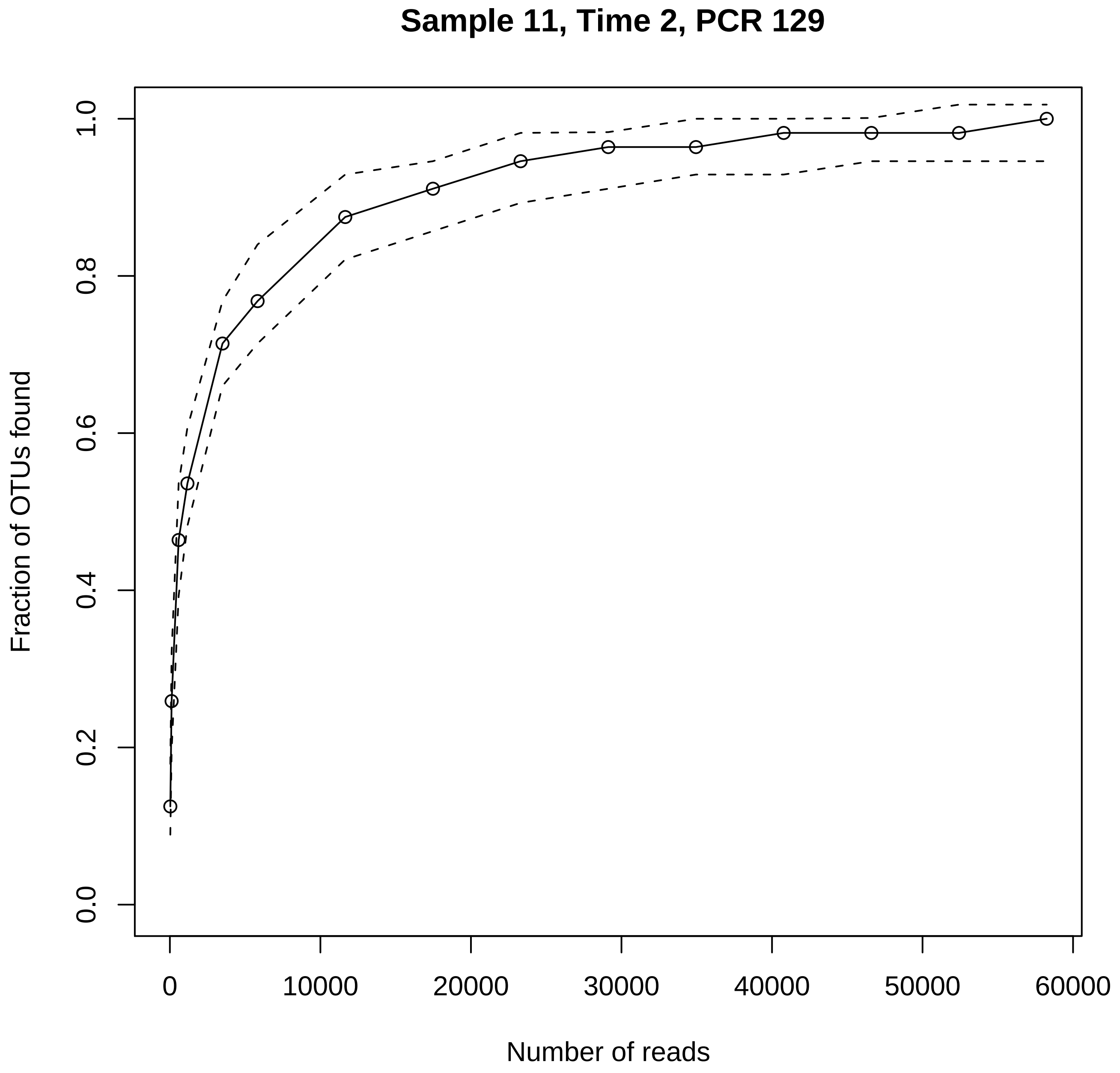
Sample 11, Time 2, PCR 129
Fraction of OTUs found
0.0
0.2
0.4
0.6
0.8
1.0
0
10000
20000
30000
40000
50000
60000
Number of reads

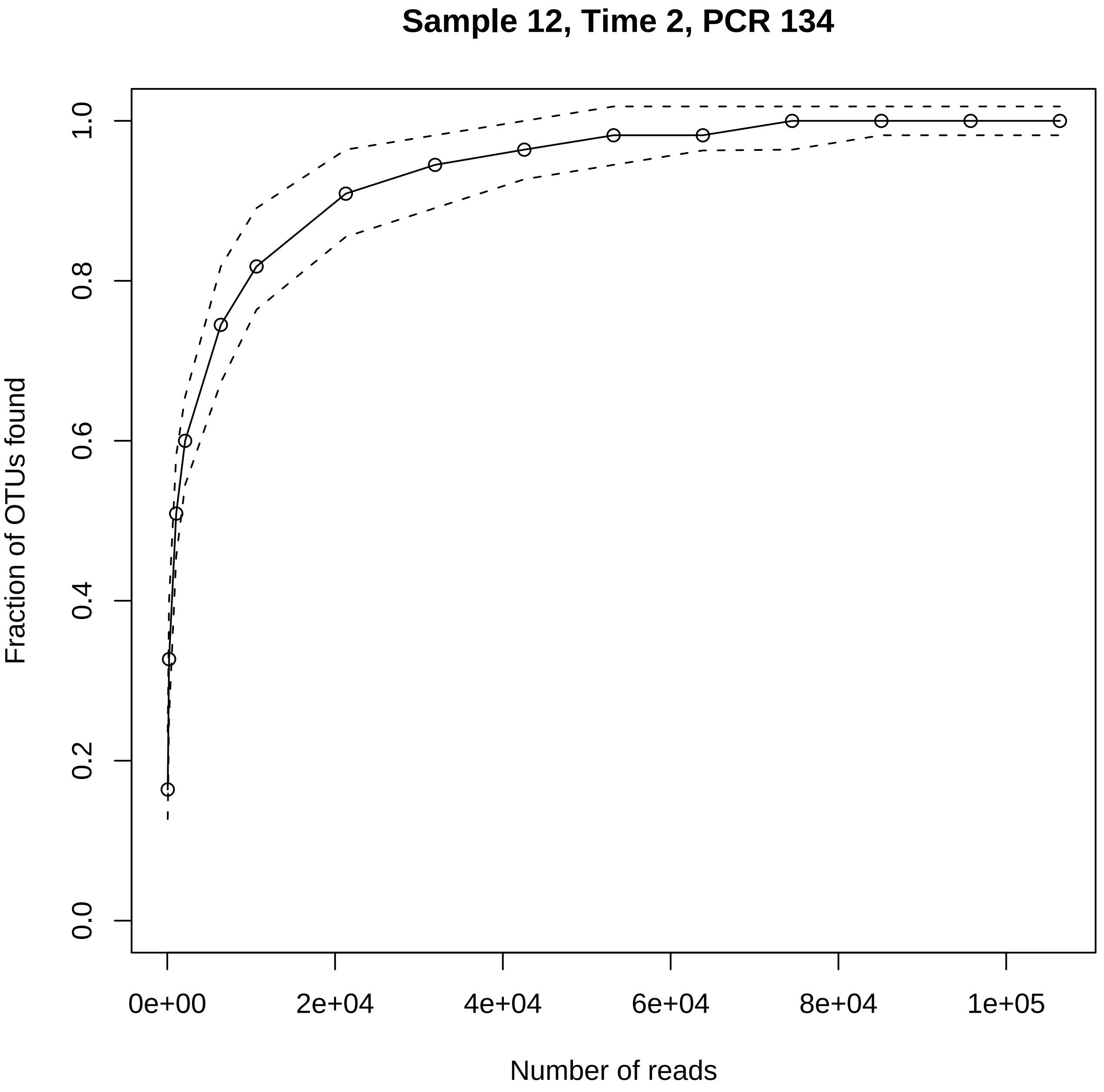

Sample 12, Time 2, PCR 134
Fraction of OTUs found
0.0
0.2
0.4
0.6
0.8
1.0
0e+00
2e+04
4e+04
6e+04
8e+04
1e+05
Number of reads

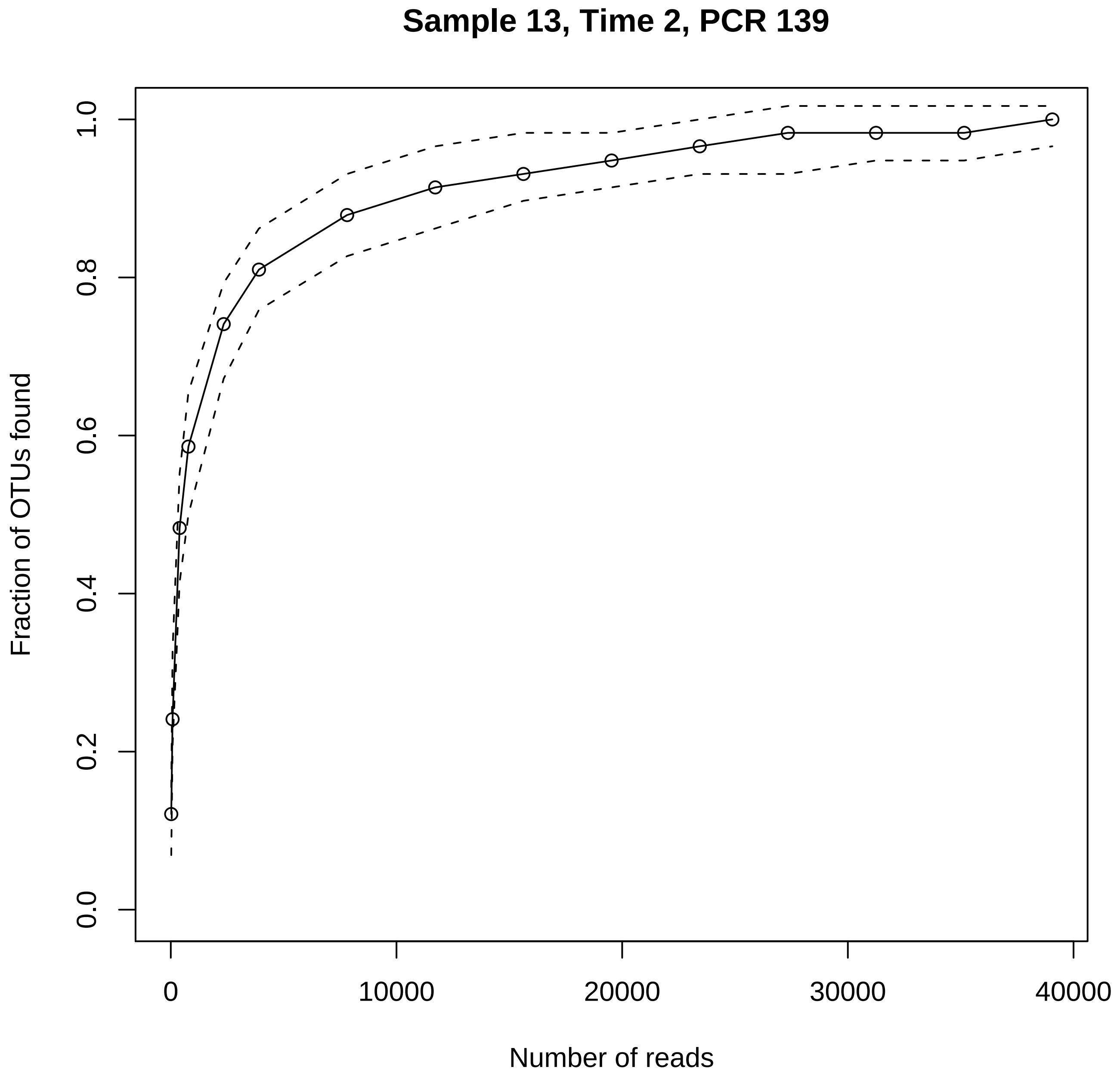
Sample 13, Time 2, PCR 139
Fraction of OTUs found
Number of reads
0.0
0.2
0.4
0.6
0.8
1.0
0
10000
20000
30000
40000

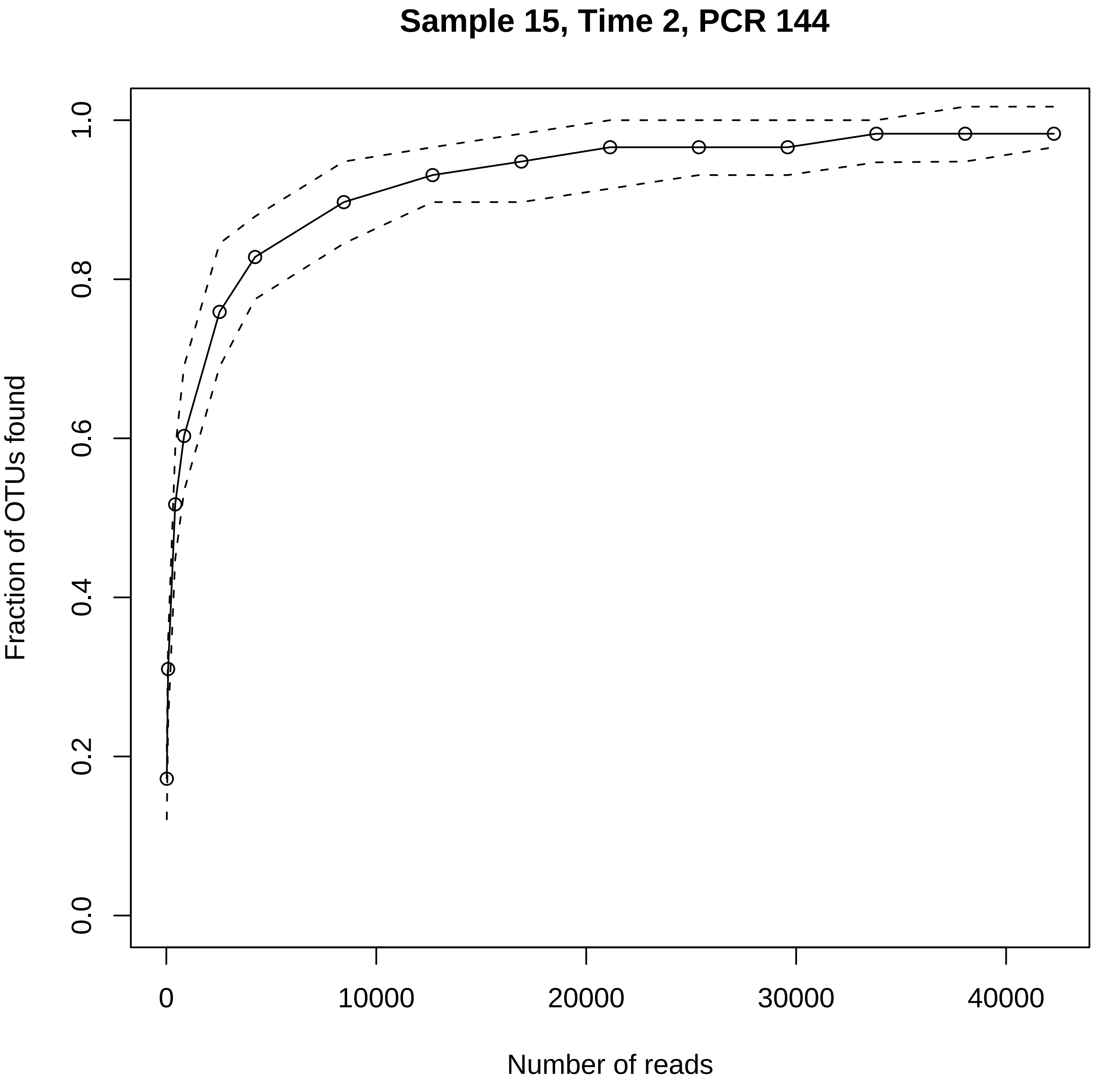

Sample 15, Time 2, PCR 144
Fraction of OTUs found
0.0
0.2
0.4
0.6
0.8
1.0
0
10000
20000
30000
40000
Number of reads

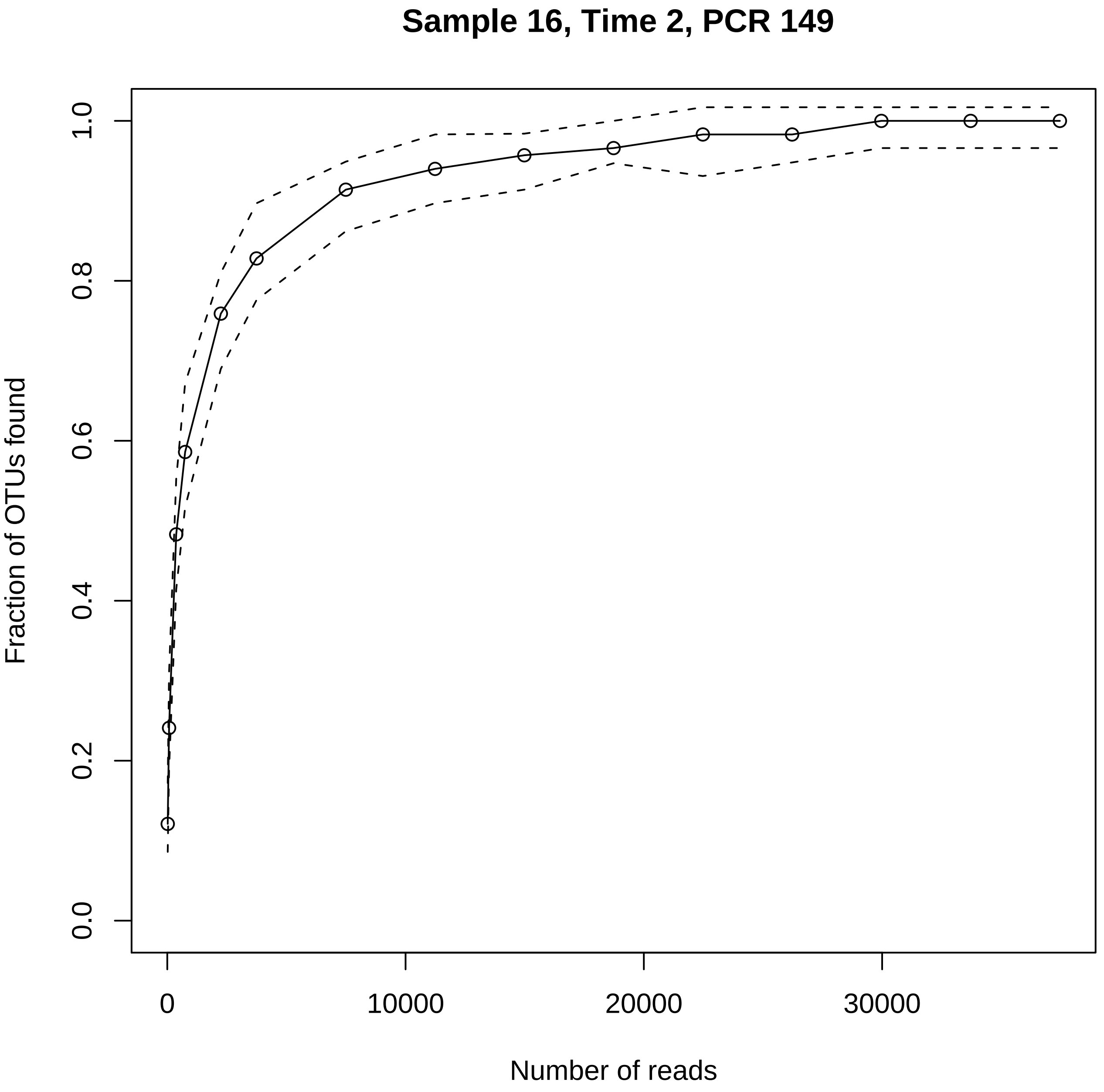
Sample 16, Time 2, PCR 149
Fraction of OTUs found
0.0
0.2
0.4
0.6
0.8
1.0
0
10000
20000
30000
Number of reads

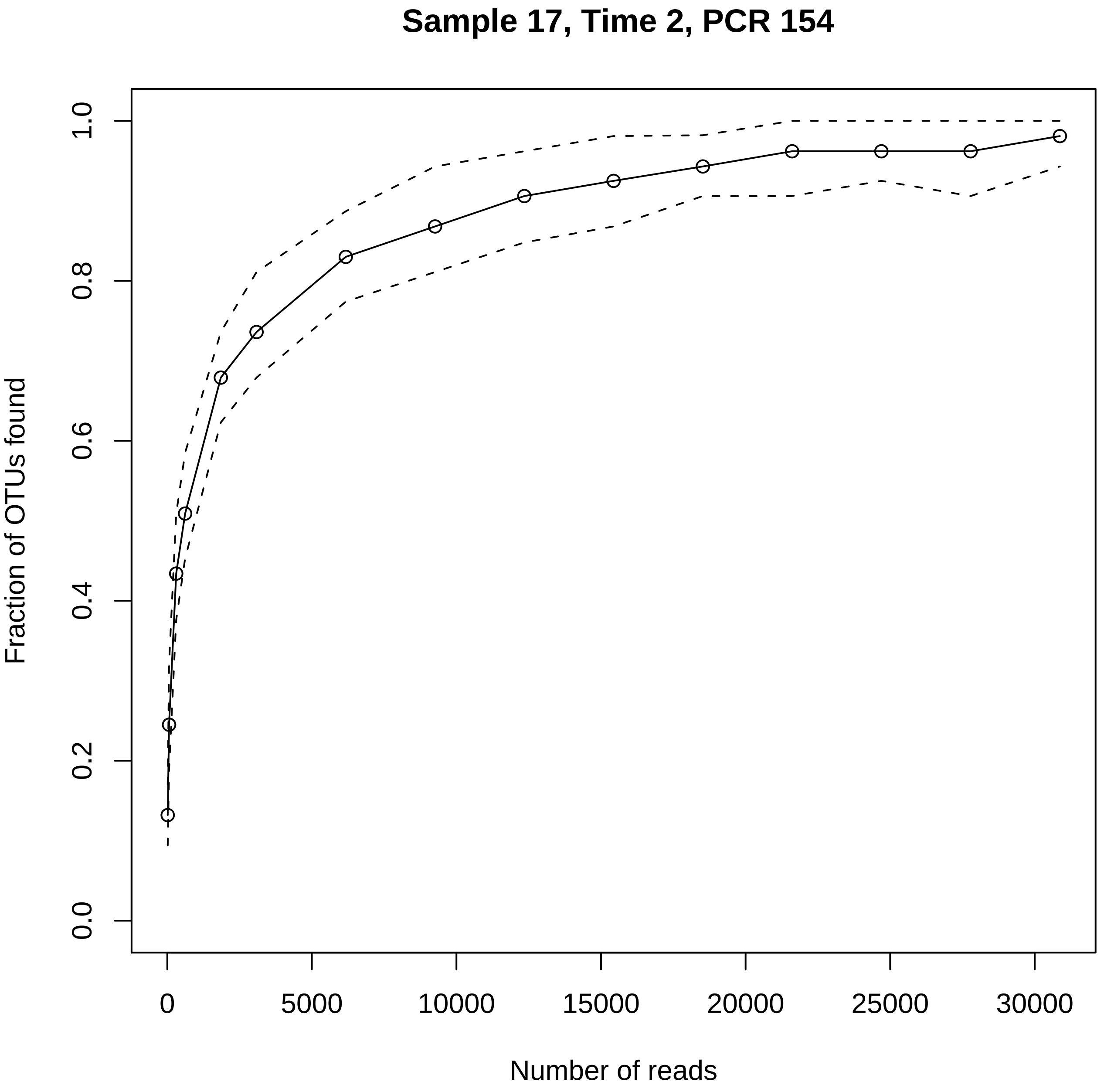

Sample 17, Time 2, PCR 154
Fraction of OTUs found
Number of reads
0.0
0.2
0.4
0.6
0.8
1.0
0
5000
10000
15000
20000
25000
30000

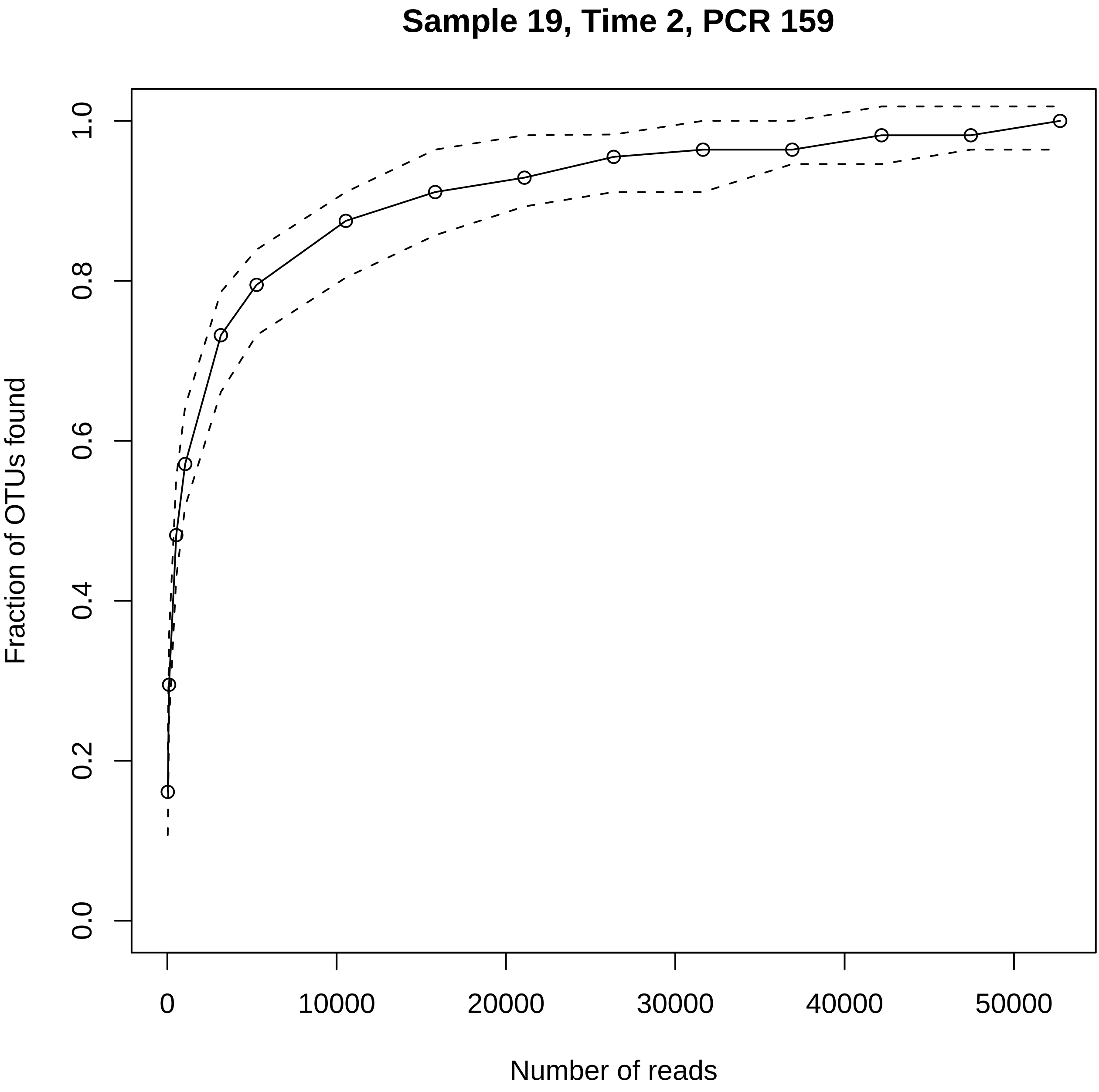
Sample 19, Time 2, PCR 159
Fraction of OTUs found
Number of reads
0.0
0.2
0.4
0.6
0.8
1.0
0
10000
20000
30000
40000
50000

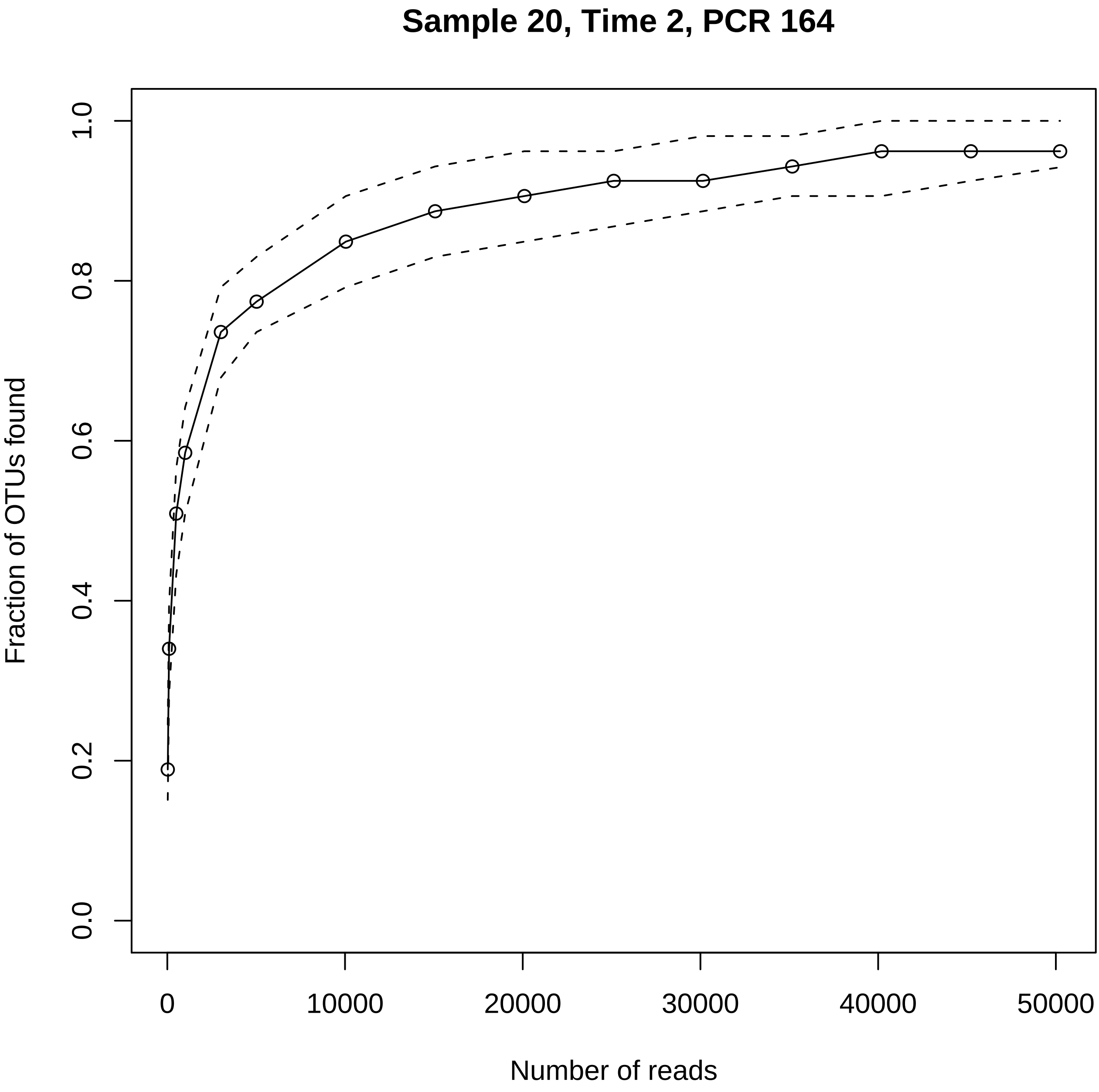
Sample 20, Time 2, PCR 164
Fraction of OTUs found
0.0
0.2
0.4
0.6
0.8
1.0
0
10000
20000
30000
40000
50000
Number of reads

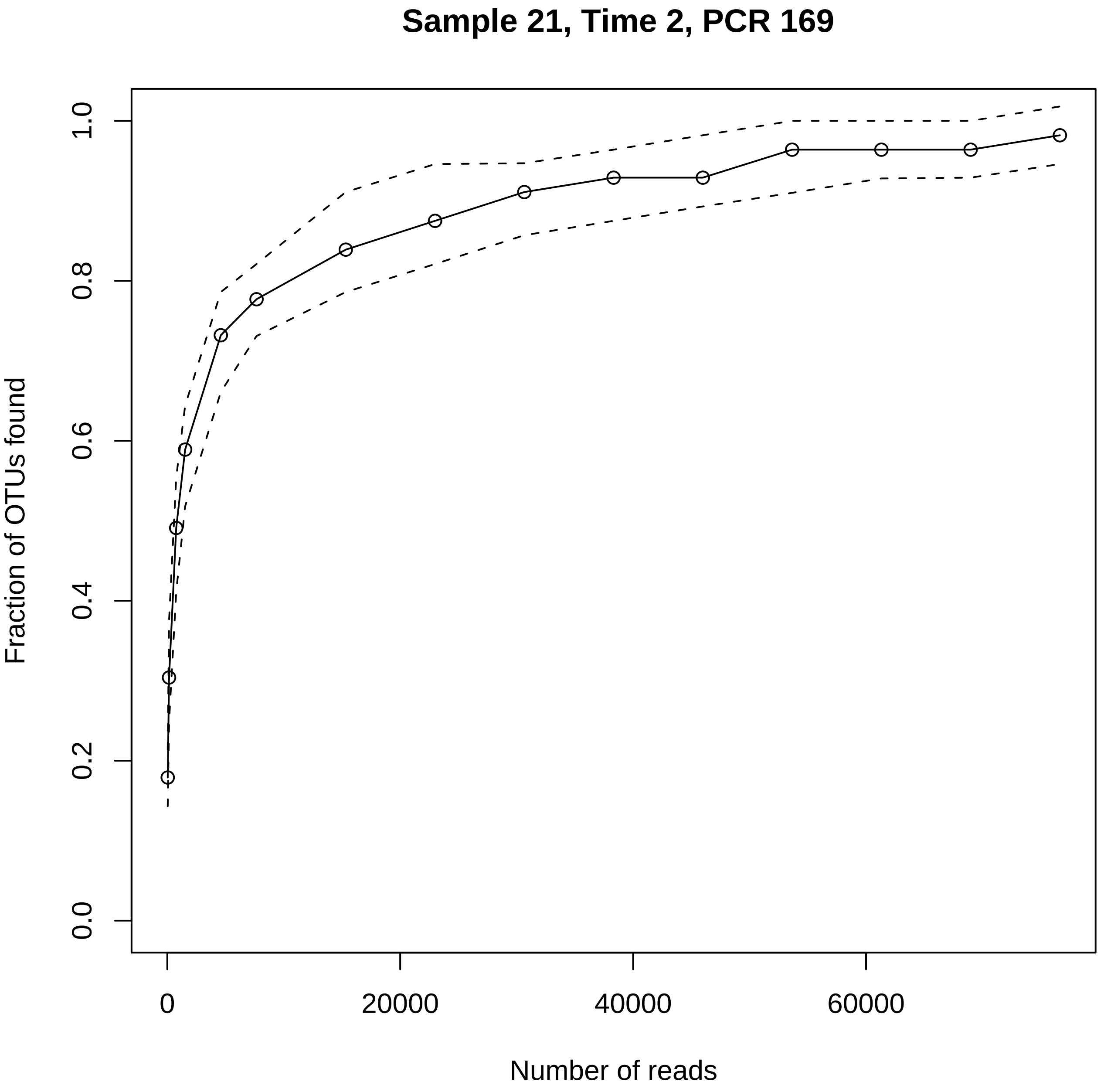
Sample 21, Time 2, PCR 169
Fraction of OTUs found
0.0
0.2
0.4
0.6
0.8
1.0
0
20000
40000
60000
Number of reads

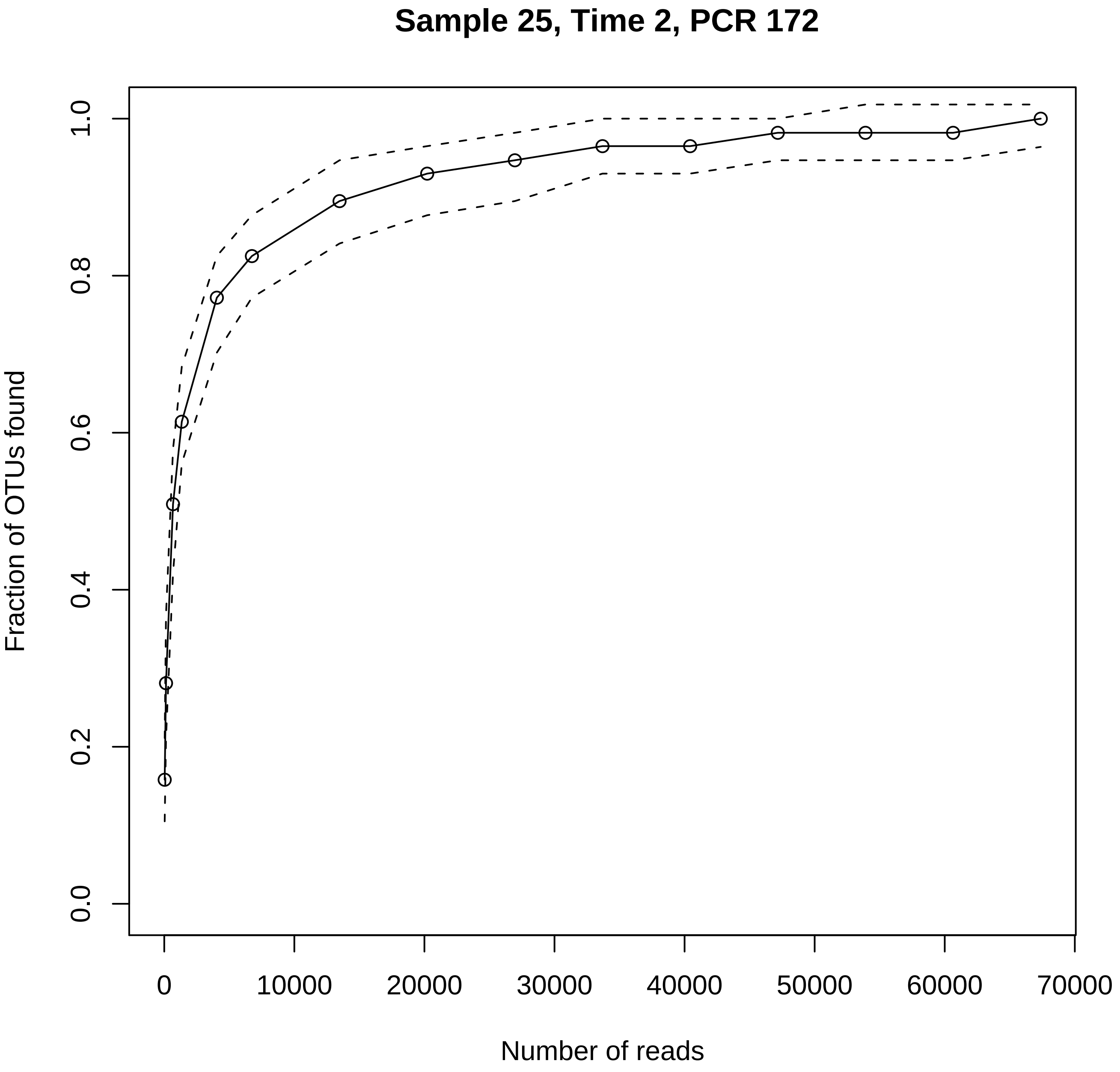
Sample 25, Time 2, PCR 172
Fraction of OTUs found
0.0
0.2
0.4
0.6
0.8
1.0
0
10000
20000
30000
40000
50000
60000
70000
Number of reads

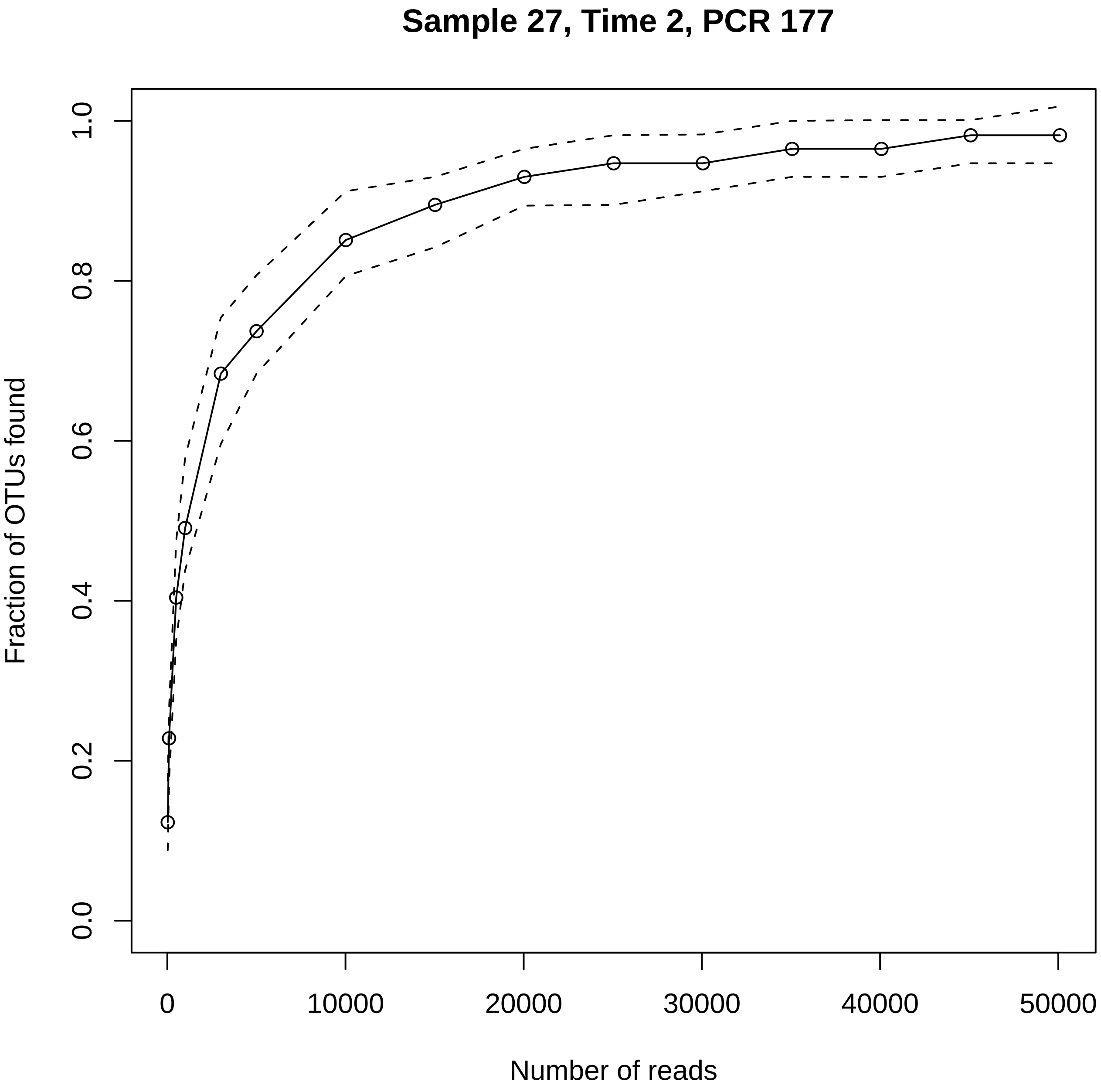

Sample 27, Time 2, PCR 177
Fraction of OTUs found
0.0
0.2
0.4
0.6
0.8
1.0
0
10000
20000
30000
40000
50000
Number of reads

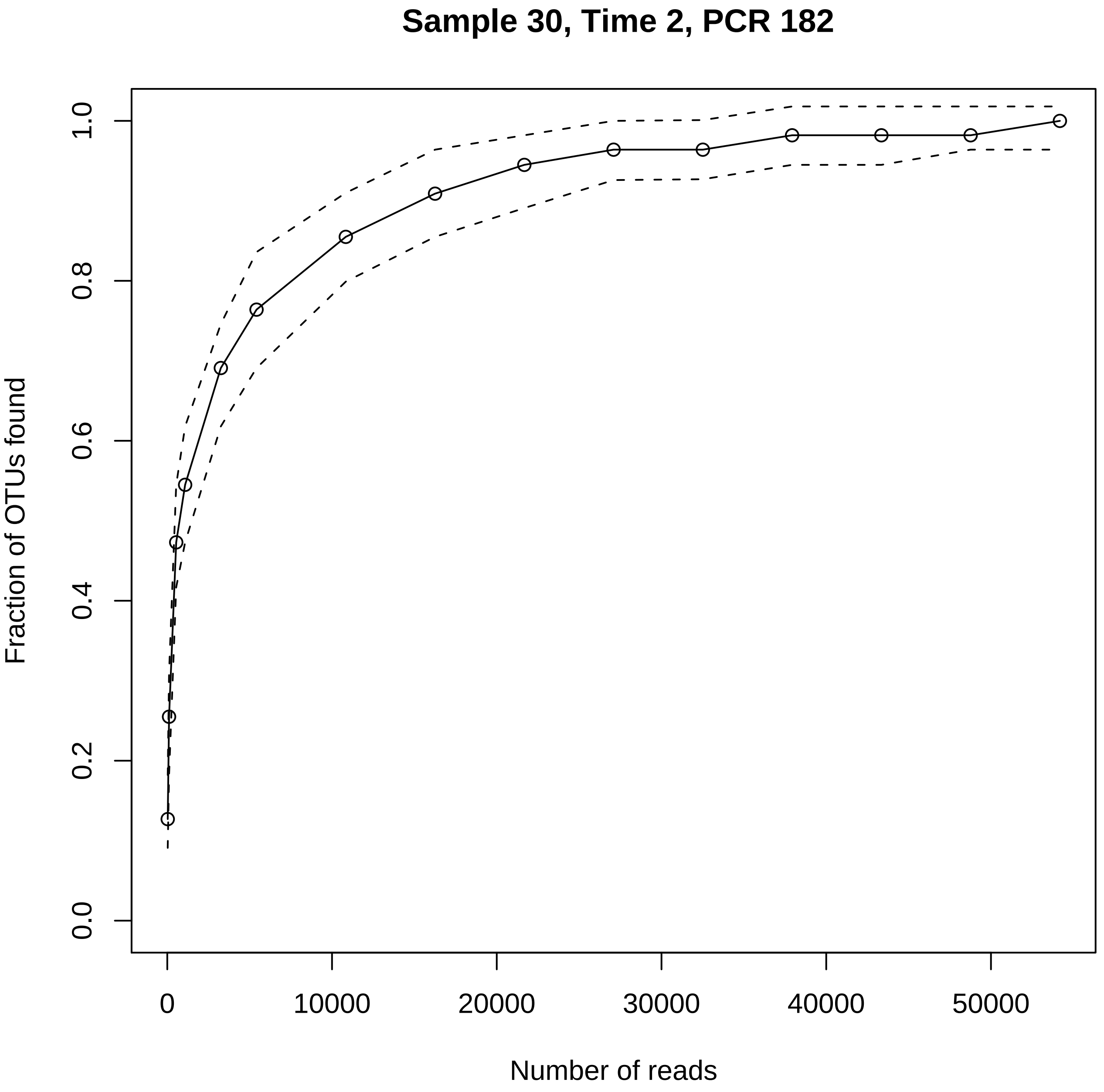

Sample 30, Time 2, PCR 182
Fraction of OTUs found
0.0
0.2
0.4
0.6
0.8
1.0
0
10000
20000
30000
40000
50000
Number of reads

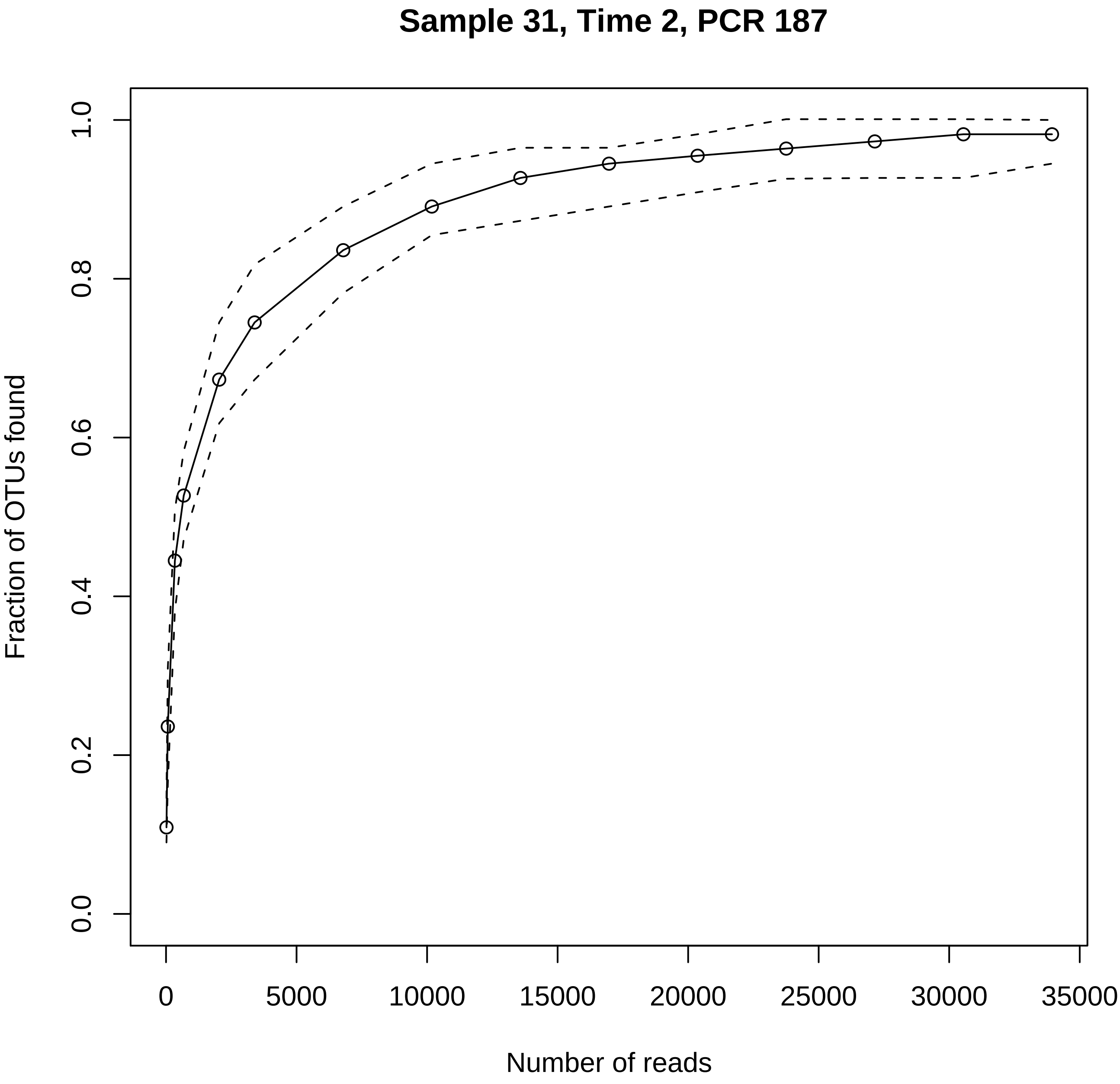
Sample 31, Time 2, PCR 187
Fraction of OTUs found
0.0
0.2
0.4
0.6
0.8
1.0
0
5000
10000
15000
20000
25000
30000
35000
Number of reads

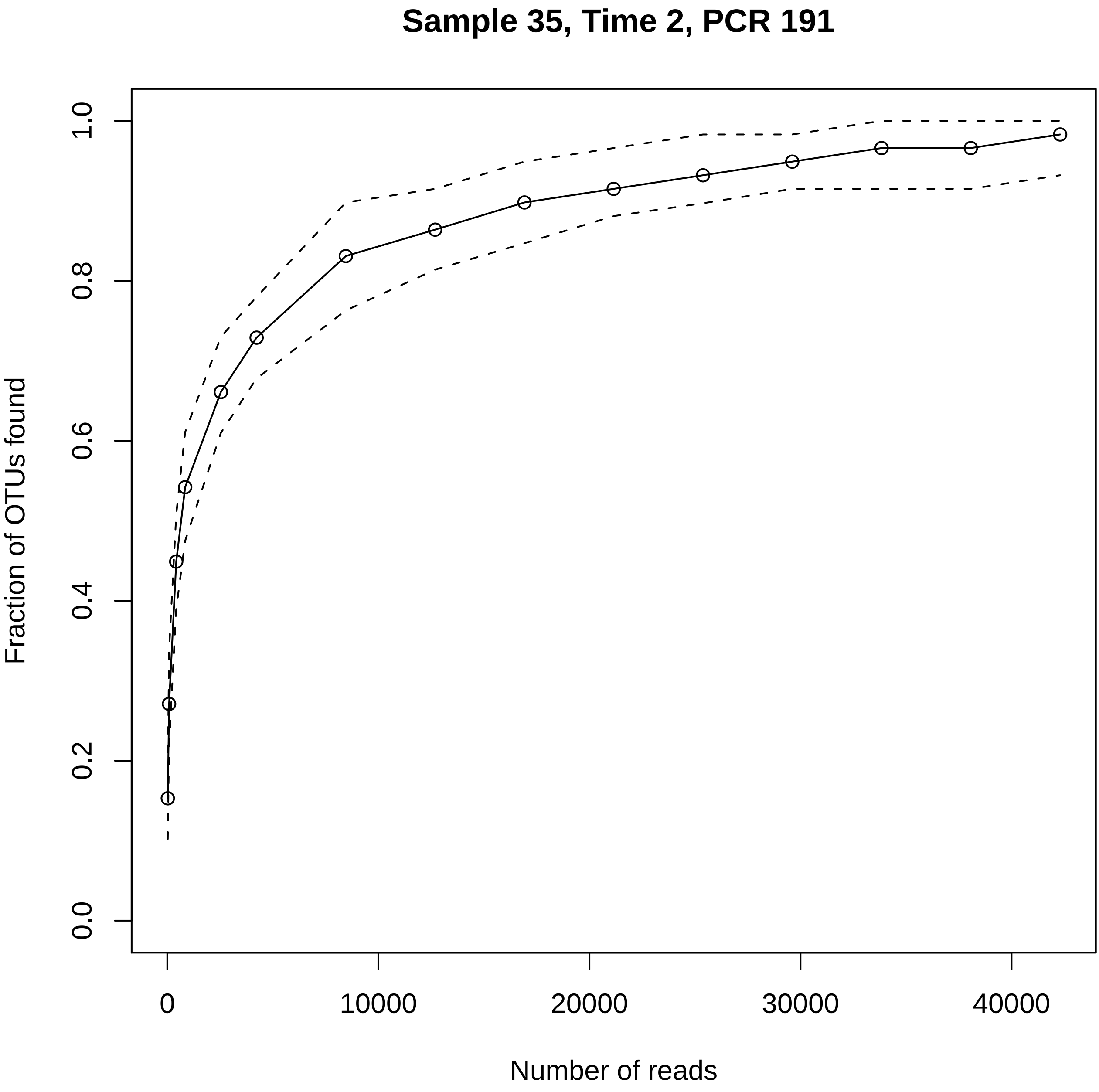

Sample 35, Time 2, PCR 191
Fraction of OTUs found
0.0
0.2
0.4
0.6
0.8
1.0
0
10000
20000
30000
40000
Number of reads

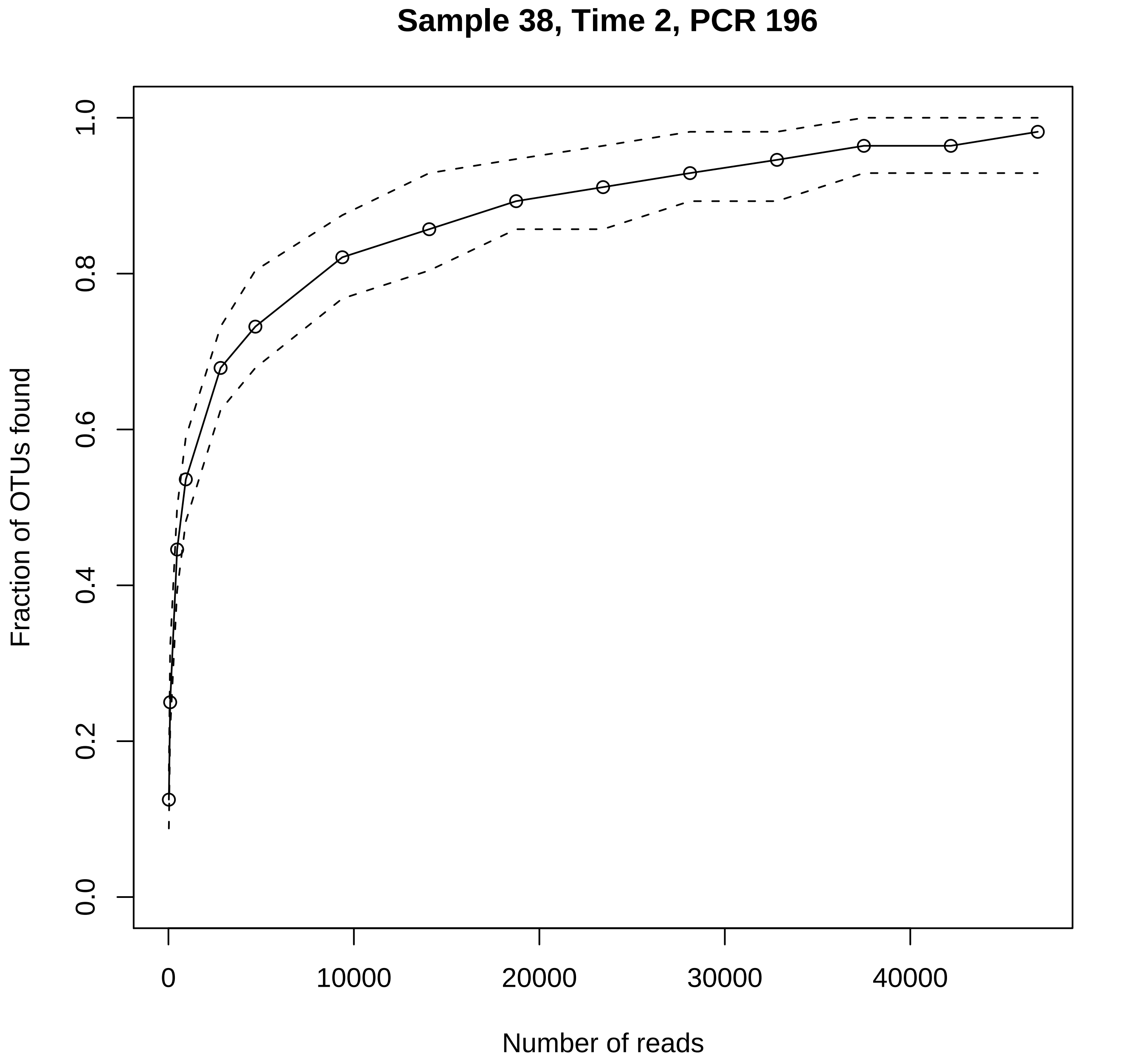
Sample 38, Time 2, PCR 196
Fraction of OTUs found
0.0
0.2
0.4
0.6
0.8
1.0
0
10000
20000
30000
40000
Number of reads

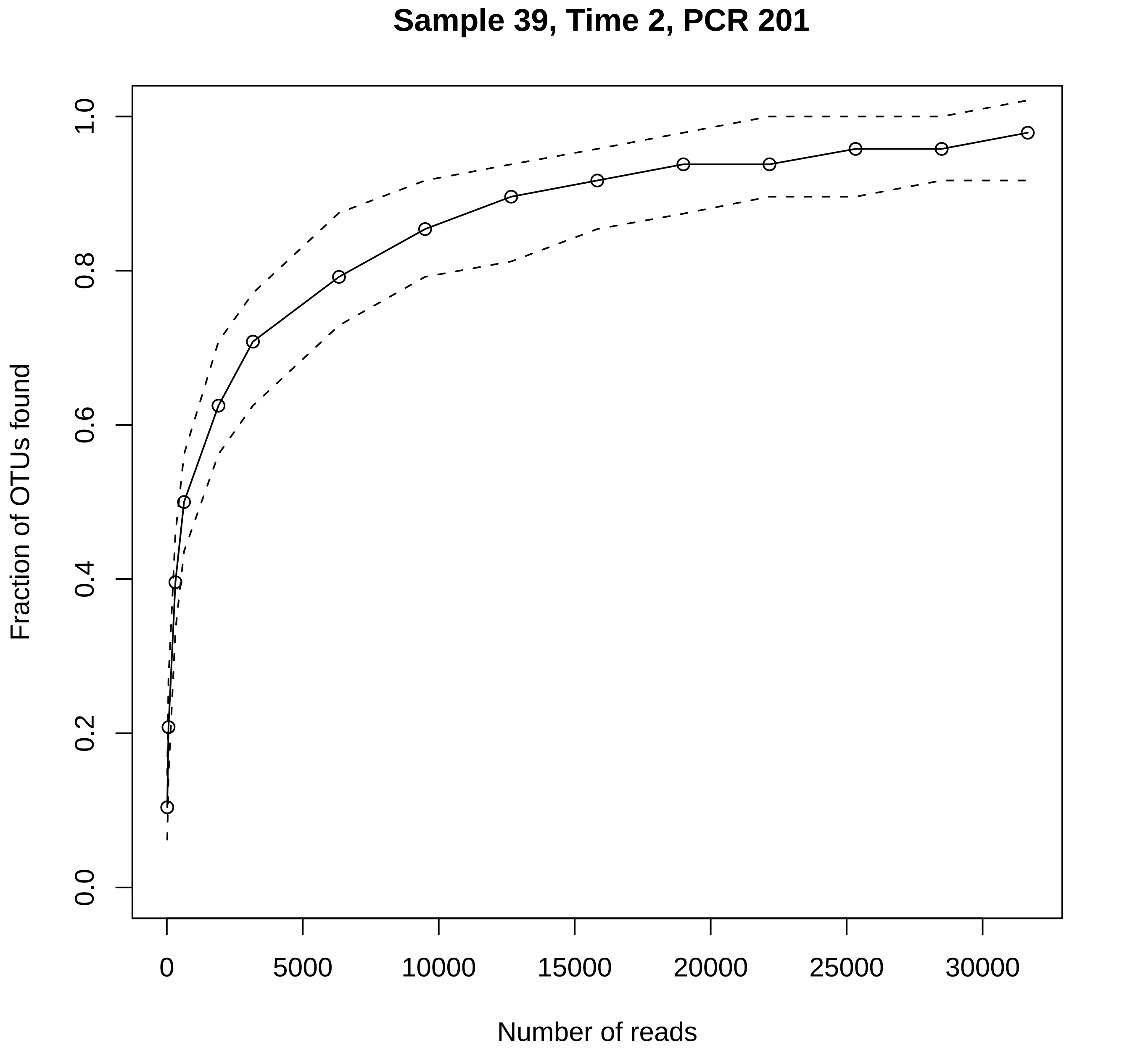
Sample 39, Time 2, PCR 201
Fraction of OTUs found
Number of reads
0.0
0.2
0.4
0.6
0.8
1.0
0
5000
10000
15000
20000
25000
30000

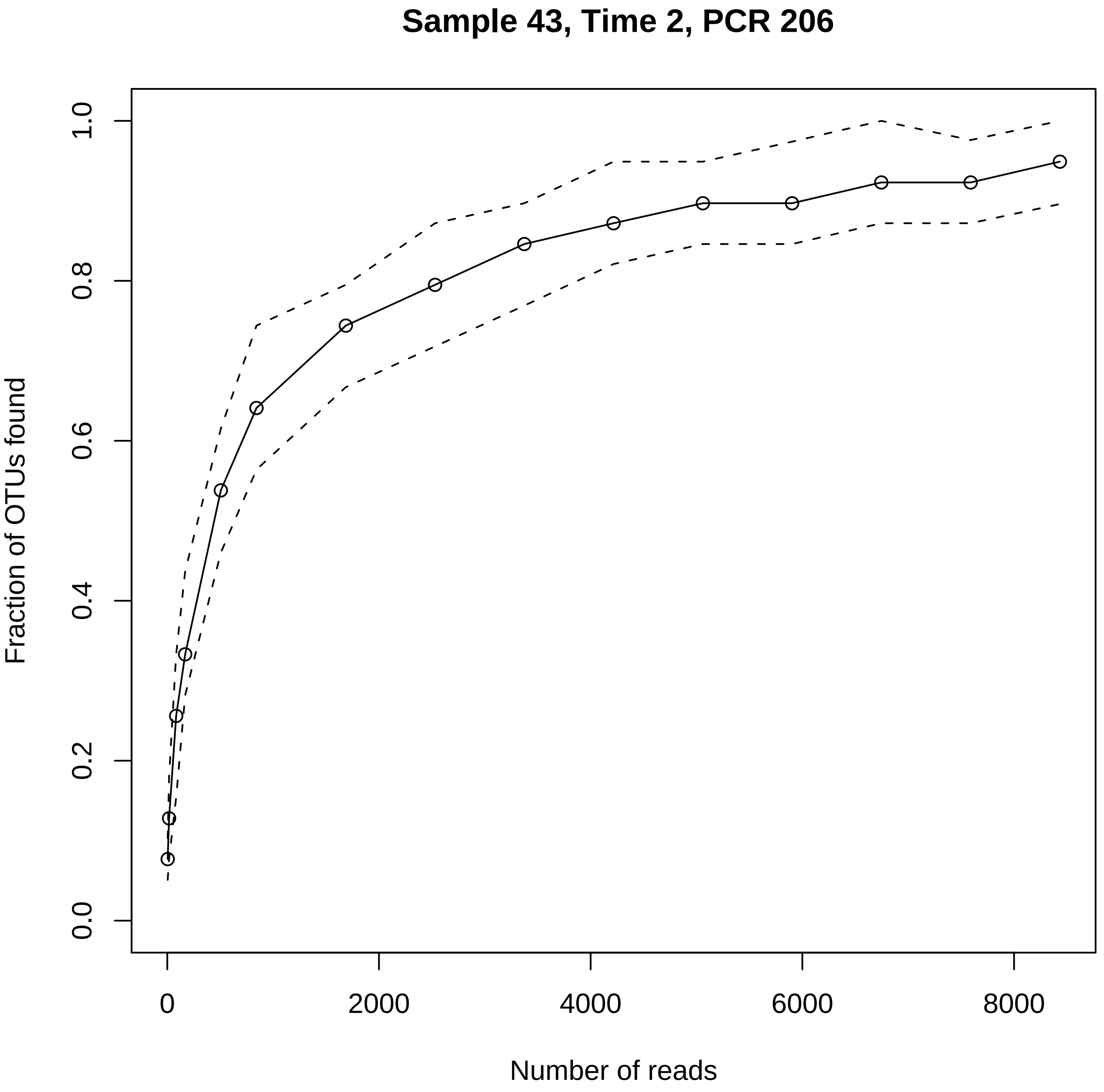

Sample 43, Time 2, PCR 206
Fraction of OTUs found
0.0
0.2
0.4
0.6
0.8
1.0
0
2000
4000
6000
8000
Number of reads

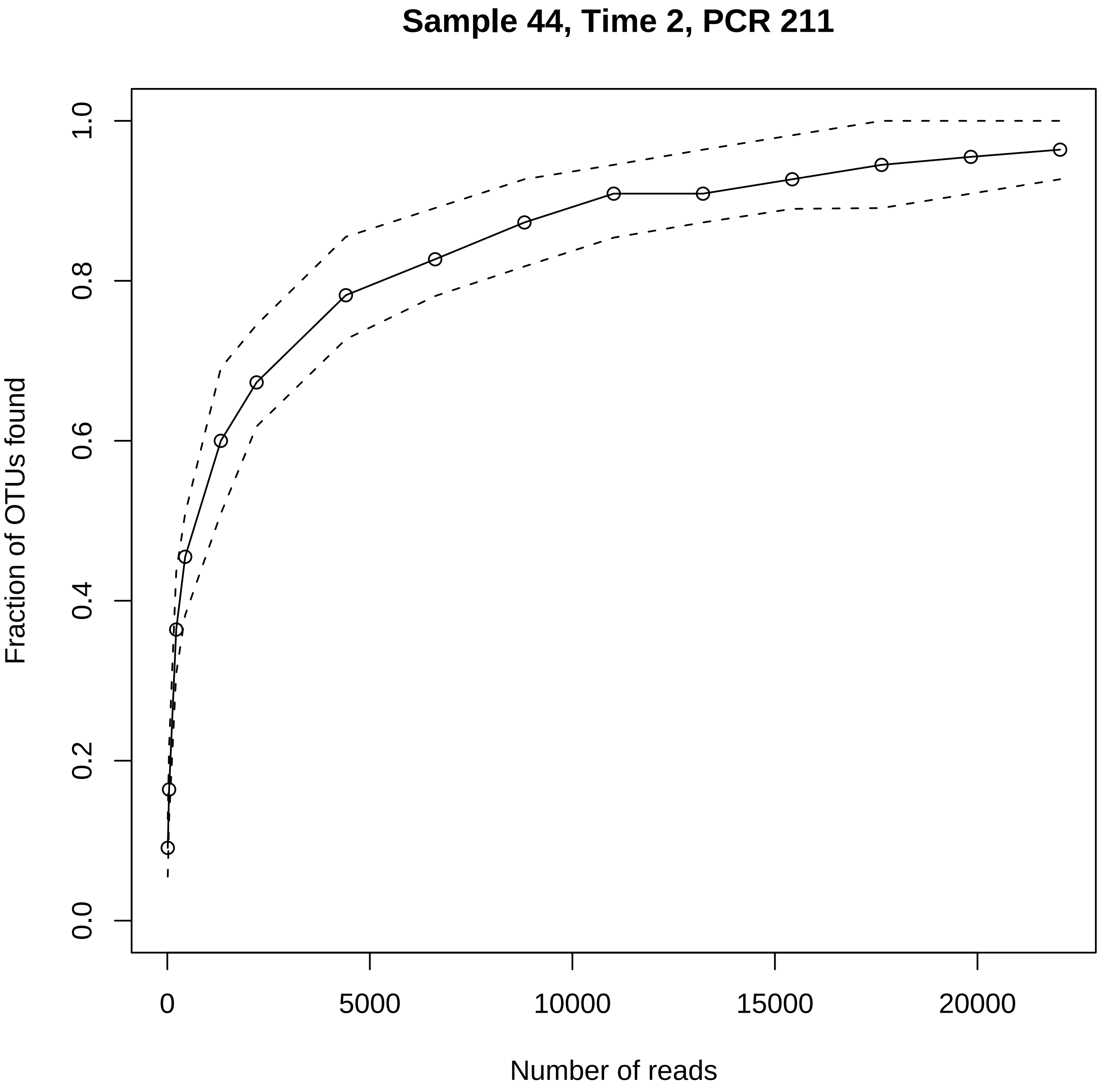
Sample 44, Time 2, PCR 211
Fraction of OTUs found
0.0
0.2
0.4
0.6
0.8
1.0
0
5000
10000
15000
20000
Number of reads

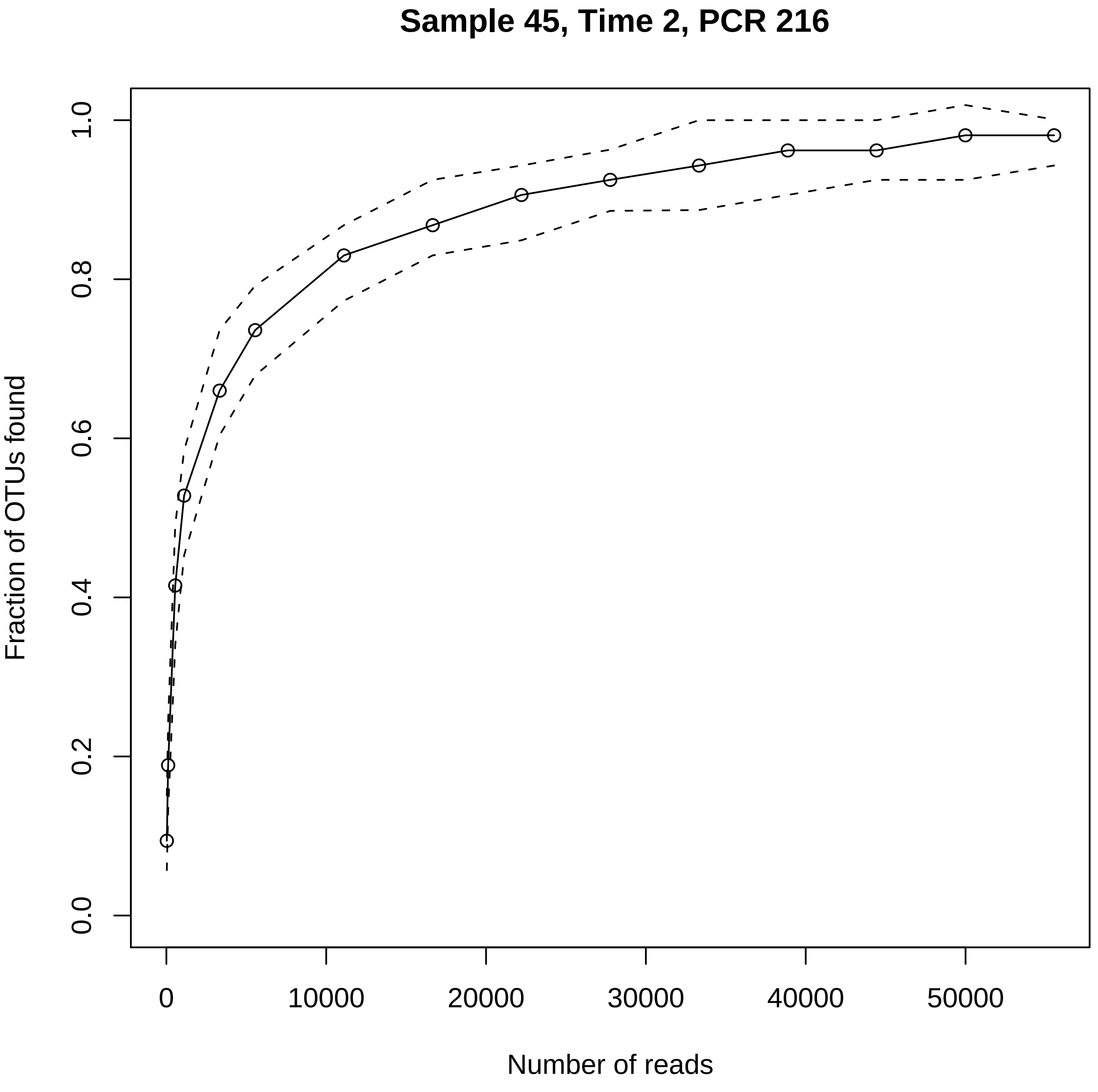
Sample 45, Time 2, PCR 216
Fraction of OTUs found
0.0
0.2
0.4
0.6
0.8
1.0
0
10000
20000
30000
40000
50000
Number of reads

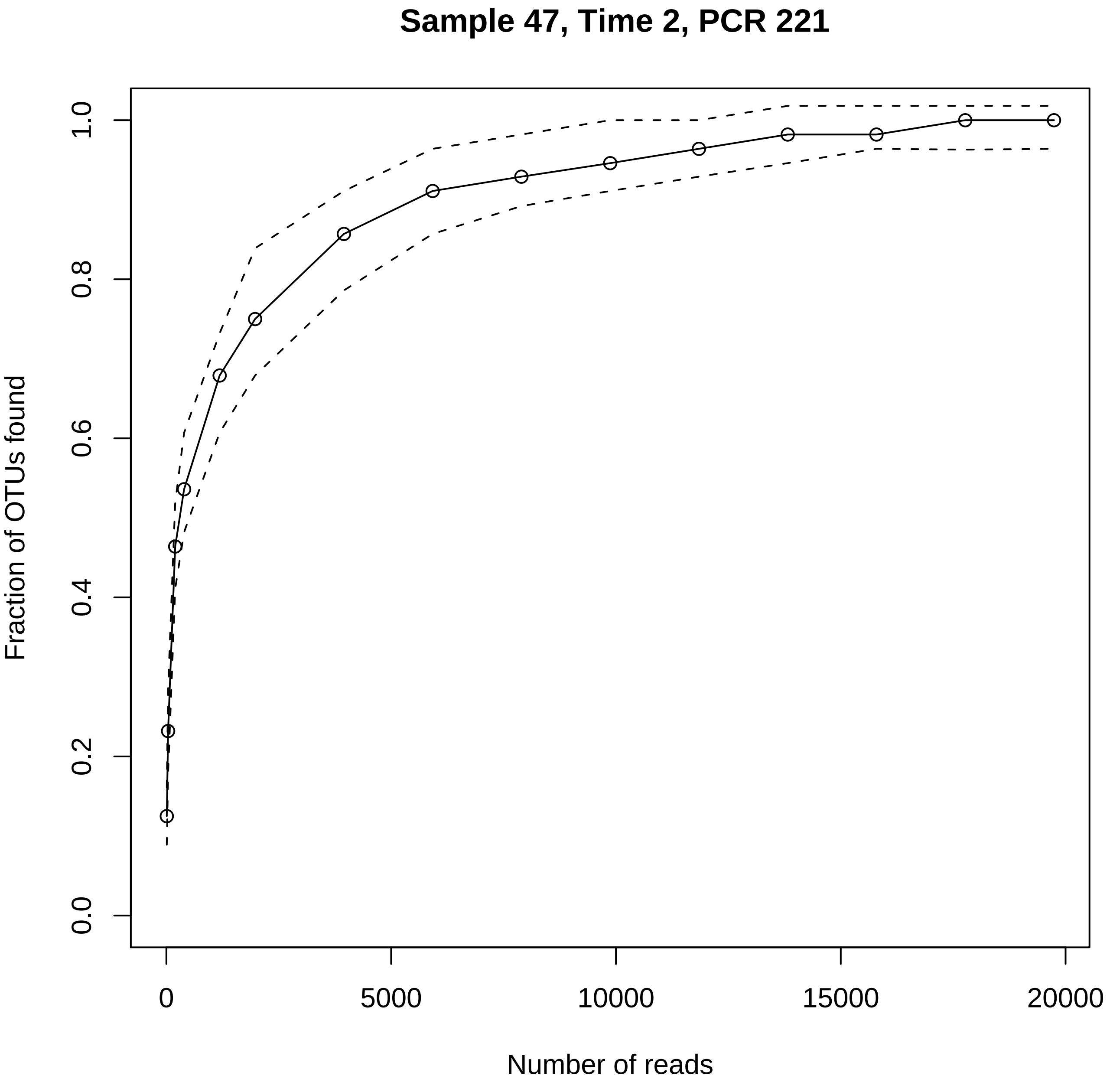

Sample 47, Time 2, PCR 221
Fraction of OTUs found
Number of reads
0.0
0.2
0.4
0.6
0.8
1.0
0
5000
10000
15000
20000

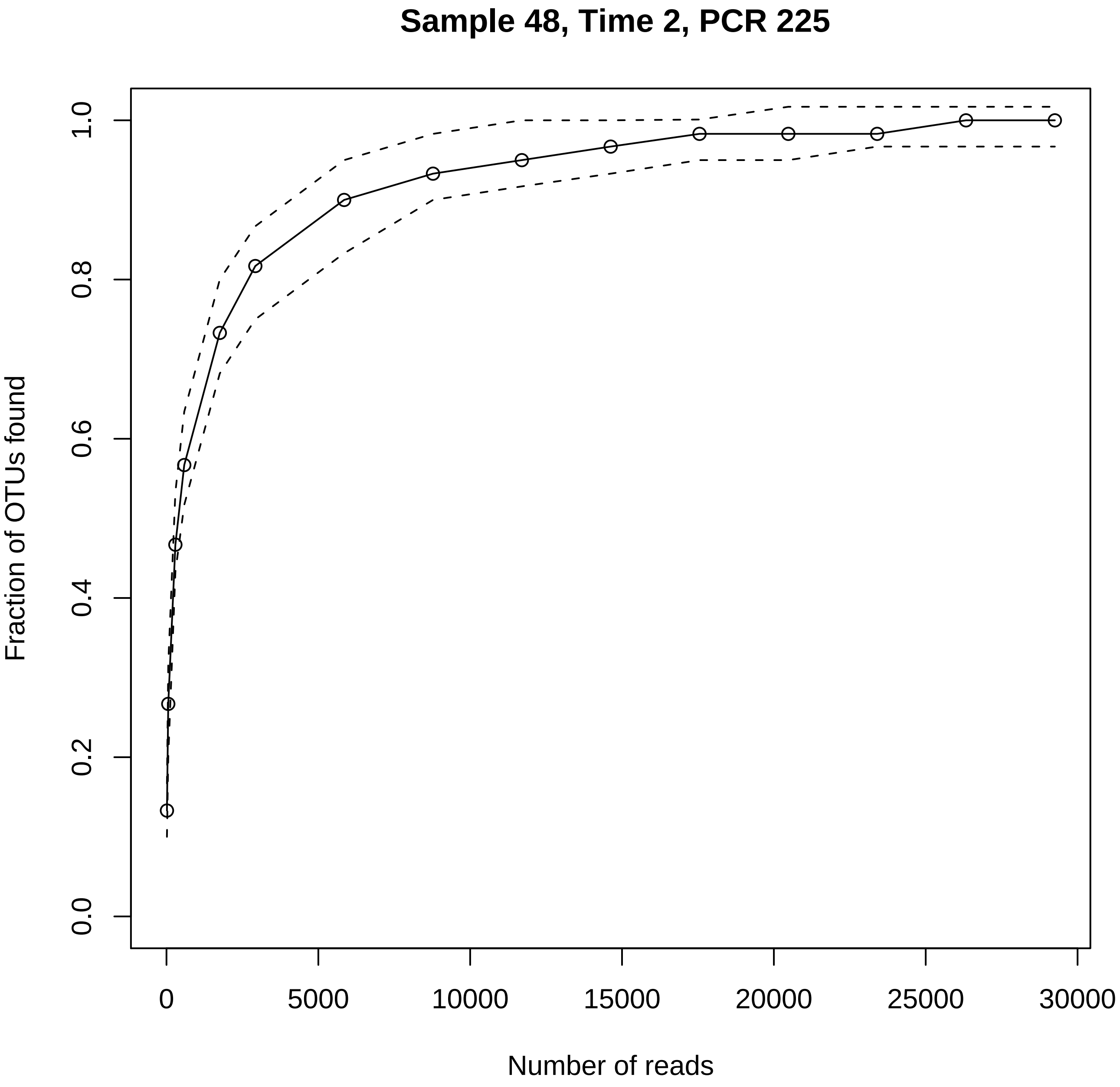

Sample 48, Time 2, PCR 225
Fraction of OTUs found
0.0
0.2
0.4
0.6
0.8
1.0
0
5000
10000
15000
20000
25000
30000
Number of reads

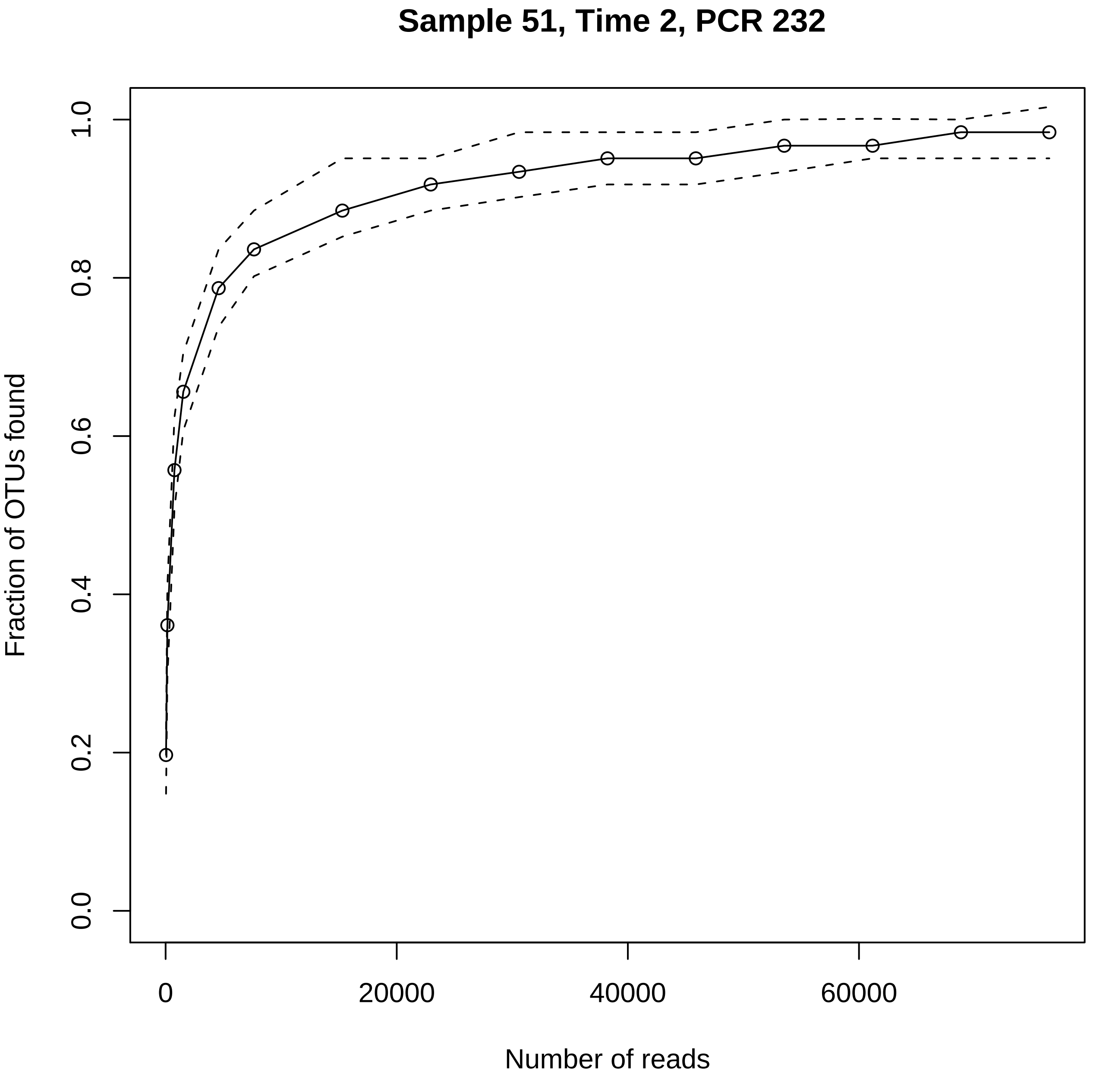

Sample 51, Time 2, PCR 232
Fraction of OTUs found
Number of reads
0.0
0.2
0.4
0.6
0.8
1.0
0
20000
40000
60000

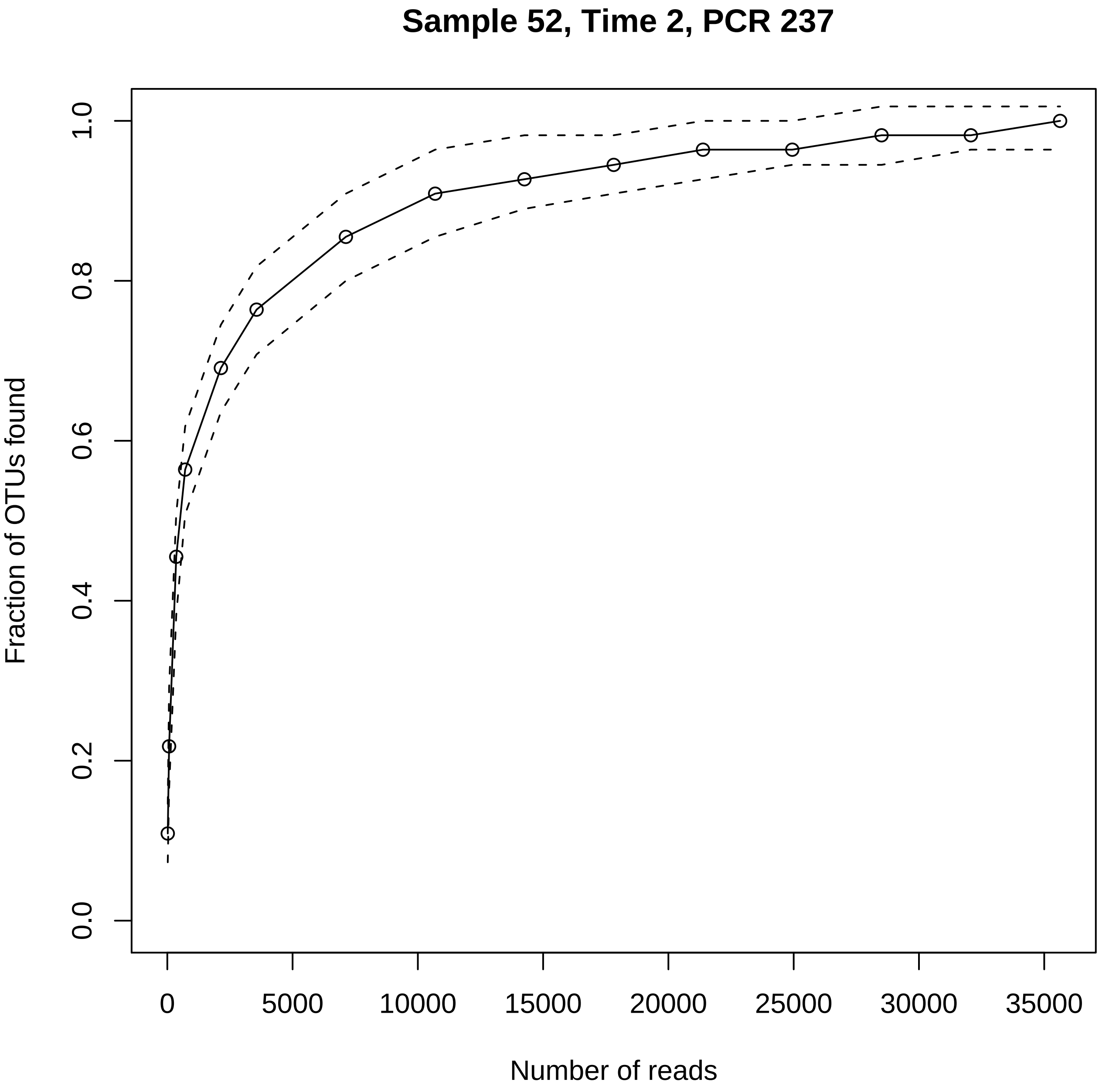

Sample 52, Time 2, PCR 237
Fraction of OTUs found
Number of reads
0.0
0.2
0.4
0.6
0.8
1.0
0
5000
10000
15000
20000
25000
30000
35000

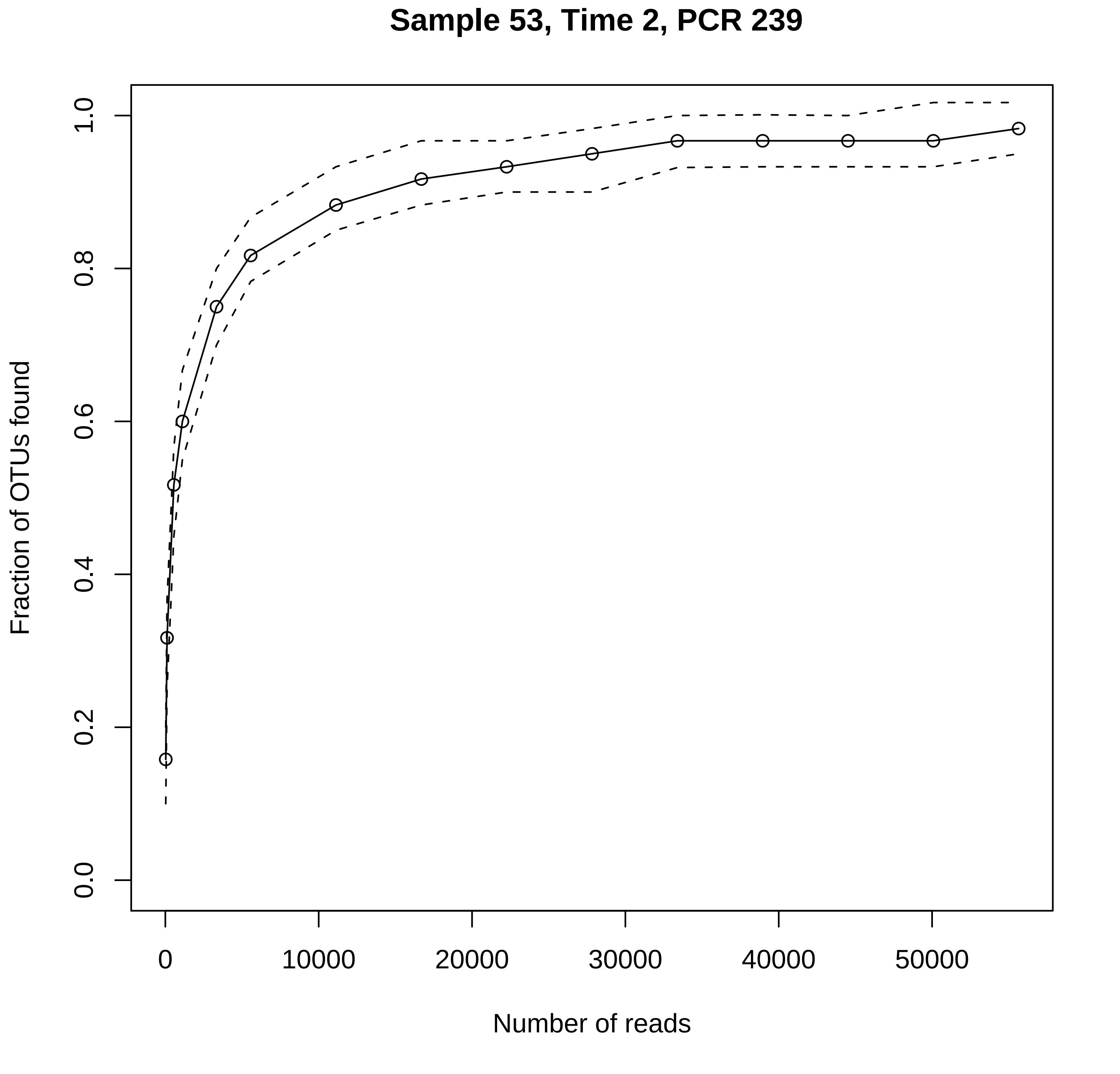

Sample 53, Time 2, PCR 239
Fraction of OTUs found
0.0
0.2
0.4
0.6
0.8
1.0
0
10000
20000
30000
40000
50000
Number of reads

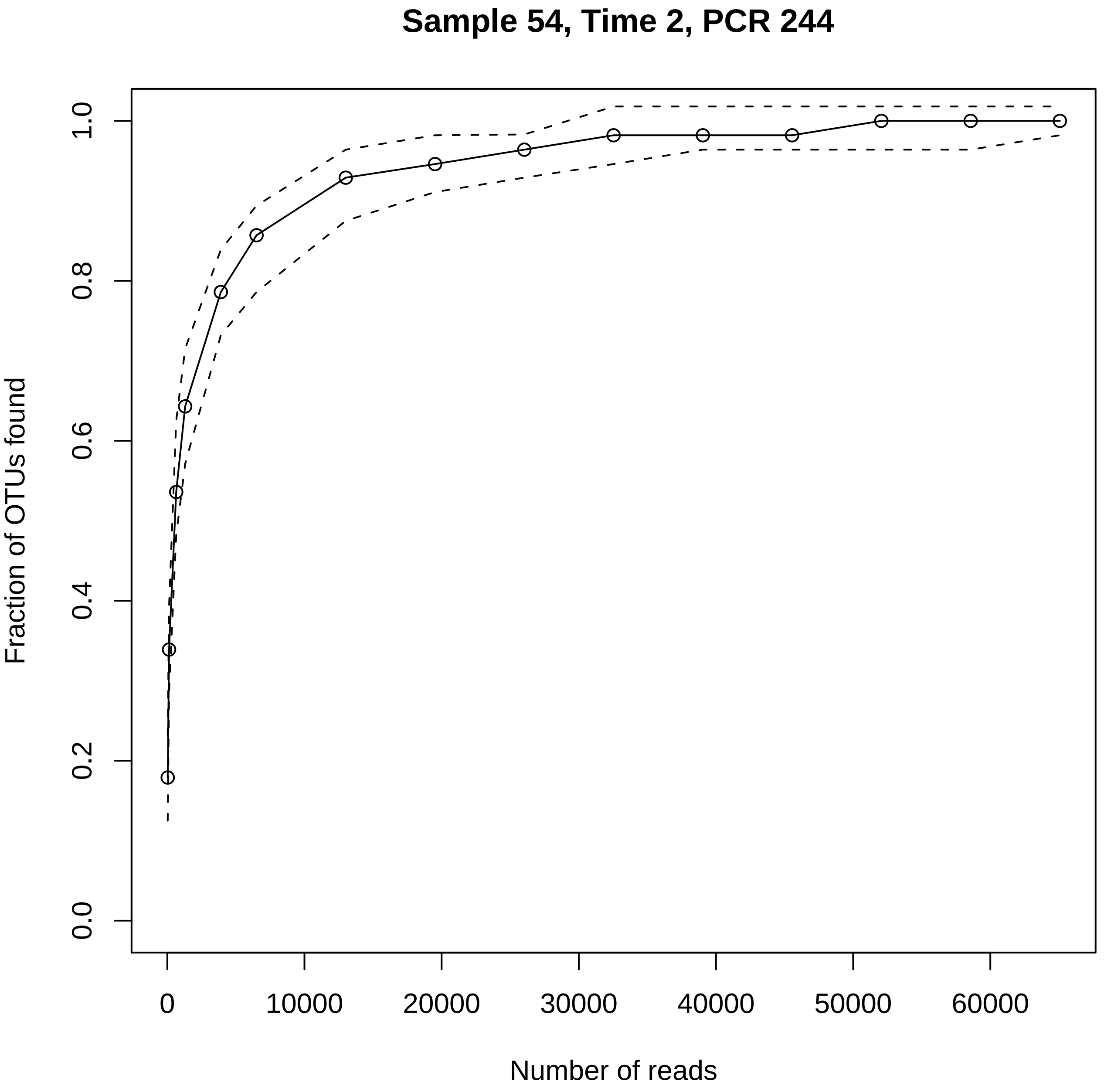

Sample 54, Time 2, PCR 244
1.0
0.8
0.6
0.4
0.2
0.0
Fraction of OTUs found
0
10000
20000
30000
40000
50000
60000
Number of reads

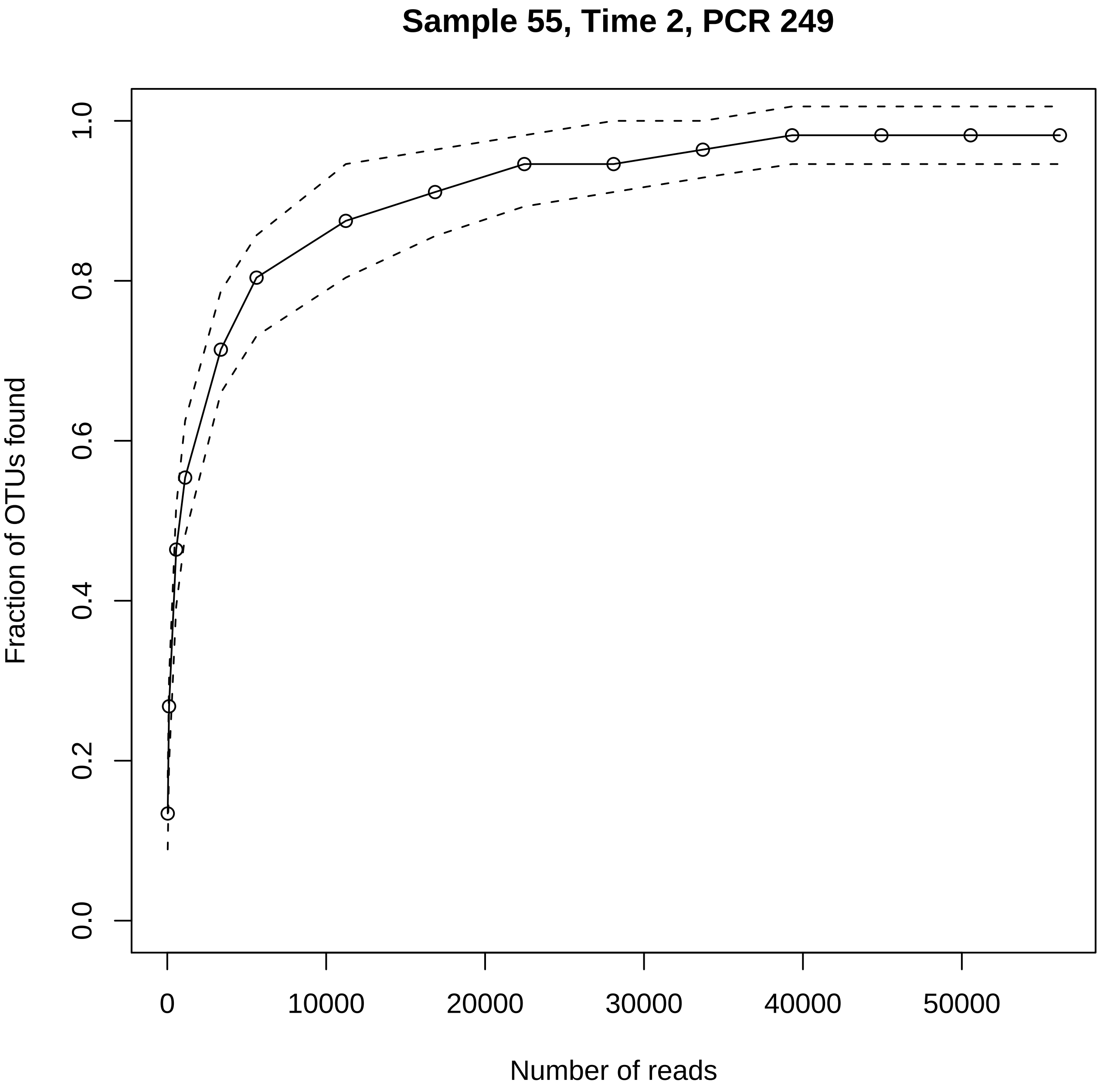
Sample 55, Time 2, PCR 249
Fraction of OTUs found
0.0
0.2
0.4
0.6
0.8
1.0
0
10000
20000
30000
40000
50000
Number of reads

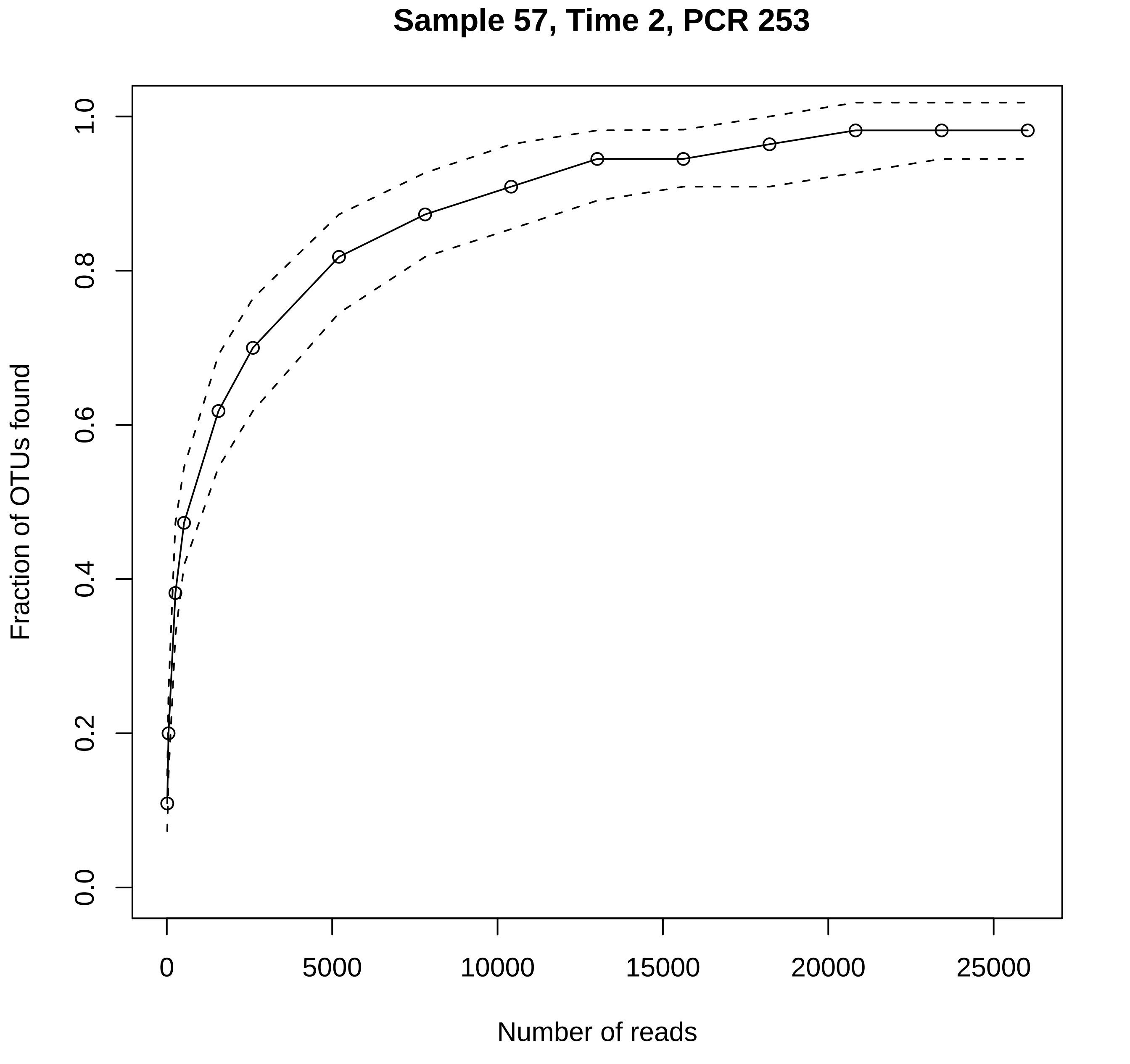

Sample 57, Time 2, PCR 253
Fraction of OTUs found
0.0
0.2
0.4
0.6
0.8
1.0
0
5000
10000
15000
20000
25000
Number of reads

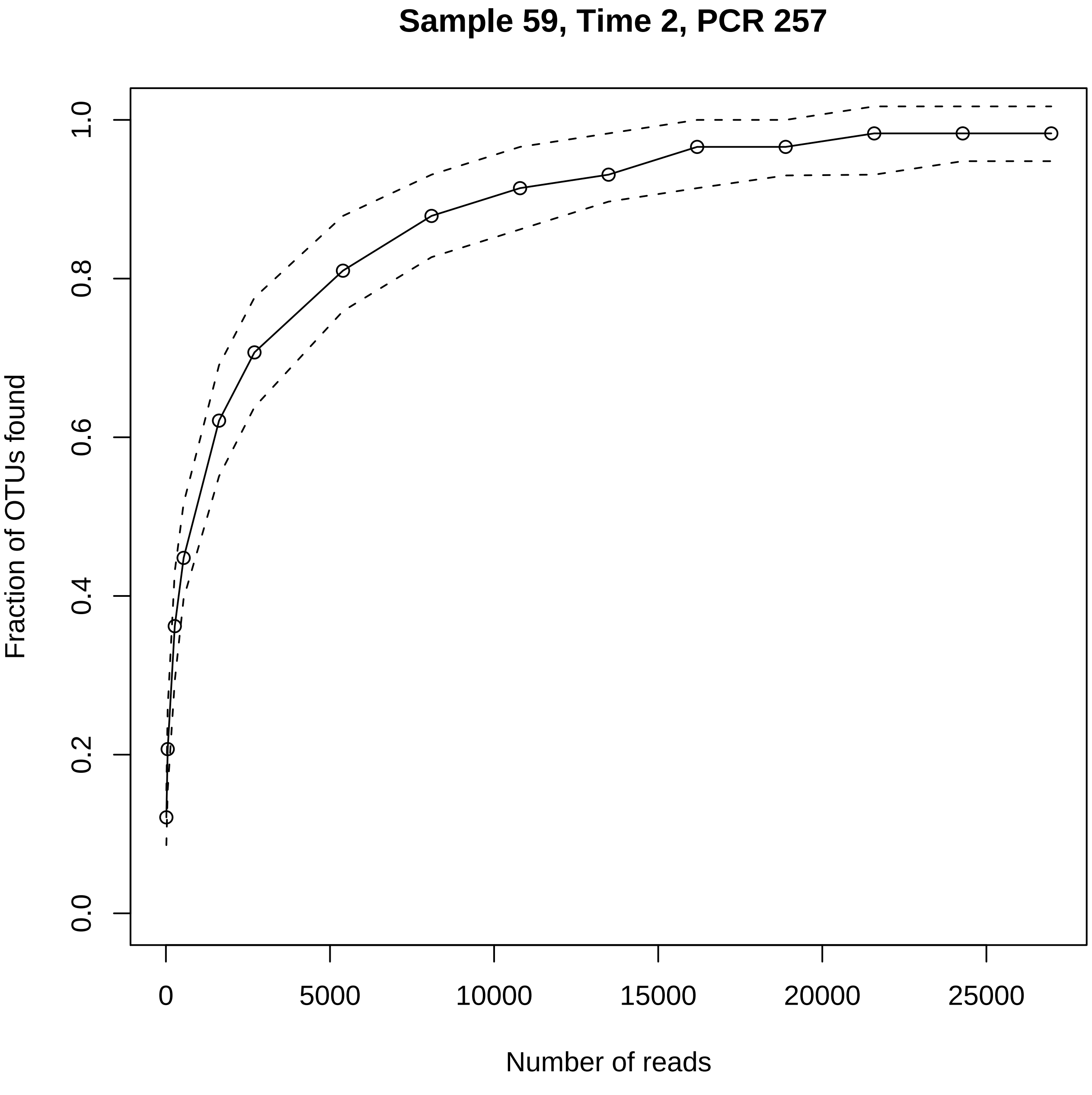
Sample 59, Time 2, PCR 257
Fraction of OTUs found
0.0
0.2
0.4
0.6
0.8
1.0
0
5000
10000
15000
20000
25000
Number of reads

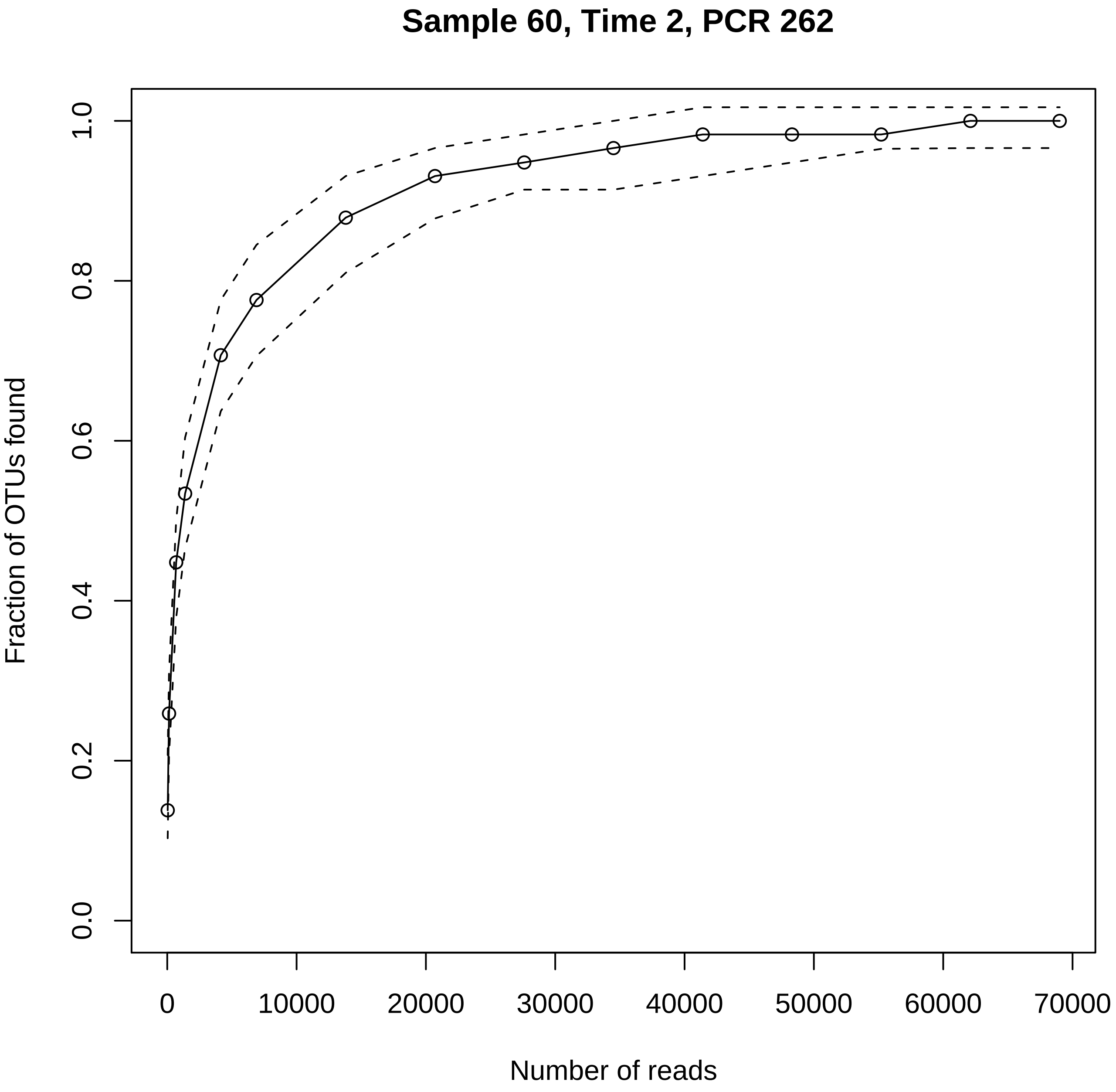

Sample 60, Time 2, PCR 262
Fraction of OTUs found
0.0
0.2
0.4
0.6
0.8
1.0
0
10000
20000
30000
40000
50000
60000
70000
Number of reads

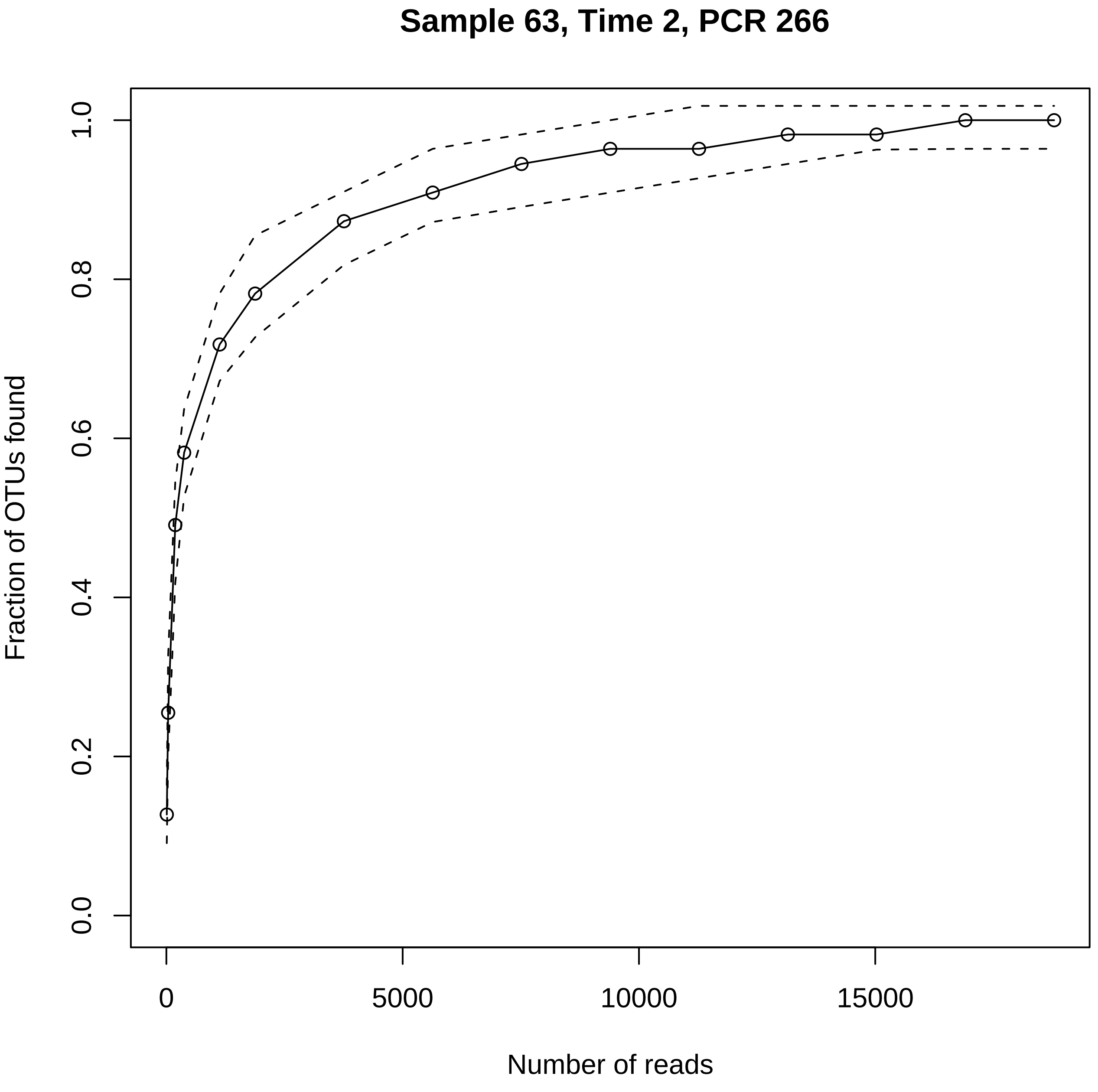
Sample 63, Time 2, PCR 266
Fraction of OTUs found
Number of reads
0.0
0.2
0.4
0.6
0.8
1.0
0
5000
10000
15000

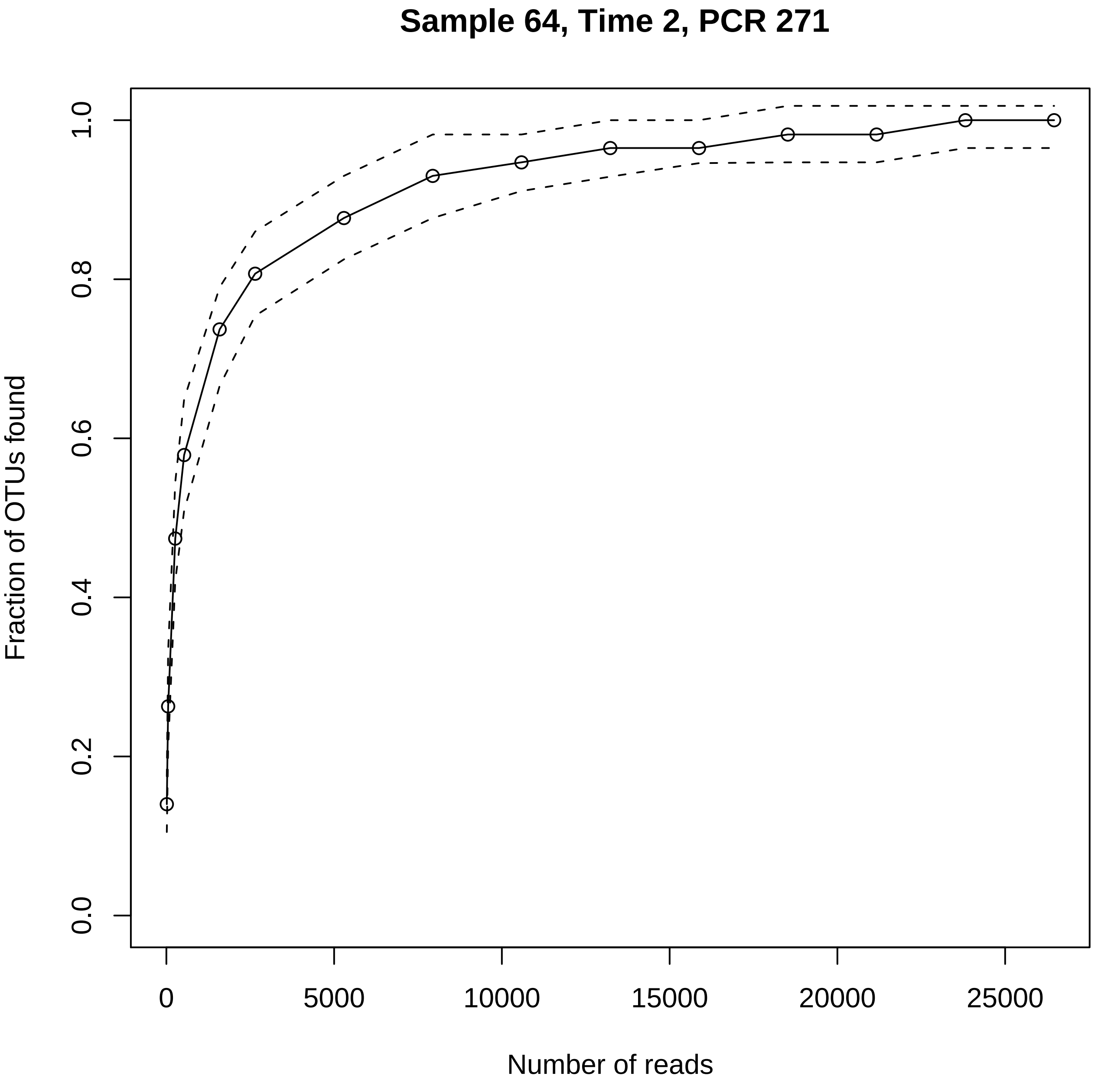
Sample 64, Time 2, PCR 271
Fraction of OTUs found
0.0
0.2
0.4
0.6
0.8
1.0
0
5000
10000
15000
20000
25000
Number of reads

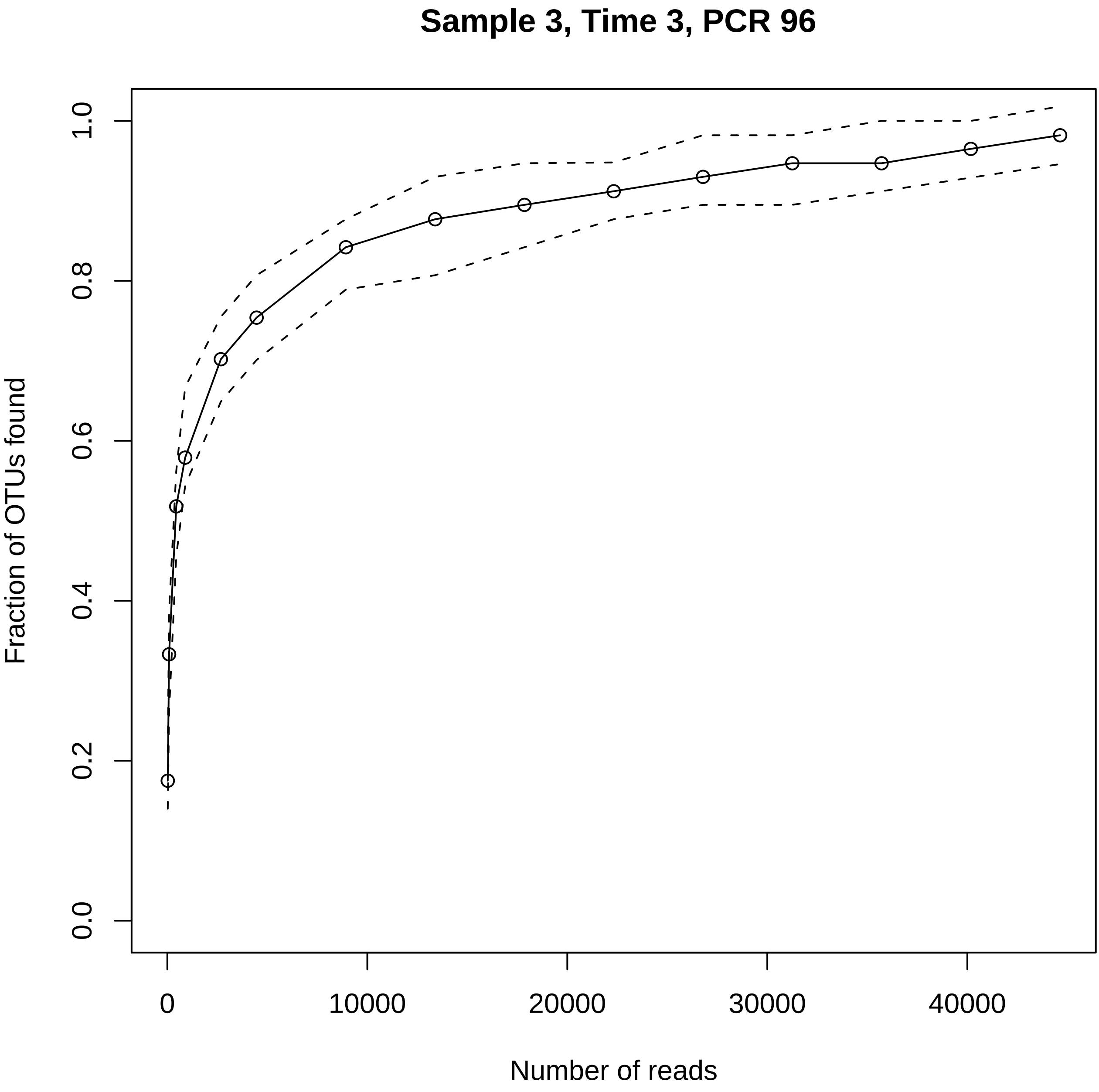

Sample 3, Time 3, PCR 96
Fraction of OTUs found
0.0
0.2
0.4
0.6
0.8
1.0
0
10000
20000
30000
40000
Number of reads

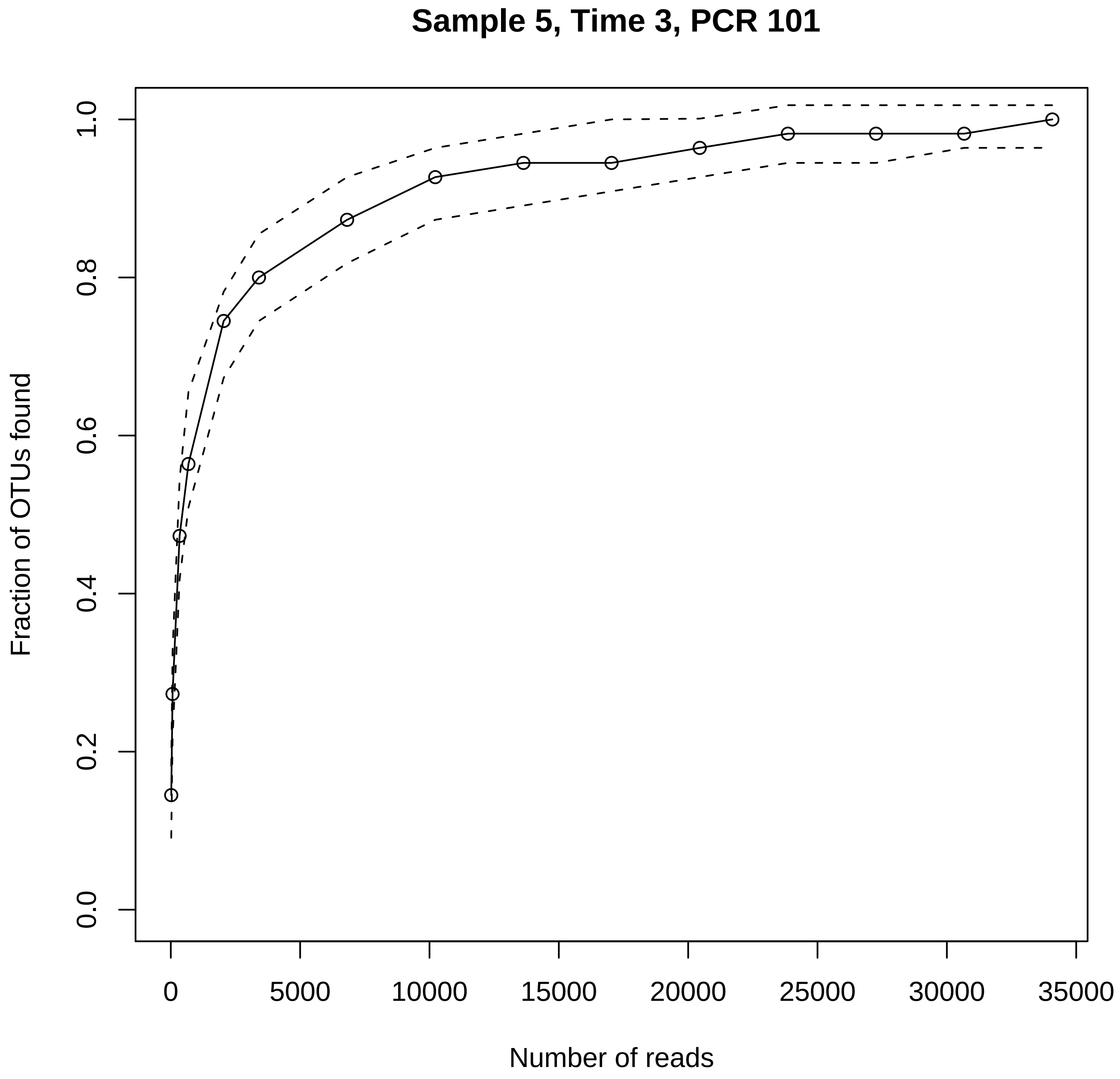

Sample 5, Time 3, PCR 101
Fraction of OTUs found
0.0
0.2
0.4
0.6
0.8
1.0
0
5000
10000
15000
20000
25000
30000
35000
Number of reads

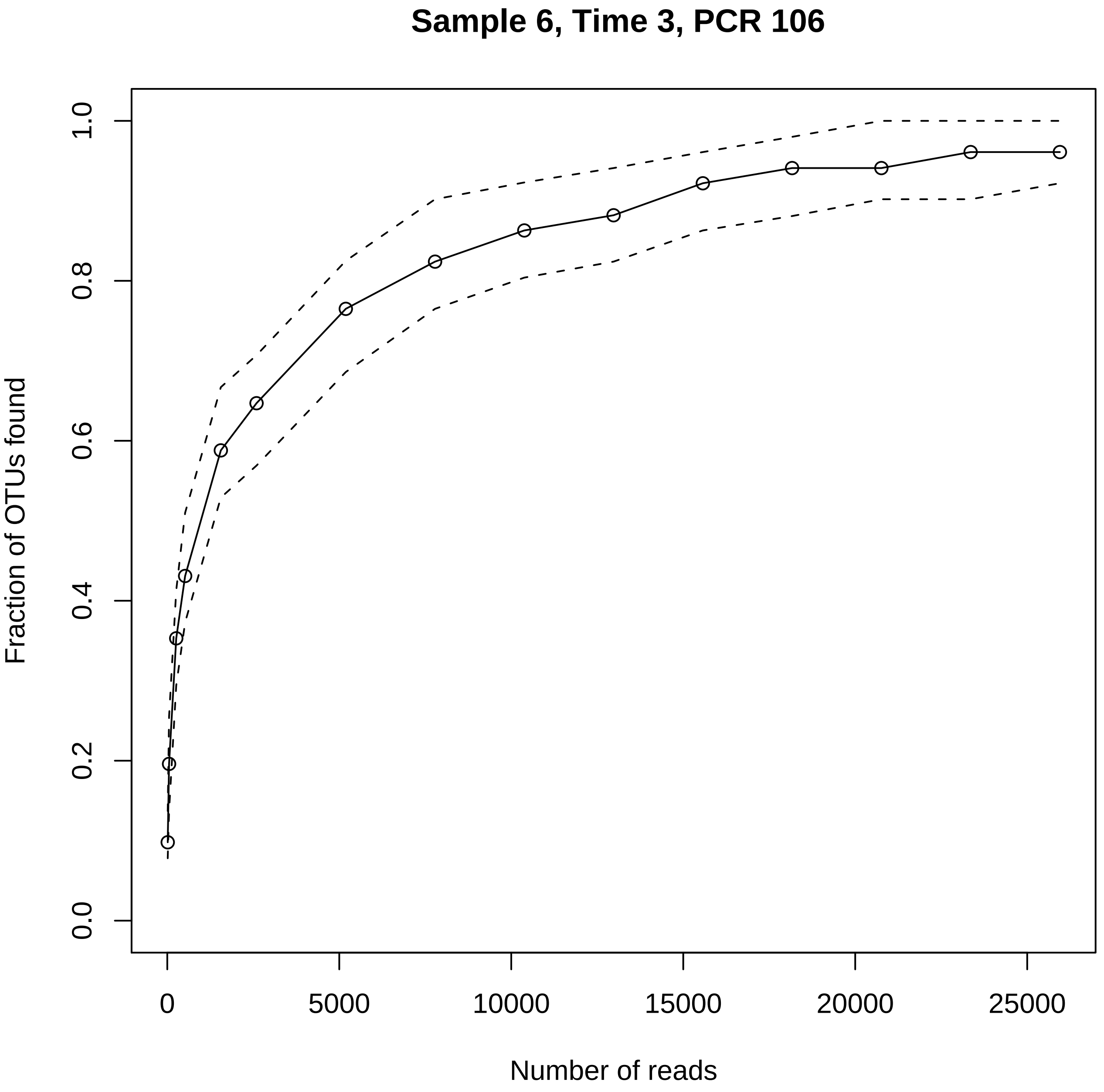
Sample 6, Time 3, PCR 106
Fraction of OTUs found
0.0
0.2
0.4
0.6
0.8
1.0
0
5000
10000
15000
20000
25000
Number of reads

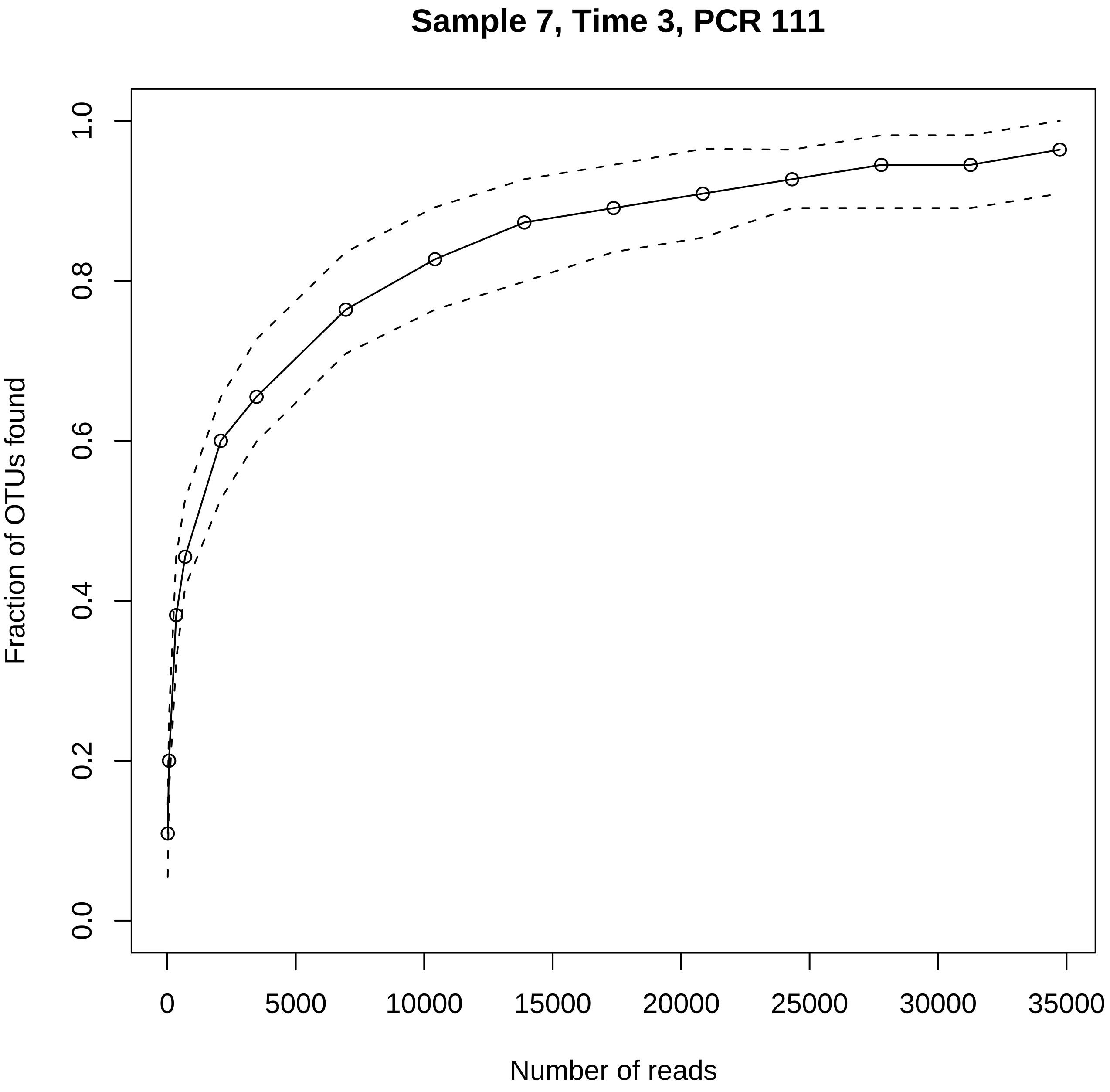
Sample 7, Time 3, PCR 111
Fraction of OTUs found
0.0
0.2
0.4
0.6
0.8
1.0
0
5000
10000
15000
20000
25000
30000
35000
Number of reads

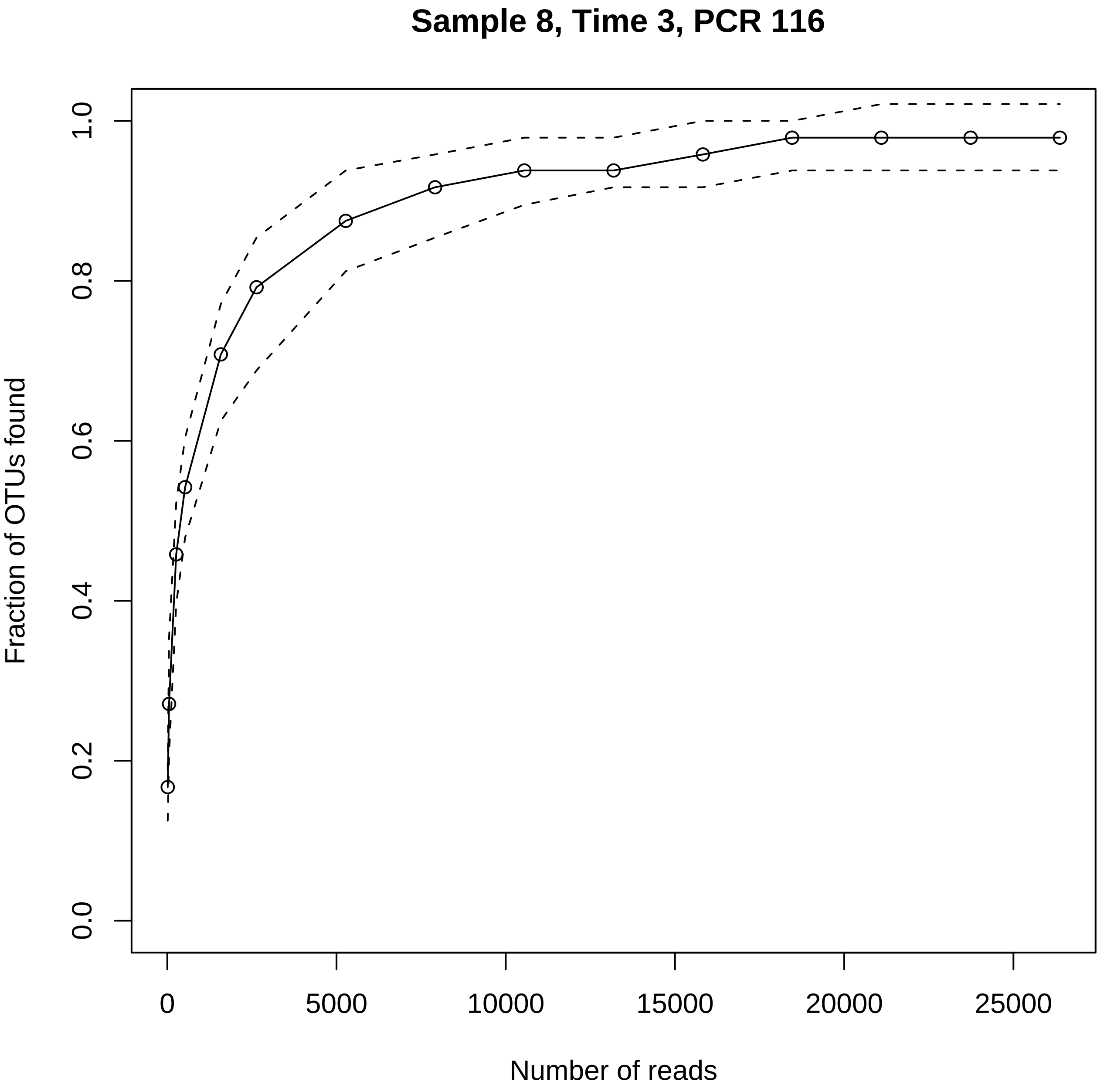
Sample 8, Time 3, PCR 116
Fraction of OTUs found
0.0
0.2
0.4
0.6
0.8
1.0
0
5000
10000
15000
20000
25000
Number of reads

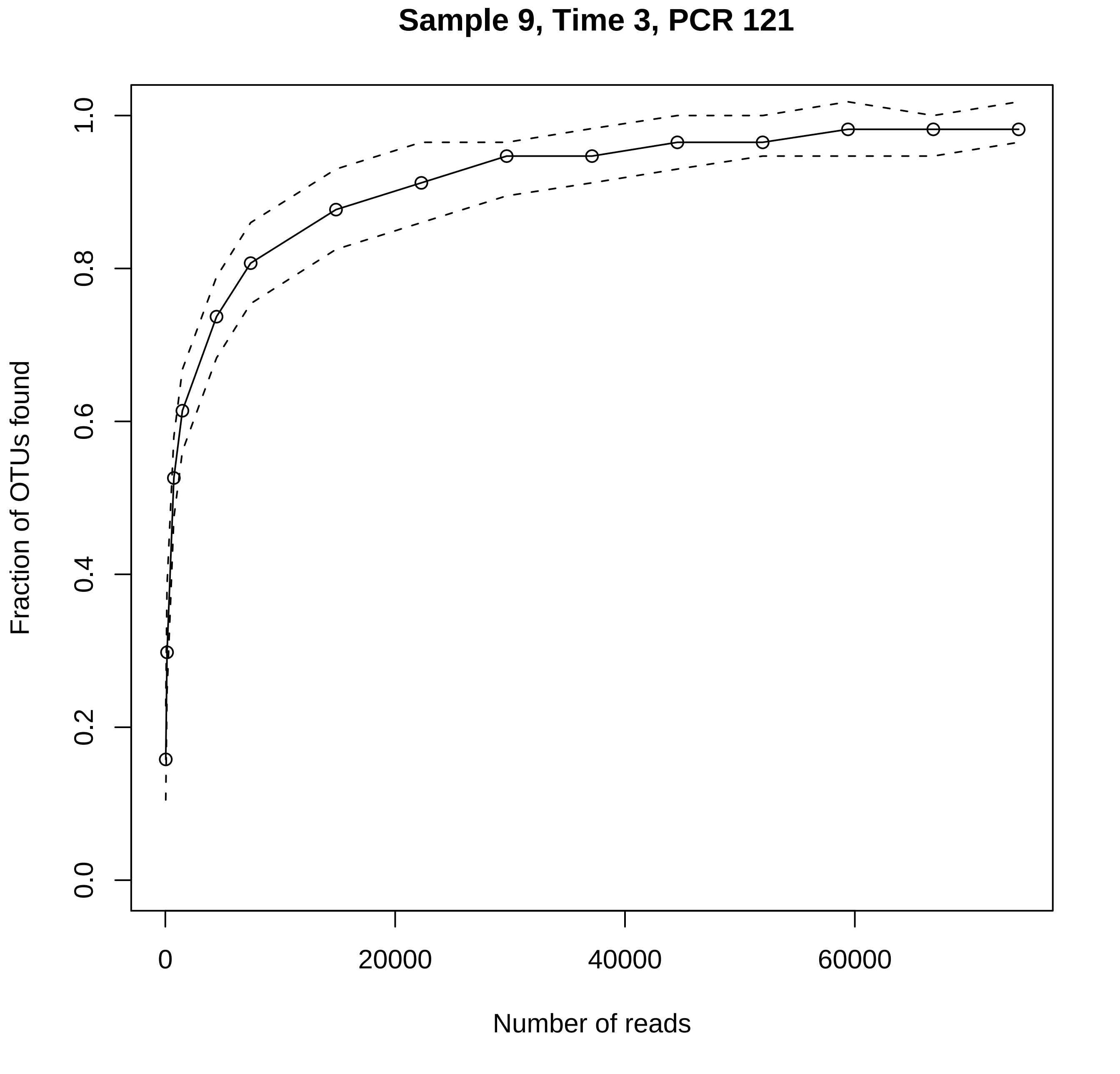
Sample 9, Time 3, PCR 121
Fraction of OTUs found
0.0
0.2
0.4
0.6
0.8
1.0
0
20000
40000
60000
Number of reads

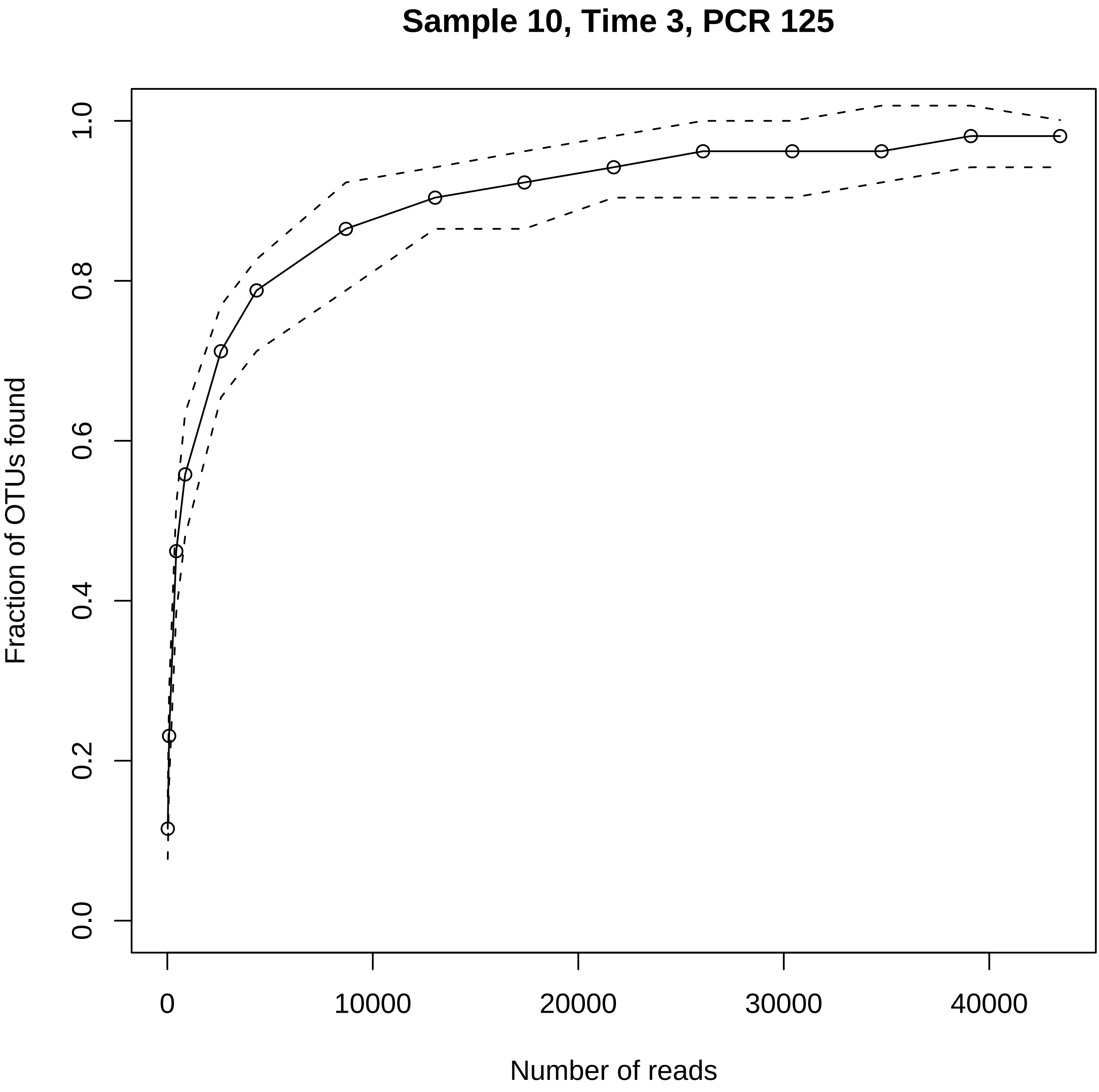
Sample 10, Time 3, PCR 125
Fraction of OTUs found
Number of reads
0.0
0.2
0.4
0.6
0.8
1.0
0
10000
20000
30000
40000

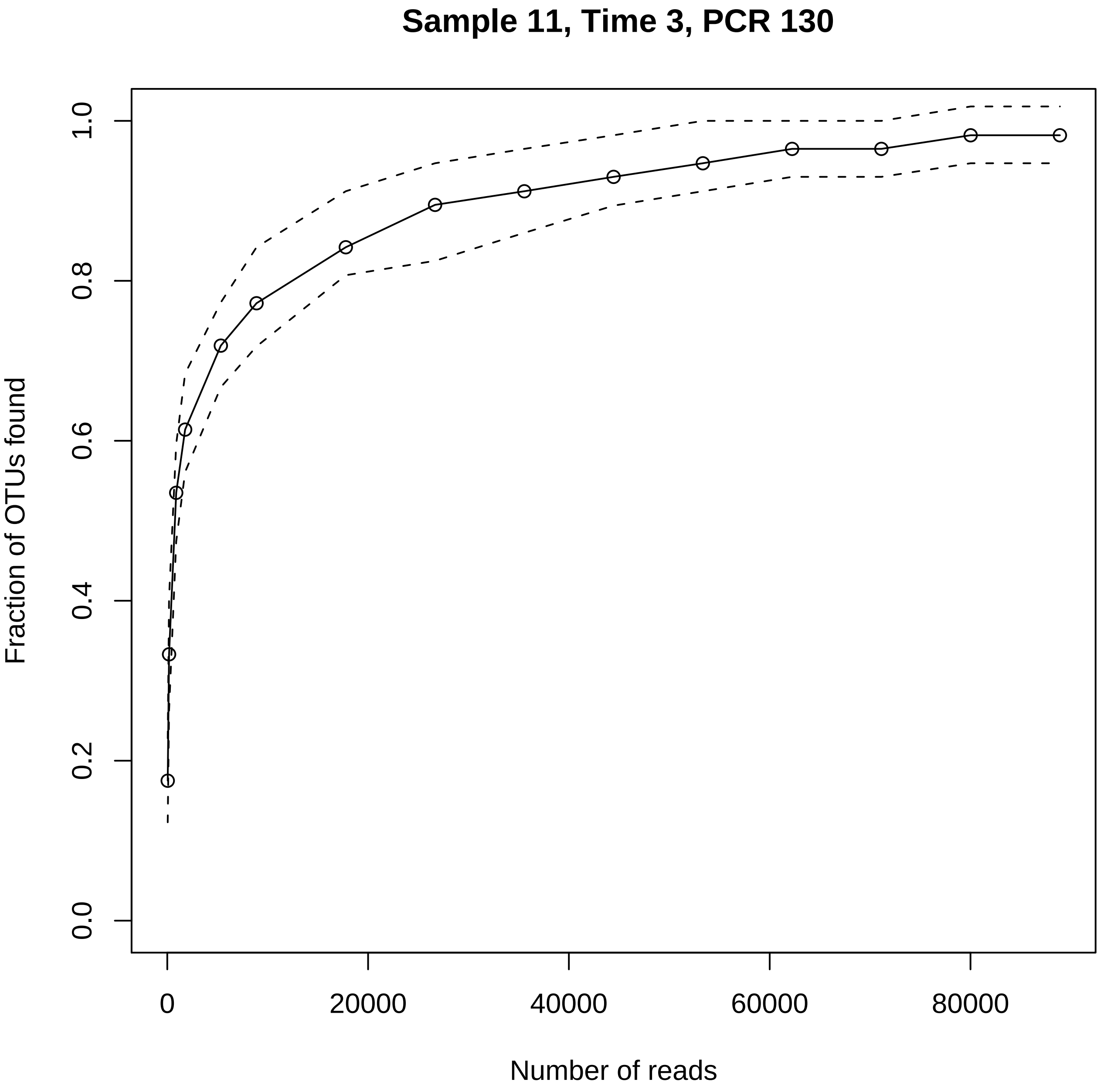

Sample 11, Time 3, PCR 130
Fraction of OTUs found
0.0
0.2
0.4
0.6
0.8
1.0
0
20000
40000
60000
80000
Number of reads

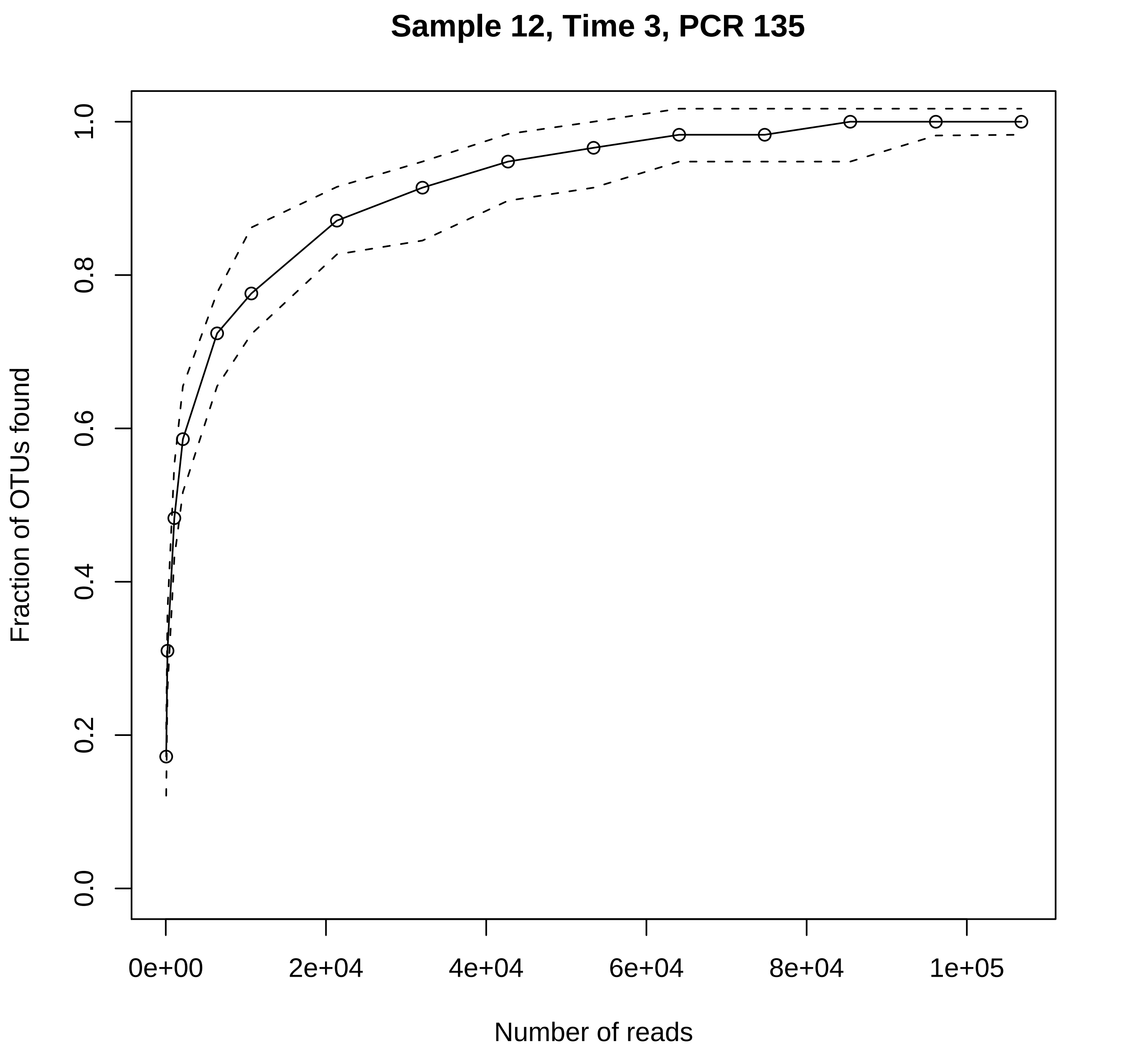
Sample 12, Time 3, PCR 135
Fraction of OTUs found
0.0
0.2
0.4
0.6
0.8
1.0
0e+00
2e+04
4e+04
6e+04
8e+04
1e+05
Number of reads

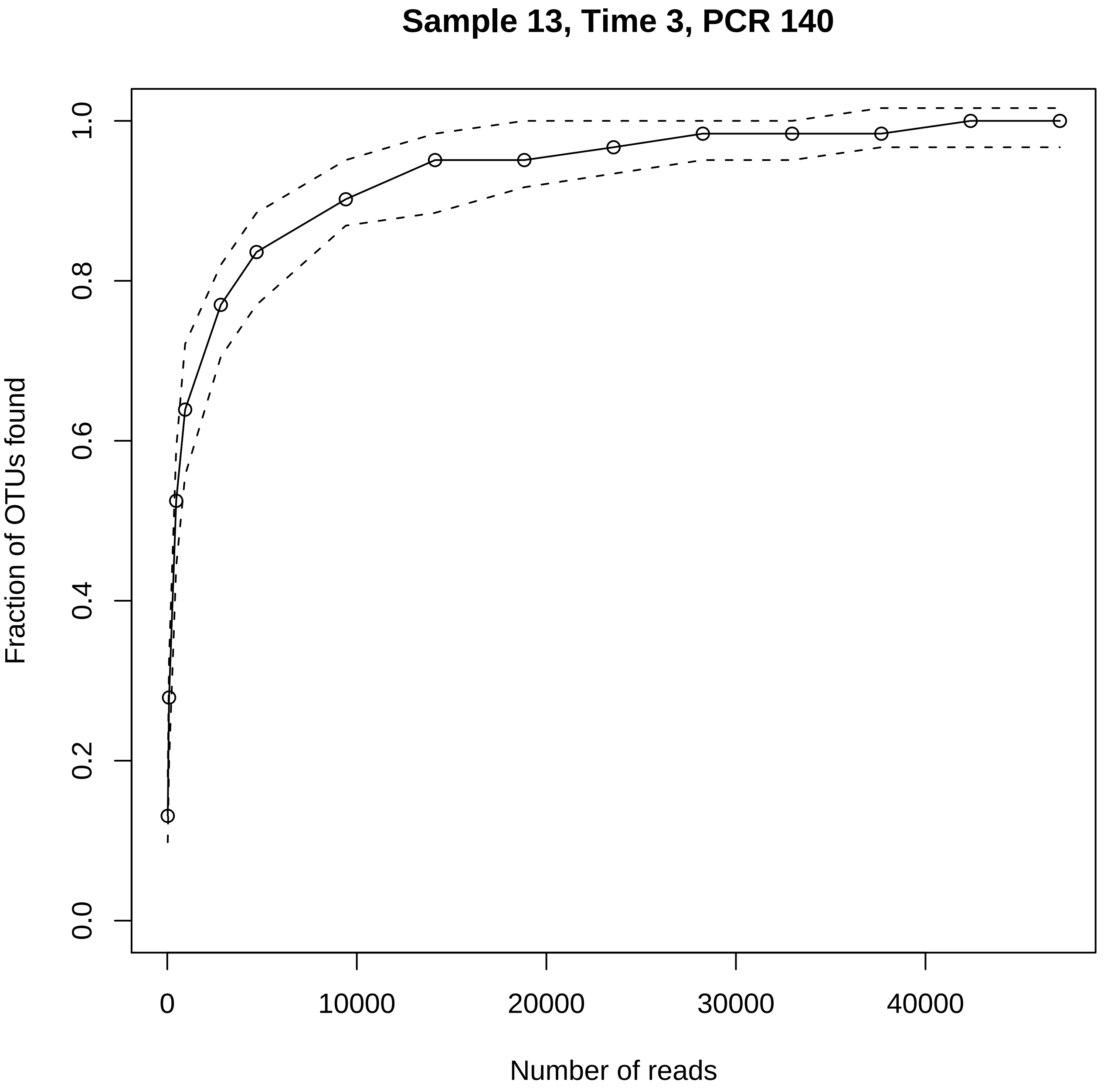
Sample 13, Time 3, PCR 140
Fraction of OTUs found
0.0
0.2
0.4
0.6
0.8
1.0
0
10000
20000
30000
40000
Number of reads

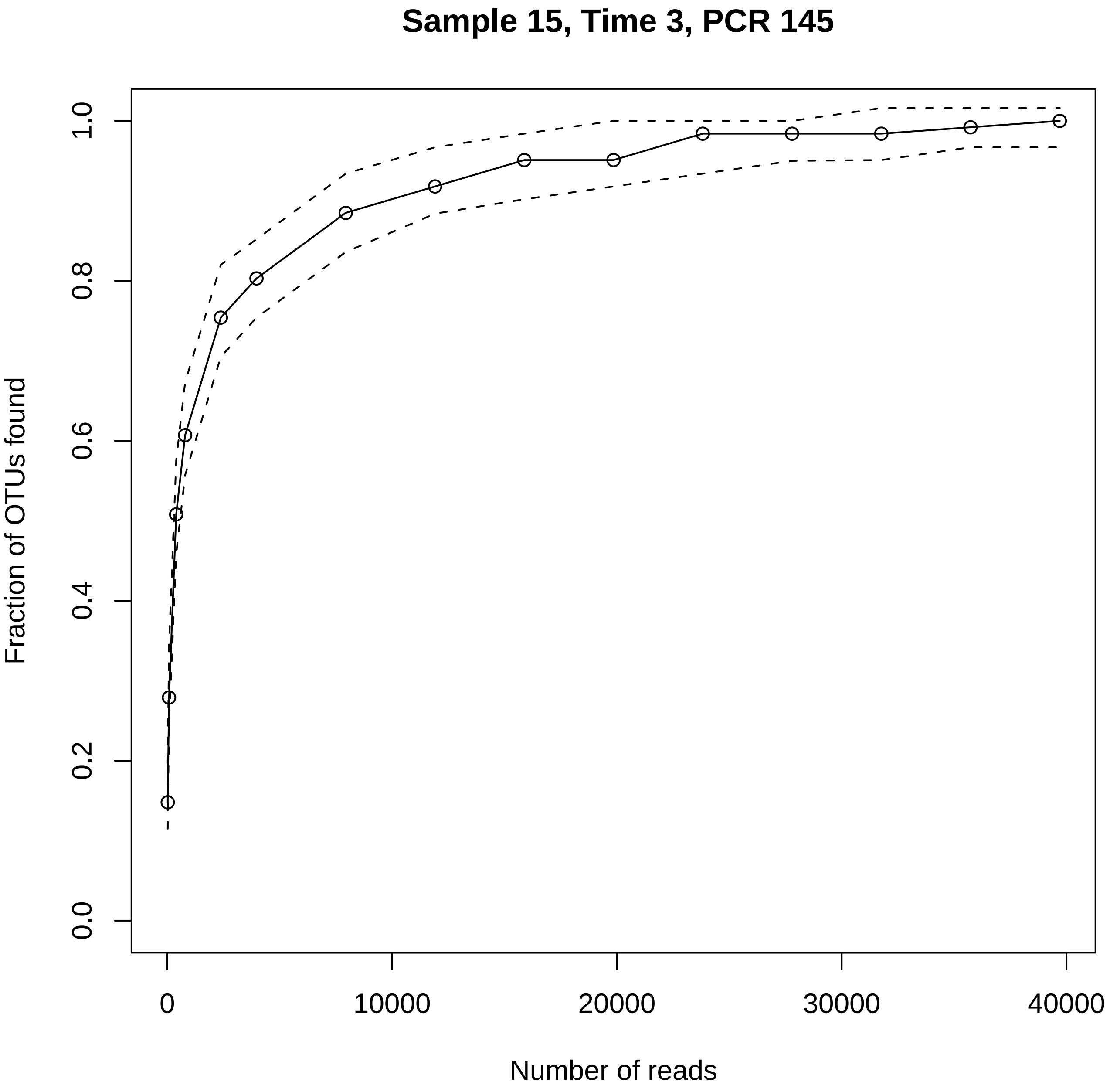
Sample 15, Time 3, PCR 145
Fraction of OTUs found
0.0
0.2
0.4
0.6
0.8
1.0
0
10000
20000
30000
40000
Number of reads

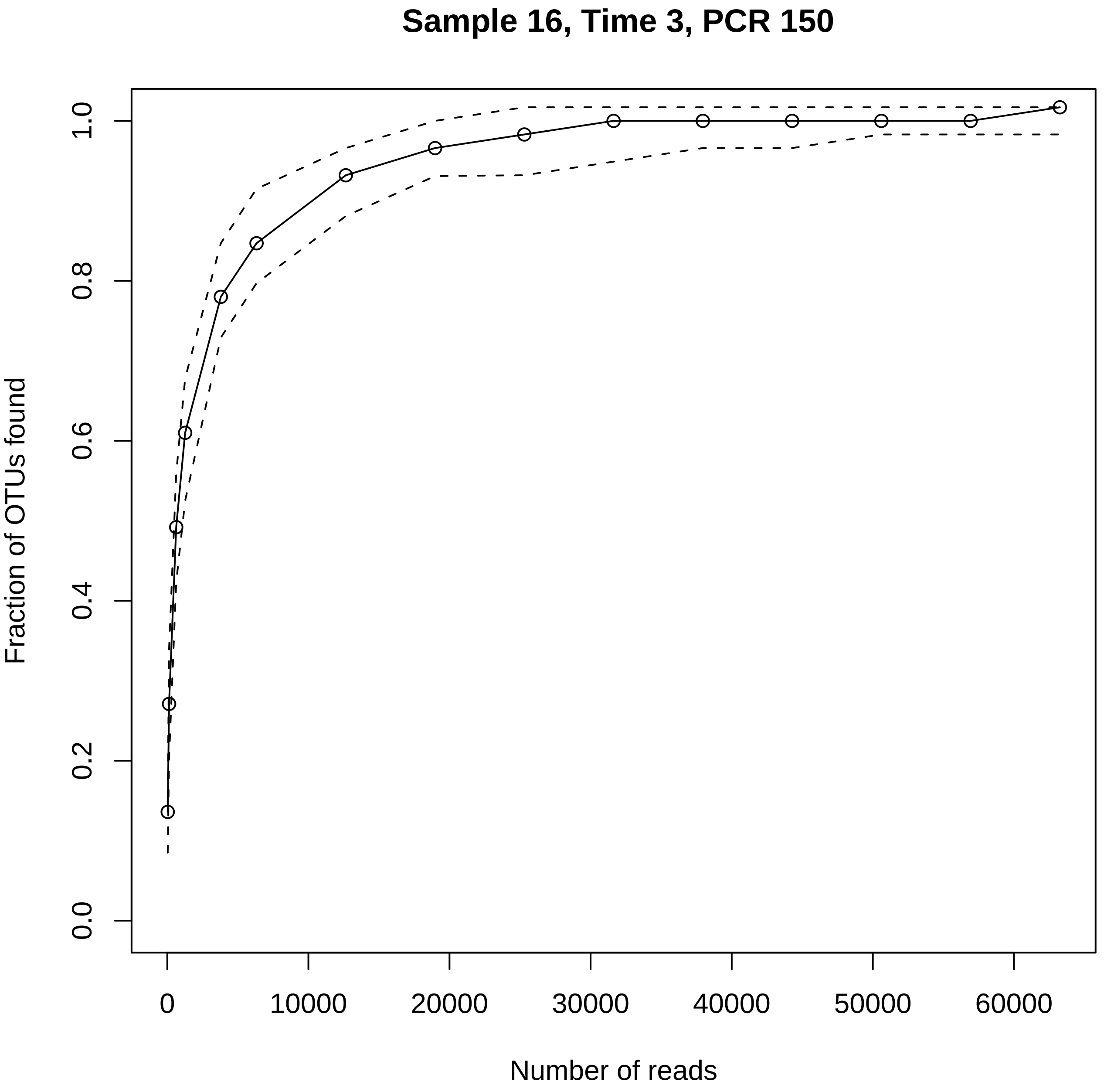
Sample 16, Time 3, PCR 150
Fraction of OTUs found
Number of reads
0.0
0.2
0.4
0.6
0.8
1.0
0
10000
20000
30000
40000
50000
60000

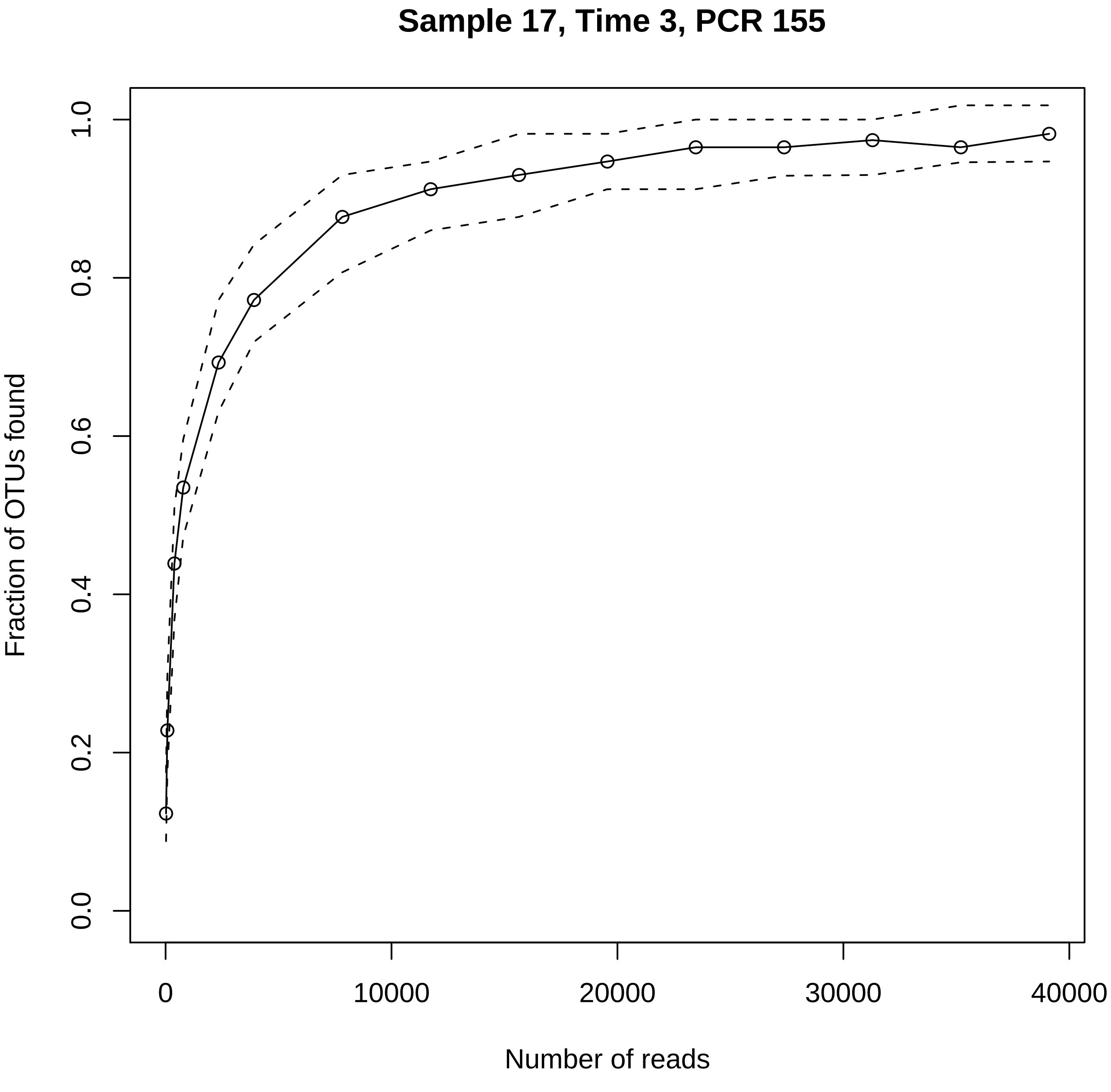

Sample 17, Time 3, PCR 155
Fraction of OTUs found
Number of reads
0.0
0.2
0.4
0.6
0.8
1.0
0
10000
20000
30000
40000

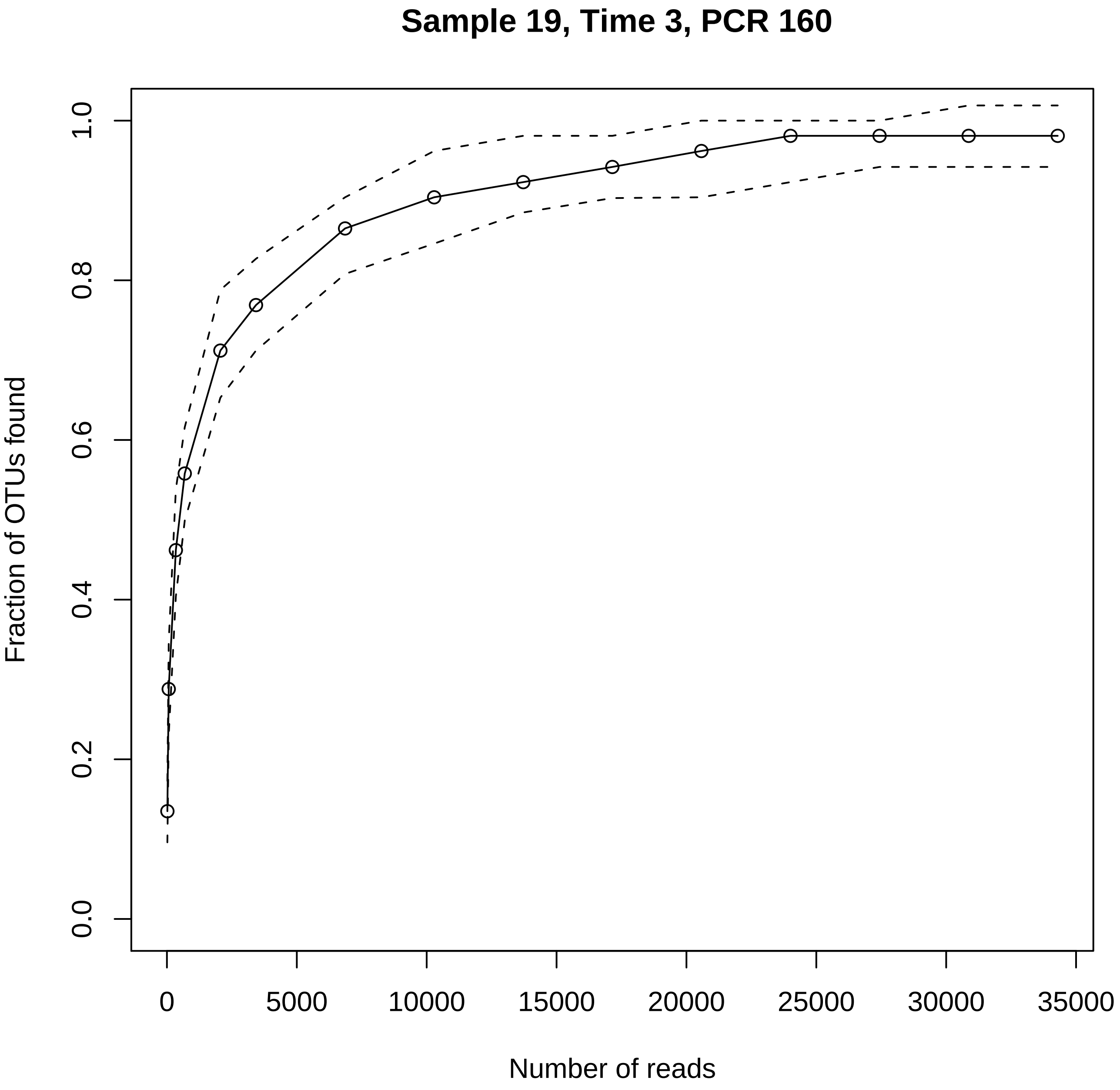

Sample 19, Time 3, PCR 160
Fraction of OTUs found
0.0
0.2
0.4
0.6
0.8
1.0
0
5000
10000
15000
20000
25000
30000
35000
Number of reads

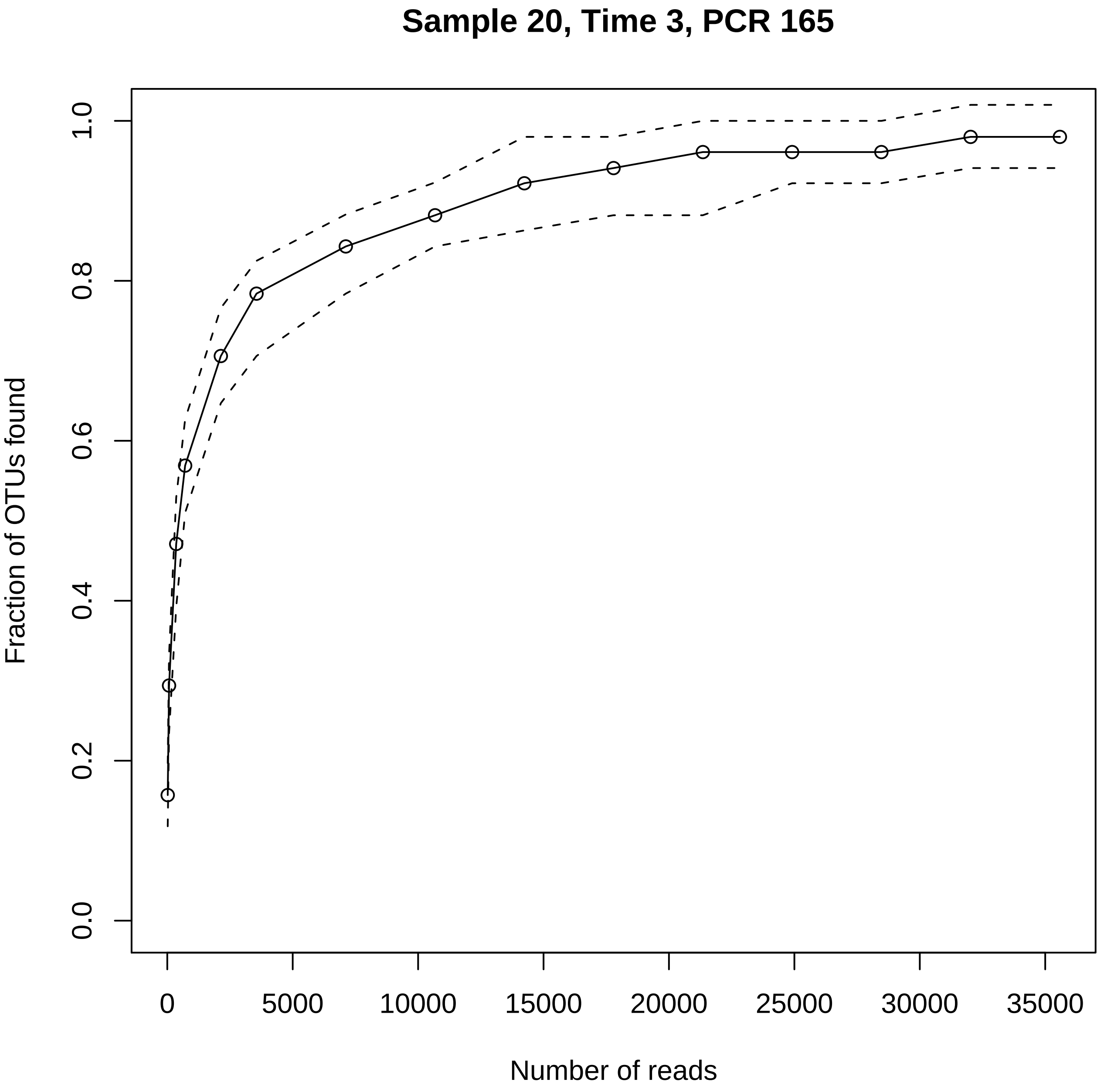

Sample 20, Time 3, PCR 165
Fraction of OTUs found
0.0
0.2
0.4
0.6
0.8
1.0
0
5000
10000
15000
20000
25000
30000
35000
Number of reads

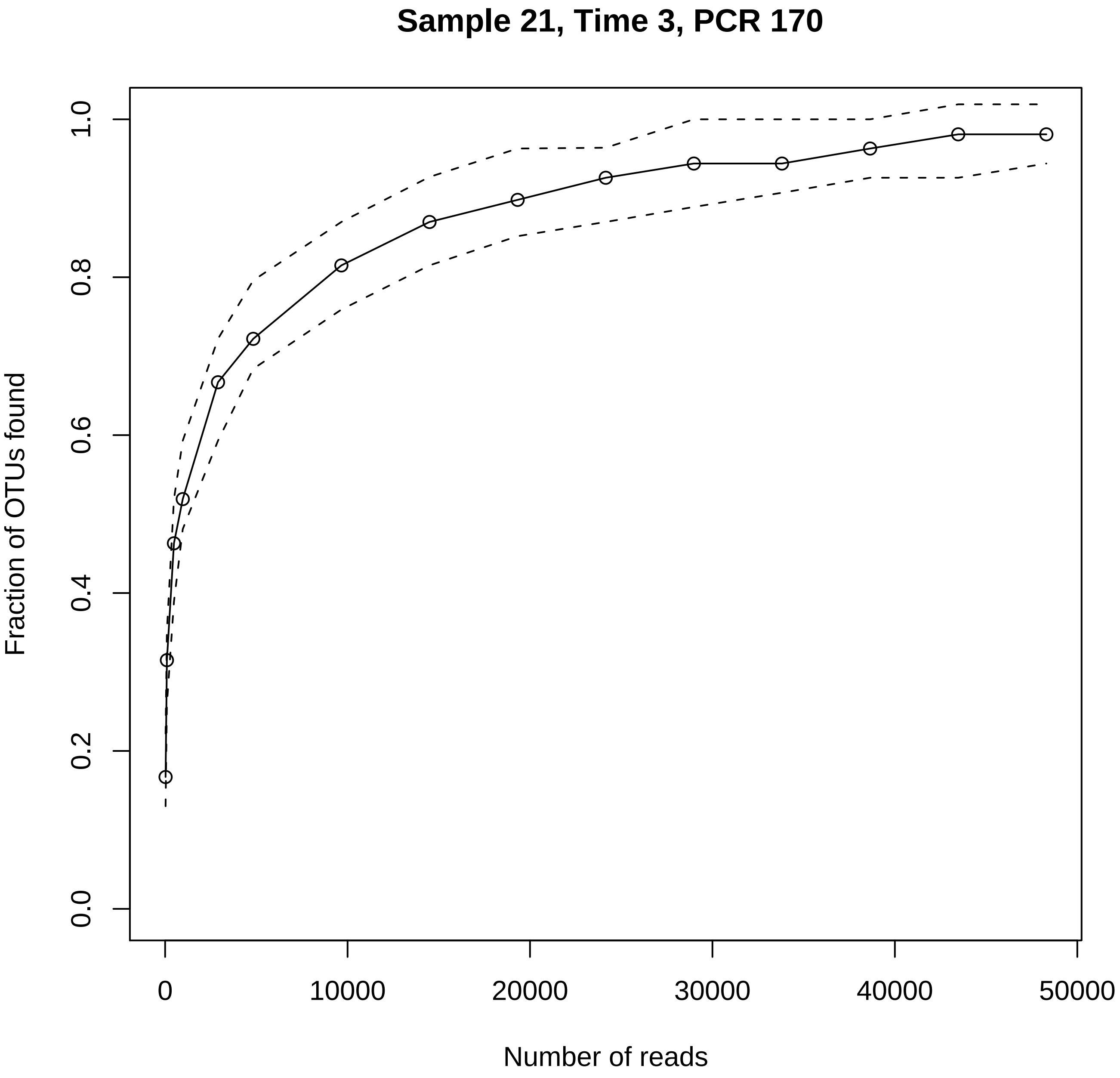
Sample 21, Time 3, PCR 170
Fraction of OTUs found
0.0
0.2
0.4
0.6
0.8
1.0
0
10000
20000
30000
40000
50000
Number of reads

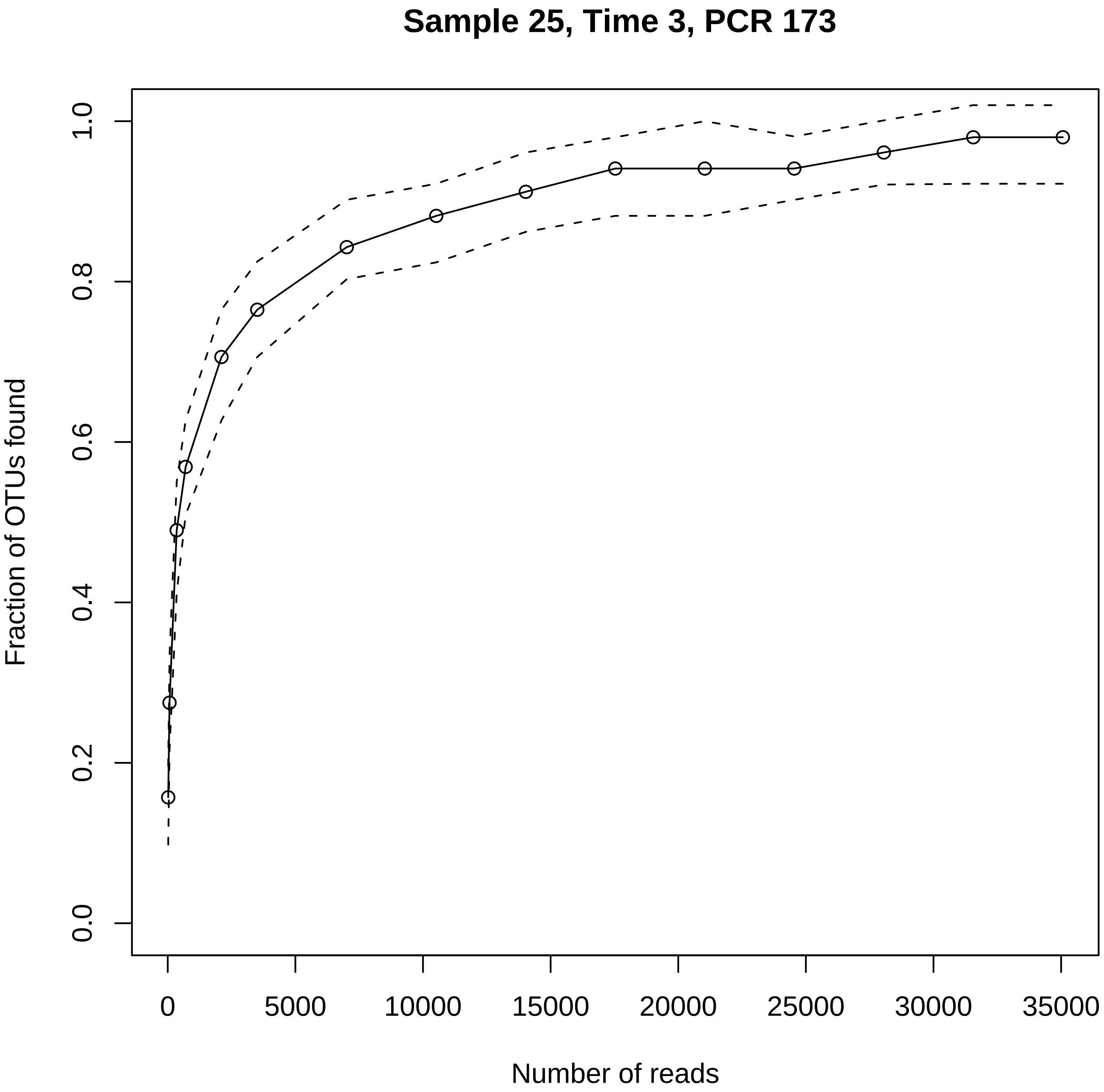

Sample 25, Time 3, PCR 173
Fraction of OTUs found
0.0
0.2
0.4
0.6
0.8
1.0
0
5000
10000
15000
20000
25000
30000
35000
Number of reads

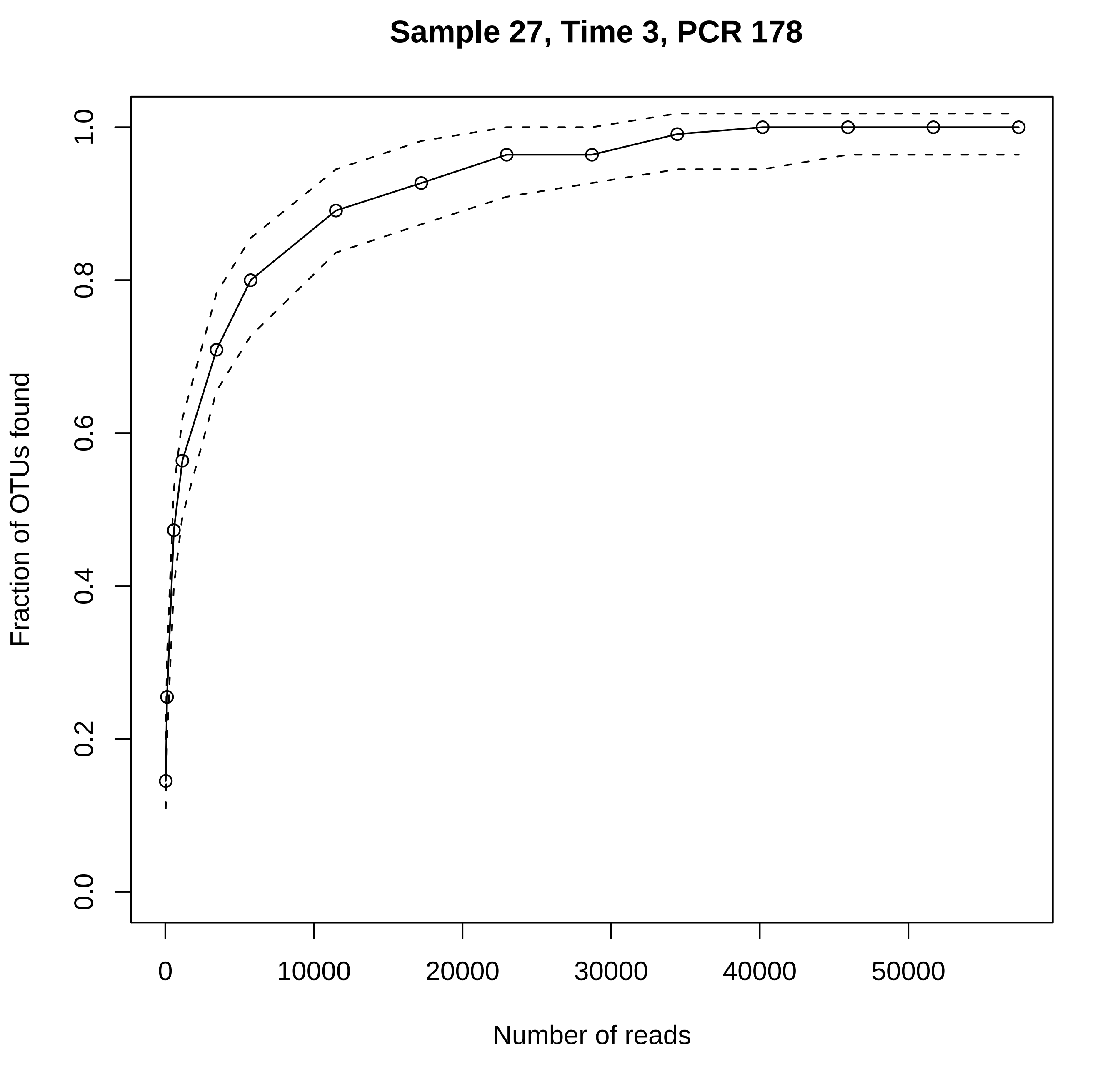
Sample 27, Time 3, PCR 178
Fraction of OTUs found
0.0
0.2
0.4
0.6
0.8
1.0
Number of reads
0
10000
20000
30000
40000
50000

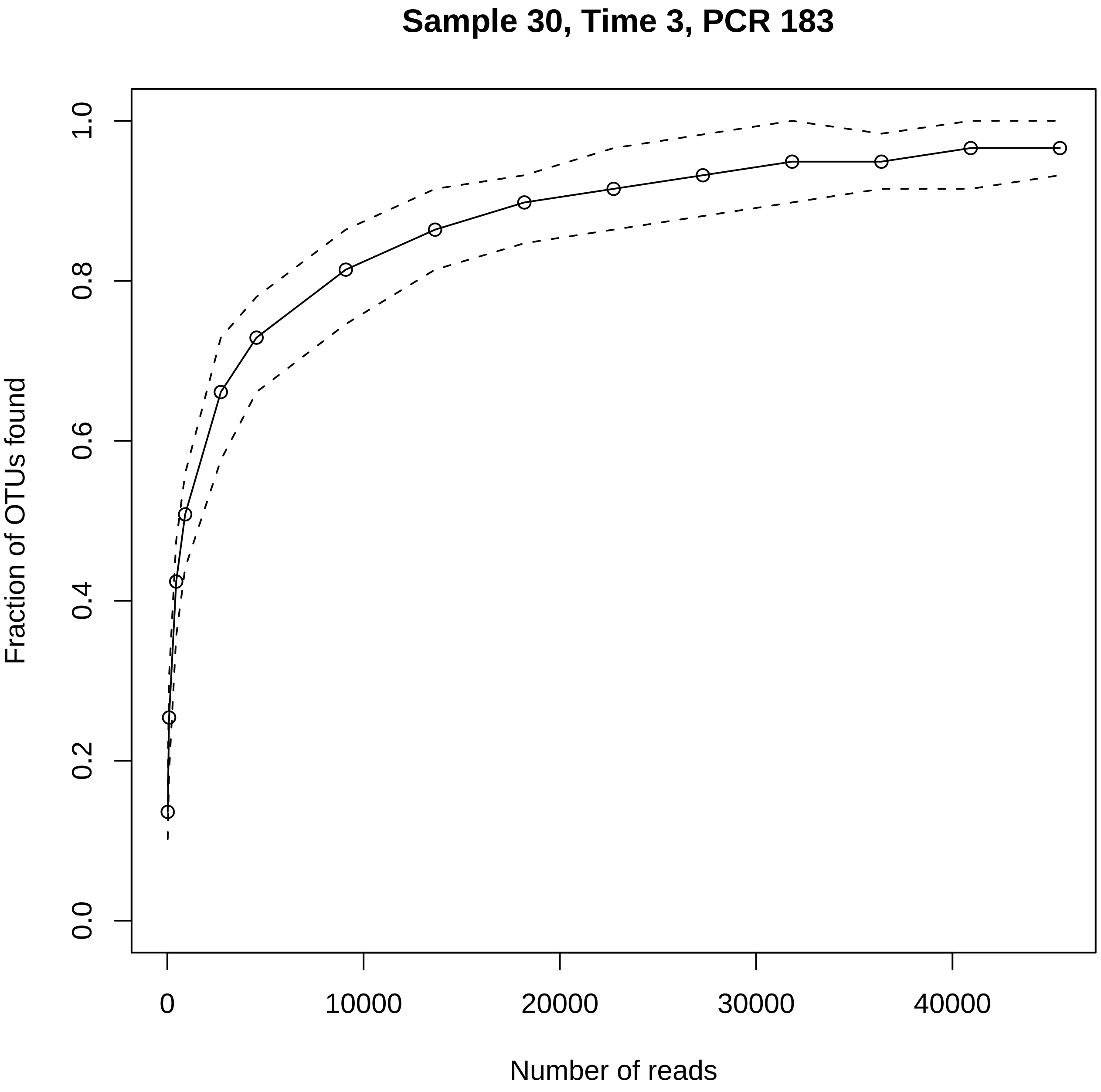
Sample 30, Time 3, PCR 183
Fraction of OTUs found
Number of reads
0.0
0.2
0.4
0.6
0.8
1.0
0
10000
20000
30000
40000

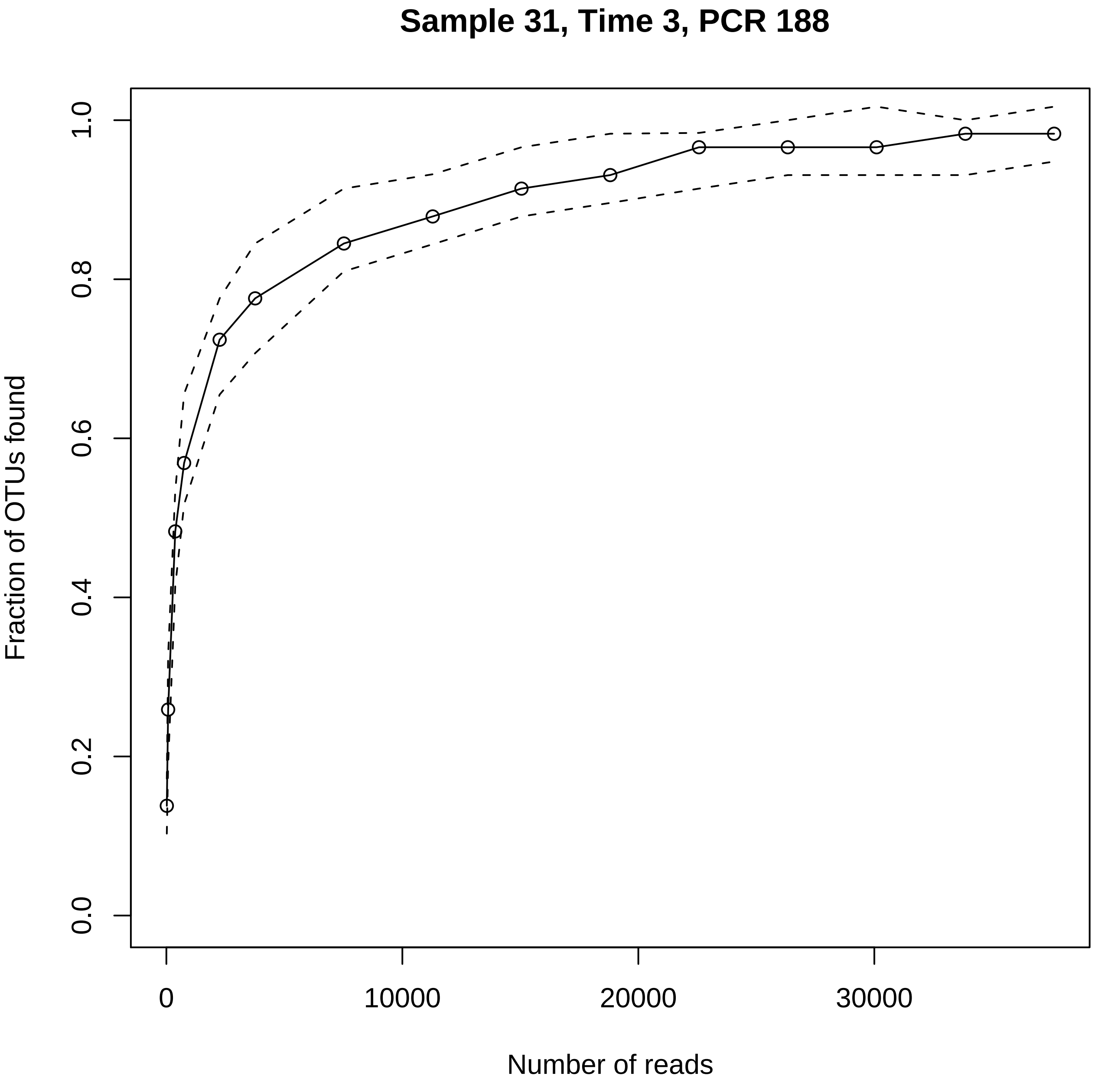
Sample 31, Time 3, PCR 188
Fraction of OTUs found
0.0
0.2
0.4
0.6
0.8
1.0
0
10000
20000
30000
Number of reads

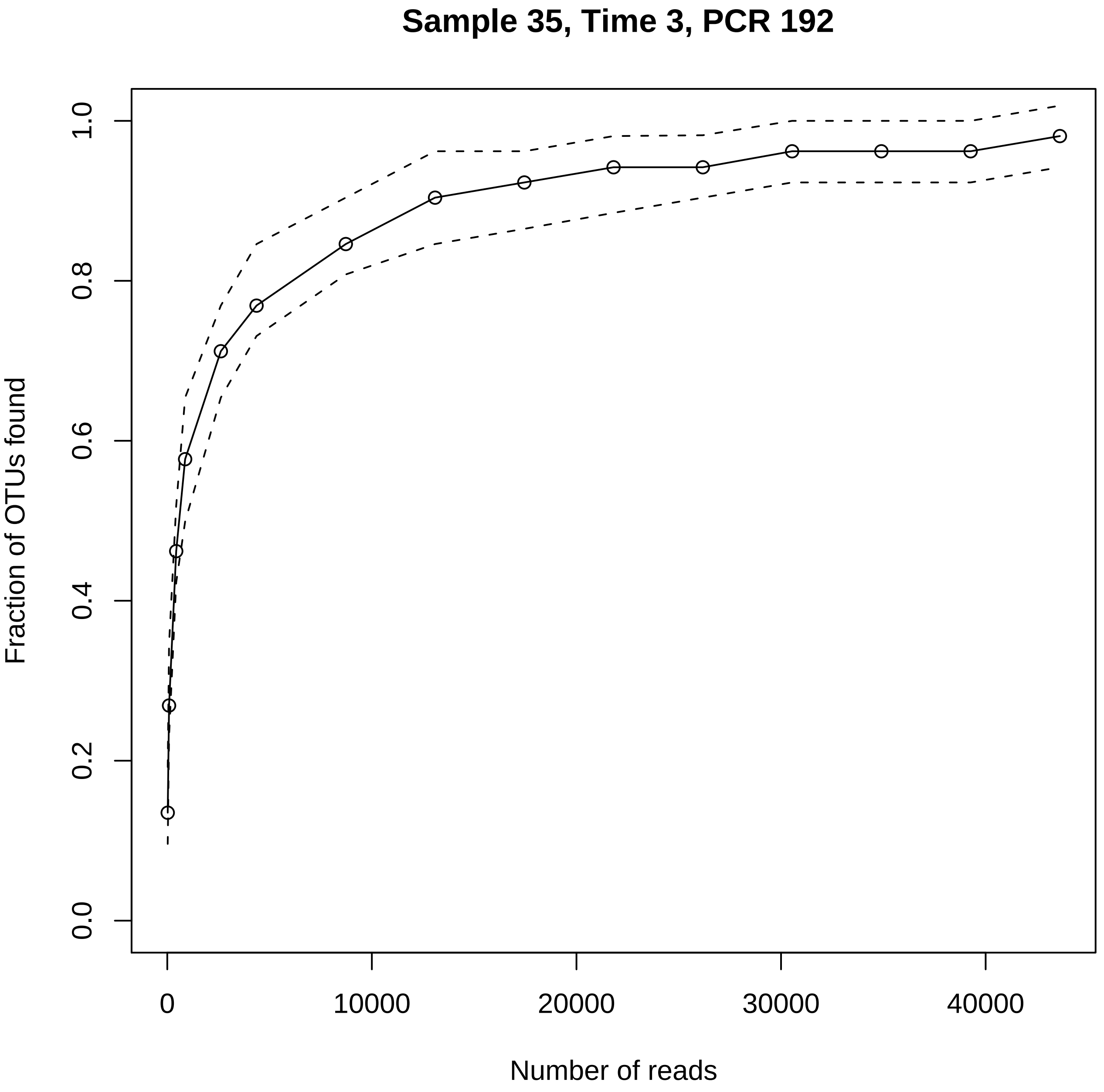

Sample 35, Time 3, PCR 192
Fraction of OTUs found
0.0
0.2
0.4
0.6
0.8
1.0
0
10000
20000
30000
40000
Number of reads

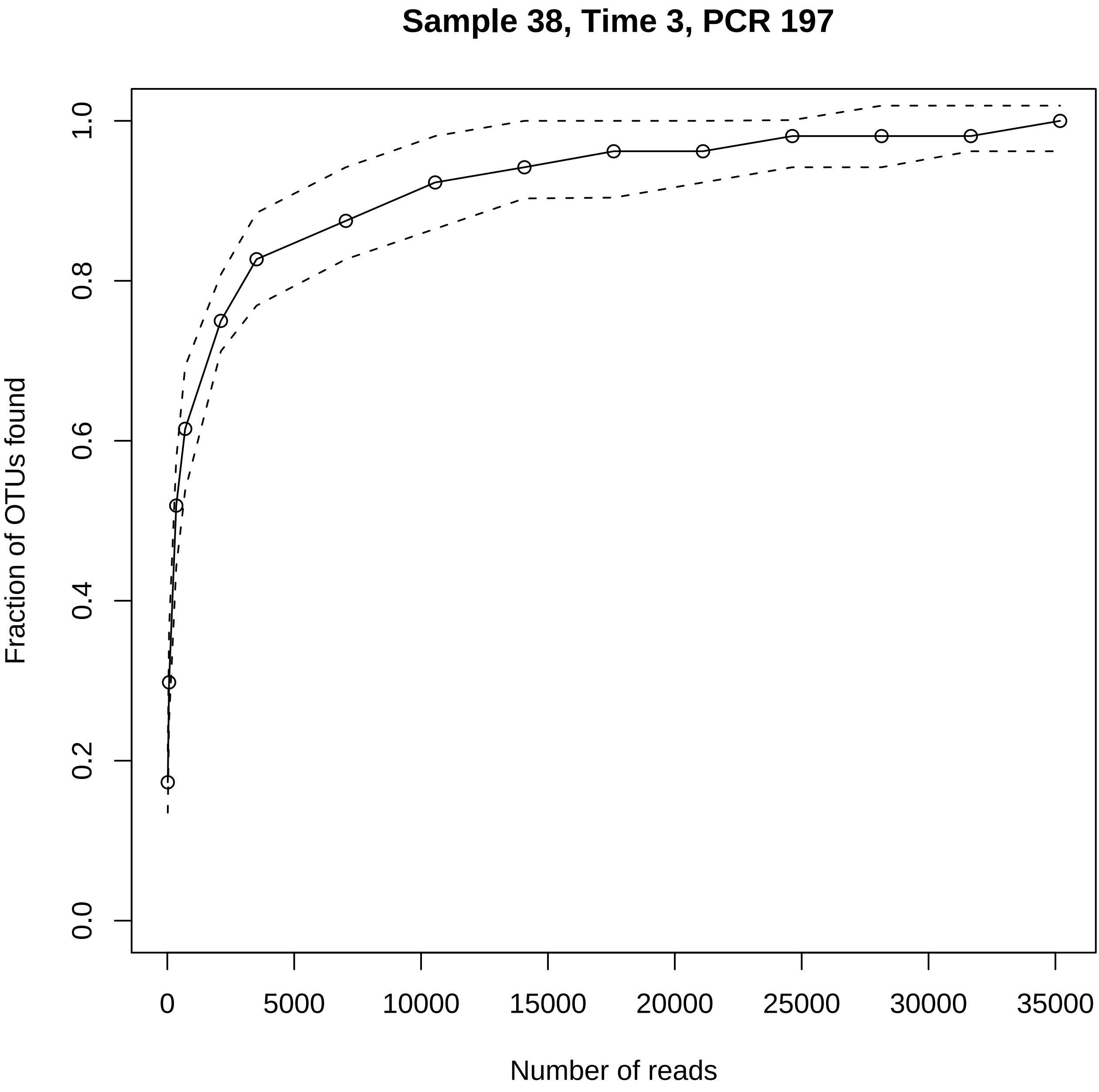
Sample 38, Time 3, PCR 197
Fraction of OTUs found
0.0
0.2
0.4
0.6
0.8
1.0
Number of reads
0
5000
10000
15000
20000
25000
30000
35000

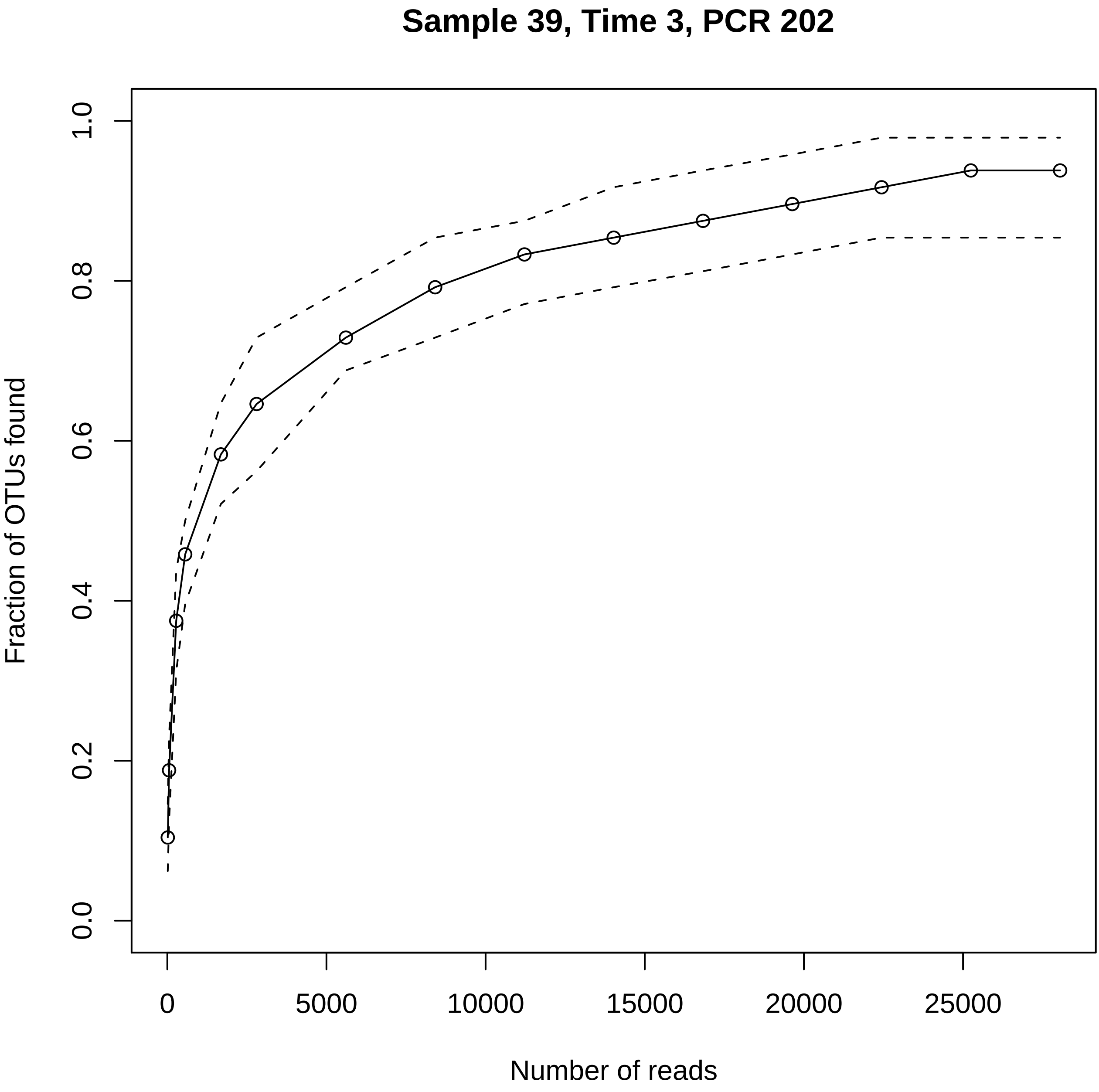

Sample 39, Time 3, PCR 202
Fraction of OTUs found
Number of reads
0.0
0.2
0.4
0.6
0.8
1.0
0
5000
10000
15000
20000
25000

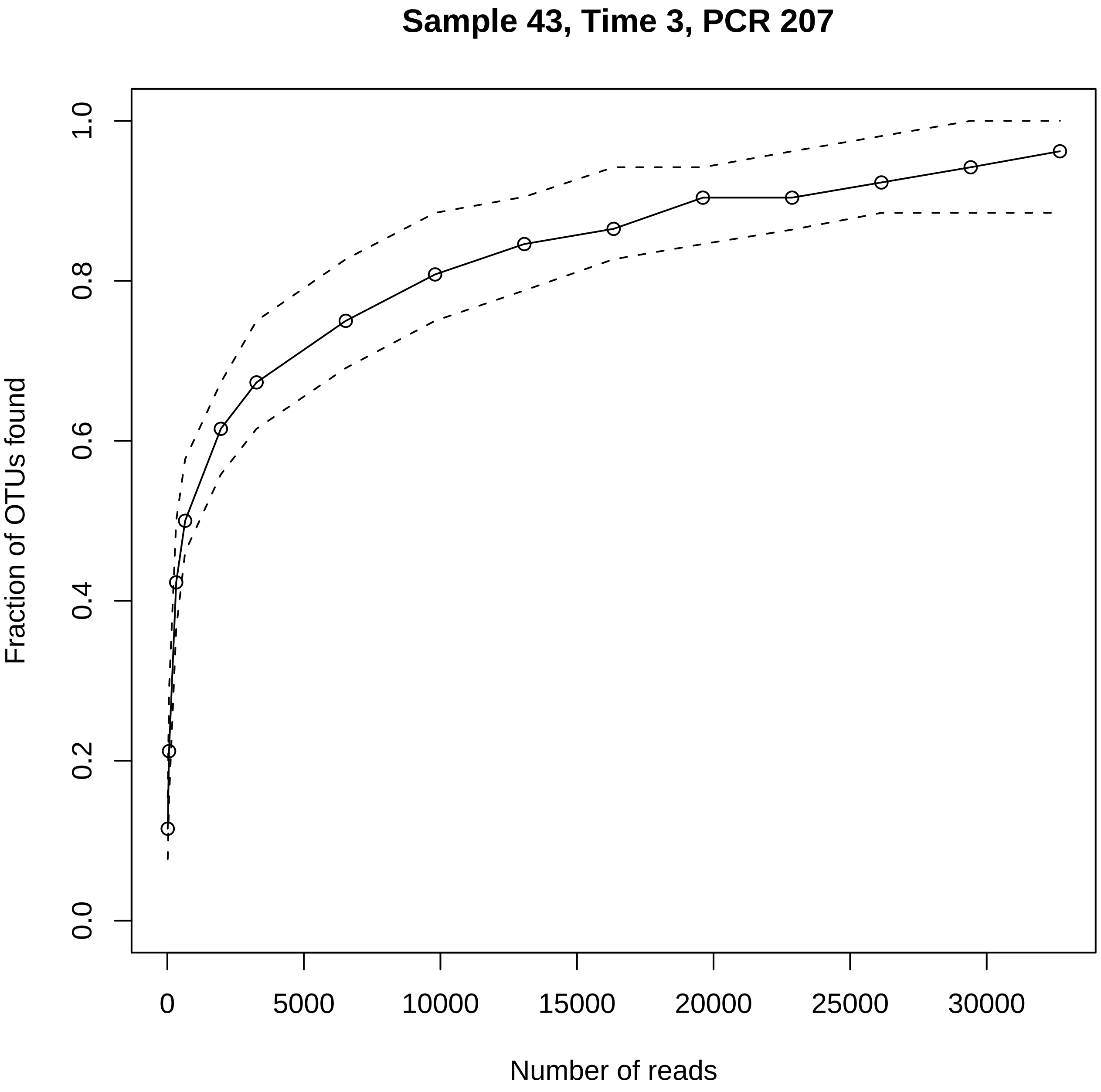
Sample 43, Time 3, PCR 207
Fraction of OTUs found
0.0
0.2
0.4
0.6
0.8
1.0
0
5000
10000
15000
20000
25000
30000
Number of reads

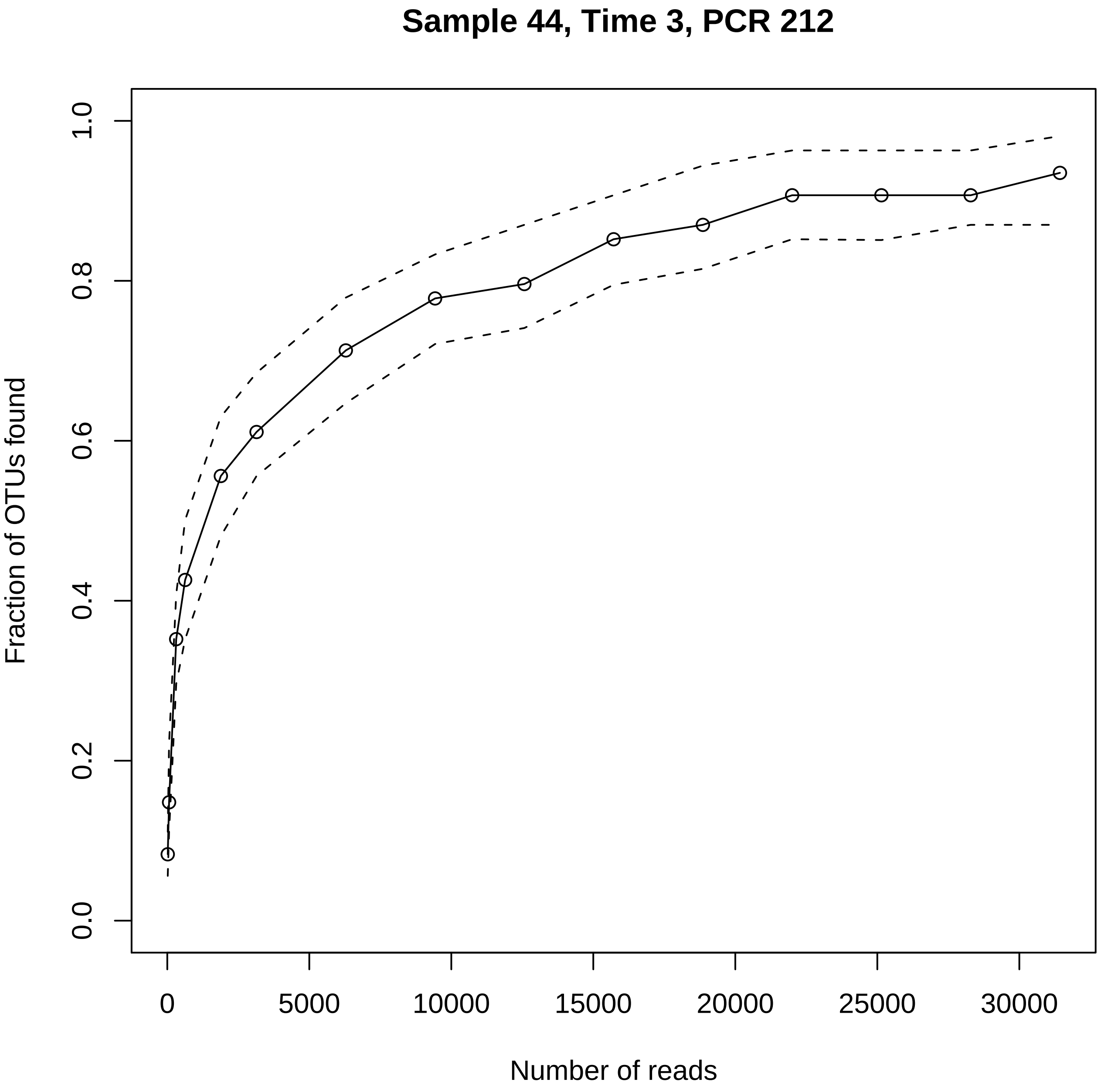
Sample 44, Time 3, PCR 212
Fraction of OTUs found
0.0
0.2
0.4
0.6
0.8
1.0
0
5000
10000
15000
20000
25000
30000
Number of reads

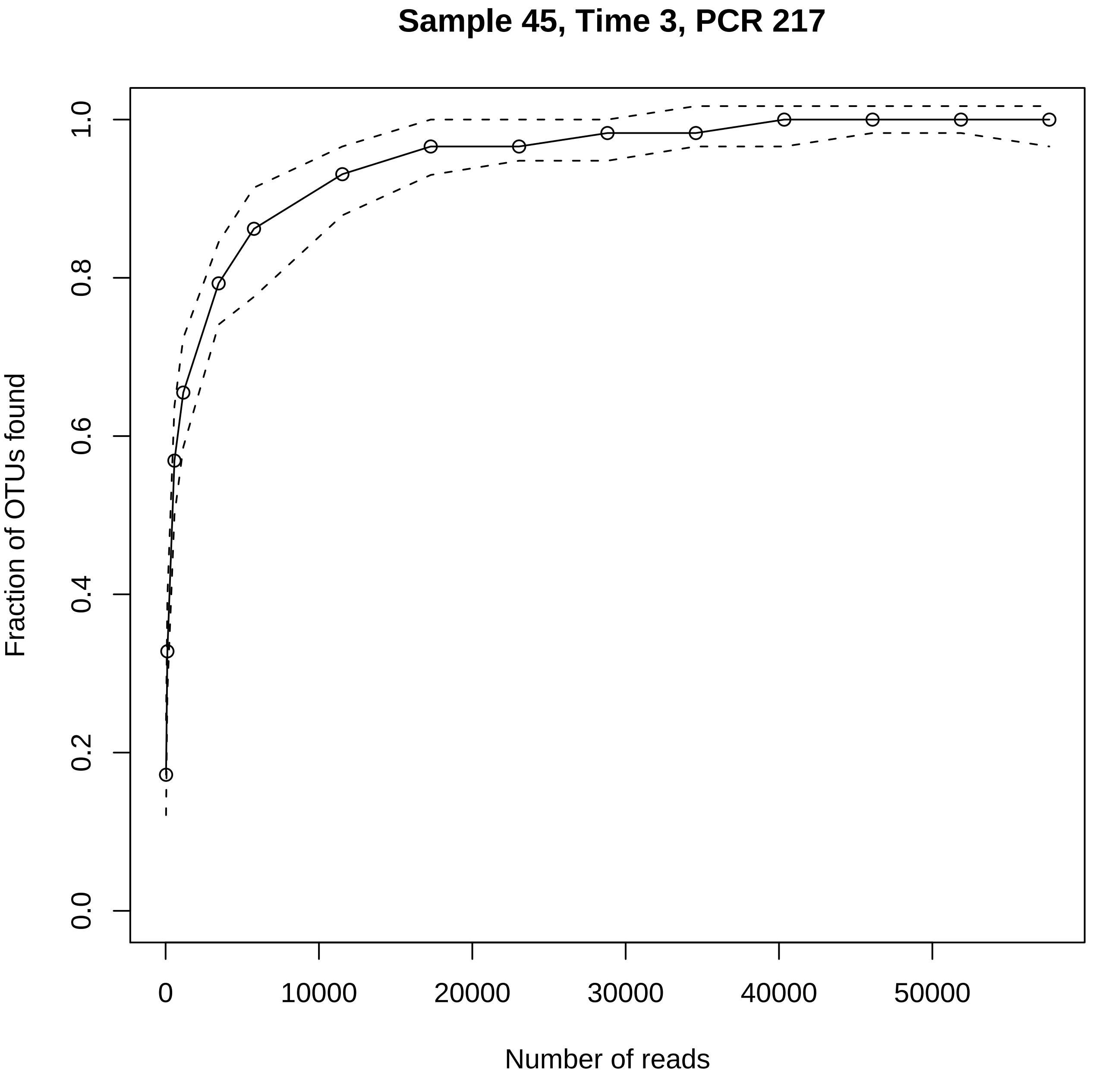
Sample 45, Time 3, PCR 217
Fraction of OTUs found
0.0
0.2
0.4
0.6
0.8
1.0
0
10000
20000
30000
40000
50000
Number of reads

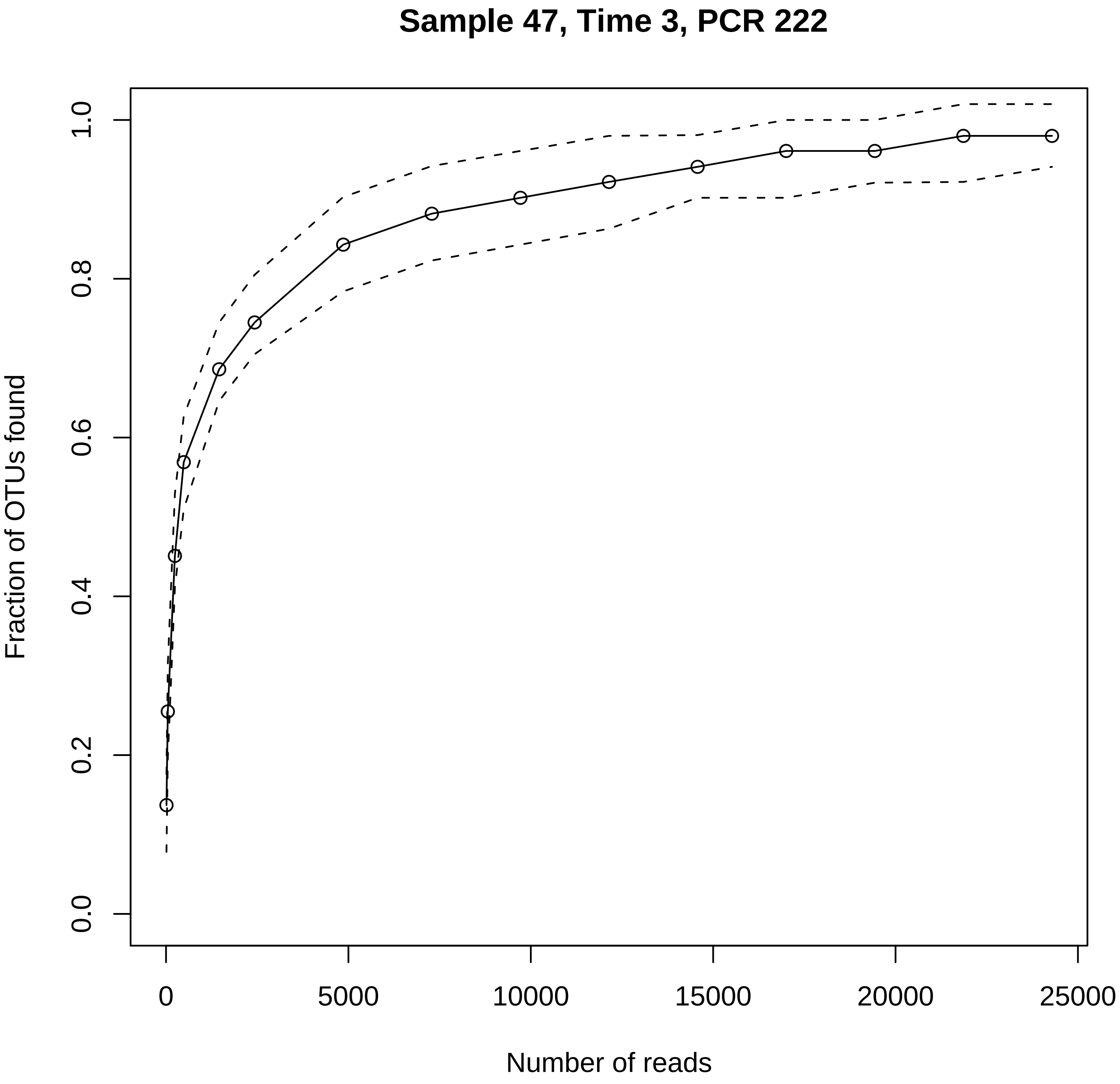
Sample 47, Time 3, PCR 222
Fraction of OTUs found
Number of reads
0.0
0.2
0.4
0.6
0.8
1.0
0
5000
10000
15000
20000
25000

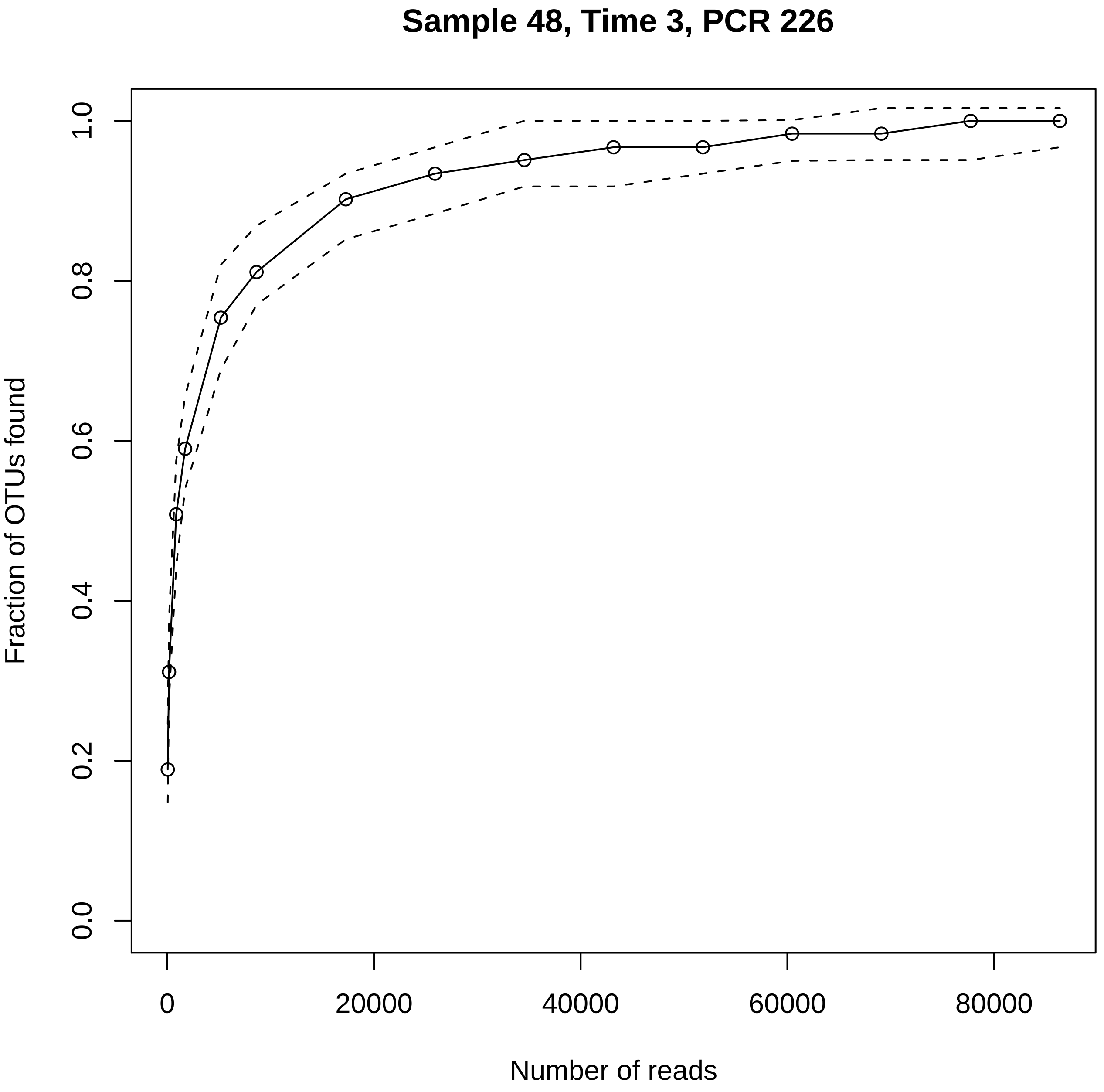

Sample 48, Time 3, PCR 226
Fraction of OTUs found
0.0
0.2
0.4
0.6
0.8
1.0
0
20000
40000
60000
80000
Number of reads

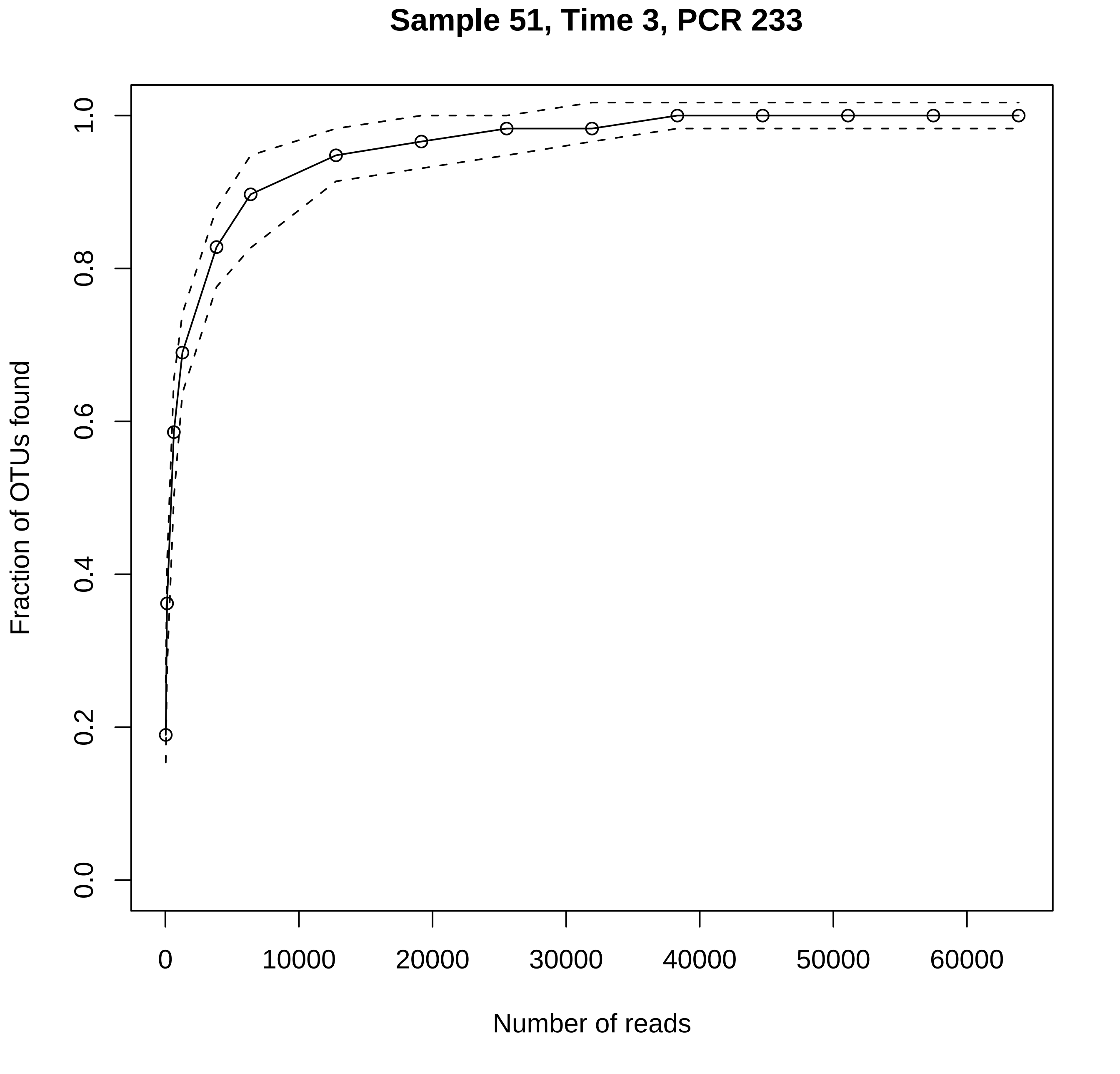
Sample 51, Time 3, PCR 233
Fraction of OTUs found
0.0
0.2
0.4
0.6
0.8
1.0
0
10000
20000
30000
40000
50000
60000
Number of reads

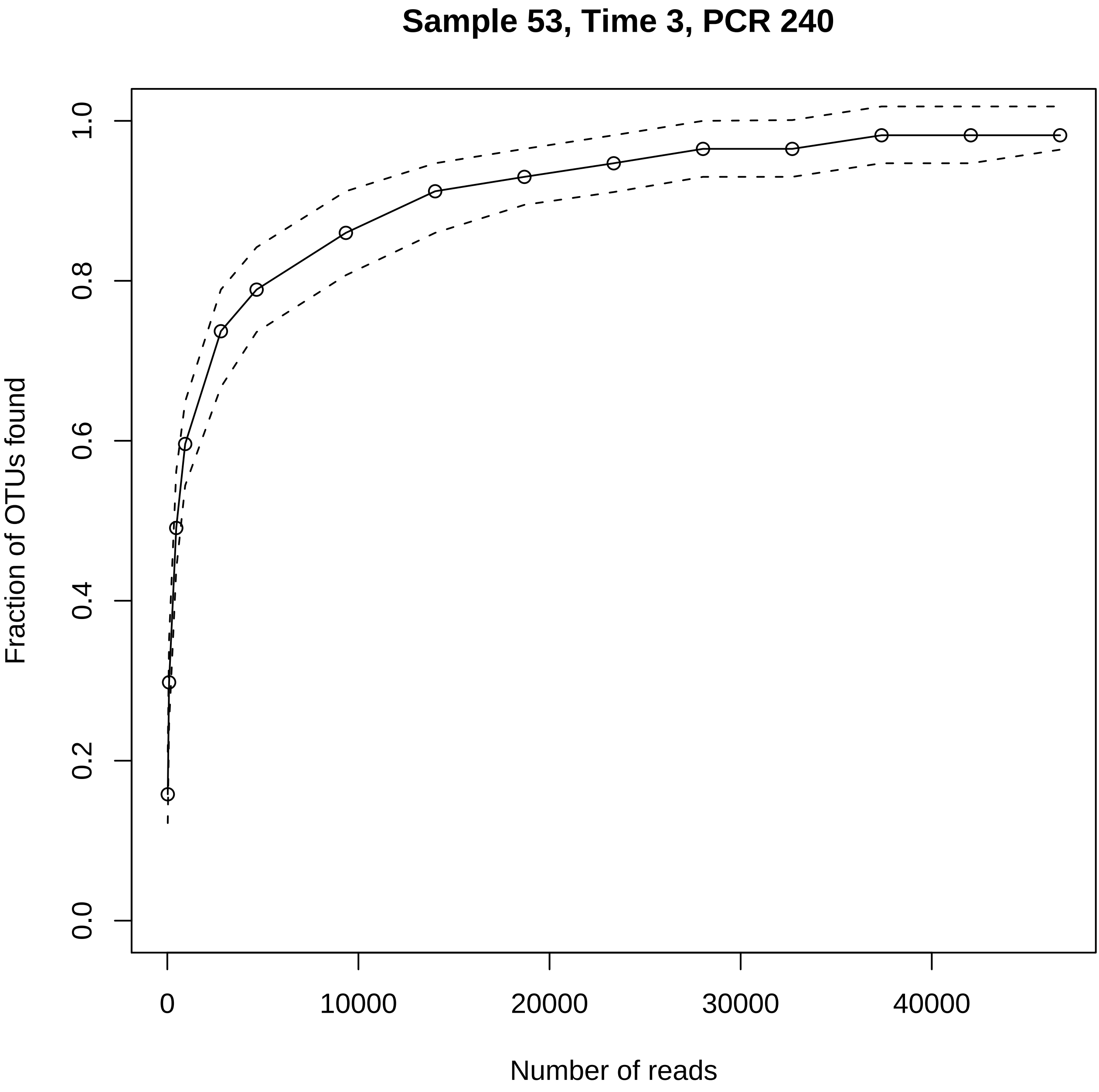
Sample 53, Time 3, PCR 240
Fraction of OTUs found
0.0
0.2
0.4
0.6
0.8
1.0
0
10000
20000
30000
40000
Number of reads

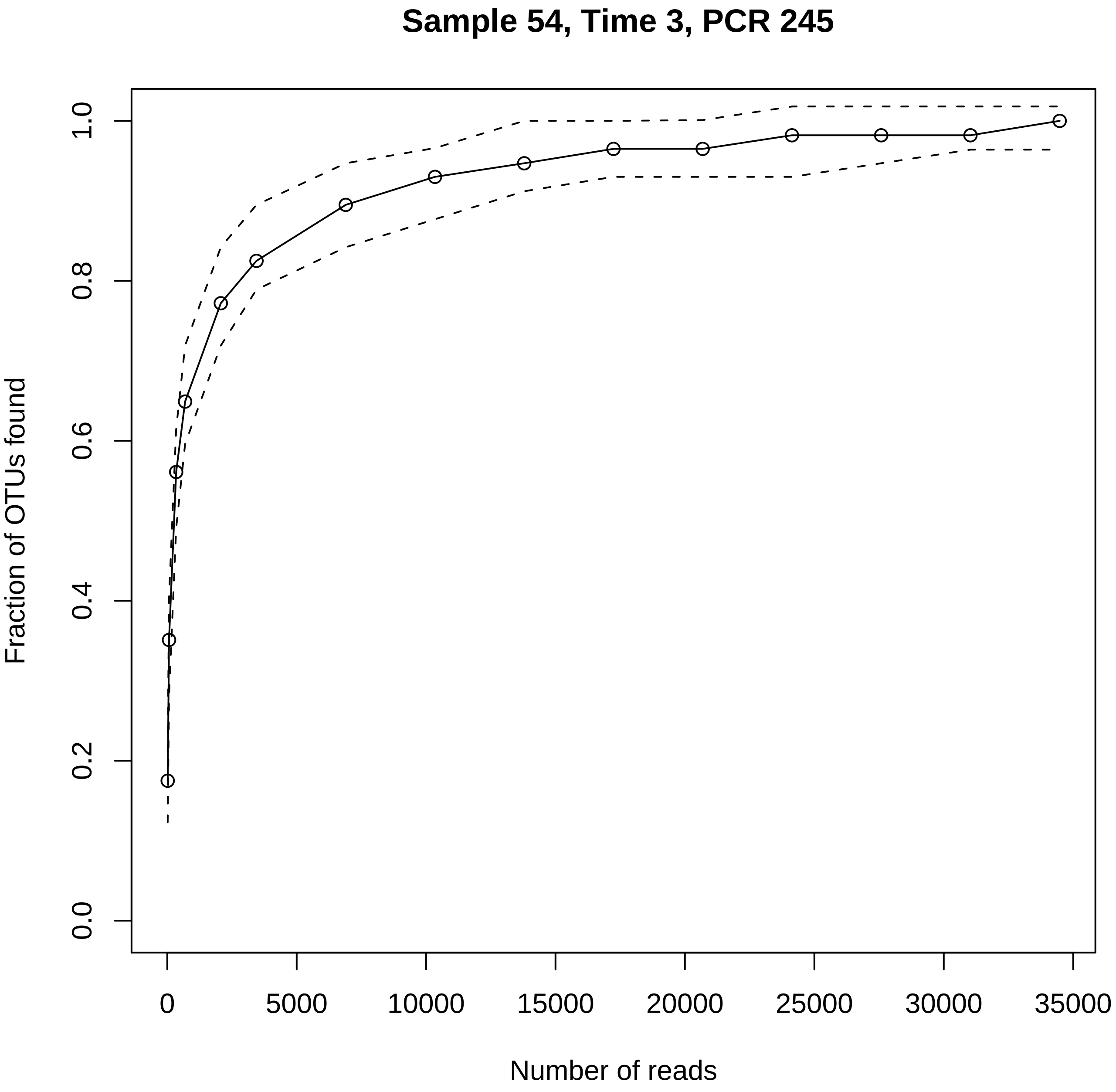
Sample 54, Time 3, PCR 245
Fraction of OTUs found
0.0
0.2
0.4
0.6
0.8
1.0
0
5000
10000
15000
20000
25000
30000
35000
Number of reads

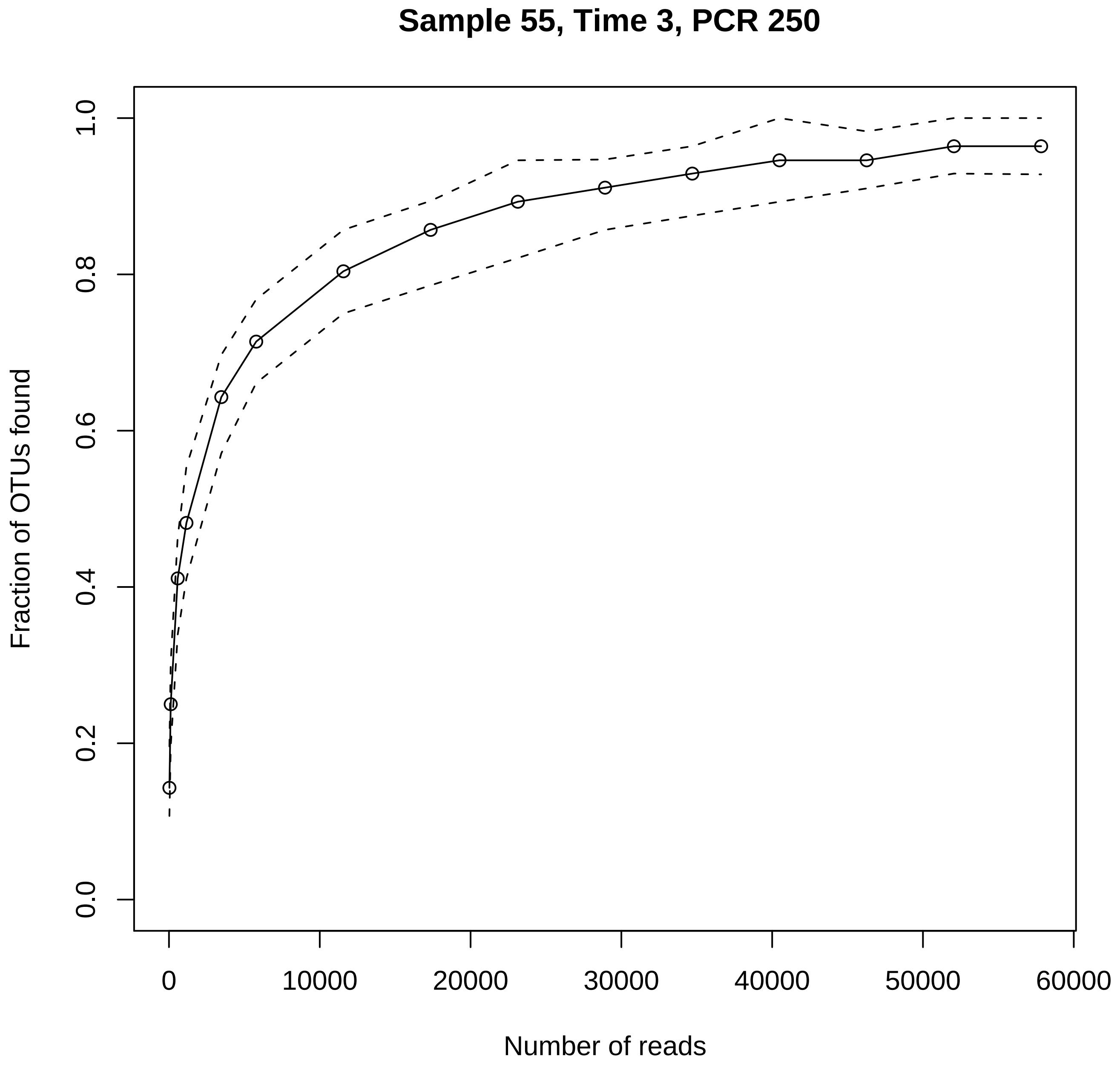
Sample 55, Time 3, PCR 250
Fraction of OTUs found
0.0
0.2
0.4
0.6
0.8
1.0
0
10000
20000
30000
40000
50000
60000
Number of reads

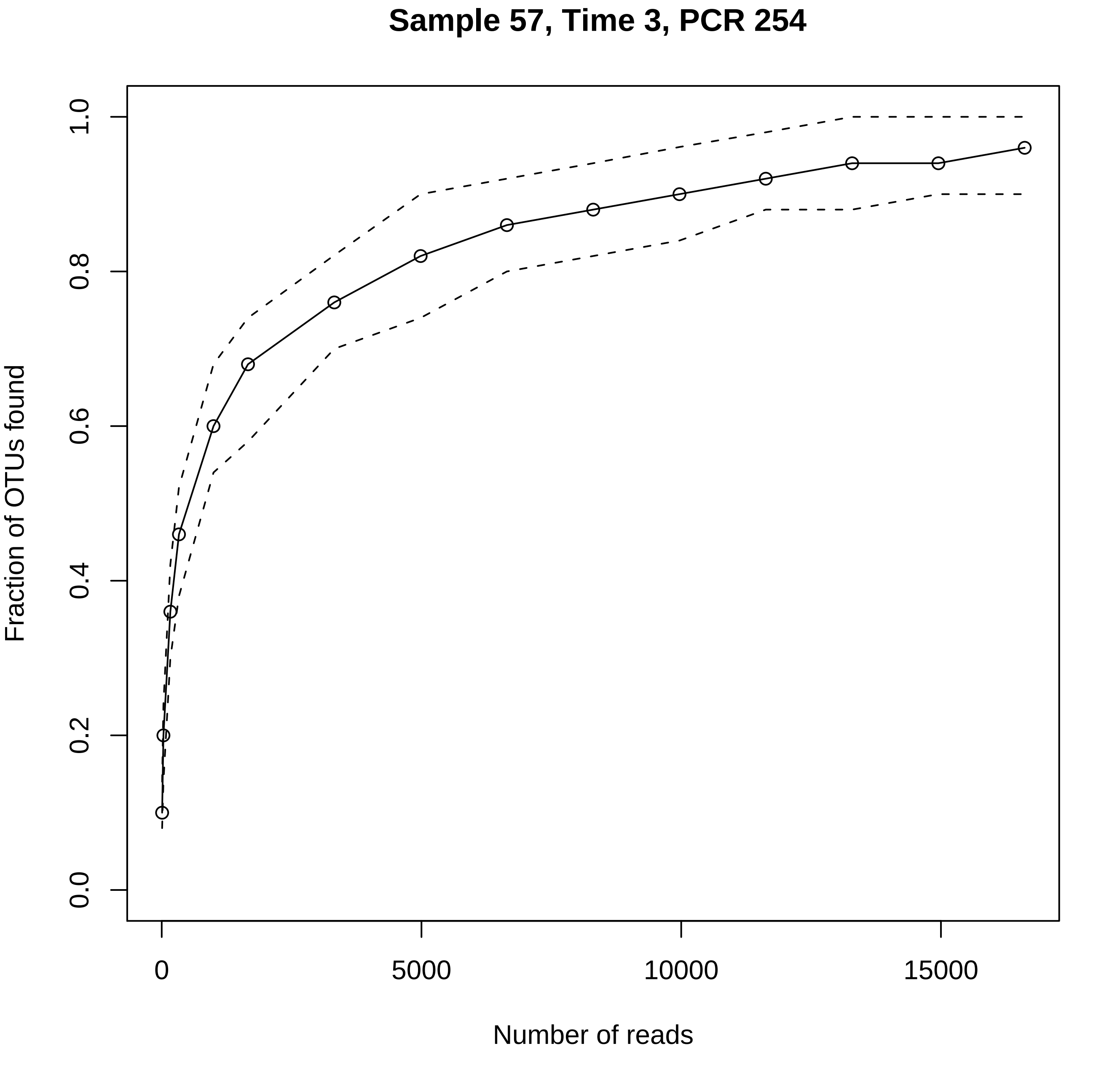
Sample 57, Time 3, PCR 254
Fraction of OTUs found
Number of reads
0.0
0.2
0.4
0.6
0.8
1.0
0
5000
10000
15000

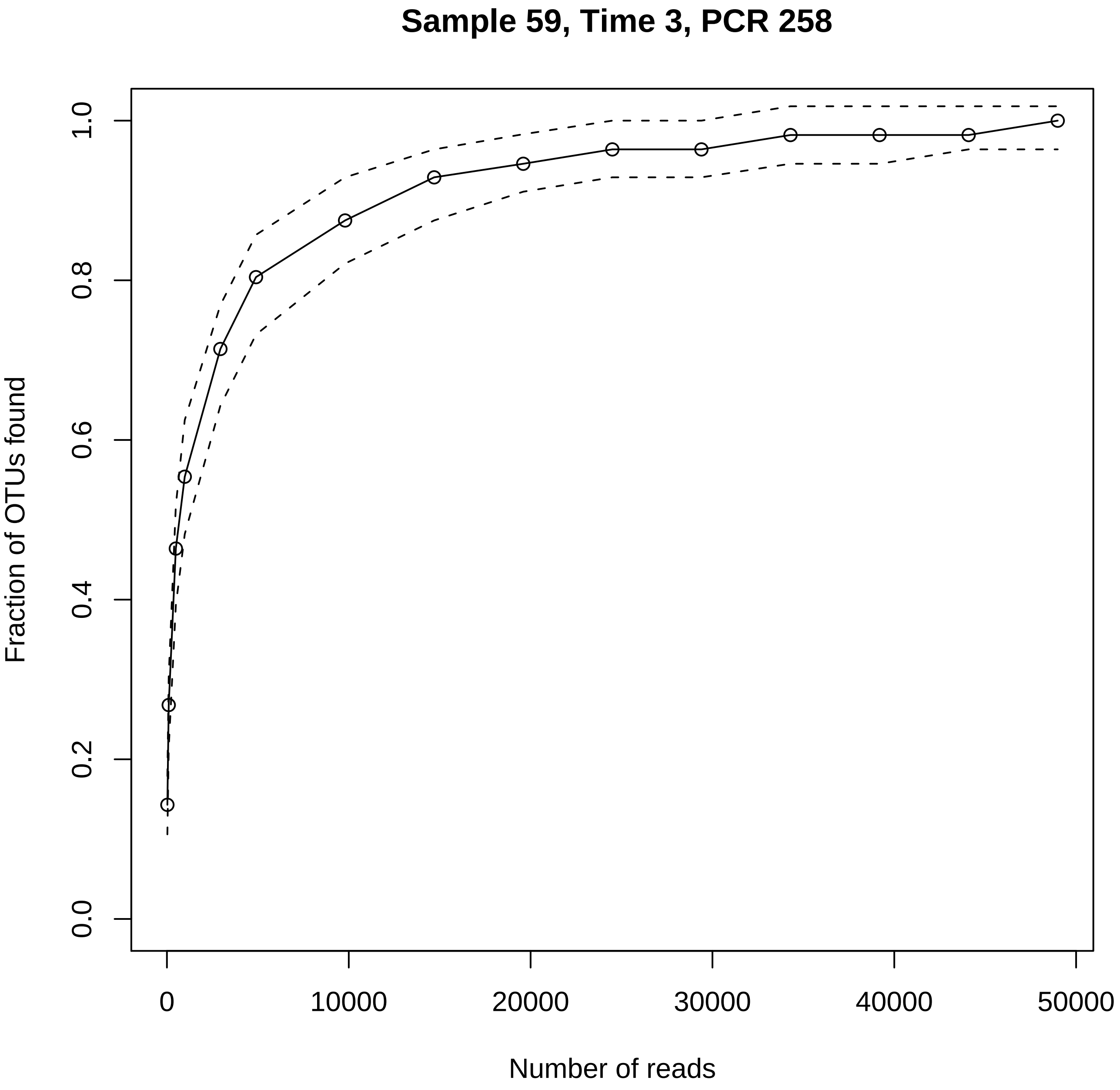

Sample 59, Time 3, PCR 258
Fraction of OTUs found
0.0
0.2
0.4
0.6
0.8
1.0
0
10000
20000
30000
40000
50000
Number of reads

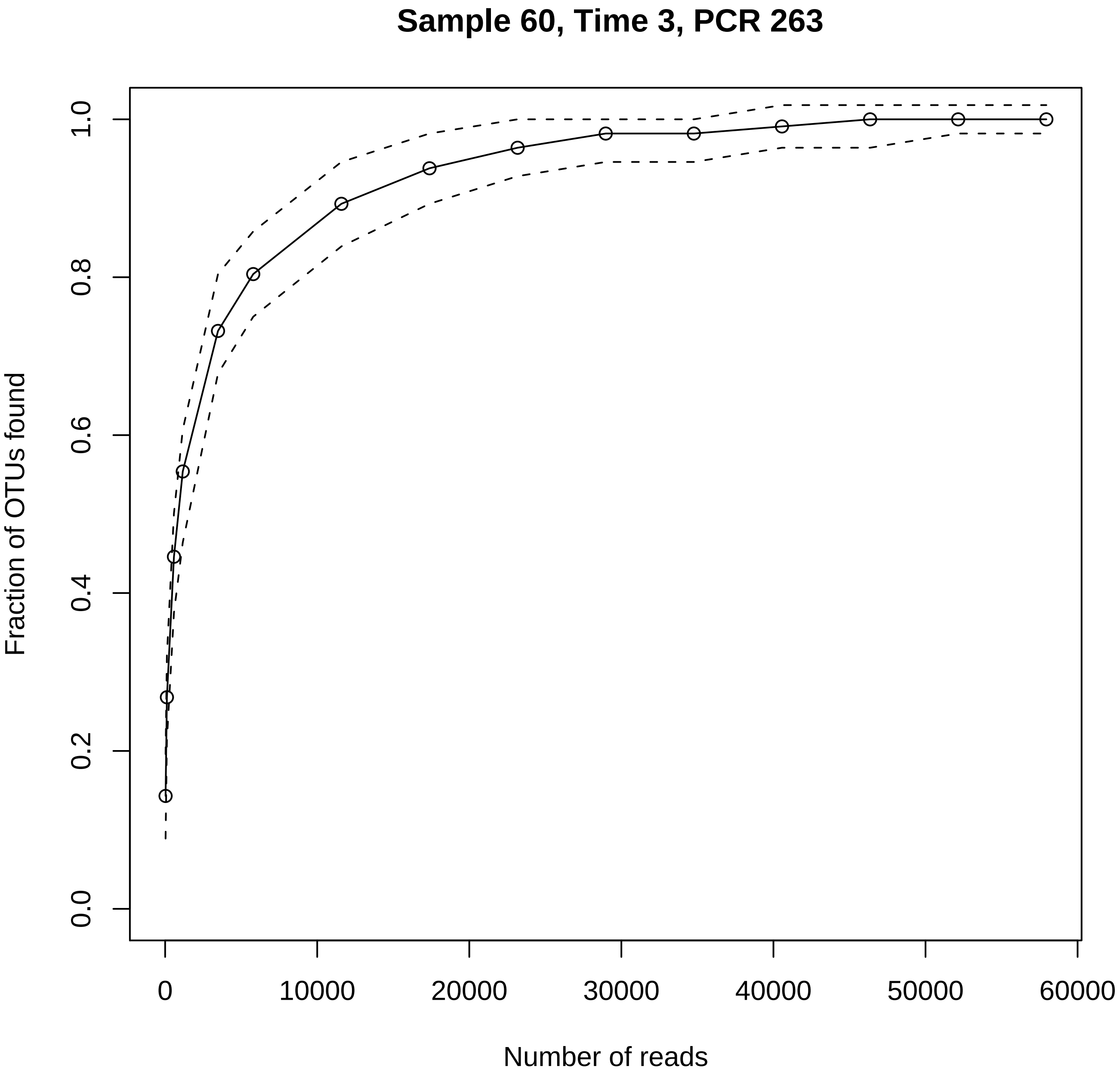
Sample 60, Time 3, PCR 263
Fraction of OTUs found
Number of reads
0.0
0.2
0.4
0.6
0.8
1.0
0
10000
20000
30000
40000
50000
60000

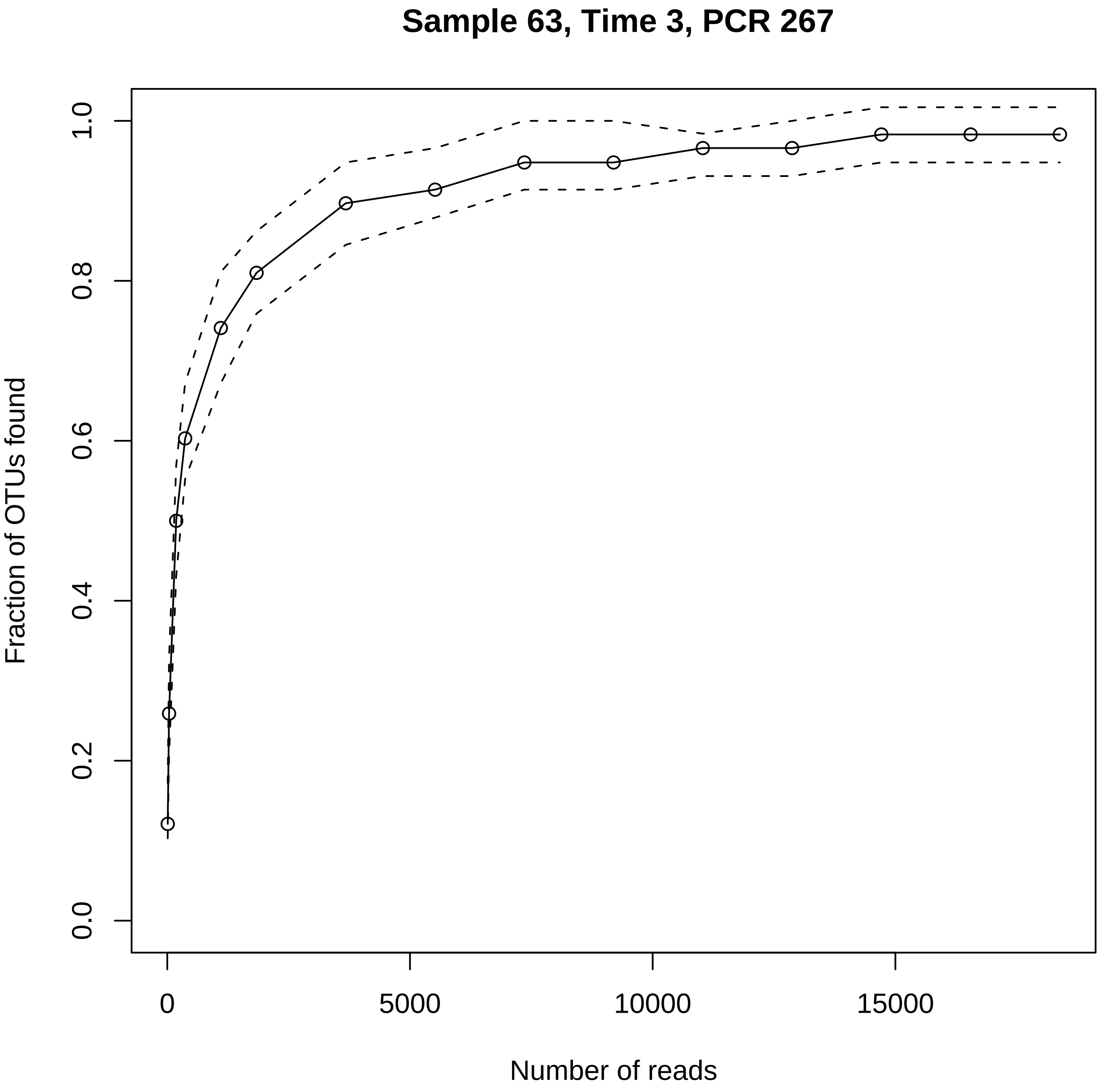

Sample 63, Time 3, PCR 267
Fraction of OTUs found
Number of reads
0.0
0.2
0.4
0.6
0.8
1.0
0
5000
10000
15000

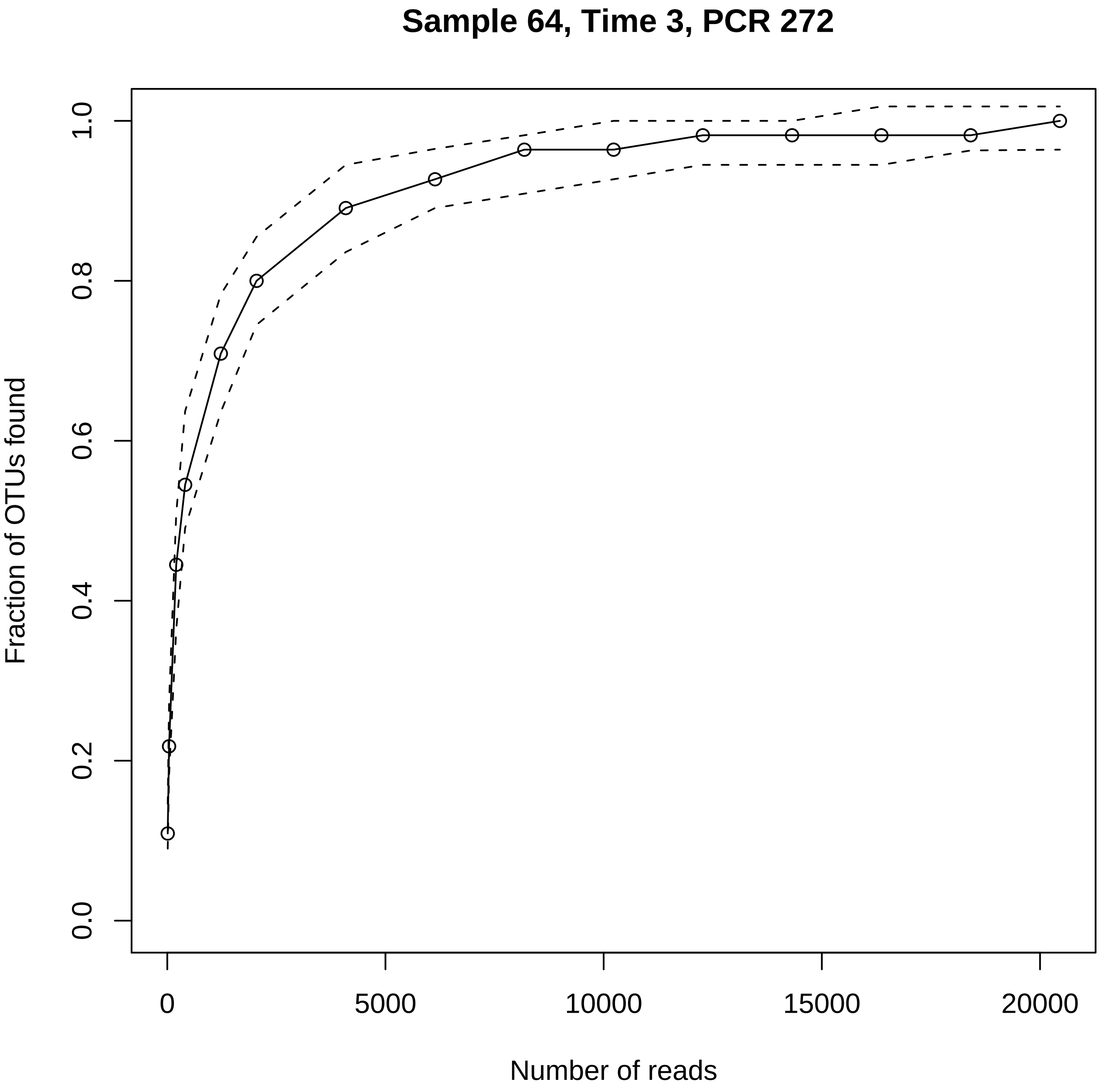

Sample 64, Time 3, PCR 272
Fraction of OTUs found
0.0
0.2
0.4
0.6
0.8
1.0
0
5000
10000
15000
20000
Number of reads

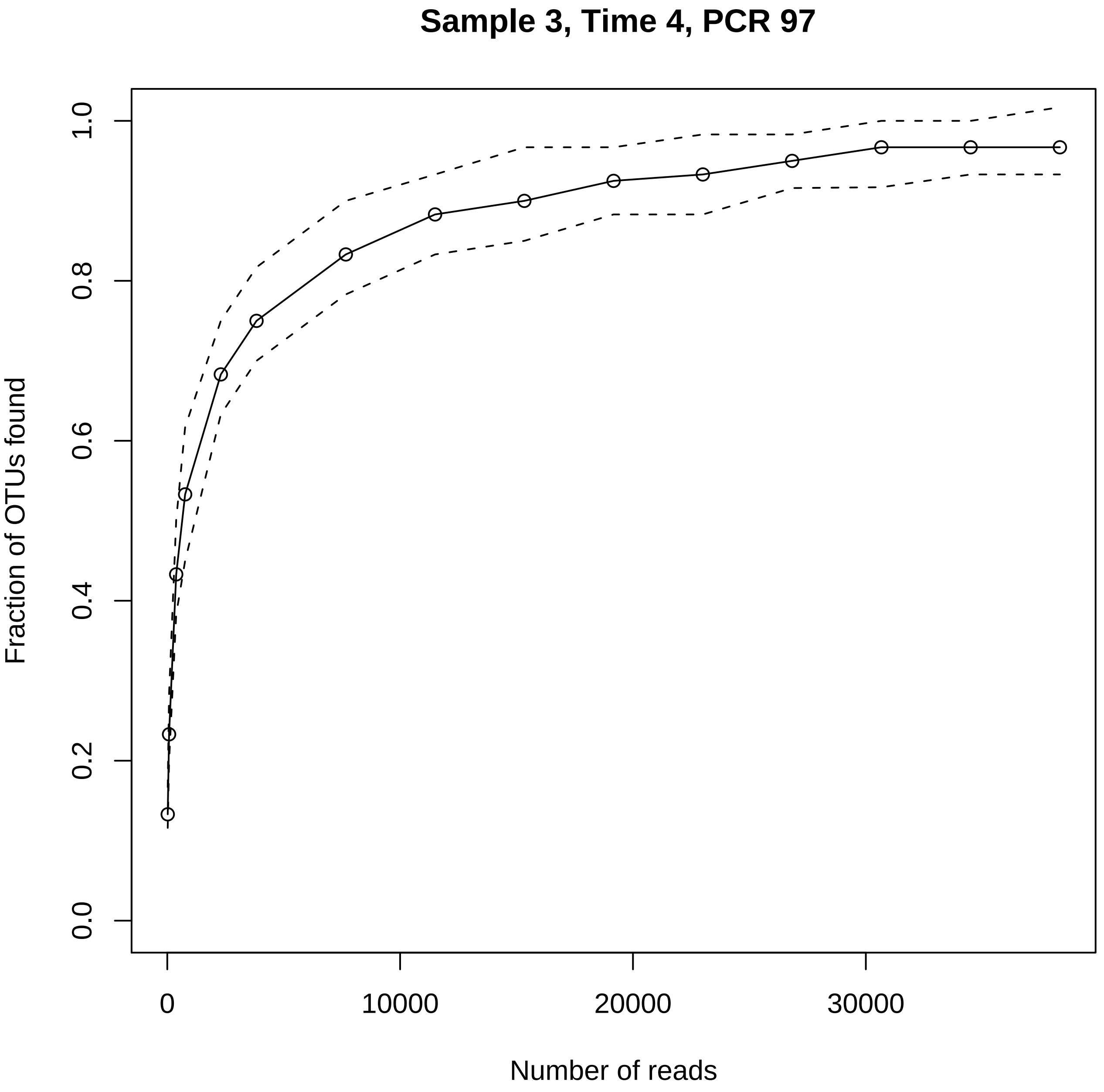
Sample 3, Time 4, PCR 97
Fraction of OTUs found
0.0
0.2
0.4
0.6
0.8
1.0
0
10000
20000
30000
Number of reads

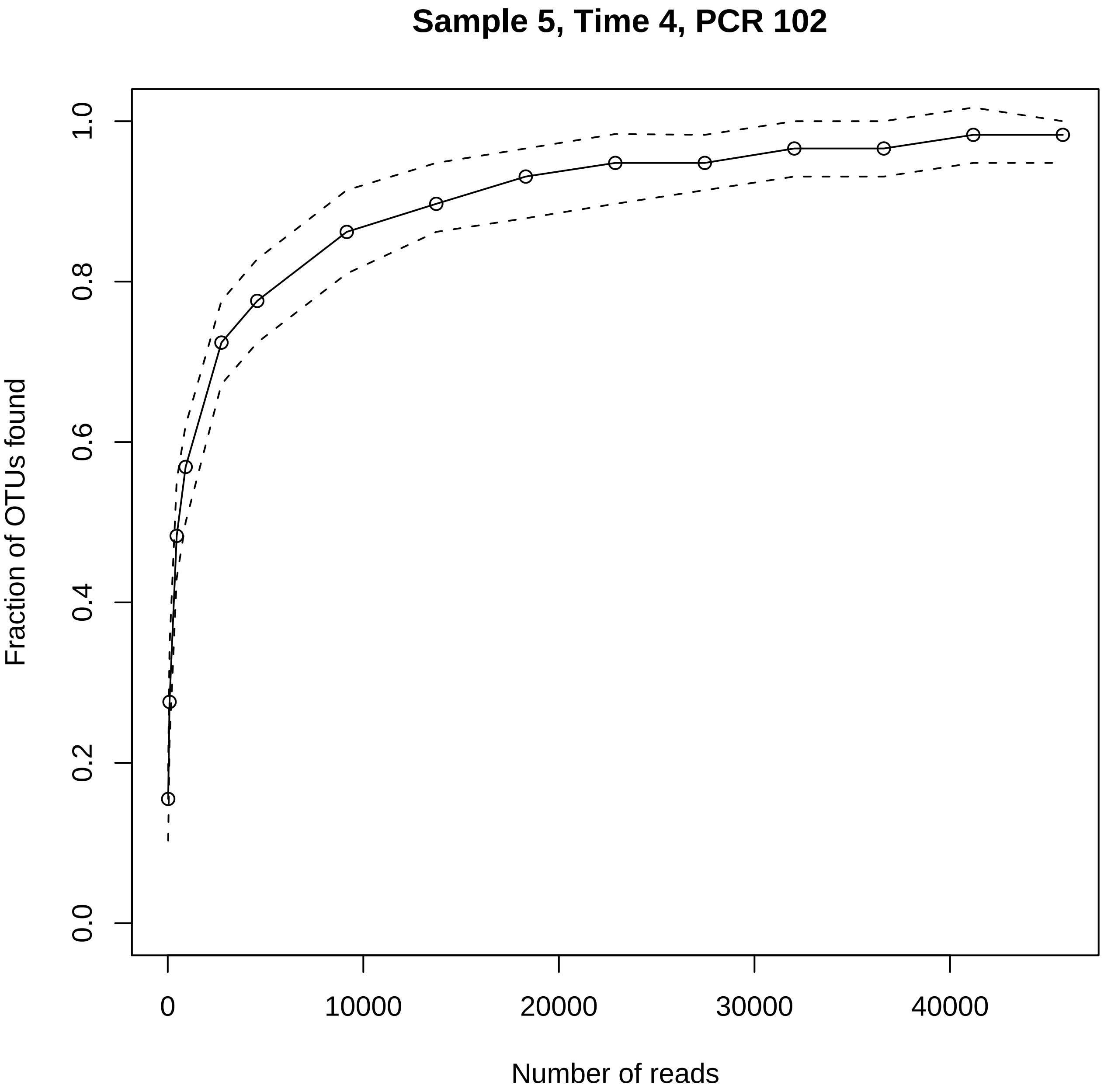
Sample 5, Time 4, PCR 102
Fraction of OTUs found
0.0
0.2
0.4
0.6
0.8
1.0
0
10000
20000
30000
40000
Number of reads

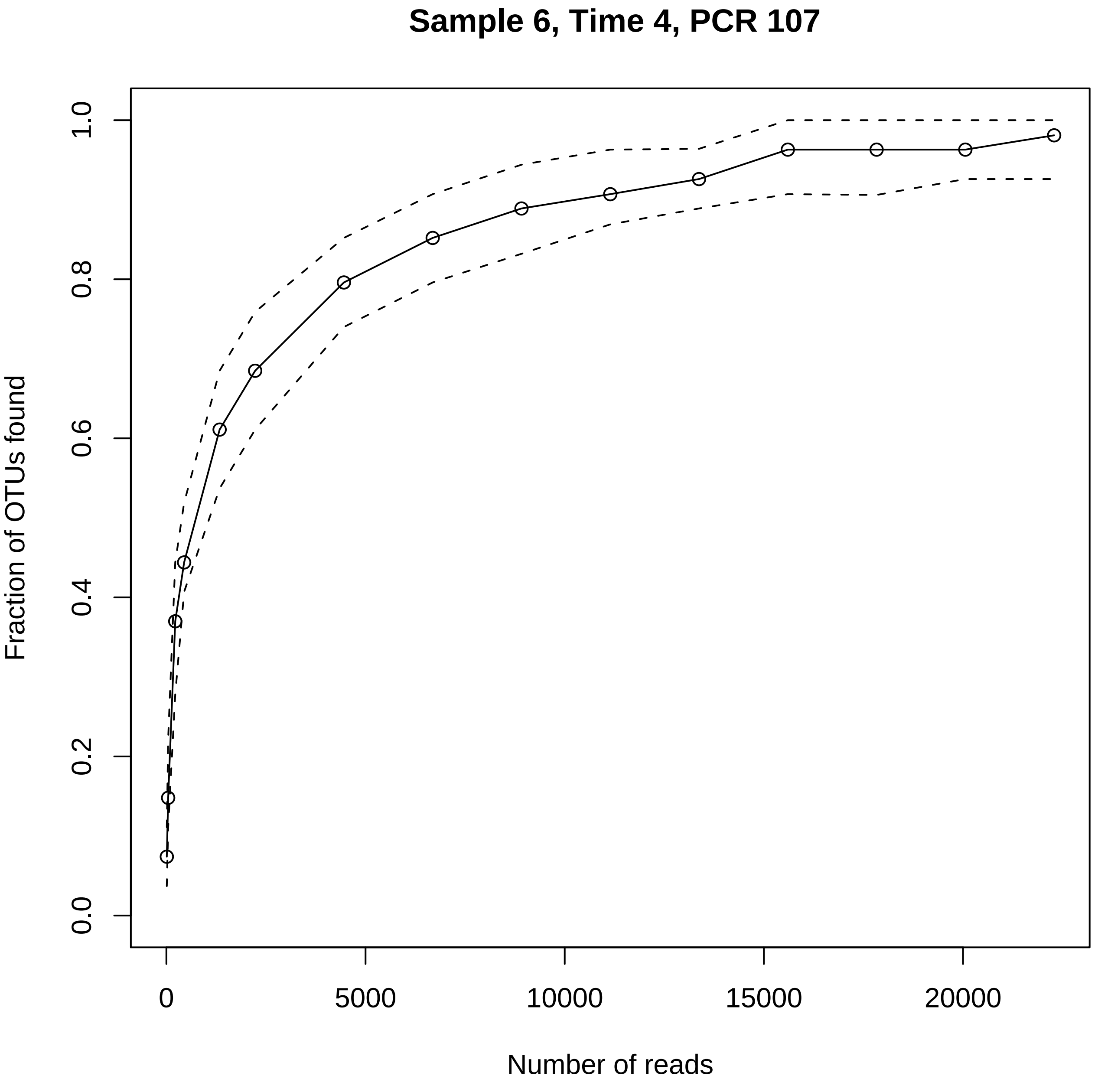

Sample 6, Time 4, PCR 107
Fraction of OTUs found
0.0
0.2
0.4
0.6
0.8
1.0
0
5000
10000
15000
20000
Number of reads

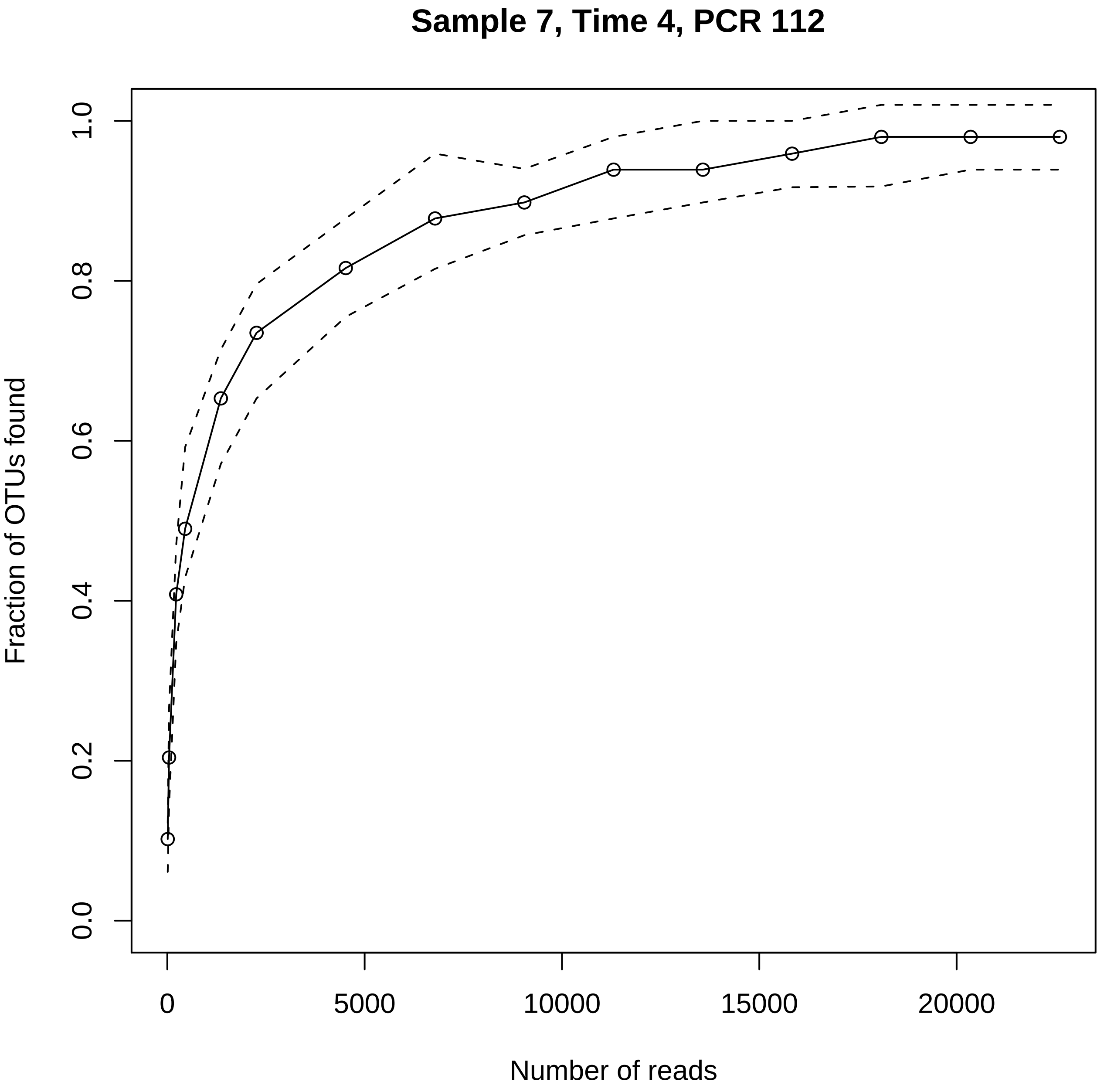
Sample 7, Time 4, PCR 112
Fraction of OTUs found
0.0
0.2
0.4
0.6
0.8
1.0
0
5000
10000
15000
20000
Number of reads

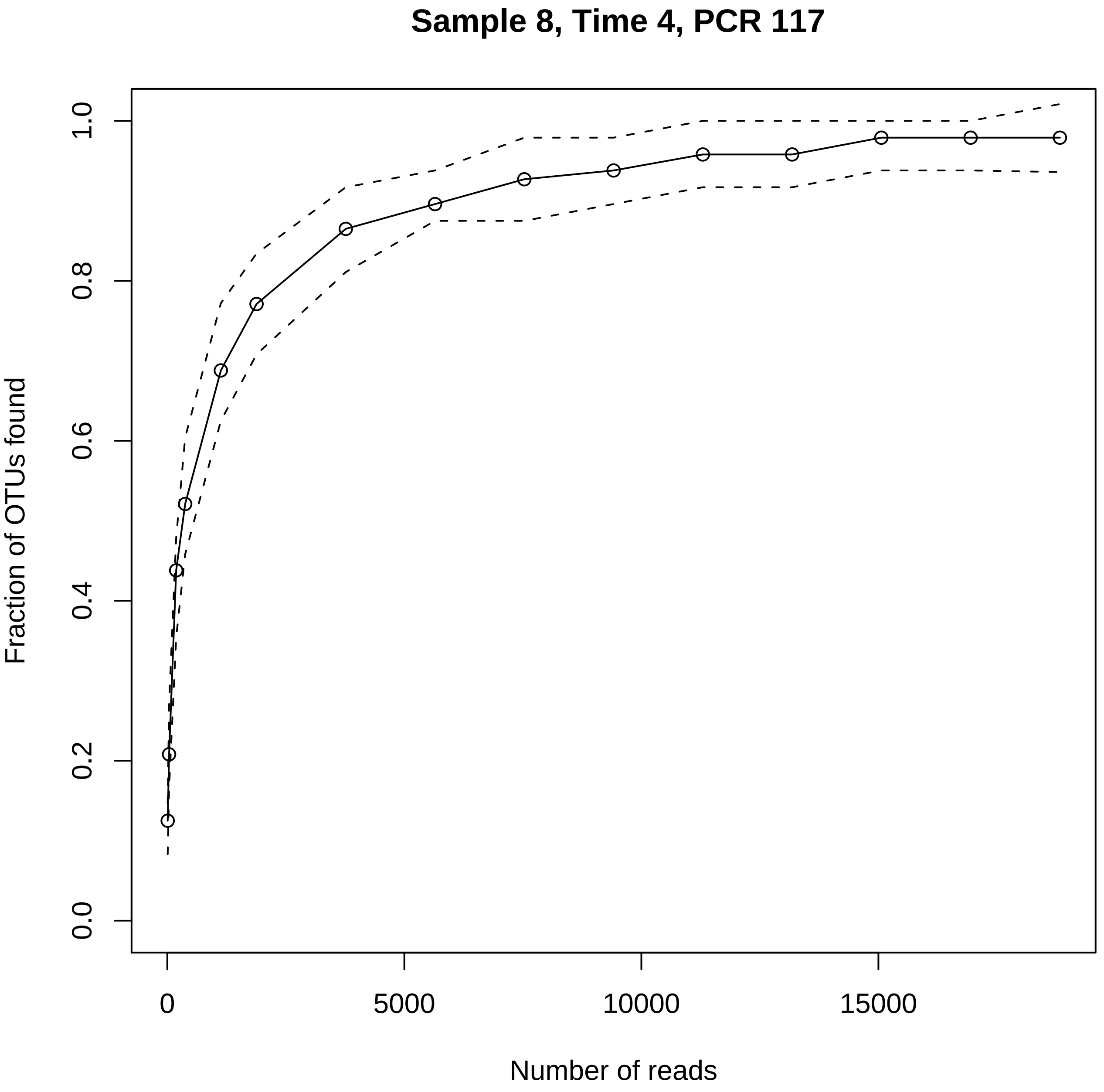

Sample 8, Time 4, PCR 117
Fraction of OTUs found
Number of reads
0.0
0.2
0.4
0.6
0.8
1.0
0
5000
10000
15000

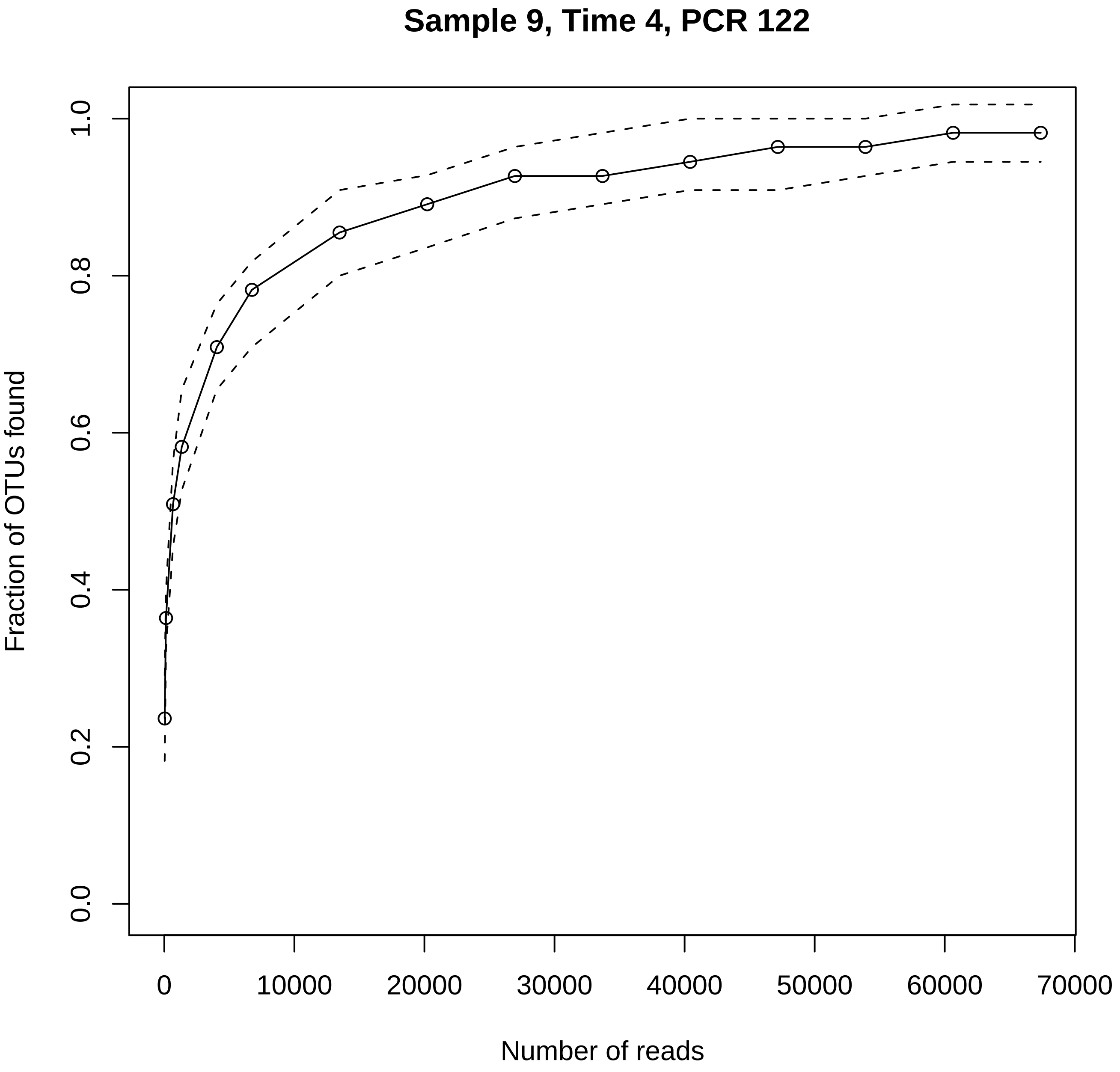

Sample 9, Time 4, PCR 122
Fraction of OTUs found
0.0
0.2
0.4
0.6
0.8
1.0
0
10000
20000
30000
40000
50000
60000
70000
Number of reads

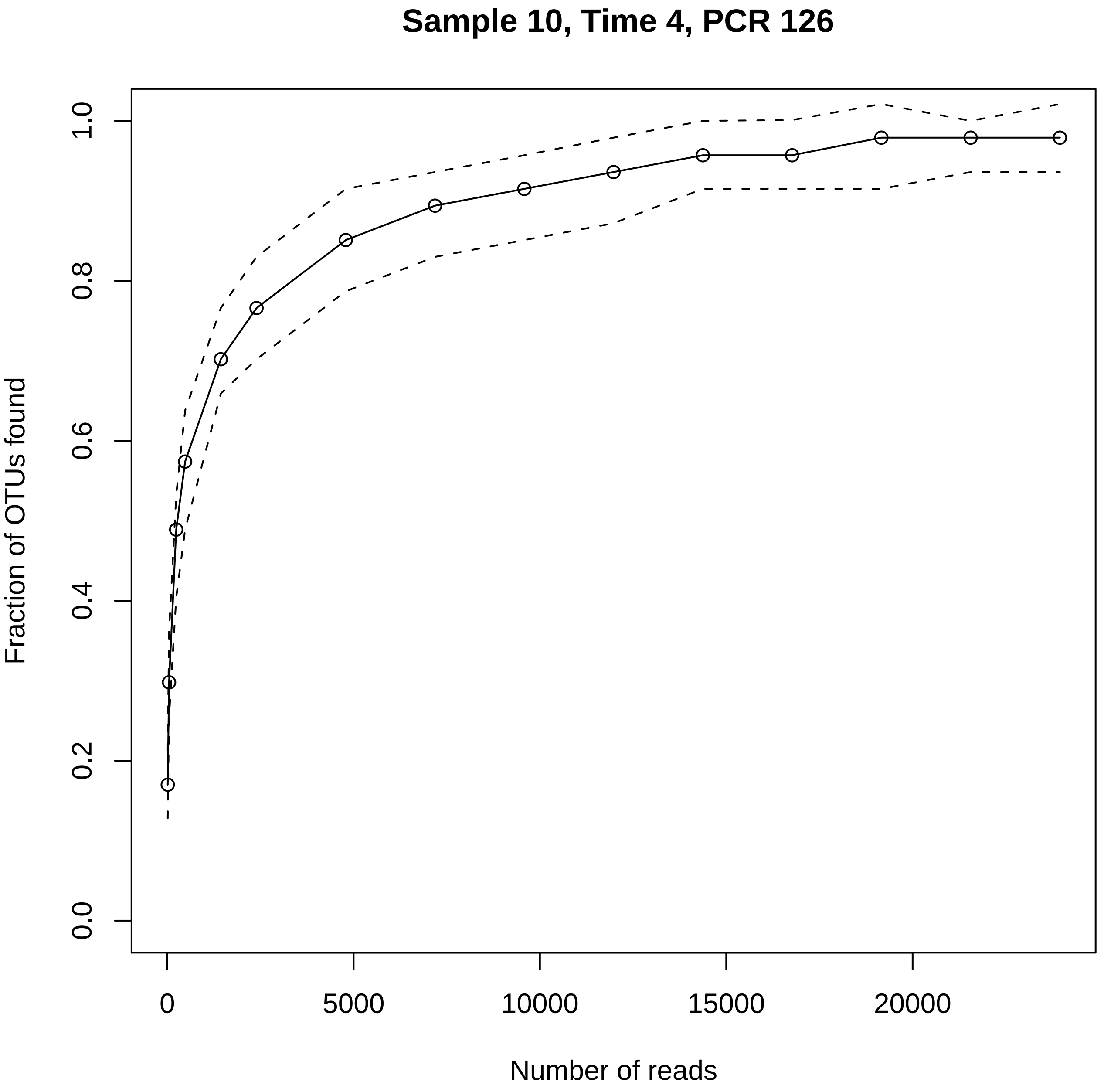

Sample 10, Time 4, PCR 126
Fraction of OTUs found
Number of reads
0.0
0.2
0.4
0.6
0.8
1.0
0
5000
10000
15000
20000

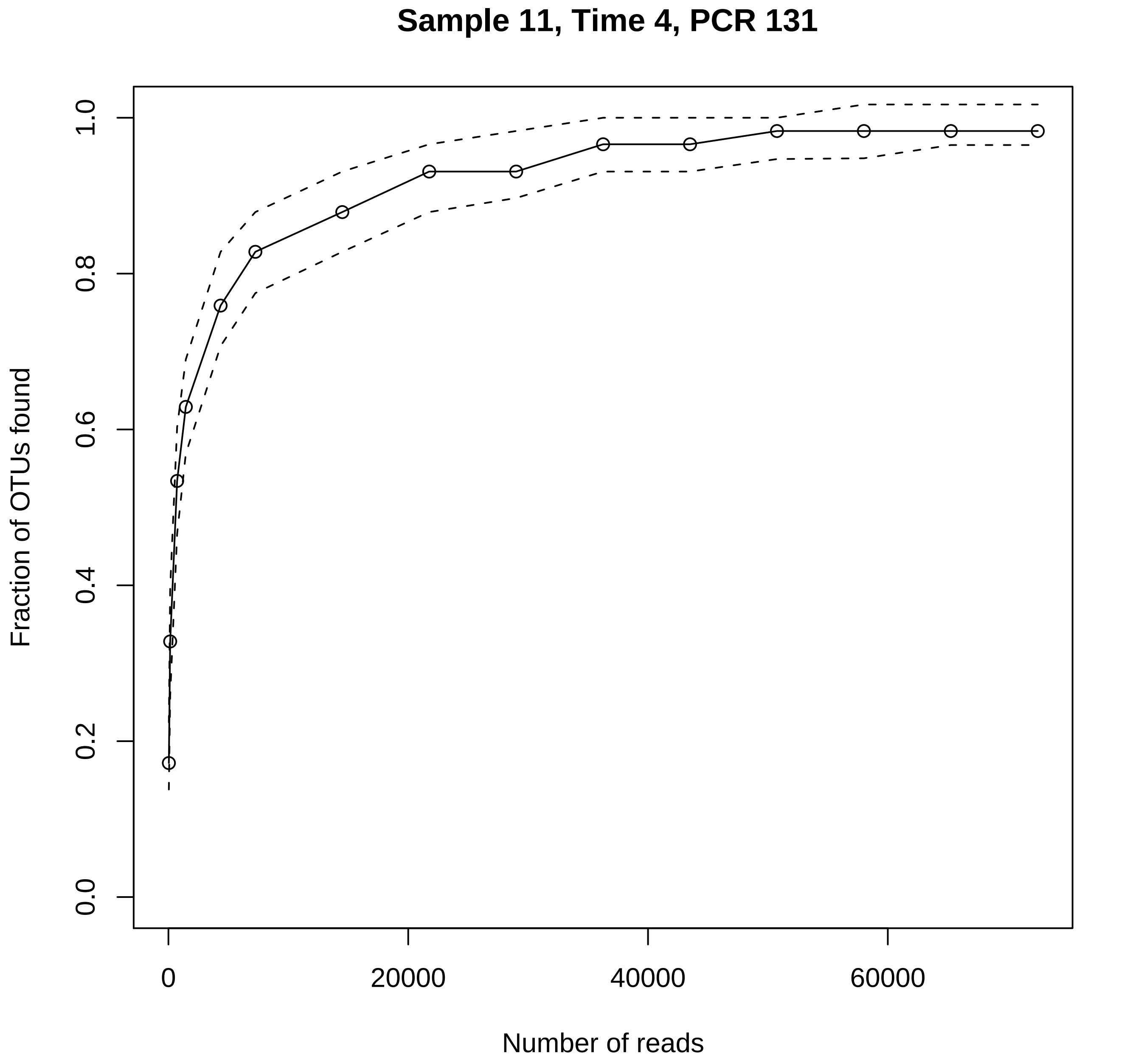
Sample 11, Time 4, PCR 131
Fraction of OTUs found
0.0
0.2
0.4
0.6
0.8
1.0
0
20000
40000
60000
Number of reads

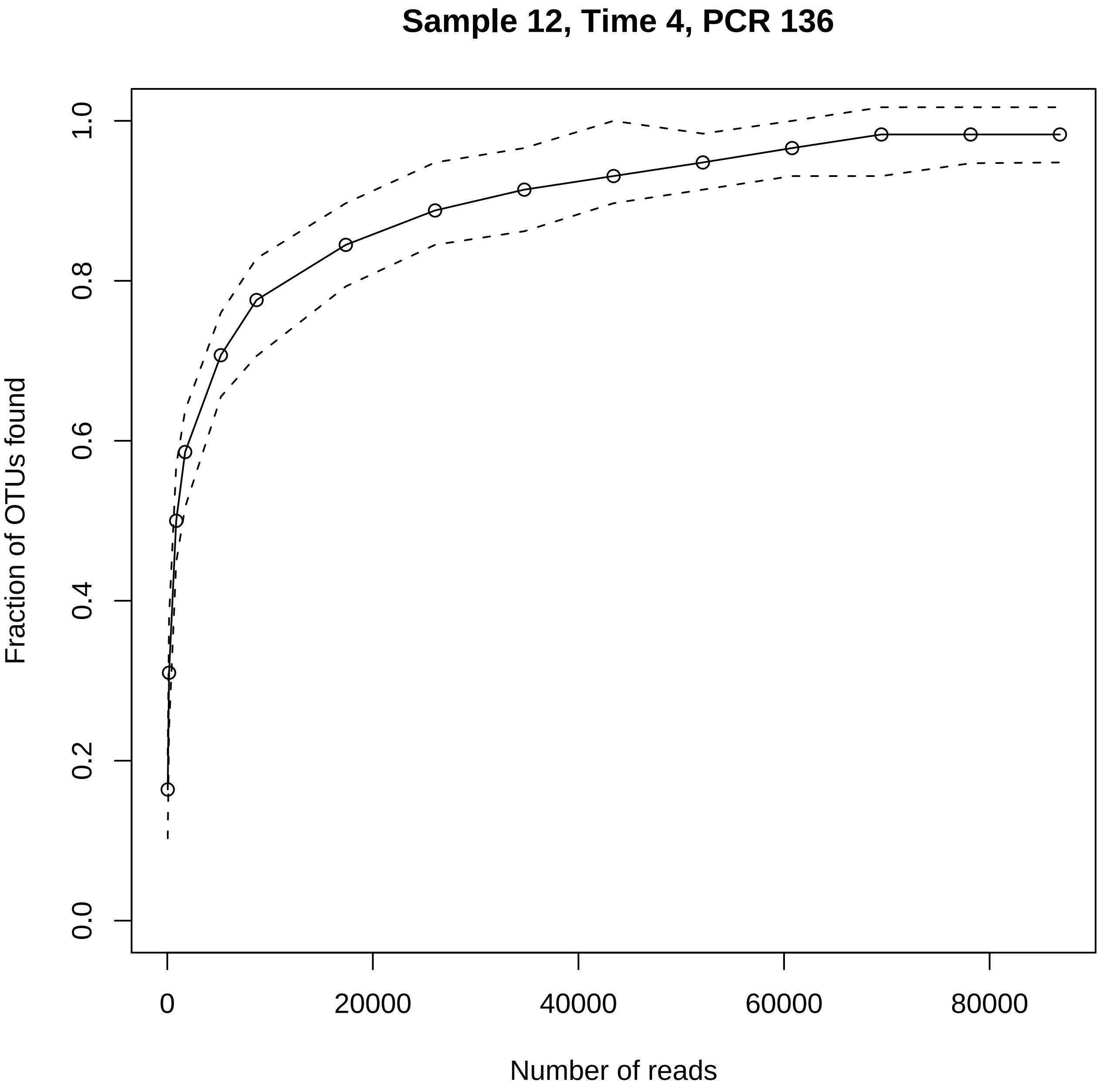
Sample 12, Time 4, PCR 136
Fraction of OTUs found
0.0
0.2
0.4
0.6
0.8
1.0
0
20000
40000
60000
80000
Number of reads

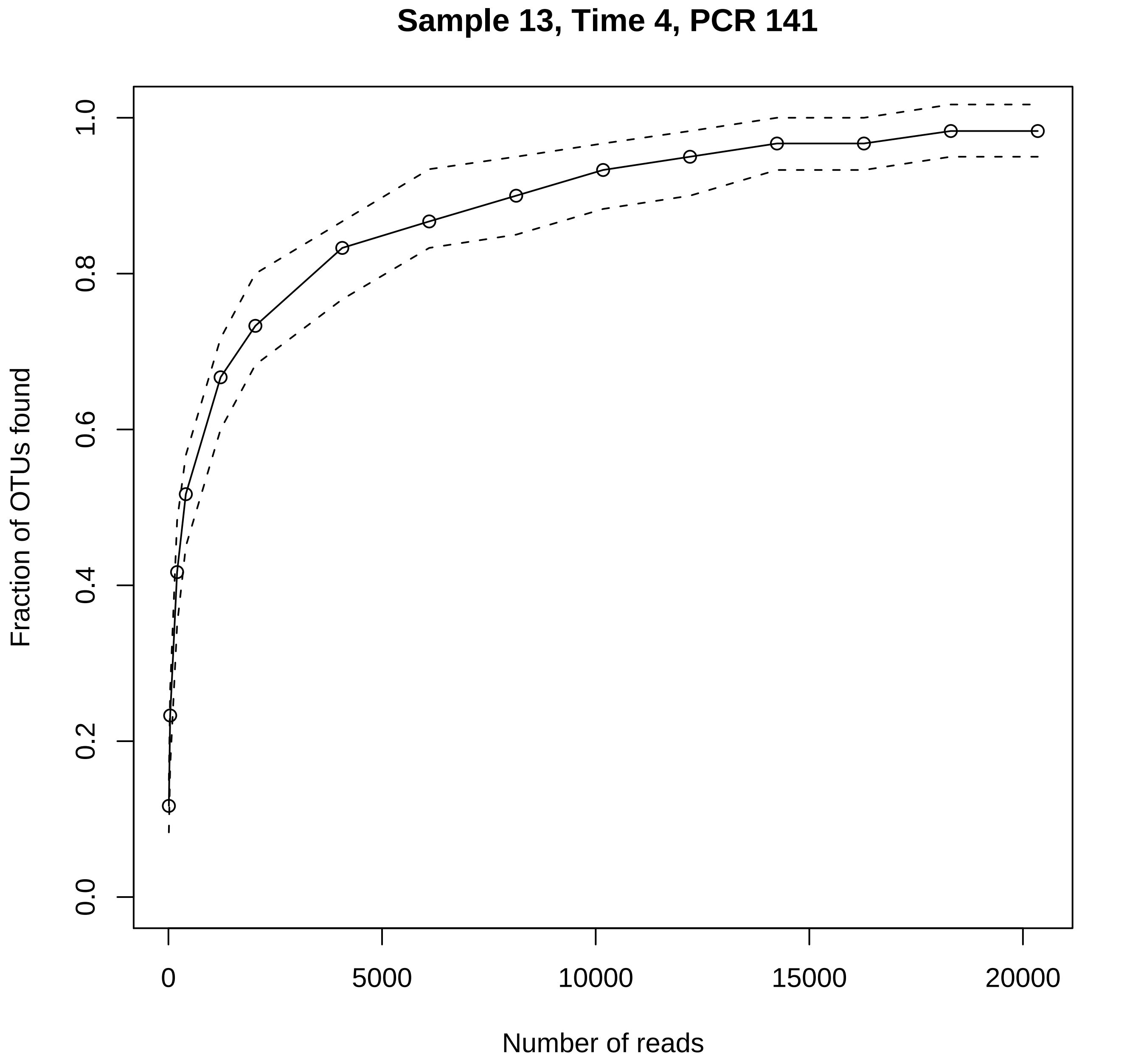

Sample 13, Time 4, PCR 141
Fraction of OTUs found
0.0
0.2
0.4
0.6
0.8
1.0
0
5000
10000
15000
20000
Number of reads

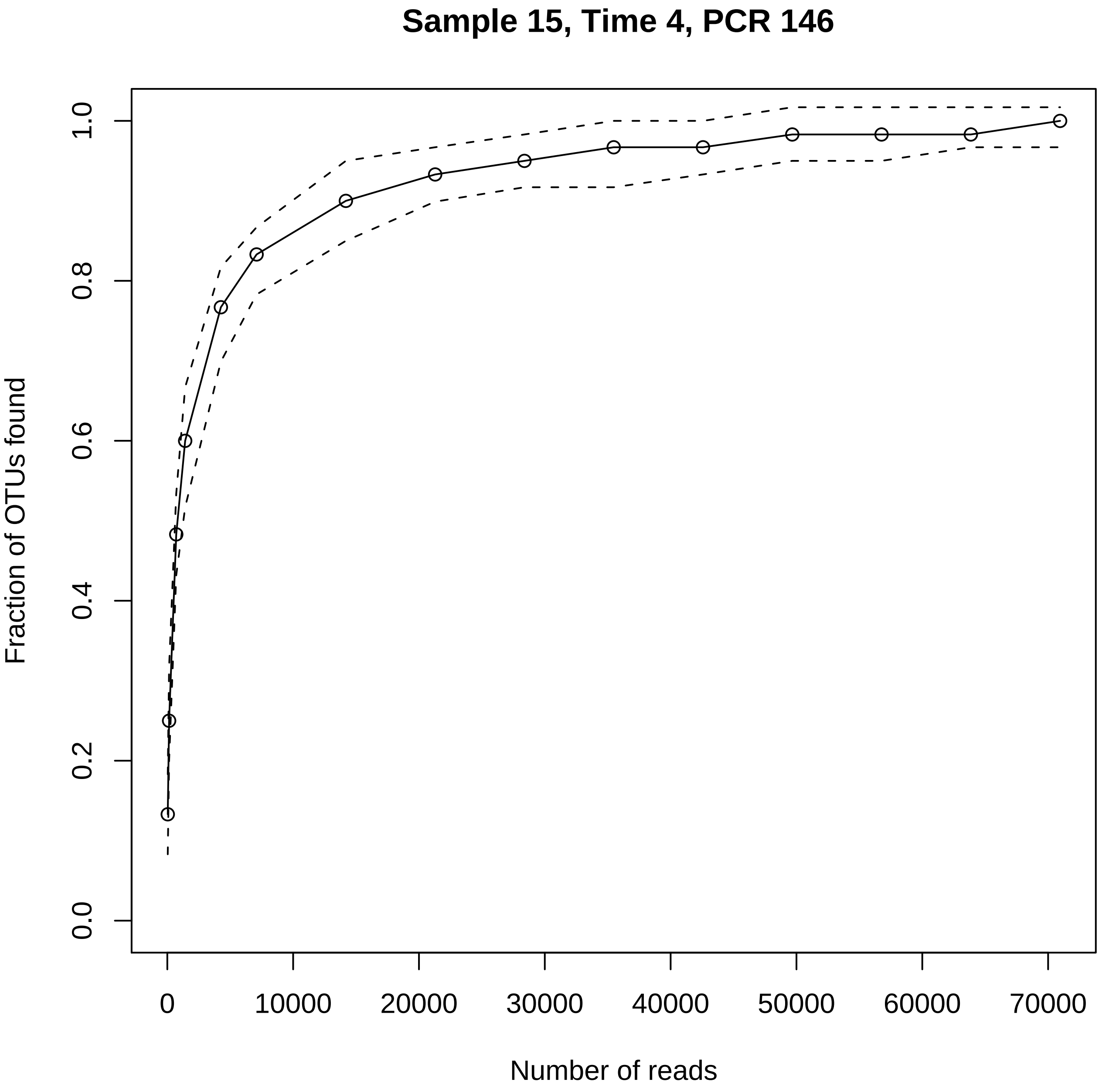

Sample 15, Time 4, PCR 146
Fraction of OTUs found
0.0
0.2
0.4
0.6
0.8
1.0
0
10000
20000
30000
40000
50000
60000
70000
Number of reads

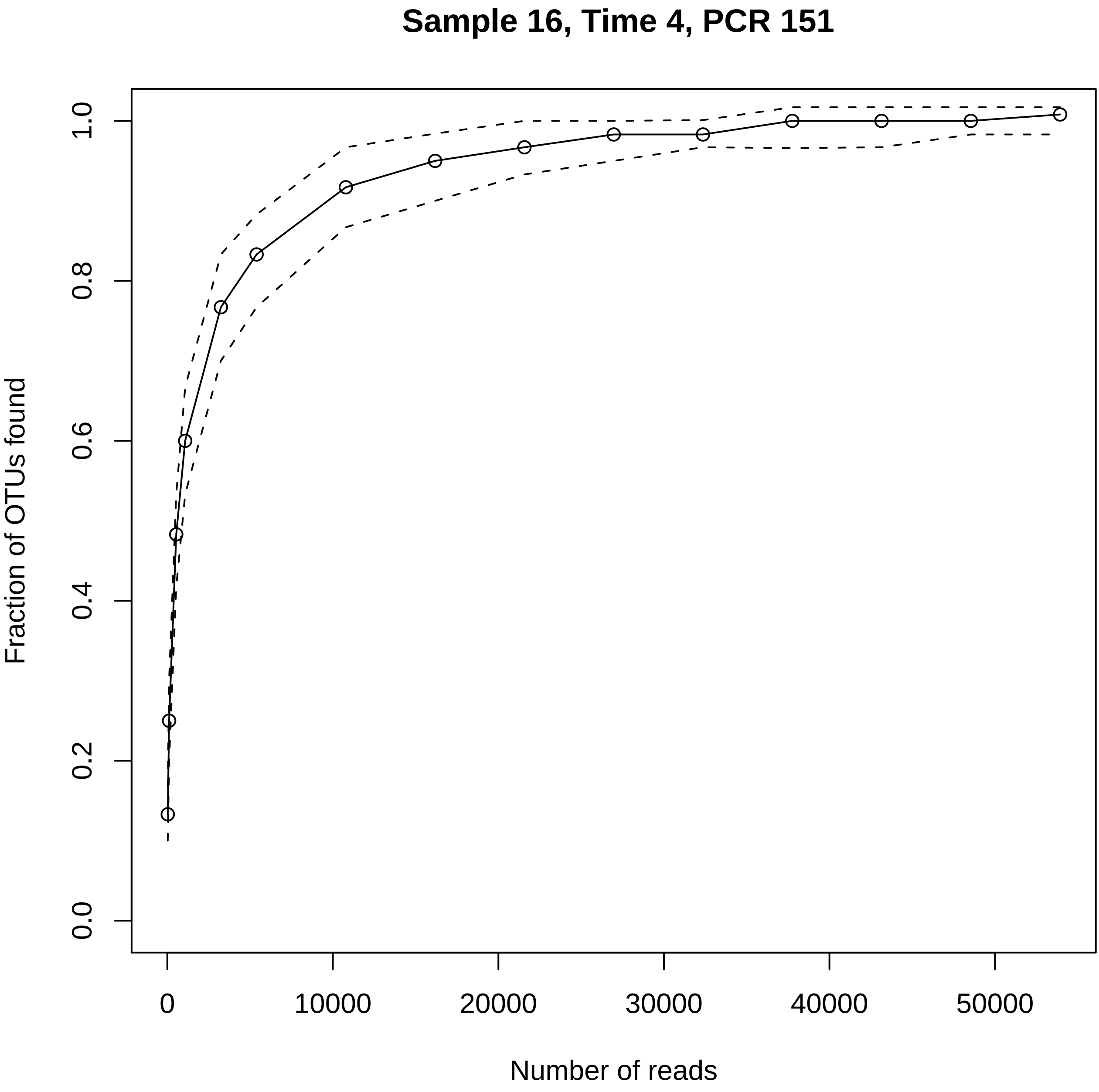
Sample 16, Time 4, PCR 151
Fraction of OTUs found
0.0
0.2
0.4
0.6
0.8
1.0
0
10000
20000
30000
40000
50000
Number of reads

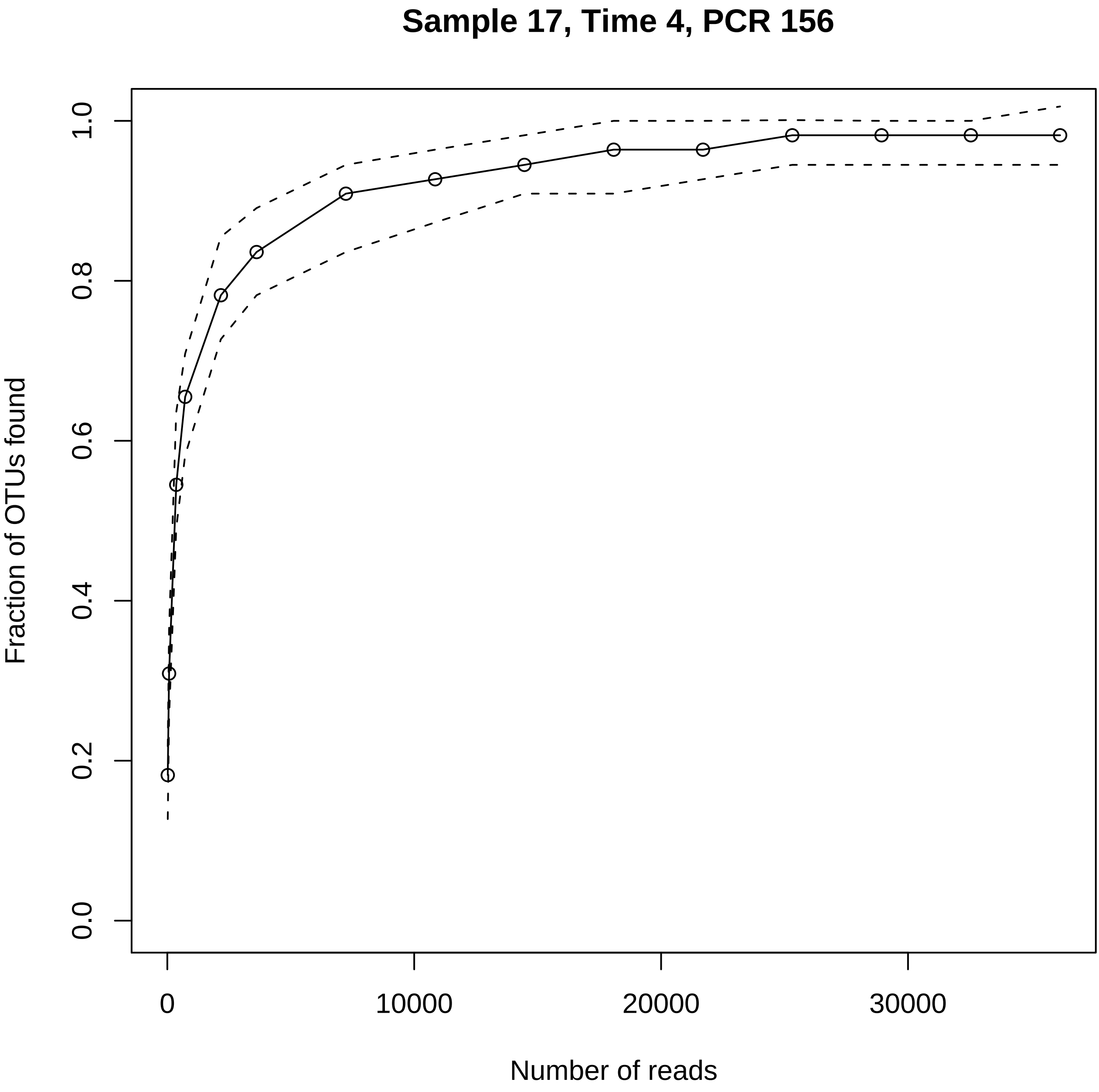

Sample 17, Time 4, PCR 156
Fraction of OTUs found
0.0
0.2
0.4
0.6
0.8
1.0
0
10000
20000
30000
Number of reads

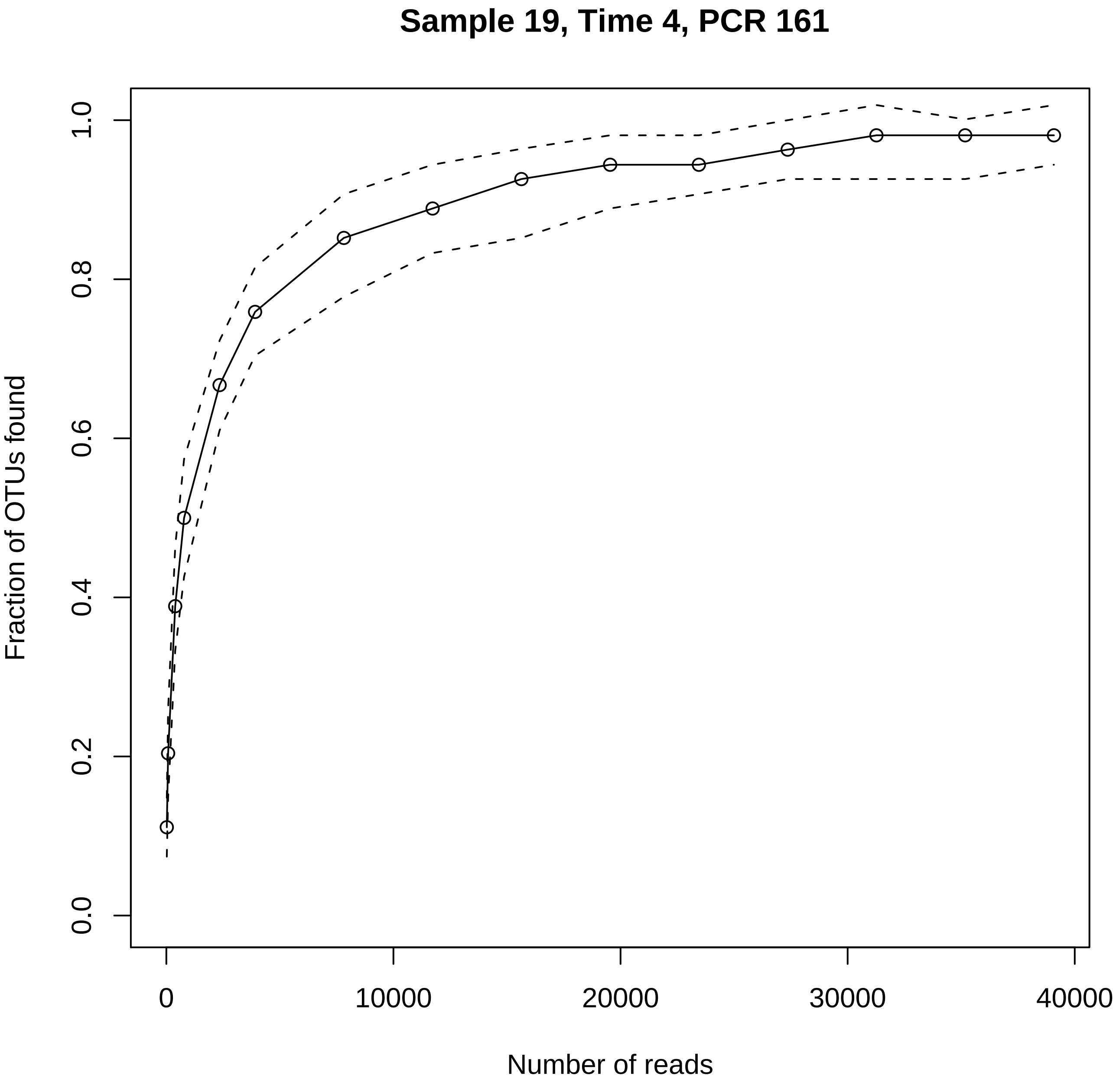
Sample 19, Time 4, PCR 161
Fraction of OTUs found
0.0
0.2
0.4
0.6
0.8
1.0
0
10000
20000
30000
40000
Number of reads

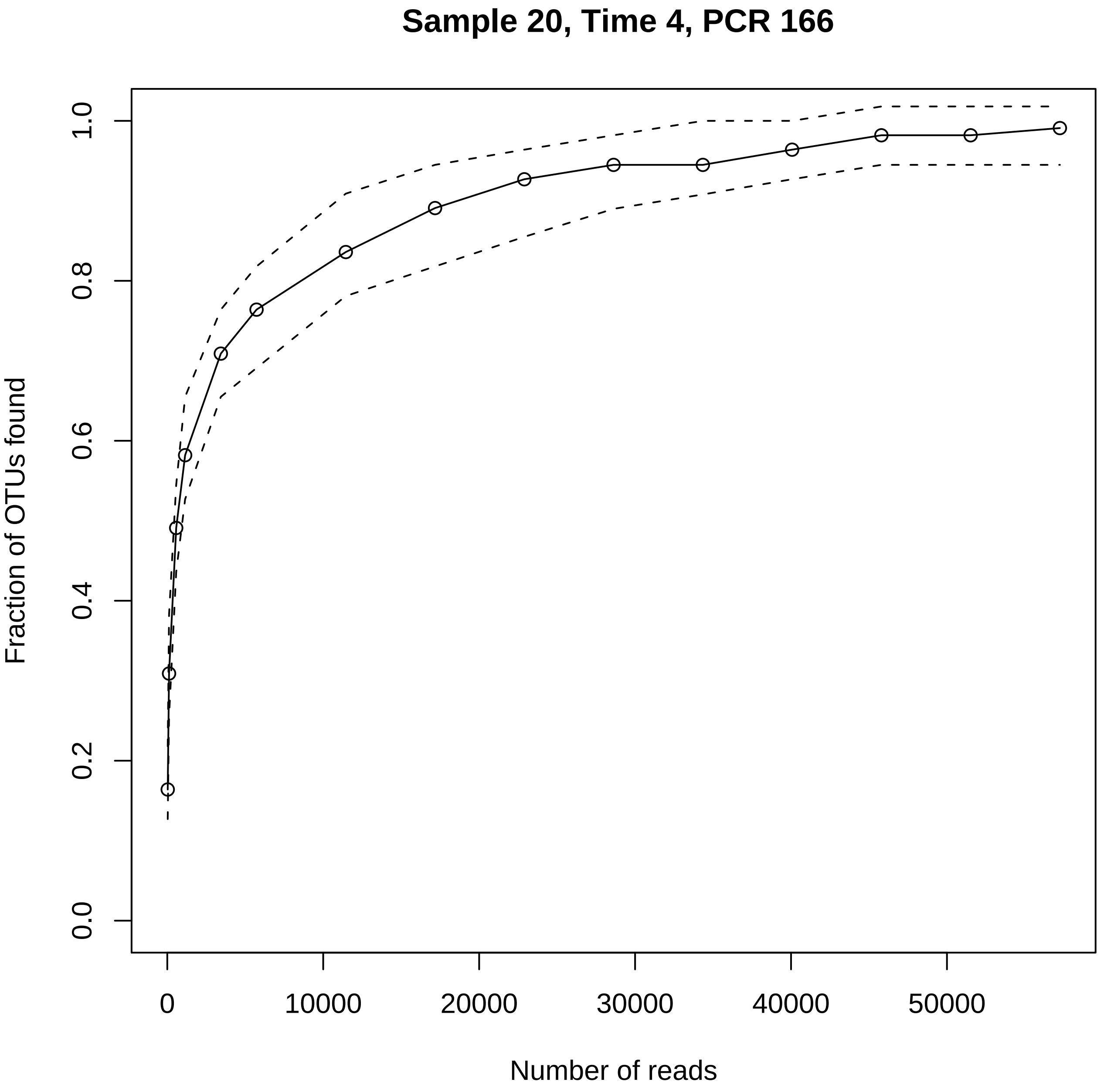

Sample 20, Time 4, PCR 166
Fraction of OTUs found
0.0
0.2
0.4
0.6
0.8
1.0
0
10000
20000
30000
40000
50000
Number of reads

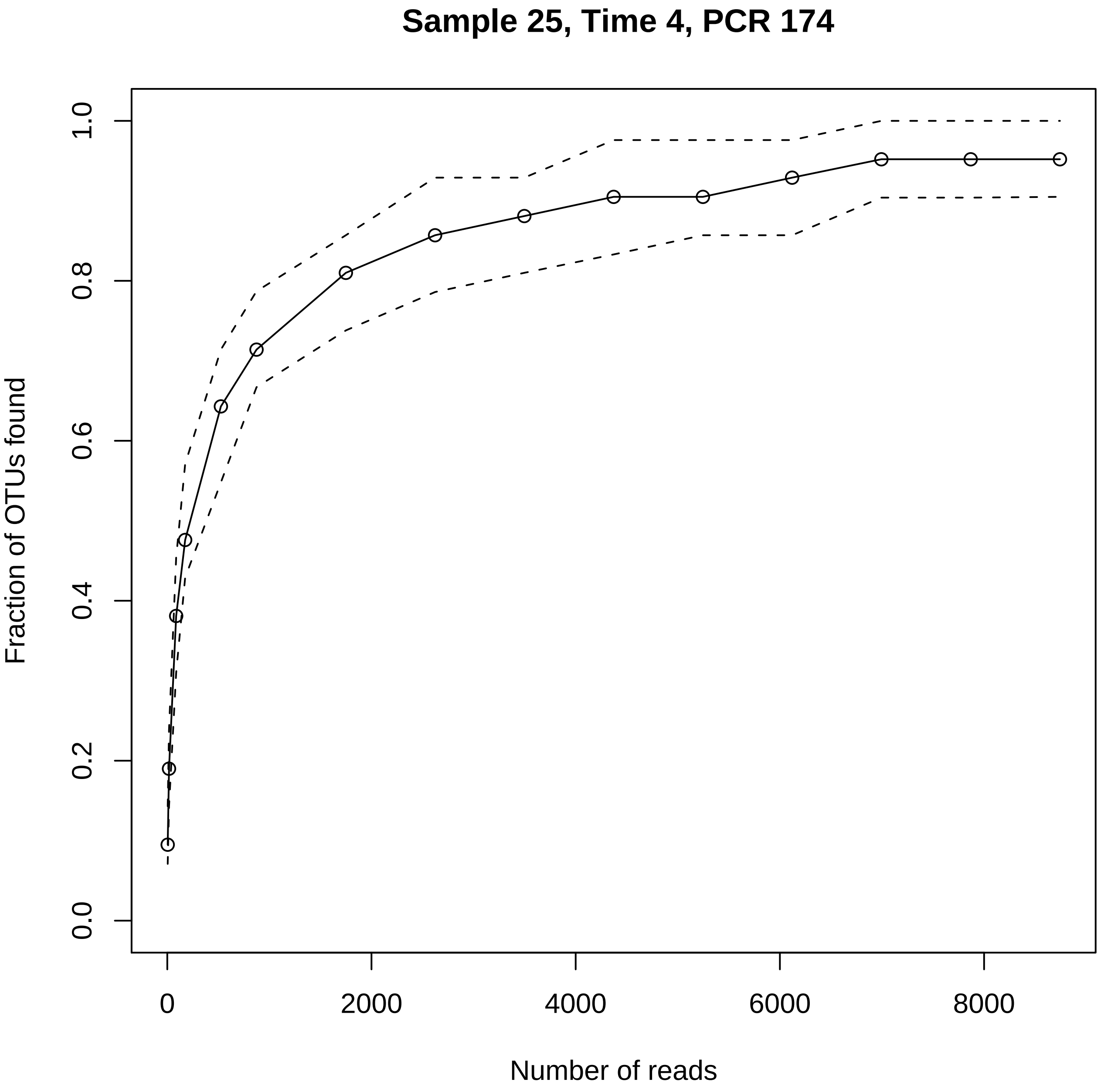

Sample 25, Time 4, PCR 174
Fraction of OTUs found
0.0
0.2
0.4
0.6
0.8
1.0
0
2000
4000
6000
8000
Number of reads

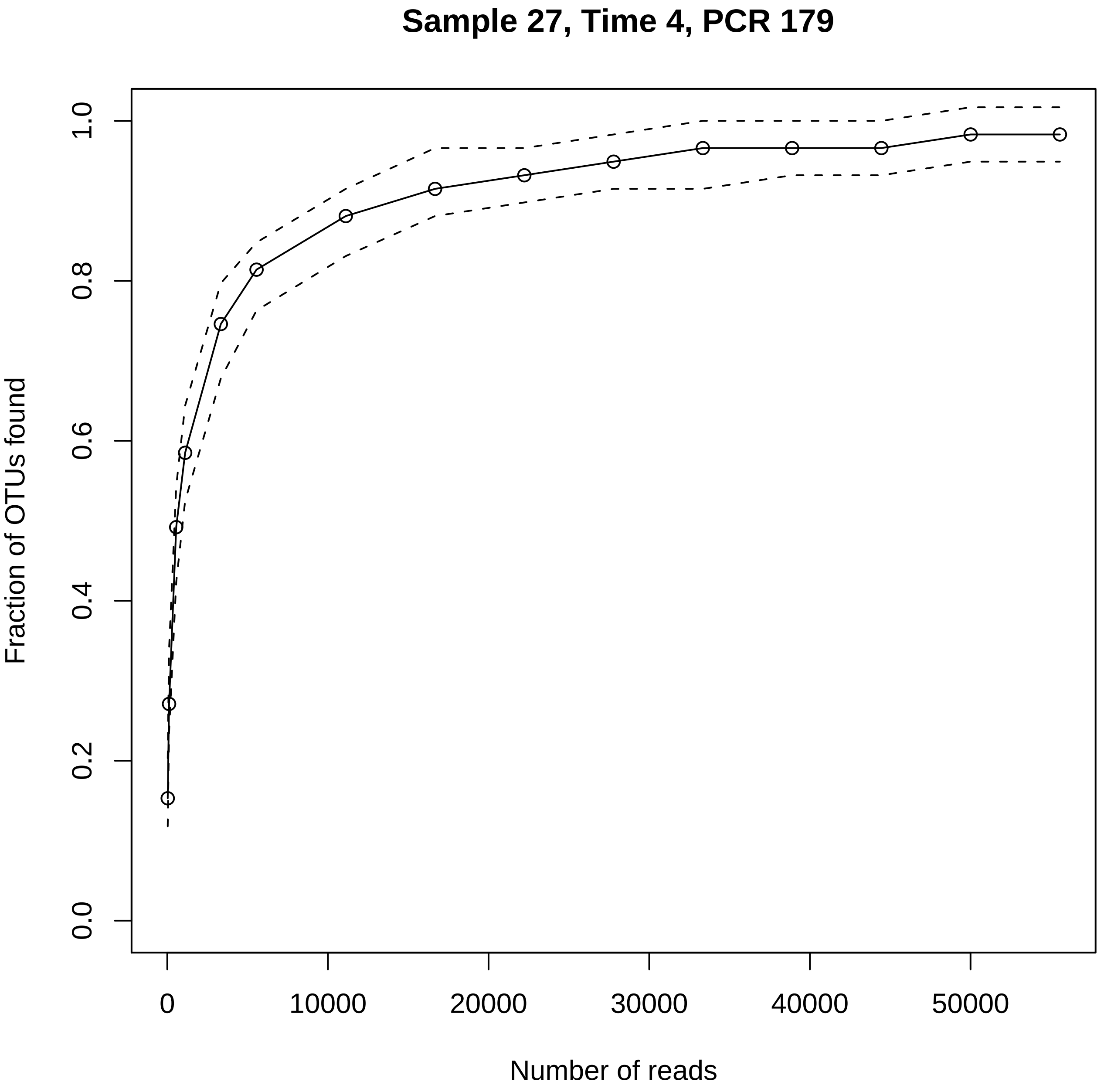

Sample 27, Time 4, PCR 179
Fraction of OTUs found
0.0
0.2
0.4
0.6
0.8
1.0
0
10000
20000
30000
40000
50000
Number of reads

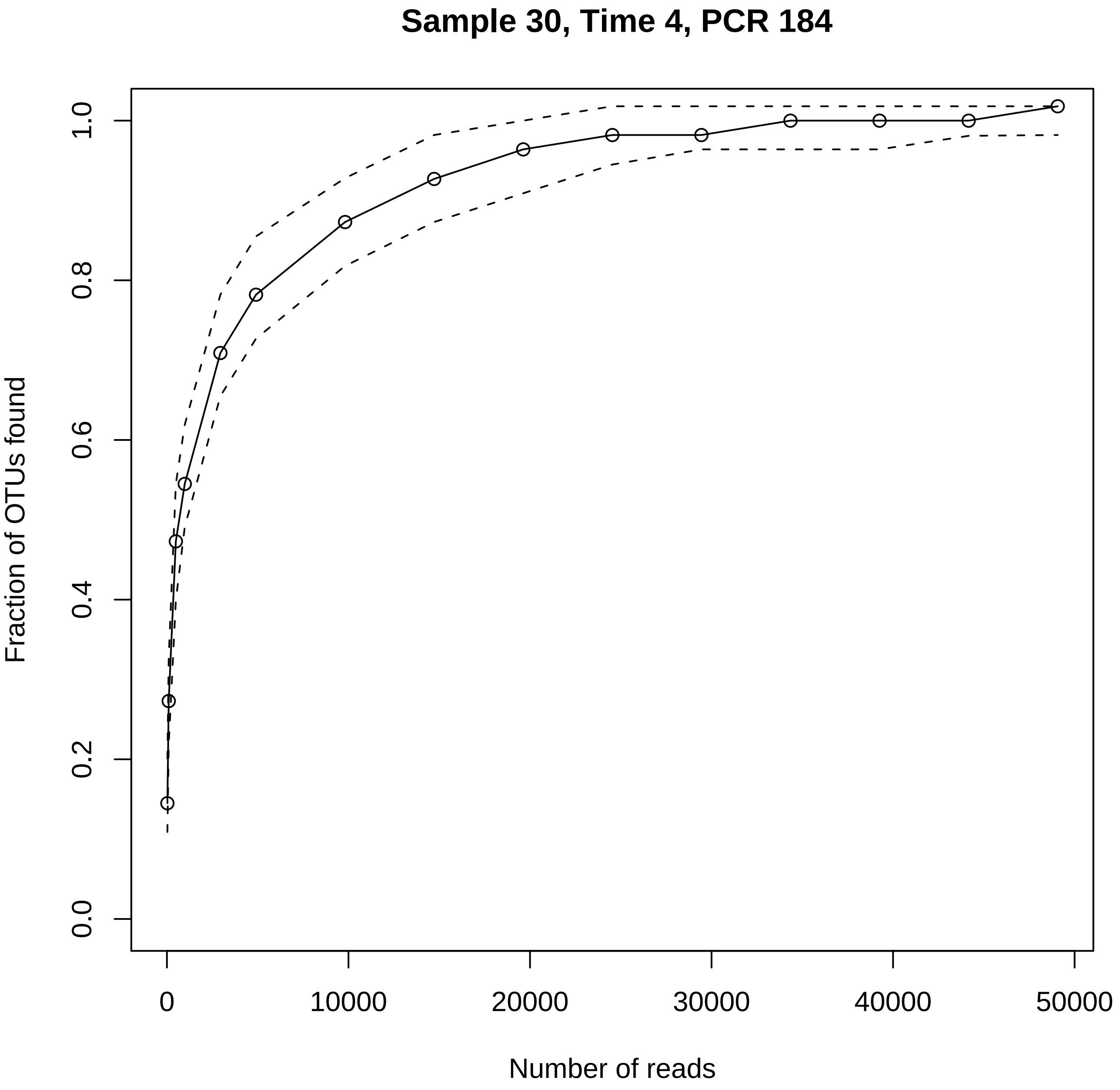
Sample 30, Time 4, PCR 184
Fraction of OTUs found
Number of reads
0.0
0.2
0.4
0.6
0.8
1.0
0
10000
20000
30000
40000
50000

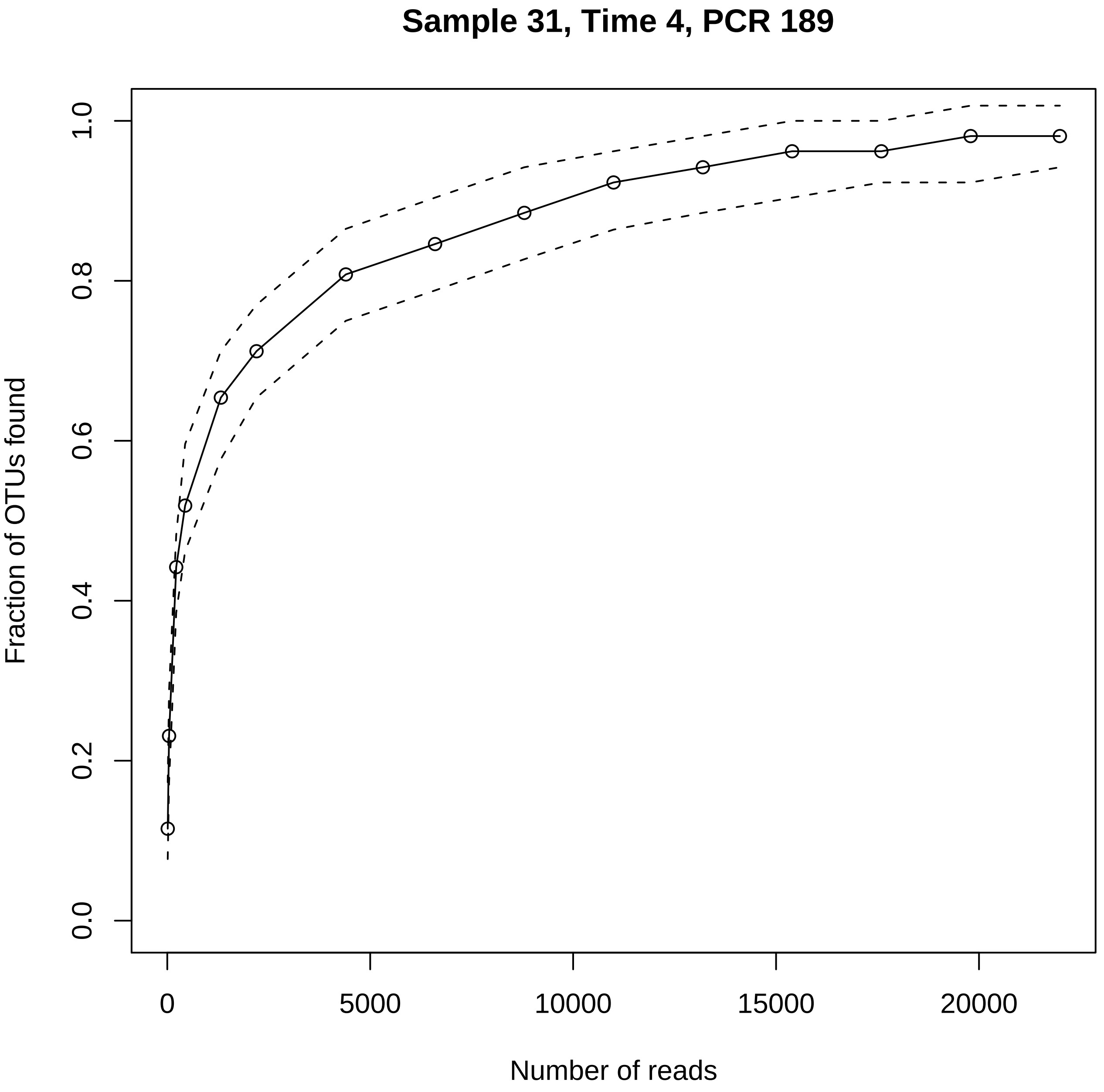
Sample 31, Time 4, PCR 189
Fraction of OTUs found
Number of reads
0.0
0.2
0.4
0.6
0.8
1.0
0
5000
10000
15000
20000

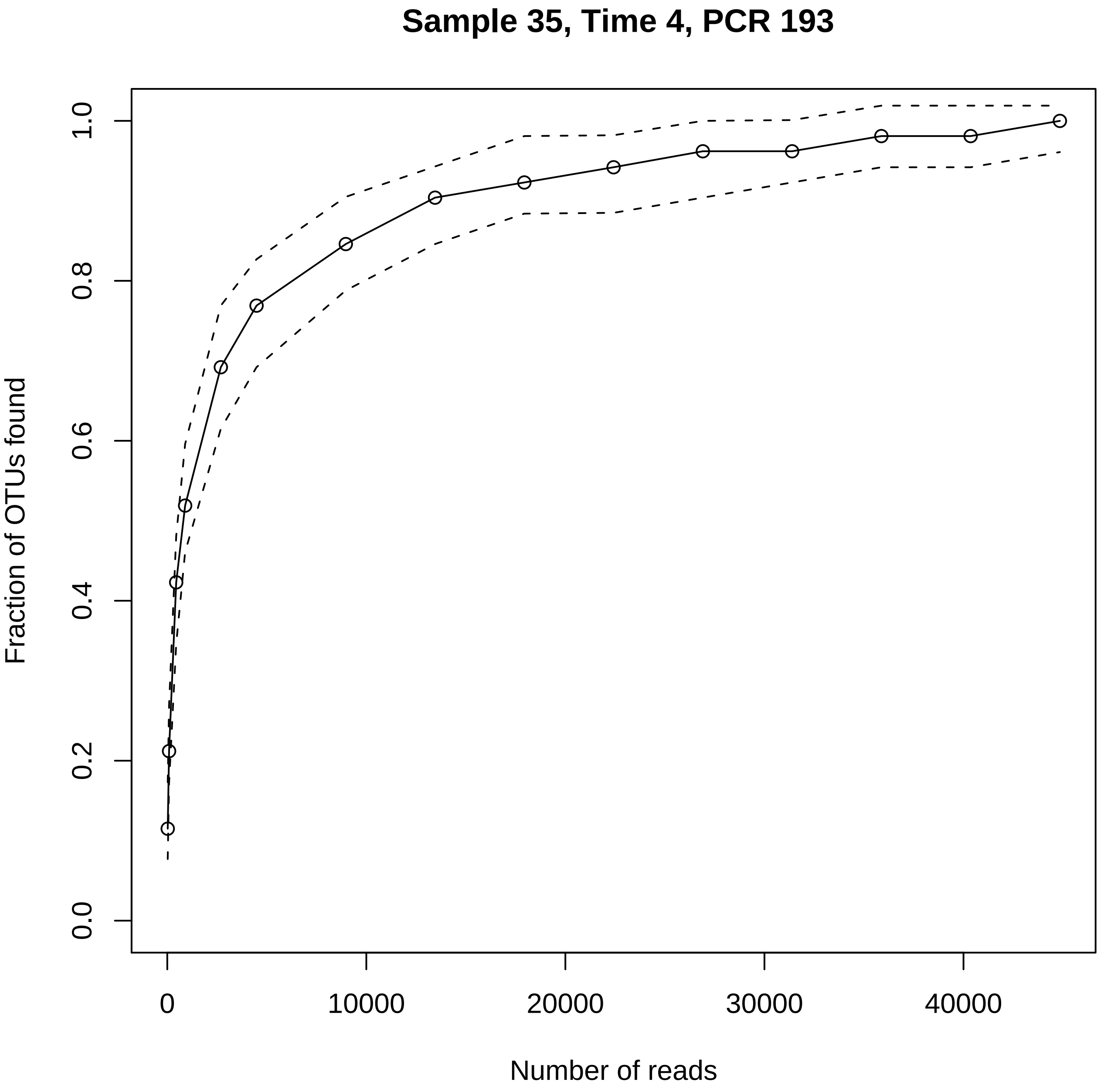
Sample 35, Time 4, PCR 193
Fraction of OTUs found
0.0
0.2
0.4
0.6
0.8
1.0
0
10000
20000
30000
40000
Number of reads

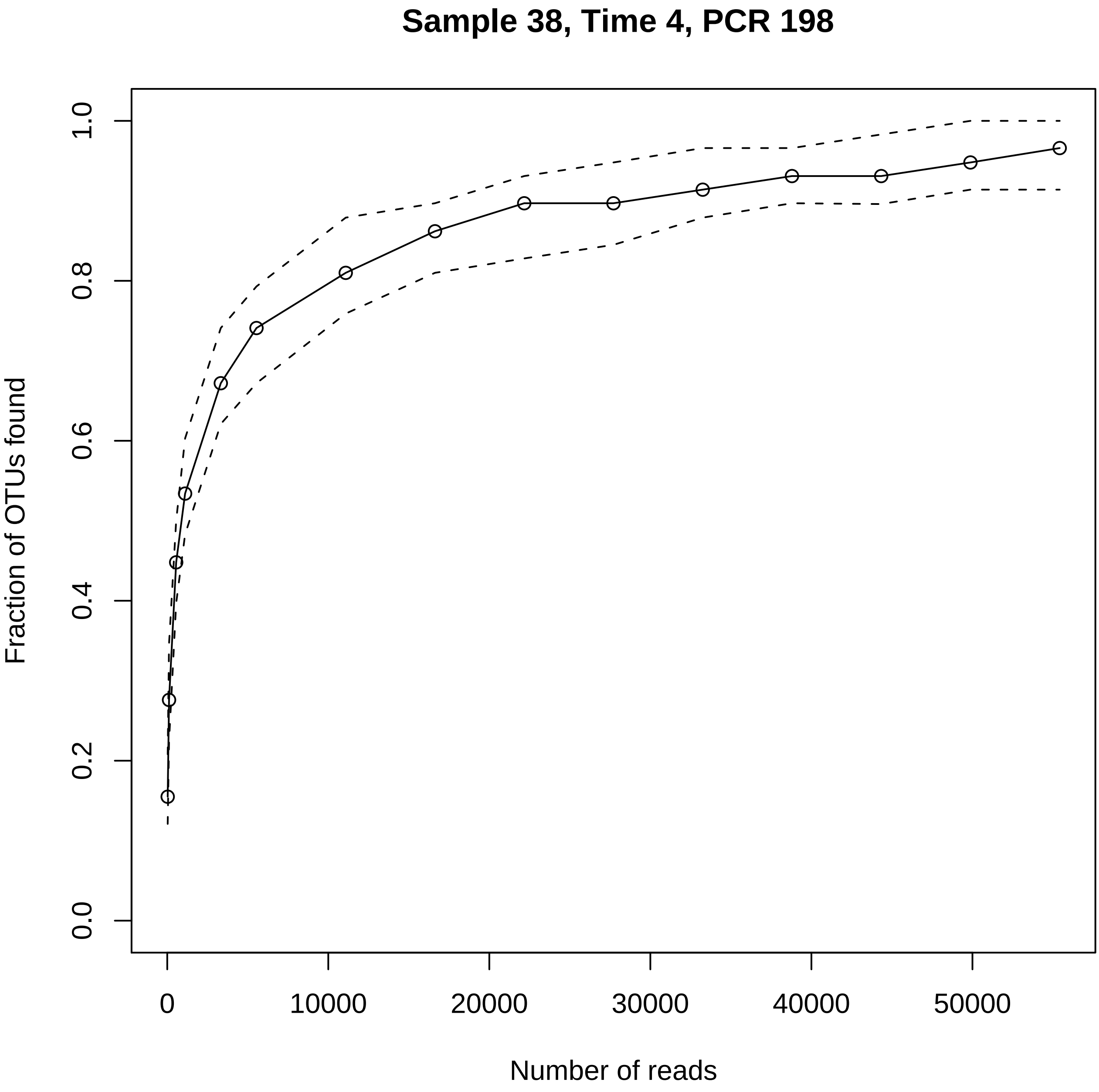

Sample 38, Time 4, PCR 198
Fraction of OTUs found
0.0
0.2
0.4
0.6
0.8
1.0
0
10000
20000
30000
40000
50000
Number of reads

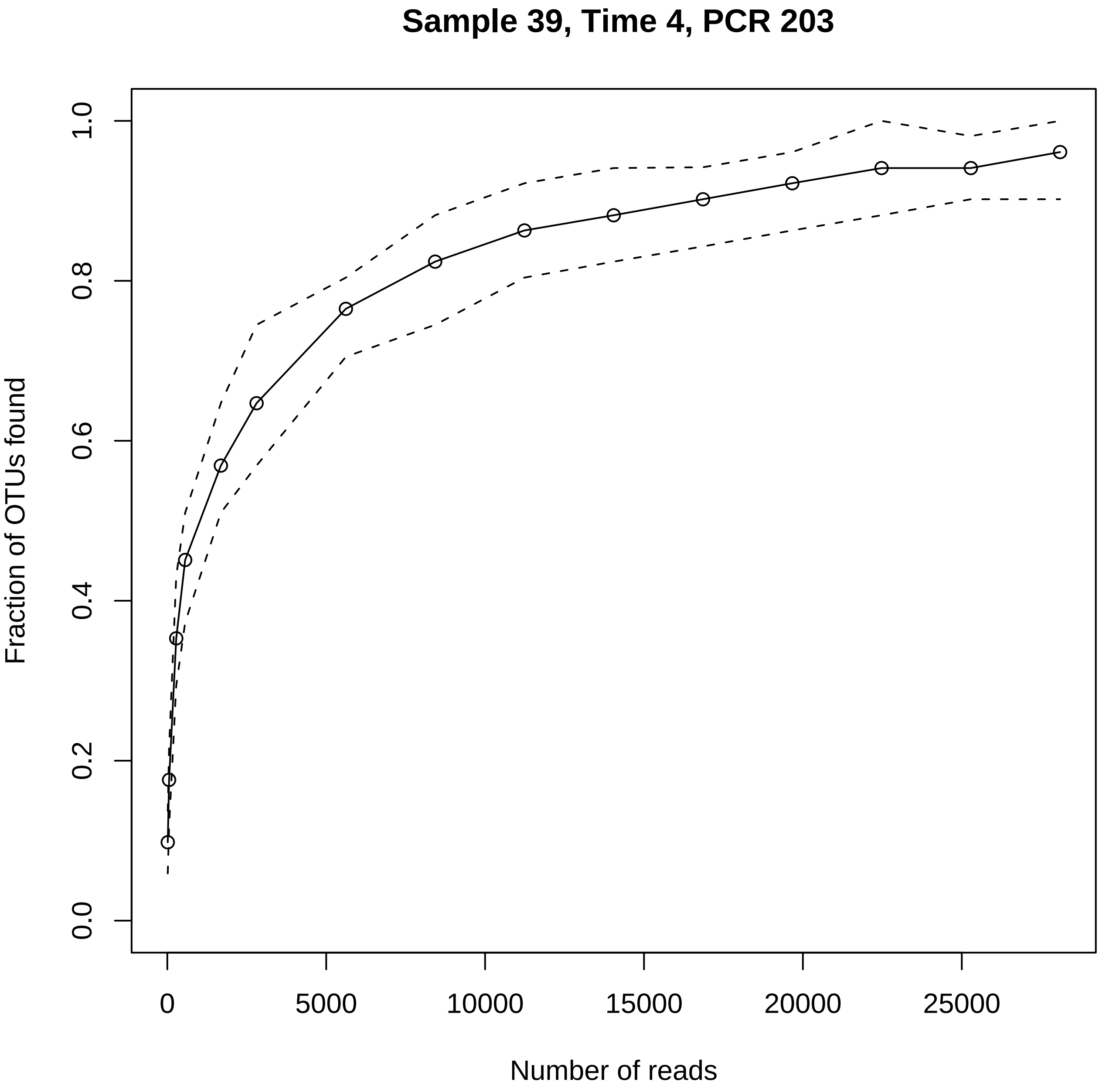

Sample 39, Time 4, PCR 203
Fraction of OTUs found
0.0
0.2
0.4
0.6
0.8
1.0
0
5000
10000
15000
20000
25000
Number of reads

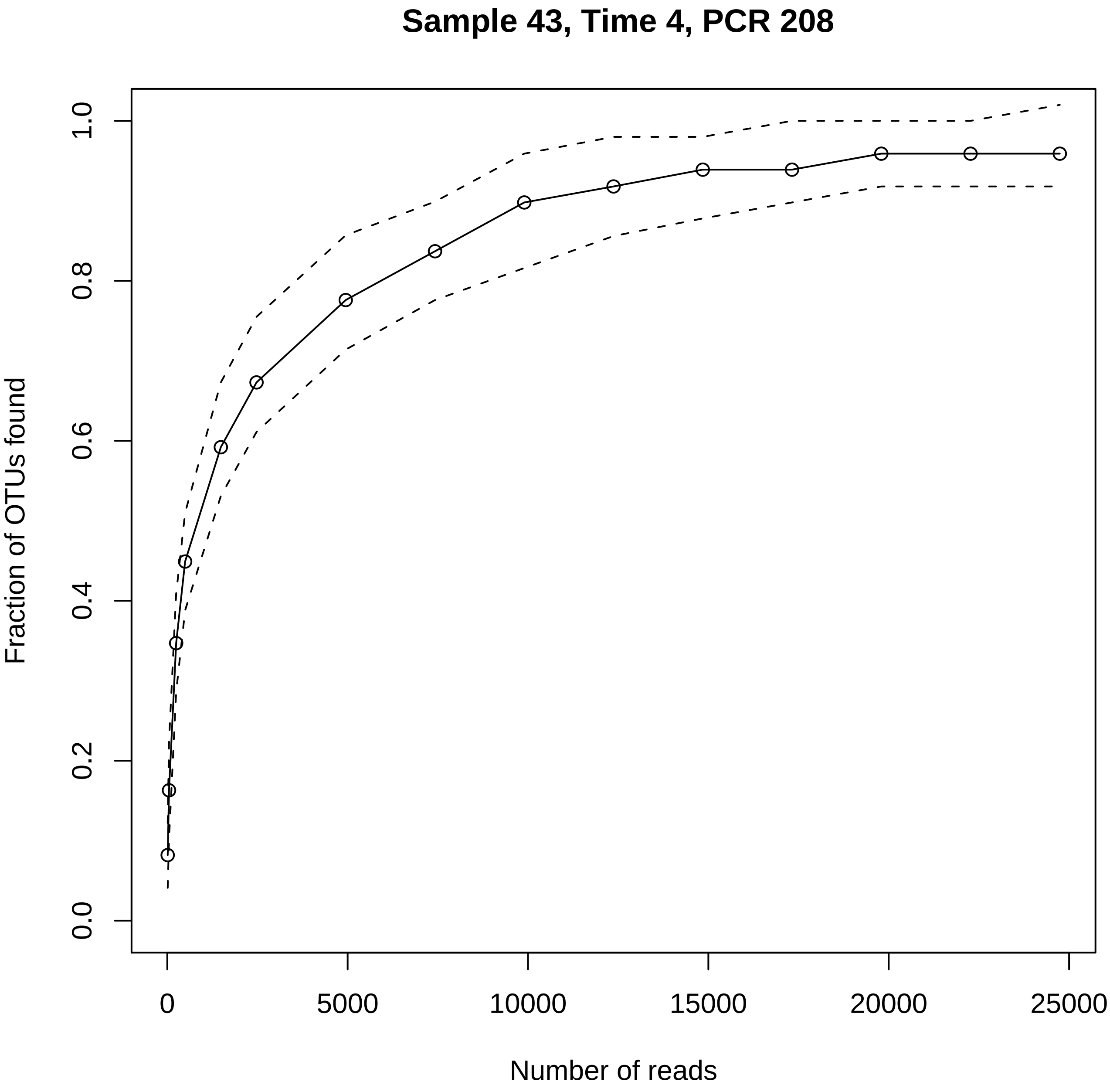

Sample 43, Time 4, PCR 208
Fraction of OTUs found
0.0
0.2
0.4
0.6
0.8
1.0
0
5000
10000
15000
20000
25000
Number of reads

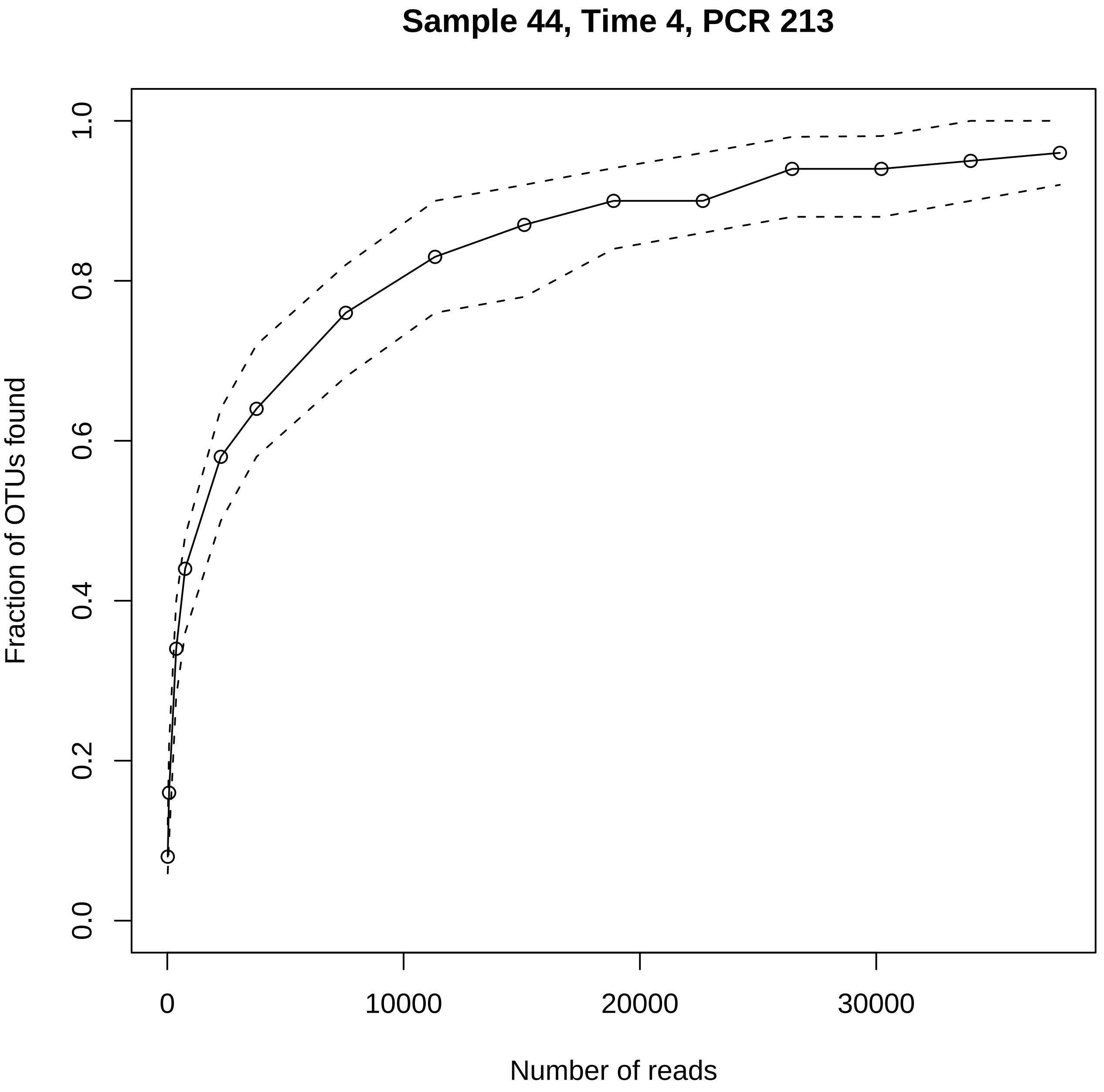
Sample 44, Time 4, PCR 213
Fraction of OTUs found
0.0
0.2
0.4
0.6
0.8
1.0
0
10000
20000
30000
Number of reads

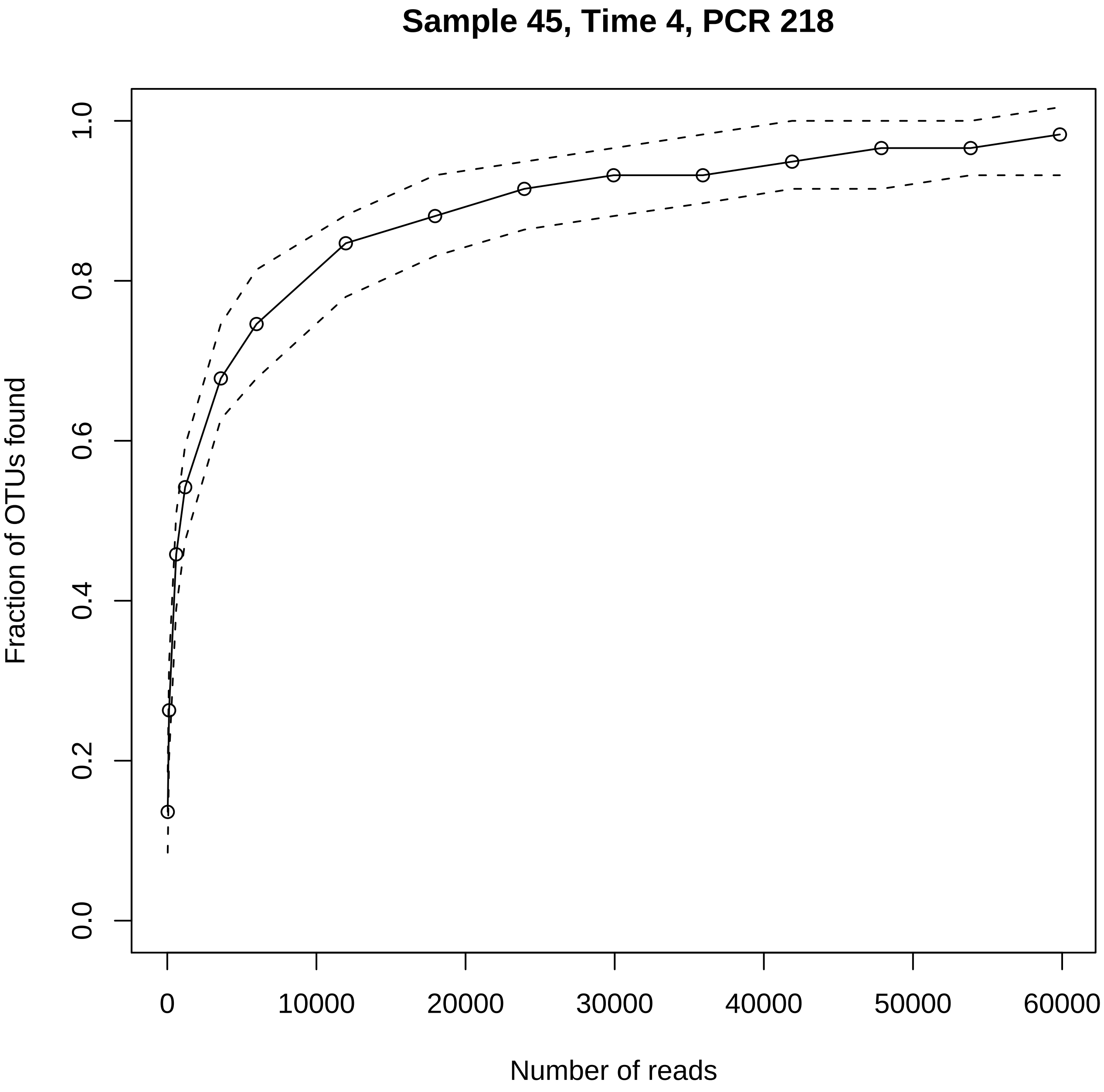

Sample 45, Time 4, PCR 218
Fraction of OTUs found
0.0
0.2
0.4
0.6
0.8
1.0
0
10000
20000
30000
40000
50000
60000
Number of reads

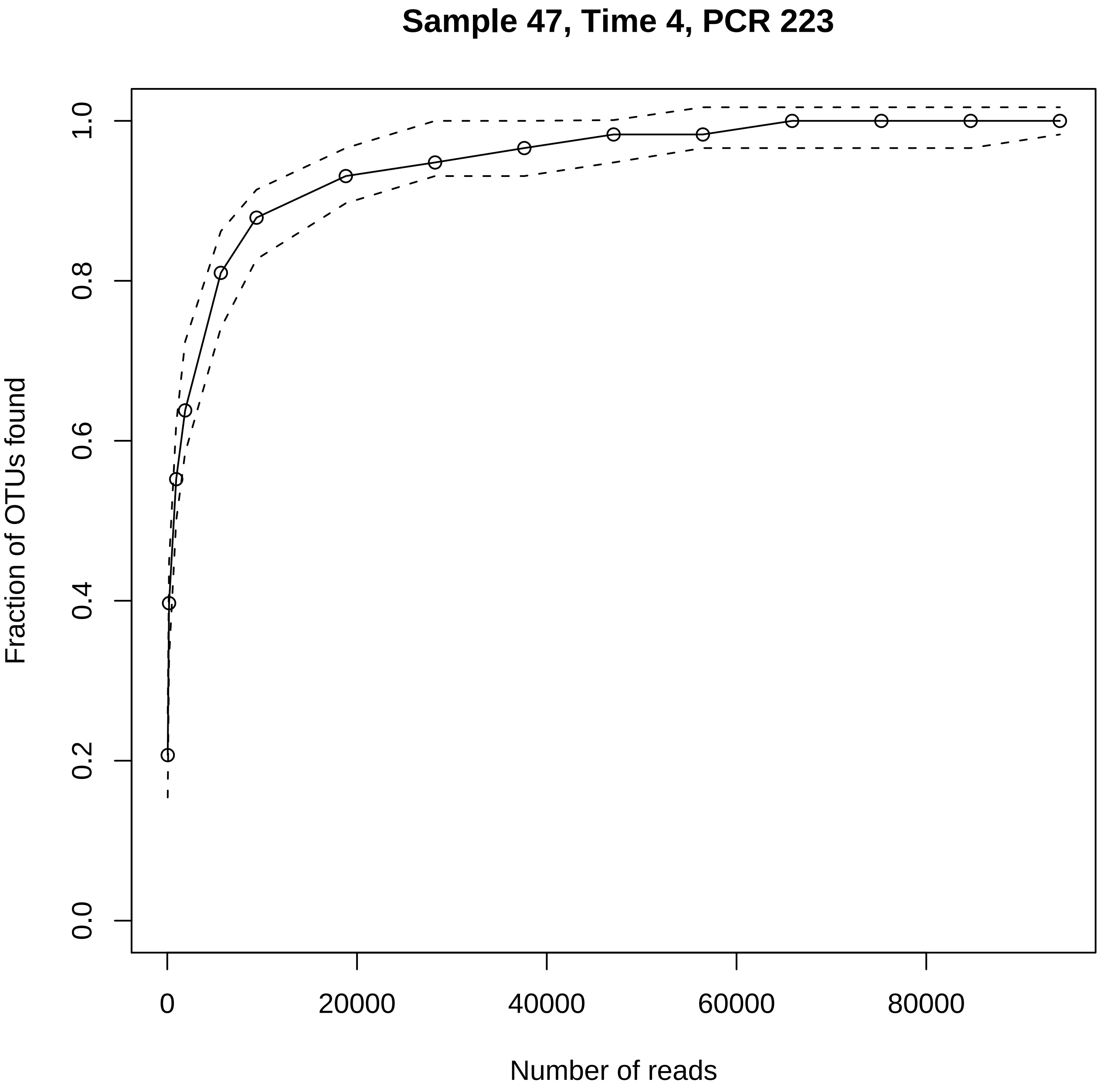

Sample 47, Time 4, PCR 223
Fraction of OTUs found
0.0
0.2
0.4
0.6
0.8
1.0
0
20000
40000
60000
80000
Number of reads

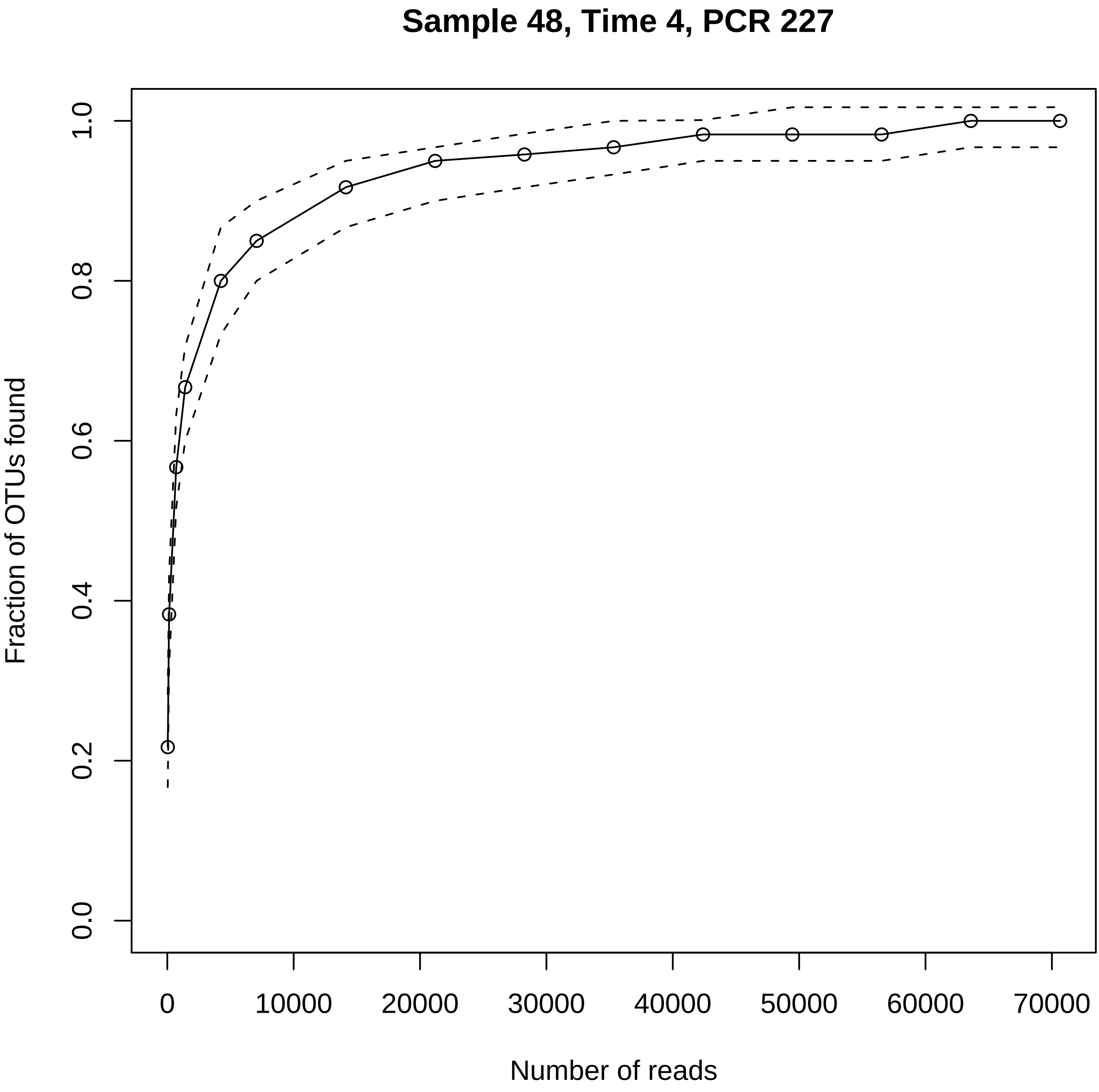

Sample 48, Time 4, PCR 227
Fraction of OTUs found
0.0
0.2
0.4
0.6
0.8
1.0
0
10000
20000
30000
40000
50000
60000
70000
Number of reads

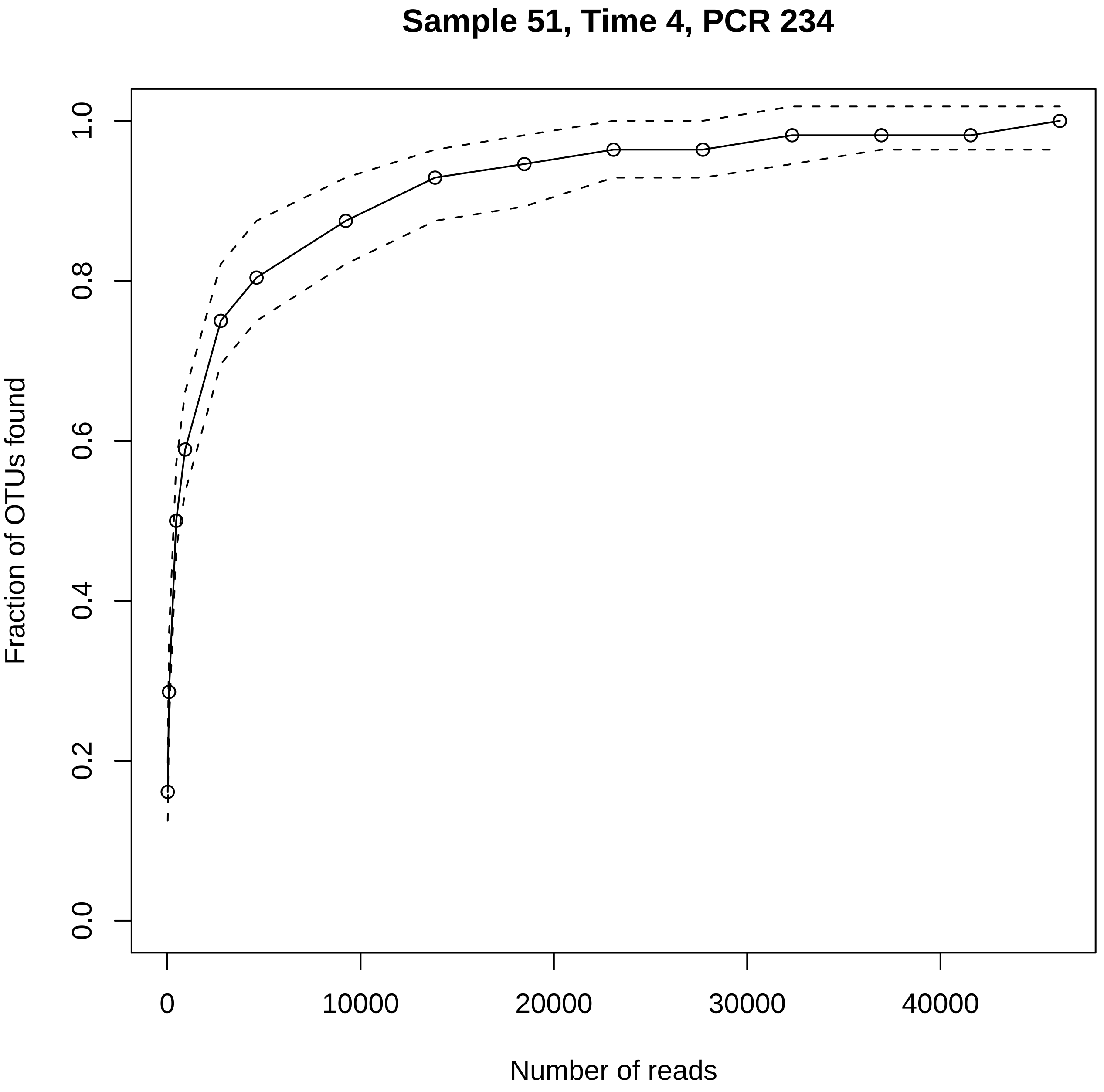
Sample 51, Time 4, PCR 234
Fraction of OTUs found
0.0
0.2
0.4
0.6
0.8
1.0
0
10000
20000
30000
40000
Number of reads

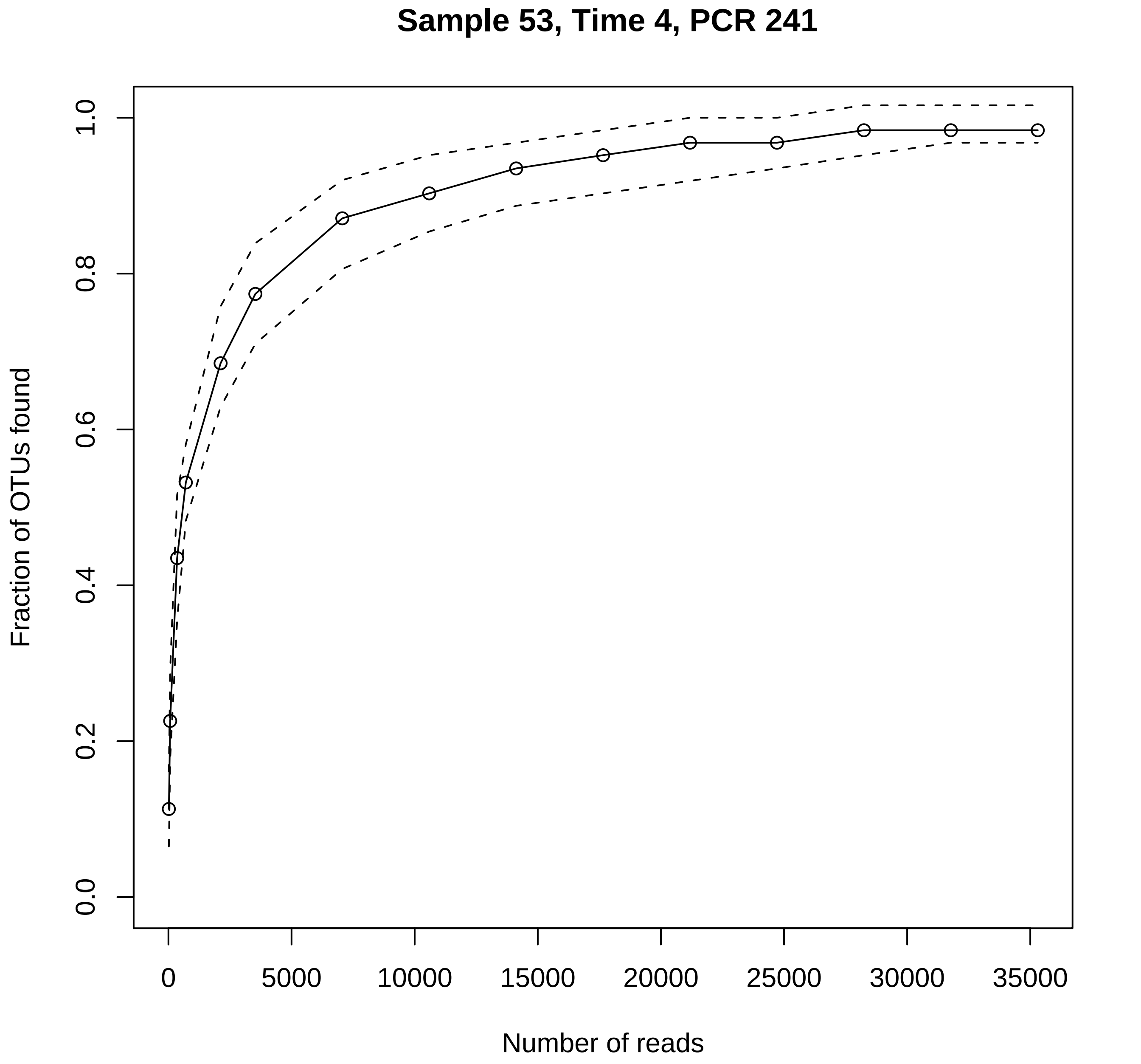
Sample 53, Time 4, PCR 241
Fraction of OTUs found
0.0
0.2
0.4
0.6
0.8
1.0
0
5000
10000
15000
20000
25000
30000
35000
Number of reads

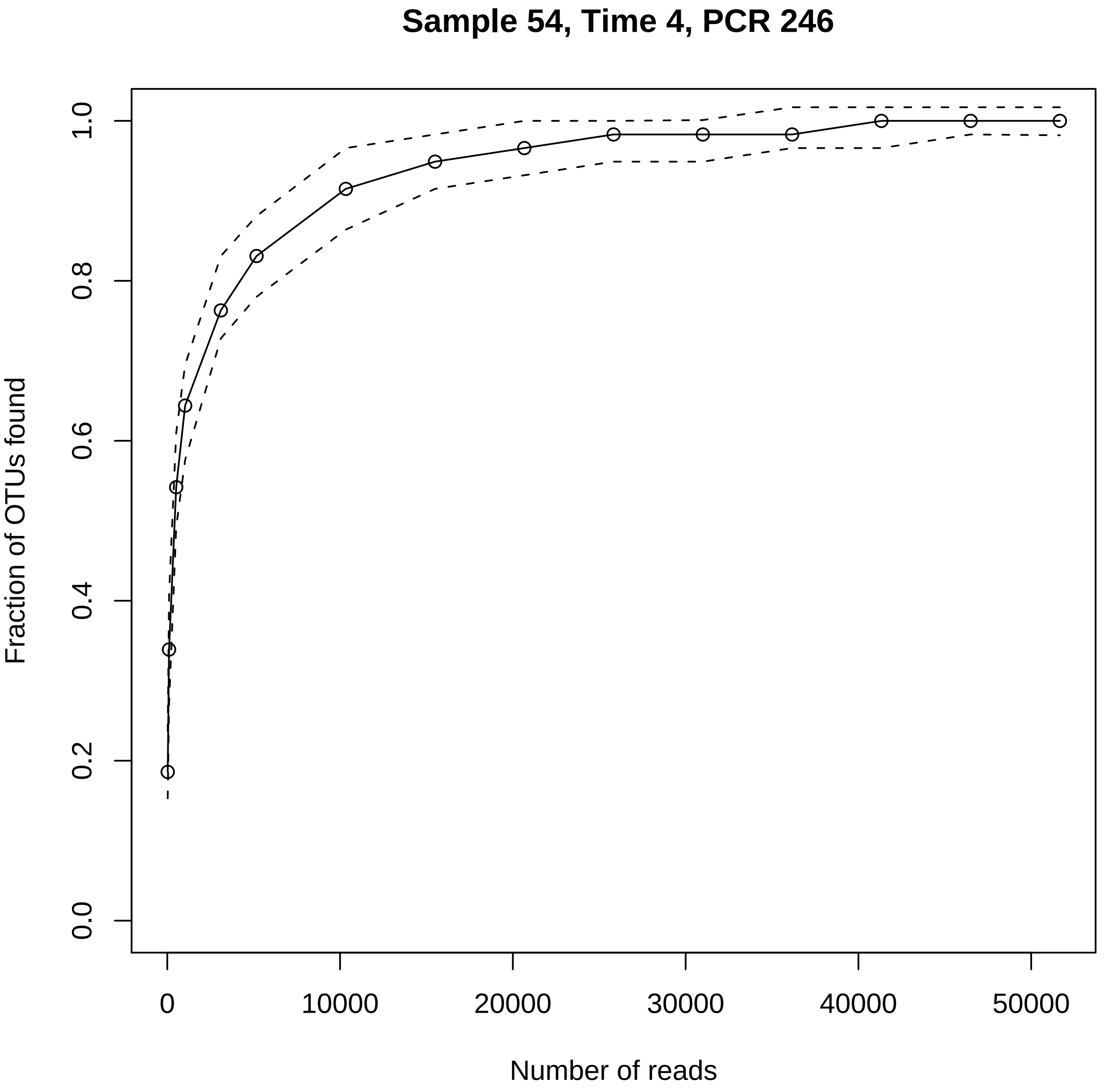
Sample 54, Time 4, PCR 246
Fraction of OTUs found
0.0
0.2
0.4
0.6
0.8
1.0
0
10000
20000
30000
40000
50000
Number of reads

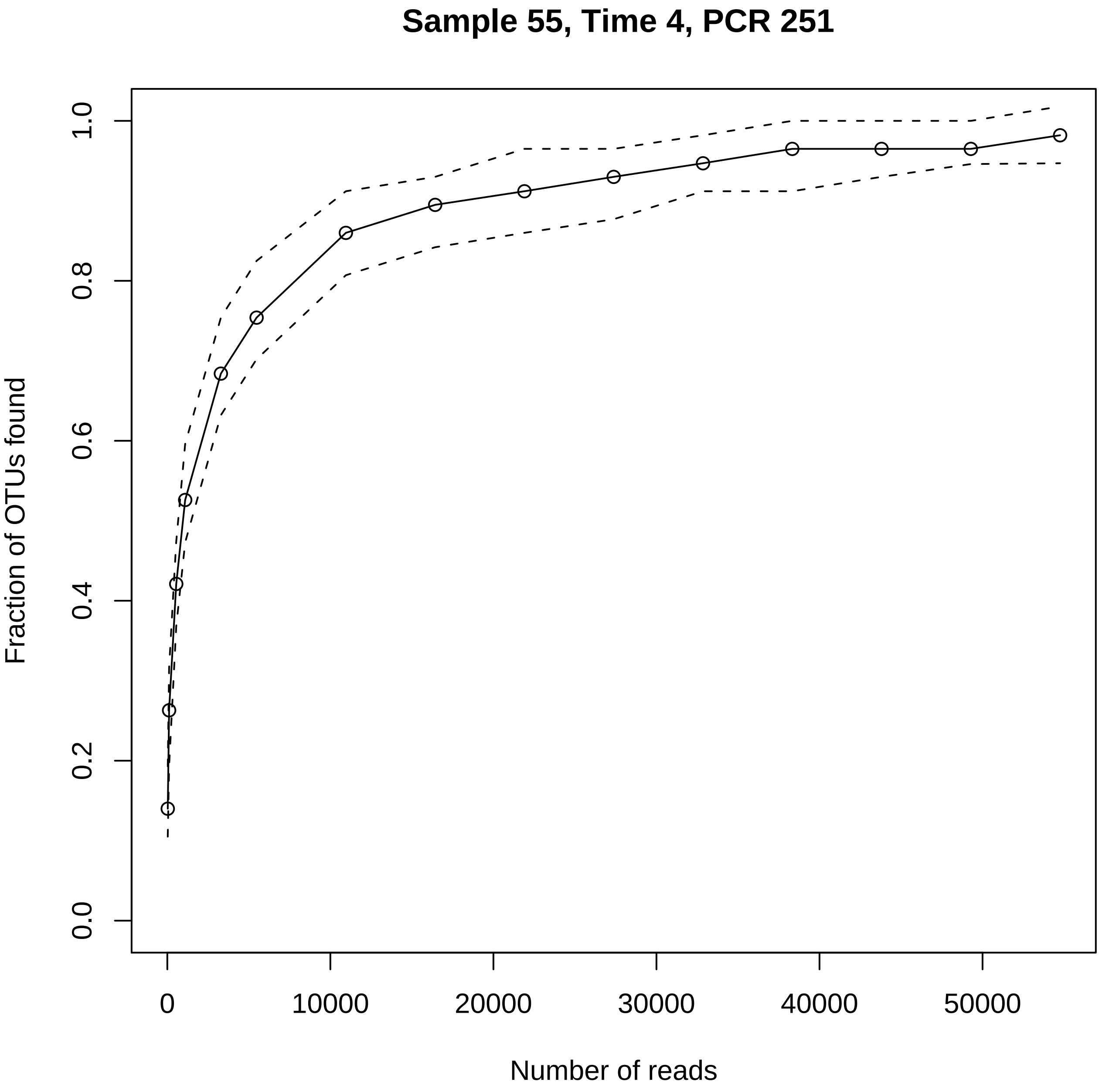

Sample 55, Time 4, PCR 251
Fraction of OTUs found
0.0
0.2
0.4
0.6
0.8
1.0
0
10000
20000
30000
40000
50000
Number of reads

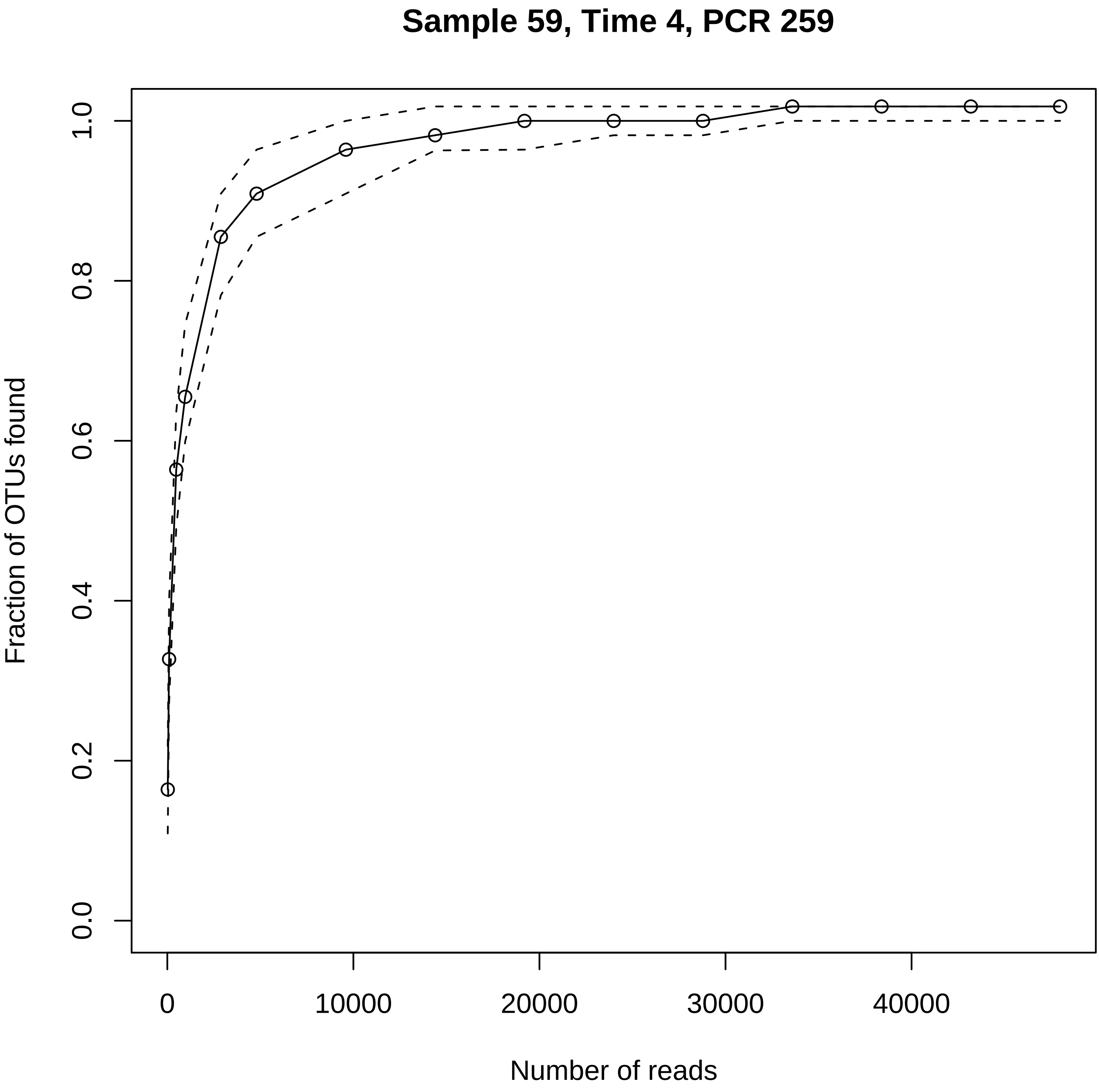
Sample 59, Time 4, PCR 259
Fraction of OTUs found
Number of reads
0.0
0.2
0.4
0.6
0.8
1.0
0
10000
20000
30000
40000

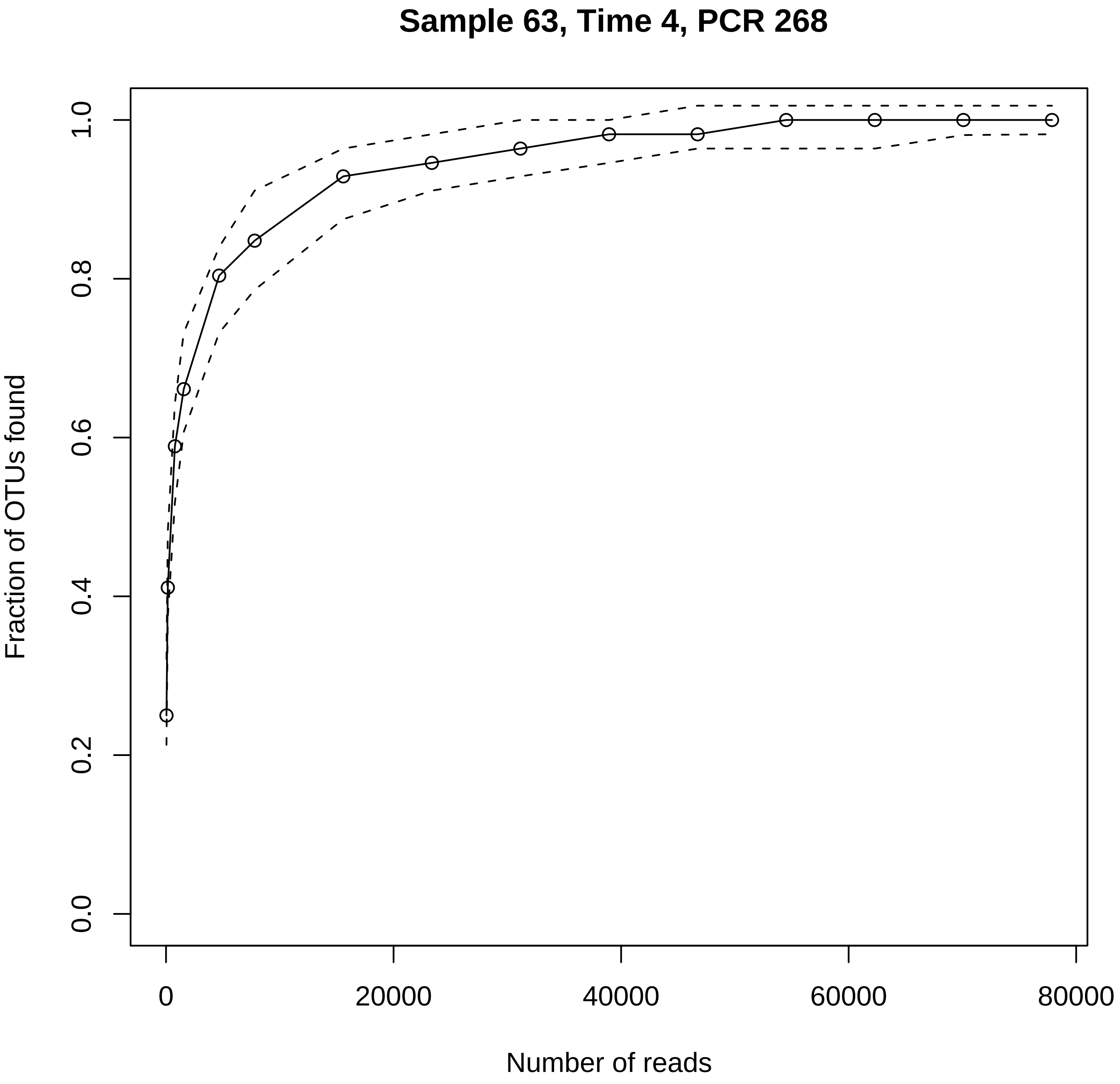
Sample 63, Time 4, PCR 268
Fraction of OTUs found
0.0
0.2
0.4
0.6
0.8
1.0
0
20000
40000
60000
80000
Number of reads

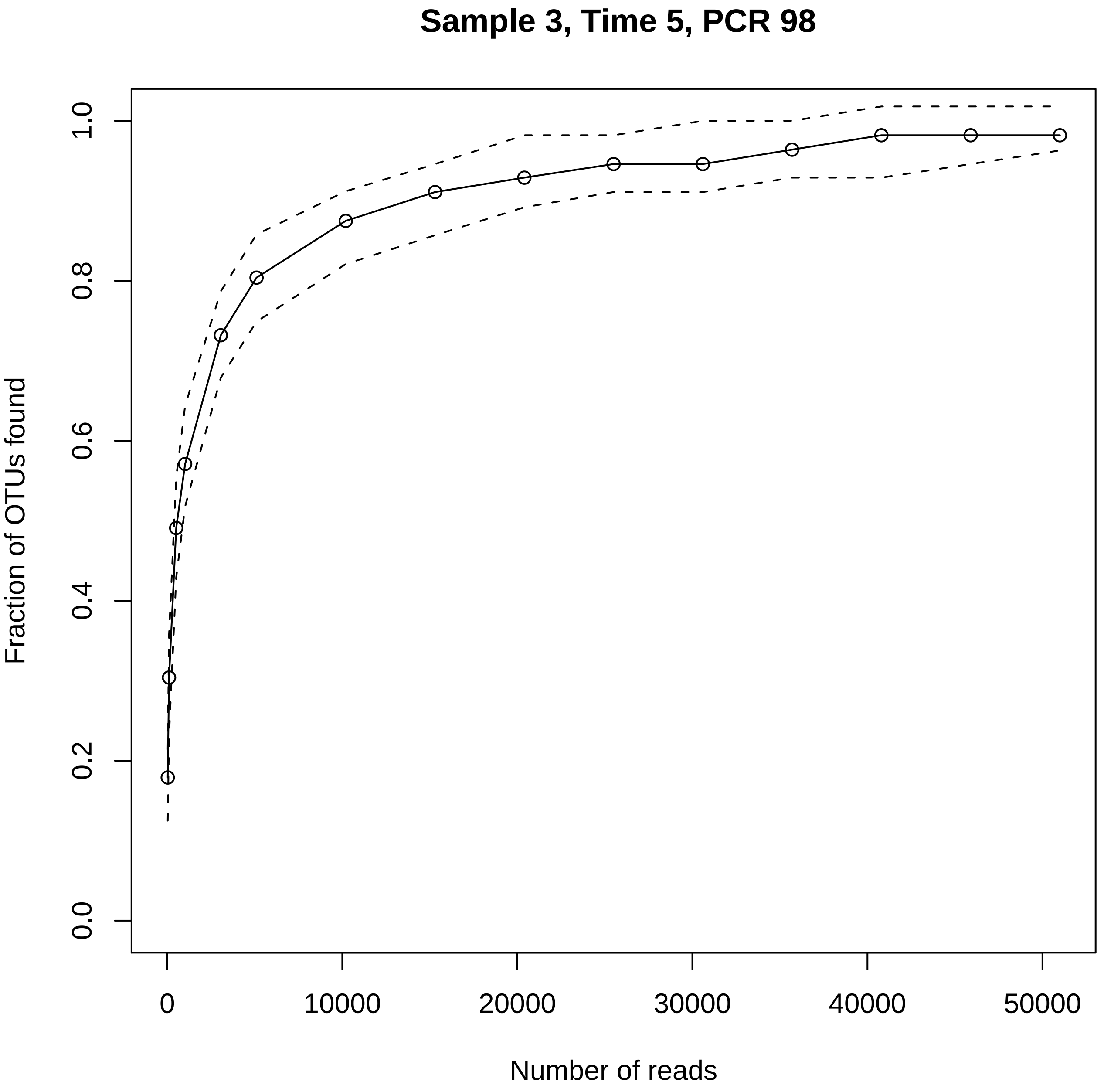
Sample 3, Time 5, PCR 98
Fraction of OTUs found
0.0
0.2
0.4
0.6
0.8
1.0
0
10000
20000
30000
40000
50000
Number of reads

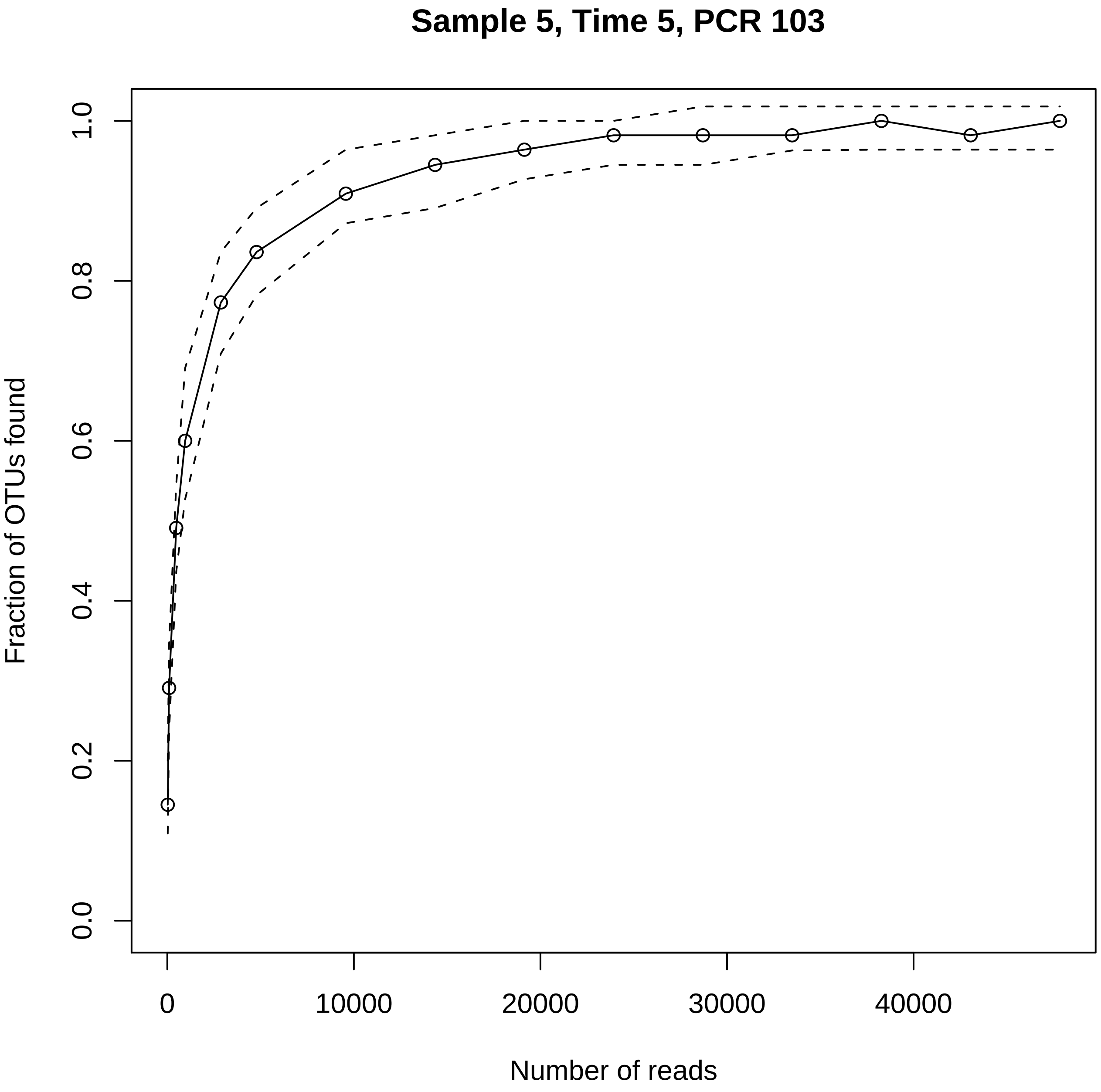
Sample 5, Time 5, PCR 103
Fraction of OTUs found
Number of reads
0.0
0.2
0.4
0.6
0.8
1.0
0
10000
20000
30000
40000

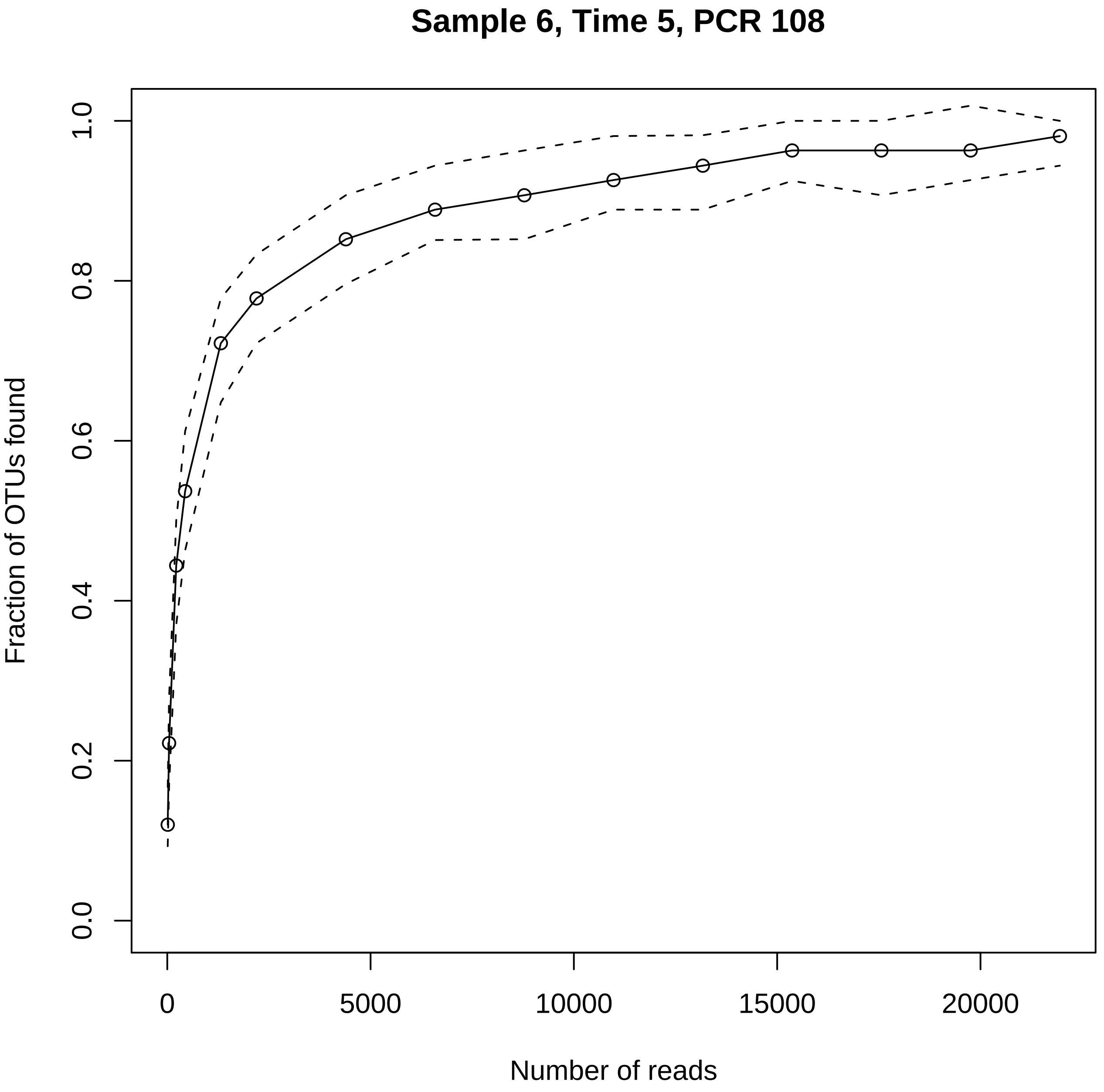

Sample 6, Time 5, PCR 108
Fraction of OTUs found
0.0
0.2
0.4
0.6
0.8
1.0
0
5000
10000
15000
20000
Number of reads

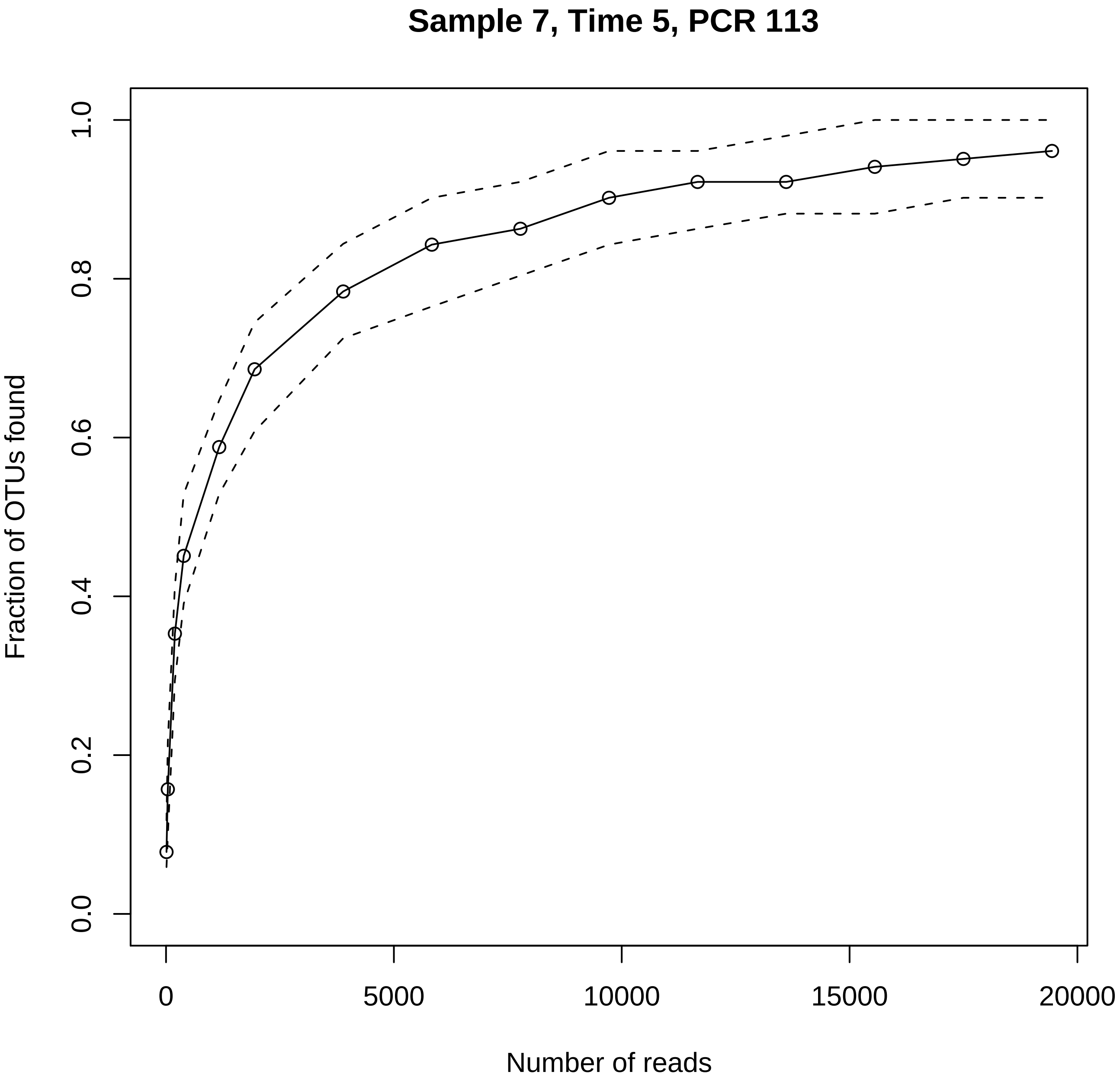
Sample 7, Time 5, PCR 113
Fraction of OTUs found
0.0
0.2
0.4
0.6
0.8
1.0
0
5000
10000
15000
20000
Number of reads

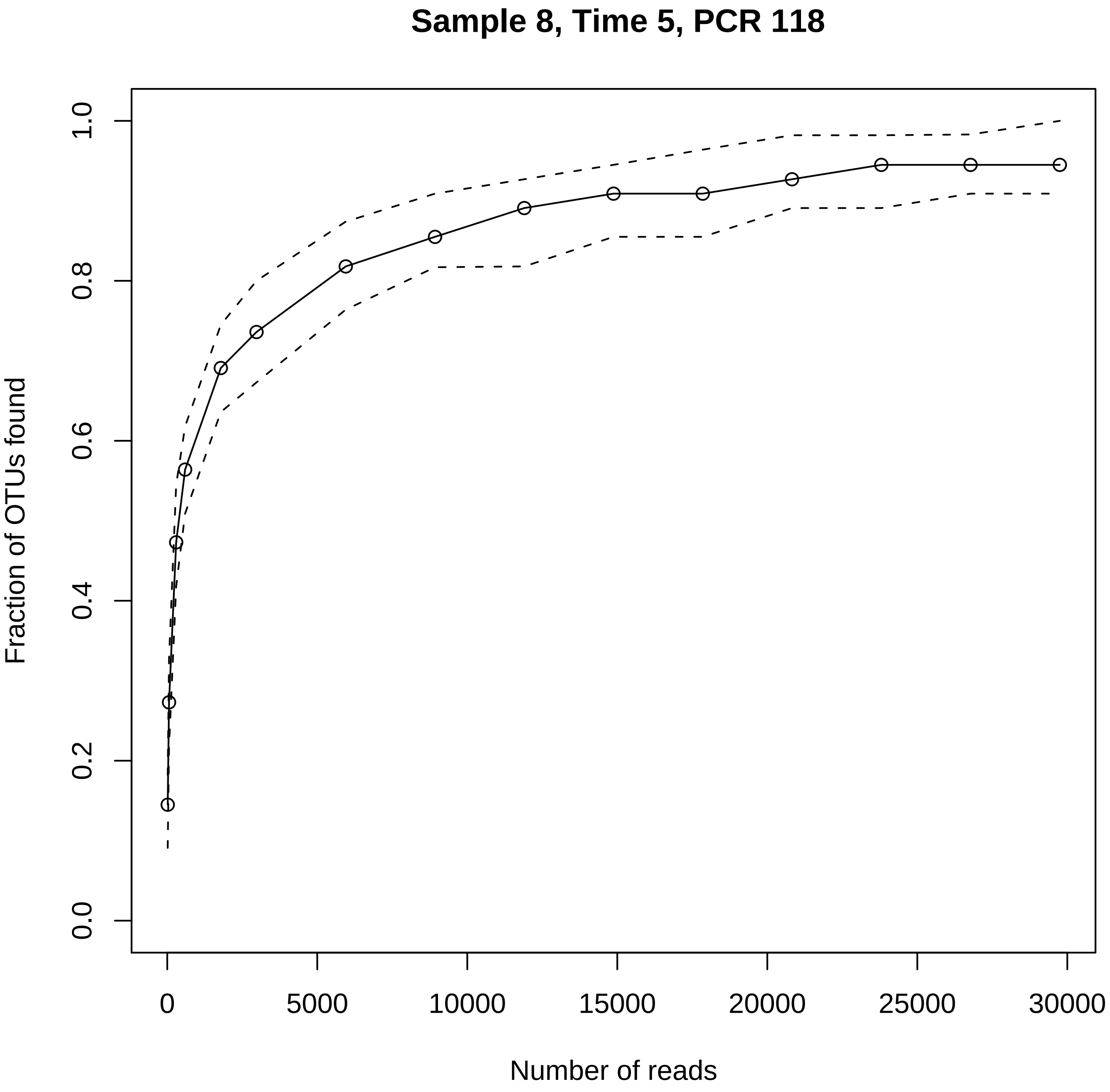
Sample 8, Time 5, PCR 118
Fraction of OTUs found
0.0
0.2
0.4
0.6
0.8
1.0
0
5000
10000
15000
20000
25000
30000
Number of reads

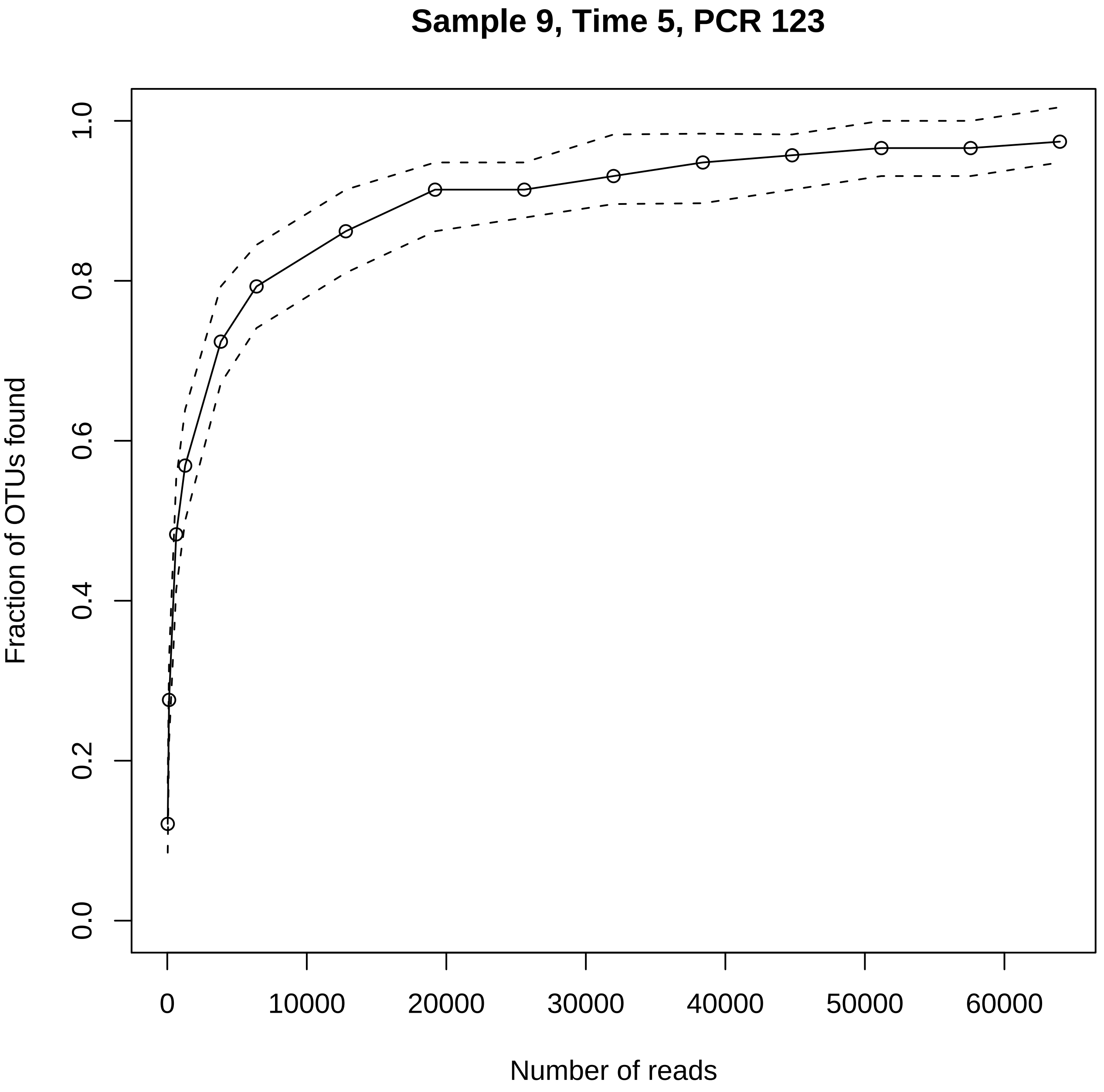
Sample 9, Time 5, PCR 123
Fraction of OTUs found
0.0
0.2
0.4
0.6
0.8
1.0
0
10000
20000
30000
40000
50000
60000
Number of reads

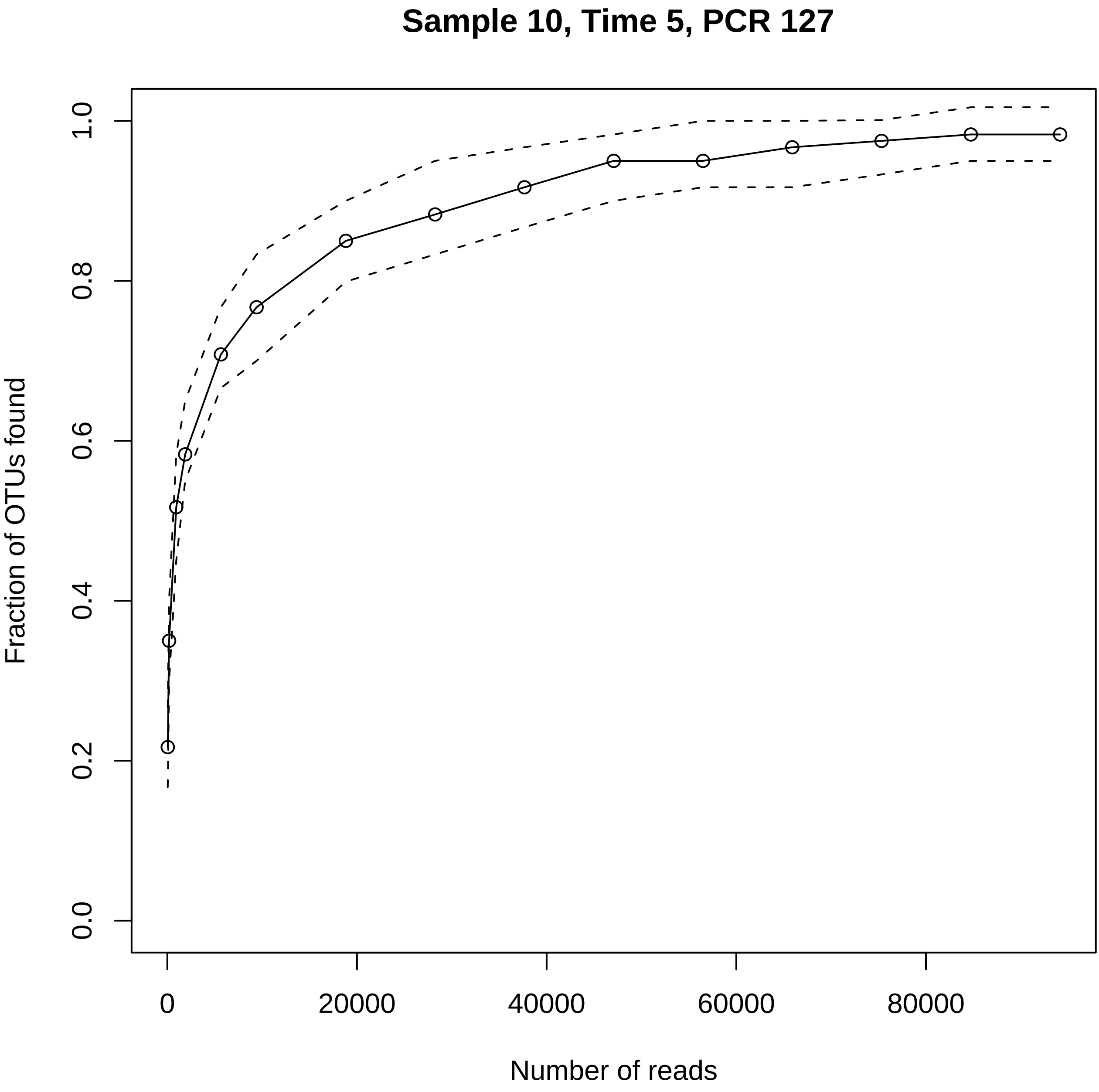
Sample 10, Time 5, PCR 127
Fraction of OTUs found
Number of reads
0.0
0.2
0.4
0.6
0.8
1.0
0
20000
40000
60000
80000

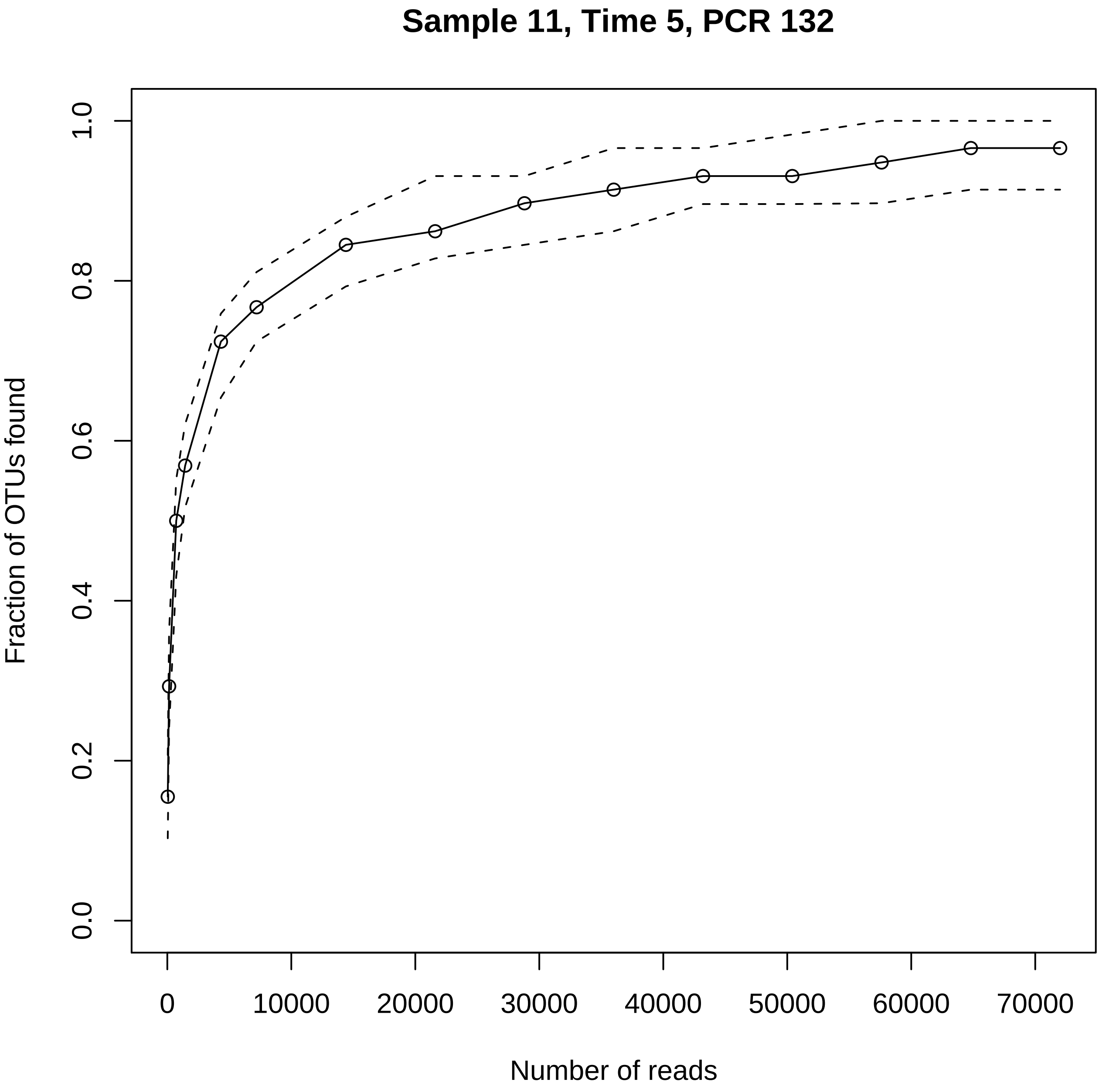
Sample 11, Time 5, PCR 132
Fraction of OTUs found
0.0
0.2
0.4
0.6
0.8
1.0
Number of reads
0
10000
20000
30000
40000
50000
60000
70000

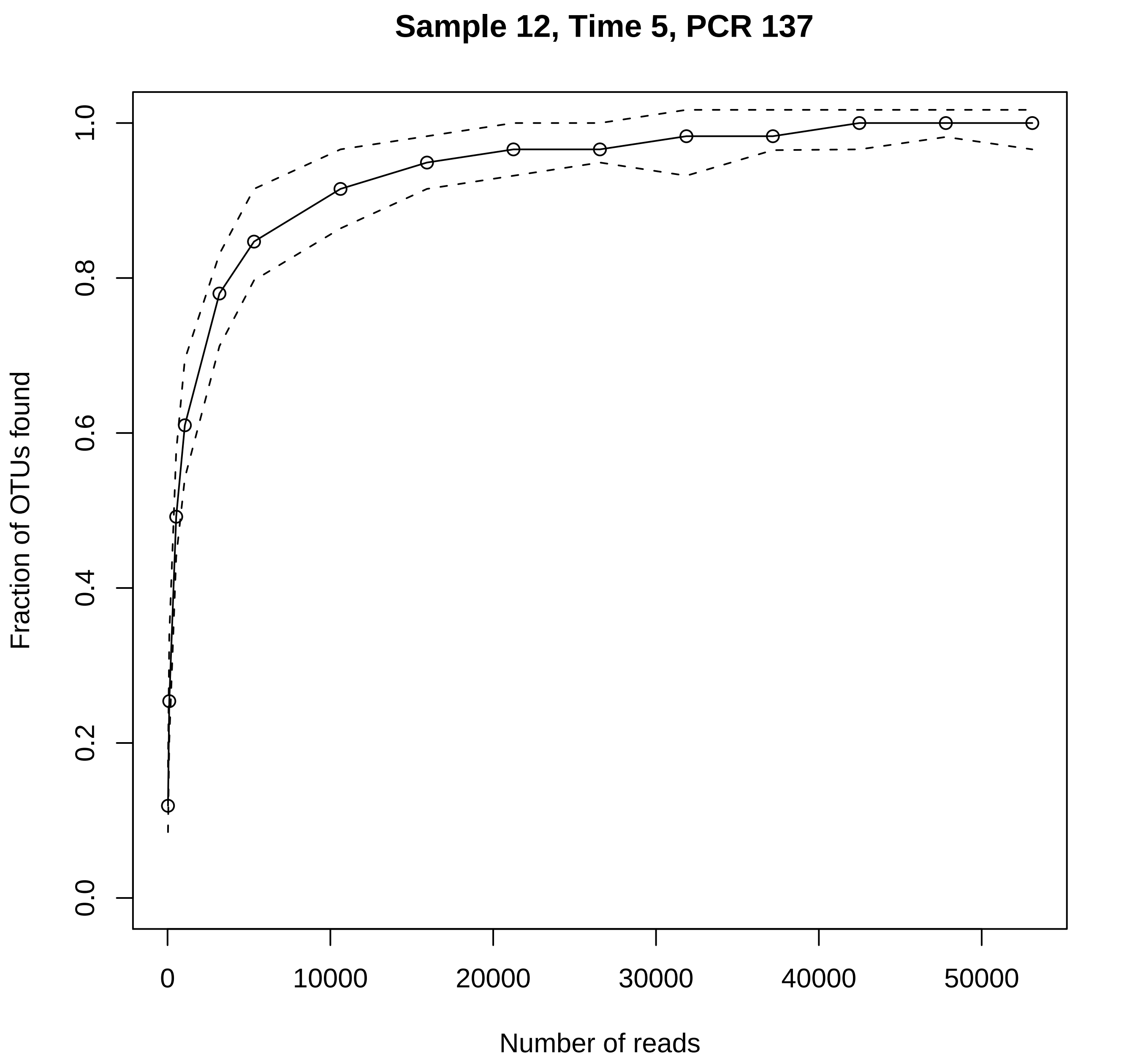
Sample 12, Time 5, PCR 137
Fraction of OTUs found
0.0
0.2
0.4
0.6
0.8
1.0
0
10000
20000
30000
40000
50000
Number of reads

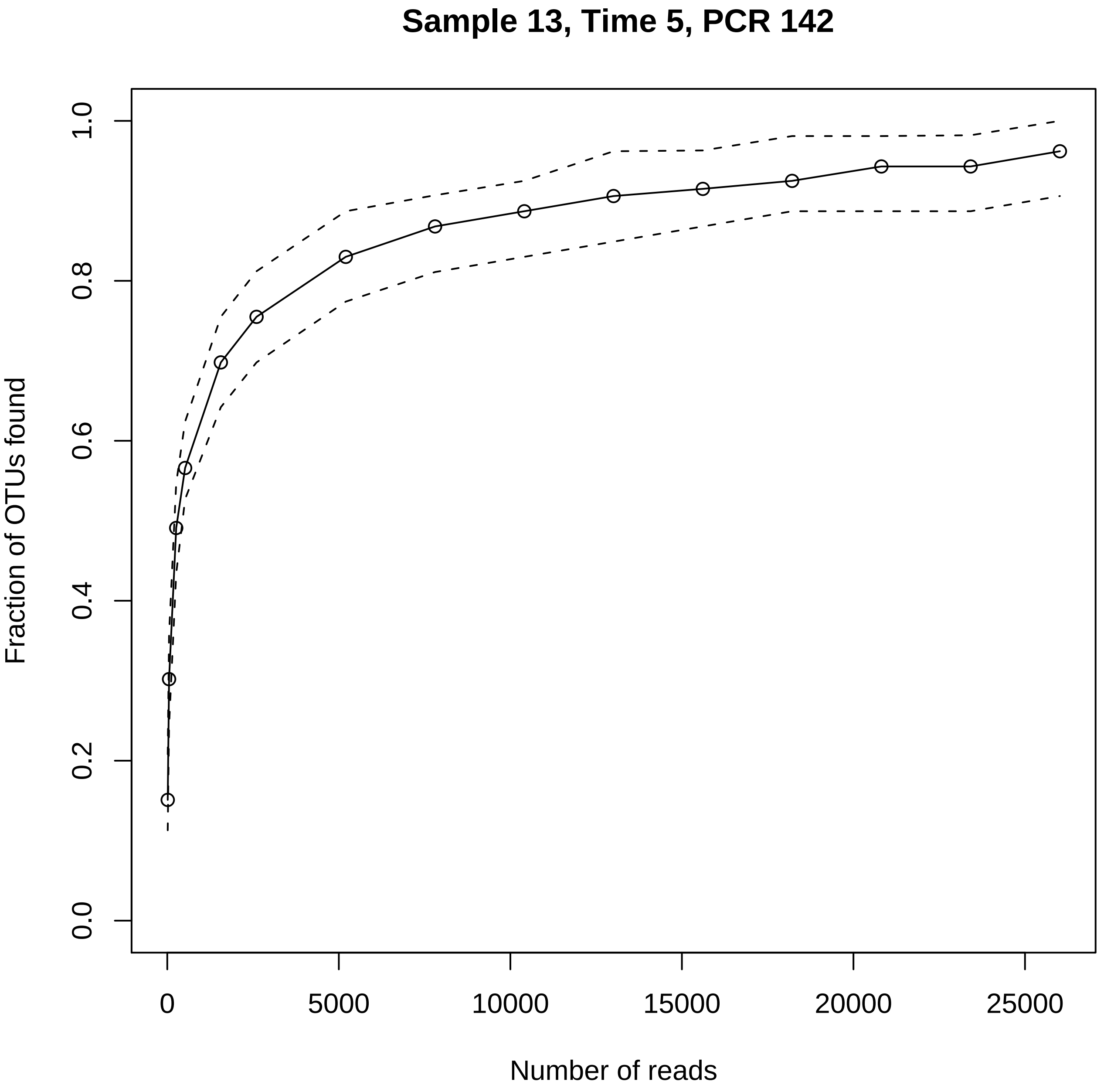

Sample 13, Time 5, PCR 142
Fraction of OTUs found
0.0
0.2
0.4
0.6
0.8
1.0
0
5000
10000
15000
20000
25000
Number of reads

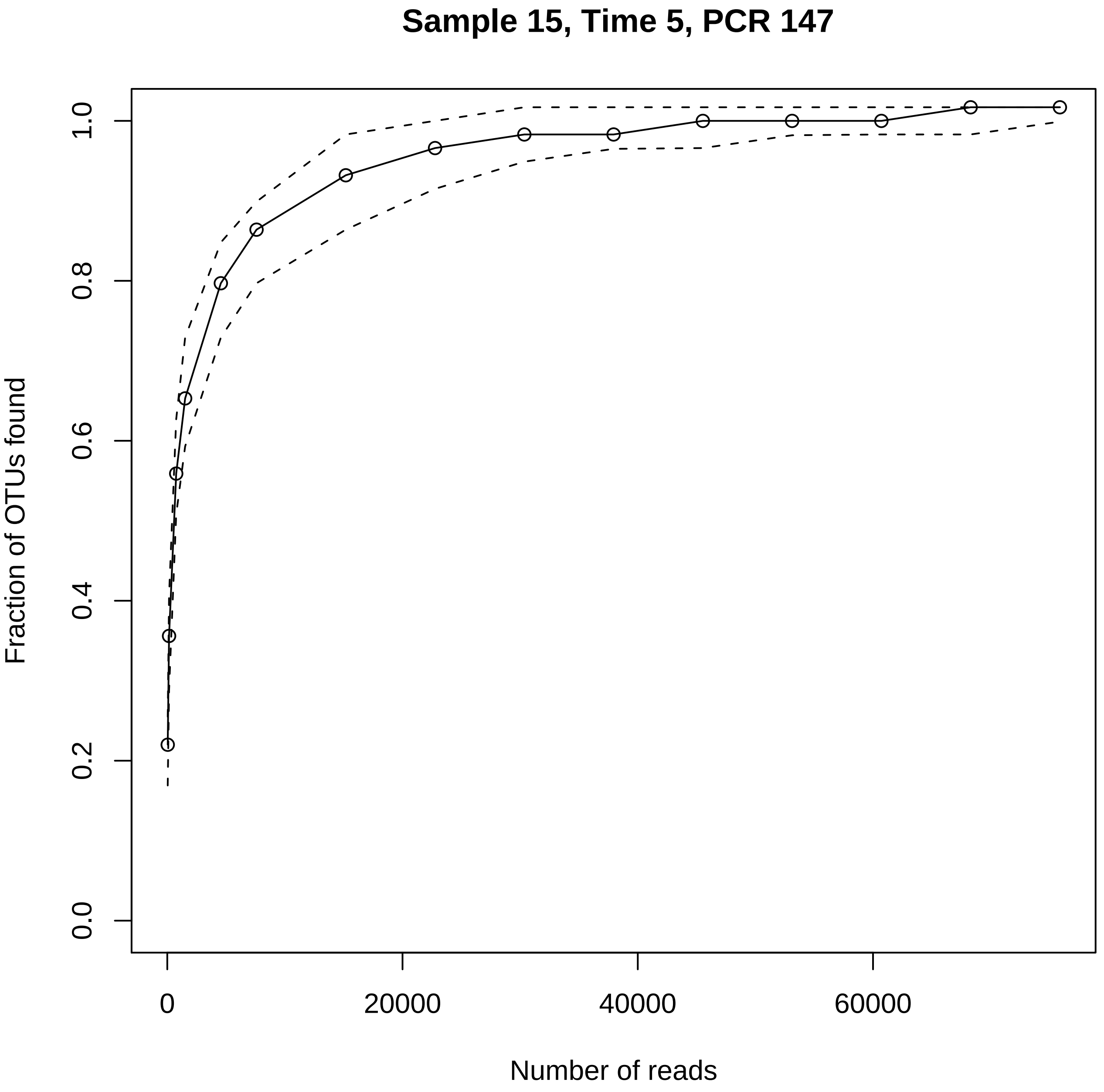
Sample 15, Time 5, PCR 147
Fraction of OTUs found
0.0
0.2
0.4
0.6
0.8
1.0
0
20000
40000
60000
Number of reads

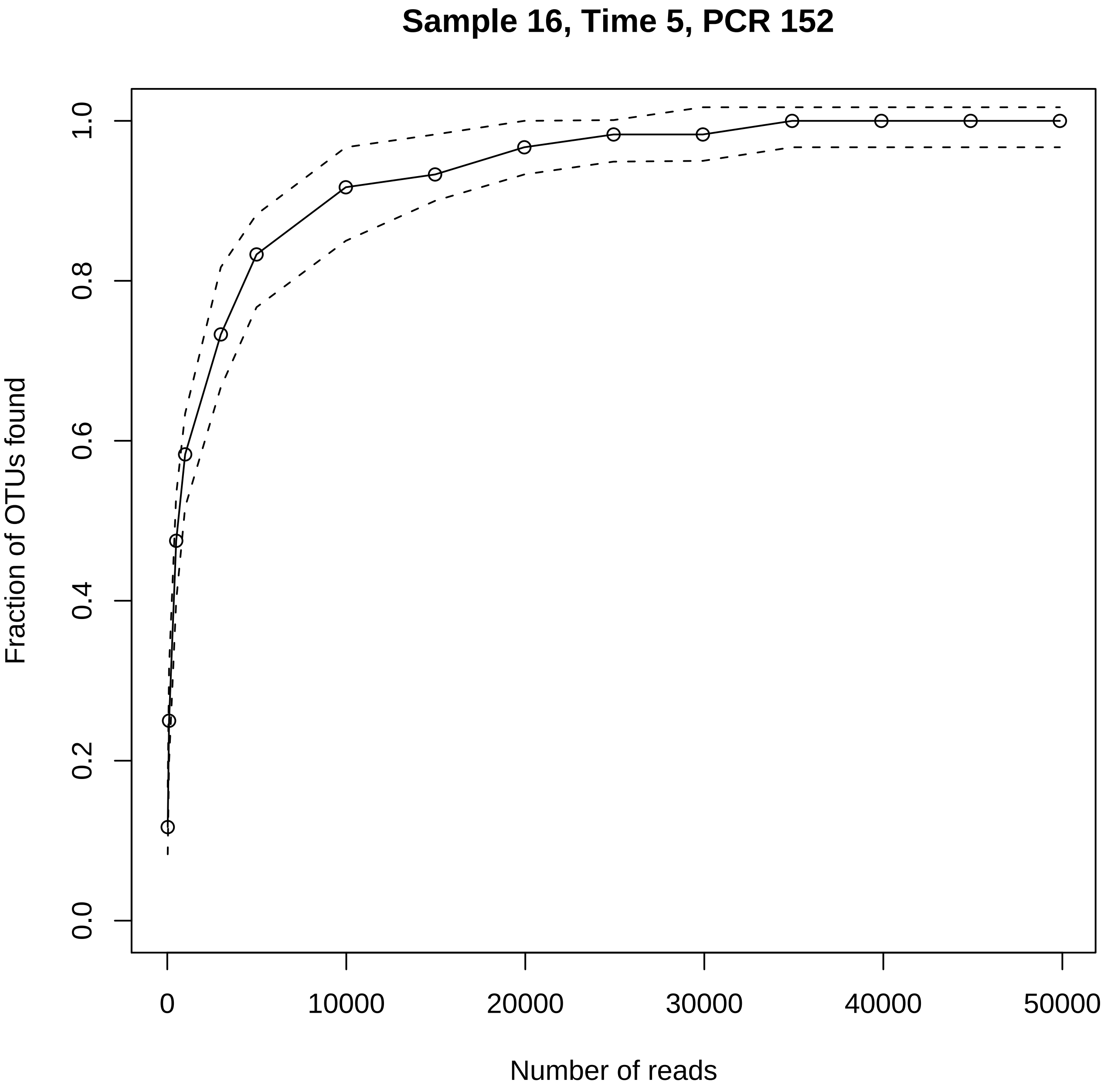

Sample 16, Time 5, PCR 152
Fraction of OTUs found
0.0
0.2
0.4
0.6
0.8
1.0
0
10000
20000
30000
40000
50000
Number of reads

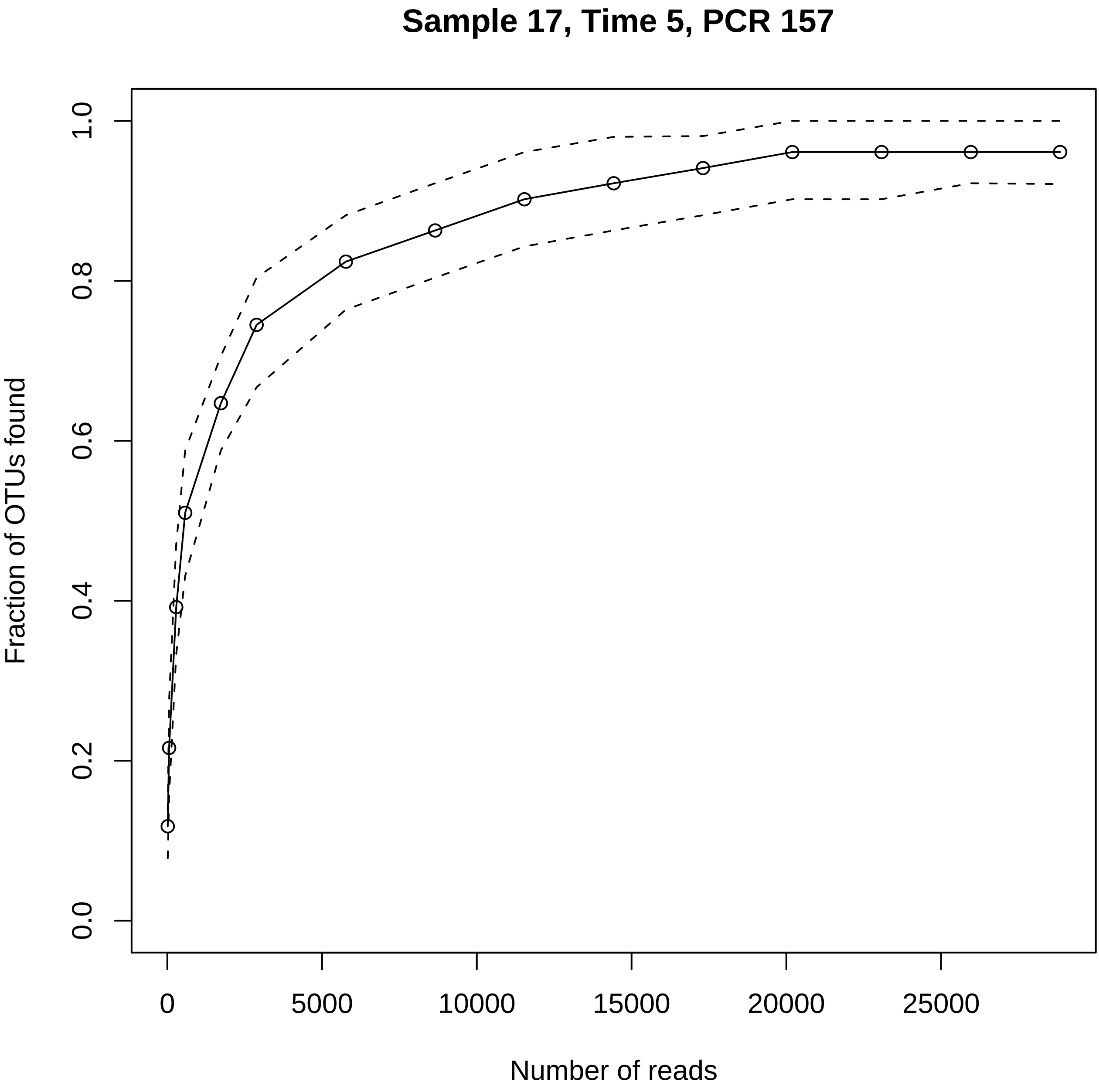
Sample 17, Time 5, PCR 157
Fraction of OTUs found
0.0
0.2
0.4
0.6
0.8
1.0
0
5000
10000
15000
20000
25000
Number of reads

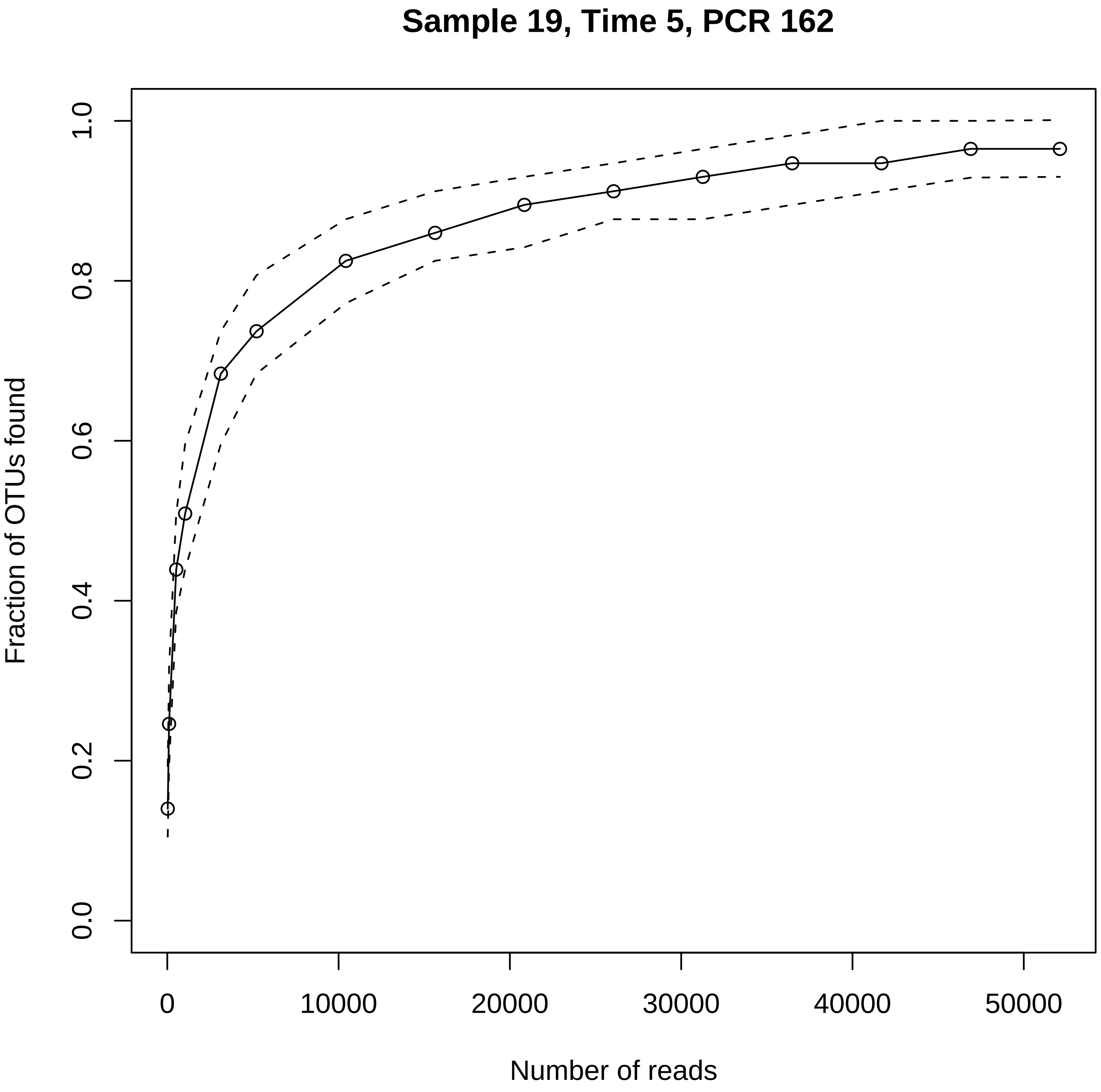
Sample 19, Time 5, PCR 162
Fraction of OTUs found
0.0
0.2
0.4
0.6
0.8
1.0
0
10000
20000
30000
40000
50000
Number of reads

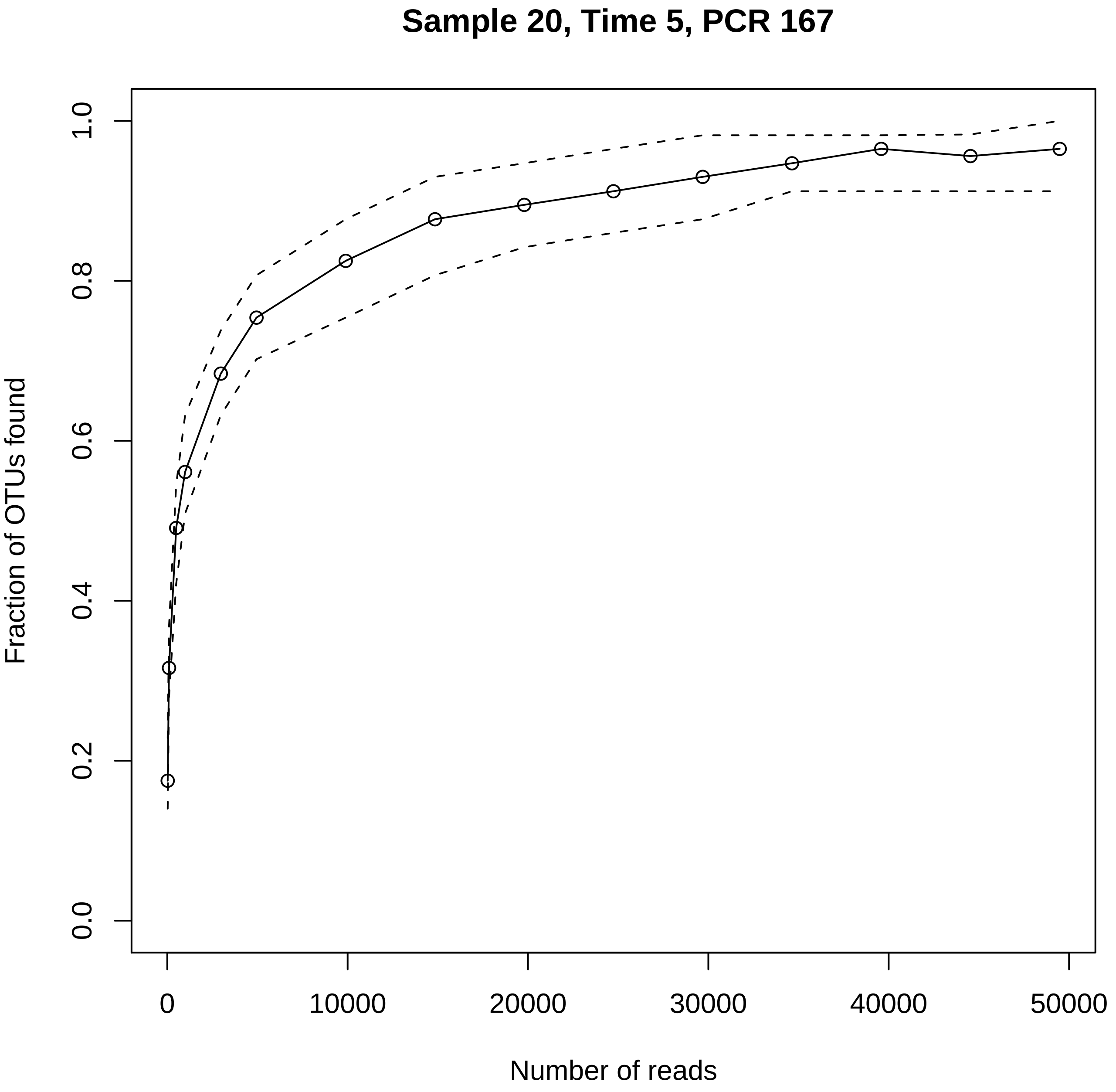

Sample 20, Time 5, PCR 167
Fraction of OTUs found
Number of reads
0.0
0.2
0.4
0.6
0.8
1.0
0
10000
20000
30000
40000
50000

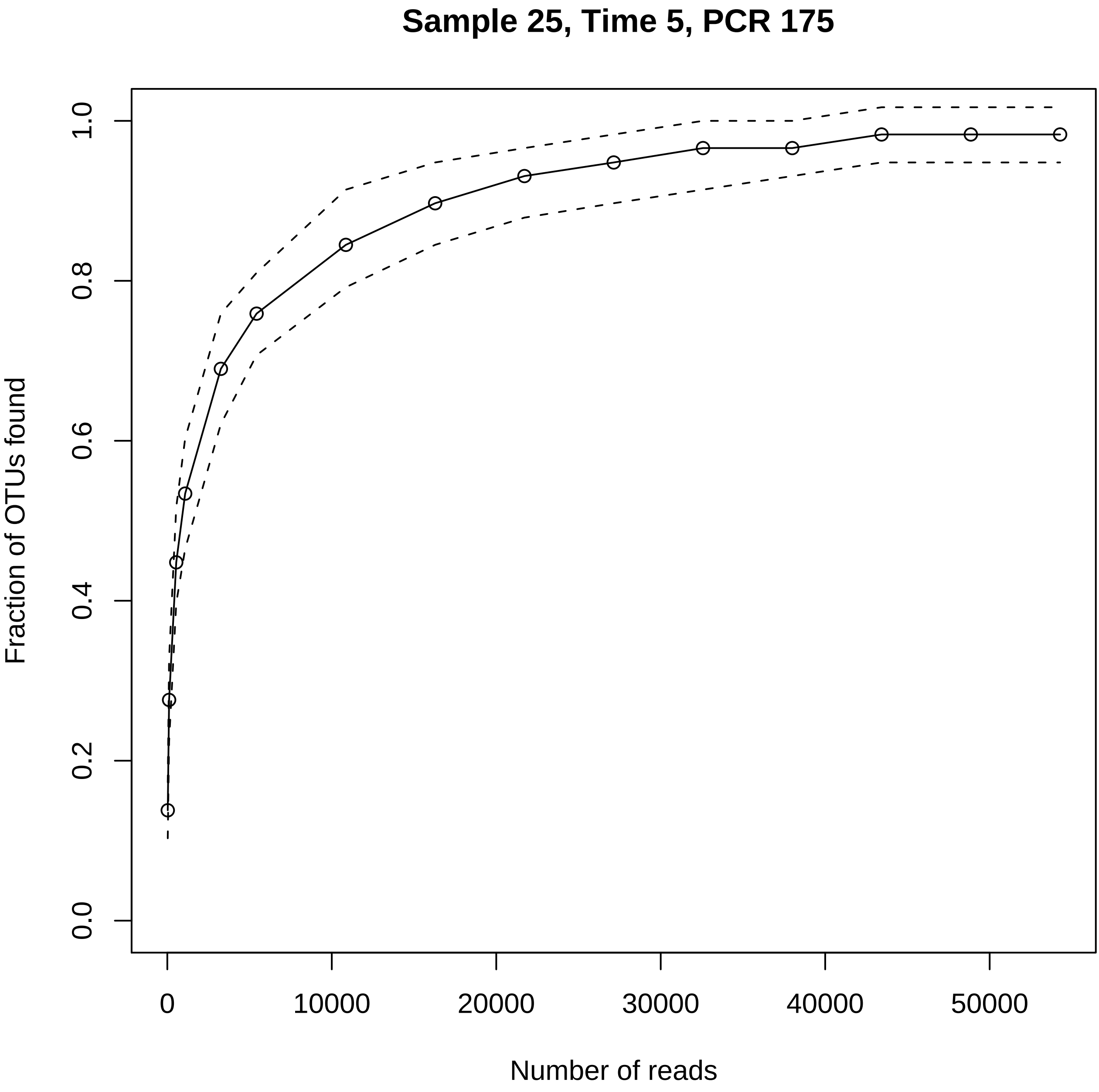
Sample 25, Time 5, PCR 175
Fraction of OTUs found
0.0
0.2
0.4
0.6
0.8
1.0
0
10000
20000
30000
40000
50000
Number of reads

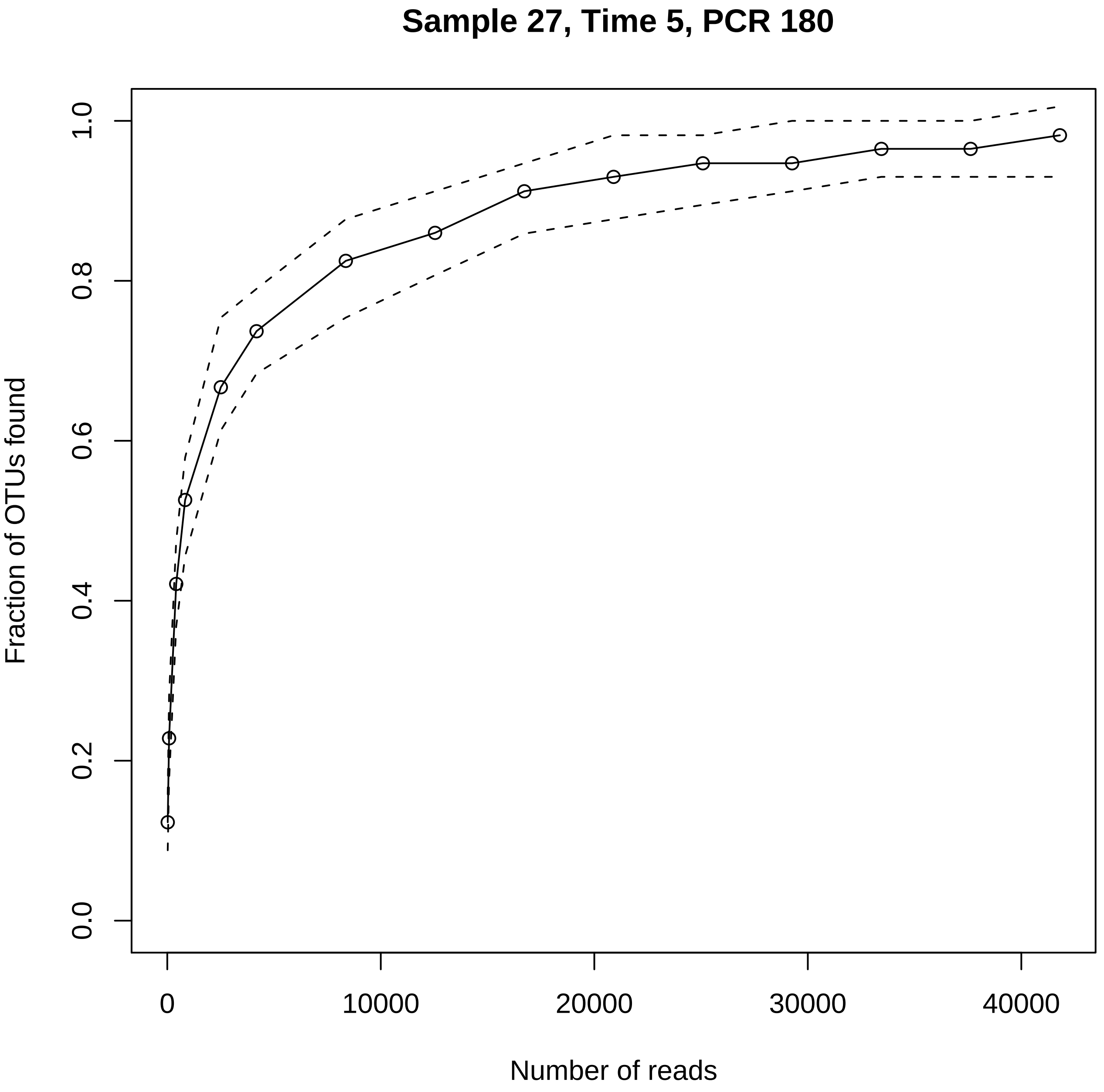
Sample 27, Time 5, PCR 180
Fraction of OTUs found
0.0
0.2
0.4
0.6
0.8
1.0
0
10000
20000
30000
40000
Number of reads

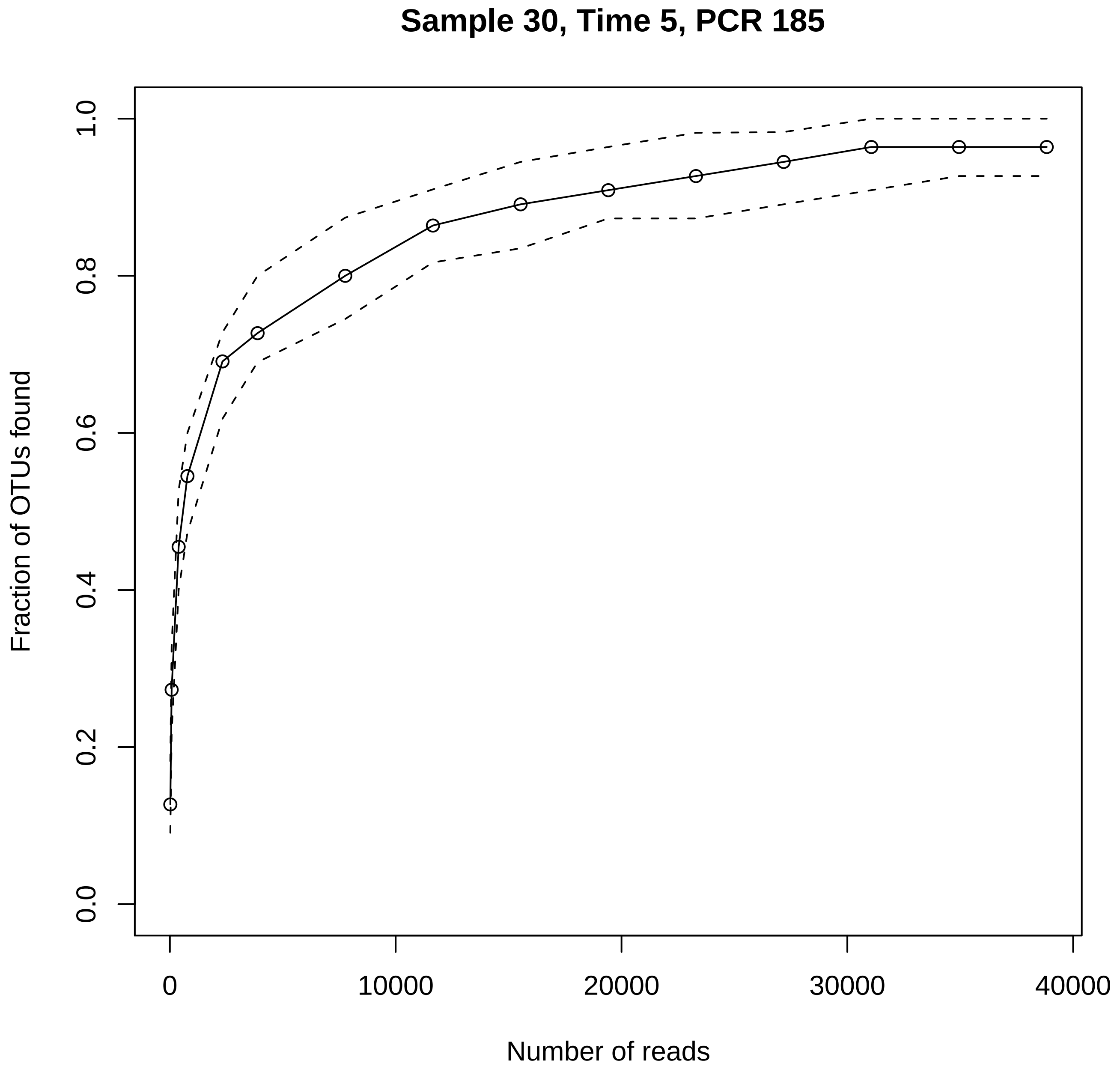
Sample 30, Time 5, PCR 185
Fraction of OTUs found
0.0
0.2
0.4
0.6
0.8
1.0
0
10000
20000
30000
40000
Number of reads

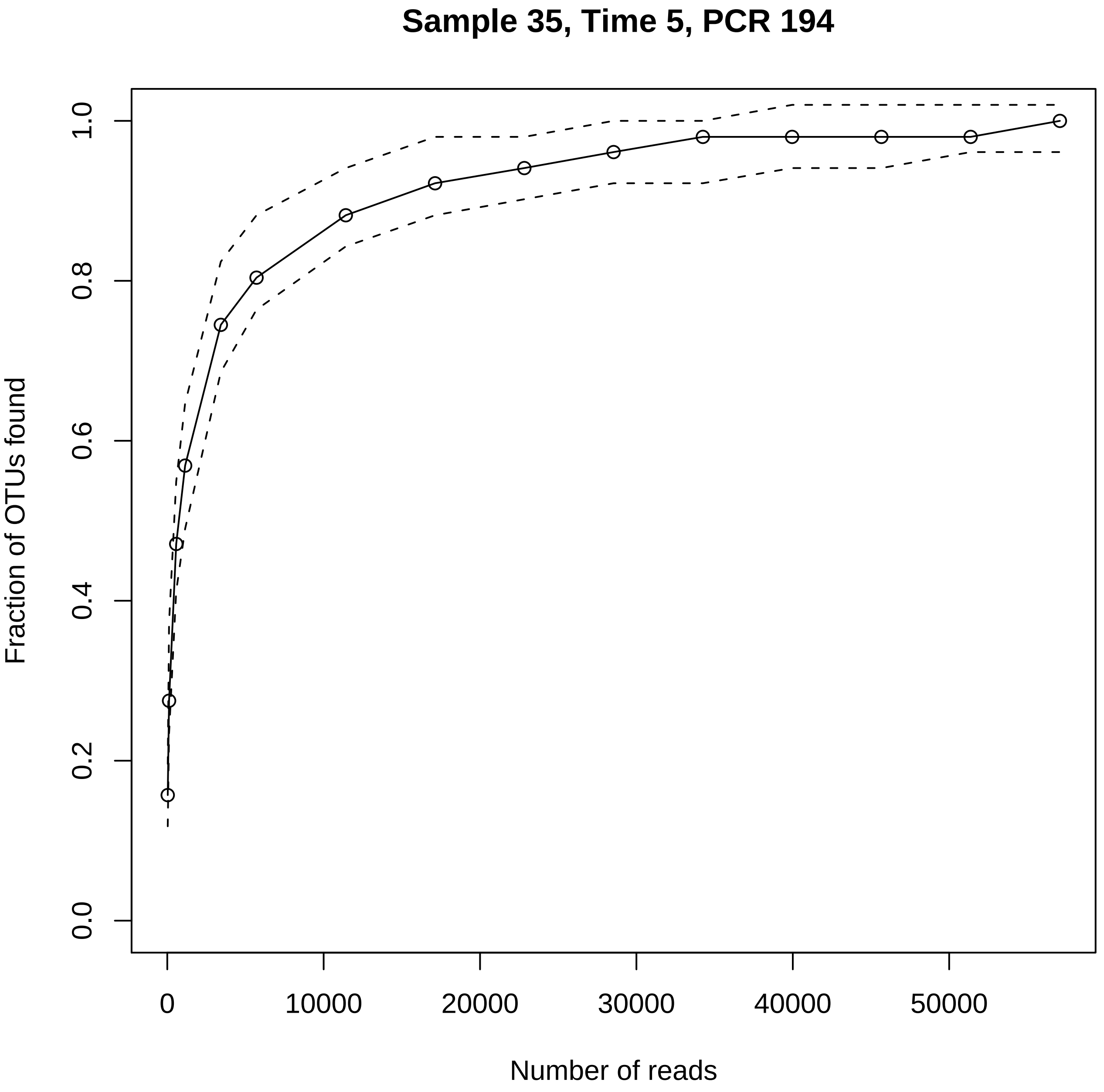
Sample 35, Time 5, PCR 194
Fraction of OTUs found
0.0
0.2
0.4
0.6
0.8
1.0
0
10000
20000
30000
40000
50000
Number of reads

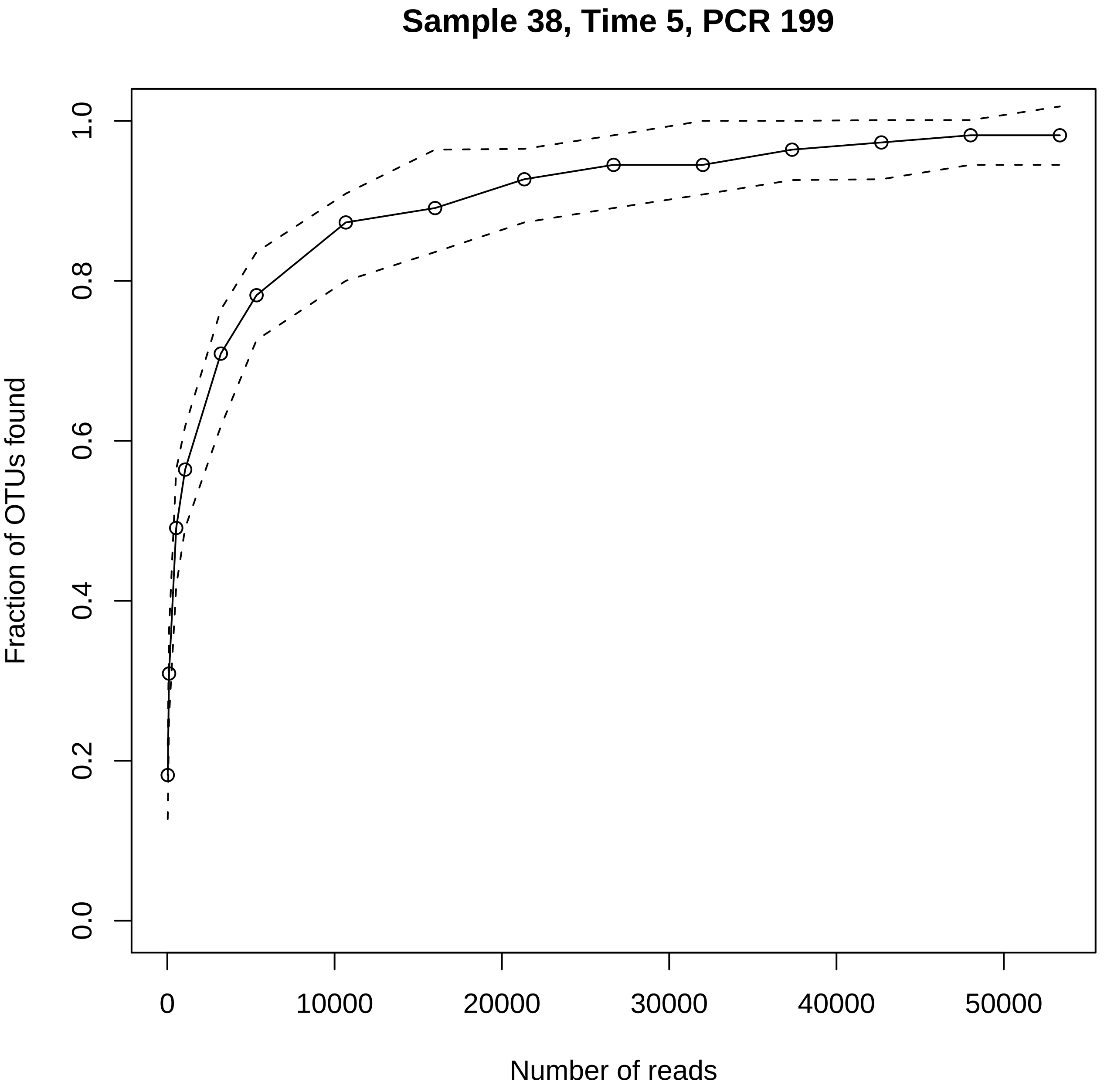

Sample 38, Time 5, PCR 199
Fraction of OTUs found
0.0
0.2
0.4
0.6
0.8
1.0
0
10000
20000
30000
40000
50000
Number of reads

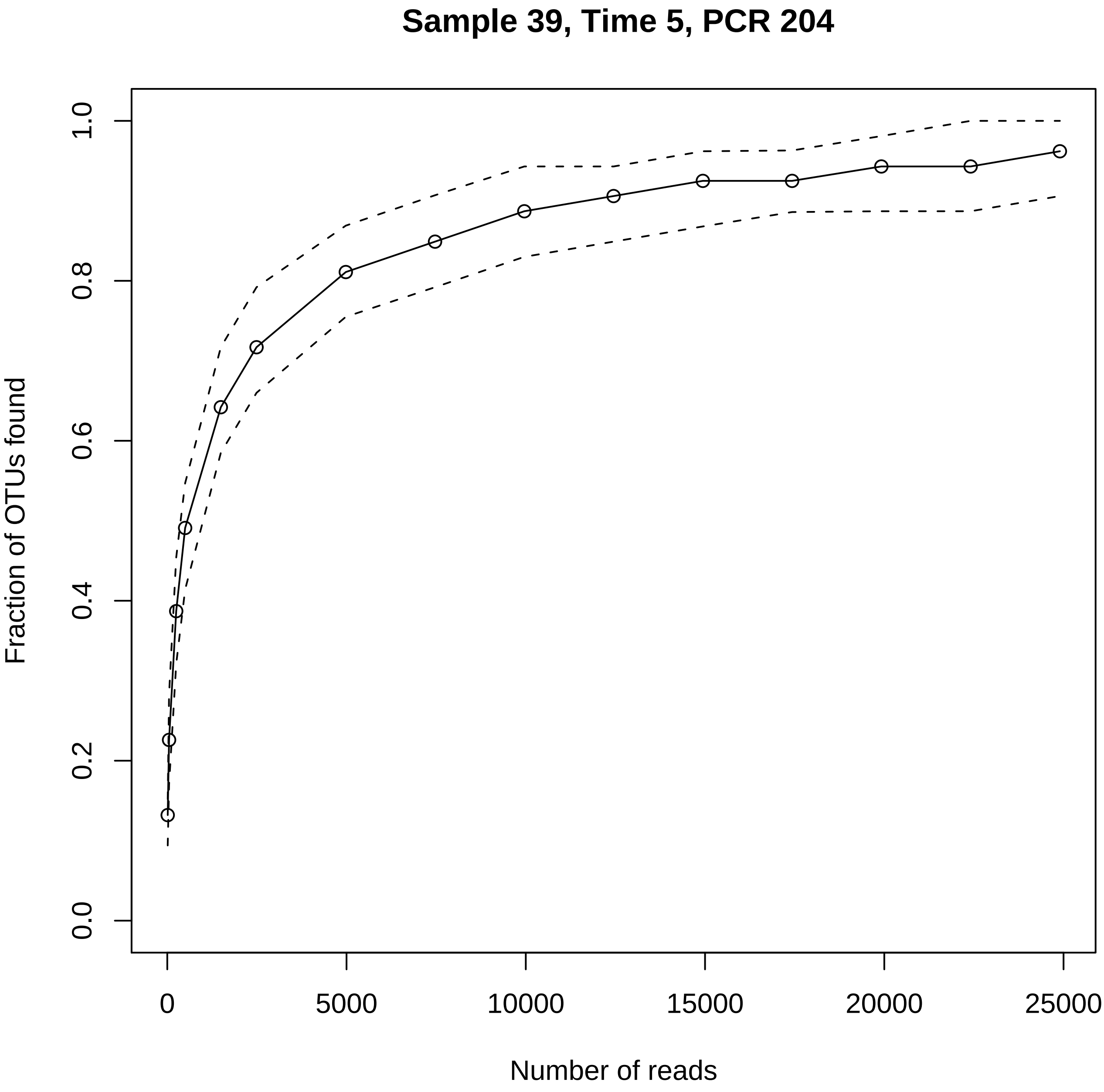
Sample 39, Time 5, PCR 204
Fraction of OTUs found
0.0
0.2
0.4
0.6
0.8
1.0
0
5000
10000
15000
20000
25000
Number of reads

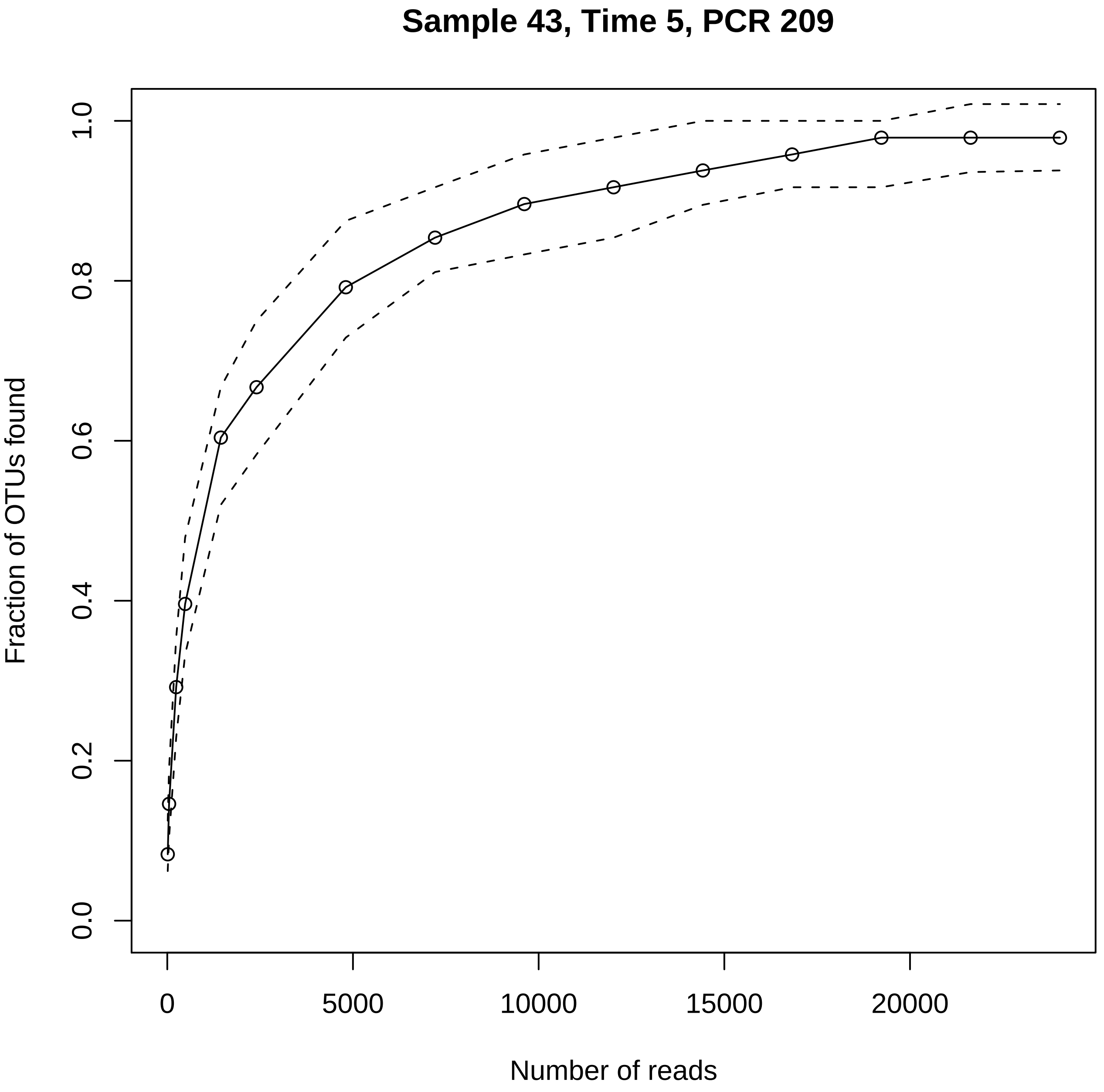
Sample 43, Time 5, PCR 209
Fraction of OTUs found
0.0
0.2
0.4
0.6
0.8
1.0
0
5000
10000
15000
20000
Number of reads

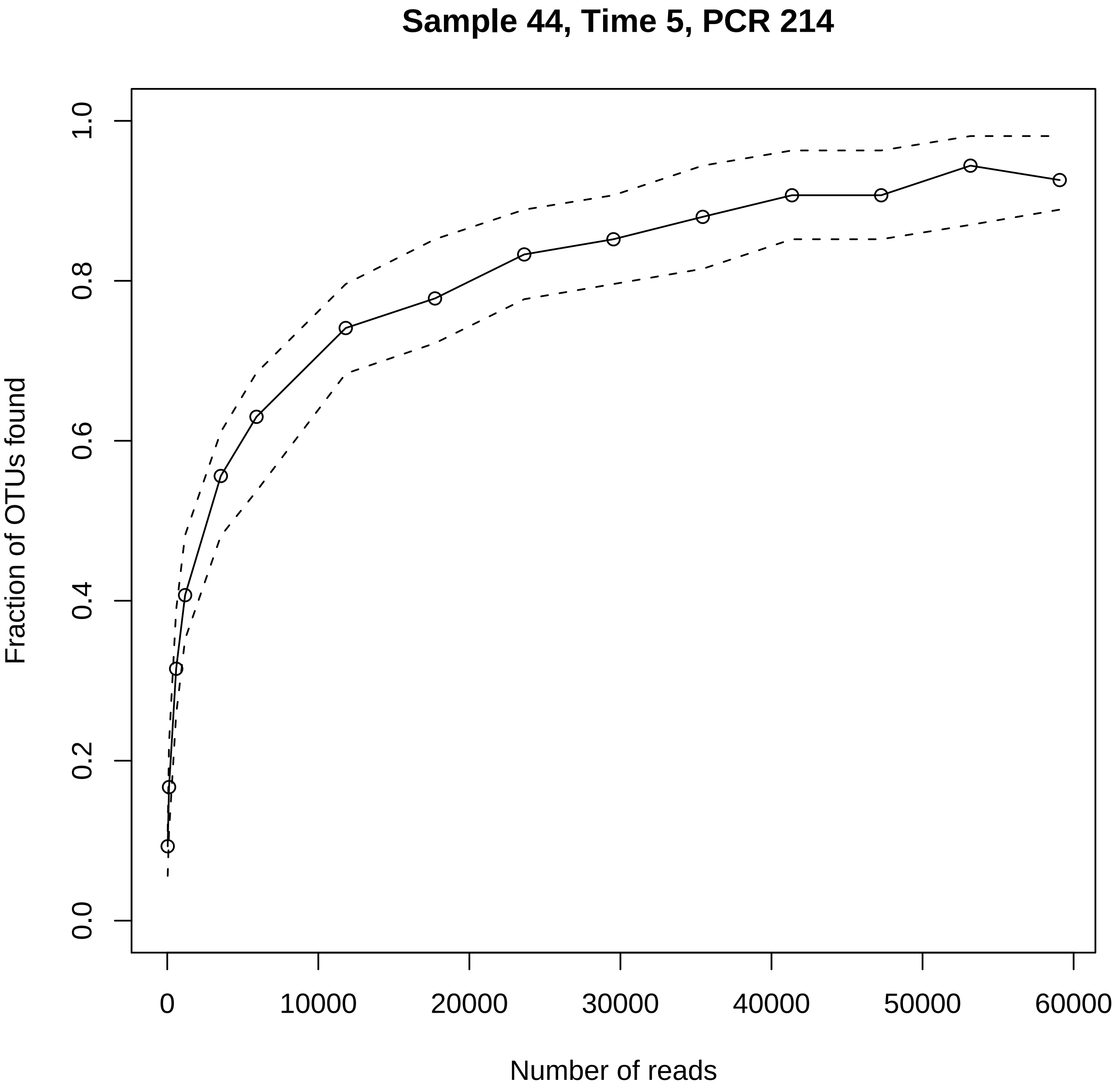

Sample 44, Time 5, PCR 214
Fraction of OTUs found
0.0
0.2
0.4
0.6
0.8
1.0
0
10000
20000
30000
40000
50000
60000
Number of reads

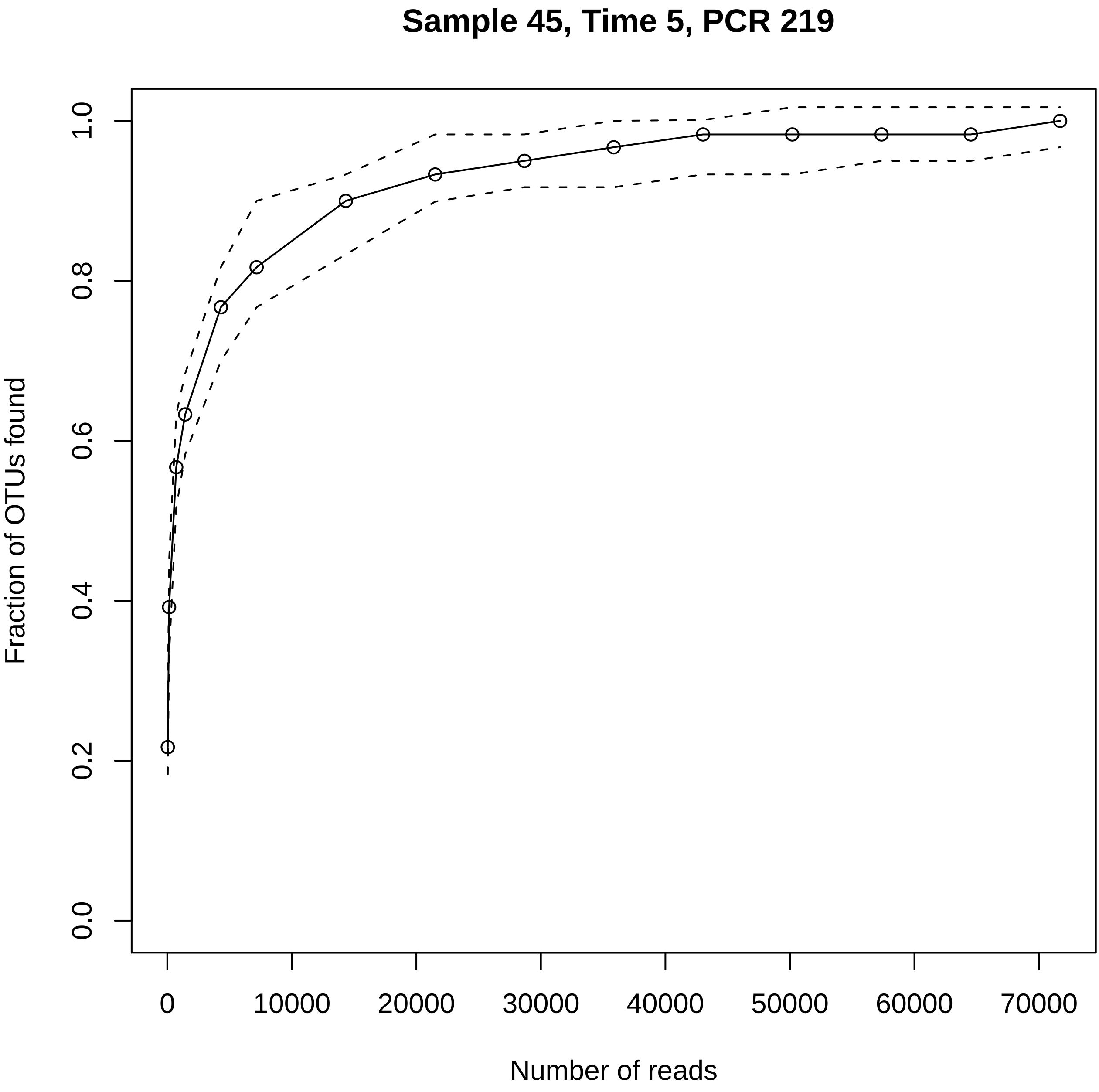

Sample 45, Time 5, PCR 219
Fraction of OTUs found
0.0
0.2
0.4
0.6
0.8
1.0
0
10000
20000
30000
40000
50000
60000
70000
Number of reads

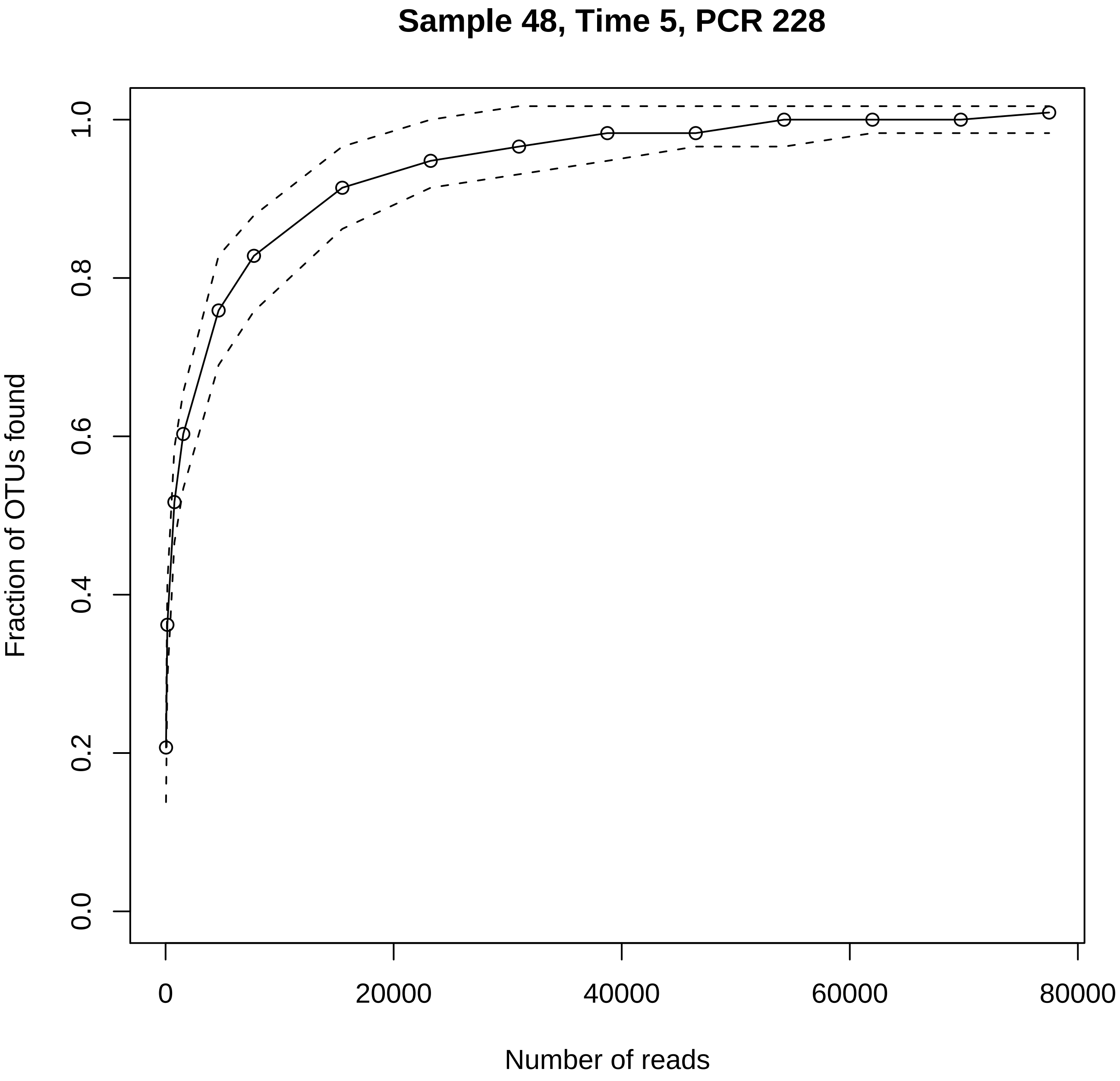

Sample 48, Time 5, PCR 228
Fraction of OTUs found
0.0
0.2
0.4
0.6
0.8
1.0
0
20000
40000
60000
80000
Number of reads

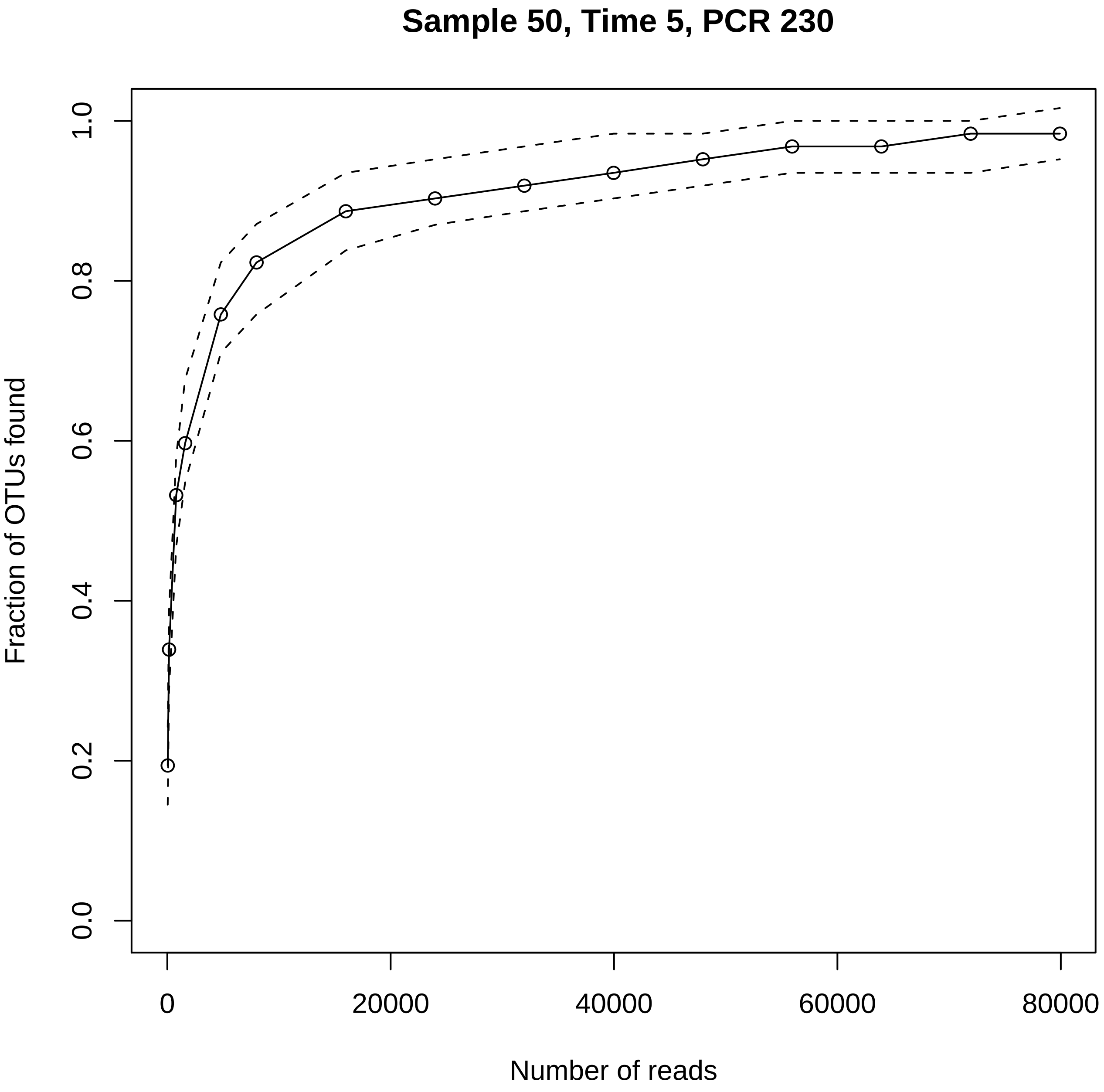
Sample 50, Time 5, PCR 230
Fraction of OTUs found
0.0
0.2
0.4
0.6
0.8
1.0
0
20000
40000
60000
80000
Number of reads

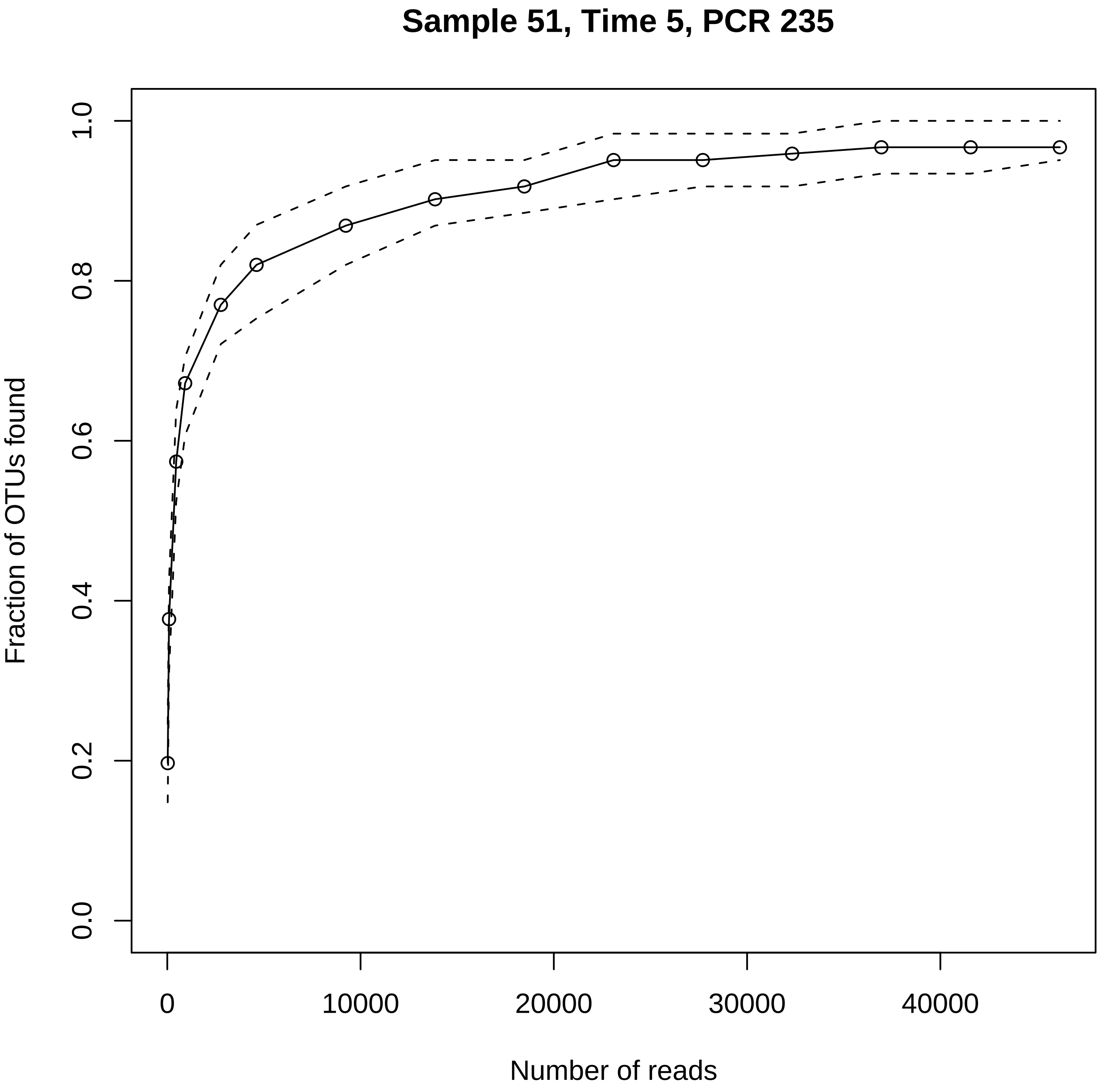
Sample 51, Time 5, PCR 235
Fraction of OTUs found
0.0
0.2
0.4
0.6
0.8
1.0
0
10000
20000
30000
40000
Number of reads

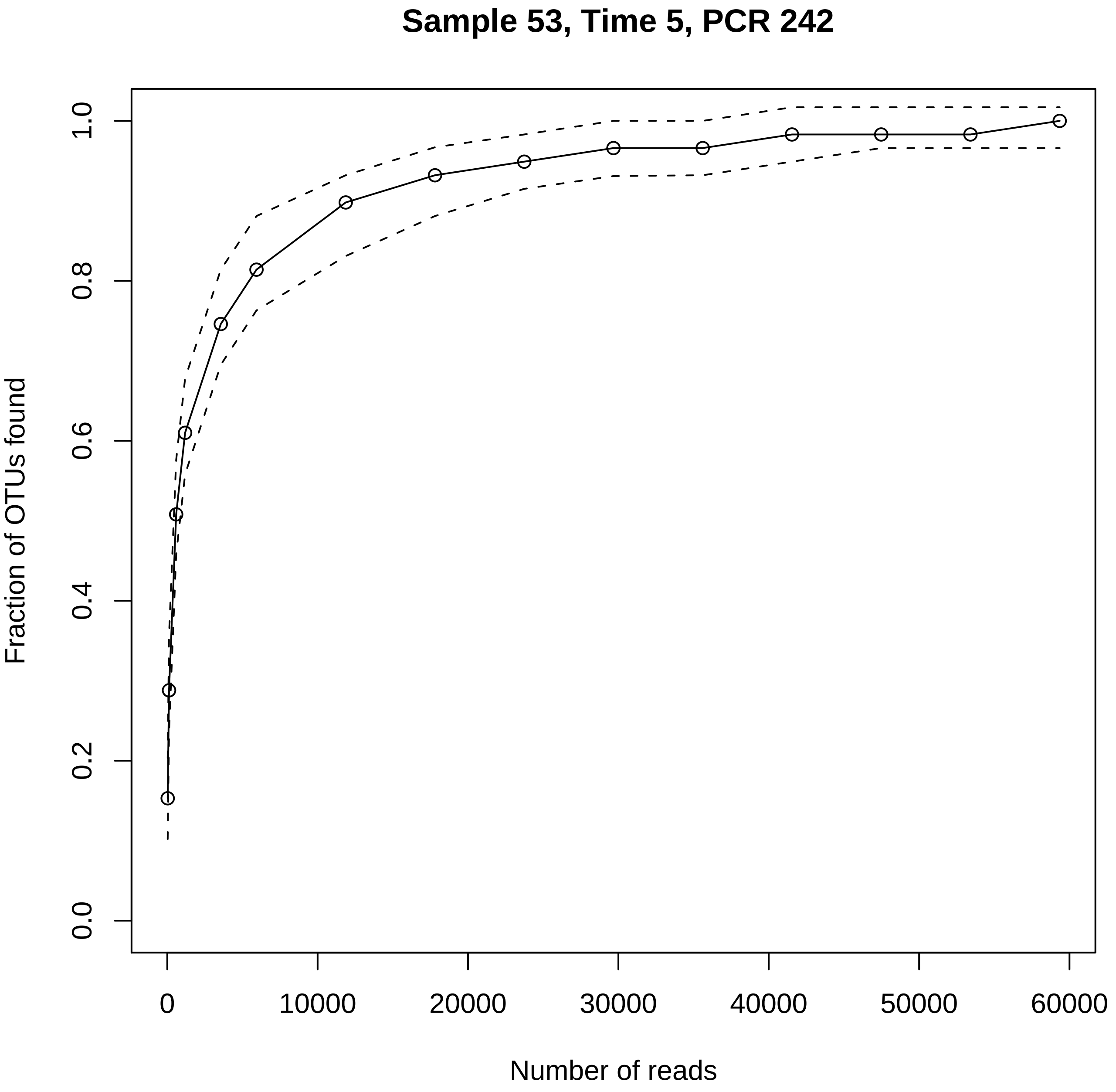
Sample 53, Time 5, PCR 242
Fraction of OTUs found
0.0
0.2
0.4
0.6
0.8
1.0
0
10000
20000
30000
40000
50000
60000
Number of reads

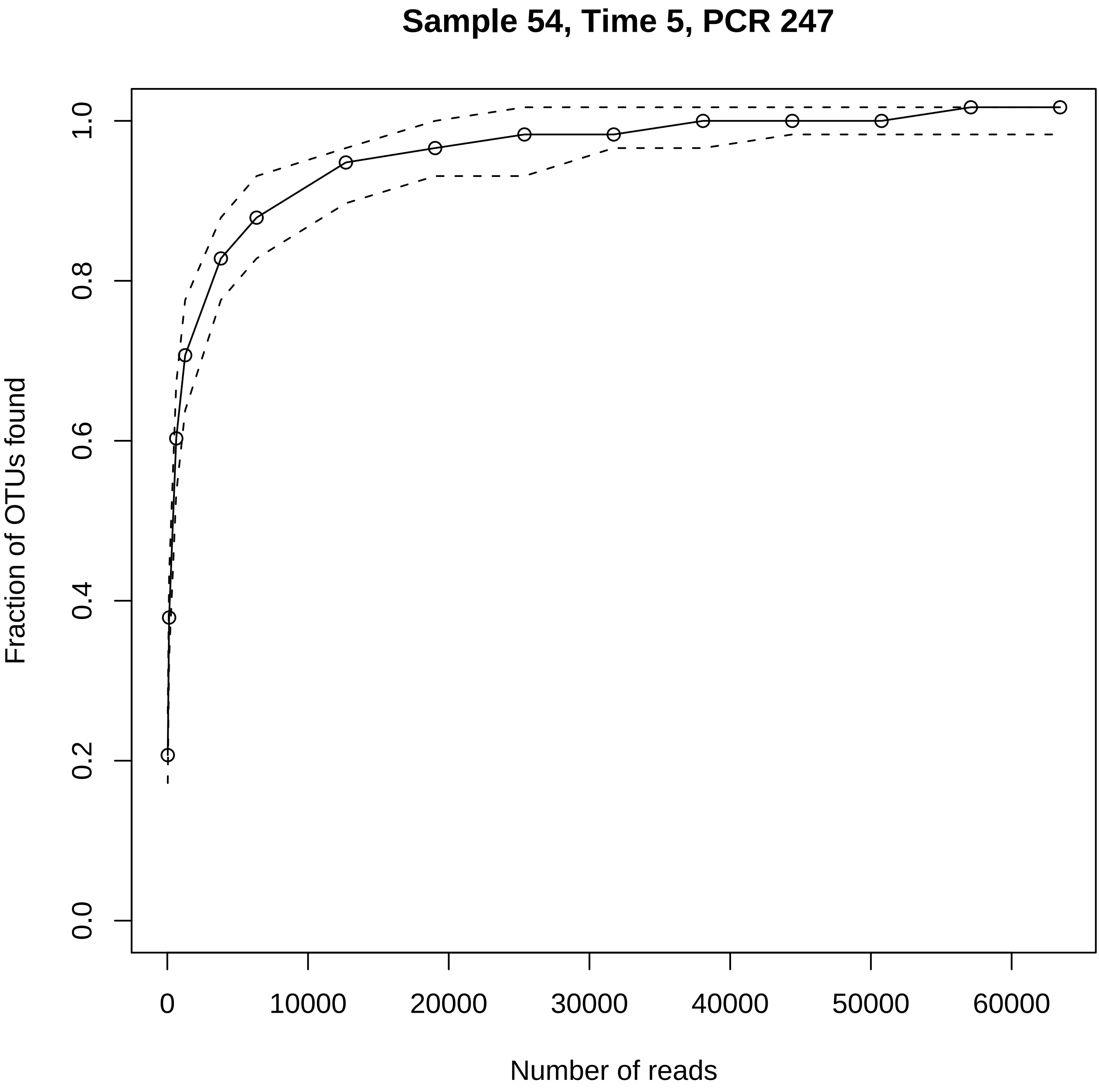

Sample 54, Time 5, PCR 247
Fraction of OTUs found
0.0
0.2
0.4
0.6
0.8
1.0
0
10000
20000
30000
40000
50000
60000
Number of reads

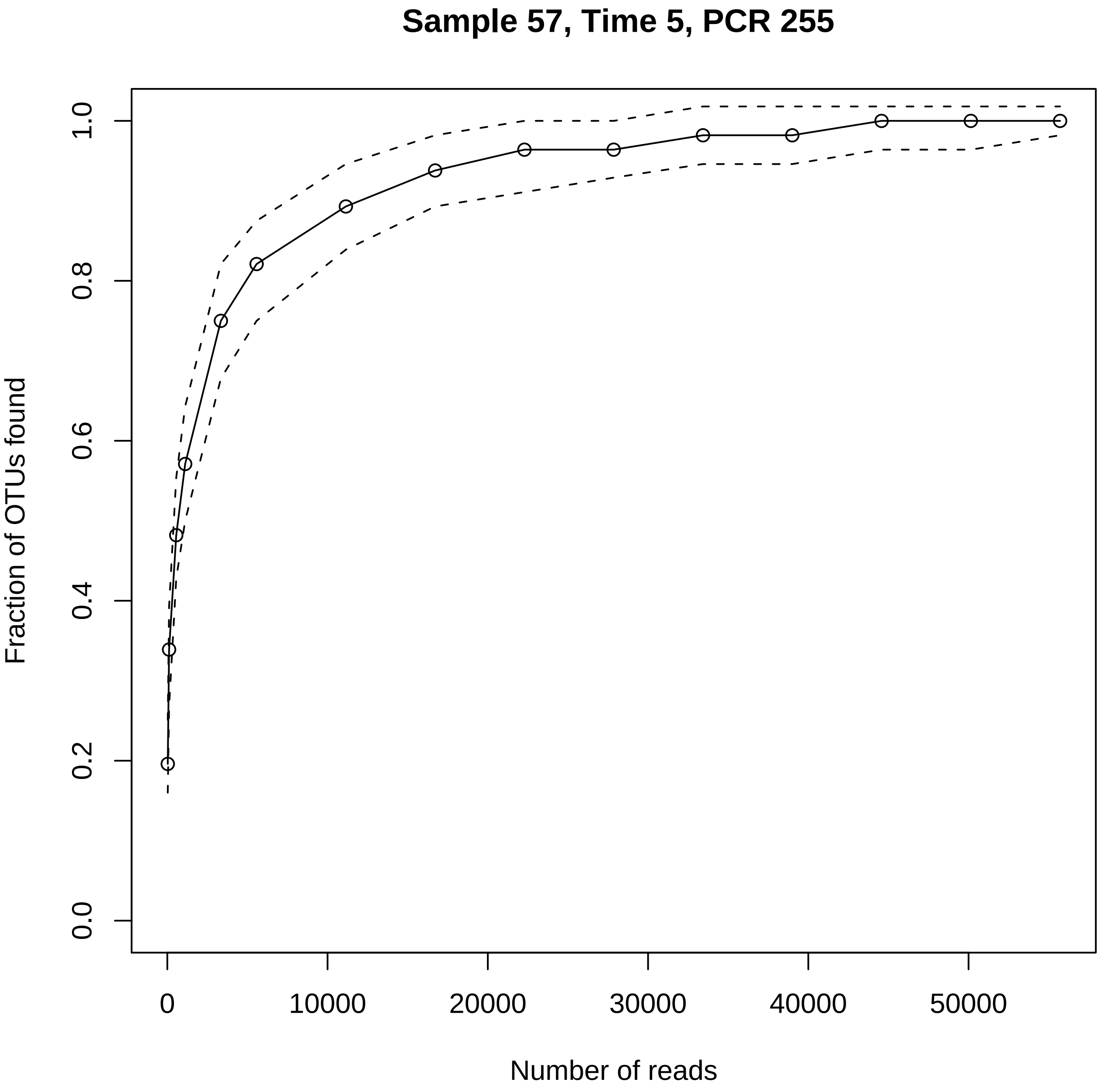

Sample 57, Time 5, PCR 255
Fraction of OTUs found
0.0
0.2
0.4
0.6
0.8
1.0
0
10000
20000
30000
40000
50000
Number of reads

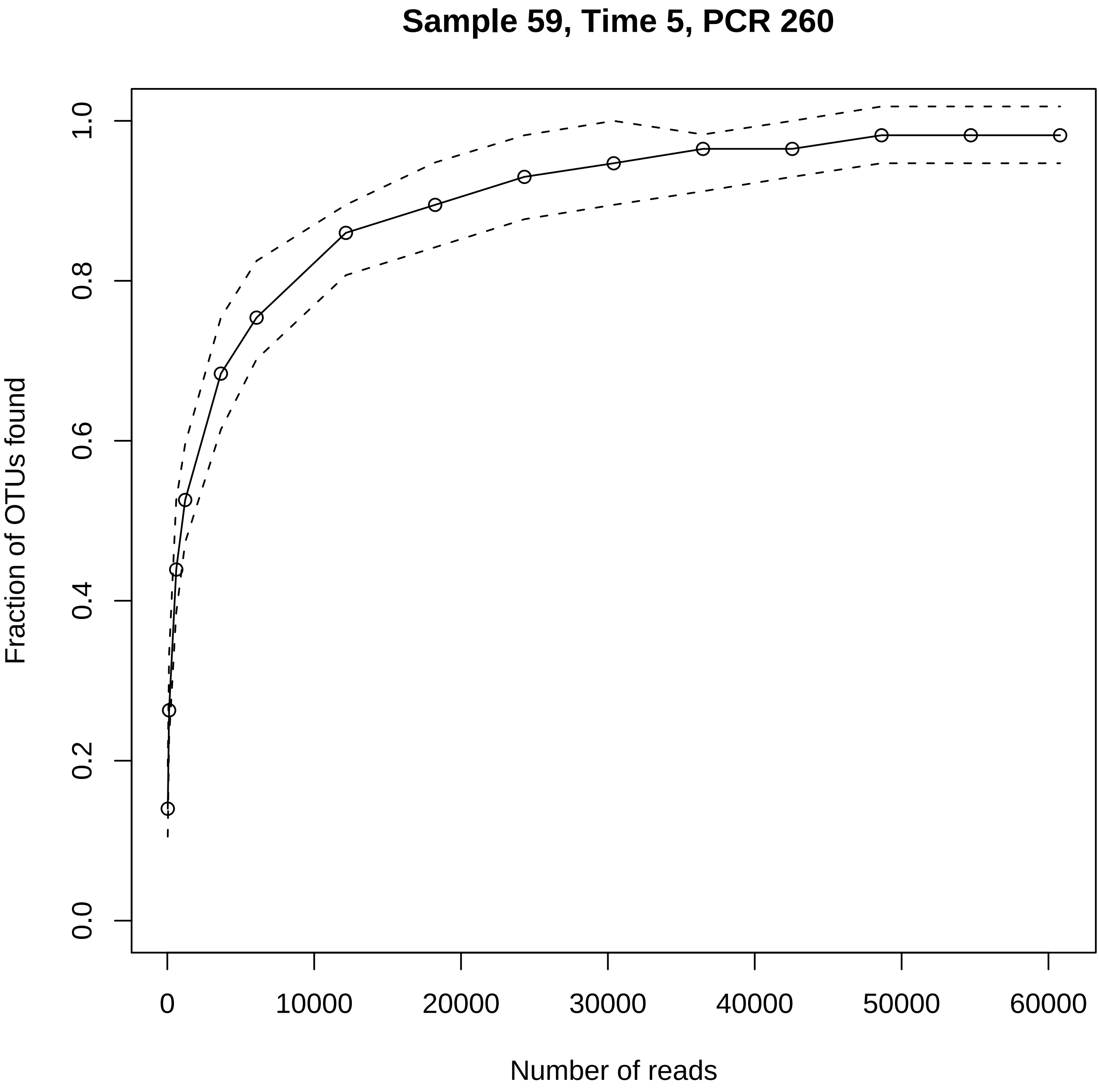

Sample 59, Time 5, PCR 260
Fraction of OTUs found
0.0
0.2
0.4
0.6
0.8
1.0
0
10000
20000
30000
40000
50000
60000
Number of reads

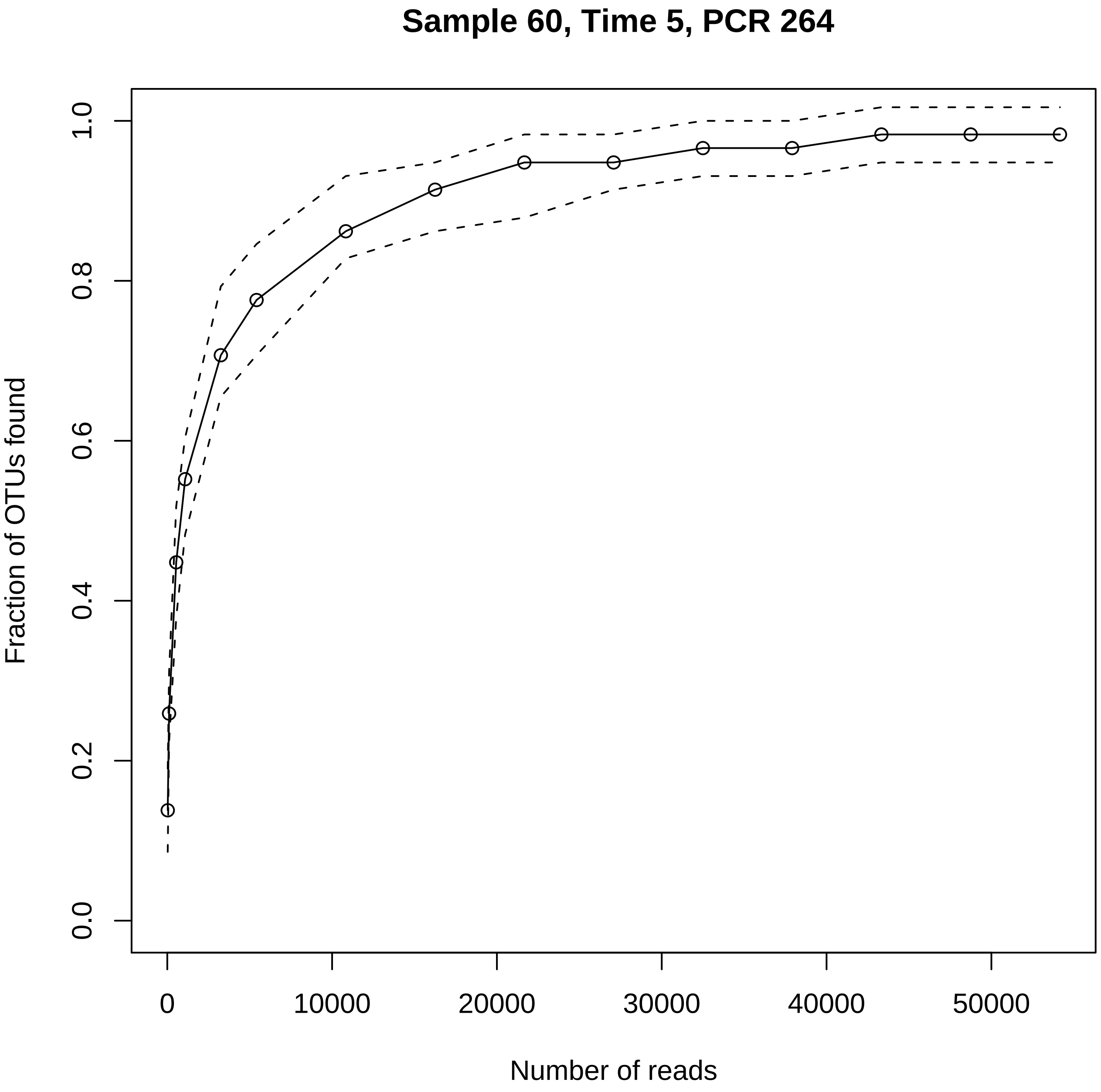

Sample 60, Time 5, PCR 264
Fraction of OTUs found
0.0
0.2
0.4
0.6
0.8
1.0
0
10000
20000
30000
40000
50000
Number of reads

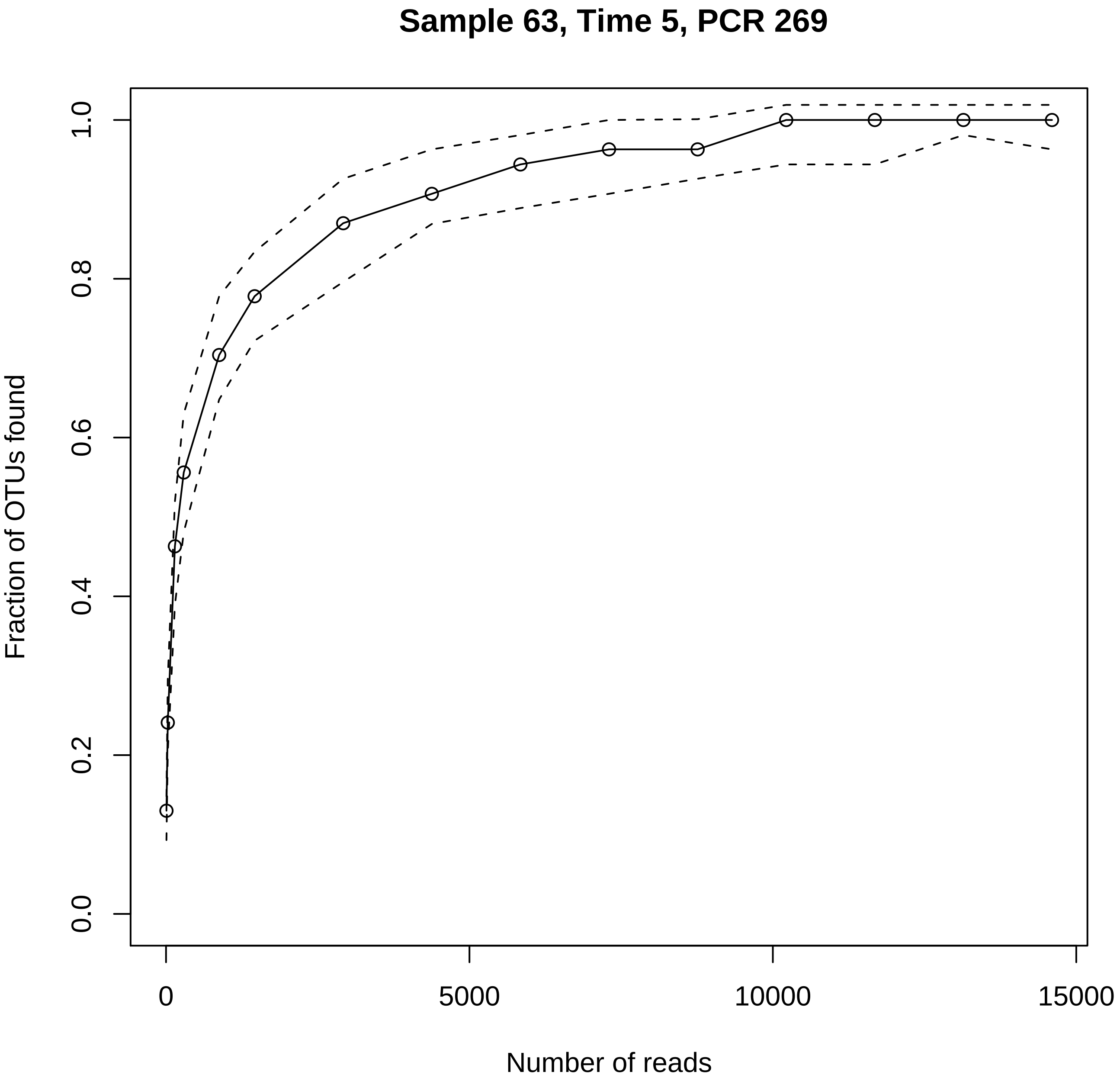

Sample 63, Time 5, PCR 269
Fraction of OTUs found
Number of reads
0.0
0.2
0.4
0.6
0.8
1.0
0
5000
10000
15000
