# Supplementary material for: Microbiome Profiling by Illumina Sequencing of Combinatorial Sequence-Tagged PCR Products
Source: PLoS One. 2010 Oct 26;5(10):e15406. doi: 10.1371/journal.pone.0015406 (PMC2964327; doi:10.1371/journal.pone.0015406)
Supplement: Table S3 — Comparison of organisms identified by DGGE or by Illumina sequencing in selected samples. (PDF) [file pone.0015406.s005.pdf]

	DGGE				Illumina
ID#	Organism Detected	Accession #	Coverage	Identity	Abundance
21	*L. crispatus*	AY335496.1	100%	91%	29%
	**L. iners*				40%
50	**G. vaginalis*				50%
54	**L. iners*				56%
63	**L. iners*				69%
71	**L. crispatus*				25%
	**L. iners*				55%
86	*L. iners*	AY283272.1	100%	98%	78%
	**G. vaginalis*				11%
6	*L. iners*	AY283272.1	100%	99%	60%
	**G. vaginalis*				21%
18	**L. iners*				54%
	G. vaginalis	CP001849.1	94%	100%	30%
49	*Streptococcus anginosus*	GU415393.1	99%	94%	28%
75	*L. iners*	AY283272.1	96%	97%	46%
	**G. vaginalis*				33%
92	*Haemophilus haemolyticus*	GU561423.1	98%	100%	15%
	**L. iners*				18%
	G. vaginalis	EF194095.1	99%	97%	27%
40	*Leptotrichia amnionii*	EF218612.1	99%	98%	15%
	**L. iners*				18%
	G. vaginalis	EF194095.1	100%	95%	23%
48	**L. crispatus*				13%
	**L. iners*				22%
	G. vaginalis	CP001849.1	99%	97%	29%
57	*L. iners*	AY283272.1	99%	98%	55%
	L. iners	AY283272.1	99%	98%	55%
	G. vaginalis	EF194095.1	99%	97%	20%
60	*G. vaginalis*	CP001849.1	98%	95%	24%
67	*Streptococcus agalactiae*	AB297817.1	100%	97%	27%
	G. vaginalis	EF194095.1	99%	97%	54%
73	*Leptotrichia amnionii*	EF218612.1	100%	95%	2%
	G. vaginalis	EF194095.1	97%	98%	34%
83	*G. vaginalis*	CP001849.1	100%	96%	23%
89	*Prevotella amniotica*	AM422125.1	100%	91%	2%
	**L. iners*				19%
	**G. vaginalis*				42%
90	*G. vaginalis*	CP001849.1	100%	97%	60%

*Determined via band location on DGGE gel
